# Using genetics and proteomics data to identify proteins causally related to COVID-19, healthspan and lifespan: a Mendelian randomization study

**DOI:** 10.18632/aging.205711

**Published:** 2024-04-03

**Authors:** Jie V. Zhao, Minhao Yao, Zhonghua Liu

**Affiliations:** 1 School of Public Health, Li Ka Shing Faculty of Medicine, The University of Hong Kong, Hong Kong SAR, China; 2 State Key Laboratory of Pharmaceutical Biotechnology, The University of Hong Kong, Hong Kong SAR, China; 3 Department of Statistics and Actuarial Science, The University of Hong Kong, Hong Kong SAR, China; 4 Department of Biostatistics, Mailman School of Public Health, Columbia University, New York, NY 10032, USA

**Keywords:** proteomics, healthspan, lifespan, COVID-19

## Abstract

Background: COVID-19 pandemic poses a heavy burden on public health and accounts for substantial mortality and morbidity. Proteins are building blocks of life, but specific proteins causally related to COVID-19, healthspan and lifespan have not been systematically examined.

Methods: We conducted a Mendelian randomization study to assess the effects of 1,361 plasma proteins on COVID-19, healthspan and lifespan, using large GWAS of severe COVID-19 (up to 13,769 cases and 1,072,442 controls), COVID-19 hospitalization (32,519 cases and 2,062,805 controls) and SARS-COV2 infection (122,616 cases and 2,475,240 controls), healthspan (*n* = 300,477) and parental lifespan (~0.8 million of European ancestry).

Results: We identified 35, 43, and 63 proteins for severe COVID, COVID-19 hospitalization, and SARS-COV2 infection, and 4, 32, and 19 proteins for healthspan, father’s attained age, and mother’s attained age. In addition to some proteins reported previously, such as SFTPD related to severe COVID-19, we identified novel proteins involved in inflammation and immunity (such as ICAM-2 and ICAM-5 which affect COVID-19 risk, CXCL9, HLA-DRA and LILRB4 for healthspan and lifespan), apoptosis (such as FGFR2 and ERBB4 which affect COVID-19 risk and FOXO3 which affect lifespan) and metabolism (such as PCSK9 which lowers lifespan). We found 2, 2 and 3 proteins shared between COVID-19 and healthspan/lifespan, such as CXADR and LEFTY2, shared between severe COVID-19 and healthspan/lifespan. Three proteins affecting COVID-19 and seven proteins affecting healthspan/lifespan are targeted by existing drugs.

Conclusions: Our study provided novel insights into protein targets affecting COVID-19, healthspan and lifespan, with implications for developing new treatment and drug repurposing.

## INTRODUCTION

Increasing lifespan and promoting healthy living into old age are among top priorities of health care systems all over the world. Although lifespan is steadily improving, an increasing proportion of the population is affected by multiple chronic diseases. Moreover, in recent years, the global COVID-19 pandemic poses a heavy burden on public health and accounts for substantial mortality and morbidity [1, 2]. According to the estimation of World Health Organization, around 14.83 million excess deaths globally were due to COVID-19 [1].

Proteins are the building blocks of life, important for etiology of disease and often used as drug targets for treatment. However, the specific proteins involved in the complex ageing process and COVID-19 risk are largely unclear. The dysregulation in inflammatory and immunomodulatory response has been thought to play a key role in multiple diseases and disorders [3], as well as in COVID-19 [4]. Several proteins related to inflammation and immune function, such as CXCL14 [5] and soluble HLA-G (sHLA-G) [6], have been identified by comparing patients with and without COVID-19. However, these associations may be confounded by some other characteristics, such as socioeconomic position. Moreover, proteins in other pathways, such as cardiometabolism, may also affect COVID-19 and longevity. Therefore, a comprehensive examination of proteins using advanced causal inference methods robust to confounding is needed.

Mendelian randomization (MR) uses genetic variants as instrument variables (IVs) to assess the causal role of proteins in COVID-19 and lifespan. As the genotypes are determined at conception and randomly allocated, MR estimates are thus not subject to residual confounding [7]. Using MR, some studies explored the associations of proteins with COVID-19 [8, 9] or with healthspan [10], but not both. In addition, the genetic instruments of these proteins are generally based on relatively smaller genome-wide association study (GWAS) (~3,301 people) [8–10]. Recently, the UK Biobank Pharma Proteomics Project (UKB-PPP), a collaboration between the UK Biobank (UKB) and thirteen biopharmaceutical companies has measured the plasma proteomic profiles in 54,306 UKB participants. In this study, we conducted MR analysis using genetic instruments from much larger GWAS of proteins and applied to large GWAS of COVID-19 (severe COVID-19, COVID-19 hospitalization and SARS-COV2 infection), healthspan and lifespan (mother’s and father’s attained age), to identify proteins causally related to COVID-19, healthspan and lifespan. We included both COVID-19 and healthspan and lifespan in the outcome, because COVID-19 which occurred in recent years reflects a new threat to longevity, whilst healthspan and lifespan reflect overall morbidity and mortality.

## METHODS

### Study design

**Figure 1:**
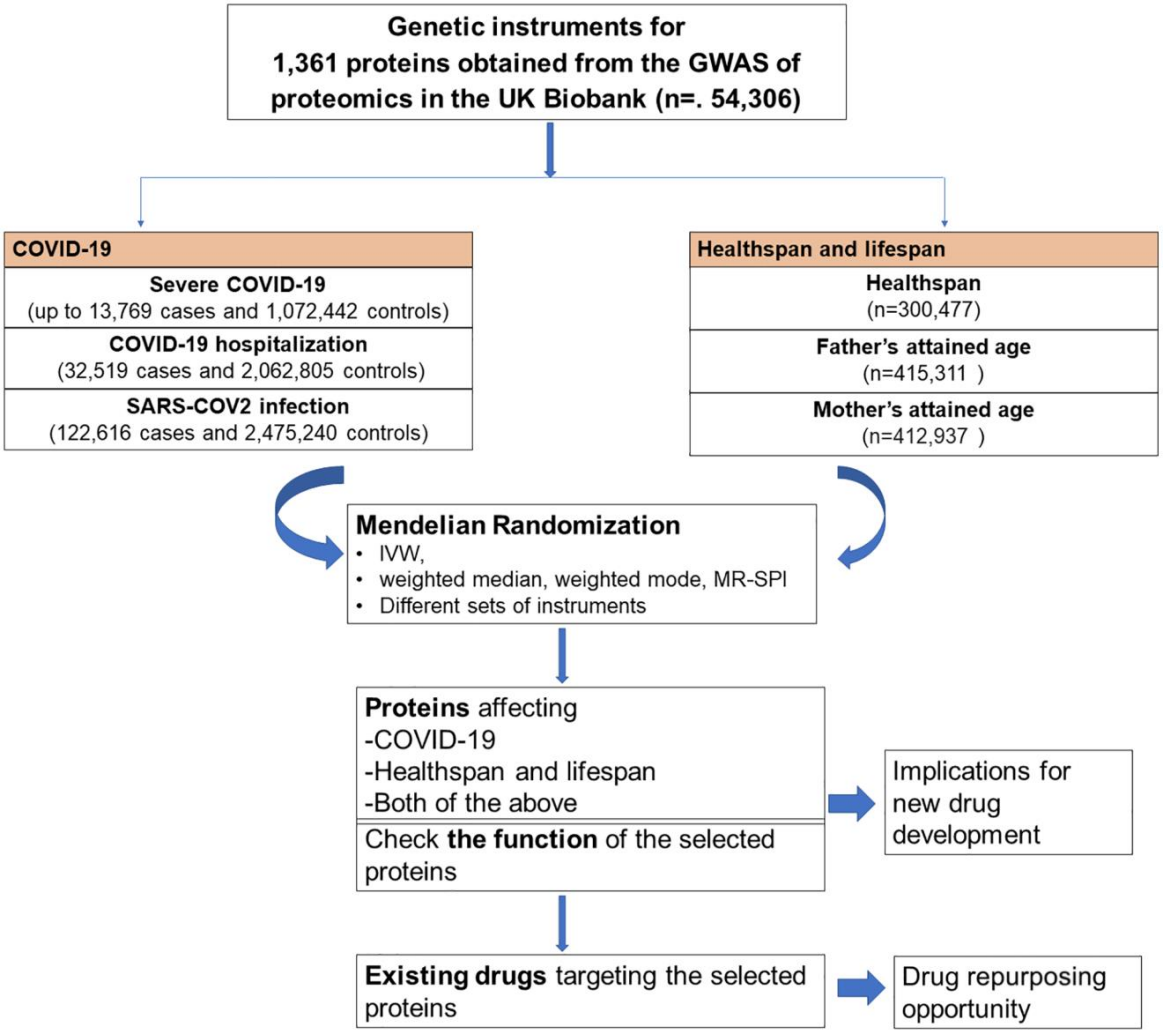
Flow chart of the study design.

We used proteome-wide MR study to identify proteins causally related to COVID-19, healthspan and lifespan. To better understand the function of these proteins, we checked the function of each selected protein. To assess the drug repurposing opportunity, we also checked whether these proteins are targeted by existing drugs. The flow chart of the study design was shown in Figure 1.

### Proteomics data from UK Biobank

The proteomic profiling measured 1,472 protein analytes and captured 1,463 unique proteins using the Olink Explore 1536 platform in 54,306 plasma samples in the UK Biobank. The blood samples were from a randomised subset of 46,673 UKB participants at baseline visit, 6,385 individuals at baseline selected by the UKB-PPP consortium and 1,268 individuals who participated in the COVID-19 repeat imaging study at multiple visits. No batch effects, plate effects or abnormalities in protein coefficients of variation were observed [11]. We used significant (*p* < 3.4 × 10^−11^) and independent (r^2^ < 0.01) single nucleotide polymorphisms (SNPs) as instruments provided by the study of Sun et al. [1]. The details of the samples and sample selection in UKB-PPP were shown in Supplementary Methods. Up to 1,361 proteins with genetic instruments available in at least one of the outcome datasets were included in the analysis. The genetic instruments for proteins used in the MR analysis are provided in Supplementary Table 1.

### Genetic associations with COVID-19

We obtained genetic associations of these genetic instruments with severe COVID-19 (up to 13,769 cases and 1,072,442 controls), hospitalization (up to 32,519 cases and 2,062,805 controls) and SARS-COV2 infection (up to 122,616 cases and 2,475,240 controls), from the latest GWAS results provided by the *COVID-19 host genetics initiative* [12]. Severe COVID-19 was defined as (1) hospitalization with laboratory confirmed SARS-CoV-2 infection based on RNA and/or serology and (2) hospitalization with COVID19 as the primary reason for admission, and (3) followed by death or respiratory support. COVID-19 hospitalization was defined as hospitalization with laboratory confirmed SARS-CoV-2 infection due to corona-related symptoms. COVID-19 infection was defined as (1) laboratory confirmed SARS-CoV-2 infection (RNA and/or serology based), or (2) physician diagnosis of COVID-19, or (3) self-report as COVID-19 positive. The GWAS was adjusted for age, age square, sex and the interaction of age and sex [12].

### Genetic associations with healthspan and lifespan (mother’s and father’s attained age)

We obtained summary statistics for healthspan from a large GWAS conducted in 300,477 people of British ancestry in the UK Biobank. Healthspan was defined as the age of the first incidence of congestive Heart Failure (CHF), Myocardial Infarction (MI), Chronic Obstructive Pulmonary Disease (COPD), stroke, dementia, diabetes, cancer, and death [13]. The GWAS used Cox-Gompertz survival models, with adjustment for sex, the first genetic principal components variables, assessment centre and genotyping batch [13].

We obtained genetic associations with parental survival (attained age) from a GWAS of parental longevity in European descent UK Biobank participants (*n* = 415,311 for father’s attained age, *n* = 412,937 for mother’s attained age) [14]. The GWAS used a Cox proportional hazards model to estimate offspring genetic variant effects on parental survival, stratified by sex, and adjusted for age and 10 principal components of ancestry. As the effect sizes obtained using genetic data from offspring are half of the actual variant effect size in the parent, they were doubled to reflect the expected genetic effects in parents [14].

In both GWAS, the summary statistics from the GWAS were reported as log hazard ratios, for ease of understanding, these were converted to years of life by inverting the sign and multiplied by 10 [13, 14].

### Statistical analysis

MR estimates were based on the genetic association with each type of COVID-19 (severe COVID-19, COVID-19 hospitalization or SARS-COV2 infection) divided by the genetic association with each protein), i.e., the Wald ratio estimates. Similarly, we obtained the MR estimates for the association of each protein with healthspan and lifespan. The genetic variant specific estimates were meta-analysed using inverse variance weighting (IVW). Multiplicative random effects model was used when three or more genetic variants were used as instruments, and fixed effects model was used when less than three genetic variants were used as instruments. To account for multiple testing, we used Bonferroni corrected significance (*p*-value < 0.05/1,361 = 3.7 × 10^−5^) as the cut-off. Heterogeneity test was also conducted for the identified protein-outcome associations. In sensitivity analysis, for proteins with three or more genetic variants as instruments, we used different MR methods under different assumptions from IVW, including weighted median, weighted mode and MR-SPI. The weighted median method can provide consistent estimates even when up to 50% of the information comes from invalid genetic variants [15]. The weighted mode is based on the assumption that a plurality of genetic variants are valid instruments, i.e., no larger subset of invalid instruments estimating the same causal parameter than the subset of valid instruments exists [16]. Also based on the plurality assumption, MR-SPI can automatically select genetic variants as valid instruments and provide robust inference in finite samples [17]. Given that some proteins lack cis-SNPs as instruments, we used cis- and trans-SNPs as instruments in the main analysis and cis-SNPs in the sensitivity analysis.

### Identification of drug repurposing opportunities

To have a better understanding of the biological function of each identified protein, we also looked into their functions in STRING [18], a database with comprehensive information on protein network and function, and UNIPROT (https://www.uniprot.org/). To identify potential drug repurposing opportunity, we checked whether the proteins are targeted by currently available drugs, by searching in DrugBank, a publicly available resource with drug and drug target information on over 13,000 drugs (https://www.drugbank.ca/).

All statistical analysis were performed using R (Foundation for Statistical Computing, Vienna, Austria; Version 4.1.1) and “TwoSampleMR”, “MendelianRandomization”, “ggplot2”, “MR.SPI” R packages.

### Availability of data and materials

All the data used in the study are publicly available. The data sources have been specified in the methods.

## RESULTS

### Proteins causally related to COVID-19

**Figure 2:**
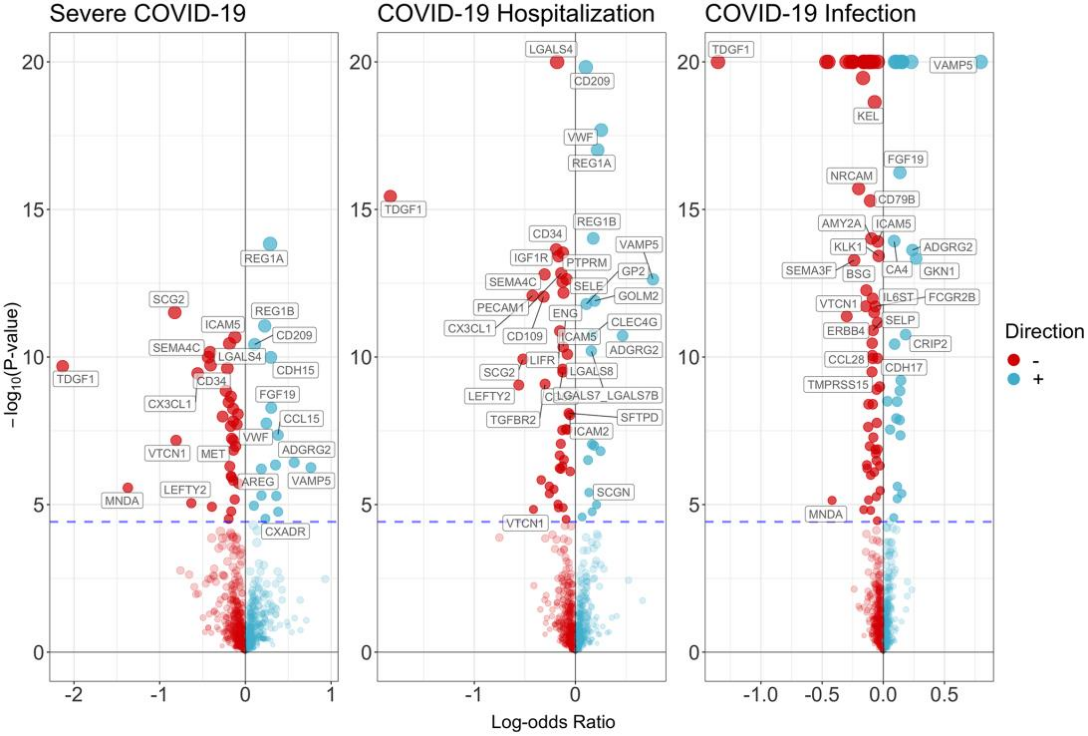
Volcano plot on the associations of proteins with severe COVID-19, COVID-19 hospitalization and SARS-COV2 infection.

**Figure 3:**
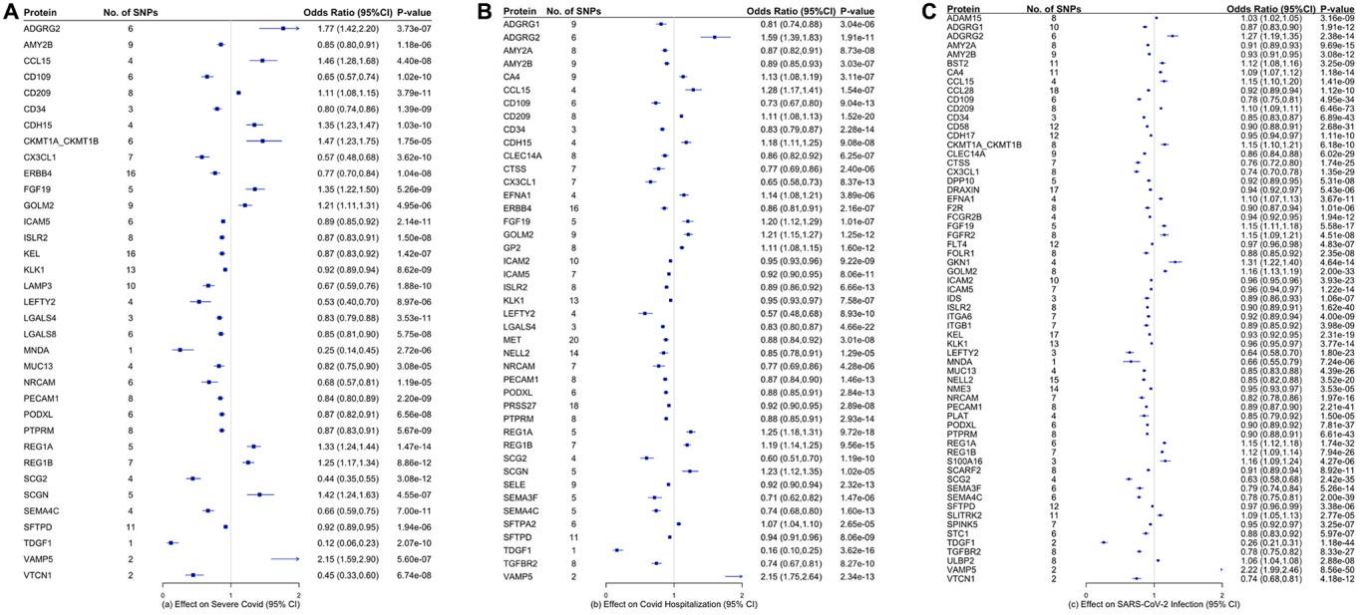
Proteins that are significantly associated with (**A**) severe COVID-19, (**B**) COVID-19 hospitalization, and (**C**) COVID-19 infection. The inverse-variance weighted (IVW) estimates are presented in log-odds ratio with the corresponding 95% confidence intervals.

Figure 2 and Supplementary Tables 2–4 showed the associations of all proteins with three types of COVID-19, healthspan and lifespan (mother’s and father’s attained age). Among these proteins, we selected proteins with Bonferroni-corrected significance. Figure 3 showed the selected proteins affecting the risk of severe COVID-19 (Figure 3A), COIVD-19 hospitalization (Figure 3B) and SARS-COV2 infection (Figure 3C). We identified 35 proteins associated with severe COVID (Figure 3A), 43 proteins associated with COVID-19 hospitalization (Figure 3B), and 63 proteins associated with SARS-COV2 infection (Figure 3C). There are 24 proteins shared by the three traits, including ADGRG2, AMY2B, CCL15, CD109, CD209, CD34, CX3CL1, FGF19, GOLM2, ICAM5, ISLR2, KLK1, LEFTY2, NRCAM, PECAM1, PODXL, PTPRM, REG1A, REG1B, SCG2, SEMA4C, SFTPD, TDGF1, and VAMP5. Among them, 8 proteins increased the risk of severe COVID-19, COVID-19 hospitalization and SARS-COV2 infection, 16 decreased the risk of COVID-19. Supplementary Table 5 showed the function of proteins affecting severe COVID-19, COVID-19 hospitalization and/or SARS-COV2 infection. These proteins had functions involved in inflammation and immunity, such as SFTPD, ICAM-2, ICAM-5, CD209, CD58, CCL15, CCL28, and MNDA; apoptosis, such as FGFR2 and ERBB4; and metabolism such as AMY2A and AMY2B.

In sensitivity analyses, we found that the estimates were generally robust to different MR methods, i.e., weighted median, weighted mode and MR-SPI (Supplementary Tables 6–11). The associations were also consistent when using cis-SNPs as instruments (Supplementary Tables 12–14).

### Proteins causally related to healthspan and lifespan

**Figure 4:**
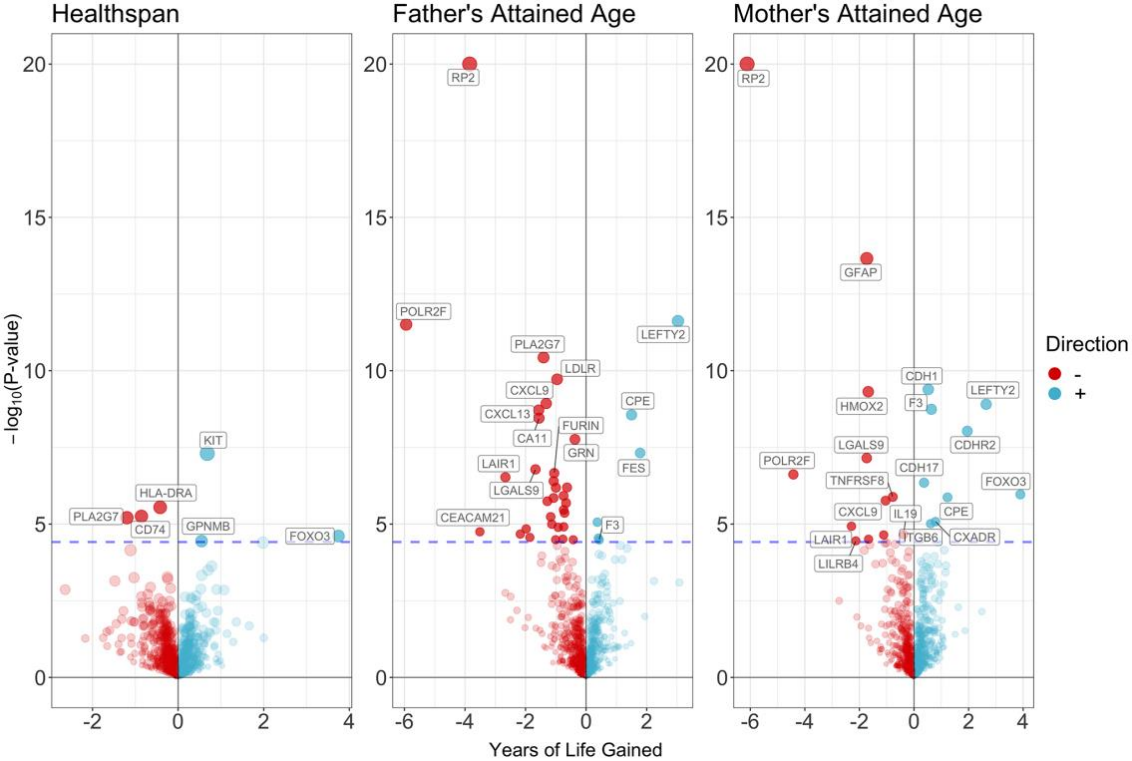
Volcano plot on the associations of proteins with healthspan and lifespan.

**Figure 5:**
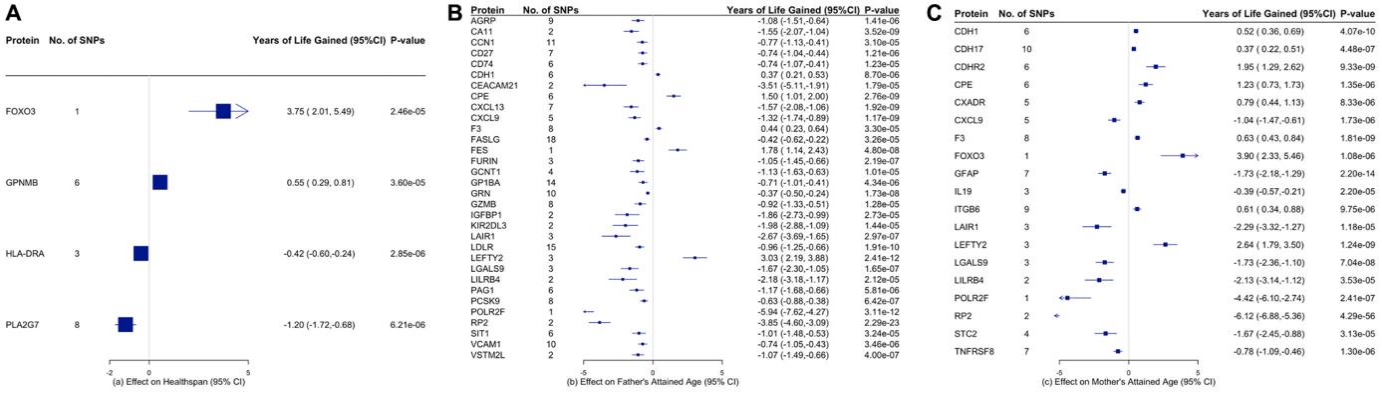
Proteins that are significantly associated with (**A**) healthspan, (**B**) father’s attained age, and (**C**) mother’s attained age. The inverse-variance weighted (IVW) estimates are presented in years of life gained with the corresponding 95% confidence intervals.

Figure 4 and Supplementary Tables 15–17 showed the associations of all proteins with healthspan and lifespan (mother’s and father’s attained age). Figure 5 showed the selected proteins associated with healthspan (Figure 5A), father’s attained age (Figure 5B) and mother’s attained age (Figure 5C). We identified 4 proteins related to healthspan (Figure 5A), 32 proteins related to father’s attained age (Figure 5B), 19 proteins related to mother’s attained age (Figure 5C). Supplementary Table 18 showed the function of proteins affecting healthspan and/or lifespan. The proteins are also involved in inflammation and immunity, such as CXCL9, HLA-DRA, LILRB4, IL19 and TNFRSF8, apoptosis, such as FOXO3, and involved in metabolism, such as PCSK9 and LDLR. Ten proteins play a role in both maternal and paternal ageing, including CDH1, CPE, CXCL9, F3, LAIR1, LEFTY2, LGALS9, LILRB4, POLR2F and RP2. The analysis was robust to weighted median, weighted mode and MR-SPI (Supplementary Tables 19–24). The associations were also consistent when using cis-SNPs as instruments (Supplementary Tables 25–27). Heterogeneity test suggested large heterogeneity for some protein-outcome associations (Supplementary Tables 28–33), such as AMY2B and severe COVID-19, in which case estimates from more robust methods are more valid.

### Proteins related to both COVID-19 and ageing

According to Figures 3 and 5, we found two proteins shared between severe COVID-19 and healthspan/lifespan, including CXADR, and LEFTY2. Two proteins shared between COVID-19 hospitalization and healthspan/lifespan, including CXADR and LEFTY2. Three proteins were shared between SARS-COV2 infection and healthspan/lifespan, including CDH17, CXADR and LEFTY2.

### Drug repurposing opportunity

Table 1 shows the proteins targeted by existing drugs. In this study, we found three proteins that affect the risk of COVID-19 in MR analysis have already been targeted by drugs currently used for the treatment of epilepsy, glaucoma, rheumatoid arthritis and neoplasm. Seven proteins that affect healthspan or lifespan have been targeted by drugs currently used for the treatment of cardiovascular disease, atherosclerosis, and neoplasm. This suggests that these existing drugs have the repurposing potential for lowering the risk of COVID-19 or improving healthspan/lifespan.

**Table 1:** Proteins targeted by existing drugs with potential for drug repurposing.

**Protein**	**Findings in this MR**	**Drug(s) targeting this protein**	**Disease of treatment**	**Action type**
CA4	Increases the risk of COVID-19 hospitalization and SARS-COV2 infection	METHAZOLAMIDE	Open-angle glaucoma	Inhibitor
		ACETAZOLAMIDE	Epilepsy	Inhibitor
		TOPIRAMATE	Migraine disorder	Inhibitor
		SULTHIAME	Epilepsy	Inhibitor
		DICHLORPHENAMIDE	Glaucoma	Inhibitor
FGFR2	Increases the risk of SARS-COV2 infection	NINTEDANIB	Pulmonary fibrosis; neoplasm	Inhibitor
		REGORAFENIB	Metastatic colorectal cancer	Inhibitor
		ERDAFITINIB	Neoplasm	Inhibitor
TDGF1	Lowers the risk of three COVID-19 related traits	BIIB-015	Neoplasm	Binding agent
CD74	Lowers father’s lifespan	MILATUZUMAB	Multiple myeloma; chronic lymphocytic leukemia	Antagonist
GP1BA	Lowers father’s lifespan	ANFIBATIDE	Myocardial infarction	Antagonist
GPNMB	Increases healthspan	GLEMBATUMUMAB VEDOTIN; GLEMBATUMUMAB	Breast cancer; melanoma	Binding agent
KIR2DL3	Lowers father’s lifespan	IPH-2101; LIRILUMAB	Multiple myeloma	Inhibitor
PCSK9	Lowers father’s lifespan	PCSK9 inhibitors, such as EVOLOCUMAB and INCLISIRAN	Cardiovascular disease	Inhibitor
PLA2G7	Lowers healthspan	DARAPLADIB	Atherosclerosis	Inhibitor
		RILAPLADIB	Atherosclerosis; Alzheimer’s disease	Inhibitor
TNFRSF8	Lowers mother’s lifespan	BRENTUXIMAB VEDOTIN	Lymphoma	Inhibitor

## DISCUSSION

Using MR to minimize confounding bias, we not only confirmed some proteins reported in previous studies, such as SFTPD lowering the risk of severe COVID-19 [8], but also identified several novel proteins which are associated with the risk of COVID-19, with functions involved in inflammation and immunity, apoptosis and metabolism. We also identified novel proteins related to healthspan and lifespan, and some of them are also involved in inflammation and immunity, such as CXCL9, HLA-DRA, LILRB4, IL19 and TNFRSF8, in apoptosis, such as FOXO3, and involved in metabolism, such as PCSK9 and LDLR. This finding implies that drugs targeting on these proteins may be used for disease prevention and treatment. For example, PCSK9 inhibitors, which have been used to treat hyperlipidemia, increase years of life. The identification of these proteins deepened the understanding of molecular mechanisms and provided new targets, with relevance to new drug development. Notably, we found 3 proteins affecting COVID-19 and 7 proteins affecting healthspan or lifespan are targeted by existing drugs, suggesting a great potential of drug repurposing. In the following discussion, we discussed in detail these proteins involved in these functions, as well as proteins targeted by existing drugs.

### Proteins involved in inflammation and immune function

In the analysis of COVID-19, our findings are consistent with a previous MR study showing SFTPD is related to lower severe COVID-19 [8]. The protein is part of the innate immune response, the protein and the gene encoding this protein protects the lung against inhaled microorganisms and chemicals [19]. SFTPD also interacts with COVID-19 spike proteins [20]. Partly consistent with a previous MR study showing sICAM-2 lowers the risk of severe COVID-19 [21] and in line with another MR study showing ICAM5 lowers the risk of severe COVID-19 [22], we found ICAM-2 and ICAM-5 lower the risk of COVID-19 hospitalization. ICAM-2 and ICAM-5 both belong to the Ig-like cell adhesion molecule family which may play a role in lymphocyte recirculation by blocking LFA-1-dependent cell adhesion. They mediate adhesive interactions important for antigen-specific immune response, NK-cell mediated clearance, lymphocyte recirculation, and other cellular interactions important for immune response and surveillance. The gene, *ICAM5,* is also related to severe COVID-19 [23].

Moreover, we identified several other inflammation-related proteins that are linked to COVID-19, such as CD209, CD58, CCL15, CCL28, and MNDA. CD209 is a pathogen-recognition receptor expressed on the surface of immature dendritic cells (DCs) and involved in initiation of primary immune response. *In vitro* experiment shows that CD209 also serves as alternative receptors for SARS-CoV-2 in disease-relevant cell types, including the vascular system [24]. This is in line with our findings that CD209 increased the risk of severe COVID-19, COVID-19 hospitalization and SARS-COV2 infection. CD58 is involved in activation of NK and T cells, a reduction in the expression of *CD58* results in reduced activation of NK and cytotoxic T cells and may play a role in COVID-19 [25]. This may explain why we found CD58 lowers the risk of severe COVID-19 in our MR study. CCL15, a chemokine involved in leukocyte trafficking, was identified as predictor for severe COVID-19 [26]. CCL28 displays strong homing capabilities for B and T cells and orchestrates the trafficking and functioning of lymphocytes [27]. CCL28 may be used as an indicator for mucosal immune responses in people with SARS-COV2 infection [28]. In our study, we found CCL28 lowers the risk of SARS-COV2 infection. MNDA plays a role in the granulocyte/monocyte cell-specific response to interferon. It is required for INFα production from human blood cells in response to viruses [29], and may contribute to the immune response to SARS-CoV-2 [30].

Meanwhile, in the analysis for healthspan and lifespan, we also found several proteins which play a role in inflammation and immune function and have not been reported in previous MR studies, including CXCL9, HLA-DRA, LILRB4, IL19 and TNFRSF8. CXCL9 was involved in T cell trafficking. In animal experiments, it increased with aging, which can be prevented by calorie restriction, an established approach of increasing longevity [31]. Consistently, we found CXCL9 was related to shorter years of life. HLA-DRA was expressed on the surface of various antigen presenting cells such as B lymphocytes, dendritic cells, and monocytes/macrophages, and plays a central role in the immune system. The expression of HLA-DRA was higher in older people [32]. LILRB4 plays an important role in adaptive immunity, and increases with age in animal experiments [33]. In our study, we further suggested that increased HLA-DRA and LILRB4 lower healthspan and lifespan, respectively. TNFRSF8 may play a role in the regulation of cellular growth, transformation of activated lymphoblasts. Its regulating gene, *TNFRSF8*, has also been shown to relate to ageing [34]. IL19 promotes the production of IL6, which increased with age and its *in vitro* synthesis is prevented by diet restriction [35]. In our study, for the first time, we clearly showed that TNFRSF8 and IL19 possibly lowered lifespan.

### Proteins involved in apoptosis

Apoptosis is an important process in aging. Consistent with previous studies [36], we found FOXO3 affects healthspan and maternal lifespan. The *FOXO3* gene functions as a trigger for apoptosis through expression of genes necessary for cell death. Notably, in the proteins related to COVID-19, we also found several proteins involved in apoptosis, such as FGFR2 and ERBB4. FGFR2 promotes gastric cancer progression by inhibiting the expression of Thrombospondin4 via PI3K-Akt-Mtor pathway [37]. Drug targeting FGFR2 has been hypothesized to be used as treatments for COVID-19 [38] but this has not been tested in trials. ERBB4 is a tyrosine-protein kinase that plays an essential role as cell surface receptor for neuregulins and EGF family members and regulates cell proliferation, differentiation, migration and apoptosis. ERBB4 was downregulated after the coronavirus infection, and was upregulated by using the Wortmannin, which may inhibit the pathological cycle and development of SARS-CoV-2 in the human hosts [39].

Notably, we also found LEFTY2 contributes to both COVID-19 and lifespan. LEFTY2 lowers the risk of all COVID-19 phenotypes and increases paternal and maternal lifespan. *LEFTY2* encodes a secreted ligand of the transforming growth factor-beta (TGF-beta) superfamily of proteins, which acts as a crucial regulator of cell growth, proliferation, differentiation and apoptosis [40]. More studies are needed to clarify the pathways underlying the effects of LEFTY2, to provide insights for clinical practice and healthcare.

### Proteins involved in metabolism

Metabolic syndrome is a known risk factor for COVID-19 and leads to multiple chronic diseases and mortality. Interestingly, we identified several proteins regulating metabolism. For example, we found AMY2B lowers the risk of severe COVID-19 and AMY2A lowers the risk of hospitalization. AMY2A and AMY2B both play a role in carbohydrate metabolism; the former (AMY2A) was FDA approved drug target. Evidently, the levels of AMY2A and AMY2B were relatively higher in survivors of COVID-19 compared to those who died of COVID-19 [41].

Among the proteins for aging, we found PLA2G7, PCSK9 and LDLR, which are all related to lipid metabolism, lower healthspan and/or lifespan. PLA2G7 has been associated with atherosclerosis, diabetes, and cardiovascular disease, and considered as a potential target that is a nexus between the immune, metabolic, and cardiovascular pathways of aging [42, 43]. PCSK9 and LDLR play an important role in lipid metabolism, and lead to higher risk of IHD, the leading cause of mortality. PCSK9 inhibitors, the drugs targeting PCSK9, are getting arising attention. In this novel study, we showed that PCSK9 inhibitors not only lower the cardiovascular risk, but also should be considered as a treatment for aging.

### Proteins related to hormone regulation

Hormones have been thought to play a vital role in COVID-19 and ageing. In our study, we also found proteins regulating hormone metabolism affected COVID-19 and healthspan/lifespan. For example, NELL2, which was involved in the regulation of hypothalamic GNRH secretion and the control of puberty, lowers the risk of COVID-19 hospitalization and SARS-COV2 infection. In line with our findings, *NELL2* is downregulated in patients with severe and mild COVID-19 in comparison to controls [44]. In proteins related to lifespan, we found IGFBP-1 lowers paternal lifespan. IGFBP-1 binds with IGF-1, which increased the risk of prostate cancer [45] and cardiometabolic diseases [46]. We also found AgRP lowers paternal lifespan. The main action of AgRP involves its antagonistic binding to melanocortin receptors 3 and 4, which are normally targeted by alpha Melanocyte Stimulating Hormone [47]. AgRP-deficiency was thought to lead to increased lifespan [48]. In our study, we confirmed its role in lifespan.

### Drug repurposing opportunity

Importantly, we found that several proteins are targeted by existing drugs, which provided novel insights into drug repurposing. For example, we found that PLA2G7 which lowers healthspan is targeted by PLA2G7 inhibitors which are currently used in the treatment for atherosclerosis and Alzheimer disease. This suggests that PLA2G7 inhibitors may be repurposed to increase healthspan. Similarly, we suggested several drugs, such as FGFR2 inhibitors which have been used for the treatment of neoplasm can be considered to lower the risk of COVID-19. These novel findings are worthwhile to be tested in future randomized controlled trials.

### Strengths and limitations

Using MR enables us to minimize unmeasured confounding bias in cohort studies and case-control studies. In this study, we used by far the largest available GWAS of proteomics, COVID-19, lifespan and healthspan, which improves the power to identify proteins causally related to these outcomes. Despite, we also acknowledge a few limitations. First, MR is based on three core assumptions, i.e., the relevance, independence, and exclusion-restriction assumption [49]. To satisfy these assumptions, we used genetic variants strongly associated with these proteins, with Bonferroni-corrected significance. We also used multiple sensitivity analysis methods robust to pleiotropy and checked the directions of associations from different genetic instruments (trans plus cis SNPs versus cis SNPs). In the situation where heterogeneity test suggested large heterogeneity, estimates from methods robust to pleiotropy are more valid than IVW. Considering that the measurements of proteins at baseline were conducted before the occurrence of the outcomes, reverse causality is not a main concern, so we did not perform bi-directional MR analysis. Second, MR studies of COVID-19 might be subject to survivor bias (selection bias), i.e., people might have died of COVID-19 or other diseases before recruitment. So, the causal effects might be underestimated. Third, population stratification might affect MR estimates. However, the GWAS data used in this study were derived from people largely of European ancestry. Meanwhile, as the study was based on people of European ancestry, the findings may not be generalizable to other ancestries. Fourth, the samples for proteins and outcomes both included UK Biobank. The partly overlapping in samples may bias two-sample MR estimates [2], but a simulation study shows two-sample MR can be safely conducted in a single large dataset [3], such as UK Biobank. So, the sample overlapping may not be a main concern, but the estimates need to be interpreted more cautiously. Finally, as we used summary statistics, we cannot assess the potential nonlinear association of these proteins with COVID-19, healthspan and lifespan.

## CONCLUSIONS

In this study, using genetic instruments from by far the largest GWAS of proteomics, as well as by far the largest GWAS of COVID-19, healthspan and lifespan, we identified multiple proteins affecting COVID-19 and aging. We not only confirmed proteins identified in previous studies, such as SFTPD which affects COVID-19 and FOXO3 which affects healthspan, notably we identified several proteins with functions involved in inflammation and immune function, apoptosis, metabolism and hormone regulation. These findings provide novel insights into disease pathogenesis and pinpoint targets for therapeutic development or drug repurposing. For example, our findings of PCSK9 shortening lifespan and PLA2G7 shortening healthspan suggested that PCSK9 inhibitors, such as Evolocumab, and drugs inhibiting PLA2G7, such as Darapladib, may be beneficial for increasing lifespan and healthspan.

## Supplementary Materials

Supplementary Methods

Supplementary Tables 1-5 and 15-18

Supplementary Tables 6-14 and 19-33

## References

[1] Msemburi W, Karlinsky A, Knutson V, Aleshin-Guendel S, Chatterji S, Wakefield J. The WHO estimates of excess mortality associated with the COVID-19 pandemic. Nature. 2023; 613:130–7. 10.1038/s41586-022-05522-2 36517599 PMC9812776

[2] Aburto JM, Kashyap R, Schöley J, Angus C, Ermisch J, Mills MC, Dowd JB. Estimating the burden of the COVID-19 pandemic on mortality, life expectancy and lifespan inequality in England and Wales: a population-level analysis. J Epidemiol Community Health. 2021; 75:735–40. 10.1136/jech-2020-215505 33468602 PMC7818788

[3] Furman D, Campisi J, Verdin E, Carrera-Bastos P, Targ S, Franceschi C, Ferrucci L, Gilroy DW, Fasano A, Miller GW, Miller AH, Mantovani A, Weyand CM, et al. Chronic inflammation in the etiology of disease across the life span. Nat Med. 2019; 25:1822–32. 10.1038/s41591-019-0675-0 31806905 PMC7147972

[4] Blanco-Melo D, Nilsson-Payant BE, Liu WC, Uhl S, Hoagland D, Møller R, Jordan TX, Oishi K, Panis M, Sachs D, Wang TT, Schwartz RE, Lim JK, et al. Imbalanced Host Response to SARS-CoV-2 Drives Development of COVID-19. Cell. 2020; 181:1036–45.e9. 10.1016/j.cell.2020.04.026 32416070 PMC7227586

[5] Bi X, Liu W, Ding X, Liang S, Zheng Y, Zhu X, Quan S, Yi X, Xiang N, Du J, Lyu H, Yu D, Zhang C, et al. Proteomic and metabolomic profiling of urine uncovers immune responses in patients with COVID-19. Cell Rep. 2022; 38:110271. 10.1016/j.celrep.2021.110271 35026155 PMC8712267

[6] Al-Bayatee NT, Ad'hiah AH. Soluble HLA-G is upregulated in serum of patients with severe COVID-19. Hum Immunol. 2021; 82:726–32. 10.1016/j.humimm.2021.07.007 34304938 PMC8282477

[7] Lawlor DA, Harbord RM, Sterne JA, Timpson N, Davey Smith G. Mendelian randomization: using genes as instruments for making causal inferences in epidemiology. Stat Med. 2008; 27:1133–63. 10.1002/sim.3034 17886233

[8] Palmos AB, Millischer V, Menon DK, Nicholson TR, Taams LS, Michael B, Sunderland G, Griffiths MJ, Hübel C, Breen G, and COVID Clinical Neuroscience Study Consortium. Proteome-wide Mendelian randomization identifies causal links between blood proteins and severe COVID-19. PLoS Genet. 2022; 18:e1010042. 10.1371/journal.pgen.1010042 35239653 PMC8893330

[9] Gaziano L, Giambartolomei C, Pereira AC, Gaulton A, Posner DC, Swanson SA, Ho YL, Iyengar SK, Kosik NM, Vujkovic M, Gagnon DR, Bento AP, Barrio-Hernandez I, et al, and VA Million Veteran Program COVID-19 Science Initiative. Actionable druggable genome-wide Mendelian randomization identifies repurposing opportunities for COVID-19. Nat Med. 2021; 27:668–76. 10.1038/s41591-021-01310-z 33837377 PMC7612986

[10] Perrot N, Pelletier W, Bourgault J, Couture C, Li Z, Mitchell PL, Ghodsian N, Bossé Y, Thériault S, Mathieu P, Arsenault BJ. A trans-omic Mendelian randomization study of parental lifespan uncovers novel aging biology and therapeutic candidates for chronic diseases. Aging Cell. 2021; 20:e13497. 10.1111/acel.13497 34704651 PMC8590095

[11] Sun BB, Chiou J, Traylor M, Benner C, Hsu YH, Richardson TG, Surendran P, Mahajan A, Robins C, Vasquez-Grinnell SG, Hou L, Kvikstad EM, Burren OS, et al. Genetic regulation of the human plasma proteome in 54,306 UK Biobank participants. bioRxiv. 2022. 10.1101/2022.06.17.496443

[12] The COVID-19 Host Genetics Initiative. https://www.covid19hg.org/.

[13] Zenin A, Tsepilov Y, Sharapov S, Getmantsev E, Menshikov LI, Fedichev PO, Aulchenko Y. Identification of 12 genetic loci associated with human healthspan. Commun Biol. 2019; 2:41. 10.1038/s42003-019-0290-0 30729179 PMC6353874

[14] Pilling LC, Kuo CL, Sicinski K, Tamosauskaite J, Kuchel GA, Harries LW, Herd P, Wallace R, Ferrucci L, Melzer D. Human longevity: 25 genetic loci associated in 389,166 UK biobank participants. Aging (Albany NY). 2017; 9:2504–20. 10.18632/aging.101334 29227965 PMC5764389

[15] Bowden J, Davey Smith G, Haycock PC, Burgess S. Consistent Estimation in Mendelian Randomization with Some Invalid Instruments Using a Weighted Median Estimator. Genet Epidemiol. 2016; 40:304–14. 10.1002/gepi.21965 27061298 PMC4849733

[16] Hartwig FP, Davey Smith G, Bowden J. Robust inference in summary data Mendelian randomization via the zero modal pleiotropy assumption. Int J Epidemiol. 2017; 46:1985–98. 10.1093/ije/dyx102 29040600 PMC5837715

[17] Yao M, Guo Z, Liu Z. Robust Mendelian Randomization Analysis by Automatically Selecting Valid Genetic Instruments for Inferring Causal Relationships between Complex Traits and Diseases. medRxiv. 2023. 10.1101/2023.02.20.23286200

[18] Szklarczyk D, Gable AL, Lyon D, Junge A, Wyder S, Huerta-Cepas J, Simonovic M, Doncheva NT, Morris JH, Bork P, Jensen LJ, Mering CV. STRING v11: protein-protein association networks with increased coverage, supporting functional discovery in genome-wide experimental datasets. Nucleic Acids Res. 2019; 47:D607–13. 10.1093/nar/gky1131 30476243 PMC6323986

[19] Ortega FJ, Pueyo N, Moreno-Navarrete JM, Sabater M, Rodriguez-Hermosa JI, Ricart W, Tinahones FJ, Fernández-Real JM. The lung innate immune gene surfactant protein-D is expressed in adipose tissue and linked to obesity status. Int J Obes (Lond). 2013; 37:1532–8. 10.1038/ijo.2013.23 23478426

[20] Chen L, Zheng S. Understand variability of COVID-19 through population and tissue variations in expression of SARS-CoV-2 host genes. Inform Med Unlocked. 2020; 21:100443. 10.1016/j.imu.2020.100443 33072849 PMC7550072

[21] Zhu J, Wu C, Wu L. Associations Between Genetically Predicted Protein Levels and COVID-19 Severity. J Infect Dis. 2021; 223:19–22. 10.1093/infdis/jiaa660 33083826 PMC7797748

[22] Zheng J, Zhang Y, Zhao H, Liu Y, Baird D, Karim MA, Ghoussaini M, Schwartzentruber J, Dunham I, Elsworth B, Roberts K, Compton H, Miller-Molloy F, et al. Multi-ancestry Mendelian randomization of omics traits revealing drug targets of COVID-19 severity. EBioMedicine. 2022; 81:104112. 10.1016/j.ebiom.2022.104112 35772218 PMC9235320

[23] Velavan TP, Pallerla SR, Rüter J, Augustin Y, Kremsner PG, Krishna S, Meyer CG. Host genetic factors determining COVID-19 susceptibility and severity. EBioMedicine. 2021; 72:103629. 10.1016/j.ebiom.2021.103629 34655949 PMC8512556

[24] Amraei R, Yin W, Napoleon MA, Suder EL, Berrigan J, Zhao Q, Olejnik J, Chandler KB, Xia C, Feldman J, Hauser BM, Caradonna TM, Schmidt AG, et al. CD209L/L-SIGN and CD209/DC-SIGN Act as Receptors for SARS-CoV-2. ACS Cent Sci. 2021; 7:1156–65. 10.1021/acscentsci.0c01537 34341769 PMC8265543

[25] van Eeden C, Khan L, Osman MS, Cohen Tervaert JW. Natural Killer Cell Dysfunction and Its Role in COVID-19. Int J Mol Sci. 2020; 21:6351. 10.3390/ijms21176351 32883007 PMC7503862

[26] Lucas C, Wong P, Klein J, Castro TBR, Silva J, Sundaram M, Ellingson MK, Mao T, Oh JE, Israelow B, Takahashi T, Tokuyama M, Lu P, et al, and Yale IMPACT Team. Longitudinal analyses reveal immunological misfiring in severe COVID-19. Nature. 2020; 584:463–9. 10.1038/s41586-020-2588-y 32717743 PMC7477538

[27] Mohan T, Deng L, Wang BZ. CCL28 chemokine: An anchoring point bridging innate and adaptive immunity. Int Immunopharmacol. 2017; 51:165–70. 10.1016/j.intimp.2017.08.012 28843907 PMC5755716

[28] Yan Y, Jiang X, Wang X, Liu B, Ding H, Jiang M, Yang Z, Dai Y, Ding D, Yu H, Zhang S, Liu J, Sha M, et al. CCL28 mucosal expression in SARS-CoV-2-infected patients with diarrhea in relation to disease severity. J Infect. 2021; 82:e19–21. 10.1016/j.jinf.2020.08.042 32871180 PMC7833095

[29] Gu L, Casserly D, Brady G, Carpenter S, Bracken AP, Fitzgerald KA, Unterholzner L, Bowie AG. Myeloid cell nuclear differentiation antigen controls the pathogen-stimulated type I interferon cascade in human monocytes by transcriptional regulation of IRF7. Nat Commun. 2022; 13:14. 10.1038/s41467-021-27701-x 35013241 PMC8748983

[30] Beltrán-Camacho L, Eslava-Alcón S, Rojas-Torres M, Sánchez-Morillo D, Martinez-Nicolás MP, Martín-Bermejo V, de la Torre IG, Berrocoso E, Moreno JA, Moreno-Luna R, Durán-Ruiz MC. The serum of COVID-19 asymptomatic patients up-regulates proteins related to endothelial dysfunction and viral response in circulating angiogenic cells ex-vivo. Mol Med. 2022; 28:40. 10.1186/s10020-022-00465-w 35397534 PMC8994070

[31] Trott DW, Lesniewski LA, Donato AJ. Selected life-extending interventions reduce arterial CXCL10 and macrophage colony-stimulating factor in aged mouse arteries. Cytokine. 2017; 96:102–6. 10.1016/j.cyto.2017.03.008 28390264 PMC5544385

[32] de Almeida Chuffa LG, Freire PP, Dos Santos Souza J, de Mello MC, de Oliveira Neto M, Carvalho RF. Aging whole blood transcriptome reveals candidate genes for SARS-CoV-2-related vascular and immune alterations. J Mol Med (Berl). 2022; 100:285–301. 10.1007/s00109-021-02161-4 34741638 PMC8571664

[33] Jonker MJ, Melis JP, Kuiper RV, van der Hoeven TV, Wackers PFK, Robinson J, van der Horst GT, Dollé ME, Vijg J, Breit TM, Hoeijmakers JH, van Steeg H. Life spanning murine gene expression profiles in relation to chronological and pathological aging in multiple organs. Aging Cell. 2013; 12:901–9. 10.1111/acel.12118 23795901 PMC3772962

[34] Tanaka T, Biancotto A, Moaddel R, Moore AZ, Gonzalez-Freire M, Aon MA, Candia J, Zhang P, Cheung F, Fantoni G, Semba RD, Ferrucci L, and CHI consortium. Plasma proteomic signature of age in healthy humans. Aging Cell. 2018; 17:e12799. 10.1111/acel.12799 29992704 PMC6156492

[35] Ershler WB, Sun WH, Binkley N, Gravenstein S, Volk MJ, Kamoske G, Klopp RG, Roecker EB, Daynes RA, Weindruch R. Interleukin-6 and aging: blood levels and mononuclear cell production increase with advancing age and in vitro production is modifiable by dietary restriction. Lymphokine Cytokine Res. 1993; 12:225–30. 8218595

[36] Willcox BJ, Tranah GJ, Chen R, Morris BJ, Masaki KH, He Q, Willcox DC, Allsopp RC, Moisyadi S, Poon LW, Rodriguez B, Newman AB, Harris TB, et al. The FoxO3 gene and cause-specific mortality. Aging Cell. 2016; 15:617–24. 10.1111/acel.12452 27071935 PMC4933667

[37] Huang T, Liu D, Wang Y, Li P, Sun L, Xiong H, Dai Y, Zou M, Yuan X, Qiu H. FGFR2 Promotes Gastric Cancer Progression by Inhibiting the Expression of Thrombospondin4 via PI3K-Akt-Mtor Pathway. Cell Physiol Biochem. 2018; 50:1332–45. 10.1159/000494590 30355943

[38] Sonkar C, Doharey PK, Rathore AS, Singh V, Kashyap D, Sahoo AK, Mittal N, Sharma B, Jha HC. Repurposing of gastric cancer drugs against COVID-19. Comput Biol Med. 2021; 137:104826. 10.1016/j.compbiomed.2021.104826 34537409 PMC8420180

[39] Selvaraj G, Kaliamurthi S, Peslherbe GH, Wei DQ. Identifying potential drug targets and candidate drugs for COVID-19: biological networks and structural modeling approaches. F1000Res. 2021; 10:127. 10.12688/f1000research.50850.3 33968364 PMC8080978

[40] Lu WQ, Qiu JL, Huang ZL, Liu HY. Enhanced circulating transforming growth factor beta 1 is causally associated with an increased risk of hepatocellular carcinoma: a mendelian randomization meta-analysis. Oncotarget. 2016; 7:84695–704. 10.18632/oncotarget.13218 27835897 PMC5356692

[41] Filbin MR, Mehta A, Schneider AM, Kays KR, Guess JR, Gentili M, Fenyves BG, Charland NC, Gonye ALK, Gushterova I, Khanna HK, LaSalle TJ, Lavin-Parsons KM, et al. Longitudinal proteomic analysis of severe COVID-19 reveals survival-associated signatures, tissue-specific cell death, and cell-cell interactions. Cell Rep Med. 2021; 2:100287. 10.1016/j.xcrm.2021.100287 33969320 PMC8091031

[42] Cao F, Lee RT. PLA2G7, caloric restriction and cardiovascular aging. J Cardiovasc Aging. 2022; 2:19. 10.20517/jca.2022.08 35497092 PMC9053108

[43] Spadaro O, Youm Y, Shchukina I, Ryu S, Sidorov S, Ravussin A, Nguyen K, Aladyeva E, Predeus AN, Smith SR, Ravussin E, Galban C, Artyomov MN, Dixit VD. Caloric restriction in humans reveals immunometabolic regulators of health span. Science. 2022; 375:671–7. 10.1126/science.abg7292 35143297 PMC10061495

[44] Aschenbrenner AC, Mouktaroudi M, Krämer B, Oestreich M, Antonakos N, Nuesch-Germano M, Gkizeli K, Bonaguro L, Reusch N, Baßler K, Saridaki M, Knoll R, Pecht T, et al, and German COVID-19 Omics Initiative (DeCOI). Disease severity-specific neutrophil signatures in blood transcriptomes stratify COVID-19 patients. Genome Med. 2021; 13:7. 10.1186/s13073-020-00823-5 33441124 PMC7805430

[45] Bonilla C, Lewis SJ, Rowlands MA, Gaunt TR, Davey Smith G, Gunnell D, Palmer T, Donovan JL, Hamdy FC, Neal DE, Eeles R, Easton D, Kote-Jarai Z, et al, and PRACTICAL consortium. Assessing the role of insulin-like growth factors and binding proteins in prostate cancer using Mendelian randomization: Genetic variants as instruments for circulating levels. Int J Cancer. 2016; 139:1520–33. 10.1002/ijc.30206 27225428 PMC4957617

[46] Larsson SC, Michaëlsson K, Burgess S. IGF-1 and cardiometabolic diseases: a Mendelian randomisation study. Diabetologia. 2020; 63:1775–82. 10.1007/s00125-020-05190-9 32548700 PMC7406523

[47] Ilnytska O, Argyropoulos G. The role of the Agouti-Related Protein in energy balance regulation. Cell Mol Life Sci. 2008; 65:2721–31. 10.1007/s00018-008-8104-4 18470724 PMC2748318

[48] Redmann SM Jr, Argyropoulos G. AgRP-deficiency could lead to increased lifespan. Biochem Biophys Res Commun. 2006; 351:860–4. 10.1016/j.bbrc.2006.10.129 17097059 PMC1771482

[49] Davies NM, Holmes MV, Davey Smith G. Reading Mendelian randomisation studies: a guide, glossary, and checklist for clinicians. BMJ. 2018; 362:k601. 10.1136/bmj.k601 30002074 PMC6041728

