# Supplementary material for: Using genetics and proteomics data to identify proteins causally related to COVID-19, healthspan and lifespan: a Mendelian randomization study
Source: Aging (Albany NY). 2024 Apr 3;16(7):6384–416. doi: 10.18632/aging.205711 (PMC11042960; doi:10.18632/aging.205711)
Supplement: Supplementary Methods [file aging-16-205711-s001.pdf]

Supplementary Methods

The proteomics data come from the UK Biobank Pharma Proteomics Project (UKB-PPP), compromising 54,306 samples (46,673 UKB participants at baseline visit, 6,385 samples selected by the UKB-PPP consortium, and 1,268 samples who participated in the COVID-19 repeat imaging study). The selection of UKB-PPP samples was based on two temporally and algorithmically separated picking processes. The first process involved pre-selecting 5,500 samples from participants' baseline recruitment visits, supplemented by additional 44,502 samples selected from the UK Biobank cohort with stratification against age, sex, and recruitment centre. The second process included a total of 7,000 samples, with 1,020 pre-selected samples, 3,637 samples from participants in the COVID-19 case-control imaging study, and 2,343 baseline samples selected randomly as described for the first sample selection. The full description of the selection criteria can be found in the Supplementary Material in Sun, et al. [1]. Generally, the 46,673 UKB participants were highly representative of the overall UKB population across various demographic characteristics except for deprivation index, distribution of recruitment centres, and time since recruitment. The 6,385 UKB-PPP consortium-selected participants were on average 2.5 years older than the overall UKB participants, and there were also differences in sex, ethnic background, body mass index, and smoking prevalence. The 1,268 COVID-19 imaging participants were on average 6.3 years younger than the overall UKB participants, while the other demographic characteristics were comparable to the UKB population.

Supplementary References

1. Sun BB, Chiou J, Traylor M, Benner C, Hsu YH, Richardson TG, Surendran P, Mahajan A, Robins C, Vasquez-Grinnell SG, Hou L, Kvikstad EM, Burren OS, et al. Genetic regulation of the human plasma proteome in 54,306 UK Biobank participants. bioRxiv. 2022. https://doi.org/10.1101/2022.06.17.496443
