# Supplementary material for: Using genetics and proteomics data to identify proteins causally related to COVID-19, healthspan and lifespan: a Mendelian randomization study
Source: Aging (Albany NY). 2024 Apr 3;16(7):6384–416. doi: 10.18632/aging.205711 (PMC11042960; doi:10.18632/aging.205711)
Supplement: Supplementary Tables 1-5 and 15-18 [file aging-16-205711-s002.pdf]

Supplementary Table 1. Genetic instruments for proteins used in the Mendelian randomization analysis.

Protein	SNP	CHR	POS	A1	A2	Frequency	BETA	SE	Pval
AARSD1	rs1354034	3	56815721	C	T	0.60	0.07	0.01	4.11E-09
AARSD1	rs4759076	12	54336088	C	T	0.46	0.05	0.01	1.20E-05
AARSD1	rs323500	17	42988528	T	C	0.29	0.13	0.01	2.34E-28
ABHD14B	rs373373105	3	51970081	G	C	0.01	-1.44	0.04	1.36E-160
ABHD14B	rs1354034	3	56815721	C	T	0.60	0.06	0.01	1.46E-06
ABHD14B	rs6993770	8	105569300	T	A	0.29	-0.06	0.01	2.11E-06
ABHD14B	rs7080386	10	63288546	A	C	0.41	0.06	0.01	4.18E-07
ABL1	rs1354034	3	56815721	C	T	0.60	0.08	0.01	6.05E-13
ACAA1	rs156265	3	38129319	G	C	0.14	-0.13	0.01	2.44E-14
ACAA1	rs4835265	4	145900258	A	C	0.16	0.09	0.01	3.51E-08
ACAA1	rs9953884	18	58426216	C	A	0.77	-0.07	0.01	1.35E-07
ACAN	rs1337526	1	47499458	G	A	0.80	0.06	0.01	8.91E-06
ACAN	rs828602	3	98754474	A	G	0.41	0.07	0.01	2.69E-11
ACAN	rs11713838	3	119209644	A	C	0.27	-0.05	0.01	2.56E-05
ACAN	rs6461354	7	17874977	T	C	0.39	-0.05	0.01	1.91E-05
ACAN	rs12684083	9	76533675	A	G	0.24	-0.10	0.01	1.03E-16
ACAN	rs2519093	9	133266456	T	C	0.18	0.09	0.01	2.04E-10
ACAN	rs56278466	10	17833858	G	T	0.66	0.06	0.01	5.54E-08
ACAN	rs11000138	10	72022264	T	A	0.39	-0.19	0.01	4.11E-71
ACAN	rs895734	11	45662704	T	C	0.59	0.09	0.01	1.24E-16
ACAN	rs59379014	11	126358105	T	C	0.07	0.10	0.01	2.55E-06
ACAN	rs200201338	15	80948905	T	A	0.02	0.20	0.02	2.11E-08
ACAN	rs11073804	15	88708781	A	T	0.20	0.13	0.01	1.13E-23
ACAN	rs186021206	17	7166093	A	G	0.01	0.34	0.05	3.99E-06
ACAN	rs55639531	18	394341	G	C	0.29	0.07	0.01	3.50E-10
ACAN	rs17549173	19	35909329	A	G	0.07	0.11	0.01	1.75E-07
ACE2	rs1047891	2	210675783	A	C	0.31	0.05	0.01	1.59E-06
ACE2	rs9258643	6	29854294	G	A	0.16	-0.08	0.01	1.18E-06
ACE2	rs112875651	8	125494452	A	G	0.39	-0.08	0.01	3.80E-13
ACE2	rs11065385	12	120985583	G	A	0.69	0.14	0.01	1.60E-38
ACE2	rs72694393	14	24404987	T	G	0.48	0.06	0.01	7.68E-08
ACE2	rs28929474	14	94378610	T	C	0.02	0.21	0.02	6.48E-09
ACE2	rs550999044	14	103101069	T	A	0.24	0.07	0.01	1.05E-07
ACE2	rs340005	15	60585831	A	G	0.62	0.05	0.01	2.53E-05
ACE2	rs57909886	15	73681321	C	T	0.48	0.04	0.01	2.30E-05
ACE2	rs72787359	16	51397319	C	A	0.07	-0.09	0.01	7.35E-06
ACE2	-	22	17968075	T	A	0.24	-0.05	0.01	2.01E-05
ACOX1	rs10852766	17	75955783	C	T	0.66	0.07	0.01	2.27E-09
ACP5	rs61801010	1	161572445	G	A	0.11	0.11	0.01	4.06E-09
ACP5	rs150184782	8	104965640	A	C	0.18	0.08	0.01	1.61E-08
ACP5	rs10813949	9	33123466	A	G	0.31	-0.06	0.01	1.53E-06
ACP5	rs2229498	10	69097096	A	G	0.83	-0.06	0.01	1.70E-05
ACP5	rs35382467	11	128329388	A	G	0.10	-0.10	0.01	2.64E-08
ACP5	rs117566084	12	101796743	T	C	0.01	0.36	0.03	9.06E-12
ACP5	rs10138671	14	65393374	G	A	0.61	0.06	0.01	3.60E-07
ACP5	rs2073333	14	94378225	T	C	0.25	0.11	0.01	2.26E-19
ACP5	rs78755089	16	79542724	T	G	0.08	0.11	0.01	1.79E-08
ACP5	rs186021206	17	7166093	A	G	0.01	0.34	0.05	4.44E-06
ACP5	rs2305799	19	11576536	T	C	0.12	-0.50	0.01	2.35E-202
ACP6	rs2999719	1	38661844	C	T	0.93	0.25	0.02	6.79E-27
ACP6	rs77542178	1	119972409	C	T	0.02	-0.19	0.03	5.57E-06
ACP6	rs2153463	1	147652507	G	T	0.70	1.01	0.01	0.00E+00
ACTA2	rs5786849	10	88993792	A	T	0.50	-0.06	0.01	7.13E-09
ACVRL1	-	1	150776487	T	A	0.07	-0.09	0.01	8.67E-06

ACVRL1	rs9264623	6	31270173	C	T	0.64	-0.06	0.01	1.97E-07
ACVRL1	rs9411365	9	133242881	G	C	0.07	0.09	0.01	1.27E-05
ACVRL1	rs77709482	12	51914237	C	T	0.08	0.56	0.01	1.25E-176
ACVRL1	rs2837988	21	41247617	A	C	0.36	0.08	0.01	3.75E-13
ACY1	rs1260326	2	27508073	C	T	0.61	-0.07	0.01	4.08E-10
ACY1	rs121912698	3	51988821	T	C	0.00	-1.68	0.05	2.50E-96
ACY1	rs3747207	22	43928975	A	G	0.22	0.08	0.01	1.24E-08
ADA	rs140373387	2	162012424	C	G	0.00	0.43	0.05	1.86E-08
ADA	rs60757417	9	132989049	G	C	0.06	-0.09	0.01	2.45E-05
ADA	rs597808	12	111535554	G	A	0.52	-0.05	0.01	1.34E-06
ADA	rs11555566	20	44626579	C	T	0.06	1.27	0.02	0.00E+00
ADA2	rs72930772	2	202754954	T	C	0.33	0.08	0.01	2.10E-11
ADA2	rs148819155	3	158627192	G	A	0.00	-0.43	0.06	1.65E-05
ADA2	rs71584289	4	105173363	A	C	0.35	0.06	0.01	1.52E-06
ADA2	rs2548999	5	132495366	T	G	0.66	0.05	0.01	1.78E-05
ADA2	rs535777	6	32609856	C	G	0.14	0.15	0.01	5.05E-22
ADA2	rs56278466	10	17833858	G	T	0.66	0.08	0.01	5.42E-12
ADA2	rs2051773	11	17343662	G	A	0.30	0.06	0.01	5.35E-07
ADA2	rs226379	12	9115989	C	T	0.35	-0.05	0.01	1.50E-05
ADA2	rs117566084	12	101796743	T	C	0.01	0.31	0.04	3.44E-08
ADA2	rs3184504	12	111446804	C	T	0.52	-0.13	0.01	3.73E-33
ADA2	rs145078947	14	93186629	T	G	0.00	0.58	0.07	9.72E-08
ADA2	rs186021206	17	7166093	A	G	0.01	0.67	0.05	4.59E-19
ADA2	rs737963	22	17228881	C	T	0.61	0.21	0.01	2.06E-73
ADA2	rs738409	22	43928847	G	C	0.22	0.08	0.01	2.84E-08
ADAM15	rs11589479	1	155060832	A	G	0.16	1.02	0.01	0.00E+00
ADAM15	rs146142570	3	50316856	A	G	0.00	0.53	0.06	1.48E-07
ADAM15	rs77468121	6	31377707	A	G	0.04	0.12	0.01	3.83E-07
ADAM15	rs62571868	9	110363931	T	A	0.23	0.05	0.01	5.39E-06
ADAM15	rs8176759	9	133254260	A	G	0.06	0.51	0.01	2.35E-152
ADAM15	rs56278466	10	17833858	G	T	0.66	0.04	0.01	2.27E-05
ADAM15	rs3184504	12	111446804	C	T	0.52	-0.06	0.01	2.99E-11
ADAM15	rs186021206	17	7166093	A	G	0.01	0.69	0.04	6.29E-28
ADAM22	rs61747728	1	179557079	T	C	0.04	0.17	0.02	7.29E-10
ADAM22	rs75166367	2	162107791	A	G	0.06	-0.11	0.01	6.48E-07
ADAM22	rs2279542	7	87935182	G	C	0.51	-0.56	0.01	0.00E+00
ADAM22	rs4997081	16	20353912	C	G	0.19	-0.07	0.01	2.33E-07
ADAM22	rs186021206	17	7166093	A	G	0.01	0.33	0.05	4.49E-06
ADAM22	rs2659005	17	81244914	T	C	0.44	0.05	0.01	2.97E-06
ADAM23	rs61747728	1	179557079	T	C	0.04	0.12	0.02	9.10E-07
ADAM23	rs72933203	2	206446625	A	T	0.10	0.88	0.01	0.00E+00
ADAM23	rs1294438	6	6751826	T	C	0.36	0.06	0.01	7.76E-08
ADAM23	rs8176741	9	133256074	A	G	0.06	0.22	0.01	8.82E-29
ADAM23	rs777834943	16	20355409	T	C	0.18	-0.05	0.01	2.01E-05
ADAM23	rs186021206	17	7166093	A	G	0.01	0.48	0.04	3.80E-13
ADAM23	-	19	41439217	C	T	0.39	-0.07	0.01	1.35E-12
ADAM8	-	1	25567436	A	T	0.46	0.07	0.01	1.88E-10
ADAM8	rs2596530	6	31419596	A	G	0.49	-0.06	0.01	1.22E-08
ADAM8	-	9	134957079	G	T	0.25	0.06	0.01	2.66E-05
ADAM8	rs2995310	10	133269306	T	C	0.89	-0.70	0.01	0.00E+00
ADAM8	rs4055121	11	126362442	T	C	0.14	-0.09	0.01	8.03E-08
ADAM8	rs7137828	12	111494996	T	C	0.52	-0.11	0.01	1.79E-23
ADAM8	rs76428106	13	28029870	C	T	0.01	0.25	0.03	7.55E-07
ADAM8	rs186021206	17	7166093	A	G	0.01	0.58	0.05	7.92E-15
ADAM8	rs2227319	17	40014592	A	G	0.37	-0.08	0.01	1.09E-11
ADAMTS13	rs11709284	3	52525689	A	G	0.56	0.05	0.01	2.48E-05

ADAMTS13	rs5023357	4	123849638	G	T	0.11	0.10	0.01	4.69E-09
ADAMTS13	-	4	174155491	T	A	0.46	0.05	0.01	1.12E-06
ADAMTS13	rs2910686	5	96916885	C	T	0.45	0.07	0.01	8.82E-12
ADAMTS13	rs9264275	6	31256777	T	C	0.74	0.06	0.01	5.10E-06
ADAMTS13	rs143073120	6	147344800	T	C	0.56	-0.05	0.01	5.19E-06
ADAMTS13	rs10253113	7	130369470	G	A	0.31	0.07	0.01	9.32E-09
ADAMTS13	rs9987289	8	9325848	G	A	0.91	-0.10	0.01	5.17E-07
ADAMTS13	rs74715985	9	133438993	G	A	0.02	-1.44	0.03	1.86E-246
ADAMTS13	rs112814955	12	6116765	A	G	0.07	0.09	0.01	1.57E-05
ADAMTS13	rs1389065	12	91384364	A	G	0.76	-0.06	0.01	2.74E-05
ADAMTS13	rs746775574	12	104577022	T	G	0.09	-0.10	0.01	7.02E-08
ADAMTS13	rs56254331	19	41320115	C	A	0.17	0.07	0.01	6.45E-06
ADAMTS13	rs2236251	20	17616400	T	C	0.76	0.05	0.01	2.42E-05
ADAMTS13	rs738408	22	43928850	T	C	0.22	0.06	0.01	1.37E-06
ADAMTS15	rs5030062	3	186736391	C	A	0.37	-0.06	0.01	9.74E-08
ADAMTS15	rs4861708	4	186236079	A	G	0.51	-0.12	0.01	3.48E-27
ADAMTS15	rs2545801	5	177414338	C	T	0.74	-0.09	0.01	6.22E-13
ADAMTS15	rs73044885	11	130474168	A	G	0.05	0.19	0.02	6.20E-13
ADAMTS15	rs186021206	17	7166093	A	G	0.01	0.53	0.05	4.38E-11
ADAMTS16	rs547625232	1	207337659	A	T	0.45	-0.05	0.01	2.86E-06
ADAMTS16	rs40468	5	4982701	G	A	0.34	-0.12	0.01	9.03E-27
ADAMTS8	rs7529464	1	230277090	C	G	0.25	0.07	0.01	4.14E-08
ADAMTS8	rs13107325	4	102267552	T	C	0.08	-0.14	0.01	6.39E-13
ADAMTS8	rs2131535	11	130411593	A	G	0.73	0.49	0.01	0.00E+00
ADAMTS8	rs7209484	17	48065680	C	T	0.24	0.06	0.01	9.69E-07
ADGRB3	rs1260326	2	27508073	C	T	0.61	0.03	0.01	1.41E-05
ADGRB3	rs3798969	6	68961693	G	A	0.62	-0.94	0.01	0.00E+00
ADGRB3	rs7952602	11	126363774	C	G	0.13	-0.05	0.01	2.17E-05
ADGRB3	rs200489612	17	7203059	A	G	0.01	0.32	0.04	2.76E-08
ADGRB3	rs573431210	17	69280242	G	T	0.03	0.12	0.02	1.04E-06
ADGRE2	rs61801010	1	161572445	G	A	0.11	0.10	0.01	5.28E-08
ADGRE2	rs1260326	2	27508073	C	T	0.61	0.05	0.01	4.43E-06
ADGRE2	rs34452456	6	32482901	C	T	0.33	0.06	0.01	1.09E-06
ADGRE2	rs56278466	10	17833858	G	T	0.66	0.12	0.01	1.38E-26
ADGRE2	rs950802	11	60385111	A	G	0.31	-0.06	0.01	5.82E-07
ADGRE2	rs78689694	11	126364925	C	G	0.13	-0.07	0.01	1.39E-05
ADGRE2	rs3184504	12	111446804	C	T	0.52	-0.10	0.01	3.16E-20
ADGRE2	rs186021206	17	7166093	A	G	0.01	1.09	0.05	2.77E-50
ADGRE2	rs7251607	19	14396179	C	A	0.75	0.29	0.01	4.41E-115
ADGRE5	rs61801010	1	161572445	G	A	0.11	0.10	0.01	1.00E-07
ADGRE5	rs1260326	2	27508073	C	T	0.61	0.05	0.01	3.75E-06
ADGRE5	rs199866843	3	99039510	A	T	0.64	0.05	0.01	2.28E-05
ADGRE5	rs10093	6	32641396	G	C	0.38	0.06	0.01	4.16E-08
ADGRE5	rs56278466	10	17833858	G	T	0.66	0.14	0.01	1.75E-33
ADGRE5	rs950802	11	60385111	A	G	0.31	-0.07	0.01	3.72E-08
ADGRE5	rs78689694	11	126364925	C	G	0.13	-0.07	0.01	2.19E-05
ADGRE5	rs3184504	12	111446804	C	T	0.52	-0.10	0.01	1.44E-21
ADGRE5	rs186021206	17	7166093	A	G	0.01	1.17	0.05	1.44E-56
ADGRE5	rs139113505	19	14388804	T	C	0.02	-0.71	0.03	2.29E-63
ADGRE5	rs6065904	20	45906012	A	G	0.22	0.07	0.01	6.09E-07
ADGRG1	rs1047891	2	210675783	A	C	0.31	0.06	0.01	2.14E-07
ADGRG1	rs10935473	3	98698056	T	G	0.44	-0.08	0.01	2.45E-12
ADGRG1	rs916951	5	136048754	G	A	0.51	0.05	0.01	4.13E-06
ADGRG1	rs28393425	6	32484785	G	A	0.18	-0.07	0.01	8.84E-07
ADGRG1	rs2519093	9	133266456	T	C	0.18	-0.11	0.01	1.98E-15
ADGRG1	rs56278466	10	17833858	G	T	0.66	0.13	0.01	1.35E-31

ADGRG1	rs376888155	11	86623516	A	G	0.31	-0.07	0.01	6.17E-08
ADGRG1	rs3184504	12	111446804	C	T	0.52	-0.08	0.01	2.21E-13
ADGRG1	rs733464	16	57648363	T	C	0.41	0.07	0.01	1.75E-10
ADGRG1	rs186021206	17	7166093	A	G	0.01	0.47	0.05	8.64E-10
ADGRG1	rs738409	22	43928847	G	C	0.22	0.06	0.01	3.93E-06
ADGRG2	rs755740174	1	59192759	A	T	0.29	0.08	0.01	1.41E-10
ADGRG2	rs13107325	4	102267552	T	C	0.08	0.10	0.01	1.07E-06
ADGRG2	rs115912456	5	83519339	G	A	0.04	-0.15	0.02	4.43E-08
ADGRG2	rs7836422	8	38992502	T	C	0.39	0.07	0.01	4.51E-12
ADGRG2	-	9	98800544	C	T	0.10	0.08	0.01	8.71E-06
ADGRG2	rs8176719	9	133257521	C	T	0.34	0.05	0.01	1.08E-05
ADGRG2	rs74612335	11	126368738	C	T	0.13	-0.09	0.01	3.66E-09
ADGRG2	rs186021206	17	7166093	A	G	0.01	0.52	0.05	4.39E-13
ADH4	rs1800759	4	99144358	G	T	0.61	0.13	0.01	1.07E-30
ADH4	rs112875651	8	125494452	A	G	0.39	-0.05	0.01	4.19E-06
ADH4	rs4979372	9	114377802	C	T	0.49	-0.06	0.01	1.26E-08
ADH4	rs10883451	10	100164661	C	T	0.50	-0.05	0.01	4.44E-06
ADH4	rs11604580	11	94119714	A	G	0.11	-0.08	0.01	2.47E-05
ADH4	rs3747207	22	43928975	A	G	0.22	0.10	0.01	7.75E-14
ADM	rs150777506	2	162200414	G	T	0.01	-0.51	0.03	8.66E-22
ADM	rs9264638	6	31270541	A	G	0.64	-0.05	0.01	1.59E-05
ADM	rs2403310	11	10341724	C	G	0.47	0.16	0.01	5.41E-51
AFP	rs12059956	1	171094121	A	G	0.42	-0.05	0.01	1.67E-05
AFP	rs4149909	1	241860596	G	A	0.03	-0.14	0.02	5.31E-06
AFP	rs11900031	2	17749607	G	A	0.11	-0.42	0.01	5.03E-128
AFP	rs3018026	2	67428185	T	C	0.69	-0.09	0.01	2.28E-13
AFP	-	3	8929643	A	T	0.76	0.09	0.01	3.11E-12
AFP	rs62280667	3	101365760	C	T	0.34	-0.13	0.01	6.44E-31
AFP	rs59950280	4	3450618	A	G	0.33	0.09	0.01	8.51E-15
AFP	rs12506899	4	73453566	G	T	0.40	-0.19	0.01	1.67E-67
AFP	rs2602874	4	99118245	T	A	0.58	-0.07	0.01	2.23E-11
AFP	rs796736099	6	42956957	A	T	0.45	-0.05	0.01	2.41E-05
AFP	rs4732620	8	28067687	C	T	0.55	-0.05	0.01	2.16E-05
AFP	rs62576492	9	132343321	G	A	0.11	0.14	0.01	8.14E-16
AFP	rs35751397	10	63154256	A	T	0.47	-0.05	0.01	3.06E-06
AFP	rs10883567	10	100996022	C	T	0.49	-0.06	0.01	2.92E-08
AFP	-	11	1665769	C	G	0.22	-0.09	0.01	1.08E-11
AFP	rs11418587	11	108236534	A	T	0.33	0.07	0.01	3.55E-07
AFP	rs7310409	12	120987058	G	A	0.62	0.10	0.01	2.47E-22
AFP	rs145058546	15	29269402	C	T	0.00	-0.42	0.06	2.38E-05
AFP	rs560191	15	43475576	C	G	0.30	0.12	0.01	2.92E-26
AFP	rs144268704	15	63500022	T	A	0.34	0.06	0.01	7.68E-07
AFP	rs17689024	16	79706081	C	G	0.31	-0.05	0.01	4.21E-06
AFP	rs11642010	16	89778703	C	T	0.09	-0.08	0.01	8.58E-06
AFP	-	17	35934977	T	A	0.15	0.09	0.01	6.55E-09
AFP	rs4794048	17	47685707	C	A	0.48	0.07	0.01	2.67E-11
AFP	rs34349423	17	49782701	C	A	0.63	-0.06	0.01	6.56E-07
AFP	rs1801689	17	66214462	C	A	0.03	-0.17	0.02	1.07E-07
AFP	rs35103294	17	75814856	T	C	0.63	0.05	0.01	6.99E-06
AFP	rs2548458	19	48706068	T	C	0.54	-0.05	0.01	5.11E-06
AFP	rs6116934	20	5751157	C	T	0.22	0.09	0.01	2.49E-13
AFP	-	20	32267968	C	T	0.17	-0.07	0.01	1.94E-06
AFP	rs6007594	22	45332489	A	G	0.26	0.13	0.01	7.93E-29
AGER	rs66906568	1	10662242	C	T	0.30	-0.07	0.01	3.23E-09
AGER	rs10753239	1	30889377	C	T	0.46	-0.05	0.01	3.59E-06
AGER	rs1289813	1	117279693	A	G	0.28	0.05	0.01	1.85E-05

AGER	rs6664840	1	175159590	G	A	0.47	-0.07	0.01	3.66E-11
AGER	rs2137715	4	89089309	C	A	0.39	0.07	0.01	1.14E-08
AGER	rs13186378	5	52975201	G	C	0.32	0.07	0.01	6.07E-10
AGER	rs204993	6	32187804	G	A	0.32	-0.17	0.01	5.62E-47
AGER	rs7820838	8	32548461	C	T	0.78	0.07	0.01	7.75E-08
AGER	rs11243421	9	131550651	C	T	0.56	-0.13	0.01	5.80E-32
AGER	rs2487928	10	30034963	A	G	0.45	-0.05	0.01	3.79E-06
AGER	rs3028371	10	69496320	C	T	0.53	-0.05	0.01	1.49E-05
AGER	rs35393359	12	514290	T	C	0.17	-0.09	0.01	2.73E-10
AGER	rs11407068	12	10091655	A	T	0.78	0.06	0.01	5.03E-06
AGER	rs1896354	12	114935160	G	A	0.43	0.07	0.01	6.52E-09
AGER	rs55703124	13	109270326	C	T	0.73	-0.06	0.01	1.08E-06
AGER	rs10152355	15	58378979	A	C	0.45	0.07	0.01	1.74E-08
AGER	rs34262842	16	20344489	G	A	0.17	-0.07	0.01	1.29E-05
AGER	-	19	32102546	G	A	0.69	0.06	0.01	3.57E-06
AGR2	rs115317719	6	32444228	T	G	0.19	0.07	0.01	7.27E-07
AGR2	rs7807123	7	16816925	T	C	0.57	0.10	0.01	4.13E-19
AGR2	rs7826120	8	58459166	C	T	0.67	0.06	0.01	1.91E-07
AGR2	rs28633254	11	1199373	C	T	0.42	-0.06	0.01	3.91E-06
AGR2	rs601338	19	48703417	A	G	0.51	-0.06	0.01	6.23E-07
AGR3	rs58198139	5	156972028	T	C	0.64	0.08	0.01	6.94E-11
AGR3	rs9271956	6	32629536	C	T	0.83	0.07	0.01	1.04E-05
AGR3	rs34954997	19	44914381	G	C	0.24	0.08	0.01	1.10E-08
AGR3	rs12983058	19	51139527	T	G	0.33	-0.05	0.01	1.61E-05
AGRN	rs3121560	1	1032846	A	C	0.34	-0.37	0.01	8.16E-226
AGRN	rs551319785	3	49942496	C	A	0.14	0.09	0.01	5.25E-07
AGRN	rs13107325	4	102267552	T	C	0.08	0.09	0.01	1.13E-05
AGRN	rs7654228	4	118378823	C	T	0.41	-0.09	0.01	7.38E-15
AGRN	rs4240624	8	9326721	A	G	0.91	-0.09	0.01	1.94E-06
AGRN	rs1149605	11	76774172	C	T	0.17	-0.09	0.01	3.25E-09
AGRN	rs6061230	20	62371133	T	A	0.36	0.11	0.01	2.28E-23
AGRP	rs12239737	1	62620330	A	T	0.34	-0.05	0.01	6.81E-06
AGRP	rs1260326	2	27508073	C	T	0.61	-0.05	0.01	4.55E-06
AGRP	rs778393561	6	32657458	G	A	0.35	-0.08	0.01	3.92E-09
AGRP	rs56393506	6	160668275	T	C	0.17	0.09	0.01	3.24E-09
AGRP	rs76722925	8	20073987	G	A	0.08	-0.11	0.01	4.58E-08
AGRP	-	8	125492141	C	T	0.60	-0.08	0.01	6.96E-10
AGRP	rs1570581	9	124471780	G	A	0.47	0.05	0.01	1.05E-05
AGRP	rs964184	11	116778201	C	G	0.87	-0.15	0.01	8.03E-20
AGRP	rs188707867	16	67481485	G	C	0.01	-0.46	0.05	4.74E-11
AGRP	rs740516	17	69086821	G	C	0.15	-0.07	0.01	1.48E-05
AGRP	rs34954997	19	44914381	G	C	0.24	0.12	0.01	9.54E-22
AGXT	rs1497406	1	16178825	G	A	0.58	0.06	0.01	1.61E-08
AGXT	rs4361977	1	155103603	T	C	0.43	-0.06	0.01	1.23E-07
AGXT	rs55649245	2	240854128	A	G	0.31	-0.28	0.01	1.29E-123
AGXT	rs4835265	4	145900258	A	C	0.16	0.13	0.01	7.17E-17
AGXT	rs6888304	5	31020414	G	A	0.28	-0.05	0.01	2.38E-05
AGXT	rs112875651	8	125494452	A	G	0.39	-0.05	0.01	4.61E-06
AGXT	rs10883451	10	100164661	C	T	0.50	-0.05	0.01	4.78E-06
AGXT	rs28929474	14	94378610	T	C	0.02	0.19	0.03	8.31E-07
AGXT	rs9959832	18	58419588	T	C	0.80	-0.10	0.01	7.22E-13
AGXT	rs10405357	19	54255803	C	T	0.44	-0.06	0.01	1.38E-06
AGXT	rs3747207	22	43928975	A	G	0.22	0.08	0.01	6.43E-09
AHCY	rs142714816	4	73408646	G	A	0.00	0.87	0.13	2.82E-05
AHCY	rs13043752	20	34295502	A	G	0.02	-1.36	0.03	3.72E-201
AHSP	rs875741	5	173860848	A	G	0.41	-0.05	0.01	3.35E-06

AHSP	rs80215559	6	25917997	C	T	0.08	0.13	0.01	4.92E-11
AHSP	rs11759553	6	135101158	T	A	0.27	-0.06	0.01	6.60E-06
AHSP	rs590856	6	139523292	A	G	0.55	-0.08	0.01	5.72E-13
AHSP	rs6592965	7	50360284	A	G	0.45	-0.06	0.01	3.67E-09
AHSP	rs10742583	11	5227411	A	G	0.85	0.12	0.01	2.49E-14
AHSP	rs7194649	16	165107	A	C	0.11	-0.12	0.01	5.84E-12
AHSP	rs771133280	17	28843137	T	C	0.20	0.08	0.01	2.03E-08
AHSP	rs570223913	17	76382231	G	T	0.47	-0.11	0.01	3.31E-25
AHSP	rs390408	22	21557983	G	A	0.82	0.09	0.01	6.08E-10
AHSP	rs855791	22	37066896	G	A	0.56	0.08	0.01	2.19E-13
AIF1	rs2261033	6	31635814	G	A	0.43	-0.11	0.01	6.29E-21
AIFM1	rs1354034	3	56815721	C	T	0.60	0.06	0.01	2.37E-06
AIFM1	rs342293	7	106731773	G	C	0.46	-0.05	0.01	2.78E-05
AKR1B1	rs2229542	7	134450869	C	T	0.01	-0.58	0.03	5.92E-31
AKR1B1	rs6993770	8	105569300	T	A	0.29	-0.06	0.01	1.47E-06
AKR1B1	rs72481564	14	105886393	T	C	0.23	0.08	0.01	7.00E-07
AKR1C4	rs11253048	10	5219196	G	A	0.18	-0.07	0.01	1.73E-05
AKT1S1	rs1354034	3	56815721	C	T	0.60	0.07	0.01	6.43E-09
AKT1S1	rs342293	7	106731773	G	C	0.46	-0.06	0.01	1.14E-06
AKT1S1	rs10733789	10	63188924	C	T	0.31	0.07	0.01	1.84E-07
AKT3	rs4515770	1	243523611	C	T	0.22	0.06	0.01	1.52E-05
AKT3	rs7916868	10	63229171	T	A	0.50	0.05	0.01	1.23E-05
ALCAM	rs34926152	3	105545332	T	G	0.05	-0.30	0.02	1.26E-35
ALCAM	rs9274626	6	32668263	C	T	0.68	-0.06	0.01	3.78E-07
ALCAM	rs199922514	8	9325592	G	A	0.91	-0.11	0.01	9.10E-10
ALCAM	rs9411365	9	133242881	G	C	0.07	0.10	0.01	3.81E-06
ALCAM	rs59822672	11	126401080	C	G	0.08	-0.09	0.01	9.99E-06
ALCAM	rs11045826	12	21181665	C	T	0.16	0.09	0.01	9.32E-10
ALCAM	rs904628	12	120904281	A	G	0.48	-0.06	0.01	5.56E-09
ALCAM	rs11075299	16	16152307	G	T	0.29	0.10	0.01	1.43E-16
ALCAM	rs186021206	17	7166093	A	G	0.01	0.84	0.05	5.87E-32
ALCAM	rs7224668	17	81261988	C	T	0.56	-0.05	0.01	3.94E-06
ALCAM	rs2638282	19	48710576	A	G	0.53	0.06	0.01	5.53E-08
ALCAM	rs3747207	22	43928975	A	G	0.22	0.08	0.01	6.57E-10
ALDH1A1	rs7775698	6	135097497	T	C	0.26	-0.09	0.01	8.80E-14
ALDH1A1	rs348452	9	72938469	T	C	0.27	0.09	0.01	7.52E-13
ALDH1A1	rs3747207	22	43928975	A	G	0.22	0.07	0.01	4.89E-08
ALDH3A1	rs887241	17	19742625	C	A	0.66	0.48	0.01	0.00E+00
ALPP	rs761164	1	39885979	G	A	0.15	0.07	0.01	4.21E-06
ALPP	rs12743084	1	155188692	G	C	0.54	0.06	0.01	1.78E-09
ALPP	rs12478529	2	232421944	T	C	0.23	0.50	0.01	0.00E+00
ALPP	rs71626052	3	138008603	T	A	0.47	-0.05	0.01	2.49E-06
ALPP	rs2076295	6	7562999	G	T	0.45	0.04	0.01	2.39E-05
ALPP	rs55792059	6	53487408	A	C	0.19	0.08	0.01	2.25E-08
ALPP	rs117847945	6	79152613	G	A	0.02	0.17	0.02	4.10E-06
ALPP	rs4236709	8	32552592	A	G	0.78	0.05	0.01	2.07E-05
ALPP	rs3936578	9	106719746	C	A	0.49	-0.08	0.01	5.34E-15
ALPP	rs782134971	9	133264504	G	C	0.25	-0.24	0.01	3.11E-96
ALPP	rs7167092	15	31334670	C	T	0.35	-0.05	0.01	7.65E-07
ALPP	rs186021206	17	7166093	A	G	0.01	0.46	0.05	1.12E-10
ALPP	rs516246	19	48702915	T	C	0.51	-0.12	0.01	1.12E-30
AMBN	-	4	70513213	T	A	0.76	0.09	0.01	1.69E-10
AMBP	rs61747728	1	179557079	T	C	0.04	0.15	0.02	1.44E-07
AMBP	rs75166367	2	162107791	A	G	0.06	-0.11	0.01	5.32E-06
AMBP	rs406658	6	32028747	A	C	0.11	0.15	0.01	5.47E-18
AMBP	rs10265221	7	151717243	C	T	0.29	0.05	0.01	6.23E-06

AMBP	rs147941577	9	114069709	G	A	0.00	-1.68	0.07	5.10E-63
AMBP	rs1169294	12	120988791	A	G	0.31	0.06	0.01	2.75E-06
AMBP	rs28929474	14	94378610	T	C	0.02	0.18	0.02	5.36E-06
AMBP	rs34262842	16	20344489	G	A	0.17	-0.06	0.01	1.39E-05
AMBP	rs704	17	28367840	A	G	0.47	-0.11	0.01	3.15E-24
AMIGO2	-	1	43812246	G	A	0.29	0.06	0.01	3.54E-06
AMIGO2	rs1524668	2	9557243	C	A	0.67	0.06	0.01	2.40E-07
AMIGO2	rs1354034	3	56815721	C	T	0.60	-0.14	0.01	4.91E-36
AMIGO2	rs10935473	3	98698056	T	G	0.44	-0.18	0.01	4.21E-54
AMIGO2	rs9272413	6	32637210	C	T	0.24	-0.07	0.01	2.96E-06
AMIGO2	rs385893	9	4763176	C	T	0.52	0.06	0.01	1.27E-07
AMIGO2	rs2519093	9	133266456	T	C	0.18	-0.16	0.01	5.11E-30
AMIGO2	rs174564	11	61820833	G	A	0.35	0.06	0.01	2.33E-07
AMIGO2	rs4055121	11	126362442	T	C	0.13	-0.12	0.01	4.00E-12
AMIGO2	rs215384	12	47366418	G	C	0.41	0.16	0.01	4.82E-42
AMIGO2	rs2242497	12	56598846	G	C	0.36	-0.06	0.01	1.90E-06
AMIGO2	rs10774625	12	111472415	G	A	0.50	-0.08	0.01	1.32E-12
AMIGO2	rs186021206	17	7166093	A	G	0.01	0.98	0.05	1.21E-36
AMIGO2	-	20	49483100	C	T	0.24	-0.06	0.01	2.16E-05
AMN	rs629301	1	109275684	T	G	0.78	-0.07	0.01	1.59E-07
AMN	rs7979473	12	120982457	G	A	0.62	0.05	0.01	1.21E-05
AMN	rs28929474	14	94378610	T	C	0.02	0.68	0.03	2.19E-62
AMN	rs2295829	14	102922666	C	G	0.39	0.77	0.01	0.00E+00
AMN	rs1801689	17	66214462	C	A	0.03	0.24	0.02	4.79E-14
AMY2A	rs78245241	1	103793104	G	T	0.04	-0.96	0.02	4.17E-201
AMY2A	rs291091	1	206940334	C	T	0.45	0.05	0.01	2.63E-05
AMY2A	-	5	44135692	C	T	0.20	0.07	0.01	1.88E-06
AMY2A	rs55901622	6	126836293	G	A	0.27	0.05	0.01	1.12E-05
AMY2A	rs1561929	8	128555127	T	C	0.88	0.08	0.01	1.62E-05
AMY2A	rs550057	9	133271182	T	C	0.26	-0.15	0.01	3.18E-34
AMY2A	rs11054116	12	10970738	A	G	0.24	-0.07	0.01	1.95E-08
AMY2A	rs533406	16	88908452	G	A	0.38	-0.07	0.01	4.00E-10
AMY2A	rs2638282	19	48710576	A	G	0.53	0.05	0.01	1.48E-06
AMY2B	rs12080068	1	103787201	A	C	0.09	0.65	0.01	3.38E-222
AMY2B	rs55901622	6	126836293	G	A	0.27	0.05	0.01	1.58E-05
AMY2B	rs1561929	8	128555127	T	C	0.88	0.08	0.01	6.81E-06
AMY2B	rs550057	9	133271182	T	C	0.25	-0.15	0.01	4.06E-34
AMY2B	rs56278466	10	17833858	G	T	0.66	0.05	0.01	4.53E-06
AMY2B	rs28456	11	61822009	G	A	0.31	0.05	0.01	1.81E-05
AMY2B	rs11054116	12	10970738	A	G	0.24	-0.07	0.01	4.35E-07
AMY2B	rs533406	16	88908452	G	A	0.38	-0.08	0.01	2.17E-11
AMY2B	rs2638282	19	48710576	A	G	0.53	0.05	0.01	1.69E-06
ANG	rs61791757	3	136186483	A	G	0.23	-0.06	0.01	4.46E-07
ANG	rs11047261	12	24104294	G	A	0.04	0.12	0.02	2.31E-06
ANG	rs8008227	14	20688145	A	G	0.12	0.66	0.01	0.00E+00
ANG	-	17	47612663	C	T	0.48	-0.05	0.01	1.74E-05
ANGPT1	rs12041331	1	156899922	A	G	0.09	-0.10	0.01	1.48E-06
ANGPT1	rs13412535	2	224010157	A	G	0.23	-0.08	0.01	6.87E-09
ANGPT1	rs7618405	3	18209017	A	C	0.21	-0.07	0.01	9.47E-07
ANGPT1	rs1354034	3	56815721	C	T	0.60	-0.09	0.01	6.27E-14
ANGPT1	rs3792366	3	123121029	A	G	0.59	-0.05	0.01	1.24E-05
ANGPT1	rs755492124	4	101856385	T	G	0.39	-0.07	0.01	1.46E-10
ANGPT1	rs10058074	5	132350453	A	G	0.48	-0.06	0.01	4.30E-08
ANGPT1	rs3128984	6	31456751	G	A	0.07	-0.13	0.01	8.86E-09
ANGPT1	rs146842698	6	70618570	A	T	0.31	-0.07	0.01	7.45E-08
ANGPT1	rs6961069	7	80589645	T	C	0.40	0.08	0.01	5.75E-11

ANGPT1	rs6993770	8	105569300	T	A	0.29	-0.19	0.01	6.03E-55
ANGPT1	rs9410207	9	88789884	C	T	0.07	-0.10	0.01	1.73E-05
ANGPT1	rs61751937	9	110549951	C	G	0.03	0.16	0.02	5.81E-06
ANGPT1	rs11251714	10	3097027	G	A	0.36	-0.06	0.01	3.75E-06
ANGPT1	rs7896518	10	63344740	G	A	0.42	0.10	0.01	1.94E-17
ANGPT1	rs71474568	10	102589091	G	T	0.34	0.07	0.01	1.50E-09
ANGPT1	rs4759076	12	54336088	C	T	0.46	0.06	0.01	5.79E-08
ANGPT1	rs61978213	14	70187041	A	G	0.04	0.13	0.02	4.28E-06
ANGPT1	rs59001897	15	64868193	A	T	0.17	0.08	0.01	8.05E-08
ANGPT1	rs151234	16	28494339	C	G	0.13	0.08	0.01	1.12E-05
ANGPT1	rs12445050	16	81837364	T	C	0.14	0.12	0.01	1.00E-13
ANGPT1	rs1654425	19	55027612	C	T	0.83	0.19	0.01	4.36E-35
ANGPT1	rs3827978	20	19300647	T	C	0.35	0.08	0.01	5.01E-12
ANGPT2	rs13107325	4	102267552	T	C	0.08	0.13	0.01	5.02E-09
ANGPT2	rs1968586	8	6421990	T	C	0.36	-0.16	0.01	1.35E-40
ANGPT2	rs4841132	8	9326086	G	A	0.91	-0.11	0.01	1.21E-08
ANGPT2	-	9	133255468	T	G	0.06	0.22	0.02	3.06E-20
ANGPT2	rs34434834	11	126437901	A	G	0.03	0.24	0.02	2.00E-14
ANGPT2	rs2137537	12	70719307	C	T	0.55	-0.05	0.01	1.44E-05
ANGPT2	rs186021206	17	7166093	A	G	0.01	0.86	0.05	1.74E-29
ANGPTL1	rs12753251	1	178560763	A	G	0.53	-0.28	0.01	1.17E-138
ANGPTL1	-	6	32215801	C	T	0.17	0.07	0.01	8.58E-06
ANGPTL1	rs1137654	9	98808054	T	A	0.09	-0.10	0.01	1.94E-06
ANGPTL1	rs28929474	14	94378610	T	C	0.02	0.19	0.02	1.64E-06
ANGPTL1	rs186021206	17	7166093	A	G	0.01	0.33	0.05	2.73E-05
ANGPTL1	rs149394327	17	66232877	C	G	0.03	0.25	0.02	3.05E-14
ANGPTL1	rs8103017	19	55487775	G	C	0.29	0.06	0.01	3.30E-06
ANGPTL2	rs2856674	6	32691868	G	A	0.15	-0.09	0.01	5.31E-09
ANGPTL2	rs4141100	7	7237323	A	G	0.61	0.06	0.01	3.43E-08
ANGPTL2	rs2142306	8	133458388	C	T	0.41	0.05	0.01	9.25E-06
ANGPTL2	rs117360431	9	127053216	A	G	0.02	0.18	0.03	6.88E-06
ANGPTL2	rs138260315	11	12015556	T	G	0.18	-0.12	0.01	3.15E-15
ANGPTL2	rs55799523	19	55488178	A	C	0.27	0.26	0.01	1.07E-90
ANGPTL2	rs181210490	20	3715330	A	G	0.01	0.58	0.03	5.29E-28
ANGPTL3	rs763015008	1	62508787	T	C	0.35	-0.28	0.01	1.10E-151
ANGPTL3	rs3916027	8	19967357	A	G	0.26	0.08	0.01	9.05E-13
ANGPTL3	rs10808546	8	125483576	T	C	0.45	0.05	0.01	3.74E-07
ANGPTL3	rs964184	11	116778201	C	G	0.87	-0.12	0.01	3.08E-15
ANGPTL3	rs28929474	14	94378610	T	C	0.02	0.24	0.02	5.39E-11
ANGPTL3	rs72786786	16	56951602	A	G	0.31	0.05	0.01	2.09E-05
ANGPTL3	rs1065853	19	44909976	T	G	0.08	0.13	0.01	4.70E-12
ANGPTL4	rs7252574	19	8376895	T	C	0.51	0.28	0.01	1.34E-132
ANGPTL7	rs28991009	1	11193627	T	G	0.01	-2.16	0.04	1.03E-286
ANGPTL7	rs6764769	3	141381438	G	A	0.44	-0.06	0.01	1.34E-07
ANGPTL7	rs7019896	9	33113198	T	G	0.93	0.11	0.01	1.24E-06
ANGPTL7	rs529700	10	17843438	C	T	0.51	0.07	0.01	2.95E-08
ANGPTL7	rs112771035	11	126355981	G	C	0.07	-0.12	0.01	5.37E-09
ANGPTL7	rs186021206	17	7166093	A	G	0.01	0.76	0.05	6.96E-25
ANGPTL7	rs704	17	28367840	A	G	0.47	0.06	0.01	4.80E-08
ANKRD54	rs35496925	22	38028600	G	A	0.06	0.14	0.02	7.92E-09
ANPEP	rs13135092	4	102276925	G	A	0.08	0.09	0.01	4.42E-06
ANPEP	rs4835265	4	145900258	A	C	0.16	0.07	0.01	5.57E-06
ANPEP	rs4055121	11	126362442	T	C	0.13	-0.10	0.01	5.47E-11
ANPEP	rs2297066	14	103100498	G	C	0.24	0.06	0.01	5.44E-06
ANPEP	rs1042499	15	89785074	C	T	0.06	0.81	0.02	5.07E-246
ANPEP	rs186021206	17	7166093	A	G	0.01	0.64	0.05	1.07E-18

ANPEP	rs12373325	18	58420416	C	T	0.80	-0.06	0.01	2.27E-05
ANXA10	rs139558368	1	155225280	A	T	0.43	0.05	0.01	1.90E-05
ANXA11	rs9281986	6	32633960	G	A	0.17	-0.10	0.01	2.66E-10
ANXA11	rs3847326	10	63572099	G	A	0.48	-0.05	0.01	2.21E-05
ANXA11	rs2784773	10	80160044	C	T	0.64	0.07	0.01	2.96E-08
ANXA3	rs1354034	3	56815721	C	T	0.60	0.05	0.01	2.83E-05
ANXA3	rs146728751	4	78579081	A	G	0.00	-1.18	0.09	5.36E-17
ANXA3	rs6993770	8	105569300	T	A	0.29	-0.07	0.01	2.26E-07
ANXA3	rs7896518	10	63344740	G	A	0.42	0.06	0.01	2.79E-07
ANXA3	rs10418046	19	53824615	G	T	0.21	0.07	0.01	3.77E-06
ANXA4	rs1354034	3	56815721	C	T	0.60	0.05	0.01	1.15E-05
ANXA4	rs7896518	10	63344740	G	A	0.42	0.07	0.01	4.76E-09
ANXA4	rs200309755	17	29518756	T	C	0.54	0.05	0.01	2.83E-05
ANXA5	rs2306411	4	121677480	G	A	0.29	-0.12	0.01	1.04E-20
AOC1	rs1049742	7	150857465	T	C	0.08	0.49	0.01	5.35E-121
AOC3	rs2270894	3	9933702	G	C	0.20	0.08	0.01	1.19E-09
AOC3	rs13135092	4	102276925	G	A	0.08	0.09	0.01	1.58E-06
AOC3	rs75901433	6	32531373	G	A	0.56	0.06	0.01	9.83E-08
AOC3	rs767298	7	7234953	T	G	0.63	-0.07	0.01	8.43E-10
AOC3	rs9411365	9	133242881	G	C	0.07	0.10	0.01	1.19E-06
AOC3	rs56278466	10	17833858	G	T	0.66	0.07	0.01	7.39E-11
AOC3	rs3967200	11	126362490	T	C	0.13	-0.10	0.01	6.72E-12
AOC3	rs1042704	14	22843385	A	G	0.22	-0.09	0.01	6.67E-14
AOC3	rs12913657	15	50708771	C	T	0.15	0.08	0.01	1.65E-07
AOC3	rs186021206	17	7166093	A	G	0.01	0.59	0.05	9.05E-15
AOC3	rs33986943	17	42852620	A	G	0.11	-0.85	0.01	0.00E+00
APBB1IP	rs3129754	6	32615269	G	A	0.42	0.05	0.01	1.74E-05
APBB1IP	rs7461753	8	143907330	C	A	0.42	0.06	0.01	4.77E-07
APBB1IP	rs2992335	10	26439005	G	C	0.60	-0.22	0.01	2.09E-82
APBB1IP	rs11671654	19	11903540	G	C	0.49	0.15	0.01	8.26E-41
APBB1IP	rs10418046	19	53824615	G	T	0.21	0.11	0.01	1.91E-15
APEX1	rs1354034	3	56815721	C	T	0.60	0.06	0.01	3.92E-08
APEX1	rs111338191	12	111388673	T	A	0.52	-0.05	0.01	5.45E-06
APEX1	rs1130409	14	20456995	G	T	0.48	-0.39	0.01	5.86E-276
APEX1	rs4632248	19	53821741	T	G	0.21	0.07	0.01	7.66E-07
APLP1	rs13412535	2	224010157	A	G	0.23	0.07	0.01	4.14E-08
APLP1	rs4602861	8	105578478	G	A	0.27	0.10	0.01	1.10E-13
APLP1	rs2229498	10	69097096	A	G	0.84	-0.11	0.01	2.01E-12
APLP1	rs12609736	19	55033916	G	A	0.83	-0.11	0.01	4.91E-12
APLP1	rs6081568	20	19318144	G	C	0.35	-0.06	0.01	6.28E-07
APOH	rs1801689	17	66214462	C	A	0.03	-1.44	0.02	0.00E+00
APOM	rs1260326	2	27508073	C	T	0.61	-0.09	0.01	2.29E-15
APOM	rs805258	6	31665775	T	C	0.03	-0.77	0.02	2.77E-124
APOM	rs115849089	8	20054859	A	G	0.12	0.09	0.01	6.47E-07
APOM	rs56332871	15	96171587	A	C	0.27	0.06	0.01	1.10E-05
APOM	rs247616	16	56955678	T	C	0.33	0.13	0.01	4.05E-27
APOM	rs112001035	17	68827664	A	G	0.06	0.13	0.02	8.29E-08
APOM	rs4121823	18	49617853	A	T	0.84	0.07	0.01	2.35E-05
APOM	rs1800961	20	44413724	T	C	0.03	-0.24	0.02	7.68E-15
APOM	rs3747207	22	43928975	A	G	0.22	-0.06	0.01	1.21E-05
APP	rs12086222	1	156899838	C	G	0.11	0.08	0.01	6.95E-06
APP	rs13412535	2	224010157	A	G	0.23	-0.06	0.01	4.18E-06
APP	rs7618405	3	18209017	A	C	0.20	-0.06	0.01	2.67E-05
APP	rs1354034	3	56815721	C	T	0.60	-0.10	0.01	4.42E-18
APP	rs755492124	4	101856385	T	G	0.39	-0.07	0.01	1.26E-09
APP	rs274555	5	132387259	T	C	0.40	0.06	0.01	8.35E-08

APP	rs138614581	6	31436393	T	A	0.08	-0.12	0.01	5.99E-08
APP	rs1917342	6	70640051	G	A	0.30	-0.06	0.01	3.59E-06
APP	rs6961069	7	80589645	T	C	0.40	0.07	0.01	3.10E-09
APP	rs6993770	8	105569300	T	A	0.29	-0.08	0.01	1.86E-11
APP	rs10820606	9	96430637	C	A	0.23	-0.07	0.01	1.44E-07
APP	rs7896518	10	63344740	G	A	0.42	0.10	0.01	1.81E-19
APP	rs2229498	10	69097096	A	G	0.84	0.11	0.01	1.36E-13
APP	rs35277580	12	6185368	A	G	0.49	-0.05	0.01	8.04E-06
APP	rs61978213	14	70187041	A	G	0.04	0.13	0.02	1.66E-05
APP	rs59001897	15	64868193	A	T	0.17	0.08	0.01	7.84E-07
APP	rs12445050	16	81837364	T	C	0.14	0.11	0.01	8.54E-12
APP	rs1654425	19	55027612	C	T	0.83	0.17	0.01	1.15E-28
APP	rs6081565	20	19307260	A	G	0.35	0.08	0.01	1.25E-10
APP	rs8131895	21	26131210	A	C	0.65	-0.07	0.01	2.69E-09
APRT	-	2	190454999	T	A	0.52	0.05	0.01	5.49E-06
APRT	rs1354034	3	56815721	C	T	0.60	0.10	0.01	2.20E-17
APRT	rs854572	7	95325384	G	C	0.53	0.05	0.01	3.67E-06
APRT	rs11397106	10	63572305	T	C	0.47	-0.05	0.01	2.79E-05
APRT	rs10876550	12	54318524	A	G	0.56	0.06	0.01	7.06E-08
APRT	rs74403919	14	91777634	A	T	0.17	0.07	0.01	1.94E-05
APRT	rs111433410	16	88802787	T	C	0.01	-0.37	0.04	4.06E-10
APRT	rs704	17	28367840	A	G	0.47	-0.08	0.01	2.29E-13
AREG	rs1691273	4	74457928	T	C	0.69	-0.18	0.01	3.40E-46
AREG	rs712831	7	55175089	C	T	0.79	0.07	0.01	8.24E-07
AREG	-	9	133263362	G	A	0.18	-0.09	0.01	2.49E-09
AREG	rs492602	19	48703160	G	A	0.51	-0.07	0.01	1.59E-09
ARG1	rs2781668	6	131576138	T	C	0.17	-0.24	0.01	6.34E-58
ARG1	rs592423	6	139519556	C	A	0.55	0.09	0.01	6.45E-17
ARG1	rs6592965	7	50360284	A	G	0.45	0.06	0.01	1.96E-07
ARG1	rs56074687	11	16589928	C	T	0.29	0.05	0.01	1.96E-05
ARG1	rs62108438	19	12889433	C	T	0.39	0.10	0.01	1.85E-17
ARHGAP1	rs6993770	8	105569300	T	A	0.29	-0.09	0.01	1.13E-12
ARHGAP1	rs12245149	10	63561387	A	C	0.49	-0.06	0.01	1.17E-07
ARHGAP1	-	16	72045090	T	G	0.18	0.15	0.01	8.21E-24
ARHGAP25	rs13413887	2	68769536	G	A	0.76	0.11	0.01	5.20E-15
ARHGAP25	rs6993770	8	105569300	T	A	0.29	-0.07	0.01	4.23E-07
ARSA	rs114165349	1	26695422	C	G	0.02	0.17	0.02	2.32E-07
ARSA	rs687339	3	136213517	T	C	0.77	0.06	0.01	1.03E-07
ARSA	-	6	130065067	G	A	0.68	0.05	0.01	1.72E-06
ARSA	rs10745925	12	101825121	C	T	0.29	-0.26	0.01	1.52E-127
ARSA	rs145078947	14	93186629	T	G	0.00	0.63	0.06	9.05E-11
ARSA	rs58542926	19	19268740	T	C	0.08	0.18	0.01	3.64E-22
ARSA	rs429358	19	44908684	C	T	0.16	-0.06	0.01	7.57E-06
ARSA	rs873697	22	50625741	A	G	0.05	1.49	0.02	0.00E+00
ARSB	rs1065757	5	78885654	T	C	0.44	-0.30	0.01	3.00E-148
ARSB	rs17622656	5	132485305	A	G	0.39	-0.06	0.01	2.26E-06
ARSB	rs1293303	8	11862718	C	G	0.44	0.07	0.01	5.95E-11
ARSB	rs6993770	8	105569300	T	A	0.29	-0.08	0.01	1.08E-11
ARSB	rs10968020	9	27628442	C	T	0.26	-0.06	0.01	1.27E-06
ARSB	rs7896518	10	63344740	G	A	0.42	0.08	0.01	2.02E-13
ARSB	rs202046353	12	8966818	T	A	0.09	0.09	0.01	8.48E-06
ARSB	rs10778152	12	101831973	G	A	0.29	-0.09	0.01	2.10E-12
ARSB	rs145078947	14	93186629	T	G	0.00	0.59	0.07	6.82E-08
ARSB	rs8072896	17	57005471	G	T	0.48	-0.05	0.01	1.05E-05
ARSB	rs892090	19	55027704	G	T	0.83	0.10	0.01	1.32E-11
ART3	rs150816167	1	179602727	C	T	0.04	0.15	0.02	1.91E-08

ART3	rs1868164	3	134650578	A	G	0.63	-0.06	0.01	1.18E-08
ART3	-	4	76109245	C	T	0.43	0.27	0.01	4.94E-143
ART3	rs11649031	16	377516	C	T	0.59	-0.06	0.01	1.51E-07
ART3	rs36060036	16	20350628	T	C	0.17	-0.08	0.01	1.76E-09
ARTN	rs112597211	14	105903575	C	T	0.14	0.09	0.01	1.03E-07
ASAH2	rs12032634	1	25681077	A	T	0.52	-0.08	0.01	1.20E-16
ASAH2	rs3732356	3	119810266	T	G	0.94	-0.11	0.01	7.88E-08
ASAH2	rs531060104	4	99128003	T	C	0.23	-0.06	0.01	7.00E-06
ASAH2	rs890793	5	154194378	T	C	0.38	-0.05	0.01	5.15E-06
ASAH2	rs12206570	6	130063959	G	T	0.58	0.04	0.01	2.01E-05
ASAH2	rs804269	8	11771610	C	T	0.69	-0.05	0.01	8.37E-07
ASAH2	rs12056768	8	115976302	G	T	0.58	0.06	0.01	1.13E-09
ASAH2	rs56278466	10	17833858	G	T	0.66	0.07	0.01	2.62E-09
ASAH2	rs1877558	10	50232820	T	C	0.24	-0.69	0.01	0.00E+00
ASAH2	rs2351958	11	16226474	A	C	0.39	0.05	0.01	9.13E-07
ASAH2	rs35764600	12	11638694	C	G	0.40	-0.08	0.01	1.87E-15
ASAH2	-	12	21191379	G	A	0.24	0.07	0.01	6.20E-10
ASAH2	rs7979473	12	120982457	G	A	0.62	-0.05	0.01	2.74E-07
ASAH2	rs28929474	14	94378610	T	C	0.02	0.16	0.02	7.50E-06
ASAH2	rs139974673	15	43735687	C	T	0.02	0.16	0.02	1.91E-06
ASAH2	rs12440841	15	50713624	T	C	0.34	-0.09	0.01	7.60E-19
ASAH2	rs2611867	17	47587495	G	A	0.51	-0.07	0.01	1.51E-12
ASAH2	rs77542162	17	69085137	G	A	0.02	0.28	0.02	3.22E-17
ASGR1	rs12740374	1	109274968	T	G	0.22	0.13	0.01	6.34E-22
ASGR1	rs11405616	5	142130696	A	C	0.61	0.05	0.01	1.98E-05
ASGR1	rs7012814	8	9315848	A	G	0.47	-0.05	0.01	4.11E-06
ASGR1	rs2622935	11	117140042	G	A	0.81	-0.14	0.01	1.87E-23
ASGR1	rs28929474	14	94378610	T	C	0.02	0.32	0.03	6.01E-16
ASGR1	rs55714927	17	7176997	T	C	0.20	-0.19	0.01	7.24E-43
ASGR1	rs1801689	17	66214462	C	A	0.03	0.20	0.02	5.25E-09
ATF2	rs58534292	14	21737747	C	T	0.65	-0.07	0.01	2.10E-08
ATF2	rs2857339	14	105916828	T	C	0.50	0.14	0.01	1.34E-23
ATG4A	rs1354034	3	56815721	C	T	0.60	0.05	0.01	5.42E-06
ATG4A	rs749679	16	88504408	G	A	0.30	0.06	0.01	8.54E-07
ATG4A	rs8887	19	4502189	C	T	0.57	0.06	0.01	1.03E-07
ATOX1	rs1354034	3	56815721	C	T	0.60	0.06	0.01	1.96E-07
ATOX1	rs17622656	5	132485305	A	G	0.39	-0.05	0.01	1.55E-05
ATOX1	rs371549947	5	151751699	T	C	0.00	-1.47	0.14	6.16E-12
ATOX1	rs139130389	11	72139110	A	C	0.07	-0.12	0.01	2.91E-08
ATOX1	rs58534292	14	21737747	C	T	0.65	-0.11	0.01	3.40E-20
ATOX1	rs73365420	14	106015481	A	C	0.62	-0.12	0.01	8.38E-23
ATOX1	rs200309755	17	29518756	T	C	0.54	0.05	0.01	1.65E-05
ATOX1	rs1613662	19	55025227	A	G	0.83	0.08	0.01	3.97E-07
ATP5IF1	rs510379	1	28208722	C	T	0.36	0.11	0.01	3.52E-20
ATP5IF1	rs1354034	3	56815721	C	T	0.60	0.06	0.01	3.02E-07
ATP5IF1	rs17622656	5	132485305	A	G	0.39	-0.05	0.01	2.38E-05
ATP5IF1	rs342298	7	106733200	T	C	0.45	-0.05	0.01	2.60E-05
ATP5IF1	rs10733789	10	63188924	C	T	0.31	0.06	0.01	6.34E-06
ATP5IF1	rs10849412	12	6176680	G	A	0.43	-0.05	0.01	1.71E-05
ATP6AP2	rs76415507	6	32524812	G	T	0.56	-0.06	0.01	4.92E-06
ATP6V1D	rs189049184	6	32519697	A	G	0.82	-0.09	0.01	3.51E-08
ATP6V1F	rs1354034	3	56815721	C	T	0.60	0.06	0.01	5.75E-08
ATXN10	rs1354034	3	56815721	C	T	0.60	0.08	0.01	8.21E-11
ATXN10	rs342293	7	106731773	G	C	0.46	-0.05	0.01	6.32E-06
ATXN10	rs6993770	8	105569300	T	A	0.29	-0.06	0.01	1.19E-06
ATXN10	rs7080386	10	63288546	A	C	0.41	0.05	0.01	7.56E-06

ATXN10	rs138166	22	45735358	A	G	0.30	0.11	0.01	2.75E-17
AXIN1	rs1354034	3	56815721	C	T	0.60	0.06	0.01	9.65E-07
AXIN1	rs6993770	8	105569300	T	A	0.29	-0.06	0.01	4.58E-07
AXIN1	rs11647223	16	311321	T	C	0.47	0.05	0.01	1.89E-05
AXL	rs79412885	1	9181780	A	G	0.07	-0.16	0.01	4.50E-14
AXL	rs7639097	3	99058485	T	G	0.61	-0.06	0.01	1.20E-06
AXL	rs28393315	6	32635400	G	A	0.26	0.06	0.01	1.60E-05
AXL	rs950802	11	60385111	A	G	0.31	0.05	0.01	1.22E-05
AXL	rs35350651	12	111469627	C	A	0.50	-0.05	0.01	2.68E-06
AXL	rs7140110	13	113841051	C	T	0.30	0.13	0.01	6.52E-25
AXL	rs186021206	17	7166093	A	G	0.01	0.98	0.05	2.56E-38
AXL	rs7224668	17	81261988	C	T	0.56	-0.05	0.01	1.18E-05
AXL	rs66841352	19	41233275	C	G	0.40	-0.17	0.01	9.54E-53
AZU1	rs2210918	1	157723195	C	T	0.47	0.05	0.01	7.84E-06
AZU1	rs2951842	8	6968999	G	C	0.50	-0.06	0.01	1.48E-07
AZU1	rs57993781	8	143547232	A	T	0.48	-0.06	0.01	1.22E-07
AZU1	-	19	831815	G	T	0.01	-1.15	0.04	5.47E-101
B4GALT1	rs61747728	1	179557079	T	C	0.04	0.16	0.02	2.68E-08
B4GALT1	rs17138646	5	116010548	G	T	0.12	0.10	0.01	2.04E-09
B4GALT1	rs617578	6	32606826	A	G	0.10	-0.10	0.01	1.90E-07
B4GALT1	rs7019909	9	33113324	T	C	0.10	0.33	0.01	1.97E-73
B4GALT1	rs550057	9	133271182	T	C	0.26	0.10	0.01	7.99E-14
B4GALT1	rs77924615	16	20381010	A	G	0.20	-0.06	0.01	1.52E-05
B4GALT1	rs7247412	19	51810615	C	T	0.27	-0.32	0.01	3.74E-146
B4GAT1	rs10168551	2	167741476	C	T	0.20	-0.06	0.01	1.26E-05
B4GAT1	rs11130630	3	58375073	G	A	0.37	0.05	0.01	3.25E-06
B4GAT1	rs9274678	6	32668869	G	A	0.15	-0.09	0.01	1.11E-09
B4GAT1	rs10217770	9	33146395	A	C	0.42	-0.08	0.01	4.29E-12
B4GAT1	rs56278466	10	17833858	G	T	0.66	0.10	0.01	4.78E-18
B4GAT1	rs10896113	11	66349640	T	C	0.22	-0.32	0.01	6.30E-131
B4GAT1	rs74611001	11	94487353	A	C	0.02	-0.20	0.02	9.19E-08
B4GAT1	rs28873836	19	51811402	C	G	0.27	-0.20	0.01	3.87E-58
B4GAT1	rs12975366	19	54255498	C	T	0.40	-0.05	0.01	3.95E-06
BACH1	rs342293	7	106731773	G	C	0.46	-0.05	0.01	2.52E-05
BACH1	rs6993770	8	105569300	T	A	0.29	-0.06	0.01	7.09E-07
BACH1	rs4759076	12	54336088	C	T	0.46	0.05	0.01	8.86E-06
BAG3	rs710446	3	186742138	C	T	0.41	-0.07	0.01	1.77E-08
BAG3	rs66530140	4	186240057	C	T	0.51	-0.09	0.01	4.22E-15
BAG3	rs2545801	5	177414338	C	T	0.74	-0.07	0.01	2.32E-07
BAG3	rs35434411	10	119669882	A	G	0.03	0.17	0.02	1.86E-07
BAG3	rs55714647	19	35055403	A	G	0.93	-0.10	0.02	1.53E-05
BAG6	-	10	63156270	A	T	0.49	0.06	0.01	2.07E-06
BAIAP2	rs4835265	4	145900258	A	C	0.16	0.07	0.01	1.47E-05
BAIAP2	rs174560	11	61814292	C	T	0.31	0.06	0.01	1.39E-05
BAIAP2	rs3741513	12	124372114	A	T	0.61	0.08	0.01	1.08E-10
BAIAP2	rs550999044	14	103101069	T	A	0.24	0.08	0.01	1.01E-08
BAIAP2	rs9989483	17	80997736	T	C	0.17	0.10	0.01	5.69E-10
BAIAP2	rs12373325	18	58420416	C	T	0.80	-0.06	0.01	2.41E-05
BAIAP2	rs189448562	20	3724468	C	G	0.01	0.40	0.04	6.74E-13
BAMBI	rs1354034	3	56815721	C	T	0.60	-0.07	0.01	2.54E-08
BANK1	rs1354034	3	56815721	C	T	0.60	0.07	0.01	2.93E-10
BANK1	rs3113676	4	102043886	C	T	0.98	0.48	0.03	3.20E-25
BANK1	rs342293	7	106731773	G	C	0.46	-0.06	0.01	8.95E-07
BAX	rs1354034	3	56815721	C	T	0.60	0.06	0.01	2.75E-07
BAX	rs3847326	10	63572099	G	A	0.48	-0.05	0.01	1.66E-05
BCAM	rs539657009	1	161658666	T	C	0.13	0.08	0.01	5.02E-06

BCAM	rs1260326	2	27508073	C	T	0.61	0.05	0.01	7.11E-06
BCAM	rs10514710	3	44842479	A	G	0.20	-0.06	0.01	1.81E-05
BCAM	rs139153665	3	58376860	C	T	0.37	0.07	0.01	1.98E-09
BCAM	-	4	144140928	A	G	0.45	0.12	0.01	4.65E-29
BCAM	rs73080552	5	39435331	A	G	0.11	-0.08	0.01	1.22E-06
BCAM	rs12522289	5	178213863	G	A	0.08	-0.09	0.01	1.08E-05
BCAM	rs9271325	6	32614736	G	C	0.47	-0.06	0.01	3.41E-09
BCAM	rs778641135	6	135097494	T	C	0.26	-0.06	0.01	1.06E-06
BCAM	rs6592965	7	50360284	A	G	0.45	-0.08	0.01	2.81E-14
BCAM	rs8176672	9	133266772	T	C	0.06	0.66	0.01	3.02E-186
BCAM	rs56278466	10	17833858	G	T	0.66	0.16	0.01	1.23E-46
BCAM	-	12	54339944	T	C	0.10	0.10	0.01	1.50E-07
BCAM	rs2137537	12	70719307	C	T	0.55	-0.05	0.01	2.16E-05
BCAM	rs200489612	17	7203059	A	G	0.01	0.41	0.05	3.77E-07
BCAM	rs1799918	19	12891586	C	G	0.38	0.11	0.01	4.41E-24
BCAM	rs28399654	19	44813331	A	G	0.03	-0.65	0.02	2.34E-104
BCAM	rs2638282	19	48710576	A	G	0.53	0.05	0.01	1.03E-05
BCAM	rs2410405	21	41208436	T	C	0.38	0.11	0.01	3.03E-23
BCAN	rs1877465	1	117301629	G	A	0.19	-0.06	0.01	1.82E-05
BCAN	rs2365715	1	156645322	G	A	0.39	-0.23	0.01	1.16E-87
BCAN	rs13086758	3	24380618	A	T	0.21	-0.07	0.01	1.15E-06
BCAN	rs2071592	6	31547563	A	T	0.35	-0.06	0.01	4.41E-07
BCAN	rs73086586	7	20355325	T	C	0.06	0.16	0.01	8.07E-12
BCAN	rs66530708	7	73611279	A	G	0.07	-0.13	0.01	1.21E-09
BCAN	rs56278466	10	17833858	G	T	0.66	0.12	0.01	2.88E-27
BCL2L11	rs13412535	2	224010157	A	G	0.23	-0.06	0.01	4.11E-06
BCL2L11	rs1354034	3	56815721	C	T	0.60	-0.05	0.01	3.05E-06
BCL2L11	rs5030062	3	186736391	C	A	0.37	-0.15	0.01	1.38E-40
BCL2L11	rs66530140	4	186240057	C	T	0.51	-0.29	0.01	5.37E-146
BCL2L11	rs1801020	5	177409531	G	A	0.74	-0.15	0.01	9.35E-33
BCL2L11	rs6961069	7	80589645	T	C	0.40	0.05	0.01	1.86E-05
BCL2L11	rs6993770	8	105569300	T	A	0.29	-0.10	0.01	4.44E-17
BCL2L11	rs10761737	10	63292445	C	T	0.41	0.08	0.01	3.48E-13
BCL2L11	rs71354995	19	38301201	G	A	0.26	-0.21	0.01	5.91E-64
BCL2L11	rs892090	19	55027704	G	T	0.83	0.11	0.01	2.34E-12
BCR	rs149005797	22	23142913	C	T	0.00	-0.54	0.06	1.10E-07
BGN	rs744233	3	126530422	A	G	0.29	0.06	0.01	5.67E-07
BGN	rs3756074	4	73982348	C	G	0.05	0.15	0.02	1.32E-08
BGN	rs4463068	4	101880317	T	A	0.40	0.06	0.01	4.35E-07
BGN	rs9266095	6	31353550	G	A	0.21	0.09	0.01	5.51E-10
BGN	rs6993770	8	105569300	T	A	0.29	0.09	0.01	1.74E-13
BGN	rs7080386	10	63288546	A	C	0.41	-0.07	0.01	3.35E-09
BGN	rs2511241	11	73234296	T	C	0.93	-0.23	0.01	8.01E-25
BGN	rs77242536	14	87979796	C	T	0.14	0.07	0.01	1.08E-05
BGN	rs780989226	15	101208405	T	A	0.11	0.12	0.01	5.00E-11
BGN	rs61758388	16	17470454	A	C	0.03	-0.36	0.02	6.95E-29
BGN	rs1654425	19	55027612	C	T	0.83	-0.12	0.01	9.28E-14
BGN	rs4814837	20	19261036	T	C	0.34	-0.06	0.01	7.85E-06
BID	rs142201367	4	186235350	C	T	0.50	0.08	0.01	1.03E-11
BID	rs10418046	19	53824615	G	T	0.21	0.10	0.01	2.70E-12
BID	rs183393610	22	17769676	A	G	0.01	-0.40	0.05	5.09E-08
BIN2	rs1354034	3	56815721	C	T	0.60	0.06	0.01	4.37E-08
BIN2	rs112838960	5	132373340	A	T	0.52	0.05	0.01	4.85E-06
BIN2	rs6993770	8	105569300	T	A	0.29	-0.06	0.01	1.24E-06
BIN2	rs149960700	12	51311384	A	C	0.11	-0.18	0.01	3.31E-23
BIRC2	rs1354034	3	56815721	C	T	0.60	0.05	0.01	6.94E-06

BIRC2	rs342298	7	106733200	T	C	0.46	-0.05	0.01	2.64E-05
BIRC2	rs10761785	10	63559006	T	G	0.51	0.05	0.01	1.07E-05
BIRC2	rs61754131	11	102377675	G	A	0.00	-0.68	0.08	2.34E-07
BLMH	rs1250258	2	215435462	T	C	0.74	0.06	0.01	4.63E-07
BLMH	-	3	52505864	C	T	0.01	0.92	0.04	9.05E-42
BLMH	rs1265889	6	32065839	A	G	0.17	-0.07	0.01	2.54E-07
BLMH	rs1050565	17	30249058	C	T	0.33	0.57	0.01	0.00E+00
BLVRB	rs7776054	6	135097778	G	A	0.26	-0.08	0.01	2.93E-09
BLVRB	rs149698066	19	40458157	A	G	0.01	-1.45	0.05	3.36E-75
BMP4	rs2314145	1	19410349	G	C	0.23	-0.06	0.01	2.34E-05
BMP4	rs535025308	11	74721143	C	G	0.04	-0.35	0.02	8.25E-34
BMP4	rs35887873	11	126349501	C	T	0.23	0.07	0.01	4.73E-07
BMP4	rs11111765	12	103778741	C	G	0.26	0.06	0.01	3.71E-06
BMP4	rs186021206	17	7166093	A	G	0.01	0.76	0.05	3.01E-22
BMP4	rs704	17	28367840	A	G	0.47	-0.06	0.01	2.14E-06
BMP6	rs68066031	2	224015781	C	T	0.23	-0.07	0.01	2.96E-06
BMP6	rs1354034	3	56815721	C	T	0.60	-0.21	0.01	8.80E-73
BMP6	rs3792386	3	123134833	A	G	0.59	-0.05	0.01	1.32E-05
BMP6	rs5030062	3	186736391	C	A	0.37	-0.09	0.01	9.53E-13
BMP6	-	4	186236113	C	A	0.51	-0.15	0.01	7.28E-38
BMP6	rs2545801	5	177414338	C	T	0.74	-0.10	0.01	2.52E-13
BMP6	rs2327009	6	7718027	T	C	0.48	0.13	0.01	3.55E-32
BMP6	rs6993770	8	105569300	T	A	0.29	-0.08	0.01	9.42E-10
BMP6	rs10820606	9	96430637	C	A	0.23	-0.11	0.01	3.17E-15
BMP6	rs2519093	9	133266456	T	C	0.18	-0.09	0.01	2.41E-09
BMP6	rs12762934	10	102600127	T	C	0.32	0.10	0.01	4.32E-17
BMP6	rs535025308	11	74721143	C	G	0.04	-0.18	0.02	2.47E-10
BMP6	rs996498	12	29365497	G	A	0.24	0.06	0.01	1.92E-05
BMP6	rs6580981	12	54329244	A	G	0.46	0.07	0.01	2.79E-10
BMP6	rs186021206	17	7166093	A	G	0.01	0.45	0.05	2.02E-08
BMP6	-	17	35682334	C	T	0.13	0.08	0.01	5.28E-06
BMP6	rs892090	19	55027704	G	T	0.83	0.10	0.01	2.37E-10
BMP6	rs2834720	21	35004542	A	G	0.27	0.06	0.01	2.15E-06
BOC	rs1260326	2	27508073	C	T	0.61	0.06	0.01	6.43E-08
BOC	rs3856718	3	113277371	A	G	0.17	0.20	0.01	1.05E-41
BOC	rs34437827	7	73509008	G	A	0.19	0.07	0.01	3.98E-06
BOC	rs56408342	8	22190977	A	G	0.05	-0.15	0.02	5.28E-09
BOC	rs8176749	9	133255801	T	C	0.06	0.13	0.02	4.33E-08
BOC	rs145878042	12	47749532	G	A	0.01	0.24	0.04	2.65E-05
BOC	rs1037117	15	101528455	A	G	0.25	-0.07	0.01	1.16E-06
BOC	rs186021206	17	7166093	A	G	0.01	0.54	0.05	1.07E-11
BPIFB1	rs1619599	3	12462791	A	G	0.58	0.08	0.01	6.75E-16
BPIFB1	rs7429705	3	72242073	T	C	0.70	0.06	0.01	1.78E-08
BPIFB1	rs791361	5	173781630	C	A	0.77	0.07	0.01	3.39E-08
BPIFB1	rs34978289	6	2504388	T	C	0.52	0.08	0.01	3.50E-15
BPIFB1	rs9389269	6	135106021	C	T	0.27	0.10	0.01	9.69E-18
BPIFB1	rs17489268	8	19994534	A	T	0.26	0.08	0.01	2.14E-11
BPIFB1	rs2721961	8	115645684	G	T	0.28	-0.06	0.01	1.27E-06
BPIFB1	rs2980888	8	125495066	C	T	0.70	0.05	0.01	2.07E-06
BPIFB1	rs4878009	9	86719896	A	G	0.41	-0.05	0.01	2.66E-07
BPIFB1	rs569255485	11	47549820	T	G	0.34	-0.05	0.01	2.93E-06
BPIFB1	-	11	116752497	T	A	0.86	0.07	0.01	2.67E-06
BPIFB1	rs147233090	15	43735849	T	C	0.02	-0.15	0.02	1.48E-05
BPIFB1	rs247617	16	56956804	A	C	0.32	0.11	0.01	8.65E-24
BPIFB1	rs12597477	16	78300974	G	A	0.20	-0.07	0.01	7.19E-07
BPIFB1	rs116843064	19	8364439	A	G	0.02	0.20	0.02	1.36E-07

BPIFB1	rs681343	19	48703205	T	C	0.51	0.08	0.01	4.48E-16
BPIFB1	rs2424963	20	33106642	C	T	0.58	-0.42	0.01	2.61E-307
BRK1	rs1354034	3	56815721	C	T	0.60	0.13	0.01	2.75E-31
BRK1	rs6054	4	154568456	T	C	0.01	0.41	0.05	1.21E-07
BRK1	rs342293	7	106731773	G	C	0.46	-0.07	0.01	1.13E-10
BRK1	rs7896518	10	63344740	G	A	0.42	0.07	0.01	9.82E-10
BRK1	rs11605246	11	198062	G	C	0.23	0.09	0.01	9.94E-12
BRK1	rs707236	16	83769555	A	T	0.35	-0.15	0.01	1.63E-36
BRK1	rs9897552	17	35647462	A	G	0.18	0.07	0.01	5.60E-06
BRK1	rs1065853	19	44909976	T	G	0.08	-0.25	0.01	1.54E-32
BSG	rs61747728	1	179557079	T	C	0.04	0.18	0.02	2.90E-10
BSG	rs78444298	1	184702964	A	G	0.02	0.21	0.03	3.21E-07
BSG	rs1260326	2	27508073	C	T	0.61	0.06	0.01	3.45E-07
BSG	rs700753	7	46714086	G	C	0.65	0.06	0.01	3.00E-07
BSG	rs9987289	8	9325848	G	A	0.91	-0.08	0.01	2.15E-05
BSG	rs2519093	9	133266456	T	C	0.18	-0.15	0.01	1.32E-24
BSG	rs77924615	16	20381010	A	G	0.20	-0.07	0.01	3.09E-07
BSG	rs200489612	17	7203059	A	G	0.01	0.43	0.05	1.70E-07
BSG	rs56101188	19	586745	C	T	0.09	0.24	0.01	9.03E-36
BSG	rs933224	22	36369958	C	T	0.68	-0.05	0.01	1.69E-05
BST1	rs2302465	4	15707569	A	G	0.13	-1.48	0.01	0.00E+00
BST1	rs199922514	8	9325592	G	A	0.91	-0.06	0.01	1.31E-06
BST1	rs7952602	11	126363774	C	G	0.14	-0.11	0.01	5.75E-24
BST1	rs186021206	17	7166093	A	G	0.01	0.54	0.03	5.05E-27
BST1	rs4760	19	43648948	G	A	0.16	-0.21	0.01	1.69E-98
BST2	rs57683598	1	1005954	A	G	0.40	0.05	0.01	2.57E-05
BST2	rs2269615	1	172446311	T	C	0.56	-0.05	0.01	3.44E-06
BST2	rs2111485	2	162254026	G	A	0.61	0.06	0.01	2.04E-07
BST2	rs4835265	4	145900258	A	C	0.16	0.07	0.01	3.50E-06
BST2	rs6926219	6	122399661	A	G	0.55	-0.06	0.01	6.46E-07
BST2	rs505922	9	133273813	C	T	0.32	0.10	0.01	1.95E-18
BST2	rs17157266	11	62432345	C	T	0.18	-0.07	0.01	4.31E-06
BST2	rs139269536	14	103105358	G	C	0.25	0.09	0.01	5.55E-11
BST2	rs12373325	18	58420416	C	T	0.80	-0.06	0.01	5.12E-06
BST2	rs35251378	19	10349293	A	G	0.29	-0.08	0.01	1.35E-11
BST2	rs8101243	19	17377984	C	T	0.96	-0.34	0.02	3.78E-36
BST2	rs5992933	22	17974277	T	G	0.24	-0.06	0.01	4.88E-06
BTC	rs1354034	3	56815721	C	T	0.60	0.05	0.01	6.96E-06
BTC	rs28549760	4	74794307	C	A	0.13	-0.60	0.01	2.10E-272
BTC	rs342296	7	106732457	A	G	0.46	-0.05	0.01	1.10E-05
BTC	rs7044478	9	97858720	G	C	0.33	-0.06	0.01	5.62E-06
BTC	rs150230220	10	63142032	T	A	0.31	0.05	0.01	1.30E-05
BTC	rs60822569	12	54323724	C	T	0.55	0.06	0.01	8.72E-08
BTN2A1	rs61747728	1	179557079	T	C	0.04	0.19	0.02	2.35E-11
BTN2A1	rs143048713	3	46851683	T	G	0.10	0.08	0.01	1.51E-05
BTN2A1	rs72843784	6	26498530	T	G	0.11	0.47	0.01	2.89E-156
BTN2A1	rs1151851	12	120902336	T	A	0.41	0.05	0.01	6.16E-06
BTN2A1	rs77924615	16	20381010	A	G	0.20	-0.08	0.01	3.73E-08
BTN2A1	rs186021206	17	7166093	A	G	0.01	0.52	0.05	4.34E-12
BTN2A1	rs2659005	17	81244914	T	C	0.44	0.06	0.01	1.86E-08
BTN3A2	rs9393710	6	26367605	G	A	0.13	-0.95	0.01	0.00E+00
BTN3A2	rs3184504	12	111446804	C	T	0.52	-0.08	0.01	2.95E-15
BTN3A2	rs74439742	16	57025515	T	C	0.17	-0.10	0.01	3.89E-13
C19orf12	rs142552223	3	172516227	A	G	0.04	-0.14	0.02	8.72E-06
C19orf12	rs705379	7	95324583	A	G	0.48	0.09	0.01	6.01E-14
C19orf12	rs16919942	11	94107249	T	C	0.11	-0.09	0.01	4.96E-06

C19orf12	rs28929474	14	94378610	T	C	0.02	0.20	0.03	1.50E-06
C19orf12	rs146170087	19	29702747	C	T	0.00	-0.98	0.12	1.49E-07
C19orf12	rs738409	22	43928847	G	C	0.22	0.10	0.01	2.96E-12
C1QA	rs12058824	1	22636557	A	G	0.08	-0.63	0.01	5.57E-217
C1QA	rs188468174	1	24965206	T	C	0.01	0.21	0.03	2.27E-05
C1QA	rs2765501	1	157834858	A	G	0.40	0.07	0.01	4.01E-09
C1QA	rs6532770	4	98862518	A	C	0.24	0.15	0.01	1.36E-32
C1QA	rs546388	5	172901130	T	C	0.45	-0.07	0.01	6.80E-10
C1QA	rs2734975	6	29865476	A	C	0.46	-0.06	0.01	1.15E-08
C1QA	rs1727	10	89307012	A	C	0.72	-0.11	0.01	7.03E-20
C1QA	rs573898146	11	122644975	T	C	0.37	-0.05	0.01	2.76E-05
C1QA	rs4766578	12	111466567	A	T	0.50	-0.06	0.01	4.66E-08
C1QA	rs7139746	13	49210231	A	G	0.68	0.05	0.01	9.71E-06
C1QA	rs2665399	17	59789061	C	A	0.84	-0.09	0.01	1.44E-09
C1QA	rs117981338	17	73092704	T	C	0.01	0.40	0.03	7.06E-18
C1QA	rs55841020	18	48294801	A	T	0.17	-0.08	0.01	1.43E-07
C1QA	rs28659908	19	35202217	C	T	0.40	0.06	0.01	9.51E-09
C1QTNF1	rs2498323	4	3449382	A	G	0.10	0.13	0.01	1.36E-12
C1QTNF1	rs12359178	10	17847614	A	G	0.14	0.10	0.01	1.38E-10
C1QTNF1	rs116539064	17	79022318	T	C	0.10	0.39	0.01	6.68E-92
C1QTNF1	rs2838952	21	45510249	T	C	0.03	-0.24	0.02	5.45E-15
C2	rs1260326	2	27508073	C	T	0.61	-0.07	0.01	2.02E-09
C2	-	6	31934288	A	C	0.01	-2.27	0.04	9.78E-261
C2	rs56278466	10	17833858	G	T	0.66	0.11	0.01	1.99E-20
C2	-	12	7074470	T	A	0.14	-0.09	0.01	2.05E-08
C2CD2L	rs142201367	4	186235350	C	T	0.49	0.05	0.01	8.67E-06
C2CD2L	rs640603	11	119093620	A	G	0.35	-0.06	0.01	7.59E-06
C4BPB	rs140584594	1	109690361	G	A	0.73	0.06	0.01	2.05E-06
C4BPB	-	1	207112189	C	T	0.07	-0.31	0.01	2.45E-46
C4BPB	rs805435	2	53837646	C	A	0.19	0.06	0.01	1.47E-05
C4BPB	rs2316653	3	90393775	G	A	0.50	-0.06	0.01	1.17E-07
C4BPB	rs9822589	3	94212297	A	G	0.47	0.06	0.01	1.01E-07
C4BPB	rs687339	3	136213517	T	C	0.77	-0.06	0.01	3.70E-06
C4BPB	rs28507491	4	76276498	A	G	0.38	-0.05	0.01	5.20E-06
C4BPB	rs757795770	4	99065369	C	A	0.71	0.07	0.01	1.41E-08
C4BPB	rs601020	6	32605402	A	G	0.24	-0.06	0.01	1.02E-06
C4BPB	rs4418728	10	93079967	T	G	0.45	-0.05	0.01	1.28E-05
C4BPB	rs139130389	11	72139110	A	C	0.07	-0.16	0.01	1.63E-14
C4BPB	rs11621792	14	24402720	T	C	0.45	-0.05	0.01	8.62E-06
C4BPB	rs28929474	14	94378610	T	C	0.02	0.37	0.02	2.41E-22
C4BPB	rs9302635	16	72110275	C	T	0.18	-0.14	0.01	1.45E-22
C4BPB	rs8178824	17	66228657	T	C	0.03	0.81	0.02	6.81E-145
CA1	rs3811444	1	247876149	T	C	0.33	0.08	0.01	4.10E-11
CA1	rs11759553	6	135101158	T	A	0.27	-0.07	0.01	1.01E-08
CA1	rs12544332	8	85343981	A	C	0.59	-0.09	0.01	1.06E-14
CA1	rs67544533	15	75069198	G	A	0.18	-0.13	0.01	7.34E-20
CA11	rs646776	1	109275908	T	C	0.78	0.19	0.01	4.02E-41
CA11	rs727415	10	71822995	A	T	0.29	-0.06	0.01	4.15E-06
CA11	-	19	48612661	C	A	0.20	0.08	0.01	4.79E-08
CA12	rs61747728	1	179557079	T	C	0.04	0.18	0.02	1.72E-09
CA12	rs1260326	2	27508073	C	T	0.61	0.07	0.01	3.25E-10
CA12	rs868399694	3	98885415	T	A	0.45	0.06	0.01	8.08E-07
CA12	rs13107325	4	102267552	T	C	0.08	0.13	0.01	6.62E-10
CA12	rs567141188	9	125628866	A	T	0.33	0.06	0.01	8.35E-07
CA12	rs12902855	15	53683717	A	G	0.48	0.06	0.01	2.31E-07
CA12	rs12916532	15	63381972	C	T	0.27	-0.29	0.01	1.96E-119

CA12	rs186021206	17	7166093	A	G	0.01	0.64	0.05	1.16E-16
CA12	rs796389476	17	49195807	T	A	0.10	0.13	0.01	1.77E-11
CA12	rs708686	19	5840608	T	C	0.27	0.11	0.01	2.07E-18
CA13	rs1354034	3	56815721	C	T	0.60	0.05	0.01	5.56E-06
CA13	rs56072918	8	85297179	T	C	0.07	-0.52	0.01	6.05E-122
CA13	rs3847326	10	63572099	G	A	0.48	-0.05	0.01	2.12E-05
CA14	rs1122967	1	150286426	G	C	0.51	0.17	0.01	1.28E-58
CA14	rs61431557	6	34749801	T	C	0.14	-0.07	0.01	1.53E-05
CA14	rs2954021	8	125469835	G	A	0.50	0.07	0.01	1.47E-11
CA14	rs35746821	12	121922683	A	C	0.53	0.06	0.01	5.94E-08
CA14	rs8096658	18	79396537	G	C	0.49	0.05	0.01	1.08E-05
CA2	rs2980370	8	85847898	G	A	0.52	0.10	0.01	4.80E-20
CA2	rs66489920	8	105580311	C	T	0.29	-0.05	0.01	1.56E-05
CA3	rs3811444	1	247876149	T	C	0.33	0.11	0.01	4.47E-22
CA3	rs1047891	2	210675783	A	C	0.31	0.09	0.01	1.99E-13
CA3	rs11759553	6	135101158	T	A	0.27	-0.11	0.01	5.80E-21
CA3	rs2072696	8	85438822	C	G	0.24	-0.18	0.01	3.27E-44
CA3	rs1867146	15	75062630	G	C	0.18	-0.09	0.01	2.36E-10
CA3	rs11085824	19	12890733	G	A	0.38	-0.06	0.01	5.25E-08
CA4	rs184775562	2	196704388	C	T	0.00	0.75	0.09	1.70E-07
CA4	rs78058190	2	218835276	A	G	0.05	-0.19	0.02	7.48E-11
CA4	-	4	26048828	A	C	0.16	0.06	0.01	2.60E-05
CA4	rs115007843	4	82654059	C	A	0.18	-0.07	0.01	2.23E-06
CA4	rs9270588	6	32593550	T	C	0.53	0.05	0.01	1.25E-06
CA4	rs582118	9	133270061	G	A	0.32	0.16	0.01	7.30E-43
CA4	rs10906857	10	15197499	G	A	0.41	0.05	0.01	8.89E-06
CA4	rs61729512	12	7485173	A	G	0.12	0.08	0.01	1.63E-06
CA4	rs117154637	17	60156793	T	A	0.04	-0.82	0.02	7.02E-161
CA4	rs10642898	17	78397594	G	C	0.40	0.06	0.01	1.88E-06
CA4	rs4760	19	43648948	G	A	0.16	-0.17	0.01	3.09E-28
CA4	rs12986064	19	54251270	C	T	0.51	-0.07	0.01	1.33E-09
CA5A	rs4835265	4	145900258	A	C	0.16	0.07	0.01	2.50E-07
CA5A	rs4979373	9	114382224	T	C	0.49	-0.05	0.01	1.18E-06
CA5A	rs10883451	10	100164661	C	T	0.50	-0.05	0.01	5.99E-06
CA5A	rs55870502	16	87893616	C	T	0.19	0.81	0.01	0.00E+00
CA5A	rs738409	22	43928847	G	C	0.22	0.05	0.01	2.58E-05
CA6	rs3765963	1	8974539	G	A	0.41	0.68	0.01	0.00E+00
CA6	rs946527	1	46020298	C	T	0.71	-0.06	0.01	2.29E-06
CA6	rs351388	1	212249238	G	C	0.59	-0.05	0.01	1.05E-05
CA6	rs891499	2	9974102	C	G	0.33	0.07	0.01	5.77E-11
CA6	rs557296878	2	60288320	C	T	0.59	0.07	0.01	2.61E-12
CA6	rs6752452	2	225937638	G	T	0.15	-0.07	0.01	6.56E-07
CA6	rs836143	11	34514038	C	G	0.67	-0.05	0.01	9.31E-08
CA6	rs4256938	11	94590411	A	G	0.09	-0.12	0.01	1.91E-14
CA6	rs290371	15	95217879	A	G	0.36	-0.07	0.01	5.99E-13
CA6	rs56156922	16	56953457	C	T	0.33	0.06	0.01	1.81E-08
CA6	rs1523135	17	49105958	G	C	0.72	0.05	0.01	8.95E-07
CA6	rs7250028	19	13842306	G	T	0.24	0.06	0.01	1.07E-07
CA6	rs601338	19	48703417	A	G	0.51	0.07	0.01	4.91E-12
CA9	rs9270160	6	32587777	T	C	0.16	-0.08	0.01	9.63E-06
CA9	rs111709193	7	174999	C	T	0.19	0.08	0.01	1.76E-06
CA9	rs183985235	9	35677783	A	C	0.00	-1.49	0.08	1.32E-30
CA9	rs56278466	10	17833858	G	T	0.66	0.14	0.01	3.83E-33
CA9	-	11	61197091	C	A	0.34	0.06	0.01	5.06E-06
CA9	rs616114	18	32217958	T	C	0.39	0.08	0.01	1.81E-12
CA9	rs2608894	19	5847989	C	T	0.76	-0.07	0.01	1.72E-06

CA9	rs516246	19	48702915	T	C	0.51	-0.05	0.01	2.02E-05
CALB1	rs143241372	8	90111355	G	A	0.01	0.65	0.04	1.74E-26
CALB1	rs9529913	13	71770957	T	C	0.60	-0.09	0.01	1.46E-13
CALB1	rs6127099	20	54114863	T	A	0.28	-0.06	0.01	1.22E-06
CALB2	-	16	71136766	C	A	0.28	-0.07	0.01	2.08E-08
CALCA	rs12619508	2	113224463	A	C	0.47	0.07	0.01	2.54E-12
CALCA	rs73995235	2	232331827	T	G	0.06	-0.11	0.01	2.99E-08
CALCA	rs7008914	8	26022884	C	T	0.26	-0.05	0.01	4.25E-06
CALCA	rs10766197	11	14900334	A	G	0.45	-0.10	0.01	1.92E-28
CALCA	rs10841569	12	20581905	G	A	0.15	0.09	0.01	8.92E-11
CALCA	rs138903858	14	105168150	T	G	0.03	-0.14	0.02	2.57E-06
CALCA	rs3790163	20	10667303	G	A	0.79	0.07	0.01	1.88E-08
CALCOCO1	rs1354034	3	56815721	C	T	0.60	0.07	0.01	1.10E-08
CALCOCO1	rs342293	7	106731773	G	C	0.46	-0.06	0.01	9.22E-07
CALCOCO1	rs6993770	8	105569300	T	A	0.29	-0.07	0.01	1.11E-08
CALCOCO1	rs17102261	12	53732948	G	T	0.15	0.14	0.01	1.09E-18
CAMKK1	rs342296	7	106732457	A	G	0.46	-0.06	0.01	3.17E-07
CAMKK1	rs4759076	12	54336088	C	T	0.46	0.08	0.01	1.30E-10
CAMKK1	rs753513	17	3890178	T	C	0.29	-0.30	0.01	5.56E-125
CANT1	rs61747728	1	179557079	T	C	0.04	0.15	0.02	9.43E-07
CANT1	rs1863622	3	186678827	C	T	0.22	-0.08	0.01	2.60E-08
CANT1	-	8	105561296	C	A	0.27	-0.07	0.01	5.14E-07
CANT1	rs10813956	9	33145545	C	T	0.49	-0.05	0.01	7.24E-06
CANT1	rs56278466	10	17833858	G	T	0.66	0.09	0.01	1.28E-13
CANT1	rs423135	16	89674465	A	G	0.52	0.06	0.01	7.65E-07
CANT1	rs17739056	17	78999123	C	T	0.12	-0.21	0.01	1.45E-32
CANT1	rs1654425	19	55027612	C	T	0.83	0.11	0.01	7.96E-12
CAPG	rs6886	2	85394936	C	T	0.67	-0.87	0.01	0.00E+00
CAPG	rs9270270	6	32589431	C	T	0.84	-0.08	0.01	7.35E-10
CAPG	rs4632248	19	53821741	T	G	0.21	0.18	0.01	5.45E-67
CARHSP1	rs12597479	16	8809732	T	G	0.53	-0.08	0.01	4.12E-12
CASP1	rs142032527	7	150521013	T	A	0.24	-0.06	0.01	3.55E-06
CASP1	rs34436714	19	53824059	A	C	0.21	0.32	0.01	4.10E-116
CASP10	rs7576306	2	201209805	G	A	0.49	0.18	0.01	7.32E-58
CASP10	rs1354034	3	56815721	C	T	0.60	0.05	0.01	2.24E-05
CASP10	rs6993770	8	105569300	T	A	0.29	-0.09	0.01	4.86E-13
CASP2	rs1354034	3	56815721	C	T	0.60	0.10	0.01	4.32E-17
CASP2	rs139141690	7	101856650	A	G	0.00	0.38	0.05	9.09E-06
CASP2	rs11502185	11	180258	C	T	0.26	0.07	0.01	7.47E-06
CASP3	rs13136635	4	184653653	T	C	0.88	0.17	0.01	3.85E-22
CASP3	rs6993770	8	105569300	T	A	0.29	-0.08	0.01	1.26E-09
CASP8	rs34841024	2	201286417	G	T	0.14	0.31	0.01	5.78E-82
CASP8	rs1354034	3	56815721	C	T	0.60	0.07	0.01	3.23E-09
CASP8	rs3847326	10	63572099	G	A	0.48	-0.05	0.01	2.67E-06
CASP8	rs60822569	12	54323724	C	T	0.55	0.06	0.01	2.30E-08
CBLIF	rs333947	1	109928142	A	G	0.15	-0.07	0.01	1.78E-06
CBLIF	rs2348194	3	12534091	T	A	0.38	0.05	0.01	1.25E-05
CBLIF	rs13088531	3	151259575	A	T	0.25	-0.06	0.01	1.82E-06
CBLIF	rs1290790	3	169373781	T	A	0.45	-0.06	0.01	3.10E-08
CBLIF	rs72704793	5	547893	A	G	0.33	0.06	0.01	2.80E-08
CBLIF	rs7718236	5	180827869	A	G	0.83	-0.09	0.01	2.71E-09
CBLIF	rs1634726	6	31018051	A	G	0.15	-0.11	0.01	6.41E-12
CBLIF	rs807309	6	119644095	T	C	0.74	-0.46	0.01	1.31E-293
CBLIF	rs34635647	8	142675472	G	A	0.44	-0.05	0.01	2.70E-06
CBLIF	rs2519093	9	133266456	T	C	0.18	-0.21	0.01	3.03E-52
CBLIF	rs56278466	10	17833858	G	T	0.66	0.07	0.01	9.18E-10

CBLIF	rs2472632	11	27374328	A	C	0.36	-0.06	0.01	1.49E-07
CBLIF	rs1787909	11	61202571	C	G	0.34	-0.22	0.01	2.93E-85
CBLIF	rs7929203	11	69157271	T	C	0.28	0.06	0.01	4.33E-06
CBLIF	rs117908897	17	17681832	C	T	0.54	-0.06	0.01	1.96E-07
CBLIF	rs61580404	17	76641961	T	G	0.10	0.08	0.01	1.18E-05
CBLIF	rs584768	19	48710027	A	G	0.53	0.05	0.01	2.98E-06
CBLN4	rs7684253	4	56861145	T	C	0.55	-0.10	0.01	8.41E-18
CBLN4	rs35887873	11	126349501	C	T	0.23	0.08	0.01	9.74E-10
CBLN4	rs215226	12	482134	G	A	0.40	0.06	0.01	7.40E-07
CBLN4	rs186021206	17	7166093	A	G	0.01	0.48	0.05	3.58E-10
CBLN4	rs12624596	20	55714960	A	G	0.29	0.19	0.01	5.20E-52
CC2D1A	rs1354034	3	56815721	C	T	0.60	0.05	0.01	1.93E-05
CC2D1A	rs61740117	19	13913456	T	C	0.01	-0.32	0.03	4.89E-11
CCDC80	rs6772337	3	112643341	G	A	0.62	-0.06	0.01	7.30E-09
CCDC80	rs13107325	4	102267552	T	C	0.08	0.14	0.01	6.00E-12
CCDC80	rs41265930	6	160543103	C	T	0.07	-0.14	0.01	7.22E-13
CCDC80	-	7	7216532	C	T	0.61	0.06	0.01	4.39E-09
CCDC80	rs2142306	8	133458388	C	T	0.41	0.13	0.01	1.47E-34
CCDC80	rs186021206	17	7166093	A	G	0.01	0.93	0.05	5.00E-39
CCL11	rs12075	1	159205564	A	G	0.58	0.08	0.01	6.18E-13
CCL11	rs2228467	3	42864624	C	T	0.06	0.41	0.01	4.31E-75
CCL11	rs3181077	3	46209161	T	C	0.71	-0.08	0.01	2.58E-11
CCL11	rs1354034	3	56815721	C	T	0.60	-0.05	0.01	1.57E-06
CCL11	rs2516471	6	31432515	A	G	0.06	-0.11	0.01	4.83E-07
CCL11	rs10808297	7	75833981	G	A	0.90	-0.21	0.01	1.15E-33
CCL11	rs4602861	8	105578478	G	A	0.27	-0.06	0.01	2.85E-06
CCL11	rs7080386	10	63288546	A	C	0.41	0.07	0.01	2.16E-09
CCL11	rs5818270	16	79995164	T	C	0.58	-0.07	0.01	1.40E-09
CCL11	rs140905292	17	34188515	A	G	0.22	-0.12	0.01	7.25E-20
CCL11	rs1613662	19	55025227	A	G	0.83	0.09	0.01	1.54E-10
CCL11	rs55951234	22	38205423	T	C	0.58	0.05	0.01	8.28E-06
CCL13	rs12075	1	159205564	A	G	0.58	0.22	0.01	6.54E-92
CCL13	rs13412535	2	224010157	A	G	0.23	-0.07	0.01	8.33E-08
CCL13	rs7433284	3	42831098	A	G	0.38	-0.11	0.01	2.58E-21
CCL13	rs12495098	3	46301011	G	T	0.07	0.11	0.01	5.43E-07
CCL13	rs2522051	5	132461886	C	T	0.45	0.05	0.01	3.17E-06
CCL13	rs3135369	6	32419444	A	G	0.23	0.06	0.01	8.55E-07
CCL13	rs2842059	6	70635151	A	C	0.30	-0.06	0.01	4.81E-06
CCL13	rs826732	8	58830080	C	G	0.50	0.05	0.01	8.67E-06
CCL13	rs2737245	8	115646356	T	G	0.28	0.10	0.01	1.65E-16
CCL13	rs7080386	10	63288546	A	C	0.41	0.07	0.01	2.84E-11
CCL13	rs11064074	12	6171873	T	C	0.46	-0.05	0.01	1.15E-05
CCL13	rs59001897	15	64868193	A	T	0.17	0.07	0.01	6.16E-07
CCL13	rs12445050	16	81837364	T	C	0.14	0.08	0.01	7.23E-07
CCL13	rs3136674	17	34355597	C	T	0.05	-0.46	0.02	2.14E-80
CCL13	rs892090	19	55027704	G	T	0.83	0.14	0.01	2.05E-21
CCL14	rs13962	1	159205737	A	G	0.18	0.17	0.01	2.35E-32
CCL14	rs13412535	2	224010157	A	G	0.23	-0.07	0.01	3.49E-07
CCL14	rs1354034	3	56815721	C	T	0.60	-0.06	0.01	6.77E-09
CCL14	rs6993770	8	105569300	T	A	0.29	-0.07	0.01	9.62E-10
CCL14	rs774510679	10	63304076	A	C	0.41	0.06	0.01	4.31E-08
CCL14	rs9903158	17	35985301	C	T	0.05	-1.01	0.02	0.00E+00
CCL14	rs1671152	19	55014977	G	T	0.84	0.10	0.01	3.80E-12
CCL15	rs10874777	1	93396463	C	T	0.63	0.04	0.01	8.83E-06
CCL15	rs28817415	4	76480299	T	C	0.46	0.04	0.01	1.74E-05
CCL15	rs782134971	9	133264504	G	C	0.25	-0.14	0.01	1.39E-36

CCL15	rs28929474	14	94378610	T	C	0.02	0.26	0.02	4.33E-15
CCL15	-	17	36001607	C	G	0.93	-1.57	0.01	0.00E+00
CCL15	rs601338	19	48703417	A	G	0.51	-0.18	0.01	1.44E-83
CCL16	rs11127048	2	27529596	A	G	0.62	-0.04	0.01	2.02E-05
CCL16	rs1047891	2	210675783	A	C	0.31	-0.05	0.01	3.50E-06
CCL16	rs4682860	3	42822312	G	A	0.61	-0.04	0.01	2.44E-05
CCL16	rs3134984	6	32677252	A	T	0.21	-0.08	0.01	2.34E-10
CCL16	rs35493868	7	73625076	G	C	0.20	-0.06	0.01	1.82E-06
CCL16	rs964184	11	116778201	C	G	0.87	-0.06	0.01	2.56E-05
CCL16	rs28929474	14	94378610	T	C	0.02	0.15	0.02	6.11E-06
CCL16	rs112689088	17	35980421	C	T	0.10	-1.32	0.01	0.00E+00
CCL16	rs4911405	20	34087161	T	C	0.65	0.05	0.01	1.58E-07
CCL17	rs12075	1	159205564	A	G	0.58	0.05	0.01	3.09E-06
CCL17	rs142289859	3	16913449	C	T	0.03	-0.55	0.02	1.25E-72
CCL17	rs116135711	3	33030977	C	T	0.04	-0.17	0.02	4.41E-08
CCL17	rs2228467	3	42864624	C	T	0.06	0.42	0.02	1.06E-74
CCL17	rs11925560	3	47217970	T	A	0.39	0.06	0.01	6.92E-08
CCL17	rs771327	3	101815656	G	A	0.56	0.06	0.01	8.66E-07
CCL17	rs3804749	3	123114156	T	C	0.59	-0.05	0.01	1.31E-05
CCL17	rs2073643	5	132387596	C	T	0.55	-0.06	0.01	2.39E-07
CCL17	rs3763287	6	31402552	A	C	0.06	-0.10	0.01	6.67E-06
CCL17	rs6993770	8	105569300	T	A	0.29	-0.13	0.01	2.74E-29
CCL17	rs61469632	9	132986603	C	T	0.06	-0.11	0.01	4.31E-07
CCL17	rs71474568	10	102589091	G	T	0.34	0.05	0.01	1.71E-05
CCL17	rs10886430	10	119250744	G	A	0.13	-0.36	0.01	1.13E-106
CCL17	rs9302690	16	57409056	A	G	0.08	0.38	0.01	6.83E-78
CCL17	rs1654425	19	55027612	C	T	0.83	0.08	0.01	1.08E-08
CCL18	rs6715862	2	42101435	G	A	0.50	-0.08	0.01	9.97E-14
CCL18	rs2015086	17	36064257	G	A	0.13	0.70	0.01	0.00E+00
CCL18	rs145144275	19	58350442	G	A	0.01	-0.34	0.04	1.18E-09
CCL18	rs56298400	20	40496162	A	G	0.08	0.08	0.01	1.44E-05
CCL19	rs2476601	1	113834946	G	A	0.90	-0.11	0.01	3.43E-09
CCL19	rs2305625	3	132522666	T	C	0.11	-0.15	0.01	1.18E-17
CCL19	rs201980192	6	32641286	T	C	0.02	0.88	0.03	7.54E-102
CCL19	rs10972202	9	34707817	T	C	0.12	0.10	0.01	1.31E-09
CCL19	rs35350651	12	111469627	C	A	0.50	-0.06	0.01	7.60E-09
CCL19	rs2412973	22	30133642	A	C	0.44	-0.05	0.01	1.87E-06
CCL2	rs12075	1	159205564	A	G	0.58	0.24	0.01	3.22E-110
CCL2	rs2228467	3	42864624	C	T	0.06	0.13	0.01	7.71E-09
CCL2	rs10510751	3	46304534	G	T	0.07	0.28	0.01	9.09E-41
CCL2	rs1130580	6	31268902	C	T	0.29	-0.05	0.01	1.97E-05
CCL2	rs4541868	8	105578477	A	C	0.27	-0.06	0.01	2.03E-06
CCL2	rs7080386	10	63288546	A	C	0.41	0.07	0.01	1.94E-09
CCL2	rs76428106	13	28029870	C	T	0.01	-0.27	0.03	5.11E-08
CCL2	rs12601658	17	34206404	A	T	0.23	-0.07	0.01	1.80E-07
CCL2	rs1671152	19	55014977	G	T	0.84	0.10	0.01	1.38E-11
CCL20	rs1395338	2	227783607	A	G	0.60	0.09	0.01	2.41E-15
CCL20	rs3129754	6	32615269	G	A	0.42	0.08	0.01	1.86E-12
CCL20	rs736794	6	41030094	T	G	0.36	-0.08	0.01	2.11E-10
CCL20	rs77542162	17	69085137	G	A	0.02	0.26	0.02	2.83E-12
CCL20	rs117900576	17	74765056	G	C	0.03	0.18	0.02	1.41E-08
CCL21	rs4671127	2	65335836	G	C	0.34	-0.05	0.01	1.80E-05
CCL21	rs1354034	3	56815721	C	T	0.60	-0.06	0.01	3.57E-08
CCL21	rs11924792	3	132521256	C	A	0.11	-0.18	0.01	1.96E-23
CCL21	rs17138669	5	116016941	T	G	0.14	-0.09	0.01	3.80E-07
CCL21	rs17764730	5	128021834	T	C	0.25	-0.09	0.01	1.71E-11

CCL21	rs12722013	6	33080683	C	T	0.05	0.32	0.02	2.60E-34
CCL21	rs10814138	9	34723038	A	C	0.32	-0.10	0.01	1.29E-16
CCL21	rs3184504	12	111446804	C	T	0.52	-0.08	0.01	1.65E-12
CCL21	rs62054656	16	90070213	A	C	0.06	-0.11	0.01	5.07E-06
CCL21	rs17065796	18	59429782	C	T	0.37	-0.05	0.01	1.54E-05
CCL21	rs2412973	22	30133642	A	C	0.44	-0.07	0.01	4.33E-09
CCL22	rs4683339	3	42835249	T	C	0.40	-0.05	0.01	1.87E-05
CCL22	rs13098877	3	188415375	T	C	0.44	-0.05	0.01	4.00E-06
CCL22	rs9296027	6	32425285	G	C	0.12	0.08	0.01	2.62E-06
CCL22	rs1504215	6	90296508	A	G	0.35	-0.06	0.01	1.59E-07
CCL22	rs3184504	12	111446804	C	T	0.52	-0.06	0.01	6.94E-07
CCL22	rs41398344	16	57360481	C	T	0.01	-0.84	0.04	2.84E-48
CCL22	rs77542162	17	69085137	G	A	0.02	0.51	0.02	5.04E-42
CCL22	rs11670056	19	18479133	T	C	0.07	0.11	0.01	8.77E-07
CCL23	rs61804164	1	161653235	C	G	0.12	-0.08	0.01	1.44E-06
CCL23	rs780093	2	27519736	C	T	0.62	-0.07	0.01	3.23E-09
CCL23	rs142021145	5	72460692	A	C	0.00	0.57	0.06	6.18E-10
CCL23	rs431204	6	32037490	T	C	0.12	-0.08	0.01	2.62E-06
CCL23	rs964184	11	116778201	C	G	0.87	-0.10	0.01	7.24E-11
CCL23	rs7484541	12	57321020	T	A	0.23	-0.06	0.01	4.46E-06
CCL23	rs11545007	16	30532720	A	C	0.01	-0.34	0.04	3.15E-08
CCL23	rs712048	17	35999179	C	A	0.87	0.56	0.01	9.55E-251
CCL23	rs7412	19	44908822	T	C	0.08	0.12	0.01	6.87E-09
CCL24	rs12083692	1	114489767	T	A	0.26	-0.05	0.01	3.29E-11
CCL24	rs2228467	3	42864624	C	T	0.06	0.17	0.01	7.62E-33
CCL24	rs2201150	3	46217411	C	T	0.63	-0.03	0.00	1.36E-05
CCL24	rs9799792	4	975534	C	T	0.56	0.03	0.00	7.39E-06
CCL24	rs115216147	5	71369699	A	T	0.07	-0.08	0.01	7.25E-08
CCL24	rs111872865	6	360124	A	G	0.01	0.16	0.02	7.73E-06
CCL24	rs2024050	7	75831075	G	A	0.90	-1.37	0.01	0.00E+00
CCL24	rs71558590	8	70284854	G	A	0.15	0.04	0.01	2.71E-05
CCL24	rs4876611	8	115659621	G	A	0.72	0.04	0.01	1.48E-06
CCL24	rs13294683	9	79664852	T	C	0.10	0.07	0.01	6.04E-09
CCL24	rs10769256	11	47356845	T	C	0.40	0.06	0.00	5.83E-16
CCL24	rs201510256	11	55025163	T	C	0.47	-0.04	0.01	1.38E-06
CCL24	rs2939849	15	90075850	G	C	0.40	0.06	0.00	1.14E-14
CCL24	rs1121985	16	79329182	C	A	0.62	0.05	0.00	4.39E-11
CCL24	rs11480320	20	53916157	T	A	0.58	0.03	0.00	1.83E-05
CCL25	rs9275576	6	32711549	T	C	0.14	0.06	0.01	3.55E-06
CCL25	-	6	160748021	C	T	0.02	-0.20	0.02	2.59E-08
CCL25	rs60270701	8	10714010	G	C	0.07	0.09	0.01	9.00E-08
CCL25	rs118062058	8	42245860	G	A	0.06	-0.15	0.01	8.00E-14
CCL25	-	9	133263362	G	A	0.18	-0.27	0.01	1.18E-120
CCL25	rs7295693	12	467630	C	T	0.59	0.12	0.01	1.57E-40
CCL25	rs186021206	17	7166093	A	G	0.01	0.40	0.04	2.41E-10
CCL25	rs7222664	17	9881966	C	G	0.33	0.04	0.01	5.76E-06
CCL25	rs112001035	17	68827664	A	G	0.06	-0.10	0.01	7.78E-07
CCL25	rs2032887	19	8056476	G	A	0.24	0.76	0.01	0.00E+00
CCL25	rs601338	19	48703417	A	G	0.51	-0.39	0.01	6.23E-280
CCL25	rs34710186	20	32400102	A	T	0.81	0.06	0.01	9.96E-08
CCL26	rs12075	1	159205564	A	G	0.58	0.19	0.01	3.91E-64
CCL26	rs13412535	2	224010157	A	G	0.23	-0.06	0.01	1.78E-05
CCL26	rs200838	5	132374707	C	A	0.52	0.05	0.01	1.15E-05
CCL26	rs3135383	6	32453024	G	T	0.13	-0.09	0.01	9.59E-08
CCL26	rs41463245	7	75771945	T	C	0.01	-1.01	0.04	1.65E-64
CCL26	-	8	105561296	C	A	0.27	-0.07	0.01	2.53E-07

CCL26	rs10761731	10	63267850	T	A	0.42	0.06	0.01	2.66E-07
CCL26	rs1654425	19	55027612	C	T	0.83	0.09	0.01	6.31E-10
CCL27	rs1150756	6	32067917	T	C	0.13	-0.10	0.01	6.95E-10
CCL27	rs118039278	6	160564494	A	G	0.08	0.11	0.01	1.59E-07
CCL27	rs2070074	9	34649445	G	A	0.10	-0.33	0.01	1.65E-72
CCL27	rs964184	11	116778201	C	G	0.87	-0.10	0.01	6.87E-11
CCL27	rs77542162	17	69085137	G	A	0.02	0.31	0.02	2.17E-17
CCL27	rs7412	19	44908822	T	C	0.08	0.15	0.01	7.23E-14
CCL27	rs2569491	19	51081659	A	G	0.30	-0.06	0.01	8.77E-07
CCL27	rs74278448	22	46139068	G	C	0.70	0.10	0.01	6.21E-17
CCL28	rs7535275	1	150381734	G	A	0.13	-0.08	0.01	4.75E-06
CCL28	rs6671171	1	156901526	A	G	0.11	0.08	0.01	3.43E-06
CCL28	rs3732083	2	206176329	C	T	0.43	-0.18	0.01	5.38E-58
CCL28	rs13412535	2	224010157	A	G	0.23	-0.15	0.01	7.21E-30
CCL28	rs2278668	3	123116385	C	T	0.59	-0.07	0.01	5.77E-09
CCL28	rs55874224	3	143101628	C	G	0.39	0.07	0.01	1.34E-08
CCL28	rs11734132	4	6889792	C	G	0.17	-0.06	0.01	2.79E-05
CCL28	rs200708155	5	43560059	T	G	0.50	-0.06	0.01	8.04E-07
CCL28	rs114694170	5	88884379	C	T	0.06	0.11	0.02	2.76E-06
CCL28	rs112838960	5	132373340	A	T	0.52	0.07	0.01	3.42E-09
CCL28	rs77024756	6	32530781	G	A	0.70	-0.07	0.01	1.36E-07
CCL28	rs778641135	6	135097494	T	C	0.26	0.07	0.01	5.23E-07
CCL28	rs139141690	7	101856650	A	G	0.00	-0.64	0.05	1.10E-14
CCL28	rs10984499	9	97953107	A	G	0.36	0.05	0.01	2.37E-05
CCL28	rs2519093	9	133266456	T	C	0.18	-0.17	0.01	1.42E-32
CCL28	rs10886430	10	119250744	G	A	0.13	-0.08	0.01	1.37E-05
CCL28	rs73000929	11	114082900	A	G	0.04	-0.24	0.02	6.82E-17
CCL28	rs56990409	12	7989603	A	G	0.55	0.09	0.01	6.39E-14
CCL28	rs60822569	12	54323724	C	T	0.55	0.13	0.01	5.27E-32
CCL28	rs141421422	12	108292778	C	A	0.01	-0.32	0.04	5.35E-06
CCL28	rs4965385	15	101455829	G	A	0.28	-0.05	0.01	1.46E-05
CCL28	rs892090	19	55027704	G	T	0.83	0.08	0.01	1.70E-07
CCL28	rs71329093	21	35020267	G	A	0.06	-0.12	0.02	5.25E-07
CCL3	rs60939770	3	46296115	G	A	0.32	-0.05	0.01	6.09E-06
CCL3	rs1354034	3	56815721	C	T	0.60	-0.05	0.01	1.82E-06
CCL3	rs756846124	4	38780642	C	T	0.18	0.07	0.01	3.97E-06
CCL3	rs13153461	5	139472784	A	G	0.74	0.07	0.01	9.82E-09
CCL3	rs9268644	6	32440267	C	A	0.55	0.06	0.01	2.80E-07
CCL3	rs3184504	12	111446804	C	T	0.52	-0.07	0.01	4.39E-12
CCL3	rs7485656	12	124831101	G	A	0.14	0.08	0.01	9.92E-07
CCL3	rs1634486	17	36082734	C	A	0.22	0.42	0.01	4.81E-228
CCL4	rs113341849	3	46342713	A	G	0.12	0.61	0.01	2.81E-285
CCL4	rs756846124	4	38780642	C	T	0.18	0.07	0.01	1.37E-07
CCL4	rs9266806	6	31410245	C	T	0.20	-0.06	0.01	2.79E-05
CCL4	rs3184504	12	111446804	C	T	0.52	-0.06	0.01	4.68E-08
CCL4	rs13338037	16	81841381	G	C	0.14	0.08	0.01	8.07E-08
CCL4	-	17	36463756	T	C	0.12	-0.41	0.01	5.14E-137
CCL4	rs892090	19	55027704	G	T	0.83	0.07	0.01	7.33E-07
CCL5	rs1354034	3	56815721	C	T	0.60	-0.07	0.01	1.02E-09
CCL5	rs6961069	7	80589645	T	C	0.40	0.06	0.01	3.94E-08
CCL5	rs6993770	8	105569300	T	A	0.29	-0.09	0.01	9.94E-14
CCL5	rs35754228	10	37914883	T	A	0.32	-0.08	0.01	6.58E-10
CCL5	rs10761731	10	63267850	T	A	0.42	0.13	0.01	9.36E-29
CCL5	rs11064074	12	6171873	T	C	0.46	-0.05	0.01	3.41E-06
CCL5	rs12445050	16	81837364	T	C	0.14	0.08	0.01	4.57E-07
CCL5	rs2107538	17	35880776	T	C	0.18	-0.22	0.01	7.84E-53

CCL5	rs1654425	19	55027612	C	T	0.83	0.13	0.01	2.72E-17
CCL5	rs3827978	20	19300647	T	C	0.35	0.06	0.01	9.11E-08
CCL7	rs12075	1	159205564	A	G	0.58	-0.27	0.01	2.79E-134
CCL7	rs2228467	3	42864624	C	T	0.06	0.30	0.01	2.26E-39
CCL7	rs28380853	6	31343827	A	T	0.14	0.09	0.01	1.08E-07
CCL7	rs9907136	17	34189226	T	C	0.65	0.11	0.01	5.04E-22
CCL7	rs1654425	19	55027612	C	T	0.83	0.08	0.01	5.02E-08
CCL8	rs12075	1	159205564	A	G	0.58	-0.20	0.01	3.19E-99
CCL8	rs35173808	6	31358371	A	C	0.10	-0.09	0.01	3.63E-08
CCL8	rs1558067	7	30463664	C	T	0.44	0.04	0.01	5.00E-06
CCL8	rs2737245	8	115646356	T	G	0.28	-0.05	0.01	6.81E-06
CCL8	rs61469632	9	132986603	C	T	0.06	-0.09	0.01	8.00E-06
CCL8	rs9414801	10	63389329	A	G	0.53	-0.04	0.01	1.14E-05
CCL8	rs3740688	11	47358789	T	G	0.54	-0.05	0.01	1.22E-06
CCL8	rs12445050	16	81837364	T	C	0.14	0.06	0.01	1.89E-05
CCL8	rs1133763	17	34320812	C	A	0.15	-1.07	0.01	0.00E+00
CCL8	rs62125968	19	3422263	A	G	0.23	0.06	0.01	5.55E-07
CCL8	rs892090	19	55027704	G	T	0.83	0.09	0.01	4.94E-13
CCN1	rs4949896	1	85596653	T	G	0.26	-0.12	0.01	1.97E-20
CCN1	rs1354034	3	56815721	C	T	0.60	-0.06	0.01	1.21E-06
CCN1	rs55730499	6	160584578	T	C	0.08	0.11	0.01	3.11E-08
CCN1	rs17145750	7	73612048	T	C	0.16	-0.07	0.01	9.98E-06
CCN1	rs328	8	19962213	G	C	0.10	-0.12	0.01	4.68E-11
CCN1	rs28601761	8	125487789	G	C	0.42	-0.05	0.01	1.45E-05
CCN1	rs7848055	9	92353210	C	A	0.33	-0.08	0.01	1.25E-11
CCN1	rs964184	11	116778201	C	G	0.87	-0.13	0.01	2.95E-16
CCN1	rs11846959	14	94379979	A	G	0.25	0.08	0.01	1.03E-09
CCN1	rs186021206	17	7166093	A	G	0.01	0.41	0.05	1.17E-07
CCN1	rs445925	19	44912383	A	G	0.11	0.12	0.01	2.63E-12
CCN2	rs1434282	1	199041592	T	C	0.73	-0.08	0.01	4.01E-09
CCN2	rs13412535	2	224010157	A	G	0.23	-0.06	0.01	8.59E-06
CCN2	rs1354034	3	56815721	C	T	0.60	-0.10	0.01	2.86E-17
CCN2	rs28625045	4	101851033	A	G	0.39	-0.06	0.01	5.96E-07
CCN2	rs2631367	5	132369766	G	C	0.52	0.05	0.01	3.82E-06
CCN2	rs3128984	6	31456751	G	A	0.07	-0.11	0.01	2.01E-06
CCN2	rs1917342	6	70640051	G	A	0.30	-0.06	0.01	7.11E-06
CCN2	rs7767139	6	131971641	G	C	0.22	-0.35	0.01	3.78E-145
CCN2	rs13236689	7	80606698	G	T	0.41	0.07	0.01	7.59E-09
CCN2	rs4631576	9	97942928	T	C	0.36	0.06	0.01	1.13E-06
CCN2	rs10761731	10	63267850	T	A	0.41	0.12	0.01	1.24E-26
CCN2	rs79977579	12	54300776	A	C	0.10	0.08	0.01	1.25E-05
CCN2	rs7176023	15	64808808	C	T	0.07	0.12	0.01	2.81E-07
CCN2	rs11075045	16	8940859	G	A	0.18	0.07	0.01	2.05E-06
CCN2	rs12445050	16	81837364	T	C	0.14	0.11	0.01	1.16E-11
CCN2	rs892090	19	55027704	G	T	0.83	0.15	0.01	4.10E-24
CCN2	rs6081565	20	19307260	A	G	0.35	0.07	0.01	1.61E-08
CCN2	rs56045941	21	35049034	C	T	0.17	0.07	0.01	3.27E-06
CCN3	rs2279112	8	119416784	A	G	0.26	-0.27	0.01	3.94E-110
CCN3	rs7827593	8	133012217	A	G	0.13	0.08	0.01	4.31E-07
CCN3	rs11172113	12	57133500	C	T	0.41	0.05	0.01	9.07E-06
CCN4	rs2274319	1	156481081	C	T	0.65	-0.05	0.01	5.01E-06
CCN4	rs68066031	2	224015781	C	T	0.23	-0.10	0.01	5.32E-16
CCN4	rs1354034	3	56815721	C	T	0.60	-0.08	0.01	1.32E-13
CCN4	rs1868715	4	120826815	T	C	0.34	-0.05	0.01	5.07E-06
CCN4	rs114694170	5	88884379	C	T	0.06	0.13	0.01	7.07E-09
CCN4	rs139141690	7	101856650	A	G	0.00	0.37	0.05	1.34E-06

CCN4	rs4541868	8	105578477	A	C	0.27	-0.06	0.01	6.57E-07
CCN4	rs12156037	8	133192215	G	A	0.35	-0.44	0.01	0.00E+00
CCN4	rs150813342	9	132989126	T	C	0.01	0.28	0.04	1.57E-06
CCN4	rs774510679	10	63304076	A	C	0.41	0.07	0.01	4.85E-10
CCN4	rs11591571	10	102583047	A	G	0.34	0.06	0.01	2.08E-07
CCN4	rs562526200	11	32892843	C	G	0.77	0.07	0.01	2.92E-09
CCN4	rs10424665	19	32404725	C	A	0.17	0.07	0.01	4.67E-07
CCN4	rs1654425	19	55027612	C	T	0.83	0.08	0.01	8.77E-09
CCN5	rs4859682	4	76489165	A	C	0.46	0.05	0.01	5.81E-06
CCN5	-	20	44683866	G	C	0.58	0.20	0.01	3.74E-79
CCS	rs498045	11	66595526	G	A	0.53	0.48	0.01	0.00E+00
CD109	rs1260326	2	27508073	C	T	0.61	0.05	0.01	1.64E-07
CD109	-	6	73791270	A	T	0.35	0.85	0.01	0.00E+00
CD109	rs507666	9	133273983	A	G	0.18	-0.23	0.01	8.48E-90
CD109	rs56278466	10	17833858	G	T	0.66	0.09	0.01	5.04E-25
CD109	rs186021206	17	7166093	A	G	0.01	0.56	0.04	6.40E-21
CD109	rs704	17	28367840	A	G	0.47	-0.04	0.01	5.92E-06
CD109	rs868875	19	7766280	G	A	0.30	-0.05	0.01	3.52E-07
CD14	rs1063412	1	172441827	A	G	0.56	0.09	0.01	7.69E-17
CD14	rs5744441	5	140637262	A	G	0.23	-0.32	0.01	1.64E-125
CD14	rs28601761	8	125487789	G	C	0.42	-0.07	0.01	1.95E-10
CD14	-	9	133276163	T	A	0.19	-0.08	0.01	6.26E-08
CD14	rs56278466	10	17833858	G	T	0.66	0.09	0.01	3.96E-14
CD14	rs9414801	10	63389329	A	G	0.53	-0.06	0.01	5.05E-08
CD14	rs149394327	17	66232877	C	G	0.03	0.17	0.02	2.68E-07
CD160	rs6699680	1	24912140	G	A	0.42	-0.05	0.01	3.95E-06
CD160	rs12562475	1	121526855	T	C	0.10	-0.12	0.01	7.52E-10
CD160	rs9728526	1	145718303	A	G	0.44	-0.39	0.01	7.18E-265
CD160	rs2009581	2	111050100	A	G	0.27	-0.16	0.01	2.98E-39
CD160	rs186535521	2	196926710	T	C	0.02	-0.28	0.03	8.24E-10
CD160	rs13081778	3	46912231	G	A	0.37	-0.05	0.01	1.11E-05
CD160	rs777572865	5	132485950	C	A	0.33	-0.07	0.01	3.16E-09
CD160	rs2523609	6	31269478	A	G	0.61	0.11	0.01	1.59E-22
CD160	rs7077401	10	5983915	G	A	0.44	-0.06	0.01	2.62E-07
CD160	rs2497318	10	92672243	T	C	0.45	0.09	0.01	1.07E-17
CD160	rs3016275	11	128197509	T	C	0.34	-0.09	0.01	1.23E-14
CD160	rs3184504	12	111446804	C	T	0.52	-0.14	0.01	7.35E-40
CD160	rs77924615	16	20381010	A	G	0.20	-0.06	0.01	1.78E-05
CD160	rs58745116	17	35476573	A	G	0.39	-0.07	0.01	2.19E-10
CD160	rs516316	19	48702888	C	G	0.51	-0.07	0.01	8.11E-10
CD163	rs2099684	1	161530340	G	A	0.32	0.06	0.01	1.24E-06
CD163	rs62165726	2	134208991	A	C	0.04	-0.68	0.02	9.12E-123
CD163	-	2	202612713	T	A	0.37	0.06	0.01	2.75E-06
CD163	rs11923060	3	186871601	T	C	0.16	-0.08	0.01	1.79E-07
CD163	rs1800759	4	99144358	G	T	0.61	-0.05	0.01	6.71E-06
CD163	rs115216147	5	71369699	A	T	0.07	0.17	0.01	1.88E-14
CD163	rs11381821	6	109320671	A	C	0.47	-0.05	0.01	9.73E-07
CD163	rs2288081	7	130672456	C	T	0.85	0.07	0.01	2.11E-05
CD163	rs10267707	7	150813885	G	C	0.45	0.05	0.01	6.42E-06
CD163	rs4876611	8	115659621	G	A	0.72	0.06	0.01	4.72E-06
CD163	rs2031902	9	33117526	T	C	0.59	-0.09	0.01	1.98E-15
CD163	rs635634	9	133279427	T	C	0.18	0.09	0.01	1.15E-10
CD163	-	9	137074738	C	T	0.63	-0.05	0.01	1.62E-06
CD163	rs768890137	11	47386310	C	T	0.39	0.07	0.01	6.89E-09
CD163	rs11220505	11	126464910	T	C	0.22	0.13	0.01	2.87E-23
CD163	rs74622686	11	134394333	G	A	0.15	0.07	0.01	1.01E-05

CD163	rs11054859	12	7617180	A	G	0.10	-0.18	0.01	2.12E-25
CD163	rs10774625	12	111472415	G	A	0.50	-0.08	0.01	5.54E-13
CD163	rs217184	16	72072066	C	T	0.20	-0.08	0.01	3.22E-09
CD163	rs186021206	17	7166093	A	G	0.01	1.79	0.05	8.43E-130
CD163	rs601338	19	48703417	A	G	0.51	0.06	0.01	3.87E-07
CD163	rs7246479	19	55312964	G	T	0.51	0.05	0.01	2.74E-05
CD164	rs4950771	1	202142651	G	T	0.93	-0.17	0.01	3.11E-13
CD164	rs45492299	1	225866633	A	G	0.18	-0.07	0.01	1.61E-06
CD164	rs9857570	3	58294963	G	A	0.32	-0.09	0.01	6.48E-14
CD164	rs6929023	6	109399147	A	G	0.33	-0.15	0.01	4.59E-36
CD164	rs1692821	8	11842479	T	C	0.28	-0.06	0.01	1.76E-05
CD164	rs4734879	8	105570896	G	A	0.27	-0.06	0.01	7.21E-06
CD164	rs10820606	9	96430637	C	A	0.23	-0.07	0.01	2.66E-07
CD164	rs10740118	10	63341447	C	G	0.41	0.07	0.01	1.14E-08
CD164	rs72701845	14	92750678	A	G	0.04	-0.20	0.02	1.53E-10
CD164	rs892090	19	55027704	G	T	0.83	0.09	0.01	1.82E-09
CD177	rs6796	7	6462736	C	T	0.28	0.04	0.01	1.95E-05
CD177	rs186021206	17	7166093	A	G	0.01	0.28	0.04	8.56E-06
CD177	rs78530667	19	43396545	C	T	0.13	-0.73	0.01	0.00E+00
CD1C	rs61788913	1	59625496	T	G	0.13	-0.10	0.01	2.01E-09
CD1C	rs115248892	1	158292829	T	G	0.00	-1.62	0.07	4.51E-54
CD1C	rs4384720	2	144667834	G	C	0.92	0.11	0.01	1.66E-07
CD1C	rs10189685	2	202623726	A	G	0.30	-0.06	0.01	4.63E-06
CD1C	rs4698932	4	105127990	A	G	0.49	0.05	0.01	3.46E-06
CD1C	rs9265969	6	31347929	A	G	0.16	0.23	0.01	3.96E-53
CD1C	rs11963621	6	44623269	C	T	0.10	0.12	0.01	7.65E-11
CD1C	rs1847472	6	90263440	A	C	0.35	-0.10	0.01	7.68E-17
CD1C	rs3808460	8	115585408	C	T	0.56	-0.05	0.01	9.11E-06
CD1C	rs139130389	11	72139110	A	C	0.07	-0.42	0.01	1.59E-90
CD1C	rs572608	11	118930931	T	A	0.55	0.06	0.01	2.17E-08
CD1C	rs3937026	11	122655058	T	G	0.25	0.07	0.01	1.25E-07
CD1C	rs150926693	12	54232988	T	C	0.10	0.08	0.01	1.14E-05
CD1C	rs653178	12	111569952	T	C	0.52	-0.11	0.01	7.08E-23
CD1C	rs76428106	13	28029870	C	T	0.01	0.36	0.03	2.80E-13
CD1C	rs12599288	16	11728210	C	T	0.30	-0.11	0.01	2.63E-21
CD1C	rs60969446	19	33268667	C	T	0.57	-0.06	0.01	3.37E-07
CD1C	rs17272847	19	49476141	A	G	0.09	0.09	0.01	3.23E-06
CD1C	rs6063502	20	50339058	G	A	0.35	0.07	0.01	1.34E-08
CD200	-	2	27526125	A	T	0.62	0.06	0.01	1.85E-07
CD200	rs6775517	3	58373240	T	G	0.37	0.07	0.01	1.92E-08
CD200	rs13318932	3	98677506	T	C	0.10	0.08	0.01	1.14E-05
CD200	rs2399420	3	112350918	A	C	0.90	0.27	0.01	1.47E-47
CD200	rs8176747	9	133255928	G	C	0.06	0.29	0.01	3.18E-36
CD200	rs56278466	10	17833858	G	T	0.66	0.10	0.01	4.26E-17
CD200	rs36060036	16	20350628	T	C	0.17	-0.07	0.01	5.65E-06
CD200	rs186021206	17	7166093	A	G	0.01	0.35	0.05	6.75E-06
CD200	rs7286917	22	39464863	G	A	0.74	-0.09	0.01	6.57E-12
CD200R1	rs6545928	2	62364985	C	T	0.23	-0.05	0.01	1.09E-06
CD200R1	rs10935473	3	98698056	T	G	0.44	-0.32	0.01	2.56E-167
CD200R1	rs16860233	3	112923275	T	C	0.04	1.81	0.02	0.00E+00
CD200R1	rs3129782	6	32680942	A	G	0.68	-0.08	0.01	9.55E-15
CD200R1	rs544503442	8	104960318	A	G	0.14	0.07	0.01	1.09E-06
CD200R1	-	9	133263362	G	A	0.18	-0.23	0.01	2.87E-86
CD200R1	rs10823374	10	69441960	G	A	0.44	0.06	0.01	2.56E-10
CD200R1	rs2233243	11	60385009	G	A	0.29	0.05	0.01	1.37E-06
CD200R1	rs75071241	11	126362291	A	G	0.06	-0.11	0.01	2.46E-09

CD200R1	rs10774625	12	111472415	G	A	0.50	-0.05	0.01	1.02E-09
CD200R1	rs186021206	17	7166093	A	G	0.01	0.91	0.04	3.82E-50
CD207	-	1	212776703	T	C	0.13	-0.08	0.01	1.11E-06
CD207	rs35533397	2	12560731	T	C	0.23	0.10	0.01	4.50E-12
CD207	rs17718987	2	70833805	C	G	0.32	0.47	0.01	0.00E+00
CD207	-	2	202690981	C	T	0.30	-0.07	0.01	6.81E-08
CD207	rs7432658	3	185330526	C	T	0.79	-0.07	0.01	1.36E-07
CD207	rs3135394	6	32440720	G	A	0.13	-0.18	0.01	4.68E-31
CD207	rs62408218	6	90222139	T	C	0.36	-0.10	0.01	3.56E-21
CD207	rs2021033	6	167110751	A	G	0.49	-0.05	0.01	1.32E-05
CD207	rs989437	7	28790881	G	A	0.62	0.08	0.01	1.91E-12
CD207	rs13275142	8	115590647	A	G	0.57	-0.08	0.01	8.50E-15
CD207	-	9	133274293	A	C	0.18	-0.08	0.01	4.52E-09
CD207	rs17450261	10	89055738	C	T	0.20	0.06	0.01	5.74E-06
CD207	rs79755767	12	54304624	A	G	0.10	0.08	0.01	1.31E-05
CD207	rs647881	12	120872224	G	A	0.49	-0.12	0.01	3.62E-34
CD207	rs34593439	15	78942615	A	G	0.11	-0.09	0.01	2.27E-07
CD207	rs4985556	16	70660097	A	C	0.12	-0.08	0.01	2.69E-07
CD207	rs2139493	16	85988018	C	T	0.27	-0.05	0.01	2.12E-05
CD207	rs7247412	19	51810615	C	T	0.27	-0.06	0.01	4.82E-08
CD209	rs12740374	1	109274968	T	G	0.22	-0.10	0.01	1.25E-21
CD209	rs288257	2	182738765	T	C	0.62	-0.04	0.01	6.86E-06
CD209	rs505922	9	133273813	C	T	0.32	0.82	0.01	0.00E+00
CD209	rs10769256	11	47356845	T	C	0.40	0.05	0.01	3.51E-09
CD209	rs10860738	12	101474219	A	G	0.67	0.04	0.01	9.91E-06
CD209	rs183130	16	56957451	T	C	0.32	0.04	0.01	5.80E-06
CD209	rs77542162	17	69085137	G	A	0.02	0.15	0.02	4.78E-07
CD209	rs4804774	19	7707287	C	G	0.24	-0.41	0.01	1.20E-283
CD22	rs4848370	2	111054088	T	C	0.27	-0.07	0.01	3.30E-09
CD22	rs967367	3	187016678	A	G	0.39	-0.12	0.01	1.00E-26
CD22	rs304154	5	88824599	C	T	0.31	0.05	0.01	5.01E-06
CD22	rs112824187	5	158838300	A	G	0.08	-0.13	0.01	4.66E-10
CD22	rs9271065	6	32607842	G	C	0.85	-0.25	0.01	3.41E-60
CD22	rs116968179	7	50149587	A	G	0.03	-0.13	0.02	1.97E-05
CD22	-	8	129609309	A	T	0.27	-0.06	0.01	2.24E-06
CD22	rs12554596	9	37002145	G	A	0.20	-0.07	0.01	8.98E-07
CD22	-	9	133263362	G	A	0.18	0.07	0.01	1.02E-07
CD22	-	10	61976858	C	T	0.65	-0.07	0.01	3.37E-10
CD22	rs12784975	10	96620380	C	T	0.19	-0.07	0.01	1.63E-06
CD22	rs11223794	11	134417502	T	C	0.16	0.06	0.01	2.73E-05
CD22	rs111338191	12	111388673	T	A	0.52	-0.05	0.01	2.20E-06
CD22	rs3213566	12	120784775	C	T	0.53	-0.05	0.01	1.12E-05
CD22	rs76428106	13	28029870	C	T	0.01	0.22	0.03	1.03E-05
CD22	rs374039502	13	108308037	A	T	0.02	0.33	0.03	6.43E-17
CD22	rs9939427	16	85982485	A	G	0.23	0.07	0.01	1.45E-08
CD22	rs186021206	17	7166093	A	G	0.01	1.01	0.05	7.47E-44
CD22	rs34557412	17	16948873	G	A	0.01	0.73	0.04	2.51E-29
CD22	rs35222145	17	39851068	G	T	0.49	-0.05	0.01	7.52E-07
CD22	rs10411704	19	35309759	T	G	0.79	-0.29	0.01	6.92E-113
CD22	rs492602	19	48703160	G	A	0.51	0.06	0.01	8.31E-08
CD22	rs6065926	20	46107215	G	A	0.75	-0.07	0.01	3.04E-08
CD22	rs73167342	22	39438097	G	C	0.74	-0.06	0.01	7.89E-07
CD22	rs73165110	22	41895852	A	G	0.08	-0.13	0.01	1.11E-10
CD244	rs37456	1	43827317	T	C	0.35	0.07	0.01	7.97E-09
CD244	rs115868021	1	160841584	G	C	0.01	-2.17	0.05	1.44E-189
CD244	rs12616639	2	62302790	G	A	0.61	0.11	0.01	2.08E-21

CD244	rs2009581	2	111050100	A	G	0.27	-0.09	0.01	3.21E-12
CD244	-	2	134248686	A	G	0.78	-0.06	0.01	1.32E-05
CD244	rs34458031	3	99033191	A	G	0.09	0.08	0.01	2.46E-05
CD244	rs6993770	8	105569300	T	A	0.29	0.08	0.01	3.81E-10
CD244	rs10815098	9	4865338	T	C	0.21	0.07	0.01	4.60E-08
CD244	rs113315674	9	97957771	A	G	0.37	0.08	0.01	6.10E-13
CD244	rs774510679	10	63304076	A	C	0.41	0.06	0.01	1.72E-07
CD244	rs12574844	11	126403491	A	G	0.07	-0.13	0.01	3.18E-09
CD244	rs79977579	12	54300776	A	C	0.10	0.12	0.01	5.21E-11
CD244	rs3184504	12	111446804	C	T	0.52	-0.15	0.01	1.79E-44
CD244	rs10146962	14	100704203	C	T	0.34	-0.06	0.01	2.93E-06
CD244	rs186021206	17	7166093	A	G	0.01	1.30	0.05	6.84E-68
CD244	rs2659005	17	81244914	T	C	0.44	0.09	0.01	2.99E-15
CD27	rs150816167	1	179602727	C	T	0.04	0.14	0.02	1.04E-06
CD27	rs3998176	6	32646350	G	C	0.22	0.06	0.01	2.04E-05
CD27	rs4722557	7	25979936	A	G	0.26	-0.06	0.01	1.50E-06
CD27	rs25680	12	6445462	A	G	0.24	0.29	0.01	1.95E-109
CD27	rs3184504	12	111446804	C	T	0.52	-0.07	0.01	1.67E-10
CD27	rs150861794	13	108351457	T	C	0.02	0.22	0.03	1.37E-06
CD27	rs34562254	17	16939677	A	G	0.10	0.10	0.01	4.88E-08
CD274	rs1801274	1	161509955	G	A	0.53	0.06	0.01	5.21E-08
CD274	rs78444298	1	184702964	A	G	0.02	0.20	0.03	6.18E-07
CD274	rs1354034	3	56815721	C	T	0.60	0.05	0.01	9.31E-06
CD274	rs6993770	8	105569300	T	A	0.29	-0.06	0.01	7.40E-07
CD274	rs822340	9	5453260	G	A	0.75	0.34	0.01	7.85E-154
CD274	rs111338191	12	111388673	T	A	0.52	-0.08	0.01	1.52E-13
CD274	rs112635299	14	94371805	T	G	0.02	0.22	0.02	7.88E-09
CD274	rs8178824	17	66228657	T	C	0.03	0.15	0.02	5.79E-06
CD276	rs4665972	2	27375230	C	T	0.61	0.06	0.01	8.77E-09
CD276	rs10740131	10	63511728	T	A	0.47	0.04	0.01	9.89E-06
CD276	rs1042704	14	22843385	A	G	0.22	0.06	0.01	6.46E-07
CD276	rs2239651	14	94382210	C	T	0.27	-0.07	0.01	6.01E-11
CD276	rs2291014	15	73704018	A	G	0.10	-1.32	0.01	0.00E+00
CD276	rs200489612	17	7203059	A	G	0.01	0.44	0.04	1.78E-10
CD276	rs62217923	21	41167366	G	C	0.26	0.07	0.01	3.92E-12
CD28	-	1	43812246	G	A	0.29	0.12	0.01	1.59E-20
CD28	rs7369876	2	203771749	T	C	0.83	0.26	0.01	2.60E-65
CD28	rs9838308	3	170033504	C	T	0.37	-0.12	0.01	1.81E-22
CD28	rs17612562	6	32647669	G	T	0.33	-0.06	0.01	2.38E-05
CD28	rs6796	7	6462736	C	T	0.28	0.06	0.01	2.72E-06
CD28	rs80318131	7	129143002	C	T	0.31	0.11	0.01	2.41E-17
CD28	rs1461729	8	9329732	G	A	0.90	-0.09	0.01	1.25E-06
CD28	rs34357864	9	133270497	A	G	0.32	0.08	0.01	2.94E-10
CD28	rs2868996	11	47553818	T	A	0.34	-0.06	0.01	1.72E-06
CD28	rs78689694	11	126364925	C	G	0.13	-0.10	0.01	6.25E-09
CD28	rs35350651	12	111469627	C	A	0.50	-0.09	0.01	1.67E-16
CD28	rs71126747	14	65443409	A	C	0.67	-0.06	0.01	1.15E-06
CD28	rs7161799	15	58478324	T	C	0.08	0.13	0.01	1.17E-09
CD28	rs186021206	17	7166093	A	G	0.01	1.36	0.05	1.92E-68
CD28	rs2725405	17	81246424	C	G	0.44	0.06	0.01	3.40E-08
CD2AP	rs1354034	3	56815721	C	T	0.60	0.06	0.01	1.49E-08
CD2AP	-	6	47469961	A	C	0.28	0.30	0.01	3.37E-133
CD300C	rs61747728	1	179557079	T	C	0.04	0.12	0.02	2.10E-05
CD300C	rs11888817	2	62303845	C	T	0.64	0.12	0.01	3.65E-27
CD300C	rs79935131	3	98779723	T	G	0.09	0.14	0.01	1.59E-13
CD300C	rs13107325	4	102267552	T	C	0.08	0.14	0.01	2.52E-12

CD300C	rs509227	6	10535358	C	A	0.52	0.06	0.01	7.57E-10
CD300C	rs17843693	6	32654345	A	G	0.37	0.06	0.01	1.08E-05
CD300C	rs4413619	6	95635103	C	T	0.89	-0.08	0.01	1.08E-05
CD300C	rs143110592	7	7196500	T	A	0.19	-0.06	0.01	5.58E-06
CD300C	rs635634	9	133279427	T	C	0.18	0.06	0.01	2.57E-05
CD300C	rs950802	11	60385111	A	G	0.31	0.05	0.01	2.29E-05
CD300C	rs181897168	11	126357200	A	G	0.01	0.26	0.04	4.09E-06
CD300C	rs774868556	12	89431449	A	A	0.31	-0.35	0.01	7.67E-186
CD300C	rs35350651	12	111469627	C	A	0.50	-0.07	0.01	2.22E-11
CD300C	-	12	120912293	G	T	0.48	-0.05	0.01	1.21E-05
CD300C	rs186021206	17	7166093	A	G	0.01	1.06	0.05	6.27E-46
CD300C	rs58440339	17	74543335	T	C	0.09	0.68	0.01	3.03E-289
CD300C	rs2659005	17	81244914	T	C	0.44	0.06	0.01	2.14E-07
CD300E	rs748544288	1	150635844	C	G	0.23	-0.06	0.01	5.14E-07
CD300E	rs13164856	5	132477512	C	T	0.29	-0.05	0.01	1.48E-05
CD300E	rs532143340	6	32522301	G	C	0.66	0.07	0.01	3.43E-08
CD300E	rs3778752	7	128939993	T	G	0.46	0.08	0.01	3.14E-14
CD300E	rs3184504	12	111446804	C	T	0.52	-0.09	0.01	2.45E-19
CD300E	rs76428106	13	28029870	C	T	0.01	0.29	0.03	3.64E-09
CD300E	rs1987102	15	79973546	C	T	0.26	-0.06	0.01	4.17E-07
CD300E	rs77924615	16	20381010	A	G	0.20	-0.06	0.01	1.75E-05
CD300E	rs424971	16	85912844	C	T	0.44	-0.05	0.01	3.42E-06
CD300E	rs6503533	17	40028327	T	C	0.62	-0.05	0.01	1.11E-05
CD300E	rs1719465	17	74580252	C	T	0.69	-0.36	0.01	2.91E-209
CD300E	rs4760	19	43648948	G	A	0.16	0.13	0.01	7.05E-21
CD300LF	rs61747728	1	179557079	T	C	0.04	0.09	0.01	2.79E-05
CD300LF	rs2256974	6	31587615	A	C	0.17	0.05	0.01	2.27E-05
CD300LF	rs149110519	6	144064640	T	C	0.04	0.09	0.01	1.95E-05
CD300LF	rs12667978	7	129137407	T	C	0.57	-0.04	0.01	4.72E-07
CD300LF	rs909098	11	308716	T	C	0.06	-0.08	0.01	4.39E-07
CD300LF	rs3184504	12	111446804	C	T	0.52	-0.06	0.01	1.96E-15
CD300LF	rs28640218	16	20347945	T	G	0.19	-0.05	0.01	6.73E-07
CD300LF	rs2241244	17	39997301	C	G	0.54	-0.06	0.01	2.35E-07
CD300LF	rs35489971	17	74704804	G	A	0.81	1.29	0.01	0.00E+00
CD300LG	rs61747728	1	179557079	T	C	0.04	0.15	0.02	1.55E-07
CD300LG	rs78058190	2	218835276	A	G	0.05	-0.13	0.02	9.45E-07
CD300LG	rs1801282	3	12351626	G	C	0.12	0.08	0.01	2.42E-07
CD300LG	rs13127398	4	102000547	A	T	0.07	0.10	0.01	3.54E-06
CD300LG	rs11967262	6	43792590	G	C	0.49	-0.08	0.01	7.42E-16
CD300LG	rs28588745	8	10789534	T	A	0.21	-0.10	0.01	1.03E-15
CD300LG	rs8176741	9	133256074	A	G	0.06	0.23	0.01	2.32E-25
CD300LG	rs2068888	10	93079885	A	G	0.45	0.05	0.01	2.20E-06
CD300LG	rs71468663	11	64250632	C	A	0.05	-0.13	0.02	1.59E-07
CD300LG	rs1716403	12	124035299	C	T	0.68	-0.05	0.01	2.69E-06
CD300LG	rs174418	15	58395404	C	T	0.60	-0.06	0.01	2.92E-07
CD300LG	rs777834943	16	20355409	T	C	0.18	-0.07	0.01	5.62E-07
CD300LG	rs12446515	16	56953103	T	C	0.32	0.06	0.01	9.46E-08
CD300LG	rs72836561	17	43848758	T	C	0.03	-1.07	0.02	2.57E-271
CD302	rs2556106	2	159798013	T	G	0.87	-0.42	0.01	1.60E-148
CD302	rs10073754	5	179879517	G	A	0.12	0.10	0.01	1.33E-09
CD302	rs9260603	6	29954977	T	C	0.44	-0.10	0.01	4.90E-19
CD302	rs782134971	9	133264504	G	C	0.25	-0.07	0.01	2.10E-09
CD302	rs7310409	12	120987058	G	A	0.62	-0.07	0.01	1.28E-09
CD302	rs9302635	16	72110275	C	T	0.18	-0.06	0.01	5.92E-06
CD302	rs8178824	17	66228657	T	C	0.03	0.30	0.02	1.31E-21
CD302	rs11662351	18	32230496	C	T	0.39	0.16	0.01	3.66E-49

CD33	rs62165726	2	134208991	A	C	0.04	-0.10	0.01	6.55E-11
CD33	rs56094988	3	98771605	C	G	0.09	0.07	0.01	3.16E-12
CD33	rs7865362	9	33117967	T	C	0.36	0.03	0.00	2.82E-06
CD33	rs10823374	10	69441960	G	A	0.44	0.05	0.00	8.48E-17
CD33	rs139130389	11	72139110	A	C	0.07	-0.05	0.01	6.31E-06
CD33	rs74612335	11	126368738	C	T	0.13	-0.04	0.01	5.25E-06
CD33	rs186021206	17	7166093	A	G	0.01	0.63	0.03	9.06E-58
CD33	rs2455069	19	51225385	G	A	0.43	1.17	0.01	0.00E+00
CD34	rs376752045	1	207900028	C	T	0.00	-1.04	0.13	1.48E-07
CD34	rs532436	9	133274414	A	G	0.19	-0.46	0.01	2.38E-216
CD34	rs35166255	11	126431861	A	G	0.03	0.21	0.02	7.06E-12
CD34	rs186021206	17	7166093	A	G	0.01	0.73	0.05	8.57E-22
CD38	rs28703311	4	15774228	A	G	0.22	-0.43	0.01	0.00E+00
CD38	rs7137828	12	111494996	T	C	0.52	-0.06	0.01	2.78E-10
CD4	rs147599819	6	32627039	T	G	0.40	-0.12	0.01	1.31E-24
CD4	rs964184	11	116778201	C	G	0.87	-0.10	0.01	1.08E-09
CD4	rs73053728	12	6787380	G	A	0.30	-0.36	0.01	1.21E-193
CD4	rs3184504	12	111446804	C	T	0.52	-0.09	0.01	6.39E-16
CD4	rs72654473	19	44911142	A	C	0.11	0.10	0.01	1.62E-08
CD40	rs1354034	3	56815721	C	T	0.60	0.09	0.01	1.69E-15
CD40	rs342293	7	106731773	G	C	0.46	-0.07	0.01	5.63E-09
CD40	rs6993770	8	105569300	T	A	0.29	-0.07	0.01	4.18E-09
CD40	rs7896518	10	63344740	G	A	0.42	0.07	0.01	6.87E-11
CD40	rs73396521	11	267568	A	G	0.25	0.06	0.01	8.99E-06
CD40	rs4608137	12	6184207	A	G	0.49	-0.05	0.01	2.18E-06
CD40	rs3184504	12	111446804	C	T	0.52	-0.05	0.01	1.35E-06
CD40	rs892090	19	55027704	G	T	0.83	0.07	0.01	2.08E-06
CD40	rs13345625	19	57324544	A	C	0.36	-0.05	0.01	2.52E-05
CD40	rs4810485	20	46119308	G	T	0.75	0.42	0.01	4.15E-229
CD40LG	rs13084580	3	39146691	T	C	0.11	0.08	0.01	5.13E-06
CD40LG	rs1354034	3	56815721	C	T	0.60	-0.07	0.01	4.00E-09
CD40LG	rs4698839	4	101880784	T	C	0.40	-0.06	0.01	1.09E-07
CD40LG	rs6848819	4	153530526	C	A	0.03	0.14	0.02	1.10E-05
CD40LG	rs2248116	5	132468655	A	C	0.57	0.06	0.01	2.07E-06
CD40LG	rs6961069	7	80589645	T	C	0.40	0.05	0.01	9.09E-06
CD40LG	rs6993770	8	105569300	T	A	0.29	-0.13	0.01	2.97E-25
CD40LG	rs151234	16	28494339	C	G	0.13	0.08	0.01	1.56E-05
CD40LG	rs12445050	16	81837364	T	C	0.14	0.09	0.01	3.10E-08
CD40LG	rs1654425	19	55027612	C	T	0.83	0.14	0.01	1.58E-18
CD40LG	rs13038837	20	19279307	A	G	0.34	0.06	0.01	1.36E-06
CD40LG	rs4239702	20	46120612	C	T	0.72	-0.11	0.01	2.58E-19
CD46	rs61747728	1	179557079	T	C	0.04	0.16	0.02	5.73E-08
CD46	rs1970530	1	207750721	A	G	0.59	-0.09	0.01	2.99E-14
CD46	rs6993770	8	105569300	T	A	0.29	-0.06	0.01	9.89E-07
CD46	rs579459	9	133278724	C	T	0.21	-0.06	0.01	1.48E-05
CD46	rs10740131	10	63511728	T	A	0.47	0.06	0.01	2.07E-07
CD46	rs972570	10	69512262	T	C	0.49	0.08	0.01	3.71E-12
CD46	rs545303153	17	7251756	A	G	0.00	0.63	0.08	1.59E-07
CD46	rs1654425	19	55027612	C	T	0.83	0.07	0.01	3.01E-06
CD48	-	1	43812246	G	A	0.29	0.06	0.01	9.26E-07
CD48	rs2295615	1	160684968	G	C	0.14	-0.92	0.01	0.00E+00
CD48	rs4672507	2	62343438	A	T	0.64	0.06	0.01	3.06E-08
CD48	rs6791438	3	170031236	G	A	0.37	-0.07	0.01	3.66E-12
CD48	rs3872724	3	187023433	T	C	0.37	-0.05	0.01	2.44E-07
CD48	rs35467801	5	35881028	T	G	0.27	-0.05	0.01	3.74E-06
CD48	rs2844622	6	31272283	G	A	0.66	0.07	0.01	1.14E-11

CD48	rs60843925	11	126368937	C	T	0.14	-0.08	0.01	8.34E-08
CD48	rs3184504	12	111446804	C	T	0.52	-0.14	0.01	2.83E-44
CD48	rs2393716	12	120904130	G	C	0.48	-0.06	0.01	1.01E-09
CD48	rs76428106	13	28029870	C	T	0.01	0.25	0.03	6.95E-08
CD48	rs186021206	17	7166093	A	G	0.01	1.18	0.04	4.25E-67
CD48	rs2659005	17	81244914	T	C	0.44	0.10	0.01	1.32E-21
CD48	rs78530667	19	43396545	C	T	0.13	-0.07	0.01	4.33E-06
CD5	rs10214237	5	35883632	C	T	0.27	-0.08	0.01	1.95E-10
CD5	rs952579	6	21884209	A	G	0.14	0.07	0.01	1.46E-05
CD5	rs9266635	6	31379202	T	C	0.77	-0.08	0.01	1.34E-08
CD5	rs4939490	11	61026179	G	C	0.39	-0.14	0.01	7.70E-33
CD5	rs3184504	12	111446804	C	T	0.52	-0.15	0.01	4.12E-41
CD5	rs7161799	15	58478324	T	C	0.08	0.13	0.01	1.73E-10
CD5	rs7208422	17	78134494	T	A	0.48	-0.05	0.01	5.62E-06
CD5	rs4893	19	48330351	G	A	0.04	-0.13	0.02	9.45E-06
CD55	rs1583090	1	207262438	C	T	0.22	-0.48	0.01	2.38E-274
CD55	rs1260326	2	27508073	C	T	0.61	0.05	0.01	2.55E-05
CD55	rs13107325	4	102267552	T	C	0.08	0.14	0.01	1.43E-10
CD55	-	7	7216532	C	T	0.61	0.06	0.01	1.52E-07
CD55	rs2978024	8	133536866	A	C	0.68	0.08	0.01	6.50E-11
CD55	rs774510679	10	63304076	A	C	0.41	0.05	0.01	1.65E-06
CD55	rs777834943	16	20355409	T	C	0.18	-0.07	0.01	7.44E-06
CD55	rs186021206	17	7166093	A	G	0.01	1.07	0.05	1.63E-45
CD55	rs3814995	19	35851310	T	C	0.32	0.06	0.01	7.72E-07
CD58	rs10801908	1	116547871	T	C	0.12	-0.26	0.01	3.41E-51
CD58	rs3213563	1	172443855	C	T	0.56	-0.18	0.01	2.89E-56
CD58	rs1260326	2	27508073	C	T	0.61	0.05	0.01	2.77E-06
CD58	rs10935473	3	98698056	T	G	0.44	-0.06	0.01	4.14E-08
CD58	rs13064814	3	186936977	G	A	0.71	-0.06	0.01	9.60E-07
CD58	rs9987289	8	9325848	G	A	0.91	-0.11	0.01	2.59E-08
CD58	rs2519093	9	133266456	T	C	0.18	-0.45	0.01	6.65E-212
CD58	rs2393969	10	63380680	C	A	0.47	0.05	0.01	1.37E-06
CD58	rs3967200	11	126362490	T	C	0.13	-0.10	0.01	9.88E-10
CD58	rs186021206	17	7166093	A	G	0.01	1.28	0.05	1.22E-64
CD58	rs2659005	17	81244914	T	C	0.44	0.05	0.01	3.56E-06
CD58	rs4760	19	43648948	G	A	0.16	0.08	0.01	4.85E-08
CD59	rs400642	1	196788128	T	C	0.47	-0.07	0.01	1.46E-10
CD59	rs1398018	4	76451770	C	T	0.44	0.05	0.01	3.27E-06
CD59	rs2273121	11	33736224	A	G	0.26	-0.26	0.01	2.48E-101
CD59	rs77924615	16	20381010	A	G	0.20	-0.07	0.01	6.45E-08
CD59	rs407307	17	39670910	G	A	0.68	0.05	0.01	5.26E-06
CD6	rs7578575	2	25265950	A	T	0.29	0.05	0.01	8.84E-06
CD6	rs4672497	2	62296430	G	C	0.22	0.05	0.01	1.68E-06
CD6	rs57383920	3	47207585	A	T	0.19	-0.05	0.01	1.72E-05
CD6	rs10214273	5	35883884	G	T	0.27	-0.05	0.01	2.33E-05
CD6	rs62454712	7	6458426	C	T	0.24	0.05	0.01	2.20E-05
CD6	rs10957898	8	78705078	A	G	0.21	0.05	0.01	2.79E-05
CD6	rs11230563	11	61008737	T	C	0.35	-0.81	0.01	0.00E+00
CD6	rs3184504	12	111446804	C	T	0.52	-0.11	0.01	4.72E-31
CD6	rs7161799	15	58478324	T	C	0.08	0.10	0.01	1.90E-08
CD6	rs71374020	16	30641303	T	A	0.30	-0.05	0.01	5.59E-06
CD6	rs186021206	17	7166093	A	G	0.01	0.48	0.04	2.71E-14
CD6	rs7208422	17	78134494	T	A	0.48	-0.07	0.01	6.22E-16
CD6	rs28413174	19	17405811	T	C	0.12	0.11	0.01	2.03E-14
CD63	rs2278668	3	123116385	C	T	0.59	-0.06	0.01	1.23E-06
CD63	rs755492124	4	101856385	T	G	0.39	-0.07	0.01	1.36E-09

CD63	rs11242109	5	132341354	T	G	0.48	-0.07	0.01	1.22E-08
CD63	rs342299	7	106733272	T	C	0.46	-0.05	0.01	7.60E-06
CD63	rs6993770	8	105569300	T	A	0.29	-0.17	0.01	5.28E-44
CD63	-	10	63433569	C	T	0.49	-0.05	0.01	2.95E-06
CD63	rs113422568	10	102578181	A	G	0.33	0.09	0.01	1.78E-13
CD63	rs187372065	12	55798095	G	C	0.00	-0.72	0.07	6.72E-10
CD63	rs12445050	16	81837364	T	C	0.14	0.08	0.01	8.11E-07
CD63	rs35496032	19	38326988	A	G	0.01	0.37	0.03	1.42E-13
CD63	rs892090	19	55027704	G	T	0.83	0.14	0.01	7.44E-21
CD63	rs4813637	20	3702115	A	G	0.59	-0.11	0.01	8.96E-21
CD69	rs1354034	3	56815721	C	T	0.60	0.09	0.01	7.63E-14
CD69	rs3804749	3	123114156	T	C	0.59	-0.05	0.01	1.34E-05
CD69	rs6993770	8	105569300	T	A	0.29	-0.12	0.01	4.03E-22
CD69	rs7896518	10	63344740	G	A	0.42	0.09	0.01	5.32E-15
CD69	rs12819552	12	9767236	G	A	0.42	-0.05	0.01	1.59E-05
CD69	rs200309755	17	29518756	T	C	0.54	0.05	0.01	2.74E-05
CD69	rs1671152	19	55014977	G	T	0.84	0.07	0.01	2.37E-05
CD70	rs17118876	1	59151238	G	C	0.57	-0.04	0.01	3.75E-06
CD70	rs12740374	1	109274968	T	G	0.22	-0.30	0.01	2.10E-205
CD70	rs57894427	6	29938267	C	T	0.09	0.07	0.01	1.48E-06
CD70	rs7254538	19	6611325	C	G	0.54	0.75	0.01	0.00E+00
CD74	rs79078220	5	150414087	A	G	0.11	-0.09	0.01	6.54E-07
CD74	rs9273368	6	32658698	A	G	0.30	0.18	0.01	6.92E-51
CD74	-	9	133263362	G	A	0.18	-0.14	0.01	2.51E-25
CD74	rs3184504	12	111446804	C	T	0.52	-0.13	0.01	1.12E-32
CD74	rs7194862	16	10871258	C	T	0.29	0.14	0.01	3.74E-31
CD74	rs186021206	17	7166093	A	G	0.01	0.39	0.05	1.61E-07
CD74	rs3827211	21	41247327	G	A	0.45	-0.05	0.01	2.57E-06
CD79B	rs72704449	1	179504138	C	T	0.04	0.13	0.02	8.97E-06
CD79B	rs78444298	1	184702964	A	G	0.02	0.31	0.03	1.57E-14
CD79B	rs112151347	2	226830730	A	G	0.03	-0.21	0.02	5.40E-09
CD79B	rs77397204	3	187021111	C	A	0.39	-0.06	0.01	2.13E-08
CD79B	rs112824187	5	158838300	A	G	0.08	-0.12	0.01	2.02E-08
CD79B	rs55654737	5	179791841	A	G	0.34	-0.09	0.01	2.43E-13
CD79B	rs2523583	6	31360118	A	G	0.71	-0.06	0.01	5.53E-06
CD79B	rs2395904	8	129605573	G	T	0.27	-0.06	0.01	5.75E-06
CD79B	rs507666	9	133273983	A	G	0.18	-0.24	0.01	1.92E-65
CD79B	rs7087507	10	61985930	G	A	0.35	0.06	0.01	1.60E-06
CD79B	rs111338191	12	111388673	T	A	0.52	-0.07	0.01	2.20E-10
CD79B	rs76428106	13	28029870	C	T	0.01	0.26	0.03	4.34E-07
CD79B	rs9939427	16	85982485	A	G	0.23	0.07	0.01	4.71E-07
CD79B	rs186021206	17	7166093	A	G	0.01	0.81	0.05	9.47E-27
CD79B	rs12946510	17	39756124	T	C	0.48	-0.06	0.01	2.01E-07
CD79B	rs12946669	17	63928647	T	C	0.39	-0.16	0.01	2.93E-47
CD79B	rs7213540	17	81282112	T	C	0.54	-0.05	0.01	3.33E-06
CD79B	rs4674	19	41424491	G	A	0.61	0.07	0.01	8.90E-09
CD83	rs11808882	1	212790930	T	A	0.15	-0.07	0.01	1.78E-05
CD83	rs35332487	2	62283253	A	C	0.31	-0.06	0.01	2.21E-06
CD83	rs7640397	3	46948338	T	C	0.58	-0.05	0.01	3.31E-06
CD83	rs12526086	6	14105666	A	G	0.22	0.20	0.01	9.99E-51
CD83	rs2394982	6	31345253	T	C	0.31	0.06	0.01	7.24E-07
CD83	rs7801921	7	6351186	G	C	0.46	0.05	0.01	2.46E-06
CD83	rs4424335	9	133238172	G	C	0.08	0.12	0.01	8.42E-09
CD83	rs7869	10	71816550	T	C	0.29	0.10	0.01	6.46E-16
CD83	rs4753126	11	94128854	T	C	0.50	0.05	0.01	9.73E-06
CD83	rs3184504	12	111446804	C	T	0.52	-0.11	0.01	4.66E-24

CD83	rs534297892	12	120911539	C	A	0.50	-0.07	0.01	4.21E-10
CD83	rs76428106	13	28029870	C	T	0.01	0.35	0.03	6.72E-12
CD83	rs186021206	17	7166093	A	G	0.01	0.46	0.05	2.49E-09
CD83	rs2150879	17	59781849	A	G	0.55	0.05	0.01	2.49E-06
CD83	rs34878396	19	3546266	T	C	0.01	0.31	0.03	3.97E-10
CD84	rs11265440	1	160583854	G	A	0.92	-0.20	0.01	1.47E-22
CD84	rs41315846	1	247549001	C	T	0.48	-0.06	0.01	1.70E-06
CD84	rs1354034	3	56815721	C	T	0.60	0.08	0.01	9.88E-13
CD84	rs114694170	5	88884379	C	T	0.06	0.13	0.02	2.14E-07
CD84	rs778641135	6	135097494	T	C	0.26	0.07	0.01	6.08E-08
CD84	rs6993770	8	105569300	T	A	0.29	-0.08	0.01	7.03E-10
CD84	rs409801	9	4744743	C	T	0.51	0.07	0.01	4.13E-09
CD84	rs467369	9	134040643	T	C	0.53	0.05	0.01	2.27E-05
CD84	rs7080386	10	63288546	A	C	0.41	0.08	0.01	2.86E-11
CD84	rs597808	12	111535554	G	A	0.52	-0.08	0.01	2.41E-12
CD84	rs10131298	14	103102277	A	T	0.25	0.21	0.01	3.63E-59
CD84	rs151165225	19	45257662	T	C	0.26	-0.09	0.01	1.71E-12
CD8A	rs3020726	2	86789383	G	A	0.16	0.36	0.01	8.69E-127
CD8A	rs35467801	5	35881028	T	G	0.27	-0.07	0.01	3.98E-08
CD8A	rs12211087	6	31302169	A	T	0.09	0.47	0.01	3.02E-134
CD8A	rs12360863	11	11289644	T	C	0.25	-0.06	0.01	5.38E-06
CD8A	rs597808	12	111535554	G	A	0.52	-0.07	0.01	1.79E-09
CD93	rs61747728	1	179557079	T	C	0.04	0.18	0.02	5.25E-09
CD93	rs1260326	2	27508073	C	T	0.61	0.06	0.01	5.08E-07
CD93	rs9264666	6	31271519	C	G	0.55	-0.05	0.01	2.73E-05
CD93	rs7821812	8	10786591	C	G	0.21	-0.08	0.01	3.90E-08
CD93	rs4962111	9	133237150	C	T	0.09	0.12	0.01	5.21E-09
CD93	rs12245367	10	63561704	T	C	0.48	-0.05	0.01	1.93E-05
CD99	rs1260326	2	27508073	C	T	0.61	0.06	0.01	1.53E-08
CD99	rs12149545	16	56959249	A	G	0.32	0.05	0.01	2.67E-05
CD99	rs704	17	28367840	A	G	0.47	0.06	0.01	2.10E-09
CD99	rs673408	18	32220156	G	A	0.46	-0.11	0.01	8.38E-28
CD99L2	rs4665710	2	20998163	C	A	0.79	-0.07	0.01	1.51E-06
CD99L2	rs139039156	3	123123409	A	G	0.01	-0.29	0.04	7.62E-06
CD99L2	rs6054	4	154568456	T	C	0.01	0.38	0.05	1.07E-06
CD99L2	rs73015965	6	160706469	G	A	0.01	0.35	0.05	6.38E-06
CD99L2	rs896173	7	35695927	A	G	0.42	-0.06	0.01	8.43E-07
CD99L2	rs10740131	10	63511728	T	A	0.47	0.05	0.01	3.39E-06
CD99L2	rs174536	11	61784455	C	A	0.35	0.12	0.01	5.17E-24
CD99L2	rs1801689	17	66214462	C	A	0.03	0.48	0.02	1.99E-47
CD99L2	rs1065853	19	44909976	T	G	0.08	0.13	0.01	4.43E-10
CDC27	rs1354034	3	56815721	C	T	0.60	0.05	0.01	2.15E-05
CDC27	rs56287938	17	47220547	G	C	0.10	0.14	0.01	1.65E-12
CDC37	rs1354034	3	56815721	C	T	0.60	0.06	0.01	5.86E-07
CDC37	rs342298	7	106733200	T	C	0.46	-0.06	0.01	1.44E-07
CDC37	-	10	63156270	A	T	0.49	0.05	0.01	1.36E-05
CDC37	rs73396521	11	267568	A	G	0.25	0.06	0.01	2.41E-05
CDCP1	rs2276862	3	45146293	G	C	0.17	-0.30	0.01	4.09E-119
CDCP1	rs113787140	6	32535124	A	T	0.50	-0.08	0.01	4.12E-17
CDCP1	rs77029323	10	62690119	T	G	0.40	0.06	0.01	6.67E-08
CDCP1	rs683486	11	126356768	A	C	0.40	-0.13	0.01	1.68E-36
CDCP1	rs11048473	12	26340881	T	C	0.27	0.06	0.01	1.19E-07
CDCP1	rs35350651	12	111469627	C	A	0.50	-0.05	0.01	1.16E-06
CDCP1	rs7485656	12	124831101	G	A	0.14	0.08	0.01	6.60E-09
CDCP1	rs186021206	17	7166093	A	G	0.01	0.56	0.04	3.42E-17
CDCP1	rs3747207	22	43928975	A	G	0.22	0.06	0.01	1.53E-07

CDH1	rs2519093	9	133266456	T	C	0.18	-0.35	0.01	1.62E-133
CDH1	rs10749609	10	80489996	A	G	0.79	0.07	0.01	8.92E-07
CDH1	rs186021206	17	7166093	A	G	0.01	0.44	0.05	7.49E-09
CDH1	rs708686	19	5840608	T	C	0.27	0.09	0.01	3.43E-13
CDH1	rs3814995	19	35851310	T	C	0.31	0.06	0.01	1.36E-06
CDH1	rs681343	19	48703205	T	C	0.51	-0.43	0.01	0.00E+00
CDH15	rs10923344	1	117554006	A	G	0.62	0.04	0.01	5.75E-06
CDH15	-	2	203358071	T	G	0.57	-0.04	0.01	1.52E-05
CDH15	rs34707604	4	68625738	C	T	0.26	-0.05	0.01	4.87E-06
CDH15	-	10	5204247	A	C	0.14	0.06	0.01	1.85E-05
CDH15	rs11646135	16	89132700	A	G	0.14	0.44	0.01	2.59E-214
CDH15	rs2240715	22	19973083	C	G	0.30	0.06	0.01	4.12E-09
CDH17	rs12084158	1	120065830	G	A	0.10	0.09	0.01	8.94E-07
CDH17	rs533137864	1	212828526	A	C	0.15	-0.10	0.01	2.68E-12
CDH17	rs55898964	2	107023243	G	A	0.29	0.15	0.01	2.77E-43
CDH17	rs9815073	3	188397894	A	C	0.34	-0.06	0.01	2.15E-08
CDH17	rs3128921	6	33102972	A	C	0.26	0.09	0.01	3.82E-15
CDH17	rs4556017	7	100989509	T	C	0.85	-0.12	0.01	3.59E-17
CDH17	rs763526069	8	94191597	A	C	0.47	-0.27	0.01	2.73E-156
CDH17	rs550057	9	133271182	T	C	0.26	-0.45	0.01	0.00E+00
CDH17	rs56278466	10	17833858	G	T	0.66	0.05	0.01	1.89E-05
CDH17	rs12801491	11	126476973	A	G	0.20	0.06	0.01	2.96E-06
CDH17	rs2280381	16	85985027	T	C	0.61	-0.05	0.01	8.35E-07
CDH17	rs186021206	17	7166093	A	G	0.01	0.55	0.04	7.83E-16
CDH17	rs708686	19	5840608	T	C	0.27	0.15	0.01	3.14E-39
CDH17	rs679574	19	48702851	G	C	0.51	-0.38	0.01	8.79E-299
CDH2	rs772351164	2	113909621	G	T	0.08	-0.11	0.01	1.05E-07
CDH2	rs4074793	5	52897294	G	A	0.07	0.12	0.01	9.31E-09
CDH2	rs17054431	5	157295418	T	C	0.05	-0.19	0.02	1.07E-15
CDH2	rs1800562	6	26092913	A	G	0.08	0.09	0.01	9.67E-06
CDH2	rs112875651	8	125494452	A	G	0.39	-0.06	0.01	1.83E-07
CDH2	rs181242111	10	17823665	A	G	0.12	0.09	0.01	2.57E-07
CDH2	rs9633740	10	80505515	G	A	0.80	0.11	0.01	1.10E-16
CDH2	rs7108216	11	702097	C	T	0.60	0.05	0.01	1.21E-05
CDH2	rs11045834	12	21188162	T	C	0.28	-0.05	0.01	1.72E-05
CDH2	rs28929474	14	94378610	T	C	0.02	0.28	0.02	7.38E-14
CDH2	rs116698525	16	528190	T	C	0.11	-0.14	0.01	2.61E-18
CDH2	rs11869771	17	9683405	C	A	0.24	0.08	0.01	6.27E-12
CDH2	rs11657440	17	81513660	C	T	0.42	0.05	0.01	1.48E-05
CDH2	rs11872284	18	28100459	T	C	0.31	-0.10	0.01	2.08E-19
CDH3	rs12137270	1	116721714	T	C	0.24	0.06	0.01	1.81E-06
CDH3	rs61747728	1	179557079	T	C	0.04	0.15	0.02	1.04E-06
CDH3	rs1260326	2	27508073	C	T	0.61	0.06	0.01	1.85E-06
CDH3	rs4934189	10	80455036	T	C	0.54	0.09	0.01	8.07E-15
CDH3	rs4997081	16	20353912	C	G	0.19	-0.07	0.01	6.96E-07
CDH3	rs138190335	16	68685216	C	T	0.00	0.75	0.06	1.08E-16
CDH3	rs3814995	19	35851310	T	C	0.31	0.07	0.01	5.25E-08
CDH5	rs61802301	1	161605359	C	T	0.57	0.05	0.01	1.06E-06
CDH5	rs1260326	2	27508073	C	T	0.61	0.06	0.01	7.97E-08
CDH5	rs13107325	4	102267552	T	C	0.08	0.12	0.01	4.65E-10
CDH5	rs10093	6	32641396	G	C	0.38	0.06	0.01	7.90E-08
CDH5	rs8176743	9	133256028	T	C	0.06	1.20	0.02	0.00E+00
CDH5	rs35166255	11	126431861	A	G	0.03	0.29	0.02	3.84E-25
CDH5	rs3184504	12	111446804	C	T	0.52	-0.04	0.01	2.27E-05
CDH5	rs16956504	16	66398478	C	T	0.10	0.31	0.01	6.25E-75
CDH5	rs186021206	17	7166093	A	G	0.01	0.58	0.05	4.68E-16

CDH5	rs3814995	19	35851310	T	C	0.31	0.05	0.01	2.73E-06
CDH5	rs660745	19	48716202	C	T	0.54	-0.05	0.01	1.07E-06
CDH5	rs5757670	22	39433731	A	G	0.69	-0.06	0.01	5.20E-07
CDH6	rs4626350	5	30839344	A	G	0.56	0.51	0.01	0.00E+00
CDH6	rs6993770	8	105569300	T	A	0.29	0.07	0.01	1.97E-10
CDH6	rs60757417	9	132989049	G	C	0.06	0.09	0.01	2.01E-05
CDH6	rs11553699	12	121779004	G	A	0.14	0.07	0.01	2.11E-05
CDH6	rs186021206	17	7166093	A	G	0.01	0.54	0.04	2.27E-15
CDH6	rs4820378	22	39473204	T	C	0.46	0.05	0.01	2.11E-07
CDHR1	rs61816761	1	152313385	A	G	0.02	0.19	0.03	2.28E-06
CDHR1	rs12330170	3	98787654	C	A	0.45	0.08	0.01	8.67E-11
CDHR1	rs73186030	3	122294618	T	C	0.13	0.11	0.01	8.56E-10
CDHR1	rs7916271	10	84194464	T	C	0.50	0.23	0.01	1.42E-84
CDHR2	rs1497406	1	16178825	G	A	0.58	0.05	0.01	5.11E-08
CDHR2	rs4835265	4	145900258	A	C	0.16	0.06	0.01	3.40E-06
CDHR2	rs2963680	5	176515947	T	C	0.50	-0.10	0.01	1.16E-24
CDHR2	rs73226270	12	120951307	T	A	0.07	-0.10	0.01	1.20E-06
CDHR2	rs11621792	14	24402720	T	C	0.45	0.04	0.01	8.89E-06
CDHR2	rs28929474	14	94378610	T	C	0.02	0.15	0.02	1.29E-05
CDHR2	rs61462345	14	103098598	G	C	0.24	0.09	0.01	6.44E-15
CDHR2	-	15	60595685	G	A	0.62	0.06	0.01	5.19E-10
CDHR2	rs429358	19	44908684	C	T	0.16	-0.06	0.01	1.75E-05
CDHR2	-	22	17981286	A	T	0.26	-0.05	0.01	2.60E-06
CDHR5	rs5993	1	197061699	T	A	0.16	0.25	0.01	7.47E-76
CDHR5	rs35457250	3	186620775	T	C	0.01	0.30	0.04	7.63E-08
CDHR5	rs3130297	6	32231204	T	C	0.13	-0.07	0.01	5.47E-06
CDHR5	-	7	28215112	A	T	0.09	0.10	0.01	4.34E-08
CDHR5	rs12360820	11	620927	C	A	0.83	0.69	0.01	0.00E+00
CDHR5	rs61889524	11	32870002	G	A	0.63	-0.05	0.01	2.02E-05
CDHR5	rs174566	11	61824890	G	A	0.35	0.06	0.01	4.87E-06
CDHR5	rs28929474	14	94378610	T	C	0.02	0.22	0.02	6.13E-10
CDHR5	rs8178824	17	66228657	T	C	0.03	0.25	0.02	7.36E-17
CDKN1A	rs1354034	3	56815721	C	T	0.60	-0.08	0.01	4.72E-12
CDKN1A	rs3176345	6	36682788	C	G	0.06	-0.28	0.01	3.45E-33
CDKN1A	rs139141690	7	101856650	A	G	0.00	-0.43	0.05	5.62E-07
CDKN1A	rs6993770	8	105569300	T	A	0.29	-0.06	0.01	4.71E-07
CDKN1A	rs7080386	10	63288546	A	C	0.41	0.06	0.01	1.96E-06
CDKN1A	rs58345103	14	68736322	A	G	0.19	-0.08	0.01	2.12E-07
CDKN2D	rs1354034	3	56815721	C	T	0.60	0.11	0.01	1.18E-21
CDKN2D	rs17622656	5	132485305	A	G	0.39	-0.06	0.01	2.50E-06
CDKN2D	rs342298	7	106733200	T	C	0.45	-0.05	0.01	1.45E-05
CDKN2D	rs6993770	8	105569300	T	A	0.29	-0.08	0.01	5.51E-10
CDKN2D	rs544626513	11	180302	C	G	0.23	0.07	0.01	2.79E-06
CDNF	rs61738953	10	14820083	G	C	0.03	-1.12	0.02	2.75E-270
CDON	rs56278466	10	17833858	G	T	0.66	0.05	0.01	1.62E-05
CDON	rs607980	11	126132788	A	G	0.91	-0.18	0.01	8.80E-21
CDON	rs215224	12	481283	C	T	0.40	0.05	0.01	1.52E-05
CDON	rs186021206	17	7166093	A	G	0.01	0.62	0.05	3.39E-16
CDSN	rs80293268	1	8147519	C	G	0.05	-0.10	0.02	2.22E-05
CDSN	rs72674184	1	58592422	C	T	0.08	-0.09	0.01	8.97E-07
CDSN	rs61816761	1	152313385	A	G	0.02	0.56	0.02	2.83E-61
CDSN	rs7545538	1	209806268	G	C	0.43	-0.04	0.01	1.36E-05
CDSN	rs1436661	3	190304132	G	A	0.59	0.07	0.01	6.98E-13
CDSN	rs75278193	4	7432124	C	A	0.19	-0.07	0.01	1.19E-08
CDSN	rs3095306	6	31095824	A	T	0.61	0.64	0.01	0.00E+00
CDSN	rs7835973	8	6865640	G	A	0.16	0.09	0.01	9.53E-11

CDSN	rs3758294	9	104902534	C	T	0.20	-0.05	0.01	9.27E-06
CDSN	rs17658212	15	40853721	T	C	0.06	-0.26	0.01	1.05E-36
CDSN	rs13329952	16	20355185	C	T	0.19	-0.06	0.01	4.32E-06
CDSN	rs7200183	16	23195287	G	C	0.58	-0.04	0.01	2.24E-05
CDSN	rs562177063	16	31131513	T	C	0.00	-0.84	0.06	2.54E-19
CDSN	rs567144736	16	35196017	A	G	0.00	-0.51	0.07	1.57E-05
CDSN	rs561579427	16	46954564	G	A	0.00	-0.53	0.07	5.53E-06
CDSN	rs35187787	16	68822063	A	G	0.01	-0.37	0.04	4.29E-08
CDSN	rs729988	17	47849091	C	G	0.37	-0.05	0.01	3.77E-07
CDSN	rs761061860	19	38280727	G	C	0.17	-0.19	0.01	4.60E-47
CDSN	rs16981329	19	49594675	C	G	0.23	0.06	0.01	7.62E-07
CDSN	rs6016547	20	41334351	C	A	0.17	-0.06	0.01	2.48E-05
CEACAM1	rs36086195	1	16184399	T	C	0.58	0.05	0.01	3.75E-06
CEACAM1	rs114165349	1	26695422	C	G	0.02	0.17	0.02	5.34E-06
CEACAM1	rs4835265	4	145900258	A	C	0.16	0.08	0.01	1.77E-06
CEACAM1	-	9	133263362	G	A	0.18	-0.25	0.01	1.42E-66
CEACAM1	rs11045856	12	21197755	G	T	0.24	0.07	0.01	2.01E-08
CEACAM1	rs56956502	14	103098707	A	G	0.25	0.06	0.01	1.43E-06
CEACAM1	rs10416089	19	42498217	G	C	0.08	-0.48	0.01	1.98E-126
CEACAM21	rs7137828	12	111494996	T	C	0.52	-0.04	0.00	4.83E-08
CEACAM21	rs186021206	17	7166093	A	G	0.01	0.23	0.03	1.06E-05
CEACAM21	rs140838345	19	41571242	C	A	0.78	-1.26	0.01	0.00E+00
CEACAM3	rs139026664	2	89897441	A	C	0.08	0.18	0.01	1.73E-16
CEACAM3	rs62148538	2	94705474	C	T	0.04	0.18	0.02	4.46E-09
CEACAM5	rs77972916	2	43534973	A	G	0.08	0.09	0.01	1.80E-05
CEACAM5	rs12523278	5	61268080	G	A	0.50	0.05	0.01	2.49E-06
CEACAM5	rs60750824	7	100329813	G	A	0.18	-0.06	0.01	1.04E-05
CEACAM5	rs4242701	9	36267264	G	A	0.43	-0.06	0.01	1.53E-07
CEACAM5	rs375524395	9	106744749	G	A	0.46	-0.10	0.01	1.54E-20
CEACAM5	-	9	133263362	G	A	0.18	-0.21	0.01	3.75E-52
CEACAM5	rs12799864	11	126483589	T	C	0.20	0.08	0.01	1.77E-08
CEACAM5	rs7332827	13	109271066	A	G	0.71	0.05	0.01	9.67E-06
CEACAM5	rs186021206	17	7166093	A	G	0.01	0.35	0.05	5.30E-06
CEACAM5	rs117990138	17	48039100	A	G	0.07	-0.10	0.01	3.66E-06
CEACAM5	rs708686	19	5840608	T	C	0.27	0.12	0.01	5.05E-19
CEACAM5	rs9621	19	41727239	A	G	0.04	0.96	0.02	4.82E-258
CEACAM5	rs681343	19	48703205	T	C	0.51	0.30	0.01	6.43E-155
CEACAM5	rs432938	21	41334846	G	A	0.21	0.10	0.01	9.61E-14
CEACAM8	rs3917932	1	36478315	G	C	0.58	-0.06	0.01	1.14E-06
CEACAM8	rs112694524	2	43226582	A	G	0.07	0.10	0.01	8.17E-06
CEACAM8	rs2236947	3	50334001	A	C	0.48	0.09	0.01	1.84E-15
CEACAM8	rs3774298	3	187742311	A	G	0.69	0.07	0.01	1.74E-08
CEACAM8	rs12675298	8	6971291	A	G	0.02	0.21	0.03	3.18E-07
CEACAM8	rs35440906	8	60733792	A	C	0.19	0.21	0.01	2.30E-49
CEACAM8	rs1058900	11	308290	C	T	0.57	0.06	0.01	5.55E-07
CEACAM8	rs2511241	11	73234296	T	C	0.93	0.16	0.01	8.85E-14
CEACAM8	rs6503533	17	40028327	T	C	0.62	-0.11	0.01	1.38E-21
CEACAM8	rs114641762	17	44339796	A	C	0.10	-0.25	0.01	6.10E-40
CEACAM8	rs119468010	17	58272835	A	G	0.00	0.46	0.06	1.07E-06
CEACAM8	rs12459454	19	41759235	G	A	0.29	-0.13	0.01	1.75E-26
CEBPB	rs1045929	17	40019173	T	C	0.39	0.07	0.01	7.43E-09
CEBPB	rs10404947	19	1081618	G	A	0.80	0.07	0.01	1.38E-06
CEBPB	rs4811031	20	50340417	G	A	0.35	-0.05	0.01	1.18E-05
CELA3A	rs34262568	1	21986878	A	G	0.23	0.23	0.01	1.72E-72
CELA3A	rs333947	1	109928142	A	G	0.15	-0.07	0.01	2.13E-06
CELA3A	rs17032925	2	67039351	C	T	0.08	-0.10	0.01	2.59E-06

CELA3A	rs3822288	4	109933388	T	C	0.34	-0.05	0.01	1.89E-05
CELA3A	rs35479797	6	32568123	T	C	0.53	-0.06	0.01	6.60E-06
CELA3A	rs7744847	6	96086797	T	G	0.39	0.13	0.01	3.94E-32
CELA3A	rs112166936	6	126411994	C	A	0.45	-0.07	0.01	3.37E-11
CELA3A	rs11778310	8	18917392	G	A	0.29	0.07	0.01	6.75E-09
CELA3A	rs8176747	9	133255928	G	C	0.06	0.50	0.01	8.17E-108
CELA3A	rs56278466	10	17833858	G	T	0.66	0.06	0.01	4.44E-07
CELA3A	rs174578	11	61838027	A	T	0.35	0.05	0.01	2.82E-05
CELA3A	rs74997273	11	100718017	T	A	0.27	-0.07	0.01	3.75E-08
CELA3A	rs6575449	14	94630966	T	C	0.18	-0.11	0.01	1.20E-14
CELA3A	rs28365941	16	283138	C	T	0.14	-0.08	0.01	4.59E-07
CELA3A	rs72802342	16	75200974	A	C	0.08	-0.26	0.01	1.75E-34
CELA3A	rs1673931	16	88910069	C	T	0.38	-0.10	0.01	4.18E-18
CELA3A	rs681343	19	48703205	T	C	0.51	0.20	0.01	4.09E-74
CEP20	rs1354034	3	56815721	C	T	0.60	0.10	0.01	2.30E-17
CEP20	rs342298	7	106733200	T	C	0.46	-0.06	0.01	2.00E-06
CEP20	rs741735	11	256976	A	C	0.24	0.06	0.01	2.81E-06
CEP20	rs74009487	16	15872040	T	C	0.06	-0.22	0.02	4.75E-18
CEP20	rs200309755	17	29518756	T	C	0.54	0.06	0.01	5.16E-06
CEP43	rs56279106	1	180134769	A	G	0.28	0.07	0.01	7.85E-08
CEP43	rs3811444	1	247876149	T	C	0.33	-0.05	0.01	1.73E-05
CEP43	rs2237276	6	167028627	T	C	0.45	0.07	0.01	1.30E-08
CEP43	rs342293	7	106731773	G	C	0.46	-0.06	0.01	2.08E-07
CEP43	rs6993770	8	105569300	T	A	0.29	-0.06	0.01	1.65E-05
CEP43	rs11170874	12	54332691	G	A	0.33	0.06	0.01	5.19E-07
CEP85	-	1	26219616	A	T	0.94	0.14	0.02	1.46E-09
CEP85	rs1354034	3	56815721	C	T	0.60	0.12	0.01	6.52E-25
CEP85	rs342293	7	106731773	G	C	0.46	-0.06	0.01	7.09E-08
CEP85	rs544626513	11	180302	C	G	0.23	0.07	0.01	4.98E-06
CERT	rs1354034	3	56815721	C	T	0.60	0.09	0.01	2.66E-13
CERT	rs10942737	5	75386988	C	T	0.08	0.10	0.01	1.12E-06
CERT	rs342298	7	106733200	T	C	0.45	-0.05	0.01	7.36E-06
CERT	rs7297265	12	54325905	G	A	0.44	0.05	0.01	6.43E-06
CES1	rs913257	1	170552235	A	G	0.46	0.05	0.01	1.47E-05
CES1	rs12144137	1	224467152	T	C	0.04	0.12	0.02	1.55E-05
CES1	-	2	20952479	T	G	0.22	0.08	0.01	7.59E-10
CES1	rs1260326	2	27508073	C	T	0.61	-0.09	0.01	6.00E-19
CES1	rs7640956	3	57648408	G	A	0.82	-0.12	0.01	2.20E-19
CES1	rs114082534	4	118813029	A	G	0.06	0.13	0.01	9.53E-09
CES1	rs13240994	7	73602532	C	T	0.20	-0.07	0.01	8.83E-07
CES1	rs75231243	7	127482218	A	G	0.07	-0.14	0.01	1.01E-10
CES1	rs199922514	8	9325592	G	A	0.91	-0.16	0.01	1.83E-19
CES1	rs112875651	8	125494452	A	G	0.39	-0.06	0.01	1.51E-07
CES1	rs72775768	9	136430122	T	C	0.29	-0.06	0.01	7.54E-08
CES1	rs2393969	10	63380680	C	A	0.47	-0.06	0.01	5.65E-09
CES1	rs10845139	12	7427058	A	G	0.09	-0.08	0.01	5.44E-06
CES1	rs7979473	12	120982457	G	A	0.62	0.06	0.01	2.91E-07
CES1	rs8018720	14	39086981	C	G	0.82	-0.06	0.01	1.46E-05
CES1	rs147233090	15	43735849	T	C	0.02	0.15	0.02	2.40E-05
CES1	rs143168962	16	55815161	G	T	0.06	-1.14	0.02	0.00E+00
CES1	rs58542926	19	19268740	T	C	0.08	0.18	0.01	3.72E-20
CES1	rs7256200	19	44912678	T	G	0.13	-0.10	0.01	1.03E-09
CES1	rs8736	19	54173495	T	C	0.44	-0.05	0.01	3.61E-06
CES2	rs4704834	5	157016055	G	A	0.65	0.05	0.01	1.88E-05
CES2	-	9	133263362	G	A	0.18	-0.07	0.01	5.58E-06
CES2	rs140461033	16	66938061	A	G	0.00	-1.07	0.06	6.51E-31

CES3	rs114165349	1	26695422	C	G	0.02	0.22	0.02	6.50E-09
CES3	rs4340676	3	57596361	G	A	0.82	-0.13	0.01	4.30E-20
CES3	rs73015965	6	160706469	G	A	0.01	0.59	0.05	5.36E-15
CES3	rs17715183	7	127555143	C	G	0.07	-0.15	0.01	3.04E-11
CES3	rs9987289	8	9325848	G	A	0.91	-0.18	0.01	3.86E-21
CES3	rs4933698	10	91881345	A	C	0.53	-0.05	0.01	4.88E-06
CES3	rs7970695	12	120985573	A	G	0.62	0.08	0.01	4.29E-11
CES3	rs71647892	16	66963853	C	T	0.01	-0.85	0.03	1.42E-63
CES3	rs11668882	19	54171328	C	T	0.44	-0.06	0.01	2.61E-07
CETN2	rs1354034	3	56815721	C	T	0.60	0.09	0.01	4.93E-13
CETN2	rs342296	7	106732457	A	G	0.46	-0.06	0.01	7.09E-08
CETN2	rs117564216	22	31553941	A	G	0.02	-0.25	0.03	9.33E-08
CFC1	rs11708067	3	123346931	G	A	0.24	0.07	0.01	2.71E-07
CFC1	rs6790951	3	160307499	T	C	0.48	0.06	0.01	8.32E-07
CFC1	rs9265969	6	31347929	A	G	0.16	-0.08	0.01	1.95E-06
CFC1	rs35510806	6	126357185	A	T	0.47	-0.05	0.01	2.09E-05
CFC1	rs2168101	11	8233861	A	C	0.31	0.06	0.01	1.17E-06
CFC1	rs4502156	15	62090956	C	T	0.44	0.07	0.01	9.65E-09
CFC1	rs28461437	16	373813	G	C	0.60	-0.10	0.01	1.22E-16
CFC1	rs34931250	17	68883786	T	C	0.06	0.11	0.02	1.17E-05
CGA	rs9388486	6	126340008	C	T	0.45	0.04	0.01	4.95E-06
CGREF1	rs2384572	2	27101883	T	G	0.62	-0.56	0.01	0.00E+00
CGREF1	rs183030488	6	32491390	C	T	0.82	0.08	0.01	1.85E-08
CGREF1	rs782134971	9	133264504	G	C	0.25	-0.25	0.01	3.73E-103
CGREF1	rs12961734	18	59416739	A	G	0.61	-0.06	0.01	3.56E-08
CGREF1	rs492602	19	48703160	G	A	0.51	-0.28	0.01	9.41E-167
CHAC2	rs78694551	2	53751676	G	A	0.27	-0.15	0.01	1.65E-31
CHAC2	rs1671152	19	55014977	G	T	0.84	0.07	0.01	1.60E-05
CHGB	rs2509902	2	157658457	C	T	0.86	-0.08	0.01	3.93E-07
CHGB	rs13159918	5	158465724	A	C	0.30	-0.05	0.01	8.11E-06
CHGB	rs386705092	6	116937001	G	C	0.32	-0.08	0.01	6.13E-12
CHGB	rs11154902	6	136980161	T	C	0.46	-0.09	0.01	4.45E-18
CHGB	rs17092104	8	20148043	C	G	0.11	0.13	0.01	4.36E-15
CHGB	rs28688991	16	20356228	T	C	0.19	-0.06	0.01	1.83E-06
CHGB	rs236153	20	5923248	G	A	0.37	-0.61	0.01	0.00E+00
CHI3L1	rs4950928	1	203186754	C	G	0.79	0.67	0.01	0.00E+00
CHI3L1	rs11127048	2	27529596	A	G	0.62	-0.06	0.01	8.51E-08
CHI3L1	rs1047891	2	210675783	A	C	0.31	0.05	0.01	7.83E-06
CHI3L1	rs28601761	8	125487789	G	C	0.42	-0.12	0.01	1.70E-31
CHI3L1	rs2464190	12	120977587	C	T	0.41	0.05	0.01	5.76E-07
CHI3L1	rs339969	15	60591082	A	C	0.62	-0.06	0.01	9.34E-08
CHI3L1	rs62117298	19	53414810	C	T	0.48	-0.06	0.01	3.41E-09
CHIT1	rs872583	1	203215638	C	T	0.20	-1.09	0.01	0.00E+00
CHIT1	rs9272672	6	32641072	C	T	0.32	-0.05	0.01	1.19E-07
CHIT1	rs149291347	6	122457804	T	C	0.54	-0.04	0.01	2.54E-05
CHIT1	rs763913092	10	62733504	G	T	0.41	0.10	0.01	7.88E-32
CHIT1	rs11048473	12	26340881	T	C	0.27	0.04	0.01	1.69E-05
CHIT1	rs11057840	12	124831509	C	A	0.14	0.08	0.01	3.07E-11
CHIT1	rs34743976	15	85068586	A	T	0.28	-0.05	0.01	1.78E-07
CHL1	-	1	43812246	G	A	0.29	0.05	0.01	1.40E-05
CHL1	rs931679	3	217842	A	T	0.09	0.53	0.01	5.75E-157
CHL1	rs16846347	3	98777624	G	A	0.16	0.12	0.01	4.01E-13
CHL1	rs78689694	11	126364925	C	G	0.13	-0.09	0.01	3.69E-08
CHL1	rs1800574	12	120979061	T	C	0.03	0.26	0.02	3.59E-15
CHL1	rs186021206	17	7166093	A	G	0.01	0.72	0.05	6.12E-22
CHL1	rs8105767	19	22032639	G	A	0.29	-0.06	0.01	6.51E-06

CHMP1A	rs1354034	3	56815721	C	T	0.60	0.05	0.01	2.08E-05
CHMP1A	rs7080386	10	63288546	A	C	0.41	0.06	0.01	3.63E-06
CHMP1A	rs460879	16	89646481	T	C	0.43	-0.09	0.01	6.29E-16
CHMP1A	rs892090	19	55027704	G	T	0.83	0.07	0.01	2.57E-05
CHRDL1	rs215225	12	481379	G	A	0.43	0.05	0.01	1.28E-05
CHRDL1	rs777834943	16	20355409	T	C	0.18	-0.07	0.01	3.91E-07
CHRDL1	rs186021206	17	7166093	A	G	0.01	0.43	0.05	1.54E-09
CHRDL1	rs1065853	19	44909976	T	G	0.08	0.11	0.01	1.29E-08
CHRDL1	rs2899319	22	39478309	C	A	0.62	0.06	0.01	1.75E-07
CHRDL2	rs9815534	3	186924486	G	C	0.29	0.06	0.01	4.17E-06
CHRDL2	rs574672574	11	74721142	A	G	0.04	-0.65	0.02	3.93E-124
CHRDL2	rs6590199	11	126365772	A	G	0.16	0.08	0.01	8.80E-08
CHRDL2	rs138903858	14	105168150	T	G	0.03	0.19	0.02	6.42E-08
CHRDL2	rs200489612	17	7203059	A	G	0.01	0.65	0.05	5.21E-16
CIAPIN1	rs1354034	3	56815721	C	T	0.60	0.07	0.01	1.23E-09
CIAPIN1	rs342298	7	106733200	T	C	0.46	-0.05	0.01	8.12E-06
CIAPIN1	rs10733789	10	63188924	C	T	0.31	0.05	0.01	1.55E-05
CIAPIN1	rs686402	16	57452701	T	C	0.53	0.06	0.01	1.84E-06
CKAP4	rs61747728	1	179557079	T	C	0.04	0.14	0.02	1.00E-06
CKAP4	rs2704895	12	106256210	A	G	0.22	0.22	0.01	6.70E-64
CKAP4	rs28929474	14	94378610	T	C	0.02	1.32	0.03	1.64E-256
CKAP4	rs28640218	16	20347945	T	G	0.19	-0.07	0.01	3.14E-06
CKAP4	-	17	66382324	C	G	0.03	0.15	0.02	1.94E-05
CKMT1A_CKMT1B	rs700882	2	101114935	T	C	0.15	0.07	0.01	1.05E-05
CKMT1A_CKMT1B	rs10205067	2	112031804	G	A	0.07	-0.18	0.01	2.67E-16
CKMT1A_CKMT1B	rs34248556	4	68488960	C	T	0.66	-0.07	0.01	2.30E-08
CKMT1A_CKMT1B	rs73082019	7	24717024	G	A	0.10	-0.09	0.01	1.33E-06
CKMT1A_CKMT1B	rs6472539	8	70717575	C	G	0.52	-0.08	0.01	1.34E-11
CKMT1A_CKMT1B	rs13292932	9	133270009	A	C	0.31	0.07	0.01	1.86E-07
CKMT1A_CKMT1B	-	12	108287518	A	G	0.01	0.55	0.05	4.01E-14
CKMT1A_CKMT1B	rs690604	15	43542666	T	G	0.08	-0.29	0.01	2.84E-40
CKMT1A_CKMT1B	rs601338	19	48703417	A	G	0.51	-0.09	0.01	9.34E-14
CLC	rs1186706	2	61446259	T	G	0.49	-0.05	0.01	1.61E-05
CLC	rs548495874	2	102356207	T	C	0.47	0.06	0.01	4.07E-07
CLC	rs4328821	3	128597592	G	A	0.11	-0.15	0.01	2.85E-16
CLC	rs11242115	5	132490721	C	G	0.39	-0.06	0.01	2.41E-08
CLC	rs448277	6	29934029	T	C	0.29	-0.06	0.01	1.33E-06
CLC	rs7809058	7	75833202	T	C	0.90	-0.19	0.01	1.43E-24
CLC	rs7819099	8	143918694	G	A	0.43	-0.07	0.01	6.31E-10
CLC	-	9	6152128	C	A	0.32	0.06	0.01	2.87E-07
CLC	rs35350651	12	111469627	C	A	0.50	-0.05	0.01	7.29E-06
CLC	rs34210653	17	4632019	A	G	0.02	-0.18	0.03	7.62E-06
CLC	rs143121234	19	39735890	G	C	0.00	-1.36	0.09	2.02E-22
CLC	rs34436714	19	53824059	A	C	0.21	0.14	0.01	9.88E-24
CLEC10A	-	3	161125948	T	A	0.47	0.05	0.01	4.20E-06
CLEC10A	rs781651672	4	144124141	T	G	0.45	0.17	0.01	5.70E-62
CLEC10A	rs9271300	6	32613805	G	C	0.54	0.07	0.01	6.64E-12
CLEC10A	rs971481	7	7203402	G	C	0.61	-0.12	0.01	1.30E-29
CLEC10A	rs13294683	9	79664852	T	C	0.10	0.20	0.01	1.83E-32
CLEC10A	rs2519093	9	133266456	T	C	0.18	0.13	0.01	3.22E-22
CLEC10A	rs12771902	10	70242743	A	G	0.33	-0.06	0.01	7.38E-07
CLEC10A	rs215223	12	481093	A	G	0.40	-0.09	0.01	1.65E-16
CLEC10A	rs3184504	12	111446804	C	T	0.52	-0.06	0.01	1.03E-08
CLEC10A	-	15	56986436	G	A	0.07	0.12	0.01	2.00E-09
CLEC10A	rs56156922	16	56953457	C	T	0.33	0.06	0.01	1.57E-07
CLEC10A	rs90951	17	7078078	G	A	0.33	0.54	0.01	0.00E+00

CLEC10A	-	17	59849030	G	T	0.45	-0.09	0.01	1.46E-16
CLEC10A	rs769669239	22	42719513	C	T	0.47	-0.06	0.01	5.39E-10
CLEC11A	rs753568788	1	205210435	A	T	0.45	-0.05	0.01	2.35E-05
CLEC11A	rs13063578	3	47046347	A	T	0.40	-0.07	0.01	1.03E-09
CLEC11A	rs62525616	8	129605834	T	C	0.57	-0.06	0.01	5.54E-07
CLEC11A	rs2282683	9	76502835	G	A	0.21	-0.07	0.01	1.74E-07
CLEC11A	rs4981022	12	103756096	A	G	0.68	0.07	0.01	1.05E-08
CLEC11A	rs12050709	15	50067027	C	T	0.24	-0.06	0.01	8.00E-06
CLEC11A	rs2179768	16	490500	A	G	0.51	-0.07	0.01	6.05E-09
CLEC11A	rs141369989	19	50723250	A	G	0.03	0.81	0.02	4.67E-126
CLEC11A	rs13036464	20	17685222	C	G	0.41	0.05	0.01	1.12E-05
CLEC14A	rs6664906	1	30881966	C	T	0.46	-0.10	0.01	4.16E-20
CLEC14A	rs61747728	1	179557079	T	C	0.04	0.17	0.02	3.92E-09
CLEC14A	rs75166367	2	162107791	A	G	0.06	-0.11	0.01	2.48E-06
CLEC14A	rs13107325	4	102267552	T	C	0.08	0.16	0.01	3.84E-14
CLEC14A	rs10093	6	32641396	G	C	0.38	0.05	0.01	2.60E-05
CLEC14A	rs507666	9	133273983	A	G	0.18	-0.34	0.01	2.04E-131
CLEC14A	rs4842659	12	89503973	A	G	0.71	-0.09	0.01	6.73E-14
CLEC14A	-	14	38496626	T	A	0.41	0.08	0.01	5.22E-13
CLEC14A	rs777834943	16	20355409	T	C	0.18	-0.08	0.01	7.98E-08
CLEC14A	rs186021206	17	7166093	A	G	0.01	0.43	0.05	7.75E-09
CLEC14A	rs71354106	19	35838485	A	G	0.07	0.16	0.01	1.06E-13
CLEC1A	rs61747728	1	179557079	T	C	0.04	0.12	0.02	2.69E-05
CLEC1A	rs7669607	4	9996177	C	T	0.79	0.06	0.01	3.17E-06
CLEC1A	rs10982156	9	114325784	A	T	0.07	0.23	0.01	5.09E-24
CLEC1A	rs10901252	9	133252613	C	G	0.06	0.78	0.02	3.25E-253
CLEC1A	rs7961483	12	10077861	T	C	0.62	-0.31	0.01	6.63E-171
CLEC1A	rs77924615	16	20381010	A	G	0.20	-0.07	0.01	7.42E-07
CLEC1A	rs186021206	17	7166093	A	G	0.01	0.45	0.05	1.32E-09
CLEC1B	rs3804749	3	123114156	T	C	0.59	-0.08	0.01	1.29E-11
CLEC1B	rs13108400	4	101873456	G	A	0.40	-0.05	0.01	2.70E-05
CLEC1B	rs78947501	4	153538803	C	G	0.03	0.15	0.02	1.04E-05
CLEC1B	rs17622656	5	132485305	A	G	0.39	-0.07	0.01	3.89E-08
CLEC1B	rs6961069	7	80589645	T	C	0.40	0.06	0.01	1.27E-06
CLEC1B	rs6993770	8	105569300	T	A	0.29	-0.13	0.01	8.51E-25
CLEC1B	rs7896518	10	63344740	G	A	0.42	0.09	0.01	5.37E-15
CLEC1B	rs521040	12	9995251	C	T	0.25	0.17	0.01	1.42E-37
CLEC1B	rs1654425	19	55027612	C	T	0.83	0.11	0.01	1.68E-12
CLEC4A	rs200688856	1	161629989	C	G	0.34	0.13	0.01	4.80E-30
CLEC4A	-	3	196784237	T	A	0.50	-0.05	0.01	2.67E-05
CLEC4A	rs791357	5	173778222	C	T	0.72	-0.06	0.01	2.15E-06
CLEC4A	rs1634791	6	31309000	G	A	0.54	0.07	0.01	3.03E-10
CLEC4A	rs327	8	19962025	G	T	0.27	0.05	0.01	1.22E-05
CLEC4A	rs11775560	8	60747604	G	A	0.19	0.07	0.01	1.19E-07
CLEC4A	rs1253387	10	97273211	A	G	0.32	-0.05	0.01	4.88E-06
CLEC4A	rs11603123	11	126435600	A	G	0.03	0.47	0.02	8.36E-58
CLEC4A	rs117213717	12	8125599	A	G	0.01	-1.99	0.03	0.00E+00
CLEC4A	rs10774624	12	111395984	A	G	0.51	0.06	0.01	3.38E-08
CLEC4A	rs174418	15	58395404	C	T	0.60	-0.09	0.01	4.14E-15
CLEC4A	rs247617	16	56956804	A	C	0.32	0.06	0.01	2.48E-08
CLEC4A	rs777152658	17	40044878	C	A	0.40	-0.05	0.01	1.45E-05
CLEC4A	rs6507939	18	49649891	C	A	0.85	0.07	0.01	1.30E-05
CLEC4A	rs4760	19	43648948	G	A	0.16	-0.11	0.01	2.03E-13
CLEC4C	rs7573683	2	60353959	C	T	0.54	-0.05	0.01	8.19E-09
CLEC4C	rs906579	2	65374464	A	T	0.45	0.07	0.01	9.91E-14
CLEC4C	rs11899659	2	98747327	G	A	0.41	0.04	0.01	2.19E-06

CLEC4C	rs1439287	2	111114320	A	G	0.48	0.10	0.01	2.26E-27
CLEC4C	rs7595770	2	144703297	T	G	0.08	-0.11	0.01	4.91E-10
CLEC4C	rs7658518	4	83239126	A	G	0.28	-0.08	0.01	8.12E-15
CLEC4C	rs1490589	4	105024619	G	A	0.66	0.12	0.01	3.59E-35
CLEC4C	rs11559051	5	9549829	G	C	0.10	-0.08	0.01	8.16E-07
CLEC4C	rs4713570	6	32658263	T	C	0.27	0.06	0.01	3.40E-10
CLEC4C	rs74551305	6	45315926	C	T	0.06	0.09	0.01	9.26E-06
CLEC4C	rs2347784	7	6485212	G	C	0.27	-0.07	0.01	6.55E-13
CLEC4C	rs876038	7	50268931	T	C	0.32	-0.21	0.01	1.38E-104
CLEC4C	rs10107630	8	129591389	T	C	0.57	0.06	0.01	5.67E-12
CLEC4C	rs10971417	9	33121482	T	C	0.25	-0.06	0.01	4.07E-08
CLEC4C	rs10441775	9	91719626	G	A	0.69	0.05	0.01	2.31E-06
CLEC4C	rs7873862	9	113351116	T	C	0.19	0.08	0.01	5.49E-12
CLEC4C	rs11789604	9	136704677	G	T	0.45	0.04	0.01	5.99E-06
CLEC4C	rs10838702	11	47389337	T	G	0.39	0.06	0.01	3.09E-09
CLEC4C	rs7129402	11	121341047	A	C	0.72	-0.14	0.01	1.58E-41
CLEC4C	rs11055602	12	7751515	G	T	0.41	0.85	0.01	0.00E+00
CLEC4C	-	12	31494219	C	T	0.69	0.07	0.01	2.06E-08
CLEC4C	rs76428106	13	28029870	C	T	0.01	0.51	0.03	2.43E-35
CLEC4C	-	16	71613731	A	T	0.84	0.06	0.01	2.00E-06
CLEC4C	rs7202624	16	85982569	C	G	0.81	-0.05	0.01	2.69E-05
CLEC4C	rs62045817	16	88978825	T	C	0.15	-0.08	0.01	4.20E-10
CLEC4C	rs62057151	17	45826476	T	C	0.23	-0.05	0.01	8.23E-07
CLEC4C	rs4968607	17	64054970	A	G	0.16	-0.07	0.01	5.80E-08
CLEC4C	rs9624326	22	23792856	T	C	0.16	0.05	0.01	2.72E-05
CLEC4D	rs3917932	1	36478315	G	C	0.58	-0.05	0.01	9.00E-06
CLEC4D	rs2070901	1	161215268	T	G	0.27	-0.20	0.01	6.13E-68
CLEC4D	rs6429432	1	235943941	C	A	0.89	-0.09	0.01	1.20E-07
CLEC4D	rs149110519	6	144064640	T	C	0.04	0.14	0.02	3.88E-07
CLEC4D	rs2158799	7	28237488	G	C	0.61	0.05	0.01	2.60E-05
CLEC4D	rs445	7	92779056	T	C	0.10	-0.08	0.01	6.59E-06
CLEC4D	rs2511241	11	73234296	T	C	0.93	0.18	0.01	1.95E-19
CLEC4D	rs4242896	12	8536403	C	A	0.57	0.53	0.01	0.00E+00
CLEC4D	rs3826331	17	39994239	C	T	0.60	-0.12	0.01	1.42E-29
CLEC4D	rs872629	19	51627385	A	C	0.11	-0.15	0.01	5.14E-20
CLEC4G	rs10935473	3	98698056	T	G	0.44	-0.11	0.01	1.35E-21
CLEC4G	rs35762167	3	186937196	C	T	0.58	0.05	0.01	2.25E-05
CLEC4G	rs371581946	6	29937723	A	G	0.42	0.10	0.01	2.65E-17
CLEC4G	rs7865362	9	33117967	T	C	0.36	0.07	0.01	3.76E-08
CLEC4G	rs505922	9	133273813	C	T	0.32	0.29	0.01	8.07E-133
CLEC4G	-	9	137109392	A	G	0.37	0.08	0.01	6.00E-11
CLEC4G	rs56278466	10	17833858	G	T	0.66	-0.08	0.01	1.72E-12
CLEC4G	rs35426769	10	69485186	A	G	0.00	0.65	0.10	1.76E-05
CLEC4G	rs10830278	11	89483257	A	T	0.80	0.08	0.01	1.45E-09
CLEC4G	rs4760254	12	57372609	C	G	0.24	-0.06	0.01	4.29E-06
CLEC4G	rs12709103	16	88646374	G	A	0.68	-0.05	0.01	6.74E-06
CLEC4G	rs17701598	17	68324304	A	G	0.25	-0.06	0.01	3.26E-06
CLEC4G	rs76560987	19	7734566	T	C	0.07	0.37	0.01	8.62E-66
CLEC5A	rs3917932	1	36478315	G	C	0.58	-0.05	0.01	1.32E-05
CLEC5A	rs10801568	1	196822751	G	T	0.25	-0.07	0.01	6.23E-08
CLEC5A	rs13107325	4	102267552	T	C	0.08	0.09	0.01	7.49E-06
CLEC5A	rs9273113	6	32644878	G	A	0.33	-0.07	0.01	5.88E-08
CLEC5A	rs6932623	6	87231349	T	C	0.82	-0.06	0.01	2.09E-05
CLEC5A	rs11769630	7	50218107	A	T	0.07	0.13	0.01	7.87E-10
CLEC5A	rs6962383	7	141961650	C	A	0.30	0.28	0.01	3.20E-125
CLEC5A	rs10838702	11	47389337	T	G	0.39	-0.06	0.01	3.36E-07

CLEC5A	rs261334	15	58434545	C	G	0.79	-0.07	0.01	1.22E-07
CLEC5A	rs186021206	17	7166093	A	G	0.01	0.44	0.05	2.71E-09
CLEC5A	rs12450688	17	39987976	A	G	0.54	-0.07	0.01	1.70E-10
CLEC5A	rs2254448	20	1570451	G	C	0.78	0.17	0.01	2.98E-42
CLEC6A	rs188468174	1	24965206	T	C	0.01	0.22	0.03	2.40E-06
CLEC6A	rs2070901	1	161215268	T	G	0.27	-0.12	0.01	1.58E-23
CLEC6A	rs10186921	2	43417417	T	C	0.57	-0.05	0.01	9.15E-07
CLEC6A	rs9880192	3	128578726	C	G	0.41	-0.05	0.01	7.75E-06
CLEC6A	rs2158799	7	28237488	G	C	0.61	0.05	0.01	1.31E-05
CLEC6A	rs13277237	8	129592317	A	G	0.57	-0.05	0.01	9.26E-06
CLEC6A	rs11045427	12	8453927	A	C	0.21	-0.64	0.01	0.00E+00
CLEC6A	rs3184504	12	111446804	C	T	0.52	-0.09	0.01	1.63E-15
CLEC6A	rs76428106	13	28029870	C	T	0.01	0.28	0.03	1.55E-08
CLEC6A	rs2239630	14	23120140	G	A	0.56	-0.05	0.01	1.33E-05
CLEC6A	rs28885693	15	50986216	A	G	0.46	0.08	0.01	5.92E-15
CLEC6A	rs4795412	17	39994351	T	C	0.39	0.06	0.01	1.22E-07
CLEC6A	rs872629	19	51627385	A	C	0.11	-0.13	0.01	4.89E-15
CLEC7A	rs2647011	6	32694138	A	C	0.54	-0.04	0.01	2.60E-05
CLEC7A	rs16910526	12	10118488	C	A	0.09	-1.56	0.01	0.00E+00
CLEC7A	rs3184504	12	111446804	C	T	0.52	-0.07	0.01	9.20E-11
CLEC7A	rs1045929	17	40019173	T	C	0.39	0.05	0.01	4.00E-07
CLIP2	rs117471007	7	74282158	T	C	0.06	0.25	0.02	2.93E-26
CLIP2	rs6993770	8	105569300	T	A	0.29	-0.07	0.01	1.19E-07
CLIP2	-	10	63156270	A	T	0.49	0.05	0.01	2.39E-05
CLMP	rs1260326	2	27508073	C	T	0.61	0.05	0.01	1.58E-06
CLMP	rs73158188	7	151718147	T	C	0.28	0.05	0.01	2.48E-05
CLMP	rs73017877	11	123192354	G	C	0.55	0.33	0.01	6.35E-221
CLMP	rs77924615	16	20381010	A	G	0.20	-0.08	0.01	4.27E-09
CLPS	rs376333472	2	240682394	A	G	0.02	0.19	0.02	1.06E-08
CLPS	rs2766588	6	35785450	C	T	0.61	0.55	0.01	0.00E+00
CLPS	rs138435015	7	50964917	G	C	0.96	0.13	0.02	4.82E-07
CLPS	rs2653414	8	17868560	A	C	0.01	0.25	0.03	2.08E-08
CLPS	rs7015116	8	128527736	G	T	0.14	-0.07	0.01	8.53E-07
CLPS	rs7068821	10	88291278	T	G	0.26	-0.05	0.01	3.70E-06
CLPS	rs2286779	10	116635040	C	G	0.52	-0.05	0.01	1.02E-05
CLPS	rs9652674	16	75221575	A	G	0.69	0.09	0.01	5.62E-17
CLPS	rs35866622	19	48714803	T	C	0.50	0.05	0.01	6.11E-07
CLSPN	rs1008982	10	129647467	C	T	0.37	-0.10	0.01	1.55E-17
CLSTN1	rs782134971	9	133264504	G	C	0.25	-0.06	0.01	1.01E-05
CLSTN2	-	2	20170879	C	A	0.48	-0.05	0.01	5.41E-06
CLSTN2	rs41265459	3	140448648	A	T	0.01	2.27	0.04	7.00E-241
CLSTN2	rs6984305	8	9320758	T	A	0.89	-0.10	0.01	4.22E-10
CLSTN2	rs77542162	17	69085137	G	A	0.02	0.20	0.02	9.45E-09
CLSTN2	rs329721	19	53097169	T	C	0.34	-0.09	0.01	8.12E-14
CLTA	rs3811444	1	247876149	T	C	0.33	-0.12	0.01	2.00E-22
CLUL1	rs34881159	1	161620713	A	T	0.49	0.04	0.01	1.00E-05
CLUL1	rs17482753	8	19975135	T	G	0.10	0.08	0.01	9.27E-07
CLUL1	rs174553	11	61807686	G	A	0.34	-0.05	0.01	1.70E-06
CLUL1	-	11	116752497	T	A	0.86	0.07	0.01	1.10E-06
CLUL1	rs3751198	12	103753429	G	A	0.60	0.05	0.01	6.84E-07
CLUL1	rs1601935	15	58379566	T	G	0.66	-0.05	0.01	1.71E-06
CLUL1	rs12149545	16	56959249	A	G	0.32	0.07	0.01	2.06E-13
CLUL1	rs186021206	17	7166093	A	G	0.01	0.34	0.04	6.16E-08
CLUL1	rs12957470	18	591690	C	G	0.29	0.61	0.01	0.00E+00
CLUL1	rs4465830	20	45956781	G	A	0.19	-0.12	0.01	3.08E-23
CNDP1	rs645040	3	136207780	T	G	0.77	0.11	0.01	1.65E-17

CNDP1	rs1030431	8	58399138	G	A	0.67	-0.06	0.01	1.92E-06
CNDP1	rs28601761	8	125487789	G	C	0.42	-0.05	0.01	1.36E-05
CNDP1	rs139974673	15	43735687	C	T	0.02	-0.21	0.02	2.11E-09
CNDP1	rs17817077	18	74542308	A	G	0.39	0.29	0.01	3.95E-142
CNDP1	rs8102710	19	23687308	T	G	0.25	-0.36	0.01	2.14E-173
CNDP1	rs12609475	19	27279853	A	C	0.17	-0.24	0.01	2.65E-58
CNDP1	rs2294915	22	43945024	T	C	0.23	-0.06	0.01	4.73E-06
CNPY2	rs28929474	14	94378610	T	C	0.02	0.48	0.03	2.08E-31
CNPY4	rs1354034	3	56815721	C	T	0.60	0.05	0.01	2.00E-05
CNPY4	rs7457787	7	100180246	C	A	0.12	-0.27	0.01	1.55E-50
CNPY4	rs342293	7	106731773	G	C	0.46	-0.05	0.01	5.52E-06
CNST	rs1354034	3	56815721	C	T	0.60	0.05	0.01	1.08E-05
CNST	rs6993770	8	105569300	T	A	0.29	-0.05	0.01	2.14E-05
CNST	rs7080386	10	63288546	A	C	0.41	0.05	0.01	5.03E-06
CNST	rs1671152	19	55014977	G	T	0.84	0.07	0.01	1.88E-05
CNTN1	rs11755689	6	32618599	G	A	0.33	0.05	0.01	2.42E-05
CNTN1	rs1814170	7	139109403	T	A	0.10	-0.15	0.01	2.92E-19
CNTN1	rs56278466	10	17833858	G	T	0.66	0.06	0.01	3.30E-08
CNTN1	rs184621187	12	37785040	A	G	0.02	0.16	0.02	2.57E-05
CNTN1	rs11177623	12	40653549	C	T	0.38	0.20	0.01	1.11E-75
CNTN1	rs28929474	14	94378610	T	C	0.02	0.15	0.02	2.83E-05
CNTN1	rs2071916	16	376476	C	T	0.59	-0.30	0.01	6.96E-182
CNTN1	rs186021206	17	7166093	A	G	0.01	0.48	0.04	5.14E-12
CNTN1	rs573431210	17	69280242	G	T	0.03	0.14	0.02	1.60E-05
CNTN2	rs2071533	1	205043070	G	T	0.87	-1.17	0.01	0.00E+00
CNTN2	rs1294406	6	6737504	G	A	0.61	-0.08	0.01	1.88E-15
CNTN2	-	9	133276163	T	A	0.19	-0.07	0.01	2.44E-09
CNTN2	rs56278466	10	17833858	G	T	0.66	0.10	0.01	2.95E-25
CNTN2	rs74612335	11	126368738	C	T	0.13	-0.08	0.01	2.86E-08
CNTN2	rs141826184	12	57184132	T	C	0.00	0.31	0.04	1.08E-05
CNTN2	rs186021206	17	7166093	A	G	0.01	0.45	0.04	2.69E-12
CNTN3	rs114165349	1	26695422	C	G	0.02	0.26	0.02	2.10E-14
CNTN3	rs1063412	1	172441827	A	G	0.56	0.05	0.01	2.77E-07
CNTN3	rs1260326	2	27508073	C	T	0.61	-0.08	0.01	7.18E-16
CNTN3	rs4677414	3	74557869	G	A	0.93	-0.51	0.01	6.08E-138
CNTN3	rs1154988	3	136206349	A	T	0.77	-0.08	0.01	5.21E-11
CNTN3	rs9270475	6	32591230	T	A	0.25	-0.05	0.01	2.49E-05
CNTN3	rs9471975	6	42951484	C	T	0.58	-0.05	0.01	9.44E-06
CNTN3	rs33951980	7	73615107	T	C	0.13	-0.12	0.01	3.05E-14
CNTN3	rs4921914	8	18414928	T	C	0.78	0.07	0.01	4.83E-09
CNTN3	-	10	5214017	A	T	0.16	0.06	0.01	1.60E-05
CNTN3	-	10	63464698	C	A	0.52	-0.06	0.01	4.22E-08
CNTN3	rs11023881	11	16225154	A	T	0.39	0.05	0.01	1.30E-06
CNTN3	rs36124182	12	24062000	G	A	0.04	0.13	0.02	4.63E-06
CNTN3	rs139974673	15	43735687	C	T	0.02	0.30	0.02	2.40E-19
CNTN3	rs144268704	15	63500022	G	A	0.34	0.06	0.01	2.89E-07
CNTN3	rs540	16	370907	C	T	0.60	-0.07	0.01	3.70E-12
CNTN3	rs11647695	16	80745434	C	A	0.55	0.06	0.01	9.97E-09
CNTN3	rs34088055	16	88469262	T	C	0.32	0.05	0.01	6.81E-06
CNTN3	rs34893138	17	59773402	T	A	0.21	0.06	0.01	1.51E-06
CNTN3	rs77542162	17	69085137	G	A	0.02	0.20	0.02	1.12E-08
CNTN4	rs163352	3	3056357	C	G	0.81	-0.31	0.01	2.90E-106
CNTN4	rs9860747	3	186869628	C	T	0.84	-0.07	0.01	3.90E-06
CNTN4	rs13133886	4	16034172	C	A	0.78	-0.08	0.01	1.59E-09
CNTN4	rs9270487	6	32591299	G	A	0.34	-0.05	0.01	8.04E-06
CNTN4	rs9987289	8	9325848	G	A	0.91	-0.09	0.01	2.15E-06

CNTN4	rs174528	11	61776027	C	T	0.38	0.05	0.01	2.47E-06
CNTN4	rs4055121	11	126362442	T	C	0.14	-0.18	0.01	2.97E-33
CNTN4	rs7138520	12	482012	G	C	0.50	-0.05	0.01	4.85E-06
CNTN4	rs186021206	17	7166093	A	G	0.01	0.90	0.05	1.75E-36
CNTN4	rs2899319	22	39478309	C	A	0.62	0.05	0.01	5.55E-06
CNTN5	rs780094	2	27518370	C	T	0.62	0.04	0.01	5.23E-06
CNTN5	rs13133886	4	16034172	C	A	0.78	-0.13	0.01	2.32E-29
CNTN5	rs7769413	6	32574282	C	T	0.80	0.07	0.01	2.93E-07
CNTN5	rs10993170	9	94346258	C	G	0.04	0.23	0.02	1.03E-22
CNTN5	rs72767003	9	125150878	T	C	0.15	-0.06	0.01	2.20E-06
CNTN5	rs898776	11	99218310	A	G	0.92	-0.79	0.01	0.00E+00
CNTN5	rs186021206	17	7166093	A	G	0.01	0.43	0.04	3.36E-11
CNTN5	rs134077	22	27667264	G	A	0.56	0.07	0.01	2.70E-13
CNTNAP2	rs4665972	2	27375230	C	T	0.61	0.05	0.01	2.40E-05
CNTNAP2	rs7810370	7	145668909	G	A	0.71	-0.55	0.01	0.00E+00
CNTNAP2	-	9	133274293	A	C	0.18	-0.08	0.01	4.36E-08
CNTNAP2	rs56278466	10	17833858	G	T	0.66	0.12	0.01	1.09E-29
CNTNAP2	rs1033415	22	39492769	G	A	0.65	0.06	0.01	7.47E-08
COL18A1	rs780094	2	27518370	C	T	0.62	-0.06	0.01	2.89E-08
COL18A1	rs28817415	4	76480299	T	C	0.46	0.06	0.01	3.79E-08
COL18A1	-	15	60595685	G	A	0.62	0.05	0.01	2.50E-05
COL18A1	rs116539064	17	79022318	T	C	0.10	0.11	0.01	5.47E-09
COL18A1	rs9976834	21	45477300	T	C	0.18	-0.20	0.01	7.89E-45
COL1A1	rs2476601	1	113834946	G	A	0.90	0.08	0.01	1.46E-05
COL1A1	rs1386625	4	38359499	G	A	0.90	-0.13	0.01	1.20E-11
COL1A1	rs1233396	6	29579022	A	G	0.14	-0.07	0.01	1.59E-05
COL1A1	rs10955915	8	118951308	G	A	0.54	0.10	0.01	5.44E-18
COL1A1	-	9	133274293	A	C	0.18	-0.07	0.01	2.93E-06
COL1A1	rs58973023	13	42384997	T	A	0.49	0.13	0.01	4.79E-30
COL1A1	rs12879725	14	74961545	T	C	0.49	-0.05	0.01	2.06E-06
COL1A1	rs117068593	14	92651884	T	C	0.19	-0.07	0.01	3.62E-07
COL1A1	rs2663344	17	1023119	C	A	0.31	0.05	0.01	2.66E-05
COL1A1	rs147266928	17	50185845	C	T	0.00	1.39	0.11	8.28E-17
COL1A1	rs146385050	17	62559897	A	C	0.18	0.13	0.01	1.04E-19
COL1A1	rs884205	18	62387624	C	A	0.75	-0.06	0.01	1.76E-06
COL4A1	rs2498323	4	3449382	A	G	0.10	-0.14	0.01	4.17E-15
COL4A1	rs72793280	5	132227207	T	C	0.46	0.05	0.01	6.44E-06
COL4A1	rs35493868	7	73625076	G	C	0.20	0.07	0.01	1.36E-07
COL4A1	rs28600710	8	54524233	A	G	0.21	0.08	0.01	1.63E-09
COL4A1	-	11	47263606	C	T	0.15	0.09	0.01	2.43E-07
COL4A1	rs2229357	12	57449928	A	G	0.25	0.07	0.01	1.41E-07
COL4A1	rs652572	13	110167110	G	A	0.66	0.18	0.01	1.03E-54
COL4A1	rs116539064	17	79022318	T	C	0.10	0.19	0.01	9.84E-24
COL4A1	rs12483377	21	45511195	A	G	0.09	0.41	0.01	1.50E-95
COL4A1	rs738408	22	43928850	T	C	0.22	0.07	0.01	7.98E-08
COL6A3	rs1050785	2	237324109	A	C	0.60	0.09	0.01	4.76E-16
COL6A3	rs77924615	16	20381010	A	G	0.20	-0.06	0.01	1.22E-05
COL6A3	rs186021206	17	7166093	A	G	0.01	0.40	0.05	2.23E-07
COL6A3	rs3088026	21	46129699	T	C	0.09	-0.11	0.01	3.05E-08
COL9A1	rs1800562	6	26092913	A	G	0.08	0.11	0.01	9.74E-08
COL9A1	rs543674622	6	70277423	G	A	0.00	1.53	0.08	2.12E-40
COL9A1	rs34072915	10	4930921	G	T	0.12	-0.10	0.01	3.97E-09
COL9A1	rs11831278	12	23807795	T	C	0.16	0.09	0.01	6.65E-09
COLEC12	rs10265221	7	151717243	C	T	0.29	0.05	0.01	9.02E-06
COLEC12	rs11606287	11	47385888	A	C	0.36	-0.05	0.01	1.69E-06
COLEC12	rs34262842	16	20344489	G	A	0.17	-0.07	0.01	3.99E-07

COLEC12	rs149622251	18	331717	T	C	0.01	-0.86	0.05	9.12E-36
COMP	rs4233367	1	161193247	C	T	0.61	-0.07	0.01	4.46E-09
COMP	rs56278466	10	17833858	G	T	0.66	0.12	0.01	2.15E-26
COMP	rs60648773	11	126367386	A	G	0.23	-0.07	0.01	4.37E-07
COMP	rs7310615	12	111427245	G	C	0.52	0.05	0.01	1.85E-05
COMP	rs186021206	17	7166093	A	G	0.01	0.99	0.05	6.98E-41
COMP	rs12974746	19	18795739	G	A	0.03	-0.58	0.02	4.09E-69
COMT	rs1354034	3	56815721	C	T	0.60	0.05	0.01	4.89E-06
COMT	rs11502185	11	180258	C	T	0.26	0.06	0.01	2.63E-05
COMT	rs4680	22	19963748	A	G	0.52	-0.45	0.01	0.00E+00
CORO1A	rs342299	7	106733272	T	C	0.46	-0.06	0.01	1.81E-07
CORO1A	rs12292693	11	65169248	C	A	0.26	-0.08	0.01	9.30E-09
CORO1A	rs10432210	18	25281830	T	C	0.21	0.06	0.01	7.70E-06
COX5B	rs112327139	4	48857303	T	C	0.01	-0.67	0.04	1.77E-23
COX5B	rs201327644	4	51863747	T	C	0.01	-0.60	0.05	3.63E-15
COX5B	rs17569603	7	44968902	C	T	0.17	0.07	0.01	1.52E-05
COX5B	rs61915365	12	27725321	G	C	0.17	-0.08	0.01	1.20E-06
COX5B	rs6580980	12	54298277	G	A	0.56	0.05	0.01	2.36E-05
CPA1	rs35004807	1	50642739	A	T	0.34	0.07	0.01	4.33E-08
CPA1	rs2641348	1	119895261	G	A	0.11	-0.10	0.01	9.30E-08
CPA1	rs200296656	4	39400322	T	G	0.18	0.09	0.01	3.96E-09
CPA1	rs10014383	4	109927163	C	T	0.34	-0.05	0.01	7.06E-06
CPA1	rs11724862	4	185651349	C	T	0.24	-0.06	0.01	1.20E-05
CPA1	rs56762787	5	158755183	C	T	0.18	-0.07	0.01	3.38E-06
CPA1	rs11759956	6	7108864	T	C	0.55	0.06	0.01	4.75E-07
CPA1	rs3215532	6	29554341	A	G	0.25	0.07	0.01	1.20E-07
CPA1	rs373980643	6	126378465	G	T	0.46	-0.07	0.01	5.39E-11
CPA1	rs572393412	7	117426325	A	C	0.31	0.07	0.01	1.03E-07
CPA1	rs13226219	7	130379650	C	T	0.13	0.25	0.01	6.60E-52
CPA1	rs4921967	8	18862119	T	G	0.69	-0.07	0.01	4.39E-09
CPA1	rs10901250	9	133246239	A	G	0.12	0.09	0.01	2.59E-07
CPA1	rs56278466	10	17833858	G	T	0.66	0.05	0.01	9.24E-06
CPA1	rs28456	11	61822009	G	A	0.31	0.07	0.01	9.14E-10
CPA1	rs686056	11	100775958	A	G	0.32	-0.09	0.01	6.42E-15
CPA1	rs1492237	12	52878444	C	T	0.66	-0.06	0.01	5.46E-07
CPA1	rs76734539	12	132531969	A	G	0.14	-0.08	0.01	1.85E-07
CPA1	rs72802342	16	75200974	A	C	0.08	-0.37	0.01	1.01E-66
CPA1	rs1673931	16	88910069	C	T	0.38	-0.11	0.01	1.26E-22
CPA1	rs17138478	17	37713312	A	C	0.13	0.09	0.01	2.49E-07
CPA1	rs8088688	18	44814416	T	A	0.08	0.09	0.01	2.66E-05
CPA1	rs1698113	19	48756599	C	T	0.48	0.06	0.01	1.52E-06
CPA1	rs8131986	21	38087224	C	A	0.47	-0.05	0.01	2.68E-06
CPA1	rs80184311	22	37512603	C	G	0.11	0.08	0.01	1.15E-05
CPA2	rs6952090	7	130282658	C	T	0.35	-0.37	0.01	4.23E-209
CPA2	rs56057779	8	10659370	C	G	0.56	0.06	0.01	6.81E-08
CPA2	-	8	75607946	A	T	0.13	-0.07	0.01	1.67E-05
CPA2	-	9	133276163	T	A	0.19	-0.11	0.01	4.31E-15
CPA2	rs56278466	10	17833858	G	T	0.66	0.08	0.01	9.88E-13
CPA2	rs72802342	16	75200974	A	C	0.08	-0.23	0.01	3.75E-28
CPB1	rs333947	1	109928142	A	G	0.15	-0.08	0.01	2.49E-06
CPB1	rs2493413	1	119956951	A	G	0.11	-0.10	0.01	8.12E-08
CPB1	rs17032925	2	67039351	C	T	0.08	-0.10	0.01	2.50E-06
CPB1	rs13318853	3	148844612	A	G	0.24	0.13	0.01	4.21E-22
CPB1	rs35400735	4	39445686	G	T	0.27	-0.06	0.01	1.37E-05
CPB1	rs3215532	6	29554341	A	G	0.25	0.07	0.01	3.01E-07
CPB1	rs9482771	6	127125465	C	G	0.50	0.07	0.01	5.09E-11

CPB1	rs572393412	7	117426325	A	C	0.31	0.07	0.01	1.28E-07
CPB1	rs4921967	8	18862119	T	G	0.69	-0.08	0.01	1.69E-10
CPB1	-	9	133263362	G	A	0.18	-0.09	0.01	3.30E-10
CPB1	rs56278466	10	17833858	G	T	0.66	0.05	0.01	7.56E-06
CPB1	rs174548	11	61803876	G	C	0.31	0.07	0.01	3.97E-09
CPB1	rs686056	11	100775958	A	G	0.32	-0.09	0.01	3.77E-14
CPB1	rs72802342	16	75200974	A	C	0.08	-0.41	0.01	1.27E-80
CPB1	rs1673931	16	88910069	C	T	0.38	-0.16	0.01	7.06E-42
CPB1	rs201566516	21	38187741	T	A	0.45	-0.06	0.01	5.68E-07
CPB1	rs80184311	22	37512603	C	G	0.11	0.08	0.01	8.07E-06
CPE	rs1550270	4	165340648	C	T	0.30	0.26	0.01	5.77E-95
CPE	rs3812316	7	73606007	G	C	0.13	0.08	0.01	3.90E-06
CPE	rs532436	9	133274414	A	G	0.18	-0.08	0.01	1.21E-08
CPE	rs56278466	10	17833858	G	T	0.66	0.06	0.01	1.16E-06
CPE	rs597808	12	111535554	G	A	0.52	0.08	0.01	2.62E-11
CPE	rs2393775	12	120986771	A	G	0.62	0.10	0.01	1.85E-17
CPE	rs9936550	16	75208952	T	C	0.08	-0.12	0.01	4.28E-08
CPM	rs11118611	1	220817131	G	A	0.29	0.09	0.01	1.11E-13
CPM	rs1862069	2	169077231	A	G	0.54	-0.05	0.01	2.77E-06
CPM	-	6	24429112	C	T	0.00	-0.74	0.06	6.26E-18
CPM	rs28601761	8	125487789	G	C	0.42	-0.08	0.01	2.49E-13
CPM	rs56278466	10	17833858	G	T	0.66	0.07	0.01	9.57E-09
CPM	rs10751647	11	306884	C	T	0.40	0.05	0.01	7.07E-06
CPM	rs7978197	12	68932767	T	C	0.01	-0.47	0.03	9.24E-24
CPM	rs174418	15	58395404	C	T	0.60	0.07	0.01	4.33E-11
CPM	rs200293726	16	79720543	T	A	0.31	0.06	0.01	2.38E-06
CPM	rs186021206	17	7166093	A	G	0.01	0.35	0.05	4.14E-06
CPM	rs5112	19	44927023	G	C	0.53	-0.05	0.01	1.16E-05
CPM	rs11668882	19	54171328	C	T	0.44	0.05	0.01	2.04E-06
CPM	rs777993362	20	25476413	G	A	0.44	-0.08	0.01	7.45E-13
CPPED1	rs2865895	16	12797425	G	C	0.17	0.82	0.01	0.00E+00
CPVL	rs710446	3	186742138	C	T	0.41	-0.05	0.01	2.02E-08
CPVL	-	4	186236113	C	A	0.51	-0.08	0.01	2.14E-18
CPVL	rs2545801	5	177414338	C	T	0.74	-0.05	0.01	5.66E-07
CPVL	rs6914950	6	32622587	T	C	0.15	0.51	0.01	0.00E+00
CPVL	rs993632	6	46118520	T	C	0.64	-0.05	0.01	1.48E-06
CPVL	-	6	159986740	C	T	0.33	0.08	0.01	1.97E-13
CPVL	rs9640023	7	6505704	A	G	0.22	0.06	0.01	8.14E-06
CPVL	rs34219043	7	29120988	T	C	0.20	-0.85	0.01	0.00E+00
CPVL	-	9	133263362	G	A	0.18	-0.06	0.01	6.34E-07
CPVL	rs56278466	10	17833858	G	T	0.66	0.06	0.01	1.78E-11
CPVL	rs769611959	10	89252465	G	A	0.30	0.08	0.01	2.48E-13
CPVL	rs4883201	12	8929985	G	A	0.10	0.10	0.01	1.22E-10
CPVL	-	12	101861457	T	A	0.38	0.06	0.01	1.54E-09
CPVL	rs111338191	12	111388673	T	A	0.52	-0.05	0.01	4.23E-07
CPVL	rs145078947	14	93186629	T	G	0.00	1.47	0.06	6.45E-64
CPVL	rs15951	16	5025247	A	G	0.53	-0.09	0.01	2.74E-22
CPVL	rs186021206	17	7166093	A	G	0.01	0.34	0.04	8.75E-08
CPVL	rs74181667	19	12647909	G	A	0.37	0.06	0.01	5.24E-09
CPXM1	rs1434282	1	199041592	T	C	0.73	-0.08	0.01	5.27E-11
CPXM1	rs13412535	2	224010157	A	G	0.23	-0.06	0.01	1.36E-05
CPXM1	rs7618405	3	18209017	A	C	0.20	-0.06	0.01	7.80E-06
CPXM1	rs1354034	3	56815721	C	T	0.60	-0.07	0.01	7.26E-09
CPXM1	rs755492124	4	101856385	T	G	0.39	-0.06	0.01	1.01E-06
CPXM1	-	5	132328823	T	A	0.63	-0.06	0.01	6.46E-08
CPXM1	rs3016013	6	31383465	A	G	0.72	0.09	0.01	2.30E-14

CPXM1	rs6961069	7	80589645	T	C	0.40	0.05	0.01	1.49E-06
CPXM1	rs773148450	7	100433879	T	C	0.18	-0.07	0.01	9.05E-06
CPXM1	rs6993770	8	105569300	T	A	0.29	-0.16	0.01	9.13E-42
CPXM1	rs296849	9	4780007	A	T	0.49	0.05	0.01	6.57E-06
CPXM1	rs61469632	9	132986603	C	T	0.06	-0.12	0.01	3.40E-07
CPXM1	rs10740118	10	63341447	C	G	0.41	0.07	0.01	1.38E-09
CPXM1	rs71474568	10	102589091	G	T	0.34	0.08	0.01	1.13E-12
CPXM1	rs139130389	11	72139110	A	C	0.07	-0.10	0.01	9.28E-06
CPXM1	rs7970830	12	6173147	A	G	0.48	-0.06	0.01	2.40E-07
CPXM1	rs4759076	12	54336088	C	T	0.46	0.05	0.01	6.21E-06
CPXM1	rs59001897	15	64868193	A	T	0.17	0.06	0.01	1.68E-05
CPXM1	rs12445050	16	81837364	T	C	0.14	0.11	0.01	8.81E-12
CPXM1	rs57839578	17	5365042	T	A	0.48	-0.05	0.01	4.90E-06
CPXM1	rs892090	19	55027704	G	T	0.83	0.17	0.01	2.28E-29
CPXM1	rs215545	20	2801369	C	T	0.76	0.36	0.01	3.89E-166
CPXM1	rs4814837	20	19261036	T	C	0.34	0.07	0.01	2.55E-08
CPXM1	rs16989483	20	44908559	C	T	0.27	-0.06	0.01	7.82E-06
CR2	rs4656100	1	86452261	C	T	0.54	0.07	0.01	4.16E-09
CR2	rs61821111	1	207422554	C	T	0.11	-0.28	0.01	8.26E-59
CR2	rs11585945	1	214235667	T	A	0.60	0.07	0.01	5.04E-11
CR2	rs78572881	2	43535820	C	T	0.07	0.09	0.01	2.73E-05
CR2	rs5001409	3	187017902	C	A	0.39	-0.07	0.01	1.71E-09
CR2	-	3	196745119	C	T	0.67	0.06	0.01	3.62E-06
CR2	rs10012161	4	177045626	G	A	0.82	-0.09	0.01	1.99E-10
CR2	rs1394622	5	100625360	T	G	0.48	-0.05	0.01	2.50E-05
CR2	rs1047437	5	123350033	G	C	0.17	-0.07	0.01	5.54E-06
CR2	rs2760984	6	32598552	C	G	0.18	-0.18	0.01	2.45E-37
CR2	rs9460100	6	170273934	C	T	0.55	0.06	0.01	3.41E-07
CR2	rs1528149	7	16067733	T	C	0.35	0.09	0.01	1.75E-15
CR2	rs7798316	7	47586799	T	A	0.43	0.05	0.01	1.07E-05
CR2	-	7	50284571	G	T	0.32	0.07	0.01	9.79E-09
CR2	rs11238349	7	55088378	A	G	0.28	0.08	0.01	4.31E-10
CR2	rs754722538	7	131198555	A	C	0.32	0.05	0.01	8.53E-06
CR2	rs12554848	9	36981825	C	T	0.27	-0.06	0.01	1.51E-06
CR2	rs4055121	11	126362442	T	C	0.13	-0.08	0.01	8.89E-07
CR2	rs114697502	12	94283783	T	C	0.09	0.11	0.01	3.60E-08
CR2	rs10774624	12	111395984	A	G	0.51	-0.07	0.01	2.78E-10
CR2	rs3751437	13	40558943	G	A	0.24	0.06	0.01	6.35E-06
CR2	rs374039502	13	108308037	A	T	0.02	0.23	0.03	2.39E-08
CR2	rs186021206	17	7166093	A	G	0.01	1.02	0.05	7.47E-42
CR2	rs34557412	17	16948873	G	A	0.01	0.80	0.04	5.28E-33
CR2	-	17	83053396	T	G	0.26	-0.09	0.01	5.07E-12
CR2	rs7228151	18	59514462	C	T	0.20	0.09	0.01	4.85E-10
CR2	rs200437269	21	35072303	G	C	0.11	0.09	0.01	2.92E-06
CR2	rs35374520	22	19863626	G	A	0.15	0.07	0.01	1.21E-05
CR2	rs73165110	22	41895852	A	G	0.08	-0.10	0.01	1.43E-06
CRACR2A	rs1354034	3	56815721	C	T	0.60	0.05	0.01	3.43E-06
CRACR2A	rs6993770	8	105569300	T	A	0.29	-0.06	0.01	4.83E-06
CRACR2A	rs12292693	11	65169248	C	A	0.26	-0.06	0.01	2.20E-06
CRACR2A	rs7977865	12	3791389	A	G	0.34	-0.14	0.01	5.07E-31
CRADD	rs1354034	3	56815721	C	T	0.60	0.06	0.01	2.49E-06
CRADD	rs342293	7	106731773	G	C	0.46	-0.05	0.01	7.87E-06
CRADD	rs7080386	10	63288546	A	C	0.41	0.05	0.01	3.76E-06
CRADD	rs56944668	12	93850077	T	C	0.16	-0.10	0.01	1.36E-09
CRADD	rs200309755	17	29518756	T	C	0.54	0.05	0.01	2.10E-05
CREG1	rs7513428	1	167546035	C	T	0.84	-0.25	0.01	1.82E-64

CREG1	rs687339	3	136213517	T	C	0.77	0.07	0.01	5.59E-08
CREG1	rs13107325	4	102267552	T	C	0.08	0.12	0.01	2.64E-08
CREG1	rs77945361	4	153333382	A	G	0.15	-0.07	0.01	8.11E-06
CREG1	rs4594967	6	130045016	G	A	0.68	0.06	0.01	2.45E-07
CREG1	rs12359178	10	17847614	A	G	0.14	0.10	0.01	2.18E-11
CREG1	rs35751397	10	63154256	A	T	0.47	0.05	0.01	1.37E-05
CREG1	rs139130389	11	72139110	A	C	0.07	-0.11	0.01	1.25E-07
CREG1	-	12	101818376	C	T	0.35	-0.35	0.01	2.63E-185
CREG1	rs150641790	13	41113708	G	T	0.07	0.11	0.01	5.02E-07
CREG1	rs145078947	14	93186629	T	G	0.00	1.02	0.07	7.46E-22
CREG1	rs58542926	19	19268740	T	C	0.08	0.21	0.01	2.27E-24
CREG1	rs429358	19	44908684	C	T	0.16	-0.12	0.01	1.70E-15
CREG1	rs3747207	22	43928975	A	G	0.21	0.07	0.01	8.18E-07
CRELD2	rs1296171	1	226871905	G	C	0.48	-0.06	0.01	6.46E-09
CRELD2	rs9852409	3	57637814	A	T	0.82	-0.18	0.01	1.11E-39
CRELD2	rs3804749	3	123114156	T	C	0.59	-0.05	0.01	2.35E-05
CRELD2	rs6796	7	6462736	C	T	0.28	0.08	0.01	4.71E-12
CRELD2	rs3752617	7	127580863	T	G	0.07	-0.15	0.01	1.93E-11
CRELD2	rs9987289	8	9325848	G	A	0.91	-0.17	0.01	9.85E-20
CRELD2	rs10488686	11	32108345	A	G	0.37	0.06	0.01	1.20E-06
CRELD2	-	11	118549526	C	T	0.15	0.08	0.01	6.06E-07
CRELD2	rs1805214	12	103847627	A	G	0.19	-0.07	0.01	4.04E-07
CRELD2	rs79091287	12	132839152	A	G	0.09	-0.09	0.01	5.53E-06
CRELD2	rs1801689	17	66214462	C	A	0.03	0.16	0.02	8.78E-07
CRELD2	rs2659005	17	81244914	T	C	0.44	0.05	0.01	5.94E-06
CRELD2	rs74510325	22	49921734	G	C	0.03	-0.81	0.02	1.84E-158
CRH	rs12740374	1	109274968	T	G	0.22	-0.11	0.01	5.36E-16
CRH	-	1	234916118	A	C	0.06	-0.11	0.02	3.56E-06
CRH	rs10183338	2	110853239	T	G	0.29	-0.06	0.01	6.01E-06
CRH	rs4561605	2	143514581	G	C	0.46	0.07	0.01	2.33E-10
CRH	rs16840585	2	156609810	C	T	0.21	-0.06	0.01	9.33E-06
CRH	rs13017862	2	199584865	C	T	0.18	-0.08	0.01	9.86E-08
CRH	rs13063578	3	47046347	A	T	0.40	-0.05	0.01	1.44E-05
CRH	rs7687767	4	56958766	G	A	0.18	0.08	0.01	8.41E-09
CRH	rs749317121	4	87037992	A	T	0.55	0.05	0.01	2.33E-06
CRH	rs6887216	5	76915470	A	T	0.64	0.06	0.01	2.96E-08
CRH	rs331700	5	125490918	A	G	0.45	0.05	0.01	1.57E-05
CRH	rs12518248	5	173654457	C	T	0.42	0.06	0.01	1.86E-07
CRH	rs9503212	6	2501301	G	A	0.52	0.11	0.01	1.62E-22
CRH	rs9264839	6	31303172	T	C	0.17	-0.10	0.01	4.02E-11
CRH	rs1229027	7	38983676	A	G	0.24	-0.09	0.01	5.66E-12
CRH	rs111632177	7	151234719	A	G	0.11	0.09	0.01	6.91E-08
CRH	rs59104426	8	66260714	A	T	0.03	-0.68	0.02	1.89E-105
CRH	rs4263799	8	71482756	T	C	0.48	0.16	0.01	2.12E-47
CRH	rs7387462	8	99591682	T	C	0.04	0.44	0.02	4.57E-56
CRH	rs5898348	9	74386918	A	G	0.27	0.08	0.01	7.15E-11
CRH	rs59585065	9	105972930	T	G	0.71	0.09	0.01	5.63E-13
CRH	rs941873	10	79379706	A	G	0.46	-0.07	0.01	2.57E-09
CRH	rs12296430	12	6394334	C	G	0.19	-0.18	0.01	9.96E-36
CRH	rs10774624	12	111395984	A	G	0.51	-0.06	0.01	4.16E-07
CRH	rs7192602	16	78312532	A	G	0.30	-0.06	0.01	3.01E-06
CRH	rs7412	19	44908822	T	C	0.08	0.10	0.01	3.68E-06
CRHBP	rs11162598	1	79030698	A	G	0.36	0.05	0.01	5.70E-06
CRHBP	rs6798915	3	194776533	G	C	0.30	0.06	0.01	1.01E-06
CRHBP	rs6896257	5	76914771	A	G	0.64	-0.59	0.01	0.00E+00
CRHBP	rs6924387	6	136761810	G	A	0.41	-0.06	0.01	5.61E-08

CRHBP	rs10104003	8	54509880	T	C	0.21	0.15	0.01	2.26E-31
CRIM1	rs201258494	2	36356199	G	T	0.19	-0.15	0.01	1.37E-27
CRIM1	rs201147962	3	58477962	T	C	0.61	0.06	0.01	1.35E-06
CRIM1	rs6474527	8	39080846	C	G	0.65	-0.10	0.01	4.22E-18
CRIM1	rs11244061	9	133278537	T	C	0.12	-0.10	0.01	2.66E-08
CRIM1	rs3751198	12	103753429	G	A	0.60	0.06	0.01	1.06E-06
CRIM1	-	12	118073854	A	G	0.03	-0.20	0.02	2.20E-09
CRIM1	rs180915454	17	7288706	T	C	0.00	0.40	0.06	1.41E-05
CRIP2	rs705379	7	95324583	A	G	0.48	0.09	0.01	2.64E-16
CRIP2	rs495203	9	133269828	T	C	0.32	0.07	0.01	3.27E-10
CRIP2	rs80324969	14	105451479	C	G	0.17	0.19	0.01	1.04E-34
CRIP2	rs5817082	16	56963437	A	C	0.27	0.06	0.01	8.80E-06
CRISP2	rs2288196	2	151525381	A	G	0.62	-0.07	0.01	1.28E-14
CRISP2	rs35303892	2	207533213	T	A	0.20	-0.08	0.01	2.94E-15
CRISP2	rs3087450	3	127608466	A	G	0.01	-0.16	0.02	1.04E-05
CRISP2	-	3	156685610	C	T	0.05	-0.08	0.01	2.60E-05
CRISP2	rs34687149	5	139617953	A	T	0.05	0.10	0.01	2.36E-08
CRISP2	rs555247	6	49742392	A	G	0.46	0.49	0.01	0.00E+00
CRISP2	rs7761970	6	87112185	T	C	0.52	-0.05	0.01	1.03E-06
CRISP2	rs6460105	7	64042314	C	A	0.70	-0.13	0.01	1.15E-50
CRISP2	rs58248492	8	119413313	T	A	0.22	-0.06	0.01	9.74E-11
CRISP2	rs10993994	10	46046326	C	T	0.61	-0.04	0.01	2.42E-06
CRISP2	rs569982136	10	133230887	T	C	0.03	-0.17	0.02	7.92E-13
CRISP2	rs1755778	14	36224785	G	A	0.33	-0.04	0.01	7.18E-06
CRISP2	rs7166139	15	41836657	C	T	0.54	0.04	0.01	6.40E-06
CRISP2	rs1054284	19	19506144	G	T	0.35	-0.04	0.01	7.48E-07
CRISP2	rs145144275	19	58350442	G	A	0.01	-0.47	0.03	1.86E-27
CRISP2	rs4911257	20	32771768	C	T	0.40	-0.05	0.01	1.48E-08
CRISP2	rs753827512	20	35616633	C	T	0.19	-0.05	0.01	2.34E-05
CRISP2	rs67610389	21	41874711	A	G	0.29	0.04	0.01	1.75E-06
CRKL	rs6993770	8	105569300	T	A	0.29	-0.07	0.01	5.54E-08
CRLF1	rs13402561	2	3592331	G	C	0.82	0.06	0.01	1.22E-05
CRLF1	rs72538440	2	64997954	C	G	0.42	-0.05	0.01	1.36E-05
CRLF1	rs1486236	2	179874723	A	C	0.37	0.05	0.01	1.27E-05
CRLF1	rs13017230	2	226407124	A	G	0.19	-0.07	0.01	5.05E-06
CRLF1	-	5	173336651	T	A	0.39	0.08	0.01	3.03E-10
CRLF1	rs11362821	12	7514128	A	G	0.98	-0.17	0.02	3.94E-06
CRLF1	rs180408	12	46771540	G	C	0.78	0.06	0.01	2.71E-05
CRLF1	rs10774624	12	111395984	A	G	0.51	-0.06	0.01	7.95E-09
CRLF1	rs1126464	16	89637957	C	G	0.24	-0.07	0.01	9.89E-08
CRLF1	rs12975366	19	54255498	C	T	0.40	-0.07	0.01	4.59E-10
CRNN	rs10494275	1	152419091	A	G	0.07	-1.18	0.01	0.00E+00
CRNN	rs28746795	6	32664757	C	T	0.78	0.07	0.01	5.75E-09
CRNN	rs582118	9	133270061	G	A	0.32	0.06	0.01	2.12E-07
CRNN	rs704	17	28367840	A	G	0.47	0.09	0.01	1.16E-19
CRTAC1	rs1042445	3	186677647	T	C	0.22	0.49	0.01	0.00E+00
CRTAC1	rs56007204	10	97865562	T	C	0.21	-0.77	0.01	0.00E+00
CRTAM	rs13401811	2	110858527	A	G	0.18	-0.07	0.01	1.01E-06
CRTAM	rs1047891	2	210675783	A	C	0.31	0.06	0.01	3.81E-07
CRTAM	rs13063578	3	47046347	A	T	0.40	-0.10	0.01	2.80E-20
CRTAM	rs35829610	5	132488170	G	C	0.34	-0.06	0.01	3.06E-07
CRTAM	rs9267084	6	31441828	A	G	0.15	0.13	0.01	7.10E-17
CRTAM	rs2327832	6	137651931	G	A	0.22	0.06	0.01	8.76E-06
CRTAM	rs10982456	9	114928478	C	T	0.43	-0.05	0.01	3.62E-06
CRTAM	rs41290291	10	7732354	C	T	0.01	0.31	0.05	2.15E-05
CRTAM	rs11215416	11	115206607	G	A	0.26	-0.07	0.01	1.87E-07

CRTAM	rs2370794	11	122844074	G	A	0.34	0.31	0.01	2.41E-157
CRTAM	rs3184504	12	111446804	C	T	0.52	-0.09	0.01	4.64E-16
CRTAM	-	16	28610251	G	T	0.34	0.06	0.01	7.66E-07
CRTAM	rs396742	16	89701648	G	C	0.42	0.05	0.01	1.44E-05
CRTAM	rs186021206	17	7166093	A	G	0.01	0.63	0.05	1.64E-17
CRTAM	rs2725405	17	81246424	C	G	0.44	0.05	0.01	7.70E-06
CSF1	rs11577194	1	109957553	C	T	0.52	-0.18	0.01	8.58E-61
CSF1	rs17315646	1	230159560	G	C	0.61	0.06	0.01	7.66E-08
CSF1	rs3132455	6	31520177	C	G	0.35	0.06	0.01	1.09E-06
CSF1	rs149044215	16	89638692	A	G	0.37	0.06	0.01	3.85E-06
CSF2RA	rs200489612	17	7203059	A	G	0.01	0.56	0.06	1.22E-10
CSF3	rs3917932	1	36478315	G	C	0.58	-0.18	0.01	3.69E-54
CSF3	rs56388170	7	28684757	T	G	0.29	-0.06	0.01	5.05E-07
CSF3	rs14408	11	308314	C	T	0.37	-0.07	0.01	8.14E-09
CSF3	rs748908389	17	39973272	T	C	0.47	0.08	0.01	2.14E-11
CST3	rs13146355	4	76490987	A	G	0.46	0.06	0.01	1.49E-09
CST3	rs13230509	7	1246556	C	G	0.69	0.05	0.01	2.80E-05
CST3	rs6464165	7	151716038	C	T	0.28	0.05	0.01	8.50E-06
CST3	rs7137828	12	111494996	T	C	0.52	-0.05	0.01	4.39E-06
CST3	rs77924615	16	20381010	A	G	0.20	-0.06	0.01	2.68E-06
CST3	rs911119	20	23632100	T	C	0.78	0.33	0.01	2.06E-144
CST5	rs72694438	1	103822256	A	G	0.21	-0.05	0.01	9.54E-06
CST5	rs4950775	1	202223295	T	C	0.42	-0.05	0.01	8.93E-08
CST5	rs6540740	1	206965124	T	A	0.45	0.06	0.01	6.72E-10
CST5	rs2576233	1	216818590	T	G	0.24	0.05	0.01	4.12E-06
CST5	rs3820640	1	226681217	C	T	0.16	0.05	0.01	2.63E-05
CST5	rs7428376	3	18816320	C	T	0.23	-0.05	0.01	7.00E-06
CST5	rs2970871	4	23888959	C	T	0.56	0.05	0.01	1.86E-08
CST5	rs4976271	5	135175676	T	G	0.60	-0.07	0.01	3.31E-12
CST5	rs745377	11	34836866	T	G	0.52	0.05	0.01	1.69E-08
CST5	rs11054082	12	10952211	C	G	0.25	-0.18	0.01	4.60E-67
CST5	rs67020211	15	63347445	G	T	0.55	-0.05	0.01	6.30E-09
CST5	-	17	55261443	T	A	0.15	-0.06	0.01	1.20E-05
CST5	rs601338	19	48703417	A	G	0.51	0.10	0.01	1.16E-26
CST5	rs1799841	20	23879541	G	A	0.44	0.73	0.01	0.00E+00
CST5	rs545804263	20	31567324	T	C	0.01	0.33	0.04	1.77E-06
CST6	rs80293268	1	8147519	C	G	0.05	-0.39	0.02	7.24E-50
CST6	rs2990223	1	155215184	G	A	0.61	-0.05	0.01	1.04E-05
CST6	rs28635651	1	218683160	G	A	0.33	0.07	0.01	3.08E-08
CST6	rs1260326	2	27508073	C	T	0.61	0.06	0.01	1.43E-07
CST6	rs13015258	2	162074215	G	T	0.62	0.06	0.01	1.32E-06
CST6	rs121908120	2	218890289	A	T	0.03	-0.19	0.02	2.81E-08
CST6	rs833270	3	181802378	C	T	0.62	0.05	0.01	2.20E-05
CST6	rs776694658	4	74459648	C	A	0.38	0.06	0.01	4.36E-06
CST6	rs75680898	4	106865391	G	A	0.14	0.07	0.01	9.63E-06
CST6	rs114964313	5	79137749	A	G	0.32	0.06	0.01	1.11E-06
CST6	rs155942	5	140152953	C	T	0.70	-0.06	0.01	1.23E-06
CST6	rs7905367	10	52574893	C	G	0.80	0.09	0.01	1.28E-10
CST6	rs4141614	10	62854095	A	C	0.60	-0.05	0.01	2.55E-06
CST6	rs12576095	11	66008834	A	G	0.02	0.59	0.02	9.01E-63
CST6	rs36034702	15	90191395	T	C	0.17	-0.11	0.01	1.50E-13
CST6	rs4997081	16	20353912	C	G	0.19	-0.06	0.01	1.35E-05
CST7	rs114694170	5	88884379	C	T	0.06	0.11	0.01	4.77E-09
CST7	-	6	31448099	T	G	0.30	0.05	0.01	7.08E-07
CST7	rs6993770	8	105569300	T	A	0.29	-0.15	0.01	1.38E-53
CST7	rs774510679	10	63304076	A	C	0.41	-0.06	0.01	2.06E-12

CST7	rs113422568	10	102578181	A	G	0.33	0.09	0.01	7.35E-20
CST7	rs2511241	11	73234296	T	C	0.93	0.09	0.01	5.11E-07
CST7	rs35979828	12	54292096	T	C	0.07	-0.09	0.01	4.53E-07
CST7	rs117566084	12	101796743	T	C	0.01	0.30	0.03	5.54E-11
CST7	rs3184504	12	111446804	C	T	0.52	-0.07	0.01	1.74E-14
CST7	rs145078947	14	93186629	T	G	0.00	0.43	0.06	9.35E-07
CST7	rs729116	16	85382505	G	C	0.88	-0.08	0.01	1.27E-09
CST7	rs7208815	17	40012462	T	C	0.39	0.08	0.01	3.13E-17
CST7	rs227651	20	24949198	G	A	0.23	-0.83	0.01	0.00E+00
CST7	rs574174001	20	30338973	T	C	0.01	1.45	0.05	2.44E-81
CSTB	-	21	43776442	C	G	0.30	-0.81	0.01	0.00E+00
CTF1	rs342298	7	106733200	T	C	0.46	-0.05	0.01	8.01E-06
CTF1	rs774510679	10	63304076	A	C	0.41	0.05	0.01	2.52E-05
CTF1	rs11170874	12	54332691	G	A	0.33	0.05	0.01	2.11E-05
CTF1	-	16	30989405	T	C	0.62	0.05	0.01	1.24E-05
CTRB1	rs2493413	1	119956951	A	G	0.11	-0.07	0.01	2.73E-05
CTRB1	rs11759956	6	7108864	T	C	0.55	0.05	0.01	3.05E-06
CTRB1	rs3132568	6	31134140	G	A	0.26	-0.05	0.01	1.25E-05
CTRB1	rs7766106	6	127133993	T	C	0.50	0.07	0.01	3.90E-12
CTRB1	rs2329566	7	50949958	A	G	0.95	0.15	0.02	3.51E-09
CTRB1	rs6666	7	142752462	C	T	0.58	-0.05	0.01	5.91E-07
CTRB1	rs4921967	8	18862119	T	G	0.69	-0.07	0.01	1.19E-09
CTRB1	rs2519093	9	133266456	T	C	0.18	-0.10	0.01	2.45E-13
CTRB1	rs174549	11	61803910	A	G	0.31	0.07	0.01	4.70E-09
CTRB1	rs61910508	11	100748595	T	G	0.29	-0.06	0.01	1.51E-06
CTRB1	rs8051363	16	75221319	G	A	0.71	0.57	0.01	0.00E+00
CTRB1	rs1126464	16	89637957	C	G	0.24	-0.09	0.01	1.69E-10
CTRB1	rs17138478	17	37713312	A	C	0.13	0.07	0.01	3.36E-06
CTRC	rs497078	1	15440540	T	C	0.10	-0.56	0.01	6.78E-218
CTRC	-	1	50576832	C	T	0.45	0.05	0.01	1.98E-05
CTRC	-	6	31768138	T	A	0.12	-0.09	0.01	6.42E-07
CTRC	rs6571015	6	96086602	A	G	0.79	0.20	0.01	1.01E-55
CTRC	rs112166936	6	126411994	C	A	0.45	-0.08	0.01	2.09E-13
CTRC	rs11778310	8	18917392	G	A	0.29	0.06	0.01	1.28E-07
CTRC	rs8176743	9	133256028	T	C	0.06	0.88	0.01	0.00E+00
CTRC	rs56278466	10	17833858	G	T	0.66	0.06	0.01	2.34E-07
CTRC	rs9581943	13	27919860	A	G	0.40	0.05	0.01	8.48E-06
CTRC	rs35775091	14	20807826	A	G	0.27	0.06	0.01	3.77E-07
CTRC	rs72802342	16	75200974	A	C	0.08	-0.24	0.01	6.64E-32
CTRC	rs529900	16	88910560	C	G	0.38	-0.10	0.01	3.42E-19
CTRC	rs200489612	17	7203059	A	G	0.01	0.52	0.05	2.28E-11
CTRC	rs485186	19	48703949	G	A	0.54	0.11	0.01	6.89E-26
CTRC	-	20	50218801	T	C	0.47	-0.05	0.01	9.12E-06
CTRC	rs530223046	22	28851606	T	A	0.13	-0.08	0.01	2.59E-06
CTSB	rs4713570	6	32658263	T	C	0.27	0.07	0.01	1.25E-08
CTSB	rs709821	8	11845085	C	G	0.26	0.59	0.01	0.00E+00
CTSB	rs10740131	10	63511728	T	A	0.47	0.05	0.01	7.14E-06
CTSB	rs41281340	10	71811862	G	C	0.09	-0.12	0.01	2.63E-11
CTSB	rs10860794	12	101823942	A	C	0.29	-0.09	0.01	3.84E-14
CTSB	rs145078947	14	93186629	T	G	0.00	0.51	0.07	1.27E-06
CTSB	rs8107974	19	19277691	T	A	0.08	0.13	0.01	8.83E-11
CTSC	-	4	99140866	C	A	0.67	-0.05	0.01	1.25E-06
CTSC	rs2854275	6	32660651	A	C	0.15	0.12	0.01	7.18E-19
CTSC	rs9457800	6	159990715	T	C	0.13	-0.07	0.01	1.23E-06
CTSC	rs10822143	10	63128096	T	C	0.50	0.05	0.01	2.29E-06
CTSC	rs11600158	11	88337746	G	A	0.10	0.98	0.01	0.00E+00

CTSC	rs4764823	12	101825988	A	G	0.29	-0.15	0.01	5.78E-46
CTSC	rs145078947	14	93186629	T	G	0.00	0.85	0.06	7.00E-21
CTSC	rs139974673	15	43735687	C	T	0.02	0.13	0.02	2.22E-05
CTSD	rs2298632	1	23383982	T	C	0.50	-0.05	0.01	8.93E-06
CTSD	rs1260326	2	27508073	C	T	0.61	-0.07	0.01	1.74E-09
CTSD	rs55861089	11	1762527	G	A	0.10	-0.50	0.01	4.93E-165
CTSD	rs10778152	12	101831973	G	A	0.29	-0.16	0.01	1.23E-37
CTSD	rs145078947	14	93186629	T	G	0.00	0.52	0.07	2.12E-06
CTSD	rs58542926	19	19268740	T	C	0.08	0.10	0.01	2.32E-06
CTSF	rs143480126	1	117553065	A	C	0.70	0.06	0.01	8.37E-06
CTSF	rs1260326	2	27508073	C	T	0.61	-0.10	0.01	4.84E-20
CTSF	rs370542983	2	241333621	A	T	0.35	-0.06	0.01	1.80E-06
CTSF	rs187325356	4	109345757	A	T	0.01	0.33	0.04	5.32E-07
CTSF	rs6818519	4	118866665	T	G	0.06	-0.17	0.01	1.65E-14
CTSF	rs41280463	4	153270074	A	G	0.17	-0.07	0.01	5.63E-06
CTSF	-	7	28129126	G	T	0.21	0.06	0.01	1.22E-05
CTSF	rs17145750	7	73612048	T	C	0.16	-0.07	0.01	8.65E-07
CTSF	rs2001945	8	125465736	C	G	0.52	-0.05	0.01	4.38E-06
CTSF	rs174567	11	61825533	G	A	0.35	-0.06	0.01	5.48E-07
CTSF	rs1044522	11	66568361	A	G	0.24	0.21	0.01	1.95E-64
CTSF	rs116309149	11	72139545	A	G	0.03	-0.15	0.02	1.83E-05
CTSF	rs10128858	12	101827588	G	A	0.29	-0.28	0.01	6.01E-118
CTSF	rs2393791	12	120986153	T	C	0.62	-0.06	0.01	4.24E-07
CTSF	rs554288187	14	39253644	A	G	0.45	0.05	0.01	2.53E-06
CTSF	rs145078947	14	93186629	T	G	0.00	0.76	0.07	8.58E-13
CTSF	rs139974673	15	43735687	C	T	0.02	0.25	0.02	2.80E-12
CTSF	rs429358	19	44908684	C	T	0.16	-0.08	0.01	6.59E-07
CTSH	rs4764822	12	101822895	T	C	0.29	-0.09	0.01	1.19E-31
CTSH	rs60250730	15	78945802	G	C	0.17	1.15	0.01	0.00E+00
CTSL	rs5030044	3	186731333	G	A	0.11	-0.09	0.01	2.57E-07
CTSL	rs3129938	6	32368718	A	T	0.83	-0.08	0.01	3.43E-08
CTSL	rs9497576	6	146604540	T	G	0.07	0.10	0.01	1.20E-06
CTSL	rs17151689	7	77321369	A	C	0.05	-0.13	0.02	1.30E-06
CTSL	rs2282240	9	27572636	T	C	0.25	0.09	0.01	5.43E-12
CTSL	rs3118869	9	87725948	A	C	0.44	-0.15	0.01	1.06E-40
CTSL	rs150370599	11	60951320	T	C	0.08	0.21	0.01	1.80E-25
CTSL	rs76904798	12	40220632	T	C	0.14	0.11	0.01	1.26E-11
CTSL	rs10846744	12	124827879	C	G	0.14	0.07	0.01	4.47E-06
CTSL	rs9604045	13	113272893	T	G	0.25	0.06	0.01	2.35E-05
CTSL	rs72695000	14	94638408	T	C	0.18	-0.10	0.01	4.97E-13
CTSL	rs10644230	14	103144061	G	A	0.80	0.06	0.01	2.30E-05
CTSL	rs9901675	17	7581494	A	G	0.05	-0.14	0.02	8.09E-08
CTSL	rs77733715	19	3537186	G	A	0.01	0.29	0.04	9.69E-07
CTSL	rs10469365	19	12621060	G	A	0.11	-0.14	0.01	1.38E-15
CTSL	rs4808758	19	18180479	G	A	0.22	0.16	0.01	2.44E-32
CTSL	rs1065853	19	44909976	T	G	0.08	0.10	0.01	2.57E-06
CTSL	rs121434529	22	42061052	T	C	0.01	0.40	0.05	2.93E-07
CTSO	rs583104	1	109278685	T	G	0.77	0.07	0.01	4.98E-08
CTSO	rs687339	3	136213517	T	C	0.77	0.09	0.01	2.77E-11
CTSO	rs2334114	4	155935041	A	C	0.21	-0.22	0.01	6.00E-57
CTSO	rs116105128	6	32543679	C	G	0.12	-0.10	0.01	2.89E-07
CTSO	rs75474551	6	159988862	T	G	0.09	-0.24	0.01	8.80E-38
CTSO	rs56278466	10	17833858	G	T	0.66	0.06	0.01	6.20E-07
CTSO	rs174560	11	61814292	C	T	0.31	0.07	0.01	3.11E-08
CTSO	rs2417888	12	20896816	T	A	0.15	0.13	0.01	2.71E-16
CTSO	-	12	101818376	C	T	0.35	-0.16	0.01	1.49E-43

CTSO	rs146326139	12	123672628	C	G	0.01	-0.23	0.03	3.03E-06
CTSO	rs58542926	19	19268740	T	C	0.08	0.25	0.01	1.42E-33
CTSO	rs738409	22	43928847	G	C	0.22	0.06	0.01	6.61E-06
CTSS	rs771911677	1	150715890	A	T	0.48	-0.44	0.01	5.29E-266
CTSS	rs8191750	6	160025566	G	A	0.14	0.10	0.01	2.36E-09
CTSS	rs545971	9	133267960	T	C	0.32	-0.07	0.01	2.52E-07
CTSS	rs79751758	10	99496620	A	C	0.11	0.09	0.01	1.05E-06
CTSS	rs10896415	11	69071866	A	G	0.36	-0.06	0.01	3.88E-07
CTSS	rs4883201	12	8929985	G	A	0.11	0.16	0.01	1.08E-17
CTSS	rs3184504	12	111446804	C	T	0.52	-0.06	0.01	4.46E-07
CTSS	rs11655264	17	39982742	C	G	0.45	-0.05	0.01	2.23E-05
CTSV	rs13407583	2	113210163	A	G	0.21	0.06	0.01	1.98E-06
CTSV	rs28493806	4	105185552	G	A	0.37	0.05	0.01	1.85E-05
CTSV	rs35716097	5	177379635	T	C	0.30	0.07	0.01	4.21E-10
CTSV	rs260237	6	42424840	T	C	0.11	-0.09	0.01	8.23E-08
CTSV	rs3957356	6	52803872	C	T	0.57	-0.11	0.01	2.19E-26
CTSV	-	7	28430643	A	G	0.37	-0.08	0.01	6.75E-14
CTSV	rs12686511	9	97146762	T	C	0.21	0.25	0.01	8.28E-83
CTSV	rs635634	9	133279427	T	C	0.18	-0.11	0.01	5.03E-16
CTSV	rs7087998	10	58511771	G	A	0.45	0.07	0.01	2.61E-11
CTSV	rs2559850	12	101699681	A	G	0.59	0.12	0.01	2.16E-28
CTSV	rs6490117	12	117161544	G	C	0.45	-0.05	0.01	2.82E-06
CTSV	rs3830659	12	120979733	C	G	0.30	-0.12	0.01	1.60E-24
CTSV	rs340029	15	60602766	T	C	0.62	-0.05	0.01	2.82E-05
CTSV	rs112746916	15	63077855	T	C	0.22	0.06	0.01	4.55E-06
CTSV	rs4077451	16	79897823	A	T	0.85	0.10	0.01	5.11E-11
CTSV	rs114711456	17	37819219	G	A	0.00	-0.34	0.05	2.28E-05
CTSV	rs34582033	17	48634777	C	T	0.17	-0.06	0.01	2.70E-05
CTSV	rs123698	19	807442	C	G	0.61	0.05	0.01	8.87E-06
CTSV	rs12019136	19	5835666	A	G	0.04	0.13	0.02	5.28E-07
CTSV	rs78581838	21	14864259	T	C	0.09	0.09	0.01	6.11E-07
CTSZ	rs41271951	1	150764744	G	A	0.09	-0.11	0.01	1.36E-08
CTSZ	rs61747728	1	179557079	T	C	0.04	0.12	0.02	2.80E-05
CTSZ	rs687339	3	136213517	T	C	0.77	0.08	0.01	4.32E-09
CTSZ	rs3021302	6	32655373	C	T	0.17	0.07	0.01	1.45E-06
CTSZ	-	6	130042457	C	T	0.57	0.06	0.01	7.43E-07
CTSZ	rs9346805	6	160001183	T	C	0.14	-0.07	0.01	1.78E-05
CTSZ	rs149871778	12	8946399	C	G	0.10	0.10	0.01	2.72E-07
CTSZ	rs4764824	12	101826225	G	C	0.29	-0.32	0.01	8.38E-152
CTSZ	rs150641790	13	41113708	G	T	0.07	0.10	0.01	2.71E-06
CTSZ	rs145078947	14	93186629	T	G	0.00	0.78	0.07	3.18E-13
CTSZ	rs200210321	19	19283081	G	A	0.07	0.17	0.01	8.67E-16
CTSZ	rs429358	19	44908684	C	T	0.16	-0.09	0.01	1.27E-08
CTSZ	rs199825459	20	58997654	G	A	0.01	-1.95	0.04	8.72E-240
CX3CL1	rs114921587	1	21345480	A	G	0.10	0.09	0.01	1.49E-06
CX3CL1	rs61747728	1	179557079	T	C	0.04	0.13	0.02	1.40E-05
CX3CL1	rs3732378	3	39265671	A	G	0.17	0.08	0.01	1.52E-08
CX3CL1	rs9264666	6	31271519	C	G	0.55	-0.09	0.01	3.33E-15
CX3CL1	rs785914	9	76485879	C	T	0.74	0.06	0.01	5.25E-06
CX3CL1	rs507666	9	133273983	A	G	0.18	-0.13	0.01	3.71E-18
CX3CL1	rs9414801	10	63389329	A	G	0.53	-0.05	0.01	2.48E-06
CX3CL1	rs781264602	16	57375150	A	G	0.62	-0.23	0.01	6.61E-87
CX3CL1	rs681343	19	48703205	T	C	0.51	0.06	0.01	1.51E-06
CXADR	rs6705147	2	132439353	T	C	0.33	-0.05	0.01	9.60E-06
CXADR	-	9	133263362	G	A	0.18	-0.34	0.01	1.71E-123
CXADR	rs56398830	13	103049340	A	G	0.01	-0.42	0.03	3.79E-15

CXADR	rs12938714	17	30318092	A	G	0.33	0.08	0.01	3.37E-12
CXADR	rs681343	19	48703205	T	C	0.51	-0.18	0.01	7.83E-57
CXADR	rs2739393	21	17562221	A	G	0.67	-0.12	0.01	4.17E-25
CXCL1	rs12075	1	159205564	A	G	0.58	0.06	0.01	6.38E-09
CXCL1	rs3097411	4	73872737	G	C	0.24	0.67	0.01	0.00E+00
CXCL1	rs34077178	6	32582426	A	T	0.22	-0.10	0.01	4.20E-15
CXCL1	rs7080386	10	63288546	A	C	0.41	0.09	0.01	5.14E-17
CXCL1	rs1362213	12	6173262	T	C	0.48	-0.05	0.01	2.81E-05
CXCL1	rs111338191	12	111388673	T	A	0.52	-0.05	0.01	1.51E-05
CXCL10	rs113010081	3	46415921	C	T	0.12	-0.08	0.01	9.28E-06
CXCL10	rs11548618	4	76022794	A	G	0.01	1.72	0.04	1.79E-152
CXCL10	rs9271566	6	32622666	G	A	0.42	-0.07	0.01	9.98E-09
CXCL10	rs67504709	9	32527512	A	T	0.11	-0.08	0.01	2.33E-05
CXCL10	rs3184504	12	111446804	C	T	0.52	-0.10	0.01	7.26E-22
CXCL11	rs76036957	2	112738876	A	G	0.01	0.28	0.04	3.38E-06
CXCL11	rs35170645	4	76043803	G	A	0.51	-0.22	0.01	5.15E-87
CXCL11	rs13181561	5	139471320	A	G	0.74	0.06	0.01	3.20E-06
CXCL11	rs11962818	6	31352399	T	C	0.16	-0.08	0.01	2.24E-07
CXCL11	rs139141690	7	101856650	A	G	0.00	1.15	0.05	1.25E-44
CXCL11	rs7080386	10	63288546	A	C	0.41	0.10	0.01	6.09E-20
CXCL11	rs113422568	10	102578181	A	G	0.33	-0.09	0.01	1.23E-12
CXCL11	rs3184504	12	111446804	C	T	0.52	-0.12	0.01	4.20E-29
CXCL11	rs1654425	19	55027612	C	T	0.83	0.10	0.01	2.23E-11
CXCL12	rs6542680	2	3592552	T	C	0.82	-0.07	0.01	4.35E-06
CXCL12	rs709162	3	12479681	G	T	0.57	0.05	0.01	6.85E-06
CXCL12	-	3	29416540	A	T	0.53	-0.05	0.01	1.12E-05
CXCL12	rs1354034	3	56815721	C	T	0.60	-0.05	0.01	7.25E-06
CXCL12	rs113760175	6	22343363	A	G	0.07	0.13	0.01	1.22E-08
CXCL12	rs3131626	6	31447373	G	A	0.32	0.06	0.01	7.77E-06
CXCL12	rs7028191	9	134225650	T	C	0.44	-0.05	0.01	2.70E-05
CXCL12	rs11239028	10	44387717	T	C	0.34	0.19	0.01	1.63E-56
CXCL12	rs7896518	10	63344740	G	A	0.42	0.07	0.01	4.94E-09
CXCL12	rs3184504	12	111446804	C	T	0.52	-0.05	0.01	2.97E-06
CXCL12	rs1654425	19	55027612	C	T	0.83	0.07	0.01	3.97E-06
CXCL13	rs2053526	4	77504875	G	A	0.40	-0.05	0.01	1.59E-05
CXCL13	rs13204736	6	32614826	A	G	0.35	0.06	0.01	1.47E-06
CXCL13	rs55730499	6	160584578	T	C	0.08	0.11	0.01	3.74E-07
CXCL13	rs10849448	12	6384185	G	A	0.75	-0.06	0.01	1.09E-05
CXCL13	rs111338191	12	111388673	T	A	0.52	-0.08	0.01	5.83E-11
CXCL13	rs34557412	17	16948873	G	A	0.01	0.36	0.05	4.44E-07
CXCL13	rs77542162	17	69085137	G	A	0.02	0.30	0.02	9.33E-15
CXCL13	rs190712692	19	44921921	A	G	0.05	0.14	0.02	7.00E-08
CXCL13	rs2412973	22	30133642	A	C	0.44	-0.07	0.01	2.89E-09
CXCL14	rs4744	1	19978433	A	G	0.23	-0.07	0.01	5.61E-07
CXCL14	rs2078404	5	135623175	A	G	0.36	-0.07	0.01	8.13E-10
CXCL14	rs766802806	6	160714635	C	T	0.29	-0.07	0.01	1.74E-08
CXCL16	rs2009581	2	111050100	A	G	0.27	0.17	0.01	4.07E-45
CXCL16	rs4347833	2	197693098	T	G	0.53	0.05	0.01	2.47E-05
CXCL16	rs79806033	3	46053110	A	G	0.20	-0.12	0.01	4.50E-20
CXCL16	rs7726159	5	1282204	A	C	0.33	-0.05	0.01	2.29E-05
CXCL16	rs117030384	6	32606168	T	A	0.02	-0.29	0.02	7.98E-15
CXCL16	rs748113	10	71749034	C	T	0.43	-0.10	0.01	2.38E-19
CXCL16	rs1250567	10	79286508	C	T	0.45	-0.08	0.01	2.36E-12
CXCL16	rs3184504	12	111446804	C	T	0.52	0.09	0.01	2.23E-16
CXCL16	rs748988	14	99319904	G	A	0.61	0.06	0.01	7.02E-07
CXCL16	rs78086290	17	4738075	T	A	0.53	0.19	0.01	4.01E-65

CXCL16	rs77542162	17	69085137	G	A	0.02	0.30	0.02	1.85E-16
CXCL16	rs875622	19	16356948	G	A	0.77	-0.09	0.01	1.40E-11
CXCL16	rs6031302	20	44027670	G	C	0.73	-0.09	0.01	3.28E-12
CXCL17	rs7592	8	22164370	A	G	0.33	-0.07	0.01	2.33E-11
CXCL17	rs13281991	8	119911334	C	T	0.56	0.05	0.01	1.74E-06
CXCL17	rs34283575	10	79577610	T	C	0.52	0.05	0.01	5.42E-06
CXCL17	rs117753190	17	69140184	G	C	0.04	0.16	0.02	1.81E-09
CXCL17	rs4807612	19	1156854	C	T	0.32	-0.06	0.01	4.15E-09
CXCL17	rs11668599	19	42450984	G	A	0.08	0.12	0.01	3.76E-10
CXCL3	rs12640189	4	6891454	T	C	0.17	0.07	0.01	5.54E-06
CXCL3	rs3104369	6	32634705	C	T	0.77	-0.10	0.01	2.45E-13
CXCL3	rs7896518	10	63344740	G	A	0.42	0.07	0.01	7.87E-09
CXCL5	rs2274319	1	156481081	C	T	0.65	-0.05	0.01	8.78E-06
CXCL5	rs34174712	2	241692164	C	T	0.55	0.05	0.01	4.71E-06
CXCL5	rs11925560	3	47217970	T	A	0.39	0.05	0.01	6.48E-06
CXCL5	rs1354034	3	56815721	C	T	0.60	-0.12	0.01	4.01E-28
CXCL5	rs12640189	4	6891454	T	C	0.17	0.09	0.01	1.06E-08
CXCL5	rs352045	4	73998970	G	T	0.88	-0.59	0.01	1.26E-259
CXCL5	rs6993770	8	105569300	T	A	0.29	0.08	0.01	4.23E-12
CXCL5	rs4631576	9	97942928	T	C	0.36	0.06	0.01	1.75E-08
CXCL5	rs10993918	9	134076423	A	G	0.32	-0.09	0.01	1.72E-14
CXCL5	rs9787438	10	63278270	C	G	0.41	0.21	0.01	7.59E-87
CXCL5	-	10	102591498	C	A	0.44	-0.06	0.01	3.91E-08
CXCL5	rs1654425	19	55027612	C	T	0.83	0.09	0.01	1.12E-09
CXCL6	rs12075	1	159205564	A	G	0.58	0.08	0.01	7.12E-15
CXCL6	rs1260326	2	27508073	C	T	0.61	-0.05	0.01	4.44E-06
CXCL6	rs16858768	2	218161975	C	A	0.00	0.44	0.06	1.06E-05
CXCL6	rs7618405	3	18209017	A	C	0.21	-0.06	0.01	1.11E-05
CXCL6	rs16850073	4	73838282	T	C	0.38	0.56	0.01	0.00E+00
CXCL6	-	9	4767255	C	T	0.22	0.06	0.01	6.84E-06
CXCL6	rs10761737	10	63292445	C	T	0.41	0.07	0.01	6.44E-10
CXCL6	rs111338191	12	111388673	T	A	0.52	-0.06	0.01	2.01E-08
CXCL6	rs892090	19	55027704	G	T	0.83	0.11	0.01	2.01E-13
CXCL8	rs28732243	6	32476311	G	C	0.52	-0.06	0.01	6.82E-07
CXCL8	rs12075	1	159205564	A	G	0.58	0.14	0.01	1.68E-34
CXCL8	rs16858768	2	218161975	C	A	0.00	0.96	0.07	1.54E-20
CXCL8	rs74468557	4	73725686	A	G	0.05	-0.17	0.02	7.74E-12
CXCL8	rs4541868	8	105578477	A	C	0.27	-0.06	0.01	8.30E-07
CXCL8	rs7080386	10	63288546	A	C	0.41	0.09	0.01	4.98E-14
CXCL8	-	10	102591498	C	A	0.44	-0.06	0.01	6.34E-07
CXCL8	rs1058935	11	308363	C	G	0.51	-0.05	0.01	2.04E-05
CXCL8	rs2305482	17	39984674	C	A	0.54	0.08	0.01	2.89E-12
CXCL8	rs892090	19	55027704	G	T	0.83	0.11	0.01	2.41E-12
CXCL9	rs2476601	1	113834946	G	A	0.90	-0.09	0.01	1.35E-07
CXCL9	rs13118503	4	75939317	T	C	0.24	-0.15	0.01	8.04E-33
CXCL9	rs2858864	6	32610452	T	C	0.22	0.07	0.01	5.62E-08
CXCL9	rs4252129	6	160731873	T	C	0.02	-0.30	0.03	2.21E-13
CXCL9	rs3184504	12	111446804	C	T	0.52	-0.11	0.01	3.23E-26
DAB2	rs342292	7	106730198	G	C	0.46	-0.05	0.01	1.58E-05
DAB2	rs6993770	8	105569300	T	A	0.29	-0.07	0.01	1.10E-08
DAB2	rs3847326	10	63572099	G	A	0.48	-0.06	0.01	5.25E-07
DAB2	rs200309755	17	29518756	T	C	0.54	0.05	0.01	1.20E-05
DAG1	-	3	49452394	C	A	0.31	0.06	0.01	2.08E-06
DAG1	rs1354034	3	56815721	C	T	0.60	0.05	0.01	1.66E-05
DAG1	rs409801	9	4744743	C	T	0.51	0.05	0.01	1.50E-05
DAG1	rs71463509	10	63248398	C	G	0.30	-0.06	0.01	1.84E-05

DAG1	rs111338191	12	111388673	T	A	0.52	-0.06	0.01	4.68E-07
DAG1	rs1671152	19	55014977	G	T	0.84	0.07	0.01	4.84E-06
DAPP1	rs1354034	3	56815721	C	T	0.60	0.06	0.01	6.43E-07
DAPP1	rs7688733	4	99831495	G	A	0.48	-0.10	0.01	4.19E-18
DARS1	rs149170955	2	135933930	A	G	0.00	-0.97	0.09	9.87E-13
DARS1	rs78909033	2	240571486	A	G	0.14	-0.10	0.01	7.27E-09
DARS1	rs1354034	3	56815721	C	T	0.60	0.07	0.01	4.65E-09
DARS1	rs342293	7	106731773	G	C	0.46	-0.06	0.01	1.27E-06
DARS1	rs7896518	10	63344740	G	A	0.42	0.05	0.01	1.53E-05
DARS1	rs60822569	12	54323724	C	T	0.55	0.05	0.01	2.10E-05
DBI	rs72833212	2	119378891	G	T	0.20	-0.32	0.01	9.40E-115
DBI	rs1354034	3	56815721	C	T	0.60	0.05	0.01	5.20E-06
DBI	rs17622656	5	132485305	A	G	0.39	-0.05	0.01	1.38E-05
DBI	rs60822569	12	54323724	C	T	0.55	0.05	0.01	1.84E-05
DBNL	rs1354034	3	56815721	C	T	0.60	0.05	0.01	6.53E-06
DBNL	rs7799945	7	44070203	G	A	0.33	0.06	0.01	9.28E-07
DBNL	rs342298	7	106733200	T	C	0.45	-0.05	0.01	3.78E-06
DBNL	rs6993770	8	105569300	T	A	0.29	-0.06	0.01	9.67E-07
DCBLD2	rs602633	1	109278889	G	T	0.78	0.06	0.01	2.79E-05
DCBLD2	rs12487717	3	98886163	A	G	0.01	-1.07	0.03	2.08E-110
DCBLD2	-	3	170961087	G	A	0.29	-0.06	0.01	3.40E-07
DCBLD2	-	4	3477071	C	A	0.40	-0.06	0.01	2.30E-08
DCBLD2	rs149999508	5	75053219	G	C	0.23	-0.07	0.01	2.11E-08
DCBLD2	-	8	58522382	T	C	0.61	0.05	0.01	2.63E-05
DCBLD2	rs28601761	8	125487789	G	C	0.42	-0.06	0.01	3.29E-08
DCBLD2	rs115478735	9	133274295	T	A	0.18	-0.06	0.01	1.15E-05
DCBLD2	rs56278466	10	17833858	G	T	0.66	0.08	0.01	1.08E-13
DCBLD2	rs10822145	10	63174788	T	C	0.47	0.05	0.01	2.60E-05
DCBLD2	rs174581	11	61839211	A	G	0.35	0.05	0.01	3.08E-06
DCBLD2	rs2464190	12	120977587	C	T	0.41	0.05	0.01	1.04E-05
DCBLD2	rs28929474	14	94378610	T	C	0.02	0.50	0.02	9.18E-41
DCBLD2	rs11078597	17	1715069	C	T	0.19	0.07	0.01	1.71E-07
DCBLD2	rs430881	17	9694808	G	T	0.24	0.06	0.01	1.24E-06
DCBLD2	rs77542162	17	69085137	G	A	0.02	-0.20	0.02	3.37E-08
DCBLD2	rs58542926	19	19268740	T	C	0.08	-0.12	0.01	1.01E-08
DCBLD2	rs1672992	19	35065825	T	C	0.86	0.14	0.01	5.43E-21
DCBLD2	rs117486964	19	49478786	G	A	0.05	0.12	0.02	5.78E-06
DCBLD2	rs9980195	21	44848868	C	T	0.53	-0.05	0.01	1.05E-05
DCN	rs1260326	2	27508073	C	T	0.61	0.06	0.01	5.49E-08
DCTN1	rs1339847	1	247875992	A	G	0.11	-0.11	0.01	6.07E-09
DCTN1	rs35505243	2	97646864	T	A	0.08	-0.10	0.01	2.58E-06
DCTN1	-	10	102638182	A	T	0.64	0.06	0.01	2.98E-06
DCTN2	rs1354034	3	56815721	C	T	0.60	0.05	0.01	2.18E-05
DCTN2	rs342293	7	106731773	G	C	0.46	-0.05	0.01	2.02E-05
DCTN6	rs112625444	10	102542139	T	G	0.35	-0.05	0.01	1.53E-05
DCTPP1	rs61801010	1	161572445	G	A	0.11	0.09	0.01	1.13E-06
DCTPP1	rs1354034	3	56815721	C	T	0.60	0.06	0.01	1.25E-06
DCTPP1	rs12342201	9	93132682	A	G	0.48	-0.05	0.01	1.48E-06
DCTPP1	rs56278466	10	17833858	G	T	0.66	0.10	0.01	9.32E-17
DCTPP1	rs3751198	12	103753429	G	A	0.60	0.05	0.01	3.24E-06
DCTPP1	rs146094440	16	30424280	G	C	0.01	-0.51	0.03	3.01E-22
DCXR	-	8	125492141	C	T	0.60	-0.05	0.01	2.82E-05
DCXR	rs10119644	9	114367312	A	T	0.50	-0.07	0.01	2.15E-11
DCXR	rs10883451	10	100164661	C	T	0.50	-0.06	0.01	8.98E-08
DCXR	rs139130389	11	72139110	A	C	0.07	-0.12	0.01	5.00E-09
DCXR	rs4342991	11	94120504	C	G	0.11	-0.10	0.01	7.68E-08

DCXR	rs61746217	17	82036043	T	C	0.00	-0.76	0.06	7.09E-15
DCXR	rs132642	22	36149089	T	A	0.83	0.06	0.01	2.65E-05
DCXR	rs3747207	22	43928975	A	G	0.22	0.08	0.01	2.52E-09
DDAH1	rs12138621	1	85318287	C	T	0.21	-0.18	0.01	5.38E-40
DDAH1	rs1434282	1	199041592	T	C	0.73	0.09	0.01	4.07E-12
DDAH1	rs342293	7	106731773	G	C	0.46	-0.05	0.01	2.53E-05
DDAH1	rs72882960	11	268927	T	C	0.24	0.06	0.01	2.33E-05
DDAH1	rs61905116	11	116778822	G	A	0.06	0.17	0.02	8.24E-13
DDC	rs62265630	3	124756354	G	T	0.15	-0.07	0.01	2.47E-05
DDC	rs34103191	4	68493562	G	A	0.66	-0.05	0.01	1.67E-05
DDC	rs17209803	6	32475284	A	C	0.04	0.15	0.02	1.22E-07
DDC	rs12112308	7	50535789	C	T	0.02	-1.43	0.03	1.17E-224
DDC	rs12342201	9	93132682	A	G	0.48	-0.06	0.01	7.33E-07
DDC	rs601338	19	48703417	A	G	0.51	-0.08	0.01	2.76E-14
DDC	rs3747207	22	43928975	A	G	0.22	0.08	0.01	3.43E-09
DDR1	rs3095353	6	30836031	A	T	0.59	-0.35	0.01	6.01E-209
DDR1	rs4841132	8	9326086	G	A	0.91	-0.13	0.01	5.19E-12
DDR1	rs8176644	9	133273734	T	C	0.06	0.10	0.01	5.46E-06
DDR1	rs56278466	10	17833858	G	T	0.66	0.06	0.01	1.95E-08
DDR1	rs78689694	11	126364925	C	G	0.13	-0.09	0.01	3.04E-08
DDR1	rs60309576	12	514259	A	G	0.26	-0.07	0.01	2.31E-08
DDR1	rs139097404	15	43641743	C	T	0.02	-0.17	0.02	8.72E-06
DDR1	rs186021206	17	7166093	A	G	0.01	0.84	0.05	3.09E-31
DDR1	rs111913125	17	49173964	C	A	0.10	0.12	0.01	5.60E-12
DDR1	rs708686	19	5840608	T	C	0.27	0.05	0.01	1.09E-05
DDR1	rs58560372	19	38268112	T	C	0.15	-0.07	0.01	5.17E-06
DDR1	rs681343	19	48703205	T	C	0.51	0.13	0.01	5.00E-32
DDR1	rs55953905	21	41510744	T	C	0.25	0.11	0.01	7.81E-20
DDX58	rs1133071	9	32455676	G	A	0.30	0.29	0.01	4.85E-116
DECR1	rs189045259	8	90039325	A	G	0.00	-0.96	0.06	5.29E-29
DECR1	rs6993770	8	105569300	T	A	0.29	-0.06	0.01	1.26E-06
DECR1	rs1671152	19	55014977	G	T	0.84	0.07	0.01	1.05E-05
DEFA1_DEFA1B	rs3917932	1	36478315	G	C	0.58	-0.08	0.01	3.85E-12
DEFA1_DEFA1B	rs12738019	1	54410526	G	A	0.56	-0.05	0.01	5.69E-06
DEFA1_DEFA1B	rs7561229	2	61510855	T	A	0.61	-0.05	0.01	2.35E-05
DEFA1_DEFA1B	rs61740288	2	71535283	A	G	0.02	0.21	0.03	7.81E-08
DEFA1_DEFA1B	rs13063578	3	47046347	A	T	0.40	-0.05	0.01	2.00E-06
DEFA1_DEFA1B	rs4857909	3	128581625	G	A	0.88	-0.07	0.01	2.59E-05
DEFA1_DEFA1B	rs218264	4	54542708	T	A	0.25	0.06	0.01	3.03E-06
DEFA1_DEFA1B	rs77547572	5	99115671	A	G	0.08	0.09	0.01	2.08E-05
DEFA1_DEFA1B	rs3128959	6	33080603	A	G	0.12	0.19	0.01	1.61E-28
DEFA1_DEFA1B	rs915125	6	81753659	T	C	0.28	0.06	0.01	4.68E-07
DEFA1_DEFA1B	rs210962	6	135182647	T	C	0.24	-0.10	0.01	1.29E-13
DEFA1_DEFA1B	rs116236945	8	7019405	T	C	0.39	-0.27	0.01	3.27E-96
DEFA1_DEFA1B	rs35440906	8	60733792	A	C	0.19	0.13	0.01	4.46E-19
DEFA1_DEFA1B	rs10103048	8	129590035	C	A	0.59	-0.06	0.01	2.61E-06
DEFA1_DEFA1B	rs59974297	10	99486544	T	G	0.89	-0.09	0.01	3.86E-07
DEFA1_DEFA1B	rs8071037	17	39996051	T	A	0.54	-0.11	0.01	1.22E-24
DEFA1_DEFA1B	rs145144275	19	58350442	G	A	0.01	-0.28	0.04	2.58E-06
DEFA1_DEFA1B	rs113033845	22	31918294	T	A	0.29	-0.05	0.01	1.61E-05
DEFB4A_DEFB4B	-	6	32451297	C	T	0.65	0.08	0.01	3.10E-12
DEFB4A_DEFB4B	rs117797877	8	8307070	T	C	0.02	0.23	0.03	6.99E-09
DEFB4A_DEFB4B	rs4831643	8	12620050	A	G	0.28	-0.13	0.01	4.18E-23
DEFB4A_DEFB4B	rs7220623	17	68987774	T	G	0.68	-0.05	0.01	1.29E-05
DEFB4A_DEFB4B	rs72654473	19	44911142	A	C	0.11	0.10	0.01	8.18E-08
DFFA	rs183281838	1	9978155	T	G	0.00	-0.64	0.08	2.45E-07

DFFA	rs1354034	3	56815721	C	T	0.60	0.06	0.01	4.48E-08
DFFA	-	10	63156270	A	T	0.49	0.05	0.01	4.02E-06
DIABLO	rs1354034	3	56815721	C	T	0.60	0.05	0.01	1.08E-05
DIABLO	rs55646585	8	143925453	T	C	0.41	0.05	0.01	1.26E-05
DIABLO	rs10733789	10	63188924	C	T	0.31	0.06	0.01	5.24E-06
DKK1	rs7618405	3	18209017	A	C	0.21	-0.07	0.01	2.59E-07
DKK1	rs1354034	3	56815721	C	T	0.60	-0.09	0.01	3.01E-14
DKK1	rs142178438	4	82998046	A	G	0.81	-0.07	0.01	5.98E-06
DKK1	rs755492124	4	101856385	T	G	0.39	-0.06	0.01	1.88E-07
DKK1	rs35173808	6	31358371	A	C	0.10	-0.09	0.01	9.82E-06
DKK1	rs6961069	7	80589645	T	C	0.40	0.07	0.01	7.95E-09
DKK1	rs6993770	8	105569300	T	A	0.29	-0.19	0.01	7.07E-52
DKK1	rs10820606	9	96430637	C	A	0.23	-0.06	0.01	1.17E-05
DKK1	rs7097068	10	52360120	G	T	0.44	-0.10	0.01	1.44E-19
DKK1	rs774510679	10	63304076	A	C	0.41	0.14	0.01	4.37E-35
DKK1	rs71474568	10	102589091	G	T	0.34	0.08	0.01	9.57E-11
DKK1	rs59001897	15	64868193	A	T	0.17	0.07	0.01	2.66E-06
DKK1	-	16	53256647	C	T	0.33	-0.05	0.01	2.70E-05
DKK1	rs12445050	16	81837364	T	C	0.14	0.10	0.01	5.07E-10
DKK1	rs34416903	16	88492837	C	T	0.28	0.07	0.01	2.92E-08
DKK1	rs892090	19	55027704	G	T	0.83	0.16	0.01	1.18E-27
DKK1	rs6081565	20	19307260	A	G	0.35	0.07	0.01	2.85E-08
DKK3	rs539657009	1	161658666	T	C	0.13	0.10	0.01	2.91E-09
DKK3	rs2389858	7	17868507	A	T	0.49	-0.07	0.01	3.19E-10
DKK3	rs507666	9	133273983	A	G	0.18	-0.10	0.01	2.02E-13
DKK3	rs196255	10	119720649	T	C	0.41	-0.07	0.01	6.08E-10
DKK3	rs11022114	11	12017327	A	G	0.32	0.37	0.01	3.25E-219
DKK3	rs2137537	12	70719307	C	T	0.55	-0.05	0.01	2.19E-05
DKK3	rs1609860	12	103654676	A	C	0.08	0.30	0.01	2.81E-57
DKK3	rs186021206	17	7166093	A	G	0.01	0.45	0.05	6.78E-10
DKK4	rs78058190	2	218835276	A	G	0.05	0.13	0.02	4.17E-06
DKK4	rs77203322	8	42289646	C	T	0.05	-0.55	0.02	6.04E-103
DKK4	rs1964528	8	75477823	T	C	0.11	-0.08	0.01	7.52E-06
DKK4	-	8	104781762	C	T	0.31	-0.05	0.01	1.30E-05
DKK4	rs782134971	9	133264504	G	C	0.25	-0.22	0.01	5.39E-68
DKK4	rs7232	11	60173126	A	T	0.37	0.07	0.01	6.14E-10
DKK4	rs77542162	17	69085137	G	A	0.02	0.54	0.02	2.52E-48
DKK4	rs492602	19	48703160	G	A	0.51	-0.05	0.01	1.66E-05
DKKL1	rs2740488	9	104899461	C	A	0.27	-0.04	0.00	3.09E-08
DKKL1	-	9	133274293	A	C	0.18	-0.04	0.00	2.21E-07
DKKL1	rs12149545	16	56959249	A	G	0.32	0.05	0.00	7.71E-16
DKKL1	rs112001035	17	68827664	A	G	0.06	0.25	0.01	1.17E-94
DKKL1	rs149615216	18	49579658	T	C	0.01	0.13	0.02	3.80E-06
DKKL1	rs2303759	19	49365794	G	T	0.25	-1.26	0.01	0.00E+00
DLK1	rs6772323	3	156509854	C	A	0.38	0.05	0.01	8.14E-06
DLK1	rs2074613	5	140334979	T	C	0.56	-0.05	0.01	1.55E-06
DLK1	rs732194	6	100178468	T	C	0.48	0.06	0.01	1.45E-07
DLK1	rs10823391	10	69466688	T	C	0.47	-0.06	0.01	1.96E-07
DLK1	rs10893501	11	126381138	G	A	0.13	0.07	0.01	1.06E-05
DLK1	rs12881760	14	100709998	C	G	0.67	0.49	0.01	0.00E+00
DLK1	rs112001035	17	68827664	A	G	0.06	-0.22	0.01	3.54E-23
DLL1	rs61747728	1	179557079	T	C	0.04	0.18	0.02	1.89E-10
DLL1	rs9264625	6	31270183	C	G	0.63	-0.08	0.01	9.14E-12
DLL1	rs9356631	6	170278382	G	A	0.41	0.15	0.01	1.79E-43
DLL1	rs8176672	9	133266772	T	C	0.06	0.18	0.01	6.75E-16
DLL1	rs139130389	11	72139110	A	C	0.07	-0.27	0.01	5.42E-38

DLL1	rs7137828	12	111494996	T	C	0.52	-0.05	0.01	2.83E-05
DLL1	-	16	20343260	T	A	0.18	-0.07	0.01	8.48E-07
DLL1	rs3814995	19	35851310	T	C	0.31	0.06	0.01	5.37E-08
DLL1	rs2837988	21	41247617	A	C	0.36	0.09	0.01	7.91E-15
DNAJA2	rs342298	7	106733200	T	C	0.46	-0.06	0.01	1.88E-07
DNAJB1	rs3811444	1	247876149	T	C	0.33	-0.07	0.01	3.40E-09
DNAJB1	rs1354034	3	56815721	C	T	0.60	0.08	0.01	1.91E-13
DNAJB1	rs342293	7	106731773	G	C	0.46	-0.05	0.01	3.06E-06
DNAJB1	rs10733789	10	63188924	C	T	0.31	0.06	0.01	4.73E-06
DNAJB1	rs60822569	12	54323724	C	T	0.55	0.05	0.01	2.17E-05
DNAJB1	rs11085896	19	14541074	C	A	0.61	-0.05	0.01	6.87E-06
DNAJB1	rs9612021	22	43187592	C	T	0.67	-0.09	0.01	6.71E-13
DNAJB8	rs12668458	7	157352780	C	T	0.46	0.08	0.01	1.46E-11
DNAJB8	rs112988467	14	106616753	T	C	0.27	0.06	0.01	2.39E-06
DNER	-	2	9967625	C	T	0.33	0.06	0.01	1.21E-07
DNER	rs34412673	2	229727643	T	A	0.33	-0.28	0.01	1.29E-118
DNER	rs571492629	4	56899224	T	C	0.18	0.08	0.01	2.41E-08
DNER	rs9379084	6	7231610	A	G	0.11	0.09	0.01	6.85E-07
DNER	rs4740728	9	3733885	T	C	0.56	-0.05	0.01	1.54E-05
DNER	rs62625031	10	69483873	T	A	0.04	0.14	0.02	6.21E-07
DNER	rs7298766	12	552490	G	A	0.29	0.05	0.01	1.03E-05
DNER	rs186021206	17	7166093	A	G	0.01	0.46	0.05	1.60E-09
DNER	rs8127156	21	41171476	G	C	0.26	0.10	0.01	1.91E-15
DNMBP	rs1354034	3	56815721	C	T	0.60	0.05	0.01	1.02E-05
DNMBP	rs114694170	5	88884379	C	T	0.06	0.12	0.02	9.41E-07
DNMBP	rs139736391	10	99692954	T	C	0.02	-0.45	0.03	1.05E-22
DNMBP	rs60822569	12	54323724	C	T	0.55	0.05	0.01	4.54E-06
DNPH1	rs77321231	6	43232465	T	C	0.10	-0.46	0.01	1.03E-141
DOK2	rs1354034	3	56815721	C	T	0.60	0.05	0.01	2.34E-05
DOK2	rs342298	7	106733200	T	C	0.46	-0.05	0.01	7.32E-06
DOK2	rs67385415	8	21996222	C	T	0.45	0.06	0.01	9.68E-08
DOK2	rs6993770	8	105569300	T	A	0.29	-0.09	0.01	1.66E-12
DPEP1	rs12508709	4	144398392	T	C	0.51	0.04	0.01	1.16E-06
DPEP1	rs10807578	6	14587916	C	G	0.80	0.05	0.01	1.05E-05
DPEP1	rs79137699	9	101486788	C	T	0.03	-0.13	0.02	1.23E-07
DPEP1	rs115478735	9	133274295	T	A	0.18	0.05	0.01	4.71E-06
DPEP1	rs78689694	11	126364925	C	G	0.13	-0.10	0.01	1.87E-15
DPEP1	rs423135	16	89674465	A	G	0.52	0.79	0.01	0.00E+00
DPEP1	rs186021206	17	7166093	A	G	0.01	0.98	0.04	2.63E-65
DPEP1	rs8111600	19	5821957	T	C	0.04	0.11	0.01	7.25E-07
DPEP2	rs148866368	6	32563805	A	G	0.55	-0.05	0.01	1.89E-05
DPEP2	-	9	133263362	G	A	0.18	-0.42	0.01	2.23E-187
DPEP2	rs7137828	12	111494996	T	C	0.52	-0.06	0.01	1.29E-07
DPEP2	rs144339907	16	67992095	C	A	0.00	-2.27	0.06	3.00E-135
DPEP2	rs186021206	17	7166093	A	G	0.01	0.66	0.05	4.65E-18
DPP10	rs12711819	2	115179094	A	T	0.50	-0.26	0.01	1.50E-120
DPP10	rs77119371	4	56961571	A	T	0.19	0.06	0.01	1.88E-05
DPP10	rs56204645	8	54509209	C	T	0.21	-0.09	0.01	6.53E-12
DPP10	rs2519093	9	133266456	T	C	0.18	-0.12	0.01	3.48E-17
DPP10	rs56278466	10	17833858	G	T	0.66	0.12	0.01	7.09E-26
DPP10	rs200489612	17	7203059	A	G	0.01	0.36	0.05	1.64E-05
DPP4	rs13015258	2	162074215	G	T	0.62	-0.22	0.01	1.69E-88
DPP4	rs7781670	7	12211934	G	C	0.42	0.07	0.01	4.40E-11
DPP4	rs1617484	7	65998108	A	G	0.54	0.06	0.01	6.26E-07
DPP4	rs35221426	12	20841602	T	G	0.15	0.07	0.01	2.77E-06
DPP4	rs2682725	12	64709452	C	T	0.60	0.07	0.01	7.20E-09

DPP6	rs567890117	1	161679960	A	C	0.50	0.05	0.01	1.15E-05
DPP6	rs2071592	6	31547563	A	T	0.35	-0.06	0.01	9.74E-07
DPP6	rs2240046	7	20331650	G	C	0.06	0.11	0.01	4.51E-06
DPP6	rs3734960	7	154892443	C	T	0.26	0.26	0.01	4.00E-101
DPP6	rs550057	9	133271182	T	C	0.26	-0.11	0.01	3.31E-18
DPP6	rs56278466	10	17833858	G	T	0.66	0.18	0.01	1.28E-54
DPP6	rs2289702	15	78944951	T	C	0.11	0.09	0.01	3.45E-07
DPP6	rs7285200	22	29342557	T	C	0.23	-0.06	0.01	2.05E-05
DPP7	rs35457250	3	186620775	T	C	0.01	0.36	0.04	4.34E-11
DPP7	rs2073562	8	17885420	G	A	0.69	0.09	0.01	1.28E-14
DPP7	-	9	137114213	C	A	0.74	0.51	0.01	0.00E+00
DPP7	rs9414801	10	63389329	A	G	0.53	-0.06	0.01	4.41E-09
DPP7	rs10778152	12	101831973	G	A	0.29	-0.22	0.01	4.80E-85
DPP7	rs145078947	14	93186629	T	G	0.00	0.53	0.06	1.14E-07
DPP7	rs147233090	15	43735849	T	C	0.02	0.16	0.02	3.21E-06
DPP7	rs58542926	19	19268740	T	C	0.08	0.14	0.01	2.89E-13
DPT	rs1018454	1	168728523	C	A	0.58	0.33	0.01	9.84E-216
DPT	rs67409736	3	52517268	T	G	0.56	0.05	0.01	8.38E-07
DPY30	rs67550529	2	31944496	C	T	0.21	0.07	0.01	2.62E-06
DPY30	rs6993770	8	105569300	T	A	0.29	-0.06	0.01	6.23E-06
DRAXIN	rs6679089	1	11692083	A	G	0.06	-0.60	0.02	7.62E-137
DRAXIN	rs76529516	2	60356235	G	A	0.03	0.23	0.02	3.59E-12
DRAXIN	rs13063578	3	47046347	A	T	0.40	-0.07	0.01	3.01E-11
DRAXIN	rs10002742	4	140099011	A	G	0.44	-0.05	0.01	3.87E-06
DRAXIN	rs17361097	4	152080067	A	G	0.44	0.06	0.01	6.33E-08
DRAXIN	rs304153	5	88825371	A	T	0.31	0.07	0.01	2.91E-09
DRAXIN	rs180798958	5	143809411	A	G	0.02	-0.20	0.03	9.55E-07
DRAXIN	rs4704963	5	158820370	C	T	0.08	-0.19	0.01	3.59E-22
DRAXIN	rs652951	6	44596271	A	C	0.49	-0.05	0.01	4.67E-07
DRAXIN	rs9483788	6	135114363	C	T	0.26	-0.06	0.01	3.03E-06
DRAXIN	rs60160896	7	55979879	T	C	0.41	0.06	0.01	3.45E-09
DRAXIN	rs62621812	7	127375029	A	G	0.02	0.27	0.02	7.36E-13
DRAXIN	rs10107630	8	129591389	T	C	0.57	0.08	0.01	3.48E-13
DRAXIN	-	10	61976858	C	T	0.65	-0.10	0.01	1.81E-18
DRAXIN	rs11410806	15	81552430	A	T	0.19	0.07	0.01	3.55E-07
DRAXIN	rs9927316	16	85982795	G	C	0.24	0.09	0.01	1.50E-12
DRAXIN	rs7508294	19	33252791	A	G	0.34	0.06	0.01	3.86E-08
DRAXIN	rs681343	19	48703205	T	C	0.51	0.06	0.01	3.83E-09
DRG2	rs1354034	3	56815721	C	T	0.60	0.07	0.01	1.40E-08
DRG2	rs17622656	5	132485305	A	G	0.39	-0.05	0.01	2.01E-05
DRG2	rs147899431	6	116571518	C	G	0.04	0.27	0.02	5.83E-22
DRG2	rs60822569	12	54323724	C	T	0.55	0.05	0.01	1.39E-05
DSC2	rs61747728	1	179557079	T	C	0.04	0.19	0.02	8.74E-11
DSC2	rs8176672	9	133266772	T	C	0.06	0.13	0.01	3.26E-08
DSC2	rs56278466	10	17833858	G	T	0.66	0.06	0.01	3.41E-08
DSC2	rs745680717	12	480706	C	G	0.40	-0.06	0.01	5.61E-07
DSC2	rs111338191	12	111388673	T	A	0.52	-0.06	0.01	6.45E-08
DSC2	-	16	20343260	T	A	0.18	-0.08	0.01	2.14E-07
DSC2	rs186021206	17	7166093	A	G	0.01	0.40	0.05	1.46E-07
DSC2	rs2302774	17	40026837	T	G	0.38	-0.05	0.01	1.21E-05
DSC2	rs2665405	17	59797931	A	G	0.55	0.10	0.01	5.83E-20
DSC2	rs4799305	18	31109181	A	G	0.88	-0.34	0.01	1.99E-84
DSC2	rs3814995	19	35851310	T	C	0.31	0.09	0.01	1.13E-13
DSC2	rs516316	19	48702888	C	G	0.51	-0.09	0.01	3.92E-17
DSG2	rs61747728	1	179557079	T	C	0.04	0.15	0.02	8.14E-08
DSG2	rs13127398	4	102000547	A	T	0.07	0.11	0.01	7.45E-07

DSG2	rs998584	6	43790159	A	C	0.48	-0.08	0.01	7.86E-14
DSG2	rs648802	6	138092196	C	G	0.54	-0.14	0.01	2.50E-37
DSG2	rs8176741	9	133256074	A	G	0.06	0.42	0.01	1.33E-77
DSG2	rs1716403	12	124035299	C	T	0.68	-0.05	0.01	2.02E-05
DSG2	rs186021206	17	7166093	A	G	0.01	0.32	0.05	1.87E-05
DSG2	rs2704053	18	31515126	G	A	0.48	-0.22	0.01	1.23E-90
DSG2	rs708686	19	5840608	T	C	0.27	0.11	0.01	3.86E-19
DSG2	rs3814995	19	35851310	T	C	0.32	0.06	0.01	2.12E-07
DSG3	rs80293268	1	8147519	C	G	0.05	-0.19	0.02	3.59E-13
DSG3	rs61816761	1	152313385	A	G	0.02	0.24	0.02	8.24E-11
DSG3	rs1260326	2	27508073	C	T	0.61	0.08	0.01	3.74E-13
DSG3	rs1440152	3	98771071	G	C	0.44	0.05	0.01	1.15E-05
DSG3	rs833270	3	181802378	C	T	0.62	0.06	0.01	2.39E-08
DSG3	rs73187740	3	186938218	C	G	0.06	0.10	0.01	2.09E-05
DSG3	rs13107325	4	102267552	T	C	0.08	0.16	0.01	4.48E-16
DSG3	rs7905367	10	52574893	C	G	0.80	0.07	0.01	1.49E-07
DSG3	rs683486	11	126356768	A	C	0.40	0.06	0.01	3.80E-09
DSG3	rs2286782	12	519070	A	C	0.22	-0.09	0.01	2.18E-11
DSG3	rs12306780	12	89528083	T	A	0.35	0.18	0.01	2.03E-60
DSG3	rs34177108	16	89826967	A	C	0.27	0.05	0.01	5.40E-06
DSG3	rs186021206	17	7166093	A	G	0.01	0.80	0.05	5.52E-29
DSG3	rs7234091	18	31439866	T	C	0.18	-0.31	0.01	5.53E-113
DSG3	rs12457893	18	63258928	C	A	0.46	-0.05	0.01	8.01E-06
DSG3	rs3814995	19	35851310	T	C	0.31	0.07	0.01	1.13E-09
DSG3	rs2682581	19	43462354	G	C	0.53	-0.07	0.01	5.65E-12
DSG4	rs80293268	1	8147519	C	G	0.05	-0.21	0.02	1.68E-14
DSG4	rs12142181	1	41447314	T	C	0.08	0.09	0.01	1.31E-05
DSG4	rs11589479	1	155060832	A	G	0.16	-0.08	0.01	1.24E-07
DSG4	rs1260326	2	27508073	C	T	0.61	0.06	0.01	7.57E-08
DSG4	-	3	11620052	C	G	0.73	-0.06	0.01	3.81E-07
DSG4	rs1345417	3	181794163	G	C	0.60	0.08	0.01	5.56E-13
DSG4	rs13107325	4	102267552	T	C	0.08	0.12	0.01	5.27E-08
DSG4	rs62370336	5	53861705	G	A	0.14	-0.08	0.01	3.04E-06
DSG4	rs2436396	5	140153978	G	A	0.70	-0.09	0.01	5.62E-13
DSG4	rs12203592	6	396321	T	C	0.22	-0.06	0.01	3.43E-06
DSG4	rs9358325	6	10259504	T	A	0.83	-0.08	0.01	7.98E-08
DSG4	-	6	21921876	T	G	0.15	0.07	0.01	9.70E-06
DSG4	rs1918719	8	116293163	C	T	0.20	-0.08	0.01	2.46E-09
DSG4	-	9	22071285	C	T	0.54	0.05	0.01	1.29E-05
DSG4	rs7905367	10	52574893	C	G	0.80	0.10	0.01	3.34E-13
DSG4	rs2286782	12	519070	A	C	0.22	-0.17	0.01	4.24E-36
DSG4	rs12426301	12	53855627	T	C	0.20	0.12	0.01	4.42E-18
DSG4	rs1800176	12	57167668	T	C	0.31	0.07	0.01	5.67E-09
DSG4	rs7973165	12	90740429	C	T	0.27	0.07	0.01	7.59E-09
DSG4	rs369230	16	89579029	T	G	0.69	-0.05	0.01	2.16E-05
DSG4	rs9896243	17	46748690	G	C	0.22	0.10	0.01	1.37E-12
DSG4	rs55842605	17	78941609	T	C	0.55	0.07	0.01	3.78E-10
DSG4	rs34620697	18	31403566	T	C	0.01	-0.37	0.05	3.76E-07
DSG4	rs8084922	18	63263288	C	G	0.47	-0.08	0.01	1.57E-12
DSG4	rs4803461	19	41402966	A	G	0.60	-0.05	0.01	8.54E-06
DTX3	rs542212699	1	223712908	A	G	0.01	-0.67	0.04	1.18E-32
DTX3	rs151828	5	96724558	C	T	0.55	0.16	0.01	3.98E-50
DTX3	rs2232659	10	103449656	T	C	0.02	0.18	0.03	1.08E-05
DTX3	rs2277323	12	57615589	A	G	0.25	-0.11	0.01	4.31E-18
DUSP3	rs10733789	10	63188924	C	T	0.31	0.07	0.01	1.63E-07
DUSP3	rs187793617	14	105894833	G	C	0.16	0.09	0.01	2.80E-08

EBAG9	rs1354034	3	56815721	C	T	0.60	0.08	0.01	2.06E-12
EBAG9	rs17622656	5	132485305	A	G	0.39	-0.05	0.01	1.06E-05
EBAG9	rs342293	7	106731773	G	C	0.46	-0.05	0.01	6.21E-06
EBAG9	rs6993770	8	105569300	T	A	0.29	-0.07	0.01	1.09E-07
EBAG9	rs2022934	8	109555545	A	G	0.39	-0.07	0.01	7.74E-09
EBI3_IL27	rs4970836	1	109279175	A	G	0.77	0.07	0.01	2.92E-08
EBI3_IL27	rs10183338	2	110853239	T	G	0.29	-0.05	0.01	1.11E-05
EBI3_IL27	rs62165726	2	134208991	A	C	0.04	-0.28	0.02	7.83E-24
EBI3_IL27	rs11456863	3	12230418	T	A	0.35	0.05	0.01	7.45E-06
EBI3_IL27	rs10513801	3	186104564	G	T	0.14	0.07	0.01	2.26E-05
EBI3_IL27	rs11713634	3	194340849	A	G	0.28	-0.13	0.01	1.65E-29
EBI3_IL27	rs5743618	4	38797027	A	C	0.22	0.11	0.01	1.71E-19
EBI3_IL27	rs115216147	5	71369699	A	T	0.07	0.09	0.01	2.20E-05
EBI3_IL27	rs4713572	6	32659175	C	T	0.40	0.07	0.01	2.34E-10
EBI3_IL27	rs2529440	7	30472178	T	C	0.44	0.05	0.01	6.23E-06
EBI3_IL27	rs339060	7	128735549	G	A	0.55	0.06	0.01	6.73E-08
EBI3_IL27	rs4986790	9	117713024	G	A	0.06	-0.12	0.01	1.09E-07
EBI3_IL27	rs4486555	10	100039545	T	G	0.41	-0.19	0.01	8.79E-72
EBI3_IL27	rs61377406	11	122664422	T	G	0.36	-0.06	0.01	1.71E-07
EBI3_IL27	rs12810719	12	29325081	G	A	0.23	0.06	0.01	2.61E-06
EBI3_IL27	rs58298943	12	56997508	T	C	0.08	0.08	0.01	1.15E-05
EBI3_IL27	rs3184504	12	111446804	C	T	0.52	-0.08	0.01	3.71E-14
EBI3_IL27	rs72749499	15	57003381	G	A	0.07	0.09	0.01	2.29E-05
EBI3_IL27	-	16	28501747	A	T	0.34	-0.07	0.01	2.74E-10
EBI3_IL27	rs186021206	17	7166093	A	G	0.01	0.82	0.05	1.94E-30
EBI3_IL27	rs704	17	28367840	A	G	0.47	-0.10	0.01	7.58E-21
EBI3_IL27	-	19	4240307	C	A	0.29	-0.41	0.01	2.19E-269
EBI3_IL27	rs4804669	19	12391643	G	A	0.78	-0.10	0.01	1.31E-13
EBI3_IL27	rs7264079	20	35567019	C	G	0.14	0.10	0.01	6.73E-11
ECE1	rs181637959	1	21219540	T	A	0.01	-1.03	0.05	5.27E-46
ECE1	rs147953260	3	52520515	A	G	0.00	0.59	0.07	2.60E-07
ECE1	-	9	133263362	G	A	0.18	-0.39	0.01	1.06E-149
ECE1	rs181242111	10	17823665	A	G	0.12	0.08	0.01	1.62E-05
ECE1	rs35166255	11	126431861	A	G	0.03	0.23	0.02	1.48E-12
ECE1	rs186021206	17	7166093	A	G	0.01	0.53	0.05	1.73E-11
EDA2R	rs4335411	1	248897507	A	G	0.76	-0.06	0.01	6.69E-08
EDA2R	rs10069690	5	1279675	T	C	0.26	-0.05	0.01	3.49E-06
EDA2R	rs78378222	17	7668434	G	T	0.01	-0.26	0.03	1.52E-10
EDAR	rs72665955	1	54375983	A	G	0.12	0.13	0.01	1.90E-14
EDAR	rs66858280	1	247878519	G	C	0.31	-0.05	0.01	1.31E-05
EDAR	rs12712870	2	42934040	G	A	0.69	0.09	0.01	9.10E-15
EDAR	rs140661471	2	108987283	A	G	0.01	1.64	0.03	5.86E-261
EDAR	rs28362642	3	39108012	G	A	0.11	0.08	0.01	5.09E-06
EDAR	rs1420476	3	169146762	A	T	0.96	-0.14	0.02	8.94E-08
EDAR	rs17501461	4	105068616	T	C	0.17	0.07	0.01	8.93E-06
EDAR	rs114694170	5	88884379	C	T	0.06	-0.21	0.01	3.55E-19
EDAR	rs139141690	7	101856650	A	G	0.00	-0.36	0.05	1.15E-05
EDAR	rs67036916	7	106727190	A	G	0.46	0.07	0.01	1.70E-11
EDAR	rs6993770	8	105569300	T	A	0.29	0.23	0.01	2.49E-80
EDAR	rs692502	9	4745645	A	G	0.56	0.05	0.01	7.06E-06
EDAR	rs12377089	9	134076173	G	A	0.32	-0.11	0.01	5.00E-20
EDAR	rs7080386	10	63288546	A	C	0.41	0.12	0.01	4.28E-26
EDAR	rs16937003	10	79178742	A	G	0.02	-0.17	0.03	2.16E-05
EDAR	rs12762934	10	102600127	T	C	0.32	-0.15	0.01	2.75E-36
EDAR	rs652963	11	32890191	T	C	0.84	0.08	0.01	6.08E-08
EDAR	rs780615646	11	128703863	A	C	0.78	-0.06	0.01	5.42E-06

EDAR	rs11553699	12	121779004	G	A	0.14	-0.10	0.01	3.33E-09
EDAR	rs141232262	14	68092020	G	A	0.15	-0.07	0.01	4.78E-06
EDAR	rs1820994	15	57005581	G	A	0.79	0.10	0.01	4.45E-15
EDAR	rs8057254	16	8959132	A	T	0.19	0.08	0.01	7.31E-09
EDAR	rs74035509	16	88500925	T	C	0.08	-0.21	0.01	3.14E-24
EDAR	rs111527738	21	34887027	G	A	0.02	-0.23	0.02	1.67E-09
EDIL3	rs56210800	3	124753745	G	C	0.13	-0.16	0.01	2.14E-21
EDIL3	-	5	84018783	C	T	0.23	-0.10	0.01	1.27E-12
EDIL3	rs111338191	12	111388673	T	A	0.52	0.05	0.01	8.61E-06
EDIL3	rs1801690	17	66212167	G	C	0.06	0.11	0.02	5.89E-06
EDIL3	rs34931250	17	68883786	T	C	0.06	0.26	0.02	1.29E-27
EFEMP1	rs188468174	1	24965206	T	C	0.01	-0.23	0.03	4.45E-07
EFEMP1	rs1260326	2	27508073	C	T	0.61	0.05	0.01	5.00E-07
EFEMP1	rs59985551	2	55879793	T	C	0.23	0.22	0.01	8.83E-72
EFEMP1	rs2070634	3	186618238	G	T	0.51	0.05	0.01	3.74E-06
EFNA1	rs4390169	1	155133578	G	A	0.52	0.31	0.01	1.11E-170
EFNA1	-	1	172422763	A	G	0.55	-0.06	0.01	3.68E-08
EFNA1	rs28817415	4	76480299	T	C	0.46	0.06	0.01	5.41E-08
EFNA1	rs174581	11	61839211	A	G	0.35	0.05	0.01	1.59E-05
EFNA1	rs28929474	14	94378610	T	C	0.02	0.19	0.03	1.46E-06
EFNA4	rs148289726	1	155066794	T	C	0.00	-1.28	0.08	6.68E-28
EFNA4	rs6737671	2	221522212	C	T	0.51	0.06	0.01	2.38E-07
EFNA4	rs3184504	12	111446804	C	T	0.52	-0.06	0.01	4.01E-08
EFNA4	rs7187875	16	363747	G	A	0.54	-0.06	0.01	8.27E-08
EFNA4	-	16	20343260	T	A	0.18	-0.08	0.01	8.99E-08
EFNA4	rs4760	19	43648948	G	A	0.16	0.09	0.01	9.66E-09
EGF	rs10016018	4	101873464	T	A	0.40	-0.07	0.01	5.20E-09
EGF	rs3831508	4	109916747	T	G	0.40	0.13	0.01	3.26E-30
EGF	rs2631367	5	132369766	G	C	0.52	0.06	0.01	1.63E-07
EGF	rs6961069	7	80589645	T	C	0.40	0.06	0.01	4.40E-08
EGF	rs6993770	8	105569300	T	A	0.29	-0.09	0.01	1.70E-13
EGF	rs10820606	9	96430637	C	A	0.23	-0.07	0.01	1.46E-06
EGF	rs7896518	10	63344740	G	A	0.42	0.09	0.01	1.21E-13
EGF	rs1362213	12	6173262	T	C	0.48	-0.06	0.01	6.13E-07
EGF	rs12445050	16	81837364	T	C	0.14	0.09	0.01	3.57E-08
EGF	rs892090	19	55027704	G	T	0.83	0.15	0.01	1.36E-21
EGF	rs6081565	20	19307260	A	G	0.35	0.06	0.01	7.51E-07
EGFL7	rs78945826	1	25730925	C	A	0.03	-0.15	0.02	2.66E-05
EGFL7	rs1434282	1	199041592	T	C	0.73	0.05	0.01	2.33E-05
EGFL7	rs823066	1	205802045	G	A	0.95	-0.11	0.02	1.39E-05
EGFL7	rs78909033	2	240571486	A	G	0.14	-0.07	0.01	5.80E-06
EGFL7	rs114694170	5	88884379	C	T	0.06	0.11	0.02	7.38E-06
EGFL7	rs3131622	6	31452723	G	T	0.45	0.07	0.01	7.02E-09
EGFL7	rs6796	7	6462736	C	T	0.28	-0.17	0.01	2.41E-41
EGFL7	rs10266182	7	35707715	A	G	0.38	-0.08	0.01	3.52E-13
EGFL7	rs17154155	7	80604927	T	G	0.41	0.05	0.01	3.04E-06
EGFL7	rs339061	7	128733774	C	T	0.55	-0.05	0.01	5.77E-06
EGFL7	rs883006	8	33436909	G	C	0.56	-0.07	0.01	1.03E-09
EGFL7	rs8193003	9	132990109	C	T	0.61	0.06	0.01	5.07E-07
EGFL7	rs74557797	9	136656051	T	G	0.09	-0.28	0.01	2.32E-42
EGFL7	rs10761731	10	63267850	T	A	0.41	0.07	0.01	2.39E-09
EGFL7	rs55646096	11	65151043	T	C	0.18	-0.16	0.01	2.27E-29
EGFL7	rs9635249	14	65331066	G	A	0.79	0.08	0.01	7.04E-08
EGFL7	rs12444373	16	88498177	G	C	0.04	0.15	0.02	5.66E-07
EGFL7	rs892090	19	55027704	G	T	0.83	0.11	0.01	3.63E-14
EGFR	rs1260326	2	27508073	C	T	0.61	-0.05	0.01	1.86E-05

EGFR	-	3	136181921	A	T	0.70	0.05	0.01	8.75E-06
EGFR	rs34241405	6	32593810	A	G	0.32	-0.05	0.01	1.31E-05
EGFR	rs10081281	7	55169103	T	C	0.45	0.17	0.01	1.57E-54
EGFR	rs199922514	8	9325592	G	A	0.91	-0.11	0.01	7.66E-09
EGFR	rs28601761	8	125487789	G	C	0.42	-0.05	0.01	2.03E-05
EGFR	rs77582897	10	17850189	C	T	0.52	0.05	0.01	2.71E-05
EGFR	rs3967200	11	126362490	T	C	0.13	-0.09	0.01	1.26E-08
EGFR	rs540639423	12	111407152	T	C	0.01	-0.28	0.04	9.48E-06
EGFR	rs186021206	17	7166093	A	G	0.01	0.62	0.05	1.88E-16
EGLN1	rs1434282	1	199041592	T	C	0.73	0.08	0.01	9.37E-11
EGLN1	rs77466488	1	231488623	G	A	0.04	0.34	0.02	1.61E-30
EGLN1	rs519417	6	31910656	A	G	0.13	-0.07	0.01	1.10E-05
EGLN1	rs342296	7	106732457	A	G	0.46	-0.08	0.01	1.22E-11
EIF4B	rs1354034	3	56815721	C	T	0.60	0.08	0.01	4.76E-13
EIF4B	rs342298	7	106733200	T	C	0.45	-0.06	0.01	7.92E-07
EIF4B	rs10733789	10	63188924	C	T	0.31	0.06	0.01	1.97E-06
EIF4B	rs11502185	11	180258	C	T	0.26	0.07	0.01	2.00E-06
EIF4B	rs60822569	12	54323724	C	T	0.55	0.05	0.01	4.67E-06
EIF4G1	rs1354034	3	56815721	C	T	0.60	0.09	0.01	8.70E-15
EIF4G1	rs342298	7	106733200	T	C	0.46	-0.06	0.01	1.45E-06
EIF4G1	rs6993770	8	105569300	T	A	0.29	-0.06	0.01	8.86E-06
EIF4G1	-	10	63156270	A	T	0.49	0.05	0.01	8.53E-06
ELOA	rs2473378	1	23724245	A	G	0.59	0.08	0.01	2.50E-13
ELOA	rs10418046	19	53824615	G	T	0.21	0.14	0.01	2.35E-25
ENAH	-	1	199025567	T	A	0.72	0.06	0.01	1.52E-05
ENAH	rs10799316	1	225480329	A	G	0.75	0.12	0.01	2.75E-19
ENAH	rs11438680	10	63414938	A	G	0.46	0.06	0.01	7.64E-07
ENG	rs61762319	3	155084189	G	A	0.03	-0.21	0.02	2.27E-11
ENG	rs6437412	3	194747684	C	T	0.29	0.06	0.01	1.95E-07
ENG	rs9987289	8	9325848	G	A	0.91	-0.08	0.01	1.99E-05
ENG	rs11794565	9	127838998	T	C	0.46	0.26	0.01	5.12E-111
ENG	rs2519093	9	133266456	T	C	0.18	-0.50	0.01	4.86E-256
ENG	rs35166255	11	126431861	A	G	0.03	0.29	0.02	1.75E-22
ENG	rs186021206	17	7166093	A	G	0.01	0.72	0.05	9.52E-23
ENG	rs4760	19	43648948	G	A	0.16	0.24	0.01	6.23E-60
ENG	rs558698413	20	35120761	A	C	0.00	-0.99	0.12	2.38E-07
ENO1	rs11544513	1	8866415	T	G	0.00	-1.29	0.06	5.13E-39
ENO1	rs1354034	3	56815721	C	T	0.60	0.06	0.01	7.00E-08
ENO1	rs17622656	5	132485305	A	G	0.39	-0.05	0.01	1.61E-05
ENO1	rs4746204	10	63568758	T	C	0.48	-0.05	0.01	2.08E-05
ENO2	rs1354034	3	56815721	C	T	0.60	0.06	0.01	9.19E-07
ENO2	rs6993770	8	105569300	T	A	0.29	-0.14	0.01	4.07E-31
ENO2	rs60757417	9	132989049	G	C	0.06	-0.11	0.02	4.96E-06
ENO2	rs7080386	10	63288546	A	C	0.41	0.10	0.01	4.22E-20
ENO2	rs17655730	11	270715	C	T	0.25	0.06	0.01	1.05E-05
ENO2	rs11446182	12	6913195	G	A	0.27	0.15	0.01	4.77E-31
ENPP2	rs34707604	4	68625738	C	T	0.26	0.06	0.01	3.85E-07
ENPP2	rs4921913	8	18414867	T	C	0.78	0.07	0.01	1.48E-09
ENPP2	rs13267597	8	119641985	G	T	0.33	-0.26	0.01	1.36E-150
ENPP2	rs10901252	9	133252613	C	G	0.06	0.10	0.01	2.45E-07
ENPP2	rs11830764	12	111077216	C	G	0.07	-0.11	0.01	3.93E-09
ENPP2	rs7970695	12	120985573	A	G	0.62	0.09	0.01	7.89E-23
ENPP2	rs186021206	17	7166093	A	G	0.01	0.34	0.04	4.68E-08
ENPP2	rs77542162	17	69085137	G	A	0.02	0.14	0.02	1.93E-06
ENPP5	rs4665972	2	27375230	C	T	0.61	0.04	0.01	3.25E-06
ENPP5	rs9472701	6	46148712	A	G	0.64	-0.77	0.01	0.00E+00

ENPP5	rs1299525	8	11864616	T	C	0.43	0.04	0.00	9.48E-07
ENPP5	rs75491173	11	16346948	A	G	0.06	0.08	0.01	1.14E-06
ENPP5	rs11072817	15	78944473	A	G	0.70	0.05	0.01	1.02E-09
ENPP5	rs12903256	15	84764910	T	G	0.34	-0.04	0.01	1.20E-07
ENPP5	rs200489612	17	7203059	A	G	0.01	0.35	0.04	7.03E-10
ENPP5	rs141947687	20	45859438	G	A	0.64	-0.04	0.01	5.41E-06
ENPP7	rs114165349	1	26695422	C	G	0.02	0.15	0.02	7.88E-07
ENPP7	rs199900492	1	234714673	C	A	0.52	-0.05	0.01	5.59E-07
ENPP7	-	5	132416951	G	A	0.19	0.05	0.01	1.28E-05
ENPP7	-	6	24429112	C	T	0.00	-0.38	0.04	3.55E-08
ENPP7	rs28601761	8	125487789	G	C	0.42	-0.04	0.01	5.93E-06
ENPP7	rs762439986	10	63154758	C	A	0.47	0.07	0.01	7.98E-14
ENPP7	rs2255531	12	120977112	A	G	0.35	0.06	0.01	2.48E-09
ENPP7	rs112635299	14	94371805	T	G	0.02	0.14	0.02	1.12E-05
ENPP7	rs147233090	15	43735849	T	C	0.02	0.15	0.02	1.01E-06
ENPP7	rs11868696	17	79732608	G	A	0.37	0.94	0.01	0.00E+00
ENTPD2	-	9	133255468	T	G	0.06	0.15	0.02	2.62E-09
ENTPD2	-	9	137049085	T	G	0.02	-1.49	0.03	4.09E-294
ENTPD2	rs56278466	10	17833858	G	T	0.66	0.05	0.01	1.20E-05
ENTPD2	rs5848	17	44352876	T	C	0.27	0.06	0.01	4.41E-07
ENTPD5	rs35457250	3	186620775	T	C	0.01	0.76	0.04	1.19E-39
ENTPD5	rs6054	4	154568456	T	C	0.01	0.33	0.05	8.49E-06
ENTPD5	rs28456	11	61822009	G	A	0.31	0.07	0.01	2.27E-08
ENTPD5	rs10843403	12	29387376	C	T	0.61	0.08	0.01	1.33E-11
ENTPD5	rs146086623	14	74000519	A	G	0.05	-0.96	0.02	0.00E+00
ENTPD5	rs28929474	14	94378610	T	C	0.02	0.54	0.03	1.43E-41
ENTPD5	rs763665	16	72044144	T	C	0.16	0.08	0.01	1.60E-07
ENTPD5	rs8178824	17	66228657	T	C	0.03	0.41	0.02	4.66E-37
ENTPD5	rs7236989	18	59343221	C	T	0.63	-0.05	0.01	1.75E-06
ENTPD6	rs61804164	1	161653235	C	G	0.12	0.07	0.01	4.19E-06
ENTPD6	rs12489828	3	52532998	T	G	0.53	0.05	0.01	7.69E-06
ENTPD6	rs505922	9	133273813	C	T	0.32	0.14	0.01	1.40E-38
ENTPD6	rs139196635	12	101863758	A	G	0.02	0.16	0.02	1.59E-05
ENTPD6	rs28929474	14	94378610	T	C	0.02	0.19	0.02	1.14E-07
ENTPD6	rs704	17	28367840	A	G	0.47	-0.05	0.01	3.74E-07
ENTPD6	rs17695224	19	51820963	A	G	0.27	-0.10	0.01	1.15E-17
ENTPD6	rs6050446	20	25214873	G	A	0.97	1.86	0.02	0.00E+00
ENTPD6	rs6061054	20	31223514	G	T	0.96	1.57	0.02	0.00E+00
EPCAM	rs1430780	2	67651196	C	T	0.68	-0.05	0.01	1.40E-05
EPCAM	rs2241764	2	132417191	T	C	0.34	-0.08	0.01	2.47E-12
EPCAM	rs35853577	2	232694169	C	T	0.43	-0.07	0.01	5.19E-09
EPCAM	rs1713810	3	154159037	G	A	0.85	-0.08	0.01	7.58E-08
EPCAM	rs939885	3	196228891	A	G	0.49	0.06	0.01	5.64E-07
EPCAM	rs9478784	6	150699194	T	C	0.10	0.12	0.01	3.82E-10
EPCAM	rs11142461	9	68444973	C	T	0.04	-0.17	0.02	1.46E-08
EPCAM	-	9	133263362	G	A	0.18	-0.15	0.01	6.19E-27
EPCAM	rs2291428	10	45463408	C	G	0.24	-0.09	0.01	1.84E-12
EPCAM	rs56398830	13	103049340	A	G	0.01	-0.82	0.03	1.24E-55
EPCAM	rs35193388	16	4370816	C	G	0.73	-0.07	0.01	3.57E-08
EPCAM	rs12938714	17	30318092	A	G	0.33	0.16	0.01	2.58E-42
EPCAM	-	17	39383590	C	T	0.26	0.07	0.01	2.54E-07
EPCAM	rs2318997	18	57861998	G	T	0.43	-0.05	0.01	6.51E-06
EPCAM	rs681343	19	48703205	T	C	0.51	-0.23	0.01	9.30E-96
EPCAM	rs3746778	20	62710120	A	G	0.42	0.06	0.01	6.47E-07
EPHA1	rs1497406	1	16178825	G	A	0.58	0.06	0.01	9.18E-09
EPHA1	rs61747728	1	179557079	T	C	0.04	0.13	0.02	4.06E-06

EPHA1	rs75045569	7	143412115	G	T	0.18	0.25	0.01	1.82E-75
EPHA1	rs10740131	10	63511728	T	A	0.47	0.10	0.01	3.41E-20
EPHA1	rs28929474	14	94378610	T	C	0.02	0.29	0.02	6.07E-15
EPHA1	rs34882080	16	20350119	G	A	0.18	-0.06	0.01	1.64E-05
EPHA1	rs3814995	19	35851310	T	C	0.31	0.06	0.01	3.88E-07
EPHA2	rs924204	1	16187431	G	A	0.59	-0.13	0.01	1.85E-31
EPHA2	rs61747728	1	179557079	T	C	0.04	0.16	0.02	1.01E-07
EPHA2	rs11689257	2	162101843	A	G	0.07	-0.11	0.01	2.68E-06
EPHA2	-	6	32643672	G	T	0.18	0.10	0.01	2.94E-10
EPHA2	rs8176746	9	133255935	T	G	0.06	0.14	0.01	1.05E-09
EPHA2	rs77924615	16	20381010	A	G	0.20	-0.07	0.01	2.68E-06
EPHA2	rs679574	19	48702851	G	C	0.51	-0.07	0.01	8.01E-10
EPHB4	rs61747728	1	179557079	T	C	0.04	0.15	0.02	8.46E-08
EPHB4	rs10179584	2	18497405	C	T	0.34	0.05	0.01	1.85E-05
EPHB4	rs314361	7	100815383	G	A	0.52	0.15	0.01	1.52E-43
EPHB4	rs507666	9	133273983	A	G	0.18	-0.37	0.01	4.66E-146
EPHB4	rs7099071	10	28704967	C	T	0.17	0.06	0.01	2.61E-05
EPHB4	rs7909516	10	76125579	T	C	0.22	-0.09	0.01	2.92E-12
EPHB4	rs9558661	13	105997744	T	C	0.20	-0.13	0.01	1.06E-20
EPHB4	rs4997081	16	20353912	C	G	0.19	-0.08	0.01	8.71E-09
EPHB4	rs186021206	17	7166093	A	G	0.01	0.39	0.05	4.49E-07
EPHB4	rs1137844	19	35852177	G	C	0.32	0.07	0.01	5.13E-08
EPHB4	-	19	49546384	G	T	0.25	0.06	0.01	1.48E-05
EPHB6	rs61747728	1	179557079	T	C	0.04	0.20	0.02	4.57E-12
EPHB6	rs7789303	7	142854785	G	A	0.27	-0.32	0.01	8.85E-134
EPHB6	rs13329952	16	20355185	C	T	0.19	-0.09	0.01	2.37E-09
EPHB6	rs1137844	19	35852177	G	C	0.32	0.06	0.01	5.25E-06
EPHX2	rs71553864	8	27536817	C	A	0.06	0.26	0.02	2.71E-25
EPO	rs218264	4	54542708	T	A	0.25	0.08	0.01	1.62E-08
EPO	rs2032444	6	26046516	G	T	0.35	-0.08	0.01	3.76E-10
EPO	rs1546723	6	109304676	A	G	0.45	0.06	0.01	1.88E-07
EPO	rs7776054	6	135097778	G	A	0.26	0.21	0.01	1.93E-56
EPO	rs11976235	7	100722758	T	C	0.01	0.53	0.05	1.16E-12
EPO	rs78744187	19	33263642	T	C	0.08	-0.12	0.01	3.61E-08
EPO	rs73036519	19	45245104	C	G	0.30	0.06	0.01	8.75E-06
EPO	rs4811073	20	50483272	T	C	0.54	-0.05	0.01	8.88E-06
EPO	rs855791	22	37066896	G	A	0.56	-0.08	0.01	2.11E-11
EPS8L2	rs9271657	6	32624805	C	T	0.21	0.06	0.01	6.29E-06
EPS8L2	rs1106192	9	93134304	G	C	0.05	-0.11	0.02	2.28E-05
EPS8L2	rs11246276	11	706284	T	G	0.16	-0.39	0.01	6.89E-149
EPS8L2	rs3747207	22	43928975	A	G	0.22	0.06	0.01	3.16E-06
ERBB2	-	3	136181921	A	T	0.70	0.07	0.01	3.27E-08
ERBB2	rs9270487	6	32591299	G	A	0.33	-0.07	0.01	3.53E-08
ERBB2	rs1461729	8	9329732	G	A	0.90	-0.09	0.01	2.09E-07
ERBB2	rs8176749	9	133255801	T	C	0.06	0.11	0.01	1.31E-06
ERBB2	-	10	63501416	C	T	0.47	0.07	0.01	1.57E-09
ERBB2	-	10	69509433	A	T	0.43	0.05	0.01	1.93E-05
ERBB2	rs11045856	12	21197755	G	T	0.24	0.07	0.01	1.79E-08
ERBB2	rs28929474	14	94378610	T	C	0.02	0.23	0.02	5.60E-10
ERBB2	rs4788460	16	72120610	T	C	0.29	0.05	0.01	1.58E-05
ERBB2	rs3764354	17	39760570	T	C	0.15	0.20	0.01	6.07E-39
ERBB2	rs1801689	17	66214462	C	A	0.03	0.31	0.02	2.16E-22
ERBB2	rs1800961	20	44413724	T	C	0.03	-0.13	0.02	2.50E-05
ERBB3	rs1260326	2	27508073	C	T	0.61	-0.13	0.01	3.32E-34
ERBB3	rs370542983	2	241333621	A	T	0.35	-0.05	0.01	2.78E-05
ERBB3	rs13234131	7	73611645	G	A	0.13	-0.08	0.01	3.27E-07

ERBB3	rs1077481	7	129102897	T	C	0.55	-0.10	0.01	2.08E-21
ERBB3	rs28601761	8	125487789	G	C	0.42	-0.06	0.01	4.01E-07
ERBB3	rs10822159	10	63336490	T	C	0.41	-0.05	0.01	1.35E-05
ERBB3	rs75526830	12	56095610	G	A	0.00	2.48	0.05	8.06E-219
ERBB3	rs150844304	15	43434427	C	A	0.02	0.20	0.02	1.03E-08
ERBB3	rs149394327	17	66232877	C	G	0.03	0.19	0.02	2.92E-09
ERBB4	rs9787076	1	43675478	C	A	0.33	0.06	0.01	9.43E-08
ERBB4	rs35383942	1	201468704	T	C	0.06	0.13	0.01	7.77E-09
ERBB4	rs12692385	2	9554491	T	C	0.67	0.08	0.01	8.59E-13
ERBB4	rs4665972	2	27375230	C	T	0.61	0.06	0.01	2.86E-08
ERBB4	rs11453664	2	212538240	C	A	0.27	0.22	0.01	1.07E-67
ERBB4	rs140620086	3	98857467	T	C	0.05	-0.12	0.02	2.16E-05
ERBB4	-	3	186932142	C	G	0.62	0.07	0.01	5.18E-10
ERBB4	rs11757137	6	32552380	T	C	0.44	0.06	0.01	3.00E-07
ERBB4	rs34673027	7	129098971	A	C	0.45	-0.07	0.01	2.36E-11
ERBB4	rs2519093	9	133266456	T	C	0.18	-0.14	0.01	1.08E-22
ERBB4	rs56278466	10	17833858	G	T	0.66	0.11	0.01	1.73E-20
ERBB4	rs11220465	11	126387884	A	G	0.15	-0.07	0.01	1.70E-05
ERBB4	rs11830324	12	510452	A	T	0.17	-0.08	0.01	4.37E-08
ERBB4	rs7324484	13	48073943	A	G	0.66	0.05	0.01	1.38E-05
ERBB4	rs2541593	16	53423	A	C	0.81	0.07	0.01	8.81E-07
ERBB4	-	16	72045090	T	G	0.18	-0.08	0.01	2.31E-08
ERBB4	rs186021206	17	7166093	A	G	0.01	0.96	0.05	1.74E-38
ERBB4	rs72835417	17	49164280	A	G	0.11	0.15	0.01	1.70E-17
ERBB4	rs485073	19	48703998	G	A	0.54	0.10	0.01	6.21E-19
ERBIN	rs17622656	5	132485305	A	G	0.39	-0.05	0.01	2.60E-05
ERBIN	rs342293	7	106731773	G	C	0.46	-0.05	0.01	8.38E-06
ERBIN	rs10822145	10	63174788	T	C	0.47	0.05	0.01	2.57E-06
EREG	rs78803121	4	74382788	T	G	0.06	-0.19	0.02	4.51E-14
EREG	rs4572884	4	101862194	T	C	0.40	-0.05	0.01	1.65E-05
EREG	rs12655145	5	151455788	G	A	0.62	0.07	0.01	9.22E-10
EREG	rs2516471	6	31432515	A	G	0.06	-0.11	0.02	1.07E-05
EREG	rs6993770	8	105569300	T	A	0.29	-0.09	0.01	2.05E-13
EREG	rs10820606	9	96430637	C	A	0.23	-0.11	0.01	1.41E-14
EREG	rs7080386	10	63288546	A	C	0.41	0.07	0.01	6.01E-09
EREG	rs12445050	16	81837364	T	C	0.14	0.08	0.01	5.29E-06
EREG	rs892090	19	55027704	G	T	0.83	0.12	0.01	3.93E-14
EREG	rs6081569	20	19323142	C	G	0.37	0.05	0.01	9.79E-06
ERP44	rs764992666	1	230159329	T	C	0.60	-0.05	0.01	3.22E-06
ERP44	rs1260326	2	27508073	C	T	0.61	-0.06	0.01	1.03E-06
ERP44	rs7614709	3	186675997	A	G	0.22	-0.25	0.01	1.15E-80
ERP44	rs6993770	8	105569300	T	A	0.29	-0.06	0.01	8.98E-07
ERP44	rs188976415	9	100161776	A	C	0.00	-0.58	0.06	5.84E-10
ERP44	rs36115375	10	7735836	A	C	0.06	0.72	0.02	6.05E-215
ERP44	rs17580	14	94380925	A	T	0.05	-0.14	0.02	1.43E-07
ESAM	rs61747728	1	179557079	T	C	0.04	0.13	0.02	2.55E-05
ESAM	rs6961069	7	80589645	T	C	0.40	0.06	0.01	5.13E-07
ESAM	rs6993770	8	105569300	T	A	0.29	-0.10	0.01	3.21E-17
ESAM	rs8176672	9	133266772	T	C	0.06	0.20	0.02	1.13E-18
ESAM	rs774510679	10	63304076	A	C	0.41	0.09	0.01	1.08E-14
ESAM	rs61753651	11	124756673	A	G	0.00	-1.84	0.06	1.82E-96
ESAM	rs2127870	14	65330128	C	G	0.79	0.09	0.01	1.13E-09
ESAM	rs188942006	15	64711627	C	G	0.07	0.11	0.01	3.10E-06
ESAM	rs1654425	19	55027612	C	T	0.83	0.13	0.01	2.34E-18
ESM1	rs1260326	2	27508073	C	T	0.61	0.05	0.01	1.77E-05
ESM1	rs16822633	2	226782243	G	A	0.04	-0.12	0.02	1.26E-05

ESM1	rs557470468	3	52696168	C	T	0.01	0.45	0.05	4.05E-08
ESM1	rs4242051	5	54902947	C	T	0.75	0.19	0.01	5.80E-49
ESM1	rs3029083	5	72841120	C	T	0.53	0.05	0.01	1.90E-05
ESM1	rs10491244	5	82420208	A	G	0.21	-0.09	0.01	7.39E-10
ESM1	rs4959186	6	2477529	G	T	0.52	0.05	0.01	1.86E-05
ESM1	rs113760175	6	22343363	A	G	0.07	0.14	0.01	3.80E-10
ESM1	rs9269891	6	32583240	T	C	0.46	-0.06	0.01	4.56E-07
ESM1	rs1475718	9	134226324	A	G	0.44	-0.08	0.01	8.63E-13
ESM1	rs2393969	10	63380680	C	A	0.47	0.06	0.01	4.49E-08
ESM1	rs76895963	12	4275678	G	T	0.02	0.19	0.03	2.09E-05
ESM1	rs7310615	12	111427245	G	C	0.52	-0.07	0.01	2.32E-09
EZR	rs3734470	6	158766315	T	A	0.70	0.10	0.01	1.74E-14
EZR	rs7208422	17	78134494	T	A	0.48	-0.06	0.01	1.64E-06
EZR	rs10405357	19	54255803	C	T	0.44	-0.06	0.01	1.03E-07
F11R	rs60315407	1	161063015	C	T	0.29	0.07	0.01	2.92E-07
F11R	rs1354034	3	56815721	C	T	0.60	0.08	0.01	1.09E-10
F11R	rs3804749	3	123114156	T	C	0.59	-0.06	0.01	5.04E-06
F11R	rs11242109	5	132341354	T	G	0.48	-0.06	0.01	6.55E-08
F11R	rs17154155	7	80604927	T	G	0.41	0.05	0.01	2.61E-05
F11R	rs342293	7	106731773	G	C	0.46	-0.05	0.01	2.61E-05
F11R	rs6993770	8	105569300	T	A	0.29	-0.07	0.01	1.40E-07
F11R	rs409801	9	4744743	C	T	0.51	0.05	0.01	1.02E-05
F11R	rs10733789	10	63188924	C	T	0.31	0.06	0.01	4.13E-06
F11R	rs4608137	12	6184207	A	G	0.49	-0.06	0.01	2.49E-08
F11R	rs60822569	12	54323724	C	T	0.55	0.05	0.01	6.38E-06
F11R	rs1654425	19	55027612	C	T	0.83	0.08	0.01	1.27E-07
F2R	rs17200557	3	16936390	T	G	0.03	0.16	0.02	1.36E-06
F2R	rs1354034	3	56815721	C	T	0.60	0.09	0.01	9.13E-15
F2R	rs168753	5	76732299	T	A	0.17	-0.15	0.01	2.25E-24
F2R	rs778641135	6	135097494	T	C	0.26	0.06	0.01	9.88E-06
F2R	rs6993770	8	105569300	T	A	0.29	-0.08	0.01	1.64E-10
F2R	rs579459	9	133278724	C	T	0.21	-0.07	0.01	5.07E-06
F2R	rs7080386	10	63288546	A	C	0.41	0.11	0.01	3.75E-22
F2R	rs73396521	11	267568	A	G	0.25	0.06	0.01	6.84E-06
F2R	rs892090	19	55027704	G	T	0.83	0.08	0.01	2.10E-07
F3	rs6666213	1	94808809	G	A	0.37	0.18	0.01	2.85E-65
F3	rs780094	2	27518370	C	T	0.62	0.05	0.01	2.32E-06
F3	rs4458205	2	232692936	T	C	0.36	-0.05	0.01	2.06E-05
F3	rs2519093	9	133266456	T	C	0.18	-0.27	0.01	2.52E-93
F3	rs199881213	13	103048390	A	C	0.07	-0.11	0.01	1.76E-07
F3	rs488703	13	113116562	A	G	0.10	0.10	0.01	4.43E-09
F3	rs77924615	16	20381010	A	G	0.20	-0.06	0.01	1.90E-05
F3	rs200489612	17	7203059	A	G	0.01	0.47	0.05	1.58E-09
F3	rs35218105	17	30317759	A	C	0.32	0.08	0.01	2.83E-13
F3	rs2659005	17	81244914	T	C	0.44	0.05	0.01	1.53E-05
F3	rs516246	19	48702915	T	C	0.51	-0.28	0.01	4.98E-160
F7	rs1260326	2	27508073	C	T	0.61	-0.12	0.01	8.75E-31
F7	rs1154401	4	99088587	C	G	0.66	0.05	0.01	2.62E-06
F7	-	6	32489866	C	T	0.64	0.05	0.01	2.59E-05
F7	rs35732917	7	73598939	C	T	0.28	-0.06	0.01	4.39E-08
F7	rs7075901	10	63521234	A	C	0.47	-0.06	0.01	7.67E-08
F7	rs7232	11	60173126	A	T	0.37	0.06	0.01	3.22E-07
F7	rs510335	13	113105441	T	G	0.11	-0.77	0.01	0.00E+00
F7	rs28929474	14	94378610	T	C	0.02	0.16	0.02	2.24E-05
F7	rs540574262	20	35175899	T	G	0.09	0.22	0.01	4.36E-31
F9	rs1260326	2	27508073	C	T	0.61	-0.11	0.01	3.48E-20

F9	rs13226650	7	73602675	G	A	0.20	-0.07	0.01	2.40E-07
FABP1	rs2241883	2	88124547	C	T	0.31	-0.23	0.01	1.97E-84
FABP1	rs17655730	11	270715	C	T	0.25	0.06	0.01	2.38E-05
FABP2	rs1375131	2	135197227	C	T	0.27	0.06	0.01	1.14E-05
FABP2	rs17009129	4	119359462	T	C	0.33	-0.21	0.01	9.36E-71
FABP2	rs6472539	8	70717575	C	G	0.52	-0.07	0.01	2.52E-10
FABP2	rs17655730	11	270715	C	T	0.25	0.08	0.01	3.65E-10
FABP2	rs8111874	19	48665685	A	G	0.46	-0.06	0.01	1.85E-06
FABP4	rs7647481	3	12350314	A	G	0.12	0.10	0.01	2.38E-10
FABP4	rs112313579	8	81478948	G	T	0.16	-0.12	0.01	3.41E-16
FABP5	rs118019635	8	81281047	A	C	0.07	-0.20	0.01	2.71E-20
FABP5	rs6993770	8	105569300	T	A	0.29	-0.06	0.01	1.51E-05
FABP6	rs1130435	5	160232266	T	C	0.40	-0.13	0.01	2.46E-28
FABP6	rs5875434	6	33061456	T	C	0.55	-0.15	0.01	7.33E-40
FABP6	rs6472539	8	70717575	C	G	0.51	-0.21	0.01	1.96E-75
FABP6	rs35866622	19	48714803	T	C	0.50	-0.08	0.01	6.74E-11
FABP9	rs80293268	1	8147519	C	G	0.05	-0.12	0.02	1.59E-05
FABP9	rs72646785	1	17276977	T	G	0.17	0.16	0.01	1.55E-26
FABP9	rs36010924	1	152116368	G	A	0.18	0.37	0.01	1.43E-153
FABP9	rs774004	3	138458290	A	G	0.74	0.08	0.01	1.93E-10
FABP9	rs1344815	3	149623026	G	A	0.65	-0.05	0.01	1.36E-05
FABP9	rs1345417	3	181794163	G	C	0.60	0.07	0.01	1.64E-10
FABP9	rs57866025	3	190239580	T	C	0.27	0.06	0.01	4.07E-06
FABP9	rs2436396	5	140153978	G	A	0.70	-0.06	0.01	1.59E-07
FABP9	rs9263750	6	31146878	C	T	0.17	0.06	0.01	2.49E-05
FABP9	rs28497692	8	81500456	T	G	0.04	-0.23	0.02	6.34E-16
FABP9	rs541551288	8	116301979	A	G	0.20	-0.09	0.01	6.55E-12
FABP9	rs7905367	10	52574893	C	G	0.80	0.08	0.01	1.16E-09
FABP9	rs3809185	12	52553543	A	G	0.06	-0.20	0.01	2.21E-19
FABP9	rs12367299	12	90734068	A	G	0.27	0.06	0.01	1.31E-06
FABP9	rs150743616	17	3524287	T	C	0.00	0.87	0.09	2.26E-09
FABP9	rs3894193	17	39969093	G	C	0.47	-0.31	0.01	6.65E-176
FABP9	rs12457893	18	63258928	C	A	0.46	-0.07	0.01	6.90E-10
FABP9	rs867348	19	39196481	T	A	0.68	-0.09	0.01	7.41E-15
FADD	rs342298	7	106733200	T	C	0.46	-0.05	0.01	2.03E-05
FADD	rs4746204	10	63568758	T	C	0.48	-0.05	0.01	6.59E-06
FADD	rs10898847	11	70199816	A	G	0.41	0.08	0.01	1.06E-11
FADD	rs60822569	12	54323724	C	T	0.55	0.06	0.01	7.16E-08
FAM3B	rs3129777	6	32678407	A	C	0.21	-0.11	0.01	3.21E-16
FAM3B	rs2519093	9	133266456	T	C	0.18	-0.11	0.01	6.19E-15
FAM3B	rs2559850	12	101699681	A	G	0.59	0.06	0.01	2.01E-07
FAM3B	rs548921462	12	120958694	T	C	0.54	-0.05	0.01	5.11E-06
FAM3B	rs17743980	17	9890522	A	G	0.31	0.06	0.01	2.89E-07
FAM3B	rs708686	19	5840608	T	C	0.27	0.25	0.01	1.25E-98
FAM3B	rs602662	19	48703728	A	G	0.54	0.25	0.01	4.83E-120
FAM3B	rs57529409	21	41346335	T	C	0.05	-1.15	0.02	0.00E+00
FAM3C	rs36198735	7	121440138	C	A	0.73	-0.11	0.01	5.88E-19
FAM3C	rs36060036	16	20350628	T	C	0.17	-0.08	0.01	1.33E-07
FAM3C	rs151204724	17	76649472	G	T	0.35	0.06	0.01	1.33E-07
FAP	rs1751479	1	65550522	A	G	0.65	-0.06	0.01	4.45E-08
FAP	rs4664454	2	162214651	G	C	0.01	1.07	0.04	1.20E-83
FAP	rs9266252	6	31358045	A	G	0.70	0.12	0.01	6.38E-22
FAP	rs74044565	11	326384	C	G	0.14	0.08	0.01	3.18E-07
FAP	rs12908546	15	50702935	C	A	0.12	0.16	0.01	4.85E-20
FAP	rs740516	17	69086821	G	C	0.15	-0.15	0.01	5.57E-21
FAP	rs7256564	19	33398687	G	A	0.69	-0.05	0.01	1.63E-05

FAS	rs113705304	6	32622676	G	C	0.09	0.15	0.01	1.24E-16
FAS	rs10887882	10	89023165	A	T	0.58	-0.25	0.01	4.16E-126
FAS	rs123698	19	807442	C	G	0.60	0.05	0.01	7.17E-06
FAS	rs738409	22	43928847	G	C	0.22	0.06	0.01	1.83E-06
FASLG	rs79419452	1	172703333	A	T	0.16	-0.10	0.01	2.12E-12
FASLG	rs11127048	2	27529596	A	G	0.62	-0.05	0.01	2.76E-06
FASLG	rs11895938	2	68391950	G	A	0.71	-0.05	0.01	1.95E-05
FASLG	rs13063578	3	47046347	A	T	0.40	-0.17	0.01	1.59E-59
FASLG	rs13111860	4	141691726	G	A	0.71	-0.05	0.01	2.85E-06
FASLG	rs9259888	6	29930604	C	T	0.65	-0.08	0.01	1.75E-09
FASLG	rs117343599	8	38998228	C	G	0.02	-0.18	0.02	9.07E-07
FASLG	rs3927680	9	16887368	A	T	0.62	0.05	0.01	7.04E-07
FASLG	rs8177655	10	5973156	A	G	0.33	0.08	0.01	1.12E-11
FASLG	rs2646421	10	8425192	C	G	0.38	0.05	0.01	2.55E-06
FASLG	rs978522	10	88988581	C	T	0.40	0.29	0.01	5.27E-164
FASLG	rs745932882	10	92682235	A	G	0.46	0.11	0.01	1.01E-23
FASLG	rs648710	11	126306481	T	A	0.74	-0.11	0.01	8.32E-21
FASLG	rs11053802	12	10444608	T	C	0.69	-0.07	0.01	1.30E-09
FASLG	rs3184504	12	111446804	C	T	0.52	-0.16	0.01	1.16E-57
FASLG	rs748988	14	99319904	G	A	0.61	0.06	0.01	3.25E-09
FASLG	rs186021206	17	7166093	A	G	0.01	0.62	0.05	1.40E-18
FASLG	rs9916257	17	35470352	T	G	0.44	-0.12	0.01	2.04E-31
FASLG	rs150597688	17	39914833	T	C	0.50	0.05	0.01	3.04E-07
FASLG	rs2659005	17	81244914	T	C	0.44	0.06	0.01	9.45E-08
FASLG	rs1205340	20	34336065	A	G	0.50	0.07	0.01	1.73E-11
FASLG	rs35477491	21	13901496	T	C	0.22	-0.09	0.01	8.38E-10
FASLG	rs738722	22	28734024	C	T	0.70	0.05	0.01	3.45E-06
FASLG	rs739141	22	39428445	T	C	0.66	-0.06	0.01	3.27E-08
FBP1	rs499206	9	94597197	G	A	0.93	0.57	0.01	1.56E-158
FBP1	rs10883451	10	100164661	C	T	0.50	-0.06	0.01	3.70E-07
FBP1	rs145920606	12	7474715	G	A	0.08	0.09	0.01	5.38E-06
FBP1	rs10405357	19	54255803	C	T	0.44	-0.07	0.01	7.96E-11
FBP1	rs738409	22	43928847	G	C	0.22	0.07	0.01	1.41E-07
FCAR	rs188468174	1	24965206	T	C	0.01	-0.57	0.03	3.22E-45
FCAR	rs2070901	1	161215268	T	G	0.27	0.05	0.01	1.74E-06
FCAR	rs9862795	3	49878073	T	A	0.53	0.04	0.01	3.69E-06
FCAR	rs4012248	3	187061819	A	G	0.07	0.09	0.01	8.93E-06
FCAR	rs2853950	6	31268398	T	C	0.44	0.06	0.01	1.67E-09
FCAR	rs11155297	6	143503967	T	G	0.26	-0.05	0.01	1.20E-05
FCAR	rs6796	7	6462736	C	T	0.28	0.09	0.01	1.82E-18
FCAR	rs174575	11	61834531	G	C	0.28	0.05	0.01	2.73E-05
FCAR	rs73017385	11	126309680	A	G	0.24	0.09	0.01	1.25E-16
FCAR	rs3184504	12	111446804	C	T	0.52	-0.08	0.01	3.52E-18
FCAR	rs186021206	17	7166093	A	G	0.01	0.65	0.04	6.91E-24
FCAR	rs3826331	17	39994239	C	T	0.60	-0.08	0.01	1.88E-17
FCAR	rs1292061	17	59833869	G	A	0.56	0.04	0.01	9.60E-06
FCAR	rs12608573	19	54879918	G	C	0.22	0.84	0.01	0.00E+00
FCER2	rs1432296	2	60841032	T	C	0.14	0.07	0.01	1.57E-05
FCER2	rs17056278	5	158825430	G	C	0.08	-0.15	0.01	1.51E-13
FCER2	rs2844606	6	31280655	G	A	0.42	-0.09	0.01	5.00E-15
FCER2	rs658228	6	44627518	T	C	0.66	0.05	0.01	1.48E-05
FCER2	rs9488914	6	116369686	T	C	0.36	0.09	0.01	1.37E-16
FCER2	rs59082587	7	7837715	T	G	0.18	-0.06	0.01	1.10E-05
FCER2	rs2278467	8	24331331	C	T	0.16	-0.08	0.01	7.68E-07
FCER2	rs562240450	8	129592026	A	C	0.32	-0.06	0.01	1.95E-06
FCER2	-	10	61976858	C	T	0.65	-0.05	0.01	8.23E-06

FCER2	rs12784975	10	96620380	C	T	0.19	-0.08	0.01	6.96E-09
FCER2	rs111338191	12	111388673	T	A	0.52	-0.05	0.01	3.47E-06
FCER2	rs374039502	13	108308037	A	T	0.02	0.20	0.03	1.96E-06
FCER2	rs9939427	16	85982485	A	G	0.23	0.07	0.01	1.05E-07
FCER2	rs34557412	17	16948873	G	A	0.01	0.30	0.04	8.33E-06
FCER2	rs35222145	17	39851068	G	T	0.49	-0.09	0.01	1.00E-16
FCER2	rs2277989	19	7696926	G	A	0.49	-0.29	0.01	1.13E-149
FCER2	-	20	46107732	T	A	0.75	-0.09	0.01	5.99E-12
FCER2	rs75743846	22	41929013	A	G	0.02	-0.19	0.03	9.29E-06
FCGR2A	rs11810143	1	161510859	G	A	0.12	-1.46	0.01	0.00E+00
FCGR2A	rs62165726	2	134208991	A	C	0.04	-0.11	0.02	2.75E-06
FCGR2A	rs199922514	8	9325592	G	A	0.91	-0.07	0.01	2.00E-05
FCGR2A	rs782819119	9	133270584	A	T	0.31	0.05	0.01	1.25E-06
FCGR2A	rs75071241	11	126362291	A	G	0.06	-0.09	0.01	4.88E-06
FCGR2A	rs7137828	12	111494996	T	C	0.52	-0.06	0.01	2.36E-10
FCGR2A	rs186021206	17	7166093	A	G	0.01	1.08	0.04	1.26E-72
FCGR2B	rs1801274	1	161509955	G	A	0.53	-0.45	0.01	0.00E+00
FCGR2B	rs4240356	4	144121097	G	C	0.55	-0.05	0.01	1.47E-05
FCGR2B	rs505922	9	133273813	C	T	0.32	-0.18	0.01	4.05E-51
FCGR2B	rs6115999	20	3719094	T	C	0.01	0.31	0.03	4.93E-09
FCGR3B	-	1	156323264	C	T	0.50	0.08	0.01	2.84E-12
FCGR3B	rs200688856	1	161629989	C	G	0.34	-0.86	0.01	0.00E+00
FCGR3B	rs34860591	3	98888620	C	T	0.08	0.09	0.01	7.31E-07
FCGR3B	rs9274505	6	32666349	A	G	0.22	-0.06	0.01	3.85E-06
FCGR3B	rs6796	7	6462736	C	T	0.28	0.06	0.01	2.51E-08
FCGR3B	rs7804185	7	50313548	C	T	0.40	-0.05	0.01	7.91E-08
FCGR3B	rs73017399	11	126316417	C	G	0.24	0.20	0.01	4.66E-66
FCGR3B	rs3184504	12	111446804	C	T	0.52	-0.07	0.01	8.87E-14
FCGR3B	rs10138570	14	65393320	G	A	0.53	-0.07	0.01	1.17E-12
FCGR3B	rs10873558	14	105658834	G	C	0.49	-0.07	0.01	8.79E-10
FCGR3B	rs36082322	15	101209436	C	T	0.11	0.07	0.01	9.57E-06
FCGR3B	rs186021206	17	7166093	A	G	0.01	0.62	0.04	3.42E-20
FCGR3B	rs2241244	17	39997301	C	G	0.54	-0.08	0.01	5.27E-18
FCGR3B	rs4760	19	43648948	G	A	0.16	-0.27	0.01	3.27E-93
FCN2	rs72550870	1	11046609	C	T	0.03	-0.21	0.02	3.33E-14
FCN2	rs35677470	3	58197909	A	G	0.08	0.12	0.01	1.99E-12
FCN2	rs12496730	3	98688089	C	G	0.44	-0.04	0.01	9.45E-06
FCN2	rs11386832	5	96785666	A	T	0.74	0.14	0.01	8.47E-38
FCN2	rs111331197	6	7883235	T	C	0.01	0.27	0.03	7.02E-09
FCN2	rs2516501	6	31388821	T	C	0.26	0.05	0.01	8.45E-06
FCN2	rs7041446	9	134884208	A	G	0.46	-0.62	0.01	0.00E+00
FCN2	rs174533	11	61781553	A	G	0.35	0.05	0.01	9.82E-06
FCN2	rs1850521	11	114570873	T	G	0.13	-0.47	0.01	2.15E-238
FCN2	rs215225	12	481379	G	A	0.43	-0.04	0.01	1.48E-05
FCN2	rs7484541	12	57321020	T	A	0.23	-0.05	0.01	8.85E-06
FCRL1	rs4971154	1	157802090	T	C	0.50	-0.36	0.01	4.91E-230
FCRL1	rs2009581	2	111050100	A	G	0.27	-0.05	0.01	1.25E-05
FCRL1	rs34762068	3	128606920	A	G	0.27	0.05	0.01	1.60E-05
FCRL1	rs5001409	3	187017902	C	A	0.39	-0.06	0.01	8.19E-09
FCRL1	rs10002742	4	140099011	A	G	0.44	-0.05	0.01	2.64E-05
FCRL1	rs17361097	4	152080067	A	G	0.44	0.05	0.01	1.08E-06
FCRL1	-	5	88820075	T	A	0.31	0.07	0.01	3.92E-08
FCRL1	rs180798958	5	143809411	A	G	0.02	-0.19	0.03	9.07E-06
FCRL1	rs112824187	5	158838300	A	G	0.08	-0.16	0.01	2.75E-15
FCRL1	rs1226013	6	10539056	G	T	0.55	0.09	0.01	2.94E-18
FCRL1	rs2524072	6	31299079	T	C	0.52	-0.07	0.01	4.49E-09

FCRL1	rs658228	6	44627518	T	C	0.66	0.05	0.01	2.16E-05
FCRL1	rs73348121	7	50344289	C	T	0.02	-0.20	0.03	1.15E-05
FCRL1	rs1433577	8	129583635	A	G	0.27	-0.08	0.01	2.95E-11
FCRL1	-	9	133263362	G	A	0.18	0.06	0.01	1.24E-05
FCRL1	rs4245597	10	61966183	A	G	0.65	-0.11	0.01	3.38E-22
FCRL1	rs12784975	10	96620380	C	T	0.19	-0.07	0.01	8.13E-08
FCRL1	rs1035606	12	29356517	T	C	0.28	0.05	0.01	8.13E-06
FCRL1	rs76428106	13	28029870	C	T	0.01	0.29	0.03	5.08E-09
FCRL1	rs374039502	13	108308037	A	T	0.02	0.30	0.03	6.96E-14
FCRL1	rs9927316	16	85982795	G	C	0.24	0.09	0.01	1.04E-12
FCRL1	rs186021206	17	7166093	A	G	0.01	0.71	0.05	7.65E-22
FCRL1	rs34557412	17	16948873	G	A	0.01	0.67	0.04	1.88E-24
FCRL1	rs35222145	17	39851068	G	T	0.49	-0.08	0.01	1.19E-14
FCRL1	rs77542162	17	69085137	G	A	0.02	0.18	0.02	1.32E-06
FCRL1	rs73926277	19	33251720	T	C	0.34	0.07	0.01	2.74E-09
FCRL1	rs2297197	20	46044907	G	C	0.29	0.05	0.01	9.06E-06
FCRL1	rs73165110	22	41895852	A	G	0.08	-0.12	0.01	1.71E-09
FCRL2	rs188468174	1	24965206	T	C	0.01	-0.30	0.03	5.58E-10
FCRL2	rs12568320	1	157774214	G	A	0.17	0.76	0.01	0.00E+00
FCRL2	rs72832854	2	110833193	G	T	0.02	-0.14	0.02	1.57E-05
FCRL2	rs61696487	3	169943075	A	C	0.16	-0.07	0.01	7.40E-07
FCRL2	rs5001409	3	187017902	C	A	0.39	-0.07	0.01	1.72E-12
FCRL2	rs9295574	6	10531011	A	G	0.43	0.07	0.01	5.91E-11
FCRL2	rs114619738	6	29916867	C	T	0.01	1.10	0.03	2.17E-103
FCRL2	rs12213174	6	170273051	A	T	0.17	-0.06	0.01	1.25E-05
FCRL2	rs778224678	7	151256873	G	T	0.11	0.10	0.01	8.04E-10
FCRL2	rs12554596	9	37002145	G	A	0.20	-0.07	0.01	3.03E-09
FCRL2	rs10791824	11	65791795	G	A	0.57	0.06	0.01	3.36E-09
FCRL2	rs4766578	12	111466567	A	T	0.50	-0.05	0.01	1.80E-07
FCRL2	rs374039502	13	108308037	A	T	0.02	0.40	0.02	5.93E-28
FCRL2	rs186021206	17	7166093	A	G	0.01	0.61	0.04	8.13E-19
FCRL2	rs34557412	17	16948873	G	A	0.01	0.83	0.04	1.08E-42
FCRL2	rs79928920	17	46261601	C	T	0.23	-0.06	0.01	4.62E-06
FCRL2	rs112001035	17	68827664	A	G	0.06	0.41	0.01	1.18E-84
FCRL2	rs11085015	19	3369574	G	T	0.80	0.09	0.01	4.17E-13
FCRL2	rs73165110	22	41895852	A	G	0.08	-0.11	0.01	1.11E-08
FCRL3	rs7522061	1	157698600	C	T	0.48	0.96	0.01	0.00E+00
FCRL3	rs2009581	2	111050100	A	G	0.27	-0.04	0.01	1.32E-05
FCRL3	rs7137828	12	111494996	T	C	0.52	-0.04	0.01	2.51E-07
FCRL3	rs186021206	17	7166093	A	G	0.01	0.52	0.04	5.48E-19
FCRL3	rs34557412	17	16948873	G	A	0.01	0.35	0.03	4.87E-11
FCRL5	rs188468174	1	24965206	T	C	0.01	-0.53	0.03	9.61E-30
FCRL5	-	1	157543980	G	A	0.21	-0.49	0.01	4.65E-303
FCRL5	rs61804164	1	161653235	C	G	0.12	-0.08	0.01	2.18E-06
FCRL5	-	6	411448	A	G	0.06	-0.10	0.02	1.28E-05
FCRL5	rs9265986	6	31348504	T	C	0.32	-0.06	0.01	9.24E-09
FCRL5	rs7797255	7	50312008	G	A	0.40	-0.06	0.01	1.30E-07
FCRL5	-	11	128538755	T	C	0.52	0.05	0.01	8.07E-06
FCRL5	rs111338191	12	111388673	T	A	0.52	-0.06	0.01	1.22E-07
FCRL5	rs76428106	13	28029870	C	T	0.01	0.23	0.03	1.21E-06
FCRL5	rs3803286	14	102780133	G	A	0.67	-0.07	0.01	3.56E-09
FCRL5	rs11624491	14	105721708	T	C	0.66	0.17	0.01	1.36E-53
FCRL5	rs3803800	17	7559652	G	A	0.79	-0.07	0.01	5.70E-09
FCRL5	rs34562254	17	16939677	A	G	0.10	0.16	0.01	8.37E-21
FCRL5	-	17	46185215	C	A	0.17	-0.07	0.01	1.68E-05
FCRL5	rs754292	19	16589002	T	C	0.55	-0.04	0.01	2.40E-05

FCRL5	-	22	30037182	G	T	0.45	-0.06	0.01	1.15E-07
FCRL6	rs55650803	1	159813358	T	C	0.19	-0.89	0.01	0.00E+00
FCRL6	rs2549006	5	132491373	G	A	0.67	0.06	0.01	3.02E-08
FCRL6	rs9270747	6	32600515	G	A	0.66	0.12	0.01	2.02E-33
FCRL6	rs756658243	11	128189307	T	A	0.29	-0.08	0.01	2.13E-13
FCRL6	rs3184504	12	111446804	C	T	0.52	-0.08	0.01	4.54E-17
FCRL6	rs186021206	17	7166093	A	G	0.01	0.31	0.04	5.37E-06
FCRL6	rs58745116	17	35476573	A	G	0.39	-0.09	0.01	3.10E-18
FCRL6	-	17	81245700	A	G	0.44	0.04	0.01	1.33E-05
FCRLB	rs188468174	1	24965206	T	C	0.01	-0.31	0.03	7.11E-10
FCRLB	rs61801180	1	161718974	C	G	0.06	1.36	0.02	0.00E+00
FCRLB	rs1973612	4	186248013	T	C	0.51	-0.06	0.01	2.47E-10
FCRLB	rs7789913	7	50313099	T	C	0.40	0.06	0.01	2.20E-11
FCRLB	rs73169662	7	151225220	C	T	0.11	0.10	0.01	3.43E-12
FCRLB	rs7232	11	60173126	A	T	0.37	0.04	0.01	1.04E-05
FCRLB	rs186021206	17	7166093	A	G	0.01	0.33	0.04	3.19E-07
FEN1	rs10418046	19	53824615	G	T	0.21	0.06	0.01	7.21E-06
FES	rs1894401	15	90885812	A	G	0.52	0.13	0.01	6.75E-31
FETUB	rs1862069	2	169077231	A	G	0.54	-0.05	0.01	4.92E-06
FETUB	rs114780909	3	186642466	A	T	0.01	-2.13	0.04	8.63E-275
FETUB	rs28601761	8	125487789	G	C	0.42	-0.12	0.01	9.84E-27
FETUB	rs11438680	10	63414938	A	G	0.46	0.08	0.01	2.23E-13
FETUB	rs150844304	15	43434427	C	A	0.02	-0.27	0.02	1.00E-14
FGF19	rs3775076	4	39460353	A	G	0.36	0.10	0.01	4.95E-17
FGF19	rs4738684	8	58480714	G	A	0.67	0.10	0.01	2.82E-15
FGF19	rs8176741	9	133256074	A	G	0.06	0.17	0.02	7.80E-13
FGF19	rs708686	19	5840608	T	C	0.27	0.08	0.01	9.64E-09
FGF19	rs492602	19	48703160	G	A	0.51	-0.23	0.01	3.78E-87
FGF2	rs1434282	1	199041592	T	C	0.73	0.06	0.01	6.15E-07
FGF2	rs1354034	3	56815721	C	T	0.60	0.06	0.01	1.43E-07
FGF2	rs2922979	4	122827461	G	C	0.31	-0.71	0.01	0.00E+00
FGF2	rs6993770	8	105569300	T	A	0.29	-0.12	0.01	9.09E-28
FGF2	rs3780420	9	97914884	C	T	0.37	0.05	0.01	4.03E-07
FGF2	rs34416903	16	88492837	C	T	0.28	0.05	0.01	6.35E-06
FGF21	rs1260326	2	27508073	C	T	0.61	-0.14	0.01	6.31E-32
FGF21	rs1047891	2	210675783	A	C	0.31	-0.06	0.01	4.86E-07
FGF21	rs35332062	7	73597712	A	G	0.13	-0.16	0.01	2.06E-21
FGF21	rs3779195	7	98364050	A	T	0.18	-0.08	0.01	7.45E-08
FGF21	rs112875651	8	125494452	A	G	0.39	-0.06	0.01	1.22E-06
FGF21	rs62074055	17	47694567	C	G	0.50	-0.05	0.01	3.30E-06
FGF21	rs838131	19	48757420	C	A	0.46	-0.12	0.01	1.47E-26
FGF23	rs17199004	2	189588231	G	T	0.26	0.08	0.01	5.26E-09
FGF23	rs6862195	5	177395511	T	G	0.31	-0.07	0.01	1.43E-08
FGF23	rs34357864	9	133270497	A	G	0.32	0.09	0.01	4.51E-13
FGF23	rs6489536	12	4382743	C	G	0.34	0.11	0.01	9.37E-20
FGF23	rs6561643	13	32934941	T	A	0.35	0.08	0.01	2.20E-10
FGF23	rs6127099	20	54114863	T	A	0.28	-0.10	0.01	6.82E-14
FGF5	rs12509595	4	80261400	C	T	0.29	0.68	0.01	0.00E+00
FGF5	rs17580	14	94380925	A	T	0.05	0.11	0.01	4.52E-07
FGFBP1	rs7528419	1	109274570	G	A	0.22	-0.11	0.01	4.99E-16
FGFBP1	rs61816761	1	152313385	A	G	0.02	0.17	0.02	9.94E-06
FGFBP1	-	2	21043902	G	A	0.34	0.06	0.01	4.72E-07
FGFBP1	rs1260326	2	27508073	C	T	0.61	0.05	0.01	2.90E-06
FGFBP1	rs6800909	3	187783783	G	T	0.29	0.06	0.01	2.88E-07
FGFBP1	rs9968276	4	15947928	A	G	0.20	-0.12	0.01	1.41E-18
FGFBP1	rs9469004	6	31440119	C	T	0.15	0.07	0.01	3.30E-06

FGFBP1	rs66717685	6	148600815	G	C	0.17	0.07	0.01	2.69E-05
FGFBP1	rs3184504	12	111446804	C	T	0.52	0.06	0.01	3.03E-07
FGFBP1	rs1077834	15	58431280	C	T	0.22	0.06	0.01	3.65E-06
FGFBP1	rs77542162	17	69085137	G	A	0.02	0.17	0.02	3.95E-06
FGFBP1	rs151113958	19	11080525	G	A	0.12	-0.08	0.01	3.58E-06
FGFBP1	rs1065853	19	44909976	T	G	0.08	-0.28	0.01	9.30E-44
FGFBP1	rs2074735	22	31139886	C	G	0.06	0.14	0.01	3.93E-10
FGFR2	rs36086195	1	16184399	T	C	0.58	0.06	0.01	6.44E-08
FGFR2	rs6968170	7	73606346	A	G	0.28	-0.06	0.01	3.08E-06
FGFR2	rs7826120	8	58459166	C	T	0.67	0.06	0.01	1.55E-06
FGFR2	rs112875651	8	125494452	A	G	0.39	-0.06	0.01	2.90E-07
FGFR2	rs505922	9	133273813	C	T	0.32	0.05	0.01	2.36E-05
FGFR2	rs2981430	10	121552184	A	G	0.58	-0.11	0.01	5.72E-26
FGFR2	rs111632263	16	68199219	A	T	0.13	0.07	0.01	1.01E-05
FGFR2	rs186021206	17	7166093	A	G	0.01	0.53	0.05	1.07E-12
FGR	rs3737803	1	27634281	A	G	0.07	-0.18	0.01	3.21E-16
FHIT	rs1354034	3	56815721	C	T	0.60	0.07	0.01	3.05E-08
FHIT	rs13070475	3	59983610	A	G	0.64	0.09	0.01	8.03E-13
FIS1	rs1354034	3	56815721	C	T	0.60	0.06	0.01	3.65E-07
FIS1	rs7778271	7	101231073	C	T	0.05	-0.34	0.02	1.25E-43
FIS1	rs342293	7	106731773	G	C	0.46	-0.05	0.01	2.20E-05
FIS1	rs10733789	10	63188924	C	T	0.31	0.06	0.01	1.84E-06
FIS1	rs73396521	11	267568	A	G	0.25	0.06	0.01	1.42E-05
FKBP1B	rs872169	2	24036318	G	C	0.21	-0.25	0.01	2.08E-73
FKBP1B	rs1354034	3	56815721	C	T	0.60	0.08	0.01	3.82E-12
FKBP1B	rs342299	7	106733272	T	C	0.46	-0.07	0.01	4.32E-09
FKBP1B	rs3847326	10	63572099	G	A	0.48	-0.07	0.01	3.05E-10
FKBP1B	rs11502185	11	180258	C	T	0.26	0.07	0.01	1.82E-06
FKBP1B	rs200309755	17	29518756	T	C	0.54	0.07	0.01	1.68E-08
FKBP4	rs1354034	3	56815721	C	T	0.60	0.08	0.01	5.95E-13
FKBP4	rs342298	7	106733200	T	C	0.45	-0.06	0.01	1.55E-06
FKBP4	rs6993770	8	105569300	T	A	0.29	-0.06	0.01	2.09E-06
FKBP4	rs201265508	11	180231	G	A	0.29	0.07	0.01	2.41E-06
FKBP4	rs3814251	12	2794482	C	T	0.04	-0.14	0.02	8.03E-06
FKBP4	rs60822569	12	54323724	C	T	0.55	0.06	0.01	2.53E-07
FKBP4	rs10774624	12	111395984	A	G	0.51	-0.06	0.01	1.58E-06
FKBP5	rs1354034	3	56815721	C	T	0.60	0.05	0.01	5.39E-06
FKBP5	rs17622656	5	132485305	A	G	0.39	-0.05	0.01	9.60E-06
FKBP5	rs2817032	6	35720842	C	T	0.29	0.21	0.01	1.10E-63
FKBP5	rs342293	7	106731773	G	C	0.46	-0.05	0.01	8.45E-06
FKBP5	rs10822145	10	63174788	T	C	0.47	0.05	0.01	1.21E-05
FKBP7	-	2	178478419	C	T	0.00	-0.73	0.06	2.35E-13
FLI1	rs1354034	3	56815721	C	T	0.60	0.06	0.01	3.79E-07
FLI1	rs6993770	8	105569300	T	A	0.29	-0.07	0.01	3.80E-08
FLI1	rs12245149	10	63561387	A	C	0.49	-0.05	0.01	3.87E-06
FLRT2	rs3748770	1	81800220	A	G	0.27	0.25	0.01	2.29E-98
FLRT2	rs1260326	2	27508073	C	T	0.61	0.05	0.01	7.87E-07
FLRT2	rs10935473	3	98698056	T	G	0.44	-0.05	0.01	3.83E-07
FLRT2	rs6863407	5	167404689	T	C	0.72	0.06	0.01	8.81E-07
FLRT2	rs4841132	8	9326086	G	A	0.91	-0.11	0.01	7.75E-10
FLRT2	rs10901252	9	133252613	C	G	0.06	0.10	0.01	1.21E-05
FLRT2	-	10	63184239	A	T	0.44	0.05	0.01	7.83E-06
FLRT2	rs141826184	12	57184132	T	C	0.00	0.43	0.05	3.20E-08
FLRT2	rs17646457	14	85622971	A	G	0.15	-0.70	0.01	0.00E+00
FLRT2	rs77542162	17	69085137	G	A	0.02	0.22	0.02	4.40E-10
FLRT2	rs6135225	20	14697489	T	G	0.31	-0.19	0.01	4.43E-64

FLRT2	rs3747207	22	43928975	A	G	0.22	0.05	0.01	2.68E-05
FLT1	rs13088856	3	159837169	G	T	0.30	0.07	0.01	3.77E-08
FLT1	rs13107325	4	102267552	T	C	0.08	0.11	0.01	9.06E-07
FLT1	rs6921438	6	43957870	A	G	0.49	0.34	0.01	1.35E-204
FLT1	rs79220982	8	10749162	C	T	0.14	0.07	0.01	1.83E-05
FLT1	rs6993770	8	105569300	T	A	0.29	0.10	0.01	5.77E-16
FLT1	rs34881325	9	2622134	T	C	0.38	0.06	0.01	2.49E-07
FLT1	rs2519093	9	133266456	T	C	0.18	-0.18	0.01	9.07E-37
FLT1	rs552252905	13	28411879	G	C	0.00	-1.14	0.11	3.49E-11
FLT1	rs200489612	17	7203059	A	G	0.01	0.50	0.05	2.11E-09
FLT3	rs2736100	5	1286401	A	C	0.50	-0.05	0.01	1.30E-05
FLT3	rs62408224	6	90246276	G	A	0.35	-0.06	0.01	1.54E-06
FLT3	rs35350651	12	111469627	C	A	0.50	-0.07	0.01	1.58E-08
FLT3	rs12579868	12	120769344	T	C	0.47	-0.07	0.01	2.91E-10
FLT3	rs9554228	13	28063701	G	A	0.63	-0.08	0.01	1.25E-11
FLT3	rs186021206	17	7166093	A	G	0.01	0.63	0.05	4.86E-15
FLT3LG	rs17534670	2	65388294	A	G	0.49	0.09	0.01	5.02E-19
FLT3LG	rs2001703	3	128664254	G	A	0.62	-0.12	0.01	4.06E-27
FLT3LG	rs1344674	3	141406344	G	A	0.45	-0.06	0.01	2.90E-07
FLT3LG	rs218265	4	54542832	C	T	0.15	-0.08	0.01	4.85E-07
FLT3LG	-	4	104943372	A	T	0.03	-0.33	0.02	7.98E-27
FLT3LG	rs7705526	5	1285859	A	C	0.33	-0.12	0.01	5.10E-26
FLT3LG	rs56353819	6	90303148	T	C	0.35	0.07	0.01	3.30E-10
FLT3LG	rs9487052	6	109295883	T	C	0.46	0.07	0.01	1.38E-09
FLT3LG	rs42032	7	92608112	A	G	0.26	-0.06	0.01	5.83E-06
FLT3LG	rs2256187	11	78208722	A	G	0.15	-0.06	0.01	2.18E-05
FLT3LG	rs1800056	11	108267276	C	T	0.01	-0.25	0.03	3.11E-08
FLT3LG	rs4936290	11	114138533	C	A	0.35	0.06	0.01	3.15E-08
FLT3LG	rs7137828	12	111494996	T	C	0.52	0.05	0.01	6.33E-07
FLT3LG	rs76428106	13	28029870	C	T	0.01	1.31	0.03	7.50E-158
FLT3LG	rs137952017	14	100709753	T	C	0.15	0.07	0.01	2.71E-05
FLT3LG	rs4924248	15	38364053	T	C	0.76	-0.06	0.01	1.63E-05
FLT3LG	rs78378222	17	7668434	G	T	0.01	-0.22	0.03	1.16E-05
FLT3LG	rs80277818	18	44437936	G	A	0.13	-0.08	0.01	2.49E-06
FLT3LG	rs17758695	18	63253621	T	C	0.03	0.18	0.02	1.03E-08
FLT3LG	rs117955557	19	45252110	T	G	0.26	0.06	0.01	4.93E-06
FLT3LG	rs58527026	21	35036488	A	G	0.07	0.09	0.01	2.53E-05
FLT3LG	-	21	38503544	A	T	0.39	-0.05	0.01	2.60E-05
FLT3LG	rs62237617	22	28365160	T	C	0.00	-0.59	0.07	4.41E-07
FLT4	rs11162623	1	79093949	T	C	0.34	0.06	0.01	5.29E-10
FLT4	rs10935473	3	98698056	T	G	0.44	-0.69	0.01	0.00E+00
FLT4	rs34221241	5	180630293	C	T	0.11	-0.44	0.01	2.52E-173
FLT4	rs6913664	6	32328097	T	C	0.47	-0.06	0.01	1.77E-10
FLT4	rs60988380	9	33117956	T	C	0.10	0.08	0.01	1.06E-06
FLT4	rs10981199	9	112004087	C	T	0.36	0.05	0.01	7.71E-07
FLT4	rs2519093	9	133266456	T	C	0.18	-0.37	0.01	2.61E-195
FLT4	-	11	61774534	G	A	0.35	0.05	0.01	2.22E-07
FLT4	rs139130389	11	72139110	A	C	0.07	-0.14	0.01	1.64E-13
FLT4	rs186021206	17	7166093	A	G	0.01	0.49	0.04	1.07E-13
FLT4	rs1012383	17	61118704	T	G	0.83	-0.06	0.01	1.47E-05
FLT4	rs67525224	21	41255293	C	G	0.19	0.08	0.01	7.36E-11
FLT4	rs4611747	22	29361101	T	C	0.29	0.06	0.01	4.43E-08
FLT4	rs132653	22	36160775	T	G	0.82	0.06	0.01	3.07E-06
FOLR1	rs423144	1	155199564	T	G	0.43	0.06	0.01	5.97E-07
FOLR1	rs61747728	1	179557079	T	C	0.04	0.14	0.02	7.50E-07
FOLR1	rs13107325	4	102267552	T	C	0.08	0.10	0.01	1.01E-06

FOLR1	rs10740131	10	63511728	T	A	0.47	0.05	0.01	2.98E-06
FOLR1	rs115974400	11	72167818	C	T	0.07	-0.18	0.01	2.64E-16
FOLR1	rs777834943	16	20355409	T	C	0.18	-0.07	0.01	7.70E-06
FOLR1	rs186021206	17	7166093	A	G	0.01	0.72	0.05	5.28E-22
FOLR1	rs78060698	19	5832762	A	G	0.04	0.21	0.02	5.84E-14
FOLR1	rs681343	19	48703205	T	C	0.51	0.11	0.01	9.38E-25
FOLR2	rs333947	1	109928142	A	G	0.15	0.08	0.01	3.14E-07
FOLR2	rs1260326	2	27508073	C	T	0.61	0.06	0.01	8.64E-07
FOLR2	-	2	196764807	T	A	0.18	-0.08	0.01	1.43E-08
FOLR2	rs11918606	3	31004162	G	A	0.31	0.05	0.01	2.72E-05
FOLR2	rs2140574	3	99071378	T	C	0.62	-0.07	0.01	7.93E-10
FOLR2	rs509227	6	10535358	C	A	0.52	0.06	0.01	1.18E-07
FOLR2	rs80177780	6	32522644	A	C	0.75	0.11	0.01	5.27E-16
FOLR2	rs17138358	7	17880630	C	G	0.39	-0.06	0.01	7.58E-09
FOLR2	rs7865362	9	33117967	T	C	0.36	0.08	0.01	9.08E-13
FOLR2	rs2519093	9	133266456	T	C	0.18	0.06	0.01	2.49E-05
FOLR2	rs603424	10	100315722	A	G	0.17	-0.11	0.01	3.50E-13
FOLR2	rs950803	11	60385090	A	T	0.63	0.10	0.01	5.35E-20
FOLR2	rs146893981	11	72206309	T	C	0.03	0.32	0.02	4.97E-23
FOLR2	rs61729512	12	7485173	A	G	0.12	0.26	0.01	1.04E-53
FOLR2	-	12	120884101	C	A	0.69	-0.06	0.01	1.05E-05
FOLR2	rs4760	19	43648948	G	A	0.16	0.14	0.01	5.16E-22
FOLR2	rs12975366	19	54255498	C	T	0.40	-0.14	0.01	1.44E-36
FOLR3	rs333947	1	109928142	A	G	0.15	0.06	0.01	1.46E-05
FOLR3	-	2	196768245	G	C	0.20	-0.05	0.01	5.46E-06
FOLR3	rs62235167	3	31011191	G	C	0.31	0.04	0.01	1.08E-05
FOLR3	rs2470870	3	98976917	T	C	0.57	-0.05	0.01	7.35E-09
FOLR3	rs70993902	6	32627073	C	T	0.78	0.07	0.01	2.52E-11
FOLR3	rs10242866	7	17880990	T	C	0.39	-0.05	0.01	1.09E-07
FOLR3	rs2031902	9	33117526	T	C	0.59	-0.06	0.01	6.47E-11
FOLR3	rs603424	10	100315722	A	G	0.17	-0.07	0.01	2.36E-09
FOLR3	rs950802	11	60385111	A	G	0.31	0.08	0.01	3.85E-10
FOLR3	rs139130389	11	72139110	A	C	0.07	1.69	0.01	0.00E+00
FOLR3	rs61729512	12	7485173	A	G	0.12	0.16	0.01	6.39E-31
FOLR3	rs4760	19	43648948	G	A	0.16	0.08	0.01	3.31E-11
FOLR3	rs12975366	19	54255498	C	T	0.40	-0.09	0.01	6.41E-25
FOXO1	rs342298	7	106733200	T	C	0.46	-0.08	0.01	2.31E-13
FOXO1	rs10820606	9	96430637	C	A	0.23	-0.12	0.01	6.73E-19
FOXO1	rs4759076	12	54336088	C	T	0.46	0.07	0.01	5.43E-09
FOXO1	rs17061453	13	40610221	T	A	0.04	0.28	0.02	4.74E-24
FOXO3	rs74379225	6	32638965	G	A	0.06	0.12	0.02	3.80E-06
FRZB	rs13009	2	182834637	T	C	0.08	-0.82	0.01	0.00E+00
FRZB	rs11452310	3	12478870	A	T	0.57	0.05	0.01	9.83E-06
FRZB	rs2856694	6	32684504	C	T	0.19	-0.15	0.01	1.98E-28
FRZB	rs35350651	12	111469627	C	A	0.50	-0.05	0.01	1.38E-07
FRZB	rs6018652	20	47712230	A	G	0.79	-0.06	0.01	8.77E-06
FST	rs1260326	2	27508073	C	T	0.61	-0.11	0.01	1.55E-21
FST	rs31226	5	54031741	C	T	0.60	-0.14	0.01	2.65E-35
FST	rs55707100	15	43528519	T	C	0.02	0.17	0.02	4.33E-06
FSTL3	rs77924615	16	20381010	A	G	0.20	-0.08	0.01	3.50E-08
FSTL3	rs4919884	19	669546	C	T	0.84	0.11	0.01	7.53E-14
FUCA1	rs13551	1	23854472	C	T	0.34	-0.38	0.00	0.00E+00
FUCA1	-	4	6609511	T	G	0.18	0.15	0.01	9.81E-76
FUCA1	rs11155297	6	143503967	T	G	0.26	-1.29	0.01	0.00E+00
FUCA1	rs4764823	12	101825988	A	G	0.29	-0.09	0.00	5.48E-39
FUCA1	rs148815576	14	93219142	G	T	0.00	0.39	0.05	4.00E-07

FUCA1	rs339969	15	60591082	A	C	0.62	0.03	0.00	1.85E-05
FUCA1	-	19	19321481	A	G	0.07	0.07	0.01	3.68E-08
FURIN	rs1260326	2	27508073	C	T	0.61	-0.10	0.01	7.47E-18
FURIN	rs34430945	7	73629335	C	T	0.13	-0.08	0.01	5.09E-06
FURIN	rs6227	15	90882002	T	C	0.33	0.20	0.01	7.53E-62
FUS	rs1354034	3	56815721	C	T	0.60	0.05	0.01	6.18E-06
FUS	rs10418046	19	53824615	G	T	0.21	0.10	0.01	5.14E-13
FUT3_FUT5	rs5860563	4	99126006	A	C	0.70	-0.06	0.01	1.37E-09
FUT3_FUT5	rs2106854	5	132433482	T	C	0.19	-0.05	0.01	2.71E-05
FUT3_FUT5	rs34434863	6	32591896	G	T	0.31	0.05	0.01	2.73E-06
FUT3_FUT5	rs28601761	8	125487789	G	C	0.42	0.07	0.01	7.37E-13
FUT3_FUT5	rs62542743	9	33385243	A	C	0.05	0.10	0.01	4.98E-06
FUT3_FUT5	-	10	5204247	A	C	0.14	0.06	0.01	8.86E-06
FUT3_FUT5	rs56278466	10	17833858	G	T	0.66	0.07	0.01	6.98E-13
FUT3_FUT5	rs9738365	12	31844701	A	C	0.27	0.09	0.01	5.78E-16
FUT3_FUT5	rs112158477	12	111328642	T	C	0.15	0.06	0.01	1.30E-05
FUT3_FUT5	rs2393791	12	120986153	T	C	0.62	0.14	0.01	3.86E-43
FUT3_FUT5	rs178765	14	23237529	A	G	0.28	0.07	0.01	7.66E-10
FUT3_FUT5	rs11868058	17	47563039	T	G	0.51	-0.04	0.01	1.52E-05
FUT3_FUT5	rs3760775	19	5841345	T	G	0.06	-1.06	0.01	0.00E+00
FUT3_FUT5	rs58542926	19	19268740	T	C	0.08	0.11	0.01	8.18E-08
FUT3_FUT5	rs492602	19	48703160	G	A	0.51	-0.08	0.01	6.44E-14
FUT3_FUT5	rs6073431	20	44411929	T	C	0.53	0.05	0.01	4.89E-06
FUT8	rs7618405	3	18209017	A	C	0.21	-0.06	0.01	1.03E-05
FUT8	rs1354034	3	56815721	C	T	0.60	-0.06	0.01	8.49E-10
FUT8	rs3804753	3	123132993	G	A	0.59	-0.05	0.01	7.12E-06
FUT8	rs13434472	4	101878353	A	C	0.40	-0.05	0.01	5.08E-07
FUT8	rs10075801	5	132341949	G	A	0.49	-0.05	0.01	8.92E-07
FUT8	rs138614581	6	31436393	T	A	0.08	-0.08	0.01	1.20E-05
FUT8	rs6961069	7	80589645	T	C	0.40	0.05	0.01	4.37E-07
FUT8	rs6993770	8	105569300	T	A	0.29	-0.15	0.01	1.00E-41
FUT8	-	10	63433569	C	T	0.49	-0.06	0.01	4.69E-10
FUT8	rs71474568	10	102589091	G	T	0.34	0.06	0.01	1.27E-07
FUT8	rs10774625	12	111472415	G	A	0.50	-0.04	0.01	2.41E-05
FUT8	rs2127870	14	65330128	C	G	0.80	-0.91	0.01	0.00E+00
FUT8	rs12445050	16	81837364	T	C	0.14	0.09	0.01	1.12E-09
FUT8	rs892090	19	55027704	G	T	0.83	0.13	0.01	5.05E-22
FUT8	rs6112319	20	19336421	A	G	0.35	0.05	0.01	5.83E-07
FXN	rs1354034	3	56815721	C	T	0.60	0.06	0.01	1.94E-06
FXN	rs6993770	8	105569300	T	A	0.29	-0.06	0.01	2.08E-06
FXN	rs112450466	9	69215313	A	T	0.38	0.08	0.01	6.35E-11
FXN	rs10733789	10	63188924	C	T	0.31	0.06	0.01	8.51E-06
FXN	rs60822569	12	54323724	C	T	0.55	0.06	0.01	1.65E-06
FXYD5	rs1354034	3	56815721	C	T	0.60	0.08	0.01	3.57E-11
FXYD5	rs7896518	10	63344740	G	A	0.42	0.06	0.01	2.38E-06
FXYD5	rs756845	19	35172347	G	A	0.78	-0.38	0.01	1.84E-160
FYB1	rs1354034	3	56815721	C	T	0.60	0.07	0.01	3.19E-10
FYB1	rs112838960	5	132373340	A	T	0.52	0.06	0.01	7.13E-08
FYB1	rs892090	19	55027704	G	T	0.83	0.07	0.01	2.29E-05
GAL	rs320366	1	117585523	T	C	0.75	-0.06	0.01	6.44E-07
GAL	rs760077	1	155208991	T	A	0.60	-0.08	0.01	1.20E-13
GAL	rs28631481	1	218678805	T	C	0.38	0.05	0.01	8.21E-06
GAL	rs11680123	2	60287613	G	A	0.59	0.19	0.01	3.11E-72
GAL	rs121908120	2	218890289	A	T	0.03	-0.25	0.02	4.18E-14
GAL	rs1530018	2	224060404	G	A	0.23	-0.08	0.01	1.35E-09
GAL	rs3773364	3	12148468	G	A	0.16	0.20	0.01	5.49E-43

GAL	rs6820414	4	38357645	G	C	0.59	-0.05	0.01	9.72E-06
GAL	rs17134145	6	1670090	A	C	0.11	0.14	0.01	5.32E-17
GAL	rs560623757	6	36749100	T	C	0.10	0.12	0.01	3.74E-11
GAL	rs13284054	9	104906792	C	T	0.12	-0.07	0.01	2.04E-05
GAL	rs7070183	10	95219370	T	A	0.47	0.05	0.01	1.64E-05
GAL	rs565427	11	65765354	A	G	0.36	0.06	0.01	9.50E-09
GAL	rs3018721	11	68656221	G	A	0.08	0.15	0.01	9.26E-14
GAL	rs2540119	12	4031710	C	T	0.76	-0.06	0.01	3.51E-06
GAL	rs9601479	13	80868601	A	G	0.51	0.05	0.01	6.39E-06
GAL	rs36034702	15	90191395	T	C	0.17	-0.09	0.01	3.26E-09
GAL	rs720130	16	11038776	T	G	0.41	-0.06	0.01	3.01E-07
GAL	rs56156922	16	56953457	C	T	0.32	0.07	0.01	1.34E-10
GAL	rs77542162	17	69085137	G	A	0.02	0.73	0.02	9.71E-94
GAL	rs55949690	17	72058283	A	G	0.52	0.05	0.01	8.86E-06
GAL	rs1884371	20	39524198	C	G	0.55	0.05	0.01	3.53E-06
GAL	rs6024978	20	56590011	C	A	0.23	-0.07	0.01	3.05E-07
GALNT10	rs4672497	2	62296430	G	C	0.22	0.07	0.01	3.20E-07
GALNT10	rs890793	5	154194378	T	C	0.38	0.38	0.01	3.55E-246
GALNT10	-	9	133276163	T	A	0.19	0.26	0.01	1.86E-77
GALNT10	rs56278466	10	17833858	G	T	0.66	0.06	0.01	3.19E-07
GALNT10	rs2695294	12	101693236	A	G	0.58	0.06	0.01	1.39E-07
GALNT10	rs3184504	12	111446804	C	T	0.52	-0.05	0.01	5.17E-07
GALNT10	rs145078947	14	93186629	T	G	0.00	0.56	0.07	1.16E-07
GALNT10	rs704	17	28367840	A	G	0.47	0.09	0.01	3.11E-16
GALNT10	rs601338	19	48703417	A	G	0.51	0.05	0.01	4.62E-06
GALNT10	rs892090	19	55027704	G	T	0.83	0.07	0.01	5.07E-06
GALNT2	rs4846914	1	230159944	A	G	0.61	0.29	0.01	2.47E-153
GALNT2	rs9273216	6	32645969	A	C	0.55	-0.05	0.01	2.94E-06
GALNT2	rs9987289	8	9325848	G	A	0.91	-0.08	0.01	8.20E-06
GALNT2	rs12359178	10	17847614	A	G	0.14	0.07	0.01	5.27E-06
GALNT2	rs201220977	10	63305512	T	C	0.47	0.05	0.01	2.77E-05
GALNT2	rs8181612	12	89510921	A	T	0.71	-0.13	0.01	2.00E-27
GALNT2	-	16	72045090	T	G	0.18	0.32	0.01	7.19E-113
GALNT2	rs704	17	28367840	A	G	0.47	0.08	0.01	1.66E-14
GALNT2	rs12975366	19	54255498	C	T	0.40	-0.15	0.01	1.89E-41
GALNT3	rs2116546	2	165867023	C	T	0.30	-0.39	0.01	3.42E-216
GALNT3	rs9310400	3	12247950	T	C	0.33	0.05	0.01	7.72E-06
GALNT3	rs13107325	4	102267552	T	C	0.07	-0.12	0.01	4.74E-08
GALNT3	rs9391787	6	32480839	C	T	0.59	0.06	0.01	6.52E-07
GALNT3	rs7791957	7	7230403	C	T	0.43	0.09	0.01	1.26E-17
GALNT3	rs782134971	9	133264504	G	C	0.25	0.31	0.01	1.02E-132
GALNT3	rs3184504	12	111446804	C	T	0.52	-0.08	0.01	8.92E-13
GALNT3	rs145078947	14	93186629	T	G	0.00	0.52	0.07	1.66E-06
GALNT3	rs535064984	17	7116978	C	T	0.01	0.35	0.05	1.35E-06
GALNT3	rs492602	19	48703160	G	A	0.51	-0.18	0.01	4.05E-62
GALNT7	rs13107325	4	102267552	T	C	0.08	-0.14	0.01	1.77E-10
GALNT7	rs144873913	4	173314153	A	C	0.00	-0.87	0.07	6.93E-14
GALNT7	rs11755689	6	32618599	G	A	0.33	0.07	0.01	2.42E-09
GALNT7	rs1404422	7	17795362	C	A	0.51	-0.06	0.01	7.61E-07
GALNT7	rs117905808	7	100911510	A	G	0.02	0.72	0.03	5.06E-70
GALNT7	rs139130389	11	72139110	A	C	0.07	0.12	0.01	1.08E-07
GALNT7	rs11054859	12	7617180	A	G	0.10	0.15	0.01	3.03E-16
GALNT7	rs534297892	12	120911539	C	A	0.50	0.05	0.01	7.67E-06
GALNT7	rs9534300	13	46055090	T	A	0.71	-0.12	0.01	5.99E-22
GALNT7	rs12975366	19	54255498	C	T	0.39	-0.22	0.01	1.64E-80
GAS6	rs56073752	2	9477232	A	G	0.51	0.05	0.01	1.17E-05

GAS6	rs1260326	2	27508073	C	T	0.61	0.06	0.01	4.19E-07
GAS6	rs1257168	2	134206321	G	A	0.50	-0.05	0.01	2.48E-05
GAS6	rs1983009	3	98839204	T	C	0.10	0.10	0.01	1.40E-07
GAS6	rs13108218	4	3442204	G	A	0.62	0.05	0.01	1.12E-05
GAS6	rs149893883	4	98980406	T	C	0.67	-0.05	0.01	5.06E-06
GAS6	rs112840324	6	32510881	G	A	0.71	0.08	0.01	1.67E-08
GAS6	rs17138358	7	17880630	C	G	0.39	-0.05	0.01	2.17E-06
GAS6	rs17145750	7	73612048	T	C	0.16	0.07	0.01	1.37E-06
GAS6	rs12667978	7	129137407	T	C	0.57	0.06	0.01	1.92E-07
GAS6	rs4841132	8	9326086	G	A	0.91	-0.13	0.01	8.73E-12
GAS6	rs4876611	8	115659621	G	A	0.72	0.07	0.01	1.15E-09
GAS6	rs11230313	11	60436568	C	A	0.33	-0.05	0.01	1.17E-05
GAS6	rs6602909	13	113849020	C	T	0.32	-0.34	0.01	9.53E-183
GAS6	rs150844304	15	43434427	C	A	0.02	-0.16	0.02	5.01E-06
GAS6	rs186021206	17	7166093	A	G	0.01	1.07	0.05	6.54E-48
GAS6	rs897764	19	35068720	C	T	0.94	-0.11	0.01	2.40E-06
GAS6	rs66841352	19	41233275	C	G	0.40	-0.22	0.01	1.79E-87
GAS6	rs3747207	22	43928975	A	G	0.22	0.08	0.01	4.05E-09
GBP2	rs7531439	1	89088541	C	A	0.79	0.12	0.01	7.27E-18
GBP4	rs608339	1	89186395	T	A	0.51	-0.81	0.01	0.00E+00
GCG	rs1260326	2	27508073	C	T	0.61	-0.05	0.01	1.83E-05
GCG	rs17145750	7	73612048	T	C	0.16	-0.09	0.01	9.96E-08
GCG	rs39275	7	90145856	G	A	0.53	0.05	0.01	7.02E-06
GCG	rs964184	11	116778201	C	G	0.87	-0.08	0.01	8.95E-06
GCG	rs72654473	19	44911142	A	C	0.11	0.09	0.01	6.24E-07
GCNT1	rs2009581	2	111050100	A	G	0.27	-0.06	0.01	9.57E-07
GCNT1	rs35829610	5	132488170	G	C	0.34	-0.06	0.01	1.48E-08
GCNT1	rs2282683	9	76502835	G	A	0.21	-0.49	0.01	1.27E-298
GCNT1	rs505922	9	133273813	C	T	0.32	0.07	0.01	8.84E-10
GCNT1	rs561088406	10	17770126	A	G	0.09	0.10	0.01	1.25E-06
GCNT1	rs61377406	11	122664422	T	G	0.36	-0.05	0.01	1.55E-05
GCNT1	rs3184504	12	111446804	C	T	0.52	-0.13	0.01	7.79E-34
GCNT1	rs492602	19	48703160	G	A	0.51	-0.07	0.01	2.70E-11
GDF15	rs1054221	19	18389048	C	T	0.15	0.39	0.01	1.51E-185
GDF2	rs34008398	2	68866281	A	G	0.01	-0.24	0.03	4.86E-07
GDF2	rs68066031	2	224015781	C	T	0.23	0.16	0.01	1.18E-39
GDF2	rs66530140	4	186240057	C	T	0.51	0.09	0.01	2.89E-18
GDF2	rs78408340	5	103003035	G	C	0.01	-0.30	0.03	2.83E-08
GDF2	rs10820966	9	92293147	T	A	0.33	-0.18	0.01	5.81E-57
GDF2	rs9414801	10	63389329	A	G	0.53	-0.06	0.01	5.92E-09
GDF2	rs67852477	10	69097207	G	C	0.01	0.32	0.03	1.00E-09
GDF2	rs12284180	11	126395606	C	A	0.25	0.05	0.01	2.19E-05
GDF2	rs3184504	12	111446804	C	T	0.52	0.05	0.01	5.70E-06
GDF2	rs7152610	14	94462135	C	T	0.85	-0.15	0.01	3.89E-25
GDF2	rs4702	15	90883330	A	G	0.56	0.06	0.01	8.09E-08
GDF2	rs12441560	15	101445718	A	G	0.27	0.06	0.01	5.00E-07
GDF2	rs77303550	16	72045758	T	C	0.20	-0.07	0.01	8.76E-08
GDF2	rs186021206	17	7166093	A	G	0.01	0.48	0.05	4.53E-11
GDNF	rs62360373	5	37820639	A	G	0.10	-0.39	0.01	7.46E-94
GDNF	rs4808305	19	14690145	C	T	0.32	0.07	0.01	6.01E-08
GFAP	rs150394890	1	223676678	T	G	0.01	-0.46	0.04	1.73E-15
GFAP	rs17737955	2	86280891	T	C	0.40	0.05	0.01	9.49E-06
GFAP	rs147795230	3	81641847	C	T	0.00	1.30	0.11	2.25E-14
GFAP	rs5011436	7	12229132	C	A	0.42	-0.06	0.01	5.37E-08
GFAP	rs5893529	8	100664952	T	A	0.46	0.05	0.01	4.08E-07
GFAP	rs7114754	11	65203925	C	T	0.08	-0.14	0.01	8.32E-13

GFAP	rs59291670	17	44914081	T	C	0.02	0.28	0.02	7.14E-15
GFAP	rs429358	19	44908684	C	T	0.16	0.10	0.01	9.77E-13
GFER	rs61516948	16	1973321	T	C	0.13	-0.10	0.01	4.83E-09
GFRA1	rs10982156	9	114325784	A	T	0.07	0.18	0.01	7.37E-17
GFRA1	rs11197603	10	116242259	C	T	0.15	-0.31	0.01	3.55E-90
GFRA1	rs6041	13	113118393	A	G	0.10	-0.21	0.01	1.03E-33
GFRA1	rs34262842	16	20344489	G	A	0.17	-0.07	0.01	2.38E-06
GFRA2	rs13107325	4	102267552	T	C	0.08	0.11	0.01	7.83E-07
GFRA2	rs115216147	5	71369699	A	T	0.07	0.10	0.01	1.18E-05
GFRA2	rs9275544	6	32708576	G	A	0.23	-0.09	0.01	5.66E-11
GFRA2	rs1480999	8	21616577	A	G	0.56	0.31	0.01	1.10E-154
GFRA2	rs603424	10	100315722	A	G	0.17	-0.07	0.01	1.45E-05
GFRA2	rs186021206	17	7166093	A	G	0.01	0.82	0.05	2.51E-27
GFRA2	rs10405357	19	54255803	C	T	0.44	-0.05	0.01	7.38E-06
GFRA3	rs12027119	1	59687013	G	A	0.67	-0.05	0.01	5.97E-06
GFRA3	rs35383942	1	201468704	T	C	0.06	0.11	0.01	1.96E-06
GFRA3	rs12710696	2	19121042	C	T	0.64	-0.06	0.01	3.31E-07
GFRA3	rs73158489	3	154040073	T	C	0.16	0.08	0.01	2.73E-07
GFRA3	rs13107325	4	102267552	T	C	0.07	0.14	0.01	4.66E-11
GFRA3	rs12654231	5	110341005	G	A	0.04	0.16	0.02	1.96E-08
GFRA3	rs56259296	5	138144920	A	T	0.37	0.09	0.01	4.34E-13
GFRA3	rs62405538	6	10324463	T	A	0.21	-0.06	0.01	6.08E-06
GFRA3	rs10956755	8	134290713	G	A	0.30	-0.05	0.01	1.44E-05
GFRA3	rs56278466	10	17833858	G	T	0.66	0.08	0.01	4.11E-12
GFRA3	rs10887793	10	88231148	T	G	0.45	0.06	0.01	1.94E-08
GFRA3	rs10840586	11	11148261	G	A	0.37	0.05	0.01	2.35E-05
GFRA3	rs10742826	11	48071893	T	C	0.68	0.06	0.01	4.73E-07
GFRA3	rs78689694	11	126364925	C	G	0.13	-0.08	0.01	3.70E-06
GFRA3	rs11830324	12	510452	A	T	0.17	-0.14	0.01	4.75E-21
GFRA3	rs1161106	12	67446687	A	C	0.12	-0.08	0.01	1.03E-05
GFRA3	rs1618875	12	115306576	T	C	0.49	0.21	0.01	1.27E-81
GFRA3	-	15	38081910	G	T	0.88	-0.09	0.01	1.68E-07
GFRA3	rs2306333	15	50925477	G	A	0.17	0.07	0.01	3.05E-06
GFRA3	rs186021206	17	7166093	A	G	0.01	0.54	0.05	1.18E-12
GFRA3	rs11655511	17	61209908	T	C	0.77	0.10	0.01	2.02E-15
GFRA3	rs3744052	17	76561975	T	C	0.21	0.07	0.01	1.73E-07
GFRA3	rs554743	20	3681495	T	C	0.69	-0.05	0.01	9.28E-06
GGH	rs114165349	1	26695422	C	G	0.02	0.20	0.02	5.82E-08
GGH	-	3	136181921	A	T	0.70	0.06	0.01	7.52E-08
GGH	rs451637	6	32027005	T	C	0.31	-0.06	0.01	3.77E-07
GGH	rs1513275	7	28219614	C	T	0.19	0.07	0.01	2.28E-06
GGH	rs74329104	8	63018637	A	G	0.07	0.72	0.01	5.21E-222
GGH	rs6488368	12	7508506	G	A	0.97	-0.24	0.02	6.64E-13
GGH	rs10778152	12	101831973	G	A	0.29	-0.25	0.01	1.72E-100
GGH	rs117230571	13	41114931	G	A	0.08	0.10	0.01	3.26E-06
GGH	rs139974673	15	43735687	C	T	0.02	0.16	0.02	3.24E-06
GGH	rs768736886	16	53459434	A	T	0.30	0.07	0.01	1.19E-08
GGH	rs199737620	17	46137699	A	T	0.20	0.07	0.01	9.44E-07
GGH	rs12970396	18	62441936	A	G	0.30	0.06	0.01	3.09E-06
GGH	rs58542926	19	19268740	T	C	0.08	0.10	0.01	3.16E-06
GGH	rs12986064	19	54251270	C	T	0.51	-0.10	0.01	1.38E-19
GGT1	rs924204	1	16187431	G	A	0.59	0.07	0.01	7.02E-10
GGT1	rs4665972	2	27375230	C	T	0.61	-0.05	0.01	1.53E-05
GGT1	rs4835265	4	145900258	A	C	0.16	0.09	0.01	2.83E-10
GGT1	rs11426880	11	58618576	T	A	0.24	-0.05	0.01	2.43E-05
GGT1	rs7970695	12	120985573	A	G	0.62	0.13	0.01	2.53E-35

GGT1	rs59643720	14	103098470	C	A	0.24	0.07	0.01	1.43E-09
GGT1	rs4998040	18	58426767	A	G	0.79	-0.06	0.01	6.34E-07
GGT1	rs2006227	22	24599789	A	C	0.34	0.25	0.01	1.18E-118
GGT5	rs74612335	11	126368738	C	T	0.13	-0.10	0.01	2.73E-10
GGT5	rs186021206	17	7166093	A	G	0.01	0.64	0.05	1.21E-16
GGT5	rs75016991	17	69428367	A	C	0.03	0.16	0.02	8.99E-06
GGT5	rs5760275	22	24247481	G	C	0.21	0.33	0.01	3.61E-122
GHRL	rs751198981	2	221201933	T	G	0.20	-0.08	0.01	1.27E-07
GHRL	rs34911341	3	10289835	T	C	0.01	1.39	0.05	3.71E-87
GHRL	rs7438696	4	26517243	T	G	0.28	0.07	0.01	5.94E-08
GHRL	rs10470729	4	71041953	T	C	0.73	0.07	0.01	4.22E-08
GHRL	rs11745870	5	170195796	T	C	0.31	-0.08	0.01	2.29E-10
GHRL	rs868223	8	20293584	A	G	0.67	0.05	0.01	1.55E-05
GHRL	rs11786721	8	142678761	C	T	0.41	0.07	0.01	8.49E-10
GHRL	rs4500149	9	110707168	A	G	0.42	0.06	0.01	2.34E-08
GHRL	rs8176746	9	133255935	T	G	0.06	-0.16	0.01	5.11E-12
GHRL	rs558760274	17	336083	C	A	0.79	0.06	0.01	7.83E-06
GHRL	rs17138478	17	37713312	A	C	0.13	0.08	0.01	2.06E-06
GHRL	rs34954997	19	44914381	G	C	0.24	0.07	0.01	4.43E-07
GHRL	rs6129534	20	40256219	T	G	0.30	0.07	0.01	2.23E-09
GKN1	rs760077	1	155208991	T	A	0.60	-0.05	0.01	2.39E-05
GKN1	rs71469854	11	1193912	T	C	0.18	0.09	0.01	1.08E-08
GKN1	rs601338	19	48703417	A	G	0.51	-0.08	0.01	5.72E-13
GKN1	rs4816636	21	39654583	G	T	0.75	0.06	0.01	3.51E-06
GLB1	rs114165349	1	26695422	C	G	0.02	0.17	0.02	3.33E-06
GLB1	rs7637133	3	33097271	A	C	0.62	0.23	0.01	7.02E-93
GLB1	rs687339	3	136213517	T	C	0.77	0.07	0.01	8.34E-08
GLB1	rs76778371	6	159997018	A	G	0.09	-0.09	0.01	7.36E-06
GLB1	rs1293303	8	11862718	C	G	0.45	0.07	0.01	1.30E-11
GLB1	rs10778152	12	101831973	G	A	0.29	-0.29	0.01	6.52E-125
GLB1	rs150641790	13	41113708	G	T	0.07	0.10	0.01	9.31E-07
GLB1	rs145078947	14	93186629	T	G	0.00	0.70	0.07	4.93E-11
GLB1	-	17	46143803	C	A	0.28	0.06	0.01	2.66E-06
GLB1	rs58542926	19	19268740	T	C	0.08	0.19	0.01	3.31E-21
GLB1	rs142042446	19	44883210	A	G	0.15	-0.07	0.01	9.98E-06
GLO1	rs4746	6	38682852	G	T	0.46	-0.33	0.01	4.36E-187
GLOD4	rs1354034	3	56815721	C	T	0.60	0.06	0.01	1.56E-06
GLOD4	rs6993770	8	105569300	T	A	0.29	-0.06	0.01	1.74E-05
GLOD4	rs150230220	10	63142032	T	A	0.31	0.06	0.01	6.88E-07
GLRX	rs6556886	5	95827745	A	G	0.13	-0.74	0.01	0.00E+00
GLRX	rs11759553	6	135101158	T	A	0.27	-0.06	0.01	2.17E-08
GLT8D2	rs117801489	12	104015054	C	T	0.02	0.68	0.03	1.68E-51
GMPR	rs1042391	6	16290530	A	T	0.38	0.25	0.01	8.95E-110
GMPR	rs342293	7	106731773	G	C	0.46	-0.05	0.01	2.26E-05
GNE	rs1354034	3	56815721	C	T	0.60	0.13	0.01	2.18E-28
GNE	rs6993770	8	105569300	T	A	0.29	-0.06	0.01	8.89E-06
GNE	rs7090758	10	63575555	C	T	0.48	-0.05	0.01	6.05E-06
GNE	rs11502185	11	180258	C	T	0.26	0.07	0.01	2.93E-06
GNLY	rs144015106	1	24748081	G	A	0.01	-0.49	0.04	2.50E-14
GNLY	rs628745	1	56941254	C	T	0.33	0.05	0.01	5.05E-07
GNLY	rs7545591	1	185426714	G	C	0.32	0.05	0.01	9.51E-07
GNLY	rs7577293	2	85708159	T	C	0.23	0.70	0.01	0.00E+00
GNLY	rs73069104	3	47239243	C	T	0.36	0.07	0.01	4.99E-11
GNLY	rs11711157	3	194341097	T	C	0.28	-0.06	0.01	9.09E-07
GNLY	rs4869313	5	96888176	G	T	0.55	-0.07	0.01	1.48E-11
GNLY	rs9368661	6	31188264	T	C	0.26	0.12	0.01	6.02E-27

GNLY	-	9	136944238	C	T	0.52	0.07	0.01	7.10E-12
GNLY	rs8177651	10	5973427	A	T	0.32	0.05	0.01	1.84E-06
GNLY	rs745932882	10	92682235	A	G	0.46	0.05	0.01	1.24E-07
GNLY	rs4919420	10	100046495	G	A	0.42	-0.07	0.01	1.54E-10
GNLY	rs7137828	12	111494996	T	C	0.52	-0.05	0.01	5.97E-06
GNLY	rs9916257	17	35470352	T	G	0.44	-0.05	0.01	1.62E-07
GOLM2	-	1	199025567	T	A	0.72	0.06	0.01	2.70E-06
GOLM2	rs35316636	2	85312167	A	C	0.51	0.05	0.01	6.55E-06
GOLM2	rs10804823	3	168092778	T	A	0.49	0.05	0.01	7.38E-06
GOLM2	rs13107325	4	102267552	T	C	0.08	-0.10	0.01	1.32E-06
GOLM2	rs1265905	6	31942164	G	A	0.13	-0.11	0.01	2.83E-11
GOLM2	rs41266341	6	158585341	C	A	0.01	0.40	0.04	1.73E-11
GOLM2	rs550057	9	133271182	T	C	0.26	0.26	0.01	5.12E-92
GOLM2	rs171052	11	117211047	G	C	0.23	0.21	0.01	6.71E-58
GOLM2	rs10777172	12	89500490	C	T	0.71	0.20	0.01	6.73E-58
GOLM2	rs550761239	15	44378276	T	C	0.00	0.56	0.06	1.61E-08
GOLM2	rs77542162	17	69085137	G	A	0.02	0.23	0.02	8.90E-10
GOPC	rs1354034	3	56815721	C	T	0.60	0.08	0.01	5.38E-11
GOPC	rs6993770	8	105569300	T	A	0.29	-0.06	0.01	8.77E-06
GOPC	rs11502185	11	180258	C	T	0.26	0.08	0.01	2.93E-07
GP1BA	rs3767809	1	117612209	C	T	0.22	0.07	0.01	2.58E-06
GP1BA	rs1434282	1	199041592	T	C	0.73	0.06	0.01	3.33E-06
GP1BA	rs3804749	3	123114156	T	C	0.59	-0.05	0.01	2.61E-05
GP1BA	rs78565404	3	184372454	T	C	0.05	0.14	0.02	1.30E-07
GP1BA	rs2853677	5	1287079	A	G	0.58	-0.05	0.01	7.92E-06
GP1BA	rs35786788	6	135097904	A	G	0.25	0.10	0.01	1.23E-14
GP1BA	rs6961069	7	80589645	T	C	0.40	0.06	0.01	3.32E-07
GP1BA	rs6993770	8	105569300	T	A	0.29	-0.12	0.01	1.00E-22
GP1BA	rs385893	9	4763176	C	T	0.52	0.11	0.01	6.09E-21
GP1BA	rs12762934	10	102600127	T	C	0.32	0.06	0.01	6.17E-06
GP1BA	rs11217176	11	119194334	A	G	0.27	0.07	0.01	2.60E-07
GP1BA	rs7944186	11	126419473	G	A	0.53	-0.09	0.01	8.96E-14
GP1BA	rs3184504	12	111446804	C	T	0.52	-0.13	0.01	1.16E-32
GP1BA	rs2127870	14	65330128	C	G	0.79	0.07	0.01	6.59E-06
GP1BA	rs1555405	14	100710432	A	G	0.25	-0.07	0.01	1.65E-07
GP1BA	rs7176023	15	64808808	C	T	0.07	0.12	0.01	6.84E-07
GP1BA	rs553749201	17	4933900	A	C	0.05	-0.26	0.02	2.31E-23
GP2	rs6571015	6	96086602	A	G	0.79	0.15	0.01	2.25E-32
GP2	rs6459924	7	159115383	C	T	0.24	-0.06	0.01	1.28E-07
GP2	rs505922	9	133273813	C	T	0.32	0.55	0.01	0.00E+00
GP2	rs2163150	10	48127889	C	T	0.66	-0.05	0.01	1.80E-05
GP2	rs10858917	12	89695013	A	G	0.80	0.06	0.01	3.31E-06
GP2	rs1129818	16	20317363	C	T	0.46	-0.23	0.01	1.02E-112
GP2	rs200489612	17	7203059	A	G	0.01	0.37	0.05	2.35E-06
GP2	rs708686	19	5840608	T	C	0.27	0.06	0.01	4.89E-08
GP2	rs492602	19	48703160	G	A	0.51	0.17	0.01	7.12E-60
GP6	rs1354034	3	56815721	C	T	0.60	0.06	0.01	1.87E-07
GP6	rs17622656	5	132485305	A	G	0.39	-0.05	0.01	3.85E-06
GP6	rs6993770	8	105569300	T	A	0.29	-0.08	0.01	1.08E-09
GP6	rs3847326	10	63572099	G	A	0.48	-0.06	0.01	4.14E-07
GP6	rs10774624	12	111395984	A	G	0.51	-0.05	0.01	2.03E-05
GP6	rs892090	19	55027704	G	T	0.83	0.41	0.01	5.72E-156
GPA33	rs72689400	1	167068982	A	C	0.02	-0.67	0.03	2.63E-55
GPA33	rs2241764	2	132417191	T	C	0.34	-0.09	0.01	1.76E-15
GPA33	rs6437071	2	232704083	A	G	0.42	-0.08	0.01	1.96E-11
GPA33	rs13065564	3	70915893	G	T	0.31	-0.05	0.01	2.59E-05

GPA33	rs9816553	3	154239261	A	G	0.85	-0.09	0.01	1.47E-08
GPA33	rs939885	3	196228891	A	G	0.48	0.07	0.01	6.99E-10
GPA33	rs533067298	4	133569430	C	T	0.51	-0.05	0.01	8.01E-06
GPA33	rs9478784	6	150699194	T	C	0.10	0.12	0.01	7.66E-11
GPA33	rs11142461	9	68444973	C	T	0.04	-0.16	0.02	3.36E-07
GPA33	-	9	133263362	G	A	0.18	-0.09	0.01	1.38E-10
GPA33	rs2291428	10	45463408	C	G	0.24	-0.08	0.01	6.27E-11
GPA33	rs56398830	13	103049340	A	G	0.01	-0.89	0.03	8.14E-66
GPA33	rs35193388	16	4370816	C	G	0.74	-0.07	0.01	4.05E-09
GPA33	rs12938714	17	30318092	A	G	0.33	0.17	0.01	1.33E-46
GPA33	-	17	39383590	C	T	0.26	0.07	0.01	4.47E-07
GPA33	rs681343	19	48703205	T	C	0.51	-0.21	0.01	9.78E-80
GPA33	rs3746778	20	62710120	A	G	0.42	0.07	0.01	7.55E-10
GPC1	rs1856027	1	93078574	C	T	0.62	0.08	0.01	2.33E-12
GPC1	rs150816167	1	179602727	C	T	0.04	0.12	0.02	7.90E-06
GPC1	rs2273967	1	230279547	T	C	0.25	0.07	0.01	6.12E-09
GPC1	rs71414736	2	127104369	G	C	0.02	-0.20	0.02	4.06E-07
GPC1	rs143362987	2	170111430	A	G	0.01	-0.27	0.03	5.17E-07
GPC1	rs77078946	2	240464000	G	T	0.11	0.50	0.01	5.06E-190
GPC1	rs568092436	4	102931225	A	C	0.43	-0.05	0.01	1.91E-05
GPC1	rs3796500	4	128285828	C	T	0.20	-0.06	0.01	2.76E-05
GPC1	rs3965330	5	177622441	A	G	0.47	0.19	0.01	4.79E-69
GPC1	rs4633028	8	39052342	G	A	0.77	-0.08	0.01	8.55E-11
GPC1	rs2625445	8	60627769	A	G	0.24	-0.07	0.01	7.30E-09
GPC1	rs7835610	8	90696825	T	C	0.50	-0.06	0.01	1.69E-08
GPC1	rs11414746	9	33150411	A	T	0.42	0.08	0.01	3.45E-12
GPC1	-	9	137464104	T	C	0.08	-0.14	0.01	1.01E-11
GPC1	rs56278466	10	17833858	G	T	0.66	0.06	0.01	1.47E-07
GPC1	rs1081975	12	55999553	G	C	0.42	0.05	0.01	3.23E-06
GPC1	rs9318186	13	73532802	G	A	0.56	-0.05	0.01	3.41E-06
GPC1	rs7147722	14	49645994	T	G	0.54	0.10	0.01	2.21E-22
GPC1	rs1801689	17	66214462	C	A	0.03	0.19	0.02	8.03E-10
GPC1	rs737939	22	29919427	T	G	0.41	0.06	0.01	1.97E-07
GPC5	rs1434282	1	199041592	T	C	0.73	0.05	0.01	1.47E-05
GPC5	rs1260326	2	27508073	C	T	0.61	-0.06	0.01	1.12E-09
GPC5	rs1354034	3	56815721	C	T	0.60	-0.05	0.01	2.08E-06
GPC5	rs9468900	6	31225221	A	G	0.23	-0.05	0.01	1.03E-05
GPC5	rs6993770	8	105569300	T	A	0.29	-0.06	0.01	5.42E-09
GPC5	rs28601761	8	125487789	G	C	0.42	-0.05	0.01	3.37E-07
GPC5	rs7979478	12	120982460	G	A	0.62	0.05	0.01	1.69E-07
GPC5	rs342706	13	91764804	C	T	0.27	-0.88	0.01	0.00E+00
GPC5	rs28734133	16	81840430	G	A	0.18	0.05	0.01	2.59E-05
GPC5	rs1654425	19	55027612	C	T	0.83	0.09	0.01	3.19E-13
GPNMB	rs4665972	2	27375230	C	T	0.61	0.05	0.01	2.38E-05
GPNMB	rs11721064	3	98689982	T	G	0.61	-0.06	0.01	7.26E-08
GPNMB	rs12203592	6	396321	T	C	0.22	-0.08	0.01	5.37E-08
GPNMB	rs75801644	7	23266522	A	G	0.02	-1.11	0.03	3.05E-185
GPNMB	rs74612335	11	126368738	C	T	0.13	-0.09	0.01	3.99E-08
GPNMB	rs186021206	17	7166093	A	G	0.01	0.74	0.05	8.75E-23
GPR37	rs780094	2	27518370	C	T	0.62	-0.08	0.01	5.67E-16
GPR37	rs511154	3	136232079	G	A	0.77	0.07	0.01	3.01E-10
GPR37	rs13235543	7	73599571	T	C	0.13	-0.11	0.01	1.93E-10
GPR37	rs4141005	7	124488565	C	A	0.26	-0.78	0.01	0.00E+00
GPR37	rs112875651	8	125494452	A	G	0.39	-0.09	0.01	6.65E-19
GPR37	rs11023881	11	16225154	A	T	0.39	0.06	0.01	2.01E-09
GPR37	rs964184	11	116778201	C	G	0.87	-0.06	0.01	1.81E-05

GPR37	rs10083137	12	24045244	G	A	0.03	0.22	0.02	1.95E-16
GPR37	rs1292066	17	59854953	A	T	0.21	0.06	0.01	2.46E-07
GPR37	rs390082	19	44913574	G	T	0.11	0.09	0.01	3.86E-08
GRAP2	rs1354034	3	56815721	C	T	0.60	0.05	0.01	9.21E-06
GRAP2	rs6993770	8	105569300	T	A	0.29	-0.06	0.01	1.61E-06
GRAP2	rs148328786	22	39946989	G	A	0.01	0.31	0.03	2.31E-09
GRK5	rs10886430	10	119250744	G	A	0.13	-0.40	0.01	7.45E-116
GRN	rs12740374	1	109274968	T	G	0.22	-0.67	0.01	0.00E+00
GRN	rs75029704	6	29881729	T	C	0.10	0.16	0.01	1.47E-19
GRN	rs2021716	6	90231570	T	C	0.35	-0.05	0.01	2.40E-05
GRN	rs12666320	7	130375535	G	C	0.12	0.07	0.01	7.64E-06
GRN	rs1137827	9	133254328	T	C	0.06	0.11	0.01	3.01E-07
GRN	rs885828	10	71818746	A	G	0.29	-0.24	0.01	1.43E-96
GRN	rs2298475	11	126408308	C	T	0.08	-0.10	0.01	2.50E-07
GRN	rs111524607	12	120665220	T	C	0.29	-0.05	0.01	1.79E-05
GRN	rs72802342	16	75200974	A	C	0.08	-0.20	0.01	1.83E-23
GRN	rs149044215	16	89638692	A	G	0.37	0.05	0.01	1.88E-05
GRN	rs186021206	17	7166093	A	G	0.01	0.70	0.05	5.95E-23
GRN	rs5848	17	44352876	T	C	0.27	-0.25	0.01	9.43E-105
GRN	rs1065853	19	44909976	T	G	0.08	0.10	0.01	2.68E-07
GRPEL1	rs4361977	1	155103603	T	C	0.43	-0.05	0.01	3.67E-06
GRPEL1	rs4835265	4	145900258	A	C	0.16	0.11	0.01	7.40E-12
GRPEL1	rs4940391	18	58438636	G	A	0.82	-0.09	0.01	1.56E-08
GSAP	rs73333648	10	98404359	G	C	0.05	-0.13	0.02	4.08E-07
GSTA1	rs28383314	6	32619436	C	T	0.63	0.06	0.01	1.38E-06
GSTA1	rs6458871	6	52801179	T	C	0.57	0.31	0.01	3.55E-173
GSTA1	rs112875651	8	125494452	A	G	0.39	-0.06	0.01	9.87E-07
GSTA1	rs10733608	9	114386150	T	G	0.49	-0.07	0.01	2.58E-09
GSTA1	rs10883451	10	100164661	C	T	0.50	-0.05	0.01	1.52E-06
GSTA1	rs4753124	11	94090069	G	C	0.11	-0.08	0.01	1.41E-05
GSTA1	rs28929474	14	94378610	T	C	0.02	0.21	0.02	4.40E-08
GSTA1	rs10405357	19	54255803	C	T	0.44	-0.06	0.01	1.22E-07
GSTA1	rs3747207	22	43928975	A	G	0.22	0.11	0.01	1.54E-15
GSTA3	rs6458871	6	52801179	T	C	0.57	0.31	0.01	2.84E-161
GSTA3	rs28601761	8	125487789	G	C	0.42	-0.05	0.01	2.62E-05
GSTA3	rs11370710	9	114396677	A	C	0.49	-0.05	0.01	2.09E-05
GSTA3	rs10883451	10	100164661	C	T	0.50	-0.05	0.01	2.04E-05
GSTA3	rs28929474	14	94378610	T	C	0.02	0.21	0.03	1.42E-07
GSTA3	rs738409	22	43928847	G	C	0.22	0.09	0.01	5.14E-12
GSTP1	rs762803	11	67584785	A	C	0.45	0.21	0.01	8.14E-69
GUCA2A	rs142157365	1	42162920	T	A	0.00	-2.11	0.08	2.20E-72
GUCA2A	rs3044077	4	68491872	T	T	0.66	-0.08	0.01	1.99E-11
GUCA2A	rs6926755	6	81845964	T	C	0.49	-0.09	0.01	4.58E-15
GUCA2A	rs782819119	9	133270584	A	T	0.31	0.07	0.01	9.02E-08
GUCA2A	rs2511241	11	73234296	T	C	0.93	0.11	0.01	8.70E-07
GUCA2A	rs4982408	14	21102455	C	A	0.84	0.09	0.01	7.50E-09
GUCA2A	rs9928757	16	20341541	C	G	0.20	-0.06	0.01	1.62E-05
GUCA2A	rs1424231	16	79672854	C	T	0.34	0.05	0.01	2.00E-05
GUCA2A	rs17743980	17	9890522	A	G	0.32	0.06	0.01	3.39E-06
GUSB	-	2	61351466	T	A	0.15	0.07	0.01	8.63E-06
GUSB	rs645040	3	136207780	T	G	0.77	0.10	0.01	2.73E-15
GUSB	rs115850161	4	118811292	C	T	0.06	0.18	0.01	5.61E-15
GUSB	rs72814178	5	173059736	G	A	0.13	0.07	0.01	1.40E-05
GUSB	rs111331197	6	7883235	T	C	0.01	-0.24	0.03	4.87E-06
GUSB	-	6	130065067	G	A	0.68	0.05	0.01	1.85E-05
GUSB	rs28538349	7	4032120	A	G	0.52	-0.05	0.01	4.01E-06

GUSB	rs149606212	7	65979854	T	C	0.00	-1.38	0.07	1.11E-33
GUSB	rs11003134	10	52778399	A	C	0.14	0.07	0.01	2.44E-06
GUSB	rs99780	11	61829161	T	C	0.35	0.08	0.01	2.29E-13
GUSB	rs10778152	12	101831973	G	A	0.29	-0.26	0.01	2.25E-105
GUSB	rs150641790	13	41113708	G	T	0.07	0.09	0.01	6.78E-06
GUSB	rs147233090	15	43735849	T	C	0.02	0.28	0.02	1.16E-15
GUSB	rs1801690	17	66212167	G	C	0.06	-0.28	0.01	1.72E-34
GUSB	rs5826313	18	74290916	A	C	0.32	0.06	0.01	2.74E-08
GUSB	rs2974755	19	12951849	T	C	0.60	0.05	0.01	9.62E-06
GUSB	rs200210321	19	19283081	G	A	0.07	0.19	0.01	4.15E-21
GYS1	rs342299	7	106733272	T	C	0.46	-0.05	0.01	2.06E-05
GYS1	rs3748136	8	9172650	A	G	0.21	-0.31	0.01	1.02E-108
GYS1	rs11553699	12	121779004	G	A	0.14	-0.08	0.01	1.34E-05
GYS1	rs112805222	16	47188773	A	G	0.05	0.13	0.02	4.51E-06
GZMA	rs76036957	2	112738876	A	G	0.01	0.29	0.04	9.61E-07
GZMA	rs13063578	3	47046347	A	T	0.40	-0.07	0.01	4.62E-09
GZMA	rs230507	4	102559089	A	C	0.65	0.06	0.01	2.32E-06
GZMA	rs2047745	5	55193713	C	T	0.21	0.07	0.01	4.55E-07
GZMA	-	5	100810354	A	C	0.62	0.05	0.01	2.12E-05
GZMA	rs3846730	5	132471088	T	C	0.24	-0.06	0.01	5.59E-06
GZMA	rs9264645	6	31270747	A	G	0.64	0.27	0.01	2.14E-116
GZMA	rs2347784	7	6485212	G	C	0.27	-0.07	0.01	7.80E-09
GZMA	rs6476398	9	33119243	T	C	0.31	-0.09	0.01	6.84E-13
GZMA	rs41280225	9	113261699	C	A	0.13	-0.09	0.01	1.07E-07
GZMA	rs8177652	10	5973421	A	G	0.32	0.06	0.01	5.28E-06
GZMA	rs113430316	11	128246346	A	T	0.28	-0.06	0.01	6.29E-06
GZMA	rs75587749	12	7462344	T	C	0.09	0.09	0.01	6.11E-06
GZMA	rs4764809	12	101702234	T	A	0.49	0.06	0.01	9.62E-08
GZMA	rs3184504	12	111446804	C	T	0.52	-0.12	0.01	6.42E-26
GZMA	rs145078947	14	93186629	T	G	0.00	0.65	0.07	1.29E-09
GZMA	rs10402422	19	16364577	A	G	0.69	-0.06	0.01	3.55E-06
GZMA	rs12986064	19	54251270	C	T	0.51	-0.08	0.01	3.35E-11
GZMB	rs2643902	1	2314000	C	T	0.79	-0.07	0.01	1.24E-06
GZMB	rs760805	1	24925432	T	A	0.57	0.06	0.01	1.48E-07
GZMB	rs2725124	5	100810073	A	C	0.62	0.07	0.01	9.03E-10
GZMB	rs41563615	6	31356598	T	G	0.05	0.42	0.02	3.40E-68
GZMB	rs2497318	10	92672243	T	C	0.45	0.06	0.01	3.94E-08
GZMB	rs756658243	11	128189307	T	A	0.29	-0.09	0.01	6.41E-13
GZMB	rs2460740	12	10428847	G	T	0.68	-0.08	0.01	2.37E-12
GZMB	rs7137828	12	111494996	T	C	0.52	-0.06	0.01	2.69E-07
GZMB	rs8192918	14	24632672	T	C	0.22	-0.36	0.01	2.12E-165
GZMB	rs12451746	17	35479844	A	G	0.43	-0.13	0.01	1.18E-34
GZMB	rs56133626	19	16331162	A	G	0.33	-0.06	0.01	7.59E-08
GZMB	rs35477491	21	13901496	T	C	0.22	-0.07	0.01	1.99E-05
GZMH	rs10798014	1	185425702	G	A	0.32	0.08	0.01	1.96E-12
GZMH	rs3732378	3	39265671	A	G	0.17	0.06	0.01	1.82E-05
GZMH	rs2844622	6	31272283	G	A	0.66	0.12	0.01	5.10E-26
GZMH	rs2347784	7	6485212	G	C	0.27	-0.07	0.01	3.01E-07
GZMH	-	9	33149200	T	A	0.39	-0.05	0.01	3.96E-06
GZMH	-	9	136946019	T	G	0.52	0.05	0.01	6.95E-06
GZMH	rs78325861	10	70618733	G	C	0.04	0.16	0.02	1.15E-08
GZMH	rs576785	11	128183379	C	T	0.29	-0.11	0.01	1.18E-18
GZMH	rs2859660	12	10429695	T	C	0.84	-0.09	0.01	9.65E-09
GZMH	rs190749747	14	24607097	G	C	0.01	-0.89	0.04	7.77E-59
GZMH	rs56133626	19	16331162	A	G	0.33	-0.06	0.01	5.16E-06
HAGH	rs115389912	16	1819973	A	G	0.01	-0.81	0.05	1.04E-26

HAO1	rs1497406	1	16178825	G	A	0.58	0.06	0.01	2.84E-08
HAO1	rs12904	1	155134221	A	G	0.41	-0.06	0.01	4.18E-07
HAO1	rs7608141	2	296381	C	A	0.33	0.06	0.01	1.66E-06
HAO1	rs4835265	4	145900258	A	C	0.16	0.12	0.01	4.95E-15
HAO1	rs112875651	8	125494452	A	G	0.39	-0.05	0.01	1.04E-05
HAO1	rs151068477	10	50882756	T	G	0.01	0.28	0.04	2.04E-05
HAO1	rs10883451	10	100164661	C	T	0.50	-0.05	0.01	2.66E-05
HAO1	rs12373325	18	58420416	C	T	0.80	-0.10	0.01	1.03E-12
HAO1	rs738409	22	43928847	G	C	0.22	0.06	0.01	2.65E-06
HARS1	rs1354034	3	56815721	C	T	0.60	0.07	0.01	5.68E-10
HARS1	rs342296	7	106732457	A	G	0.46	-0.05	0.01	7.77E-06
HARS1	rs60822569	12	54323724	C	T	0.55	0.05	0.01	1.11E-05
HAVCR1	-	1	43259622	C	A	0.43	0.07	0.01	2.19E-12
HAVCR1	rs2070803	1	155185239	G	A	0.43	0.05	0.01	2.55E-07
HAVCR1	rs56139727	5	697265	T	G	0.70	-0.06	0.01	2.19E-06
HAVCR1	rs6878069	5	157044058	G	A	0.48	-0.48	0.01	0.00E+00
HAVCR1	rs3117182	6	32099042	A	T	0.85	0.05	0.01	2.76E-05
HAVCR1	rs112875651	8	125494452	A	G	0.39	-0.06	0.01	1.71E-10
HAVCR1	rs2070895	15	58431740	A	G	0.22	0.08	0.01	5.05E-12
HAVCR1	rs77924615	16	20381010	A	G	0.20	-0.12	0.01	2.55E-24
HAVCR1	rs112001035	17	68827664	A	G	0.06	0.22	0.01	7.19E-30
HAVCR2	rs61747728	1	179557079	T	C	0.04	0.16	0.02	4.08E-08
HAVCR2	rs7569939	2	62278920	G	A	0.52	-0.06	0.01	3.15E-08
HAVCR2	rs7630058	3	98618673	T	C	0.22	-0.08	0.01	2.78E-09
HAVCR2	rs116363290	5	157109126	T	C	0.01	-1.72	0.05	2.58E-108
HAVCR2	rs509227	6	10535358	C	A	0.52	0.05	0.01	1.25E-06
HAVCR2	rs950802	11	60385111	A	G	0.31	0.11	0.01	1.18E-21
HAVCR2	rs77924615	16	20381010	A	G	0.20	-0.07	0.01	4.31E-06
HAVCR2	rs186021206	17	7166093	A	G	0.01	0.48	0.05	2.91E-10
HBEGF	rs3811444	1	247876149	T	C	0.33	-0.06	0.01	2.76E-07
HBEGF	rs78909033	2	240571486	A	G	0.14	-0.08	0.01	4.41E-07
HBEGF	rs2079797	3	47300311	A	G	0.45	0.06	0.01	3.59E-06
HBEGF	rs1354034	3	56815721	C	T	0.60	-0.16	0.01	7.47E-43
HBEGF	rs10016018	4	101873464	T	A	0.40	-0.05	0.01	2.42E-05
HBEGF	rs274555	5	132387259	T	C	0.40	0.05	0.01	8.44E-06
HBEGF	rs4098923	6	31225979	G	A	0.45	-0.06	0.01	6.96E-08
HBEGF	rs62396356	6	41200025	G	A	0.13	0.09	0.01	2.44E-07
HBEGF	rs6961069	7	80589645	T	C	0.40	0.05	0.01	2.81E-05
HBEGF	rs342299	7	106733272	T	C	0.46	-0.05	0.01	4.61E-06
HBEGF	rs447124	9	4746539	T	C	0.49	0.06	0.01	1.25E-06
HBEGF	rs10820606	9	96430637	C	A	0.23	-0.11	0.01	3.91E-17
HBEGF	rs467369	9	134040643	T	C	0.53	0.06	0.01	2.04E-08
HBEGF	rs10761737	10	63292445	C	T	0.41	0.17	0.01	2.46E-52
HBEGF	rs80137017	10	69477626	T	C	0.13	0.08	0.01	3.44E-06
HBEGF	rs71474568	10	102589091	G	T	0.34	0.06	0.01	1.65E-06
HBEGF	rs489298	12	3129999	T	C	0.40	-0.05	0.01	1.40E-05
HBEGF	rs4608137	12	6184207	A	G	0.49	-0.05	0.01	2.19E-05
HBEGF	rs1716505	12	64611299	G	C	0.32	0.05	0.01	1.21E-05
HBEGF	rs11553699	12	121779004	G	A	0.14	0.16	0.01	9.58E-22
HBEGF	rs12445050	16	81837364	T	C	0.14	0.08	0.01	3.93E-06
HBEGF	rs516051	17	29555687	C	A	0.52	-0.07	0.01	1.10E-09
HBEGF	rs58635160	19	8454684	C	T	0.50	-0.06	0.01	2.12E-06
HBEGF	rs1654425	19	55027612	C	T	0.83	0.15	0.01	2.56E-23
HBEGF	rs3790176	20	19281278	A	G	0.34	0.06	0.01	1.36E-06
HBEGF	rs116905185	20	50604766	C	T	0.01	0.30	0.04	5.70E-08
HBQ1	rs11759553	6	135101158	T	A	0.27	-0.05	0.01	2.63E-05

HBQ1	rs3760047	16	231300	A	G	0.81	0.47	0.01	2.71E-242
HBQ1	-	16	72045090	T	G	0.18	0.09	0.01	4.39E-10
HCLS1	rs1354034	3	56815721	C	T	0.60	0.05	0.01	1.76E-05
HCLS1	rs3732410	3	121696873	C	T	0.23	0.09	0.01	3.83E-12
HCLS1	rs4759076	12	54336088	C	T	0.46	0.05	0.01	3.99E-06
HCLS1	rs10418046	19	53824615	G	T	0.21	0.11	0.01	1.28E-15
HDGF	rs3806417	1	156741831	A	C	0.33	1.14	0.01	0.00E+00
HDGF	rs6993770	8	105569300	T	A	0.29	-0.05	0.01	3.92E-10
HEBP1	rs1941	12	12975329	G	C	0.04	-0.32	0.02	9.77E-26
HEXIM1	rs1354034	3	56815721	C	T	0.60	0.13	0.01	3.24E-32
HEXIM1	rs114694170	5	88884379	C	T	0.06	0.12	0.02	1.05E-06
HEXIM1	rs7080386	10	63288546	A	C	0.41	0.06	0.01	6.31E-07
HEXIM1	rs11502185	11	180258	C	T	0.26	0.08	0.01	1.69E-08
HEXIM1	rs60822569	12	54323724	C	T	0.55	0.06	0.01	7.39E-08
HEXIM1	-	17	45145418	G	T	0.48	0.07	0.01	1.07E-08
HEXIM1	rs1671152	19	55014977	G	T	0.84	0.07	0.01	2.50E-05
HGF	rs59950280	4	3450618	A	G	0.33	0.14	0.01	3.56E-31
HGF	rs5745687	7	81729735	T	C	0.07	-0.19	0.01	5.93E-17
HGF	-	8	105561296	C	A	0.27	-0.06	0.01	7.05E-07
HGF	rs2511241	11	73234296	T	C	0.93	0.16	0.01	6.21E-13
HGF	rs7310615	12	111427245	G	C	0.52	-0.06	0.01	3.20E-07
HGF	rs704	17	28367840	A	G	0.47	-0.06	0.01	2.17E-07
HGS	rs3811444	1	247876149	T	C	0.33	-0.06	0.01	2.04E-07
HGS	rs1354034	3	56815721	C	T	0.60	0.06	0.01	8.79E-07
HGS	rs342293	7	106731773	G	C	0.46	-0.05	0.01	5.10E-06
HGS	rs6993770	8	105569300	T	A	0.29	-0.06	0.01	2.56E-05
HGS	-	10	63156270	A	T	0.49	0.05	0.01	1.28E-05
HLA-DRA	rs36096565	6	32592248	G	A	0.21	0.68	0.01	0.00E+00
HLA-DRA	rs76904798	12	40220632	T	C	0.14	0.09	0.01	4.21E-10
HLA-DRA	rs3184504	12	111446804	C	T	0.52	-0.06	0.01	6.01E-10
HLA-E	rs2927608	5	96916728	A	G	0.45	-0.19	0.01	3.76E-69
HLA-E	rs2523594	6	31358852	T	C	0.62	-0.32	0.01	1.65E-174
HLA-E	rs7137828	12	111494996	T	C	0.52	-0.09	0.01	5.32E-19
HLA-E	rs74439742	16	57025515	T	C	0.17	-0.09	0.01	3.16E-11
HMBS	rs72997349	11	119074792	C	T	0.03	0.38	0.02	7.79E-33
HMBS	-	22	21586875	C	T	0.19	-0.07	0.01	1.15E-06
HMOX1	rs371562288	1	62553032	C	T	0.63	0.05	0.01	1.32E-05
HMOX1	rs385076	2	32264782	C	T	0.64	0.17	0.01	1.40E-48
HMOX1	rs6786207	3	69789164	T	G	0.65	-0.08	0.01	9.81E-11
HMOX1	rs17229943	5	69386709	C	A	0.05	0.17	0.02	7.85E-11
HMOX1	rs13167071	5	156946626	C	G	0.64	-0.06	0.01	6.36E-07
HMOX1	rs2032444	6	26046516	G	T	0.35	0.06	0.01	4.58E-07
HMOX1	rs2637678	6	116466215	C	T	0.41	0.07	0.01	2.88E-10
HMOX1	rs11379524	9	93120463	C	A	0.63	0.05	0.01	2.65E-05
HMOX1	rs17476364	10	69334748	C	T	0.11	0.08	0.01	9.30E-06
HMOX1	rs7080536	10	113588287	A	G	0.04	0.35	0.02	1.70E-37
HMOX1	rs10846744	12	124827879	C	G	0.14	0.09	0.01	3.81E-08
HMOX1	rs261290	15	58386521	C	T	0.65	-0.06	0.01	1.47E-07
HMOX1	rs12149545	16	56959249	A	G	0.32	0.07	0.01	5.17E-08
HMOX1	rs34931250	17	68883786	T	C	0.06	0.12	0.02	3.78E-07
HMOX1	rs1065853	19	44909976	T	G	0.08	0.19	0.01	2.06E-20
HMOX1	rs34632751	19	48211939	C	T	0.03	0.32	0.02	8.86E-21
HMOX1	rs117280289	20	26252163	T	C	0.05	-0.13	0.02	2.42E-07
HMOX1	rs113460913	20	31534093	A	T	0.15	-0.19	0.01	1.68E-36
HMOX1	rs2071747	22	35381192	C	G	0.06	-0.15	0.02	1.68E-10
HMOX1	rs738409	22	43928847	G	C	0.22	0.06	0.01	2.28E-05

HMOX2	rs4835265	4	145900258	A	C	0.16	0.10	0.01	3.28E-10
HMOX2	rs3747584	16	4466091	C	T	0.74	-0.12	0.01	5.49E-20
HMOX2	rs4503880	18	58416822	C	T	0.80	-0.09	0.01	3.45E-09
HMOX2	rs429358	19	44908684	C	T	0.16	0.10	0.01	3.15E-10
HNMT	rs11558538	2	138002079	T	C	0.11	-1.11	0.01	0.00E+00
HNMT	rs7032525	9	93120619	C	T	0.20	-0.06	0.01	1.11E-06
HNMT	rs10418046	19	53824615	G	T	0.21	0.06	0.01	2.58E-07
HNMT	rs738409	22	43928847	G	C	0.22	0.07	0.01	3.70E-08
HNRNPK	rs6993770	8	105569300	T	A	0.29	-0.06	0.01	5.60E-07
HPCAL1	rs375285744	2	10336102	C	T	0.15	0.22	0.01	4.08E-42
HPGDS	rs763228358	1	196666644	A	G	0.25	-0.06	0.01	3.11E-06
HPGDS	rs2865353	4	94349727	C	T	0.61	0.46	0.01	0.00E+00
HPGDS	rs2398827	9	93144825	C	T	0.60	0.07	0.01	4.40E-09
HS3ST3B1	rs4970836	1	109279175	A	G	0.77	0.07	0.01	2.12E-08
HS3ST3B1	rs4846914	1	230159944	A	G	0.61	0.14	0.01	4.13E-37
HS3ST3B1	rs11096641	2	20174397	A	G	0.47	-0.05	0.01	8.86E-07
HS3ST3B1	rs5030062	3	186736391	C	A	0.37	-0.06	0.01	2.62E-07
HS3ST3B1	rs13107325	4	102267552	T	C	0.08	-0.11	0.01	1.33E-07
HS3ST3B1	rs3836616	4	186233118	A	T	0.51	-0.09	0.01	6.82E-17
HS3ST3B1	rs1801020	5	177409531	G	A	0.75	-0.06	0.01	4.43E-07
HS3ST3B1	rs28929474	14	94378610	T	C	0.02	0.23	0.02	2.39E-09
HS3ST3B1	rs9302635	16	72110275	C	T	0.18	0.07	0.01	5.08E-07
HS3ST3B1	rs17669311	17	13933734	A	G	0.39	0.33	0.01	1.78E-174
HS6ST1	rs12740374	1	109274968	T	G	0.22	-0.07	0.01	3.21E-08
HS6ST1	rs907866	2	20171619	A	G	0.45	-0.07	0.01	1.32E-10
HS6ST1	rs6761320	2	128292780	T	C	0.43	-0.21	0.01	3.42E-76
HS6ST1	rs17282078	3	124762913	A	T	0.13	-0.10	0.01	7.69E-09
HS6ST1	rs13107325	4	102267552	T	C	0.08	0.17	0.01	4.44E-15
HS6ST1	rs1290147	5	150540177	C	G	0.65	-0.05	0.01	2.37E-05
HS6ST1	rs573098	6	31874113	T	A	0.67	0.07	0.01	4.80E-08
HS6ST1	rs71556736	7	73620599	T	C	0.13	-0.08	0.01	4.64E-06
HS6ST1	rs6952090	7	130282658	C	T	0.35	-0.06	0.01	3.92E-07
HS6ST1	rs56278466	10	17833858	G	T	0.66	0.07	0.01	2.61E-08
HS6ST1	rs1149596	11	76758049	T	C	0.17	-0.08	0.01	2.51E-07
HS6ST1	rs770578737	11	117146838	G	A	0.79	-0.06	0.01	1.07E-05
HS6ST1	rs35350651	12	111469627	C	A	0.50	-0.05	0.01	3.83E-06
HS6ST1	rs12903915	15	50736551	C	T	0.34	-0.06	0.01	1.94E-07
HS6ST1	rs72802342	16	75200974	A	C	0.08	-0.19	0.01	1.62E-19
HS6ST1	rs704	17	28367840	A	G	0.47	-0.15	0.01	1.50E-42
HS6ST1	rs77542162	17	69085137	G	A	0.02	0.29	0.02	2.94E-15
HSD11B1	rs78444298	1	184702964	A	G	0.02	0.25	0.03	2.77E-09
HSD11B1	rs7542235	1	196854483	G	A	0.19	0.16	0.01	5.37E-28
HSD11B1	rs4240624	8	9326721	A	G	0.91	0.17	0.01	1.67E-18
HSD11B1	rs174547	11	61803311	C	T	0.34	0.07	0.01	9.21E-10
HSD11B1	rs695110	11	75745536	C	T	0.15	0.08	0.01	1.12E-06
HSD11B1	rs1076485	11	116901725	T	C	0.12	0.11	0.01	4.37E-10
HSD11B1	rs28929474	14	94378610	T	C	0.02	0.65	0.03	3.56E-60
HSD11B1	rs56156922	16	56953457	C	T	0.33	0.05	0.01	2.68E-05
HSD11B1	rs2532351	17	46259631	G	C	0.42	0.05	0.01	6.30E-06
HSD11B1	rs1801689	17	66214462	C	A	0.03	0.44	0.02	6.68E-40
HSD11B1	rs58542926	19	19268740	T	C	0.08	0.22	0.01	2.18E-25
HSD11B1	rs141887102	19	58565664	C	A	0.18	0.09	0.01	5.48E-09
HSPA1A	rs181412562	6	31849800	T	C	0.00	-1.28	0.09	3.49E-21
HSPA1A	rs342293	7	106731773	G	C	0.46	-0.06	0.01	1.73E-06
HSPA1A	rs11218968	11	123087770	G	A	0.04	-0.14	0.02	7.00E-07
HSPB1	rs1354034	3	56815721	C	T	0.60	0.05	0.01	2.57E-06

HSPB1	rs6926219	6	122399661	A	G	0.55	-0.09	0.01	1.66E-16
HSPB1	rs111642997	7	76290678	A	C	0.24	-0.55	0.01	0.00E+00
HSPB1	rs342298	7	106733200	T	C	0.46	-0.08	0.01	9.99E-11
HSPB1	rs7896518	10	63344740	G	A	0.42	0.08	0.01	1.76E-11
HSPB1	rs11553699	12	121779004	G	A	0.14	-0.08	0.01	2.68E-06
HSPB6	rs12463674	2	178567458	G	A	0.32	0.05	0.01	1.35E-06
HSPB6	rs12331051	4	186218653	T	C	0.43	0.06	0.01	5.62E-08
HSPB6	rs151828	5	96724558	C	T	0.55	0.05	0.01	9.12E-07
HSPB6	-	6	28200548	A	C	0.23	0.06	0.01	3.17E-07
HSPB6	rs2234962	10	119670121	C	T	0.22	0.06	0.01	5.39E-06
HSPB6	-	11	22178894	C	G	0.53	0.06	0.01	4.41E-10
HSPB6	rs1337298	13	73556538	T	C	0.44	-0.05	0.01	1.08E-06
HSPG2	rs6684152	1	21821271	G	A	0.29	-0.17	0.01	5.03E-45
HSPG2	rs1398018	4	76451770	C	T	0.44	0.05	0.01	9.16E-06
HSPG2	rs10480747	7	121240519	C	A	0.35	-0.05	0.01	2.49E-05
HSPG2	rs77924615	16	20381010	A	G	0.20	-0.08	0.01	1.23E-08
HTRA2	rs6993770	8	105569300	T	A	0.29	-0.06	0.01	9.95E-06
HYAL1	rs11801243	1	180158216	A	G	0.06	-0.10	0.02	1.72E-05
HYAL1	rs116482870	3	50302191	T	C	0.06	-0.54	0.02	1.62E-119
HYAL1	rs12321232	12	9163072	T	G	0.23	-0.06	0.01	6.39E-06
HYAL1	rs142650905	12	57208108	T	C	0.00	-1.53	0.10	2.04E-25
HYOU1	rs7532110	1	47496292	A	T	0.80	0.09	0.01	1.37E-09
HYOU1	rs61747728	1	179557079	T	C	0.04	0.23	0.02	1.06E-14
HYOU1	rs59484402	1	230167092	T	C	0.61	-0.05	0.01	2.75E-05
HYOU1	rs75166367	2	162107791	A	G	0.06	-0.13	0.02	2.50E-08
HYOU1	rs149547076	3	57571717	C	T	0.01	-0.33	0.04	2.33E-09
HYOU1	rs74676624	7	127459985	C	T	0.07	-0.10	0.01	6.97E-06
HYOU1	rs2509121	11	119057542	T	C	0.40	0.18	0.01	9.39E-58
HYOU1	rs28929474	14	94378610	T	C	0.02	0.30	0.03	4.16E-14
HYOU1	rs4965806	15	101212230	A	G	0.39	0.05	0.01	2.95E-06
HYOU1	rs777834943	16	20355409	T	C	0.18	-0.06	0.01	1.72E-05
HYOU1	rs1801689	17	66214462	C	A	0.03	0.29	0.02	2.65E-18
HYOU1	rs2659005	17	81244914	T	C	0.44	0.08	0.01	1.64E-13
HYOU1	rs33950747	19	35848345	T	C	0.08	0.11	0.01	1.02E-07
HYOU1	rs10419198	19	49534760	T	C	0.25	0.06	0.01	6.17E-06
HYOU1	rs136205	22	36358158	G	C	0.75	-0.06	0.01	9.10E-06
ICA1	rs1354034	3	56815721	C	T	0.60	0.05	0.01	1.20E-05
ICA1	rs60822569	12	54323724	C	T	0.55	0.05	0.01	2.46E-05
ICA1	rs3952	22	38060591	G	A	0.31	-0.22	0.01	5.68E-72
ICAM1	-	9	133263362	G	A	0.18	-0.27	0.01	3.82E-90
ICAM1	rs1049728	11	65653646	C	G	0.06	-0.17	0.01	1.74E-14
ICAM1	rs34434834	11	126437901	A	G	0.03	0.15	0.02	1.04E-06
ICAM1	rs7137828	12	111494996	T	C	0.52	-0.08	0.01	6.07E-15
ICAM1	rs186021206	17	7166093	A	G	0.01	0.63	0.05	1.98E-18
ICAM1	rs5030377	19	10282558	G	A	0.38	0.41	0.01	6.83E-283
ICAM1	rs3136642	19	38907776	G	A	0.37	-0.06	0.01	1.35E-06
ICAM2	-	1	43812246	G	A	0.29	0.06	0.01	6.70E-09
ICAM2	rs11682865	2	62390661	C	G	0.19	-0.05	0.01	1.92E-05
ICAM2	rs10935473	3	98698056	T	G	0.44	-0.71	0.01	0.00E+00
ICAM2	rs9399137	6	135097880	C	T	0.26	0.06	0.01	1.01E-08
ICAM2	rs6993770	8	105569300	T	A	0.29	-0.06	0.01	2.16E-10
ICAM2	rs2519093	9	133266456	T	C	0.18	-0.85	0.01	0.00E+00
ICAM2	rs78689694	11	126364925	C	G	0.13	-0.08	0.01	3.15E-11
ICAM2	rs111626763	12	26681871	C	T	0.59	0.04	0.01	3.82E-06
ICAM2	rs111338191	12	111388673	T	A	0.52	-0.08	0.01	2.74E-20
ICAM2	rs186021206	17	7166093	A	G	0.01	1.25	0.04	1.68E-102

ICAM2	rs574698129	17	64022514	C	A	0.19	0.10	0.01	1.42E-18
ICAM2	rs368565	19	48697960	T	C	0.43	-0.04	0.01	1.42E-06
ICAM2	rs2837989	21	41248192	A	C	0.19	0.05	0.01	8.79E-07
ICAM3	rs188468174	1	24965206	T	C	0.01	-0.35	0.03	3.04E-14
ICAM3	rs778683816	1	43812878	C	T	0.35	0.05	0.01	8.06E-06
ICAM3	rs2289235	2	230873453	A	G	0.47	-0.15	0.01	2.11E-43
ICAM3	rs6764279	3	187006187	A	C	0.28	-0.09	0.01	7.14E-13
ICAM3	rs13135092	4	102276925	G	A	0.08	0.09	0.01	2.17E-05
ICAM3	rs1049709	6	31269077	C	T	0.17	-0.19	0.01	3.17E-40
ICAM3	rs195075	6	119332122	C	T	0.26	0.06	0.01	5.83E-06
ICAM3	rs7758383	6	142848586	G	A	0.49	0.08	0.01	1.37E-13
ICAM3	rs2278467	8	24331331	C	T	0.16	-0.21	0.01	2.60E-46
ICAM3	rs12574844	11	126403491	A	G	0.07	-0.15	0.01	3.17E-13
ICAM3	rs3184504	12	111446804	C	T	0.52	-0.14	0.01	6.28E-38
ICAM3	rs494632	12	120751313	T	C	0.47	-0.06	0.01	5.57E-08
ICAM3	rs76428106	13	28029870	C	T	0.01	0.30	0.03	3.70E-09
ICAM3	rs9316367	13	48236653	T	C	0.25	-0.11	0.01	1.06E-19
ICAM3	rs186021206	17	7166093	A	G	0.01	1.18	0.05	6.42E-58
ICAM3	rs34562254	17	16939677	A	G	0.10	0.09	0.01	4.80E-06
ICAM3	rs2305482	17	39984674	C	A	0.54	-0.06	0.01	3.55E-09
ICAM3	-	17	46185215	C	A	0.17	-0.07	0.01	1.20E-05
ICAM3	rs56378716	17	58279141	G	A	0.01	0.30	0.03	2.34E-10
ICAM3	rs2725405	17	81246424	C	G	0.44	0.10	0.01	3.29E-19
ICAM3	rs28378712	19	10346796	G	T	0.31	0.32	0.01	5.35E-158
ICAM3	rs602662	19	48703728	A	G	0.54	0.05	0.01	3.38E-06
ICAM3	rs6517655	21	41164319	C	G	0.27	0.07	0.01	1.13E-07
ICAM4	rs139898146	1	25274256	A	C	0.42	-0.68	0.01	0.00E+00
ICAM4	rs11576522	1	207615924	G	A	0.66	0.08	0.01	1.46E-10
ICAM4	rs7606173	2	60498316	C	G	0.43	-0.06	0.01	4.36E-10
ICAM4	rs11731918	4	144133421	T	A	0.45	0.22	0.01	2.20E-108
ICAM4	rs2523990	6	30109452	G	A	0.54	-0.05	0.01	8.37E-08
ICAM4	rs62427982	6	107115962	T	C	0.33	0.06	0.01	1.79E-07
ICAM4	rs9376091	6	135098498	T	C	0.26	-0.09	0.01	6.58E-17
ICAM4	rs6592965	7	50360284	A	G	0.45	-0.05	0.01	3.50E-08
ICAM4	-	9	133263362	G	A	0.18	-0.32	0.01	3.44E-145
ICAM4	rs35141569	10	79040331	T	G	0.24	0.05	0.01	1.16E-05
ICAM4	rs11627485	14	65020976	C	T	0.45	0.05	0.01	1.37E-06
ICAM4	rs17580	14	94380925	A	T	0.05	0.16	0.01	7.62E-13
ICAM4	rs186021206	17	7166093	A	G	0.01	0.53	0.04	2.96E-15
ICAM4	-	17	46105617	G	A	0.17	0.06	0.01	1.19E-05
ICAM4	rs73425119	18	46173865	C	G	0.26	0.07	0.01	2.20E-11
ICAM4	rs5030377	19	10282558	G	A	0.38	-0.11	0.01	2.03E-29
ICAM4	rs746064	21	41209521	A	G	0.38	0.09	0.01	2.76E-21
ICAM5	rs3864106	3	186938813	C	G	0.58	-0.05	0.01	1.51E-06
ICAM5	-	4	55570661	T	A	0.36	0.05	0.01	3.47E-08
ICAM5	-	6	29887544	G	C	0.01	0.22	0.03	8.60E-06
ICAM5	rs2049865	8	115576319	A	C	0.58	0.05	0.01	5.78E-07
ICAM5	rs2519093	9	133266456	T	C	0.18	-0.41	0.01	7.37E-253
ICAM5	rs75384243	11	126369257	T	G	0.09	0.09	0.01	5.22E-09
ICAM5	rs186021206	17	7166093	A	G	0.01	0.59	0.04	1.24E-20
ICAM5	rs77542162	17	69085137	G	A	0.02	0.52	0.02	3.25E-64
ICAM5	rs281439	19	10289434	C	G	0.78	0.84	0.01	0.00E+00
ICOSLG	rs74152937	10	100045864	A	G	0.05	0.12	0.02	3.77E-06
ICOSLG	rs7103461	11	126369390	G	C	0.13	-0.07	0.01	2.36E-05
ICOSLG	rs186021206	17	7166093	A	G	0.01	0.77	0.05	1.64E-24
ICOSLG	rs6518352	21	44226815	G	A	0.72	-0.40	0.01	3.99E-215

IDI2	rs1044261	10	1019770	T	C	0.08	-0.60	0.01	5.30E-182
IDI2	-	13	73526441	G	A	0.50	0.05	0.01	6.64E-06
IDS	rs529565	9	133274084	C	T	0.32	-0.06	0.01	5.44E-06
IDS	rs10128858	12	101827588	G	A	0.29	-0.21	0.01	8.83E-63
IDS	rs704	17	28367840	A	G	0.47	-0.09	0.01	4.33E-15
IDUA	rs6682862	1	177969302	A	G	0.17	0.06	0.01	6.17E-06
IDUA	rs687339	3	136213517	T	C	0.77	0.06	0.01	2.09E-07
IDUA	rs3796622	4	989272	C	T	0.64	0.54	0.01	0.00E+00
IDUA	rs114816312	4	109717668	T	C	0.01	0.30	0.04	5.45E-06
IDUA	rs2389688	4	118815641	C	A	0.44	-0.05	0.01	7.55E-06
IDUA	rs11791806	9	34633532	T	C	0.17	0.06	0.01	1.08E-05
IDUA	rs10778152	12	101831973	G	A	0.29	-0.24	0.01	3.30E-105
IDUA	rs2254971	12	121012581	C	G	0.37	0.05	0.01	1.84E-06
IDUA	rs71435622	14	39242324	T	A	0.29	0.06	0.01	2.00E-06
IDUA	rs145078947	14	93186629	T	G	0.00	0.73	0.06	2.47E-14
IDUA	rs139974673	15	43735687	C	T	0.02	0.20	0.02	1.82E-10
IDUA	rs58542926	19	19268740	T	C	0.08	0.12	0.01	3.67E-10
IDUA	rs429358	19	44908684	C	T	0.16	-0.08	0.01	1.01E-08
IDUA	rs1883711	20	40551182	C	G	0.03	0.14	0.02	5.12E-06
IFNG	rs76830965	3	159919889	A	C	0.12	0.08	0.01	1.11E-05
IFNG	rs2844622	6	31272283	G	A	0.66	0.06	0.01	3.99E-06
IFNGR1	rs61747728	1	179557079	T	C	0.04	0.19	0.02	1.28E-10
IFNGR1	rs1260326	2	27508073	C	T	0.61	0.05	0.01	4.15E-06
IFNGR1	rs73065135	3	46847019	C	G	0.10	0.08	0.01	1.07E-05
IFNGR1	rs9853056	3	52521941	C	T	0.56	0.05	0.01	7.43E-06
IFNGR1	rs2394177	6	29819592	G	A	0.08	0.12	0.01	1.08E-08
IFNGR1	rs11754268	6	137219198	T	C	0.21	-0.21	0.01	1.00E-55
IFNGR1	rs8176672	9	133266772	T	C	0.06	0.36	0.02	7.18E-56
IFNGR1	rs77924615	16	20381010	A	G	0.20	-0.08	0.01	9.72E-09
IFNGR1	rs186021206	17	7166093	A	G	0.01	0.84	0.05	1.12E-28
IFNGR1	rs2659005	17	81244914	T	C	0.44	0.05	0.01	2.84E-06
IFNGR2	rs12740374	1	109274968	T	G	0.22	-0.07	0.01	4.43E-13
IFNGR2	rs61804164	1	161653235	C	G	0.12	0.06	0.01	1.23E-05
IFNGR2	-	7	35695033	C	G	0.42	-0.07	0.01	2.55E-18
IFNGR2	rs10901252	9	133252613	C	G	0.06	0.13	0.01	1.93E-13
IFNGR2	rs56278466	10	17833858	G	T	0.66	0.08	0.01	2.11E-18
IFNGR2	rs174564	11	61820833	G	A	0.35	0.10	0.01	6.31E-29
IFNGR2	rs12149545	16	56959249	A	G	0.32	0.05	0.01	2.03E-09
IFNGR2	rs72802342	16	75200974	A	C	0.08	-0.07	0.01	6.24E-06
IFNGR2	rs756350040	19	19259531	T	A	0.07	-0.07	0.01	2.76E-05
IFNGR2	rs1065853	19	44909976	T	G	0.08	0.11	0.01	2.48E-12
IFNGR2	rs492602	19	48703160	G	A	0.51	-0.05	0.01	7.69E-11
IFNGR2	rs9808753	21	33415005	G	A	0.14	1.43	0.01	0.00E+00
IFNL1	rs35667974	2	162268127	C	T	0.02	-0.32	0.03	4.77E-16
IFNL1	rs3775291	4	186082920	T	C	0.30	-0.09	0.01	1.08E-13
IFNL1	rs9270747	6	32600515	G	A	0.66	0.07	0.01	4.92E-09
IFNL1	rs10642330	6	166955046	T	C	0.47	-0.06	0.01	3.88E-06
IFNL1	rs10424978	19	4837545	A	C	0.60	-0.06	0.01	4.00E-06
IFNL1	rs28668750	19	39329162	T	G	0.11	-0.09	0.01	7.50E-06
IFNLR1	rs139958347	1	24187233	C	G	0.04	-0.94	0.02	1.14E-188
IFNLR1	rs10753239	1	30889377	C	T	0.46	-0.06	0.01	9.29E-07
IFNLR1	rs1801689	17	66214462	C	A	0.03	0.24	0.02	7.38E-13
IGF1R	rs9269047	6	32471006	G	C	0.85	-0.12	0.01	2.37E-14
IGF1R	rs2519093	9	133266456	T	C	0.18	-0.48	0.01	1.35E-251
IGF1R	rs3184504	12	111446804	C	T	0.52	-0.06	0.01	8.68E-08
IGF1R	rs3743249	15	98962194	T	G	0.25	0.12	0.01	4.40E-22

IGF1R	rs11574938	16	30474072	C	G	0.52	0.09	0.01	5.64E-18
IGF1R	rs200489612	17	7203059	A	G	0.01	0.89	0.05	4.76E-29
IGF1R	rs9916257	17	35470352	T	G	0.44	-0.06	0.01	5.46E-09
IGF1R	rs34006614	19	16331971	T	C	0.33	-0.07	0.01	4.90E-09
IGF1R	rs760462	21	44908184	C	T	0.82	0.08	0.01	3.51E-08
IGF2R	rs6727484	2	112057425	A	G	0.17	0.07	0.01	4.66E-07
IGF2R	rs1265754	6	32335915	A	T	0.13	0.13	0.01	4.35E-14
IGF2R	rs2282140	6	160073489	T	C	0.13	0.55	0.01	5.97E-240
IGF2R	rs9987289	8	9325848	G	A	0.91	-0.09	0.01	4.35E-06
IGF2R	rs72752535	9	114592585	C	A	0.01	0.42	0.05	3.44E-07
IGF2R	rs7974486	12	8979368	G	A	0.84	0.08	0.01	1.54E-07
IGF2R	rs11111000	12	101756445	T	C	0.09	-0.12	0.01	2.12E-11
IGF2R	rs10144207	14	87967790	T	C	0.35	-0.06	0.01	7.85E-07
IGF2R	rs145078947	14	93186629	T	G	0.00	1.00	0.07	1.52E-22
IGF2R	rs186021206	17	7166093	A	G	0.01	0.36	0.05	7.02E-07
IGF2R	rs1838105	17	46931569	G	A	0.65	-0.05	0.01	1.18E-05
IGF2R	rs1801689	17	66214462	C	A	0.03	0.15	0.02	2.03E-06
IGF2R	rs200378	19	18178824	C	T	0.51	0.06	0.01	1.40E-08
IGF2R	rs746064	21	41209521	A	G	0.38	0.05	0.01	2.45E-05
IGFBP1	rs12740374	1	109274968	T	G	0.22	-0.07	0.01	1.19E-07
IGFBP1	rs1260326	2	27508073	C	T	0.61	-0.08	0.01	8.27E-12
IGFBP2	rs1260326	2	27508073	C	T	0.61	0.07	0.01	2.19E-08
IGFBP2	rs4674100	2	216615701	A	G	0.29	-0.07	0.01	5.54E-08
IGFBP2	rs13108218	4	3442204	G	A	0.62	-0.05	0.01	5.02E-06
IGFBP2	rs34690971	4	148059521	T	A	0.74	0.06	0.01	3.01E-06
IGFBP3	rs4234798	4	7218206	G	T	0.62	0.10	0.01	1.32E-21
IGFBP3	rs71538508	6	32567140	T	C	0.28	0.06	0.01	4.50E-06
IGFBP3	rs2854746	7	45921046	C	G	0.40	0.45	0.01	0.00E+00
IGFBP3	rs9987289	8	9325848	G	A	0.91	-0.08	0.01	5.57E-06
IGFBP3	rs73485896	16	1816338	A	G	0.07	0.21	0.01	3.37E-26
IGFBP3	rs738409	22	43928847	G	C	0.22	-0.07	0.01	1.37E-07
IGFBP4	rs13146355	4	76490987	A	G	0.46	0.06	0.01	2.95E-07
IGFBP4	rs10265221	7	151717243	C	T	0.29	0.05	0.01	1.40E-05
IGFBP4	rs28640218	16	20347945	T	G	0.19	-0.07	0.01	8.01E-07
IGFBP6	rs13146355	4	76490987	A	G	0.46	0.05	0.01	1.18E-06
IGFBP6	rs10265221	7	151717243	C	T	0.29	0.06	0.01	8.14E-07
IGFBP6	rs10876406	12	53107653	C	T	0.10	-0.14	0.01	1.49E-16
IGFBP6	rs4997081	16	20353912	C	G	0.19	-0.08	0.01	1.33E-10
IGFBP7	rs1718860	4	57083351	A	G	0.73	-0.37	0.01	6.26E-208
IGFBP7	rs3104394	6	32699743	A	G	0.86	-0.12	0.01	2.20E-16
IGFBP7	rs7282172	21	45026946	G	C	0.17	0.06	0.01	4.81E-06
IGFBP7	rs738409	22	43928847	G	C	0.22	0.06	0.01	5.60E-06
IGFBPL1	rs1260326	2	27508073	C	T	0.61	-0.07	0.01	9.82E-11
IGFBPL1	rs7857243	9	38409084	A	G	0.81	0.49	0.01	1.17E-286
IGFBPL1	-	12	57001714	G	A	0.08	0.10	0.01	9.47E-08
IGSF3	rs2284860	1	116580265	C	T	0.22	0.11	0.01	3.50E-15
IGSF3	rs7529665	1	205155743	A	G	0.23	0.06	0.01	2.51E-05
IGSF3	rs8176749	9	133255801	T	C	0.06	0.14	0.02	1.52E-09
IGSF3	rs56278466	10	17833858	G	T	0.66	0.15	0.01	1.27E-38
IGSF3	rs3747207	22	43928975	A	G	0.22	0.07	0.01	2.52E-06
IGSF8	rs12408242	1	160092144	C	T	0.22	0.16	0.01	7.71E-30
IGSF8	rs6967348	7	129607293	A	G	0.91	-0.09	0.01	2.17E-05
IGSF8	rs6993770	8	105569300	T	A	0.29	-0.09	0.01	1.53E-11
IGSF8	rs28365809	11	130159766	C	T	0.15	-0.13	0.01	1.09E-15
IGSF8	rs892090	19	55027704	G	T	0.83	0.08	0.01	1.07E-06
IGSF8	rs28823595	20	3724486	A	G	0.09	0.09	0.01	2.76E-05

IKBKG	rs1354034	3	56815721	C	T	0.60	0.05	0.01	2.08E-05
IKZF2	rs34136174	6	32615905	C	A	0.43	0.08	0.01	2.30E-10
IKZF2	rs762465	22	22220463	T	C	0.64	0.11	0.01	3.13E-18
IL10	rs2476601	1	113834946	G	A	0.90	-0.08	0.01	1.65E-05
IL10	rs3024495	1	206769068	T	C	0.15	-0.14	0.01	8.35E-19
IL10	rs67619842	6	32568308	T	A	0.12	0.18	0.01	6.20E-24
IL10	rs3135932	11	117993348	G	A	0.17	0.26	0.01	5.16E-65
IL10	rs7203793	16	11088277	G	C	0.36	-0.07	0.01	6.30E-08
IL10	rs9303280	17	39917778	C	T	0.50	0.05	0.01	1.25E-05
IL10RA	rs3135932	11	117993348	G	A	0.17	0.19	0.01	7.54E-34
IL10RB	rs7532110	1	47496292	A	T	0.80	0.06	0.01	1.51E-05
IL10RB	rs61747728	1	179557079	T	C	0.04	0.19	0.02	1.80E-12
IL10RB	rs532436	9	133274414	A	G	0.18	-0.08	0.01	2.32E-08
IL10RB	rs777834943	16	20355409	T	C	0.18	-0.06	0.01	1.04E-05
IL10RB	rs186021206	17	7166093	A	G	0.01	0.40	0.05	1.85E-08
IL10RB	rs2659005	17	81244914	T	C	0.44	0.07	0.01	4.76E-11
IL10RB	rs2247960	21	33288840	G	A	0.45	0.52	0.01	0.00E+00
IL11	rs188468174	1	24965206	T	C	0.01	0.23	0.03	1.08E-05
IL12A_IL12B	rs12471768	2	64701469	C	T	0.71	0.05	0.01	9.88E-06
IL12A_IL12B	rs13025330	2	111093635	T	C	0.22	-0.06	0.01	3.65E-06
IL12A_IL12B	rs72852162	2	144728756	C	A	0.11	0.08	0.01	5.83E-06
IL12A_IL12B	rs11130215	3	4984323	G	A	0.17	0.10	0.01	7.41E-12
IL12A_IL12B	rs9815073	3	188397894	A	C	0.34	0.30	0.01	2.64E-156
IL12A_IL12B	rs4244437	5	159346109	A	G	0.68	0.54	0.01	0.00E+00
IL12A_IL12B	rs9265987	6	31348511	C	T	0.32	0.10	0.01	1.32E-20
IL12A_IL12B	rs836559	7	6409132	C	G	0.46	0.07	0.01	1.04E-10
IL12A_IL12B	rs4641365	10	23377333	T	C	0.21	-0.06	0.01	1.87E-06
IL12A_IL12B	rs11039216	11	47385041	T	C	0.53	0.05	0.01	1.00E-05
IL12A_IL12B	rs561877	11	96286090	A	G	0.32	-0.06	0.01	7.50E-07
IL12A_IL12B	rs10876864	12	56007301	A	G	0.57	-0.07	0.01	3.98E-11
IL12A_IL12B	rs3184504	12	111446804	C	T	0.52	-0.13	0.01	7.52E-36
IL12A_IL12B	rs76428106	13	28029870	C	T	0.01	0.38	0.03	2.14E-15
IL12A_IL12B	-	14	68710109	G	T	0.26	0.06	0.01	5.12E-07
IL12A_IL12B	rs12588969	14	102764421	C	G	0.68	-0.11	0.01	9.89E-22
IL12A_IL12B	rs1987102	15	79973546	C	T	0.26	-0.07	0.01	5.31E-10
IL12A_IL12B	rs7251900	19	55278914	C	T	0.67	-0.05	0.01	1.13E-05
IL12B	rs58394161	1	92474402	C	T	0.15	0.07	0.01	7.26E-06
IL12B	rs2422287	2	64717631	T	C	0.68	0.05	0.01	8.70E-06
IL12B	rs13025330	2	111093635	T	C	0.22	-0.06	0.01	7.06E-06
IL12B	rs72852162	2	144728756	C	A	0.11	0.08	0.01	3.36E-07
IL12B	rs11130215	3	4984323	G	A	0.17	0.09	0.01	1.52E-10
IL12B	rs9815073	3	188397894	A	C	0.34	0.30	0.01	1.32E-160
IL12B	rs11739135	5	132397705	C	G	0.41	0.05	0.01	1.48E-05
IL12B	rs4244437	5	159346109	A	G	0.68	0.56	0.01	0.00E+00
IL12B	rs2394982	6	31345253	T	C	0.31	0.11	0.01	2.45E-21
IL12B	rs836558	7	6408360	A	C	0.46	0.07	0.01	3.29E-11
IL12B	rs4427476	10	23381873	T	G	0.20	-0.06	0.01	4.07E-06
IL12B	rs11039216	11	47385041	T	C	0.53	0.05	0.01	2.08E-06
IL12B	rs502822	11	96289325	T	C	0.30	-0.06	0.01	1.98E-07
IL12B	rs10876864	12	56007301	A	G	0.57	-0.07	0.01	2.02E-11
IL12B	rs3184504	12	111446804	C	T	0.52	-0.13	0.01	9.14E-38
IL12B	rs76428106	13	28029870	C	T	0.01	0.38	0.03	3.95E-16
IL12B	rs374173	14	68715402	C	G	0.48	-0.05	0.01	3.85E-07
IL12B	rs12588969	14	102764421	C	G	0.68	-0.11	0.01	2.17E-22
IL12B	rs1987102	15	79973546	C	T	0.26	-0.08	0.01	8.48E-11
IL12B	rs73068668	19	55251894	A	G	0.08	0.09	0.01	5.67E-06

IL12RB1	rs2844619	6	31274446	G	C	0.61	0.08	0.01	1.23E-13
IL12RB1	rs950802	11	60385111	A	G	0.31	0.28	0.01	2.00E-120
IL12RB1	rs35350651	12	111469627	C	A	0.50	-0.08	0.01	2.29E-13
IL12RB1	rs12720356	19	10359299	C	A	0.10	-0.13	0.01	3.77E-12
IL12RB1	rs447009	19	18074382	A	C	0.21	-0.46	0.01	3.44E-249
IL13	rs34118068	14	105968520	A	G	0.11	0.15	0.01	4.90E-14
IL13RA1	rs139130389	11	72139110	A	C	0.07	-0.14	0.01	2.58E-10
IL13RA1	rs123698	19	807442	C	G	0.61	0.07	0.01	9.71E-10
IL13RA1	rs73201506	21	31672095	G	A	0.05	-0.14	0.02	8.07E-07
IL15	rs149968614	1	64846685	T	C	0.00	0.67	0.09	1.27E-06
IL15	rs6693121	1	101279077	A	C	0.41	0.05	0.01	7.18E-06
IL15	rs2009581	2	111050100	A	G	0.27	0.14	0.01	3.35E-29
IL15	rs1058315	2	231709234	C	G	0.23	-0.07	0.01	1.31E-06
IL15	rs4683324	3	47209794	C	T	0.58	-0.08	0.01	1.24E-12
IL15	rs9844706	3	128661938	A	G	0.89	-0.08	0.01	4.01E-06
IL15	rs3131623	6	31451033	A	T	0.18	0.15	0.01	1.43E-25
IL15	rs173288	6	109205086	G	C	0.58	-0.05	0.01	4.80E-06
IL15	rs78997768	8	59167516	T	G	0.16	-0.07	0.01	2.41E-06
IL15	rs1378901	8	127201562	C	T	0.44	0.05	0.01	6.44E-06
IL15	rs8177636	10	5976631	C	T	0.43	-0.06	0.01	1.18E-06
IL15	rs2646420	10	8425074	T	C	0.38	-0.06	0.01	5.17E-08
IL15	rs1926197	10	88988869	G	A	0.45	-0.06	0.01	7.67E-07
IL15	rs12363646	11	128197447	C	T	0.29	-0.06	0.01	1.96E-06
IL15	rs7310615	12	111427245	G	C	0.52	0.09	0.01	2.88E-15
IL15	rs76428106	13	28029870	C	T	0.01	-0.30	0.03	8.57E-09
IL15	rs11632933	15	70451887	A	G	0.62	0.05	0.01	6.39E-06
IL15	rs9916257	17	35470352	T	G	0.44	0.15	0.01	1.34E-37
IL15	rs3212802	19	17826295	T	G	0.18	0.09	0.01	1.89E-09
IL15	rs7268811	20	36922468	A	C	0.17	0.07	0.01	8.75E-06
IL15	rs35477491	21	13901496	T	C	0.22	0.07	0.01	1.12E-05
IL15	rs228951	22	37133758	G	A	0.37	0.07	0.01	7.66E-10
IL15RA	rs539115936	5	96887303	T	C	0.47	-0.06	0.01	1.43E-07
IL15RA	rs1050486	6	31356925	T	C	0.62	0.06	0.01	5.05E-07
IL15RA	-	9	133263362	G	A	0.18	-0.08	0.01	1.06E-07
IL15RA	rs3136630	10	5955857	T	C	0.30	-0.26	0.01	8.51E-96
IL15RA	rs111338191	12	111388673	T	A	0.52	-0.05	0.01	1.89E-06
IL16	rs12342201	9	93132682	A	G	0.48	-0.06	0.01	1.27E-09
IL16	rs58754882	15	81303017	A	G	0.09	-0.96	0.01	0.00E+00
IL17C	rs144937970	3	9910943	A	G	0.01	0.34	0.04	5.59E-08
IL17C	rs8177833	5	151087129	G	C	0.06	0.11	0.02	1.70E-05
IL17C	rs17700884	16	88618087	T	G	0.36	-0.12	0.01	1.28E-24
IL17D	rs4770059	13	20576908	C	T	0.74	-0.40	0.01	5.22E-246
IL17D	rs77542162	17	69085137	G	A	0.02	0.24	0.02	1.45E-12
IL17D	rs1065853	19	44909976	T	G	0.08	0.08	0.01	2.59E-05
IL17F	rs66834286	6	52241065	T	C	0.11	-0.26	0.01	1.51E-42
IL17F	rs10152051	14	106116963	G	A	0.45	-0.07	0.01	1.36E-07
IL17RA	rs78689694	11	126364925	C	G	0.13	-0.07	0.01	1.45E-07
IL17RA	rs186021206	17	7166093	A	G	0.01	0.49	0.04	6.41E-18
IL17RA	rs4819959	22	17105741	A	G	0.50	0.85	0.01	0.00E+00
IL17RB	rs1260326	2	27508073	C	T	0.61	0.06	0.01	9.91E-10
IL17RB	rs6445607	3	53843122	T	G	0.62	-0.80	0.01	0.00E+00
IL17RB	rs7643425	3	171019606	G	A	0.11	-0.08	0.01	6.87E-06
IL17RB	rs34690971	4	148059521	T	A	0.74	0.05	0.01	1.78E-05
IL17RB	rs2894204	6	31269284	T	C	0.63	-0.07	0.01	2.57E-12
IL17RB	rs34346326	7	73601851	C	T	0.20	0.05	0.01	2.24E-06
IL17RB	rs1870148	10	80511585	A	G	0.79	0.05	0.01	1.36E-06

IL17RB	rs10841753	12	21168436	C	T	0.19	0.06	0.01	2.70E-08
IL17RB	rs1169284	12	120982123	C	T	0.31	-0.06	0.01	1.07E-08
IL17RB	rs28929474	14	94378610	T	C	0.02	0.23	0.02	1.82E-13
IL17RB	rs77542162	17	69085137	G	A	0.02	0.15	0.02	6.25E-07
IL18	rs385076	2	32264782	C	T	0.64	0.22	0.01	1.77E-84
IL18	rs77762937	2	202602747	T	G	0.29	0.06	0.01	2.89E-06
IL18	-	5	69992376	C	A	0.06	0.24	0.02	2.38E-21
IL18	rs130066	6	31154538	C	G	0.46	-0.05	0.01	2.33E-06
IL18	rs2290414	8	143561433	A	G	0.44	-0.07	0.01	3.19E-11
IL18	rs117558678	11	270330	T	A	0.24	0.07	0.01	2.75E-08
IL18	rs17103763	11	105133145	A	T	0.26	0.11	0.01	2.65E-17
IL18	rs2250417	11	112214593	C	T	0.54	-0.29	0.01	7.10E-155
IL18	rs10675507	12	47838013	T	C	0.52	-0.05	0.01	9.92E-06
IL18	rs7137828	12	111494996	T	C	0.52	-0.07	0.01	9.68E-10
IL18	rs10418046	19	53824615	G	T	0.21	0.06	0.01	1.53E-06
IL18BP	rs61747728	1	179557079	T	C	0.04	0.17	0.02	6.74E-09
IL18BP	rs3132455	6	31520177	C	G	0.35	0.05	0.01	2.42E-05
IL18BP	rs10901252	9	133252613	C	G	0.06	0.16	0.01	1.29E-11
IL18BP	rs5743659	11	71998832	A	G	0.01	-0.55	0.05	1.74E-14
IL18BP	rs766175824	11	126356659	G	A	0.07	0.14	0.01	6.34E-11
IL18BP	rs10774625	12	111472415	G	A	0.50	-0.12	0.01	6.75E-28
IL18BP	rs4767937	12	120785441	C	G	0.47	-0.05	0.01	3.83E-06
IL18BP	rs186021206	17	7166093	A	G	0.01	0.86	0.05	8.79E-31
IL18BP	rs1292045	17	59869851	C	T	0.47	-0.05	0.01	2.76E-05
IL18BP	rs10445407	17	81288009	A	C	0.48	-0.05	0.01	8.47E-06
IL18R1	-	1	161704318	G	A	0.11	0.09	0.01	1.32E-08
IL18R1	rs10190555	2	102377596	G	A	0.77	-0.89	0.01	0.00E+00
IL18R1	rs407258	3	155843189	C	T	0.25	-0.07	0.01	4.88E-10
IL18R1	rs28636815	4	76276244	G	A	0.38	-0.04	0.01	4.93E-06
IL18R1	rs73015965	6	160706469	G	A	0.01	0.27	0.04	2.24E-05
IL18R1	-	7	35695033	C	G	0.42	-0.04	0.01	2.10E-05
IL18R1	rs2519093	9	133266456	T	C	0.18	-0.05	0.01	1.83E-05
IL18R1	rs56278466	10	17833858	G	T	0.66	0.11	0.01	2.22E-32
IL18R1	rs111338191	12	111388673	T	A	0.52	-0.04	0.01	2.57E-06
IL18R1	rs7970695	12	120985573	A	G	0.62	-0.05	0.01	5.46E-07
IL18R1	rs28929474	14	94378610	T	C	0.02	0.26	0.02	2.62E-16
IL18R1	rs147233090	15	43735849	T	C	0.02	0.15	0.02	2.47E-06
IL18R1	rs77303550	16	72045758	T	C	0.20	-0.07	0.01	1.65E-08
IL18R1	rs1801689	17	66214462	C	A	0.03	0.43	0.02	7.79E-60
IL18R1	rs12019136	19	5835666	A	G	0.04	0.11	0.01	1.04E-06
IL18RAP	rs72490060	2	88998554	T	C	0.19	0.12	0.01	2.17E-12
IL18RAP	rs4110740	2	94747462	T	C	0.21	0.08	0.01	1.17E-07
IL18RAP	rs9380143	6	29834268	T	C	0.28	-0.08	0.01	2.59E-09
IL18RAP	rs4774021	14	106809925	T	G	0.14	0.16	0.01	2.07E-21
IL19	rs2243191	1	206842612	C	T	0.78	0.35	0.01	6.60E-158
IL19	rs80235202	6	32536054	G	A	0.28	0.06	0.01	1.27E-05
IL19	rs34819998	11	126351095	C	G	0.15	0.08	0.01	4.38E-06
IL19	rs2608894	19	5847989	C	T	0.76	-0.07	0.01	3.46E-07
IL19	rs492602	19	48703160	G	A	0.51	0.38	0.01	3.10E-250
IL1A	rs28464365	6	32619273	C	T	0.04	-0.27	0.02	3.63E-21
IL1B	rs6993770	8	105569300	T	A	0.29	-0.08	0.01	4.02E-09
IL1B	rs34436714	19	53824059	A	C	0.21	0.29	0.01	3.53E-97
IL1R1	rs61830291	1	220827800	C	A	0.10	-0.12	0.01	7.21E-11
IL1R1	rs6545929	2	62383044	A	G	0.19	-0.08	0.01	7.47E-09
IL1R1	rs3917238	2	102156623	T	C	0.30	-0.18	0.01	5.37E-52
IL1R1	rs55709272	2	113109711	C	T	0.44	0.14	0.01	1.14E-33

IL1R1	rs10935473	3	98698056	T	G	0.44	-0.29	0.01	2.18E-148
IL1R1	rs1634775	6	31335459	G	A	0.62	-0.07	0.01	6.38E-11
IL1R1	rs9987289	8	9325848	G	A	0.91	-0.08	0.01	1.27E-05
IL1R1	rs8176672	9	133266772	T	C	0.06	0.21	0.01	7.48E-20
IL1R1	rs56278466	10	17833858	G	T	0.66	0.06	0.01	3.23E-08
IL1R1	rs28929474	14	94378610	T	C	0.02	0.25	0.03	1.23E-10
IL1R1	rs186021206	17	7166093	A	G	0.01	0.59	0.05	7.88E-15
IL1R1	rs2292642	17	78399349	T	C	0.60	-0.07	0.01	5.56E-10
IL1R1	rs67525224	21	41255293	C	G	0.19	0.11	0.01	5.07E-14
IL1R2	rs165316	1	91067740	G	A	0.20	-0.06	0.01	2.32E-06
IL1R2	rs629301	1	109275684	T	G	0.78	-0.07	0.01	6.09E-08
IL1R2	rs2310170	2	101999692	G	T	0.40	-0.51	0.01	0.00E+00
IL1R2	rs9274718	6	32669692	C	T	0.38	0.07	0.01	3.91E-10
IL1R2	rs6973520	7	129100389	T	C	0.51	0.07	0.01	1.75E-10
IL1R2	rs28601761	8	125487789	G	C	0.42	-0.12	0.01	1.68E-31
IL1R2	rs1870138	10	80509855	G	A	0.80	0.08	0.01	7.11E-11
IL1R2	rs10831245	11	94586722	A	G	0.39	0.07	0.01	9.19E-10
IL1R2	rs60843925	11	126368937	C	T	0.14	-0.07	0.01	1.45E-05
IL1R2	rs11045856	12	21197755	G	T	0.24	0.07	0.01	1.18E-07
IL1R2	rs1169296	12	120990604	G	A	0.33	0.07	0.01	6.59E-11
IL1R2	rs11621792	14	24402720	T	C	0.45	-0.05	0.01	7.80E-06
IL1R2	rs186021206	17	7166093	A	G	0.01	0.48	0.05	1.85E-11
IL1R2	rs77542162	17	69085137	G	A	0.02	0.17	0.02	2.19E-06
IL1RAP	rs6444442	3	190628271	G	A	0.85	-1.39	0.01	0.00E+00
IL1RAP	-	4	99103313	T	A	0.73	0.04	0.01	1.86E-06
IL1RAP	rs753950345	5	132882930	A	C	0.16	0.06	0.01	1.89E-08
IL1RAP	rs146812806	8	18414993	G	C	0.78	-0.05	0.01	2.04E-09
IL1RAP	rs7310409	12	120987058	G	A	0.62	0.04	0.00	3.48E-07
IL1RAP	rs139974673	15	43735687	C	T	0.02	0.19	0.02	2.15E-15
IL1RAP	rs273510	19	18112540	G	A	0.29	-0.04	0.01	1.09E-07
IL1RL1	rs13028833	2	61417194	G	C	0.52	0.06	0.01	1.24E-08
IL1RL1	rs12470864	2	102309902	A	G	0.39	-0.68	0.01	0.00E+00
IL1RL1	rs1257221	2	134259047	C	A	0.76	-0.06	0.01	3.30E-07
IL1RL1	rs7616330	3	71066600	A	C	0.17	-0.05	0.01	5.28E-06
IL1RL1	rs9829963	3	143181686	C	T	0.21	0.07	0.01	3.34E-11
IL1RL1	rs13107325	4	102267552	T	C	0.08	0.11	0.01	3.43E-12
IL1RL1	rs2519093	9	133266456	T	C	0.18	-0.09	0.01	5.76E-17
IL1RL1	rs11603123	11	126435600	A	G	0.03	0.38	0.02	1.30E-58
IL1RL1	rs1378942	15	74785026	A	C	0.68	0.05	0.01	4.03E-08
IL1RL1	rs12447180	16	88451314	C	T	0.33	-0.06	0.01	1.95E-10
IL1RL1	rs186021206	17	7166093	A	G	0.01	0.96	0.04	1.50E-61
IL1RL1	rs58526981	17	60919686	T	A	0.16	-0.06	0.01	1.59E-07
IL1RL1	rs2899319	22	39478309	C	A	0.62	0.04	0.01	7.31E-07
IL1RL2	rs10779835	1	230164203	C	T	0.61	-0.05	0.01	1.27E-05
IL1RL2	rs3917265	2	102162001	C	T	0.54	-0.44	0.01	0.00E+00
IL1RL2	rs11928797	3	33416001	A	C	0.12	0.08	0.01	1.65E-06
IL1RL2	rs13203302	6	95559822	T	C	0.08	0.11	0.01	3.58E-09
IL1RL2	rs10815254	9	5630344	T	C	0.28	0.05	0.01	9.98E-06
IL1RL2	rs56278466	10	17833858	G	T	0.66	0.07	0.01	8.11E-10
IL1RL2	rs2291075	12	21178691	T	C	0.39	0.05	0.01	2.92E-06
IL1RL2	rs11065385	12	120985583	G	A	0.69	-0.16	0.01	2.11E-49
IL1RL2	rs17774889	14	49593139	C	G	0.55	0.06	0.01	5.28E-08
IL1RL2	rs28929474	14	94378610	T	C	0.02	0.22	0.02	2.07E-09
IL1RL2	rs62011287	15	63499029	G	A	0.34	-0.05	0.01	1.00E-06
IL1RL2	rs7599	19	35547488	G	A	0.63	-0.06	0.01	9.20E-08
IL1RN	rs55709272	2	113109711	C	T	0.44	-0.21	0.01	3.49E-75

IL1RN	rs12941811	17	40003082	C	T	0.57	-0.06	0.01	1.16E-06
IL20RA	rs143005532	6	137009349	T	C	0.00	0.86	0.10	1.18E-08
IL22RA1	rs7355101	1	24124595	T	A	0.20	-0.31	0.01	1.63E-105
IL22RA1	-	9	133263362	G	A	0.18	-0.12	0.01	4.52E-17
IL22RA1	rs681343	19	48703205	T	C	0.51	-0.15	0.01	3.84E-37
IL2RA	rs61747728	1	179557079	T	C	0.04	0.13	0.02	1.20E-06
IL2RA	rs734866	3	24434006	C	A	0.19	0.09	0.01	3.38E-11
IL2RA	rs3094005	6	31497270	T	G	0.14	0.12	0.01	2.79E-16
IL2RA	rs12722497	10	6053965	A	C	0.09	0.95	0.01	0.00E+00
IL2RA	rs7137828	12	111494996	T	C	0.52	-0.08	0.01	2.71E-16
IL2RA	rs1955512	14	32706616	A	G	0.57	0.06	0.01	1.86E-09
IL2RA	rs924135	16	16029602	T	A	0.61	-0.06	0.01	6.49E-09
IL2RA	rs2659007	17	81243678	A	G	0.45	0.06	0.01	7.55E-08
IL32	rs9272154	6	32633242	A	G	0.35	0.05	0.01	1.98E-05
IL32	rs117199990	8	19963405	T	C	0.10	0.10	0.01	2.09E-08
IL32	rs145920606	12	7474715	G	A	0.08	0.10	0.01	1.96E-06
IL32	rs12905732	15	58391343	C	G	0.20	0.09	0.01	1.21E-11
IL32	rs45499297	16	3065271	C	T	0.09	-0.40	0.01	2.03E-97
IL32	rs247617	16	56956804	A	C	0.32	0.10	0.01	3.30E-18
IL32	rs429358	19	44908684	C	T	0.16	0.09	0.01	7.86E-09
IL32	rs12986064	19	54251270	C	T	0.51	-0.07	0.01	1.42E-09
IL32	rs3747207	22	43928975	A	G	0.22	0.06	0.01	1.24E-05
IL33	rs143215670	9	6264551	C	G	0.00	-0.38	0.06	2.05E-05
IL34	rs4985556	16	70660097	A	C	0.12	-1.07	0.01	0.00E+00
IL3RA	rs876038	7	50268931	T	C	0.32	-0.05	0.01	7.42E-06
IL3RA	-	9	133263362	G	A	0.18	-0.59	0.01	0.00E+00
IL3RA	rs508487	11	117204850	T	C	0.06	0.10	0.01	2.33E-05
IL3RA	rs35458154	11	126426930	A	G	0.03	0.25	0.02	2.86E-13
IL3RA	rs186021206	17	7166093	A	G	0.01	0.75	0.05	3.45E-25
IL4R	rs10935473	3	98698056	T	G	0.44	-0.05	0.01	2.08E-06
IL4R	rs541020703	7	34921504	A	T	0.32	0.13	0.01	2.72E-29
IL4R	rs2519093	9	133266456	T	C	0.18	-0.08	0.01	4.02E-09
IL4R	rs149425950	12	20847698	T	A	0.15	0.08	0.01	2.59E-07
IL4R	rs28929474	14	94378610	T	C	0.02	0.47	0.03	4.27E-35
IL4R	rs8060025	16	27315893	G	T	0.61	0.31	0.01	5.53E-169
IL4R	rs186021206	17	7166093	A	G	0.01	0.64	0.05	3.31E-17
IL5RA	rs188468174	1	24965206	T	C	0.01	-0.72	0.03	2.27E-61
IL5RA	rs77400868	3	3109280	G	A	0.13	0.51	0.01	3.70E-214
IL5RA	rs9819371	3	141487958	T	C	0.07	-0.11	0.01	3.25E-06
IL5RA	rs9869437	3	196501489	A	C	0.35	0.08	0.01	2.04E-10
IL5RA	rs93059	4	102547361	A	G	0.44	-0.05	0.01	5.89E-07
IL5RA	rs6810926	4	146047700	G	A	0.17	0.07	0.01	4.74E-08
IL5RA	rs561074184	5	95923240	A	G	0.26	-0.06	0.01	6.26E-08
IL5RA	rs7705189	5	132287665	G	A	0.47	-0.07	0.01	1.14E-10
IL5RA	rs3134745	6	31274985	T	C	0.33	-0.12	0.01	3.19E-30
IL5RA	rs66867810	6	41973148	A	G	0.32	-0.06	0.01	6.96E-07
IL5RA	-	6	139307965	G	A	0.41	0.06	0.01	5.41E-09
IL5RA	rs7758383	6	142848586	G	A	0.49	-0.06	0.01	1.16E-07
IL5RA	rs7797255	7	50312008	G	A	0.40	-0.07	0.01	1.59E-10
IL5RA	rs78740585	7	151247216	A	G	0.11	0.19	0.01	5.95E-34
IL5RA	rs2511713	8	102565637	G	A	0.26	-0.06	0.01	4.31E-08
IL5RA	rs12342831	9	33124874	C	T	0.25	-0.05	0.01	7.77E-06
IL5RA	rs968567	11	61828092	T	C	0.19	0.06	0.01	2.18E-05
IL5RA	rs10896045	11	65788053	G	A	0.70	-0.05	0.01	4.67E-06
IL5RA	rs12880641	14	102785115	G	T	0.66	-0.06	0.01	5.03E-08
IL5RA	rs71374030	16	30719197	T	A	0.74	-0.09	0.01	2.35E-13

IL5RA	rs3803800	17	7559652	G	A	0.79	-0.07	0.01	1.53E-08
IL5RA	rs34562254	17	16939677	A	G	0.10	0.20	0.01	3.39E-30
IL5RA	rs59716545	17	39875604	G	T	0.48	-0.09	0.01	1.73E-17
IL5RA	rs4794063	17	47727128	T	C	0.26	-0.05	0.01	2.61E-05
IL5RA	rs11085015	19	3369574	G	T	0.80	-0.06	0.01	2.80E-05
IL5RA	rs1005980	19	19160689	G	C	0.46	-0.07	0.01	1.45E-12
IL5RA	rs386812017	20	4989627	T	G	0.39	0.05	0.01	5.85E-07
IL6	rs55709272	2	113109711	C	T	0.44	0.05	0.01	2.26E-06
IL6	rs12133641	1	154455807	G	A	0.41	0.23	0.01	1.80E-92
IL6	rs7449585	6	32612814	C	G	0.27	0.08	0.01	8.91E-10
IL6	rs9977672	21	39091357	A	G	0.26	-0.06	0.01	4.44E-06
IL6R	rs4129267	1	154453788	T	C	0.40	1.10	0.01	0.00E+00
IL6R	rs11757137	6	32552380	T	C	0.44	0.04	0.00	8.00E-07
IL6R	rs4055121	11	126362442	T	C	0.13	-0.05	0.01	2.02E-06
IL6R	rs7137828	12	111494996	T	C	0.52	-0.03	0.00	2.04E-05
IL6R	rs186021206	17	7166093	A	G	0.01	0.42	0.03	2.90E-17
IL6R	rs760482467	17	59791184	C	T	0.58	0.04	0.00	1.99E-07
IL6R	rs351988	19	815086	G	A	0.57	0.04	0.00	2.95E-06
IL6ST	rs3791101	1	43900578	A	G	0.31	0.06	0.01	5.39E-06
IL6ST	rs10935473	3	98698056	T	G	0.44	-0.17	0.01	1.61E-55
IL6ST	rs138283229	3	155828335	T	C	0.01	0.38	0.05	9.20E-07
IL6ST	rs11739016	5	55967545	T	C	0.12	0.26	0.01	8.71E-52
IL6ST	rs4721064	7	12244752	A	G	0.40	0.05	0.01	1.94E-05
IL6ST	rs507666	9	133273983	A	G	0.18	-0.21	0.01	2.18E-51
IL6ST	rs11011672	10	19916136	T	G	0.70	0.06	0.01	2.97E-06
IL6ST	rs3967200	11	126362490	T	C	0.13	-0.15	0.01	1.31E-19
IL6ST	rs7145500	14	65417494	A	G	0.67	0.05	0.01	1.99E-05
IL6ST	rs186021206	17	7166093	A	G	0.01	1.21	0.05	4.19E-58
IL6ST	rs77542162	17	69085137	G	A	0.02	0.16	0.02	2.63E-05
IL6ST	rs11444782	20	3699816	T	A	0.56	-0.06	0.01	1.22E-06
IL6ST	rs2008174	22	39464125	T	C	0.74	-0.08	0.01	8.38E-09
IL7	rs3804749	3	123114156	T	C	0.59	-0.07	0.01	4.52E-08
IL7	rs11734132	4	6889792	C	G	0.17	0.07	0.01	2.62E-05
IL7	rs3207561	6	31268908	G	A	0.37	0.05	0.01	1.43E-05
IL7	-	8	78816116	T	A	0.10	-0.18	0.01	5.60E-20
IL7	rs6993770	8	105569300	T	A	0.29	-0.11	0.01	6.73E-17
IL7R	rs36053809	1	26756602	C	A	0.08	-0.08	0.01	3.43E-06
IL7R	rs3791101	1	43900578	A	G	0.31	0.06	0.01	1.37E-08
IL7R	rs9850919	3	169460136	T	C	0.59	0.05	0.01	9.51E-08
IL7R	rs13071610	3	186955149	A	G	0.12	-0.06	0.01	2.28E-05
IL7R	rs6897932	5	35874473	T	C	0.27	-0.88	0.01	0.00E+00
IL7R	rs12189871	6	31284147	T	C	0.09	0.16	0.01	1.47E-25
IL7R	rs170622	7	34992488	T	C	0.21	-0.05	0.01	1.58E-06
IL7R	rs2519093	9	133266456	T	C	0.18	-0.59	0.01	0.00E+00
IL7R	rs1059091	11	309127	G	A	0.32	-0.04	0.01	7.28E-06
IL7R	rs35166255	11	126431861	A	G	0.03	0.47	0.02	1.78E-82
IL7R	rs186021206	17	7166093	A	G	0.01	1.11	0.04	6.19E-77
IL7R	rs11083619	19	41407362	C	A	0.61	0.06	0.01	1.16E-12
ILKAP	rs6993770	8	105569300	T	A	0.29	-0.06	0.01	1.28E-06
ILKAP	rs60822569	12	54323724	C	T	0.55	0.05	0.01	1.43E-05
IMPA1	rs342293	7	106731773	G	C	0.46	-0.05	0.01	2.79E-05
IMPA1	rs1967328	8	81671536	T	G	0.30	-0.21	0.01	4.14E-67
ING1	rs6993770	8	105569300	T	A	0.29	-0.06	0.01	1.15E-05
INHBC	rs1730858	1	107076622	C	T	0.65	0.05	0.01	2.16E-06
INHBC	rs1260326	2	27508073	C	T	0.61	-0.06	0.01	1.79E-08
INHBC	rs204893	6	32126816	C	T	0.43	0.05	0.01	9.15E-06

INHBC	rs7896518	10	63344740	G	A	0.42	-0.06	0.01	1.56E-07
INHBC	rs3741414	12	57450266	T	C	0.25	-0.65	0.01	0.00E+00
INHBC	rs28929474	14	94378610	T	C	0.02	0.19	0.02	4.24E-07
INPP1	rs141472596	2	190286791	G	A	0.00	-1.53	0.10	1.93E-22
INPP1	rs342293	7	106731773	G	C	0.46	-0.05	0.01	6.05E-06
INPP1	rs3847326	10	63572099	G	A	0.48	-0.05	0.01	2.62E-05
IPCEF1	rs1354034	3	56815721	C	T	0.60	0.06	0.01	3.62E-08
IPCEF1	rs2499649	6	154354781	C	T	0.31	-0.09	0.01	2.03E-12
IQGAP2	rs10037254	5	76634815	A	G	0.60	0.15	0.01	1.36E-36
IRAG2	rs7080536	10	113588287	A	G	0.04	-0.13	0.02	4.96E-06
IRAG2	rs72932837	11	65109268	T	C	0.24	-0.08	0.01	1.49E-08
IRAK1	rs1354034	3	56815721	C	T	0.60	0.06	0.01	1.14E-06
IRAK4	-	10	63156270	A	T	0.49	0.05	0.01	1.89E-05
IRAK4	rs183549317	12	43736640	A	G	0.00	-1.09	0.13	2.88E-08
ISLR2	rs35518360	4	102225733	T	A	0.08	0.09	0.01	4.76E-06
ISLR2	rs9263451	6	31072711	A	C	0.10	-0.07	0.01	2.47E-05
ISLR2	rs2519093	9	133266456	T	C	0.18	-0.65	0.01	0.00E+00
ISLR2	rs60843925	11	126368937	C	T	0.14	-0.14	0.01	2.46E-18
ISLR2	rs34868798	15	74177375	C	T	0.22	0.25	0.01	1.64E-83
ISLR2	rs142359784	16	67352232	T	C	0.04	-0.12	0.02	9.55E-06
ISLR2	rs186021206	17	7166093	A	G	0.01	0.96	0.05	3.03E-41
ISLR2	rs4802117	19	41404712	A	G	0.61	0.06	0.01	3.11E-08
ISM1	rs539657009	1	161658666	T	C	0.13	0.08	0.01	1.10E-05
ISM1	rs7641117	3	194343614	G	C	0.67	0.06	0.01	9.83E-07
ISM1	-	4	186236113	C	A	0.51	-0.07	0.01	4.67E-09
ISM1	-	10	63184239	A	T	0.44	0.05	0.01	2.29E-05
ISM1	rs1998123	20	13495237	G	A	0.70	-0.19	0.01	1.25E-53
ITGA11	rs1260326	2	27508073	C	T	0.61	0.06	0.01	6.12E-08
ITGA11	-	10	63464698	C	A	0.52	0.06	0.01	1.67E-07
ITGA11	rs7927820	11	116811440	A	G	0.88	-0.12	0.01	5.39E-12
ITGA11	rs35887873	11	126349501	C	T	0.23	0.12	0.01	6.77E-20
ITGA11	rs753768034	15	58389334	T	G	0.62	-0.07	0.01	6.98E-10
ITGA11	rs2306022	15	68335825	T	C	0.09	-0.36	0.01	5.31E-76
ITGA11	rs186021206	17	7166093	A	G	0.01	0.81	0.05	4.48E-27
ITGA11	-	20	45927136	G	C	0.15	-0.08	0.01	1.67E-07
ITGA5	rs539657009	1	161658666	T	C	0.13	0.09	0.01	4.83E-08
ITGA5	rs2124440	2	181463487	A	G	0.56	0.06	0.01	1.85E-07
ITGA5	rs139078629	2	215386815	A	G	0.01	-0.67	0.04	9.39E-33
ITGA5	rs62246443	3	47130917	C	T	0.18	-0.07	0.01	8.62E-06
ITGA5	rs78776915	6	32501603	T	C	0.42	0.07	0.01	1.02E-07
ITGA5	rs41341748	8	16155085	A	G	0.01	0.24	0.03	9.08E-06
ITGA5	rs8176746	9	133255935	T	G	0.06	0.22	0.01	1.38E-22
ITGA5	rs10748526	10	80513323	C	T	0.79	0.12	0.01	3.77E-17
ITGA5	rs199991349	11	58610344	A	C	0.01	0.22	0.03	7.94E-06
ITGA5	rs3184504	12	111446804	C	T	0.52	-0.11	0.01	6.67E-22
ITGA5	rs7161799	15	58478324	T	C	0.08	0.09	0.01	2.71E-05
ITGA5	rs186021206	17	7166093	A	G	0.01	0.71	0.05	4.54E-21
ITGA5	rs7288265	22	44027516	A	G	0.33	-0.07	0.01	3.81E-10
ITGA6	rs6714597	2	172473758	C	T	0.31	0.23	0.01	3.82E-79
ITGA6	rs710446	3	186742138	C	T	0.41	0.07	0.01	1.59E-10
ITGA6	rs4861708	4	186236079	A	G	0.51	0.17	0.01	8.54E-50
ITGA6	rs2897457	5	53056546	T	G	0.49	-0.12	0.01	2.33E-27
ITGA6	rs2731673	5	177412897	C	T	0.74	0.08	0.01	1.02E-10
ITGA6	rs67612510	6	32558659	A	G	0.54	-0.06	0.01	1.06E-05
ITGA6	rs579459	9	133278724	C	T	0.21	-0.10	0.01	1.50E-13
ITGA6	-	10	63156270	A	T	0.49	0.06	0.01	4.15E-07

ITGA6	-	11	1778277	A	G	0.09	-0.10	0.01	5.80E-07
ITGA6	rs1362213	12	6173262	T	C	0.48	-0.05	0.01	8.43E-06
ITGAM	rs10164604	2	134296795	A	T	0.90	0.08	0.01	1.12E-05
ITGAM	rs118162691	8	21910298	A	C	0.04	0.14	0.02	4.28E-07
ITGAM	rs7865362	9	33117967	T	C	0.36	0.11	0.01	2.17E-20
ITGAM	rs4601794	11	126375250	G	A	0.06	-0.12	0.02	1.73E-06
ITGAM	rs3184504	12	111446804	C	T	0.52	-0.06	0.01	6.29E-07
ITGAM	rs11150613	16	31346489	T	C	0.68	0.16	0.01	1.27E-41
ITGAM	rs776419906	16	48401235	A	G	0.00	-1.04	0.12	3.21E-08
ITGAM	rs186021206	17	7166093	A	G	0.01	1.01	0.05	2.43E-40
ITGAM	rs4804774	19	7707287	C	G	0.24	0.08	0.01	1.94E-09
ITGAM	rs2230531	21	44888885	T	C	0.01	0.50	0.04	5.34E-15
ITGAV	rs61801010	1	161572445	G	A	0.11	0.08	0.01	2.02E-05
ITGAV	rs2595391	2	186667646	G	T	0.11	0.23	0.01	3.80E-40
ITGAV	rs112771035	11	126355981	G	C	0.07	-0.11	0.01	9.89E-08
ITGAV	rs111338191	12	111388673	T	A	0.52	0.05	0.01	8.45E-07
ITGAV	rs17580	14	94380925	A	T	0.05	0.12	0.02	1.55E-06
ITGAV	rs186021206	17	7166093	A	G	0.01	0.87	0.05	4.50E-32
ITGAV	rs2868346	20	45919331	T	C	0.76	-0.05	0.01	2.34E-05
ITGB1	-	1	92568371	C	T	0.79	0.06	0.01	2.14E-05
ITGB1	rs246411	5	52980628	C	T	0.32	0.10	0.01	2.14E-17
ITGB1	rs4841132	8	9326086	G	A	0.91	-0.09	0.01	1.16E-05
ITGB1	rs507666	9	133273983	A	G	0.18	-0.11	0.01	2.83E-14
ITGB1	rs7924036	10	63431885	T	G	0.50	0.05	0.01	6.29E-06
ITGB1	rs60843925	11	126368937	C	T	0.14	-0.13	0.01	9.37E-16
ITGB1	rs186021206	17	7166093	A	G	0.01	1.09	0.05	1.73E-48
ITGB1	rs2413593	22	39476122	G	A	0.49	0.05	0.01	5.38E-06
ITGB1BP2	rs2631367	5	132369766	G	C	0.52	0.05	0.01	9.92E-06
ITGB1BP2	rs342298	7	106733200	T	C	0.46	-0.10	0.01	6.47E-17
ITGB1BP2	rs11553699	12	121779004	G	A	0.14	-0.10	0.01	1.19E-08
ITGB1BP2	rs13338372	16	8944922	G	A	0.19	-0.06	0.01	1.89E-05
ITGB1BP2	rs200309755	17	29518756	T	C	0.54	0.05	0.01	1.95E-05
ITGB2	-	1	161704318	G	A	0.11	0.08	0.01	2.17E-05
ITGB2	rs9784005	2	68348488	A	T	0.44	0.05	0.01	2.69E-05
ITGB2	rs7433300	3	47079150	A	G	0.62	-0.05	0.01	1.92E-05
ITGB2	rs118162691	8	21910298	A	C	0.04	0.14	0.02	4.17E-07
ITGB2	rs7865362	9	33117967	T	C	0.36	0.09	0.01	7.76E-14
ITGB2	rs56278466	10	17833858	G	T	0.66	0.07	0.01	2.10E-08
ITGB2	rs75071241	11	126362291	A	G	0.06	-0.12	0.02	3.23E-07
ITGB2	rs3184504	12	111446804	C	T	0.52	-0.12	0.01	1.21E-29
ITGB2	rs11574639	16	31360760	A	G	0.68	0.12	0.01	6.86E-24
ITGB2	rs776419906	16	48401235	A	G	0.00	-0.99	0.12	9.51E-08
ITGB2	rs186021206	17	7166093	A	G	0.01	1.11	0.05	2.90E-49
ITGB2	rs57543961	17	35475912	T	C	0.42	-0.05	0.01	2.02E-05
ITGB2	rs760462	21	44908184	C	T	0.82	0.15	0.01	2.60E-25
ITGB5	rs35986780	2	186724153	G	A	0.30	-0.06	0.01	1.70E-06
ITGB5	rs1354034	3	56815721	C	T	0.60	0.08	0.01	1.69E-11
ITGB5	rs144195265	3	124790520	A	G	0.03	0.22	0.02	5.79E-12
ITGB5	rs56399423	5	132336964	C	T	0.43	-0.05	0.01	1.24E-05
ITGB5	rs7896518	10	63344740	G	A	0.42	0.09	0.01	2.43E-15
ITGB5	rs139130389	11	72139110	A	C	0.07	-0.43	0.01	8.99E-92
ITGB5	rs186021206	17	7166093	A	G	0.01	0.51	0.05	1.65E-11
ITGB6	rs6432601	2	160121432	G	A	0.69	0.29	0.01	8.62E-134
ITGB6	rs13107325	4	102267552	T	C	0.07	-0.14	0.01	4.01E-11
ITGB6	rs77041499	4	109443812	G	A	0.01	0.34	0.04	2.19E-07
ITGB6	-	8	60642852	C	T	0.24	0.07	0.01	3.15E-08

ITGB6	rs11414746	9	33150411	A	T	0.42	0.06	0.01	9.12E-08
ITGB6	rs57994353	9	136462535	C	T	0.30	0.08	0.01	3.95E-11
ITGB6	rs56278466	10	17833858	G	T	0.66	0.05	0.01	2.91E-06
ITGB6	rs7310615	12	111427245	G	C	0.52	0.05	0.01	8.48E-07
ITGB6	rs75348693	14	49565212	A	G	0.55	0.06	0.01	3.03E-09
ITGB6	rs7203746	16	4206062	G	A	0.28	0.07	0.01	4.59E-08
ITGB6	rs77542162	17	69085137	G	A	0.02	0.20	0.02	2.64E-08
ITGB7	rs37454	1	43828739	A	C	0.35	0.17	0.01	4.19E-54
ITGB7	rs343808	1	110787385	T	C	0.24	0.06	0.01	8.92E-06
ITGB7	rs6759003	2	62332070	C	T	0.64	0.08	0.01	1.91E-11
ITGB7	rs62168051	2	134325432	G	C	0.16	0.21	0.01	1.18E-47
ITGB7	rs6771051	3	170006440	T	G	0.46	-0.11	0.01	7.59E-23
ITGB7	rs4686445	3	186909819	C	T	0.32	-0.13	0.01	1.60E-29
ITGB7	rs764264929	4	99163742	T	C	0.46	-0.05	0.01	1.12E-05
ITGB7	rs9272209	6	32634200	C	A	0.15	0.10	0.01	7.26E-11
ITGB7	rs6796	7	6462736	C	T	0.28	0.07	0.01	4.69E-09
ITGB7	rs6955795	7	150811537	G	C	0.45	0.05	0.01	7.35E-07
ITGB7	rs9987289	8	9325848	G	A	0.91	-0.08	0.01	8.69E-06
ITGB7	rs7849148	9	33138354	T	A	0.31	0.07	0.01	3.10E-09
ITGB7	rs635634	9	133279427	T	C	0.18	0.11	0.01	2.52E-14
ITGB7	rs78689694	11	126364925	C	G	0.13	-0.15	0.01	7.92E-21
ITGB7	rs12232003	12	53199848	C	T	0.07	0.18	0.01	2.58E-19
ITGB7	rs10774625	12	111472415	G	A	0.50	-0.05	0.01	1.53E-06
ITGB7	rs7161799	15	58478324	T	C	0.08	0.14	0.01	2.57E-12
ITGB7	rs2325756	17	3727327	A	G	0.55	-0.35	0.01	4.12E-222
ITGB7	rs186021206	17	7166093	A	G	0.01	1.81	0.05	8.46E-126
ITGB7	rs2659005	17	81244914	T	C	0.44	0.09	0.01	2.67E-14
ITIH3	rs1260326	2	27508073	C	T	0.61	-0.05	0.01	3.41E-06
ITIH3	rs9881468	3	52794612	T	C	0.34	0.31	0.01	6.96E-164
ITIH3	rs35656703	3	126539442	A	C	0.25	-0.20	0.01	3.72E-62
ITIH3	rs244451	5	129891684	C	T	0.68	-0.08	0.01	1.49E-13
ITIH3	rs35246381	8	18415025	T	C	0.78	0.06	0.01	2.54E-06
ITIH3	rs10808546	8	125483576	T	C	0.45	0.06	0.01	5.45E-08
ITIH3	rs3751198	12	103753429	G	A	0.60	0.05	0.01	1.37E-06
ITM2A	rs9264683	6	31273163	T	A	0.17	-0.10	0.01	2.75E-10
ITM2A	rs709932	14	94382864	T	C	0.15	-0.30	0.01	6.64E-80
ITM2A	rs756845	19	35172347	G	A	0.78	-0.08	0.01	3.82E-08
JAM2	rs3811444	1	247876149	T	C	0.33	0.15	0.01	2.44E-40
JAM2	rs1260326	2	27508073	C	T	0.61	0.05	0.01	9.97E-06
JAM2	rs8176741	9	133256074	A	G	0.06	0.24	0.01	1.82E-26
JAM2	rs764351876	10	63247110	A	C	0.34	0.06	0.01	6.95E-06
JAM2	rs12277151	11	134103559	T	C	0.08	-0.10	0.01	5.81E-07
JAM2	rs3184504	12	111446804	C	T	0.52	0.05	0.01	2.28E-06
JAM2	rs36060036	16	20350628	T	C	0.17	-0.08	0.01	7.21E-08
JAM2	-	21	25542219	C	A	0.05	0.16	0.02	5.71E-10
JCHAIN	rs188468174	1	24965206	T	C	0.01	0.35	0.03	1.35E-12
JCHAIN	-	1	157830356	T	C	0.46	-0.07	0.01	1.87E-09
JCHAIN	-	1	160740283	T	A	0.67	0.05	0.01	1.36E-05
JCHAIN	rs139015452	2	111050019	G	C	0.23	-0.07	0.01	4.99E-07
JCHAIN	rs617119	3	32414275	T	C	0.51	0.06	0.01	2.53E-07
JCHAIN	rs116446171	6	484453	G	C	0.02	0.21	0.03	3.75E-07
JCHAIN	rs1264457	6	30490287	A	G	0.61	-0.08	0.01	2.91E-11
JCHAIN	rs62445869	7	50409913	A	G	0.27	-0.06	0.01	9.59E-06
JCHAIN	rs4872089	8	23218821	G	T	0.24	-0.06	0.01	1.17E-05
JCHAIN	rs2511710	8	102563721	T	G	0.26	-0.09	0.01	5.49E-11
JCHAIN	rs9658720	10	89000912	G	T	0.55	-0.06	0.01	4.08E-07

JCHAIN	rs36051450	11	123474251	C	T	0.20	0.09	0.01	5.26E-09
JCHAIN	rs117972357	14	95577209	A	G	0.00	0.51	0.08	1.70E-05
JCHAIN	rs3803286	14	102780133	G	A	0.67	-0.05	0.01	2.08E-05
JCHAIN	rs885107	16	30661398	C	T	0.26	0.08	0.01	1.55E-10
JCHAIN	-	17	7586439	T	A	0.05	0.14	0.02	2.46E-07
JCHAIN	rs4815598	20	3720778	C	G	0.54	0.05	0.01	2.92E-06
KAZALD1	rs66782572	3	52533601	G	A	0.54	0.06	0.01	1.04E-08
KAZALD1	rs2228243	3	186677324	G	A	0.20	-0.22	0.01	6.74E-64
KAZALD1	rs3731211	9	21986848	A	T	0.72	-0.06	0.01	8.20E-08
KAZALD1	-	9	133274293	A	C	0.18	-0.07	0.01	4.82E-07
KAZALD1	rs11190812	10	101064535	A	G	0.04	-1.69	0.02	0.00E+00
KAZALD1	rs3751198	12	103753429	G	A	0.60	0.05	0.01	3.76E-06
KAZALD1	rs35350651	12	111469627	C	A	0.50	-0.07	0.01	1.35E-10
KDR	rs6727115	2	62389406	C	T	0.19	-0.06	0.01	8.86E-07
KDR	rs10935473	3	98698056	T	G	0.44	-0.16	0.01	5.68E-55
KDR	rs34231037	4	55106779	G	A	0.03	-1.32	0.02	0.00E+00
KDR	rs9267947	6	32243441	G	A	0.48	-0.07	0.01	4.06E-12
KDR	rs932589	6	136762000	T	C	0.40	-0.06	0.01	1.18E-07
KDR	rs7012814	8	9315848	A	G	0.47	-0.05	0.01	5.54E-06
KDR	rs201102287	8	104962043	G	A	0.19	0.06	0.01	2.11E-05
KDR	-	9	133263362	G	A	0.18	-0.48	0.01	6.34E-291
KDR	rs2031610	10	88994730	C	T	0.36	-0.05	0.01	1.20E-06
KDR	rs139130389	11	72139110	A	C	0.07	-0.14	0.01	1.40E-12
KDR	rs17625540	11	95451191	A	G	0.30	-0.05	0.01	2.49E-05
KDR	rs35166255	11	126431861	A	G	0.03	0.25	0.02	1.77E-19
KDR	rs215225	12	481379	G	A	0.43	-0.06	0.01	2.20E-09
KDR	rs146261845	17	7108935	T	C	0.01	0.40	0.04	7.55E-10
KEL	rs28594470	1	25409808	T	A	0.42	-0.05	0.01	4.00E-06
KEL	rs2285170	1	172456389	G	C	0.50	-0.05	0.01	8.87E-06
KEL	rs1427407	2	60490908	G	T	0.85	-0.15	0.01	4.36E-28
KEL	rs35271699	2	218252759	A	G	0.50	-0.04	0.01	2.75E-05
KEL	rs35852078	3	186937047	A	G	0.58	-0.31	0.01	9.66E-203
KEL	rs11752051	6	2379263	A	G	0.49	0.07	0.01	9.74E-12
KEL	rs8176059	7	142954267	A	G	0.01	-1.25	0.03	1.93E-149
KEL	rs34009363	8	97689464	A	T	0.06	-0.09	0.01	1.15E-05
KEL	rs183332045	8	143616297	T	G	0.01	-0.33	0.04	9.70E-08
KEL	rs2519093	9	133266456	T	C	0.18	-0.42	0.01	6.46E-226
KEL	rs17476364	10	69334748	C	T	0.11	0.07	0.01	4.59E-06
KEL	rs201519335	11	16218217	T	C	0.08	0.09	0.01	1.52E-06
KEL	rs4055121	11	126362442	T	C	0.13	-0.10	0.01	4.76E-11
KEL	rs35350651	12	111469627	C	A	0.50	-0.05	0.01	6.06E-07
KEL	rs11630124	15	50723716	A	T	0.02	0.22	0.02	8.95E-11
KEL	rs186021206	17	7166093	A	G	0.01	1.34	0.04	1.28E-86
KEL	rs8887	19	4502189	C	T	0.57	-0.07	0.01	1.51E-10
KEL	rs492602	19	48703160	G	A	0.51	0.06	0.01	2.64E-09
KEL	rs6014993	20	57416581	G	A	0.49	0.05	0.01	1.63E-07
KIFBP	rs1354034	3	56815721	C	T	0.60	0.11	0.01	2.61E-21
KIFBP	rs342293	7	106731773	G	C	0.46	-0.08	0.01	4.40E-13
KIFBP	-	10	63156270	A	T	0.49	0.06	0.01	2.82E-07
KIFBP	rs11502185	11	180258	C	T	0.26	0.07	0.01	3.43E-06
KIFBP	rs58603603	15	63850409	G	A	0.08	-0.10	0.01	8.42E-06
KIR2DL3	rs1050451	6	31272025	G	C	0.65	-0.54	0.01	0.00E+00
KIR2DL3	rs35522829	12	10489694	T	G	0.12	-0.09	0.01	2.49E-09
KIR2DL3	rs7137828	12	111494996	T	C	0.52	-0.07	0.01	7.20E-13
KIR2DL3	rs16985907	19	54735345	C	G	0.04	-0.87	0.02	1.04E-218
KIR3DL1	rs9259983	6	29936626	A	C	0.03	0.53	0.01	8.07E-136

KIR3DL1	rs9916257	17	35470352	T	G	0.44	-0.03	0.00	1.48E-05
KIR3DL1	rs12150998	19	54831584	G	A	0.56	-0.92	0.01	0.00E+00
KIRREL2	-	6	7113135	C	T	0.55	0.06	0.01	5.97E-07
KIRREL2	rs6571015	6	96086602	A	G	0.79	0.15	0.01	1.17E-28
KIRREL2	rs8176746	9	133255935	T	G	0.06	0.54	0.02	2.64E-121
KIRREL2	rs421195	16	283146	T	C	0.11	-0.30	0.01	9.78E-64
KIRREL2	rs8051363	16	75221319	G	A	0.71	-0.11	0.01	3.30E-17
KIRREL2	rs1673931	16	88910069	C	T	0.38	0.07	0.01	4.36E-09
KIRREL2	rs186021206	17	7166093	A	G	0.01	0.50	0.05	8.53E-11
KIRREL2	rs404299	19	35858850	A	G	0.03	-0.46	0.02	1.29E-49
KIRREL2	rs2548458	19	48706068	T	C	0.54	-0.07	0.01	3.49E-09
KIT	rs115340020	1	92300881	A	G	0.03	-0.17	0.02	2.13E-07
KIT	rs2884425	1	234558737	T	C	0.49	-0.07	0.01	3.57E-10
KIT	rs74227709	1	247559286	A	G	0.07	0.16	0.01	4.99E-14
KIT	rs72853300	2	144881199	T	C	0.16	0.07	0.01	1.41E-05
KIT	rs12470153	2	222291192	C	G	0.66	-0.06	0.01	1.87E-06
KIT	rs6795463	3	69796957	T	G	0.43	-0.06	0.01	6.09E-08
KIT	rs6440573	3	148890132	G	C	0.95	0.25	0.02	2.73E-23
KIT	rs9839302	3	198068553	C	T	0.30	-0.08	0.01	2.28E-11
KIT	rs218263	4	54541937	T	C	0.12	-0.08	0.01	3.91E-06
KIT	rs72704195	4	174569481	G	A	0.38	-0.05	0.01	6.51E-06
KIT	rs12203592	6	396321	T	C	0.22	-0.11	0.01	1.40E-15
KIT	rs9266141	6	31356246	G	C	0.24	-0.07	0.01	1.10E-06
KIT	rs12350739	9	16885019	A	G	0.61	-0.14	0.01	9.99E-35
KIT	rs10118718	9	106676694	A	G	0.10	0.09	0.01	1.99E-06
KIT	rs7098111	10	117813667	T	C	0.17	0.11	0.01	8.38E-14
KIT	rs3008336	10	133286029	C	T	0.84	-0.07	0.01	2.50E-06
KIT	rs35056404	11	44579183	A	G	0.06	0.18	0.01	7.55E-15
KIT	rs2155311	11	126421580	A	G	0.48	-0.08	0.01	1.79E-11
KIT	rs12316107	12	12782294	A	C	0.24	0.07	0.01	2.78E-08
KIT	rs138854007	12	57128474	G	C	0.08	-0.12	0.01	3.56E-08
KIT	rs746028214	12	120818823	T	A	0.63	-0.06	0.01	5.65E-07
KIT	rs562342198	16	1242412	G	A	0.04	-0.17	0.02	3.53E-09
KIT	rs1805007	16	89919709	T	C	0.11	-0.15	0.01	6.43E-18
KIT	rs17758695	18	63253621	T	C	0.03	-0.23	0.02	7.00E-13
KIT	rs78744187	19	33263642	T	C	0.08	0.32	0.01	8.89E-62
KIT	rs12609373	19	51795028	C	T	0.22	-0.06	0.01	2.73E-05
KIT	rs2248555	21	41148410	G	A	0.50	0.10	0.01	1.23E-19
KITLG	rs6672758	1	230167766	T	C	0.80	-0.06	0.01	1.82E-05
KITLG	rs705379	7	95324583	A	G	0.48	0.08	0.01	2.52E-12
KITLG	rs983309	8	9320222	G	T	0.88	0.08	0.01	5.90E-06
KITLG	rs112875651	8	125494452	A	G	0.39	0.07	0.01	3.99E-09
KITLG	rs2740488	9	104899461	C	A	0.27	-0.10	0.01	1.59E-15
KITLG	rs635634	9	133279427	T	C	0.18	-0.07	0.01	4.73E-07
KITLG	rs174564	11	61820833	G	A	0.35	0.07	0.01	1.00E-08
KITLG	rs673335	11	75739531	C	T	0.15	0.08	0.01	1.46E-07
KITLG	rs11105121	12	88970809	T	C	0.66	-0.05	0.01	8.39E-06
KITLG	rs150844304	15	43434427	C	A	0.02	-0.20	0.02	3.39E-08
KITLG	rs247617	16	56956804	A	C	0.32	0.13	0.01	1.75E-29
KITLG	rs2292318	16	67951803	T	C	0.12	0.15	0.01	3.61E-19
KITLG	rs34931250	17	68883786	T	C	0.06	-0.11	0.02	5.12E-06
KITLG	rs367070	19	54296648	G	A	0.23	0.11	0.01	1.14E-15
KITLG	rs2868346	20	45919331	T	C	0.76	-0.31	0.01	1.47E-124
KLB	rs114165349	1	26695422	C	G	0.02	-0.14	0.02	1.61E-05
KLB	rs13108218	4	3442204	G	A	0.62	0.05	0.01	2.81E-05
KLB	rs13103023	4	39455997	A	G	0.33	-0.76	0.01	0.00E+00

KLB	rs3135911	5	177086895	A	C	0.29	-0.17	0.01	3.49E-55
KLB	-	7	150805380	A	T	0.11	0.07	0.01	1.91E-05
KLB	rs35254320	12	20885935	T	C	0.15	0.08	0.01	7.00E-09
KLB	rs28929474	14	94378610	T	C	0.02	0.33	0.02	5.62E-22
KLB	rs60134803	17	9697411	G	A	0.24	0.06	0.01	3.44E-06
KLB	rs112001035	17	68827664	A	G	0.06	-0.66	0.01	2.01E-221
KLB	rs897764	19	35068720	C	T	0.94	0.10	0.01	3.08E-07
KLK1	rs3888951	1	15997599	T	C	0.40	0.04	0.01	4.66E-06
KLK1	rs2576570	1	117605053	C	A	0.58	-0.05	0.01	2.18E-09
KLK1	rs423144	1	155199564	T	G	0.43	0.05	0.01	2.03E-08
KLK1	rs35275076	2	121212785	C	T	0.55	-0.05	0.01	4.83E-08
KLK1	rs11889674	2	213162094	A	G	0.39	0.04	0.01	3.20E-07
KLK1	-	3	186932142	C	G	0.62	0.06	0.01	7.58E-13
KLK1	rs2170582	5	44371567	G	A	0.67	-0.04	0.01	1.21E-05
KLK1	rs8176693	9	133262254	T	C	0.06	0.18	0.01	2.06E-23
KLK1	rs3862630	11	126373754	T	C	0.12	0.21	0.01	2.88E-59
KLK1	rs7397745	12	123733479	C	T	0.58	0.05	0.01	1.37E-08
KLK1	rs9529913	13	71770957	T	C	0.60	0.06	0.01	1.43E-11
KLK1	rs55714927	17	7176997	T	C	0.19	0.06	0.01	3.53E-09
KLK1	rs601338	19	48703417	A	G	0.51	0.89	0.01	0.00E+00
KLK1	rs66464836	21	41312157	G	C	0.11	-0.07	0.01	6.96E-07
KLK10	rs4576692	1	119539250	T	C	0.68	-0.06	0.01	2.11E-07
KLK10	rs2596487	6	31357279	T	C	0.22	-0.06	0.01	1.04E-05
KLK10	rs375524395	9	106744749	G	A	0.46	0.05	0.01	5.70E-06
KLK10	rs139130389	11	72139110	A	C	0.07	-0.19	0.01	1.71E-21
KLK10	rs1065853	19	44909976	T	G	0.08	0.10	0.01	4.80E-06
KLK10	rs2569454	19	51019947	C	T	0.58	0.59	0.01	0.00E+00
KLK11	rs34255800	2	241792031	C	G	0.15	-0.23	0.01	1.28E-50
KLK11	-	5	109693185	A	C	0.32	0.06	0.01	5.04E-07
KLK11	rs369705328	10	17806193	C	G	0.13	0.08	0.01	5.95E-06
KLK11	rs139130389	11	72139110	A	C	0.07	-0.45	0.01	4.45E-103
KLK11	rs36060036	16	20350628	T	C	0.17	-0.07	0.01	2.56E-06
KLK11	rs186021206	17	7166093	A	G	0.01	0.48	0.05	1.92E-10
KLK11	rs117268623	19	51024714	T	C	0.03	-1.14	0.02	1.31E-289
KLK12	rs2479016	6	2245111	G	A	0.43	-0.07	0.00	2.10E-22
KLK12	rs11220477	11	126405507	T	C	0.04	-0.13	0.01	2.58E-14
KLK12	rs186021206	17	7166093	A	G	0.01	0.23	0.03	2.65E-06
KLK12	rs79744308	19	5827754	A	G	0.04	0.15	0.02	1.26E-06
KLK12	rs3745540	19	51031874	G	A	0.58	-1.16	0.01	0.00E+00
KLK13	rs12995525	2	106943538	G	T	0.50	0.06	0.01	1.50E-08
KLK13	rs2479016	6	2245111	G	A	0.43	-0.06	0.01	2.76E-07
KLK13	rs529700	10	17843438	C	T	0.51	0.06	0.01	5.55E-06
KLK13	rs226379	12	9115989	C	T	0.36	0.08	0.01	4.81E-12
KLK13	rs5511	14	94567258	T	A	0.22	0.14	0.01	5.90E-27
KLK13	rs55707100	15	43528519	T	C	0.02	-0.17	0.02	6.02E-07
KLK13	rs186021206	17	7166093	A	G	0.01	0.66	0.05	3.16E-19
KLK13	rs75181977	18	48043515	G	A	0.10	0.10	0.01	3.35E-08
KLK13	rs78060698	19	5832762	A	G	0.04	0.18	0.02	3.55E-10
KLK13	rs681343	19	48703205	T	C	0.51	-0.07	0.01	1.81E-10
KLK13	rs3760739	19	51035305	T	G	0.35	-0.45	0.01	0.00E+00
KLK14	rs61816761	1	152313385	A	G	0.02	0.17	0.02	5.63E-06
KLK14	rs58741371	2	118876005	C	T	0.20	0.07	0.01	1.77E-07
KLK14	rs6982210	8	10742484	A	G	0.60	-0.05	0.01	7.57E-07
KLK14	rs60978445	9	108010756	C	T	0.19	-0.07	0.01	2.13E-07
KLK14	rs635634	9	133279427	T	C	0.18	-0.08	0.01	1.32E-08
KLK14	rs11599750	10	100045685	T	C	0.41	-0.06	0.01	2.55E-09

KLK14	rs61729512	12	7485173	A	G	0.12	0.14	0.01	1.64E-17
KLK14	rs1863115	17	46548562	A	C	0.74	-0.07	0.01	2.91E-08
KLK14	rs2569491	19	51081659	A	G	0.30	0.41	0.01	9.63E-258
KLK14	rs12986064	19	54251270	C	T	0.51	-0.07	0.01	1.50E-08
KLK14	rs1295837	20	57012542	G	C	0.51	-0.05	0.01	2.28E-05
KLK4	rs10157145	1	205292835	C	T	0.50	-0.04	0.01	1.45E-06
KLK4	rs28929474	14	94378610	T	C	0.02	-0.44	0.02	1.88E-44
KLK4	rs2664153	19	50911996	A	G	0.64	-0.42	0.01	0.00E+00
KLK6	rs9933330	16	20355488	T	C	0.18	-0.07	0.01	6.51E-06
KLK6	rs268891	19	50968727	C	A	0.65	-0.19	0.01	5.82E-58
KLK6	rs12986064	19	54251270	C	T	0.51	-0.07	0.01	7.71E-09
KLK8	rs61816761	1	152313385	A	G	0.02	0.32	0.02	1.22E-18
KLK8	rs121908120	2	218890289	A	T	0.03	-0.15	0.02	4.02E-06
KLK8	rs186021206	17	7166093	A	G	0.01	0.41	0.05	1.90E-08
KLK8	rs74705037	19	51001552	A	G	0.05	1.05	0.02	0.00E+00
KLK8	rs12986064	19	54251270	C	T	0.51	-0.06	0.01	8.54E-07
KLRB1	rs4149909	1	241860596	G	A	0.03	0.15	0.02	3.45E-06
KLRB1	rs4672505	2	62333197	G	A	0.64	0.08	0.01	3.62E-12
KLRB1	rs2009581	2	111050100	A	G	0.27	-0.07	0.01	2.02E-08
KLRB1	rs4449583	5	1284020	T	C	0.33	0.07	0.01	1.34E-08
KLRB1	rs2549002	5	132493886	A	C	0.66	0.05	0.01	1.26E-05
KLRB1	rs2249932	6	31359384	C	T	0.30	0.08	0.01	2.34E-11
KLRB1	rs900567	8	78647387	T	C	0.75	-0.06	0.01	2.65E-06
KLRB1	-	9	133276163	T	A	0.19	-0.09	0.01	3.20E-09
KLRB1	rs7086174	10	5974436	A	C	0.33	0.06	0.01	3.60E-07
KLRB1	rs563890415	12	9601084	C	T	0.01	-1.12	0.05	6.77E-58
KLRB1	rs3184504	12	111446804	C	T	0.52	-0.13	0.01	9.24E-31
KLRB1	rs12437803	15	50705069	A	G	0.19	-0.18	0.01	9.85E-38
KLRB1	rs11071528	15	60409598	C	G	0.81	0.08	0.01	8.50E-09
KLRB1	rs34785252	19	54814361	A	C	0.29	-0.06	0.01	2.21E-05
KLRD1	rs2009581	2	111050100	A	G	0.27	-0.11	0.01	1.17E-20
KLRD1	rs13063578	3	47046347	A	T	0.40	-0.08	0.01	1.02E-14
KLRD1	rs2706385	5	132496074	G	T	0.66	0.06	0.01	5.37E-07
KLRD1	rs1655895	6	29944740	C	T	0.85	-0.28	0.01	1.32E-79
KLRD1	rs12684650	9	136218808	T	C	0.32	-0.06	0.01	8.07E-07
KLRD1	rs8177655	10	5973156	A	G	0.33	0.05	0.01	8.55E-06
KLRD1	rs2537752	12	10314934	A	T	0.60	-0.42	0.01	0.00E+00
KLRD1	rs3184504	12	111446804	C	T	0.52	-0.10	0.01	1.34E-21
KLRD1	rs34853473	14	65554433	A	T	0.66	-0.05	0.01	8.24E-07
KLRD1	rs58745116	17	35476573	A	G	0.39	-0.06	0.01	8.00E-09
KLRD1	rs3744283	17	64082771	C	G	0.16	-0.07	0.01	2.30E-06
KLRD1	rs875622	19	16356948	G	A	0.77	0.05	0.01	2.81E-05
KLRD1	rs28873836	19	51811402	C	G	0.27	-0.24	0.01	4.16E-93
KLRD1	rs35545130	19	54815642	G	C	0.32	-0.06	0.01	5.91E-07
KRT18	rs79287178	3	172576710	A	G	0.03	-0.15	0.02	1.71E-05
KRT18	rs7678352	4	145893165	T	C	0.16	0.07	0.01	1.56E-06
KRT18	rs7043196	9	114371911	T	C	0.50	-0.05	0.01	2.95E-06
KRT18	rs35696875	10	69222380	A	T	0.70	0.06	0.01	5.03E-06
KRT18	rs10883451	10	100164661	C	T	0.50	-0.05	0.01	1.18E-06
KRT18	rs11601507	11	5679844	A	C	0.07	0.20	0.01	8.43E-21
KRT18	rs28929474	14	94378610	T	C	0.02	0.19	0.02	6.20E-07
KRT18	rs200210321	19	19283081	G	A	0.07	0.09	0.01	9.65E-06
KRT18	rs132642	22	36149089	T	A	0.83	0.07	0.01	7.35E-07
KRT18	rs3747207	22	43928975	A	G	0.22	0.10	0.01	2.55E-14
KRT19	rs7313347	12	52871715	A	G	0.59	-0.05	0.01	5.65E-06
KRT19	rs7405611	17	41535160	A	G	0.62	0.15	0.01	4.33E-36

KRT19	rs601338	19	48703417	A	G	0.51	-0.06	0.01	4.22E-07
KRT5	rs72656914	1	150579726	T	C	0.04	0.28	0.02	5.64E-23
KRT5	rs210135	6	33572915	A	T	0.70	-0.08	0.01	6.10E-10
KRT5	rs73015965	6	160706469	G	A	0.01	-0.71	0.05	1.03E-21
KRT5	rs607860	12	52519245	T	C	0.41	-0.11	0.01	8.87E-21
KRT5	rs7240884	18	59901461	G	A	0.42	0.08	0.01	1.12E-13
KYAT1	rs138429240	1	6628579	T	C	0.00	0.68	0.05	2.70E-17
KYAT1	rs3811444	1	247876149	T	C	0.33	-0.06	0.01	6.79E-06
KYAT1	rs1354034	3	56815721	C	T	0.60	0.06	0.01	2.84E-07
KYAT1	rs35072117	3	101822845	G	T	0.46	0.06	0.01	6.00E-06
KYAT1	rs6993770	8	105569300	T	A	0.29	-0.06	0.01	9.48E-06
KYAT1	-	9	128849806	A	G	0.71	-0.14	0.01	2.32E-28
KYAT1	rs60822569	12	54323724	C	T	0.55	0.06	0.01	5.92E-07
KYNU	rs78244381	2	142924180	A	C	0.05	0.57	0.02	8.02E-108
KYNU	rs1150755	6	32070773	T	C	0.17	-0.07	0.01	2.25E-06
KYNU	rs113609637	9	93140817	T	A	0.14	-0.07	0.01	1.30E-05
KYNU	rs10846740	12	124818736	C	T	0.86	-0.08	0.01	2.36E-07
KYNU	rs200210321	19	19283081	G	A	0.07	0.10	0.01	1.07E-06
KYNU	rs3747207	22	43928975	A	G	0.22	0.10	0.01	2.79E-14
L1CAM	rs3791101	1	43900578	A	G	0.31	0.06	0.01	5.18E-08
L1CAM	rs1260326	2	27508073	C	T	0.61	0.06	0.01	3.20E-07
L1CAM	-	9	133263362	G	A	0.18	-0.49	0.01	8.65E-266
L1CAM	rs56278466	10	17833858	G	T	0.66	0.05	0.01	7.08E-06
L1CAM	rs139130389	11	72139110	A	C	0.07	-0.31	0.01	3.01E-50
L1CAM	rs3781781	11	126410650	C	T	0.32	-0.10	0.01	2.37E-19
L1CAM	rs186021206	17	7166093	A	G	0.01	1.30	0.05	2.46E-70
L1CAM	rs5757680	22	39448788	C	T	0.75	-0.08	0.01	3.25E-11
LACTB2	rs1354034	3	56815721	C	T	0.60	0.07	0.01	3.40E-09
LACTB2	rs191588099	8	70617631	A	G	0.01	-1.11	0.05	2.08E-56
LACTB2	-	10	63156270	A	T	0.48	0.05	0.01	1.12E-05
LACTB2	rs1613662	19	55025227	A	G	0.83	0.07	0.01	2.15E-05
LAG3	rs12740374	1	109274968	T	G	0.22	-0.19	0.01	5.08E-48
LAG3	rs115216147	5	71369699	A	T	0.07	-0.15	0.01	7.39E-12
LAG3	rs35829610	5	132488170	G	C	0.34	-0.07	0.01	1.60E-10
LAG3	rs9264419	6	31262360	A	G	0.11	0.11	0.01	1.43E-09
LAG3	rs11773763	7	50231903	T	C	0.32	-0.05	0.01	1.99E-05
LAG3	-	10	80523403	A	T	0.73	0.06	0.01	1.31E-06
LAG3	rs950802	11	60385111	A	G	0.31	0.05	0.01	6.06E-06
LAG3	rs1056008	12	553672	C	T	0.26	-0.06	0.01	6.25E-06
LAG3	rs3782735	12	6775910	A	G	0.60	0.18	0.01	3.21E-53
LAG3	rs597808	12	111535554	G	A	0.52	-0.11	0.01	5.55E-23
LAG3	rs139974673	15	43735687	C	T	0.02	-0.21	0.02	5.62E-09
LAG3	rs186021206	17	7166093	A	G	0.01	0.53	0.05	5.83E-12
LAG3	rs77542162	17	69085137	G	A	0.02	0.28	0.02	9.88E-15
LAIR1	rs333947	1	109928142	A	G	0.15	0.06	0.01	1.72E-05
LAIR1	rs3184504	12	111446804	C	T	0.52	-0.05	0.01	6.73E-07
LAIR1	rs77924615	16	20381010	A	G	0.20	-0.06	0.01	7.24E-07
LAIR1	rs111949142	19	54358584	A	T	0.18	0.72	0.01	0.00E+00
LAIR2	rs13063578	3	47046347	A	T	0.40	-0.04	0.01	7.60E-07
LAIR2	rs3184504	12	111446804	C	T	0.52	-0.07	0.01	1.71E-15
LAIR2	rs4781977	16	17478817	C	T	0.23	0.07	0.01	2.14E-12
LAIR2	rs35663614	19	16327617	C	G	0.63	-0.06	0.01	2.45E-07
LAIR2	rs73070113	19	54502956	T	A	0.14	-1.06	0.01	0.00E+00
LAMA4	rs61809091	1	183149528	C	T	0.41	0.08	0.01	2.88E-11
LAMA4	rs1421929	5	53057783	G	A	0.49	-0.05	0.01	1.96E-05
LAMA4	rs1917342	6	70640051	G	A	0.30	-0.06	0.01	7.02E-06

LAMA4	rs7765769	6	112138348	C	T	0.44	0.17	0.01	1.46E-51
LAMA4	rs41281051	7	107972762	C	T	0.46	-0.06	0.01	7.71E-07
LAMA4	rs6993770	8	105569300	T	A	0.29	-0.10	0.01	1.36E-16
LAMA4	rs10761731	10	63267850	T	A	0.42	0.09	0.01	1.61E-14
LAMA4	rs7980201	12	7487993	T	C	0.10	0.10	0.01	3.52E-07
LAMA4	rs13339274	16	81841954	T	C	0.14	0.08	0.01	3.93E-07
LAMA4	rs143040759	17	35766707	A	G	0.02	0.62	0.03	2.94E-57
LAMA4	rs892090	19	55027704	G	T	0.83	0.13	0.01	1.34E-17
LAMA4	rs4813351	20	19288289	T	C	0.37	0.05	0.01	1.68E-05
LAMP2	rs9427104	1	154616756	T	C	0.48	-0.06	0.01	1.52E-06
LAMP2	rs41274050	10	50814012	T	C	0.01	0.27	0.04	2.51E-06
LAMP3	rs11921884	3	183110457	T	G	0.65	0.23	0.01	2.01E-93
LAMP3	rs13107325	4	102267552	T	C	0.08	0.10	0.01	4.76E-06
LAMP3	rs9263519	6	31099407	A	G	0.33	-0.06	0.01	1.58E-07
LAMP3	rs13281991	8	119911334	C	T	0.56	0.05	0.01	1.46E-05
LAMP3	rs507666	9	133273983	A	G	0.18	-0.07	0.01	2.62E-07
LAMP3	rs35705950	11	1219991	T	G	0.11	0.09	0.01	9.10E-07
LAMP3	rs7962469	12	51954475	G	A	0.65	0.05	0.01	1.34E-05
LAMP3	-	12	101880278	C	T	0.38	0.07	0.01	1.39E-10
LAMP3	rs3184504	12	111446804	C	T	0.52	-0.06	0.01	3.25E-08
LAMP3	rs117169628	16	89196249	A	G	0.14	-0.09	0.01	6.45E-09
LAMP3	rs186021206	17	7166093	A	G	0.01	0.44	0.05	3.34E-09
LAMP3	rs892225	19	1152657	G	A	0.38	-0.07	0.01	2.85E-09
LAT	rs13412535	2	224010157	A	G	0.23	-0.06	0.01	1.45E-05
LAT	rs3804749	3	123114156	T	C	0.59	-0.06	0.01	1.24E-06
LAT	rs17622656	5	132485305	A	G	0.39	-0.05	0.01	6.11E-06
LAT	rs6993770	8	105569300	T	A	0.29	-0.09	0.01	1.03E-13
LAT	rs7080386	10	63288546	A	C	0.41	0.07	0.01	2.93E-08
LAT	rs892090	19	55027704	G	T	0.83	0.09	0.01	1.94E-09
LAT2	rs1354034	3	56815721	C	T	0.60	0.06	0.01	7.61E-08
LAYN	rs61747728	1	179557079	T	C	0.04	0.12	0.02	1.08E-05
LAYN	rs539726870	6	32638754	G	A	0.12	0.09	0.01	3.98E-07
LAYN	rs8176719	9	133257521	C	T	0.34	0.07	0.01	3.79E-10
LAYN	rs542275	11	111560889	T	C	0.62	0.39	0.01	8.47E-272
LAYN	rs7203642	16	20355808	G	A	0.19	-0.07	0.01	8.11E-08
LBP	rs34931250	17	68883786	T	C	0.06	0.11	0.01	4.01E-07
LBP	rs573329980	20	32563114	A	G	0.19	0.09	0.01	1.24E-08
LBP	rs2232613	20	38369011	T	C	0.08	-1.18	0.01	0.00E+00
LBR	rs1354034	3	56815721	C	T	0.60	0.16	0.01	3.30E-47
LBR	rs342293	7	106731773	G	C	0.46	-0.06	0.01	9.86E-08
LBR	rs10733789	10	63188924	C	T	0.31	0.06	0.01	5.40E-07
LBR	rs7080536	10	113588287	A	G	0.04	-0.22	0.02	2.42E-15
LBR	rs11604127	11	196944	T	C	0.23	0.09	0.01	1.22E-10
LBR	rs10876550	12	54318524	A	G	0.56	0.05	0.01	3.08E-06
LCN2	rs3917932	1	36478315	G	C	0.58	-0.07	0.01	1.31E-10
LCN2	rs3014874	1	153365467	A	G	0.26	0.07	0.01	7.07E-09
LCN2	rs113542380	2	43237679	A	G	0.07	0.10	0.01	5.75E-06
LCN2	rs2305637	3	47004356	T	C	0.18	0.08	0.01	1.47E-07
LCN2	rs218264	4	54542708	T	A	0.25	0.08	0.01	1.38E-08
LCN2	rs5869700	5	95917754	T	C	0.33	0.08	0.01	1.56E-10
LCN2	rs12520928	5	132970199	A	C	0.16	0.09	0.01	6.87E-10
LCN2	rs2158799	7	28237488	G	C	0.61	0.06	0.01	5.95E-07
LCN2	rs1870028	7	50218717	T	C	0.07	0.16	0.01	8.12E-13
LCN2	rs7846314	8	60738272	T	A	0.19	0.13	0.01	1.08E-18
LCN2	rs4407843	8	129597122	C	A	0.57	-0.06	0.01	2.08E-08
LCN2	rs542243688	9	128143271	A	C	0.03	-0.22	0.02	8.32E-09

LCN2	rs181969679	12	53617945	G	A	0.01	0.34	0.04	4.61E-08
LCN2	rs4997081	16	20353912	C	G	0.19	-0.06	0.01	2.41E-05
LCN2	rs2241244	17	39997301	C	G	0.54	-0.12	0.01	1.74E-27
LCN2	rs7246563	19	12411814	C	T	0.22	-0.07	0.01	2.57E-06
LCN2	rs78030362	19	18464383	G	A	0.07	-0.15	0.01	4.04E-11
LCN2	rs243591	21	17783489	C	A	0.48	-0.06	0.01	7.09E-07
LDLR	rs765974791	1	62541132	T	C	0.64	0.10	0.01	2.37E-17
LDLR	rs1260326	2	27508073	C	T	0.61	-0.08	0.01	2.18E-12
LDLR	rs4704834	5	157016055	G	A	0.65	0.08	0.01	1.16E-12
LDLR	rs451637	6	32027005	T	C	0.31	-0.06	0.01	1.86E-07
LDLR	rs3812316	7	73606007	G	C	0.13	-0.09	0.01	1.05E-08
LDLR	rs13702	8	19966981	C	T	0.29	-0.09	0.01	3.61E-14
LDLR	rs2737246	8	115647351	C	G	0.28	-0.07	0.01	8.82E-09
LDLR	rs28601761	8	125487789	G	C	0.42	-0.09	0.01	5.19E-17
LDLR	rs7896832	10	80497814	C	T	0.79	0.06	0.01	1.85E-05
LDLR	rs2068888	10	93079885	A	G	0.45	-0.05	0.01	1.03E-05
LDLR	rs174533	11	61781553	A	G	0.35	0.07	0.01	2.44E-09
LDLR	rs964184	11	116778201	C	G	0.87	-0.20	0.01	8.00E-36
LDLR	rs261342	15	58438954	C	G	0.78	-0.06	0.01	1.41E-05
LDLR	rs247617	16	56956804	A	C	0.32	-0.09	0.01	8.03E-16
LDLR	rs77542162	17	69085137	G	A	0.02	0.21	0.02	2.13E-08
LDLR	rs116843064	19	8364439	A	G	0.02	-0.21	0.03	3.49E-07
LDLR	rs8107974	19	19277691	T	A	0.08	-0.12	0.01	3.29E-08
LDLR	rs1065853	19	44909976	T	G	0.08	0.43	0.01	5.11E-104
LEFTY2	rs748667196	1	225887710	G	C	0.37	-0.71	0.01	0.00E+00
LEFTY2	rs10027275	4	148060345	C	G	0.74	0.05	0.01	4.39E-07
LEFTY2	rs635634	9	133279427	T	C	0.18	-0.08	0.01	6.02E-13
LEFTY2	rs113878851	16	20353760	T	C	0.17	-0.06	0.01	7.01E-06
LEFTY2	rs7412	19	44908822	T	C	0.08	0.11	0.01	7.73E-12
LEFTY2	rs738408	22	43928850	T	C	0.22	0.05	0.01	9.27E-07
LEP	rs56094641	16	53772541	G	A	0.40	0.04	0.01	1.63E-05
LEPR	rs10399687	1	65608299	T	C	0.17	-0.27	0.01	1.08E-76
LEPR	rs1260326	2	27508073	C	T	0.61	-0.15	0.01	3.52E-39
LEPR	rs687339	3	136213517	T	C	0.77	-0.06	0.01	2.17E-05
LEPR	rs111269058	7	73561372	T	C	0.12	-0.08	0.01	3.08E-06
LEPR	rs2298475	11	126408308	C	T	0.07	-0.09	0.01	1.38E-05
LEPR	rs2464190	12	120977587	C	T	0.41	0.06	0.01	7.05E-08
LEPR	rs28929474	14	94378610	T	C	0.02	0.30	0.03	4.28E-14
LEPR	rs186021206	17	7166093	A	G	0.01	0.41	0.05	8.87E-08
LEPR	rs1801689	17	66214462	C	A	0.03	0.26	0.02	4.29E-15
LGALS1	rs10418046	19	53824615	G	T	0.21	0.07	0.01	1.02E-06
LGALS1	rs4820294	22	37675036	A	G	0.33	-0.28	0.01	1.41E-123
LGALS3	rs78542815	7	150609714	G	A	0.22	0.11	0.01	4.99E-22
LGALS3	rs6472539	8	70717575	C	G	0.51	-0.05	0.01	7.50E-07
LGALS3	rs751544923	11	126353222	A	G	0.06	0.17	0.01	2.68E-15
LGALS3	rs765911263	12	7448239	A	G	0.05	0.10	0.01	9.61E-06
LGALS3	rs2393791	12	120986153	T	C	0.62	-0.08	0.01	4.36E-15
LGALS3	rs3832943	14	55140083	T	A	0.42	-0.53	0.01	0.00E+00
LGALS3	rs17855739	19	5831829	T	C	0.04	0.18	0.02	1.32E-13
LGALS3	rs516246	19	48702915	T	C	0.51	0.08	0.01	4.50E-15
LGALS3	rs12986064	19	54251270	C	T	0.51	-0.06	0.01	2.37E-10
LGALS4	rs113323158	8	71005067	C	A	0.51	-0.05	0.01	5.19E-06
LGALS4	rs505922	9	133273813	C	T	0.32	-0.52	0.01	0.00E+00
LGALS4	rs17655730	11	270715	C	T	0.25	0.06	0.01	7.45E-06
LGALS4	rs140695578	19	38774589	T	A	0.03	0.27	0.02	1.06E-20
LGALS7_LGALS7B	rs61816761	1	152313385	A	G	0.02	0.32	0.02	5.60E-18

LGALS7_LGALS7B	rs833270	3	181802378	C	T	0.62	0.05	0.01	1.10E-05
LGALS7_LGALS7B	rs505922	9	133273813	C	T	0.32	0.31	0.01	1.94E-163
LGALS7_LGALS7B	rs17658212	15	40853721	T	C	0.06	-0.11	0.01	6.07E-06
LGALS7_LGALS7B	rs9939286	16	31150982	G	T	0.63	-0.06	0.01	5.89E-07
LGALS7_LGALS7B	rs6508818	19	38786514	G	A	0.58	0.23	0.01	2.68E-96
LGALS7_LGALS7B	rs601338	19	48703417	A	G	0.51	0.05	0.01	2.08E-05
LGALS8	rs1475133	1	236553386	A	G	0.76	0.30	0.01	5.85E-125
LGALS8	rs1354034	3	56815721	C	T	0.60	0.08	0.01	1.28E-11
LGALS8	rs342293	7	106731773	G	C	0.46	-0.08	0.01	7.48E-14
LGALS8	rs55646585	8	143925453	T	C	0.41	0.06	0.01	1.16E-08
LGALS8	rs2519093	9	133266456	T	C	0.18	-0.48	0.01	1.76E-248
LGALS8	-	10	63156270	A	T	0.49	0.06	0.01	5.25E-07
LGALS8	rs60822569	12	54323724	C	T	0.55	0.05	0.01	1.51E-06
LGALS8	rs8037137	15	90963407	C	T	0.13	-0.08	0.01	5.22E-06
LGALS9	rs3184504	12	111446804	C	T	0.52	-0.08	0.01	5.95E-14
LGALS9	rs12936577	17	22648851	T	G	0.02	-0.30	0.03	9.95E-13
LGALS9	rs79526289	17	27644953	G	A	0.25	-0.51	0.01	0.00E+00
LGALS9	rs601338	19	48703417	A	G	0.51	0.08	0.01	4.73E-15
LGMN	rs823078	1	205807330	C	T	0.95	-0.11	0.02	1.41E-05
LGMN	rs35340377	1	247874908	A	G	0.28	-0.07	0.01	2.33E-08
LGMN	rs35677470	3	58197909	A	G	0.08	0.13	0.01	8.61E-11
LGMN	rs6848819	4	153530526	C	A	0.03	0.14	0.02	7.77E-06
LGMN	rs1655901	6	29949027	C	T	0.56	-0.05	0.01	2.43E-05
LGMN	rs1036951	6	160039050	G	C	0.33	0.06	0.01	4.21E-07
LGMN	rs17154130	7	80600168	C	T	0.06	0.12	0.01	5.73E-07
LGMN	rs4734879	8	105570896	G	A	0.27	-0.12	0.01	1.95E-22
LGMN	rs2737246	8	115647351	C	G	0.28	-0.05	0.01	1.44E-05
LGMN	rs6986384	8	123819941	A	G	0.08	0.10	0.01	3.17E-06
LGMN	rs7870723	9	97936960	C	T	0.36	0.05	0.01	9.03E-06
LGMN	rs600038	9	133276354	C	T	0.21	-0.07	0.01	6.98E-08
LGMN	rs2250645	10	89249116	T	C	0.30	0.07	0.01	4.66E-09
LGMN	rs12767683	10	102556824	A	C	0.32	0.06	0.01	1.66E-07
LGMN	rs149871778	12	8946399	C	G	0.10	0.37	0.01	4.10E-95
LGMN	rs117566084	12	101796743	T	C	0.01	0.46	0.04	3.12E-16
LGMN	rs148659834	14	92709697	A	G	0.01	-1.04	0.03	5.51E-93
LGMN	rs3026096	17	5372623	A	G	0.49	-0.05	0.01	1.16E-06
LGMN	rs55976836	19	48271426	A	G	0.23	-0.06	0.01	1.89E-05
LGMN	rs1654425	19	55027612	C	T	0.83	0.10	0.01	2.35E-12
LHB	rs72481571	14	105904017	A	G	0.11	0.16	0.01	2.02E-15
LHB	rs3795048	19	49017585	T	A	0.06	-0.12	0.02	5.33E-07
LHPP	-	10	124470382	G	C	0.18	0.46	0.01	2.05E-230
LIFR	rs61804164	1	161653235	C	G	0.12	0.11	0.01	4.00E-11
LIFR	rs1260326	2	27508073	C	T	0.61	0.05	0.01	1.37E-05
LIFR	rs34579268	3	58424827	T	C	0.37	0.06	0.01	2.63E-07
LIFR	rs34211178	3	98664718	A	G	0.44	-0.06	0.01	5.44E-07
LIFR	rs11923060	3	186871601	T	C	0.16	0.08	0.01	1.73E-07
LIFR	rs3729734	5	38527206	A	G	0.05	-0.36	0.02	5.64E-42
LIFR	rs35464067	6	32591711	C	T	0.15	0.08	0.01	3.39E-06
LIFR	rs4841132	8	9326086	G	A	0.91	-0.08	0.01	1.81E-05
LIFR	rs2519093	9	133266456	T	C	0.18	-0.42	0.01	1.17E-184
LIFR	rs11596680	10	19919554	G	A	0.33	0.06	0.01	1.13E-06
LIFR	rs7952602	11	126363774	C	G	0.13	-0.10	0.01	1.65E-09
LIFR	rs186021206	17	7166093	A	G	0.01	1.02	0.05	1.02E-41
LIFR	rs2252576	21	41243366	T	C	0.24	0.06	0.01	5.77E-06
LILRA2	rs2070901	1	161215268	T	G	0.27	0.07	0.01	5.65E-09
LILRA2	rs1257169	2	134206291	A	C	0.50	-0.08	0.01	3.22E-15

LILRA2	rs140473879	4	154557913	T	C	0.00	-0.99	0.05	9.22E-34
LILRA2	rs539981616	6	32651394	G	C	0.29	-0.05	0.01	1.54E-05
LILRA2	rs2723509	7	17777413	A	G	0.41	-0.05	0.01	6.92E-06
LILRA2	rs635634	9	133279427	T	C	0.18	0.06	0.01	6.48E-06
LILRA2	rs4055121	11	126362442	T	C	0.14	-0.08	0.01	1.56E-08
LILRA2	rs3184504	12	111446804	C	T	0.52	-0.07	0.01	2.87E-11
LILRA2	rs76428106	13	28029870	C	T	0.01	0.22	0.03	1.30E-06
LILRA2	rs186021206	17	7166093	A	G	0.01	0.89	0.04	1.49E-40
LILRA2	rs35866622	19	48714803	T	C	0.50	0.05	0.01	5.18E-06
LILRA2	rs10407899	19	54576244	G	A	0.09	-1.00	0.01	0.00E+00
LILRA5	rs748544288	1	150635844	C	G	0.23	-0.07	0.01	4.03E-07
LILRA5	rs4233366	1	161189357	T	C	0.27	0.12	0.01	1.63E-23
LILRA5	rs2019727	1	196705584	A	T	0.19	0.21	0.01	9.03E-53
LILRA5	rs597697	6	10538712	G	A	0.58	0.05	0.01	3.94E-06
LILRA5	rs9264942	6	31306603	C	T	0.35	0.05	0.01	1.60E-05
LILRA5	rs10980802	9	111156576	G	A	0.48	-0.07	0.01	2.73E-11
LILRA5	rs1892548	13	40428504	C	T	0.66	-0.06	0.01	6.03E-08
LILRA5	rs8033923	15	79965405	G	A	0.21	-0.07	0.01	5.71E-07
LILRA5	rs186021206	17	7166093	A	G	0.01	0.40	0.05	1.01E-07
LILRA5	rs34464703	19	54302450	G	T	0.33	-0.36	0.01	3.23E-205
LILRA5	rs913672	20	50290430	G	T	0.34	0.05	0.01	2.60E-05
LILRB1	rs1257169	2	134206291	A	C	0.50	-0.07	0.01	1.67E-13
LILRB1	-	2	202612713	T	A	0.37	0.05	0.01	1.65E-06
LILRB1	rs27295	5	97022983	T	C	0.55	-0.05	0.01	2.83E-06
LILRB1	rs9266271	6	31358305	C	T	0.35	0.20	0.01	2.19E-87
LILRB1	rs2031902	9	33117526	T	C	0.59	-0.04	0.01	6.51E-06
LILRB1	rs115478735	9	133274295	T	A	0.18	0.08	0.01	1.90E-11
LILRB1	rs1072160	10	69434250	A	G	0.44	0.08	0.01	2.69E-15
LILRB1	rs75974417	16	57029582	T	C	0.21	0.06	0.01	7.42E-08
LILRB1	rs186021206	17	7166093	A	G	0.01	0.95	0.04	9.33E-49
LILRB1	rs377056194	19	10597180	C	T	0.09	0.10	0.01	3.35E-07
LILRB1	rs367070	19	54296648	G	A	0.23	-0.67	0.01	0.00E+00
LILRB2	rs10801559	1	196735074	A	G	0.40	-0.08	0.01	3.53E-21
LILRB2	rs9267084	6	31441828	A	G	0.15	0.14	0.01	4.25E-34
LILRB2	rs2031902	9	33117526	T	C	0.59	-0.04	0.01	1.39E-05
LILRB2	rs1072160	10	69434250	A	G	0.44	0.06	0.01	4.13E-12
LILRB2	rs3184504	12	111446804	C	T	0.52	-0.05	0.01	9.47E-10
LILRB2	rs72744945	15	56638995	C	A	0.10	0.06	0.01	2.04E-05
LILRB2	rs186021206	17	7166093	A	G	0.01	0.58	0.04	5.65E-26
LILRB2	rs77542162	17	69085137	G	A	0.02	0.13	0.02	5.33E-06
LILRB2	rs383925	19	54279666	T	C	0.21	-1.15	0.01	0.00E+00
LILRB2	rs3747207	22	43928975	A	G	0.22	0.04	0.01	9.03E-06
LILRB4	rs9270270	6	32589431	C	T	0.84	-0.73	0.01	0.00E+00
LILRB4	rs3184504	12	111446804	C	T	0.52	-0.06	0.01	1.17E-08
LILRB4	rs1065853	19	44909976	T	G	0.08	0.09	0.01	1.91E-06
LILRB4	rs7247412	19	51810615	C	T	0.27	-0.07	0.01	4.31E-10
LILRB4	rs417477	19	54660452	G	A	0.12	0.38	0.01	1.67E-130
LILRB5	rs2082206	10	69444867	A	G	0.44	0.05	0.00	3.62E-15
LILRB5	rs186021206	17	7166093	A	G	0.01	0.35	0.03	1.17E-17
LILRB5	rs12975366	19	54255498	C	T	0.40	-1.18	0.01	0.00E+00
LPCAT2	rs79575505	16	55556698	A	C	0.05	-0.25	0.02	8.59E-21
LPL	rs1260326	2	27508073	C	T	0.61	0.05	0.01	3.21E-08
LPL	rs10649609	2	164696196	T	A	0.37	0.05	0.01	1.24E-06
LPL	rs1515104	2	226227689	A	T	0.65	-0.05	0.01	3.77E-07
LPL	rs72959041	6	127133748	A	G	0.05	-0.10	0.01	1.69E-05
LPL	rs149522	7	130765083	T	C	0.40	-0.05	0.01	6.12E-07

LPL	rs1801177	8	19948197	A	G	0.02	-1.46	0.02	0.00E+00
LPL	rs2980888	8	125495066	C	T	0.70	0.05	0.01	2.29E-05
LPL	rs12600110	16	912154	C	T	0.38	0.05	0.01	5.97E-06
LPL	rs116843064	19	8364439	A	G	0.02	0.23	0.02	1.03E-10
LPO	rs61804164	1	161653235	C	G	0.12	0.08	0.01	3.73E-06
LPO	rs6703244	1	202218392	G	A	0.41	0.08	0.01	1.81E-11
LPO	rs908702	1	206932296	T	C	0.45	0.05	0.01	5.66E-06
LPO	rs9274442	6	32665454	C	G	0.28	-0.08	0.01	2.68E-08
LPO	rs62418808	6	119197753	A	G	0.33	-0.17	0.01	1.61E-49
LPO	rs7791463	7	98189735	A	G	0.54	-0.08	0.01	1.62E-11
LPO	rs56278466	10	17833858	G	T	0.66	0.14	0.01	1.33E-31
LPO	rs1549791	11	34846421	G	A	0.43	-0.05	0.01	2.71E-05
LPO	rs1427751	12	11307297	T	C	0.83	-0.11	0.01	3.36E-13
LPO	rs7219860	17	58243910	A	G	0.22	-0.31	0.01	1.56E-115
LPO	rs12019136	19	5835666	A	G	0.04	0.62	0.02	2.35E-105
LPO	rs34262244	19	17795098	A	G	0.29	-0.07	0.01	2.01E-07
LPO	rs601338	19	48703417	A	G	0.51	0.15	0.01	1.83E-41
LPO	rs61739245	20	33289907	T	A	0.02	-0.25	0.03	1.94E-09
LRIG1	rs10127775	1	230160042	T	A	0.61	0.09	0.01	9.21E-21
LRIG1	rs10182990	2	241464525	G	T	0.43	-0.05	0.01	5.62E-06
LRIG1	rs2306272	3	66384219	C	T	0.29	-0.86	0.01	0.00E+00
LRIG1	-	5	180805265	G	A	0.18	0.06	0.01	7.05E-06
LRIG1	rs9372538	6	119470047	T	C	0.28	0.08	0.01	8.75E-13
LRIG1	rs56278466	10	17833858	G	T	0.66	0.07	0.01	6.55E-14
LRIG1	rs11045856	12	21197755	G	T	0.24	0.08	0.01	1.30E-13
LRIG1	rs10083143	12	24045634	C	A	0.04	0.14	0.02	1.19E-07
LRIG1	rs7979473	12	120982457	G	A	0.62	-0.04	0.01	2.35E-05
LRIG1	rs28929474	14	94378610	T	C	0.02	0.32	0.02	9.15E-23
LRIG1	rs339969	15	60591082	A	C	0.62	0.05	0.01	1.54E-06
LRIG1	rs17616063	16	51402971	G	A	0.07	-0.08	0.01	2.52E-05
LRIG1	rs774552666	19	35062896	A	G	0.93	0.09	0.01	1.91E-06
LRP1	rs1010554	3	52508903	C	T	0.47	-0.05	0.01	7.58E-06
LRP1	rs140556958	4	3294944	A	G	0.00	1.86	0.13	3.96E-22
LRP1	rs59674561	6	31435246	T	G	0.03	1.84	0.02	0.00E+00
LRP1	rs328	8	19962213	G	C	0.10	-0.22	0.01	2.70E-42
LRP1	rs10820966	9	92293147	T	A	0.33	-0.11	0.01	3.09E-27
LRP1	rs1935	10	63168063	G	C	0.47	0.05	0.01	2.03E-07
LRP1	rs2229498	10	69097096	A	G	0.84	-0.12	0.01	1.46E-18
LRP1	rs71279465	11	126354409	A	T	0.30	0.06	0.01	1.55E-07
LRP1	rs11846959	14	94379979	A	G	0.25	0.12	0.01	1.05E-25
LRP1	rs217181	16	72080103	T	C	0.20	-0.05	0.01	2.28E-05
LRP1	rs200489612	17	7203059	A	G	0.01	0.61	0.05	2.30E-16
LRP1	rs2277998	19	7766742	A	G	0.30	-0.05	0.01	1.01E-05
LRP1	rs112467088	21	41507476	T	A	0.31	0.05	0.01	6.39E-07
LRP11	rs61747728	1	179557079	T	C	0.04	0.18	0.02	5.11E-11
LRP11	rs7684253	4	56861145	T	C	0.55	-0.05	0.01	2.12E-05
LRP11	rs2342858	6	149805967	T	C	0.36	0.60	0.01	0.00E+00
LRP11	rs9987289	8	9325848	G	A	0.91	-0.08	0.01	1.50E-05
LRP11	rs777834943	16	20355409	T	C	0.18	-0.07	0.01	3.38E-07
LRP11	rs186021206	17	7166093	A	G	0.01	0.43	0.05	1.75E-09
LRP11	rs1801689	17	66214462	C	A	0.03	0.18	0.02	1.16E-09
LRPAP1	rs143486729	4	3523929	A	G	0.03	-0.54	0.02	9.21E-92
LRPAP1	rs13107325	4	102267552	T	C	0.08	0.10	0.01	1.79E-07
LRPAP1	rs115881826	6	31357496	G	A	0.04	1.78	0.02	0.00E+00
LRPAP1	rs7923609	10	63374062	G	A	0.47	0.05	0.01	5.66E-08
LRPAP1	rs10895986	11	65134531	A	C	0.18	-0.07	0.01	4.78E-08

LRPAP1	rs117654599	12	123743753	A	T	0.01	0.31	0.03	3.21E-12
LRPAP1	rs11846959	14	94379979	A	G	0.25	0.07	0.01	3.76E-11
LRRC25	rs1260326	2	27508073	C	T	0.61	0.05	0.01	2.51E-05
LRRC25	rs4672491	2	62284452	G	T	0.52	-0.07	0.01	5.42E-11
LRRC25	rs13388024	2	218233050	G	A	0.50	-0.05	0.01	6.64E-06
LRRC25	rs2470750	3	98971748	T	A	0.39	-0.21	0.01	8.12E-83
LRRC25	rs13107325	4	102267552	T	C	0.08	-0.14	0.01	2.46E-12
LRRC25	rs509227	6	10535358	C	A	0.52	0.29	0.01	3.54E-165
LRRC25	rs2858331	6	32713500	G	A	0.37	-0.08	0.01	4.16E-13
LRRC25	rs12667978	7	129137407	T	C	0.57	-0.14	0.01	1.59E-40
LRRC25	rs199922514	8	9325592	G	A	0.91	-0.09	0.01	9.69E-07
LRRC25	-	9	133263362	G	A	0.18	0.06	0.01	2.13E-05
LRRC25	-	10	80523403	A	T	0.73	0.06	0.01	1.64E-06
LRRC25	rs950802	11	60385111	A	G	0.31	0.17	0.01	3.99E-51
LRRC25	rs4055121	11	126362442	T	C	0.13	-0.08	0.01	1.30E-07
LRRC25	rs186021206	17	7166093	A	G	0.01	1.05	0.05	2.09E-47
LRRC25	rs2659005	17	81244914	T	C	0.44	0.08	0.01	8.18E-13
LRRC25	rs754032589	19	18402007	T	A	0.41	-0.34	0.01	9.21E-210
LRRC25	rs4893	19	48330351	G	A	0.04	-0.45	0.02	7.31E-60
LRRN1	rs9839475	3	3766094	A	G	0.35	0.81	0.01	0.00E+00
LRRN1	rs11443126	5	53980126	A	C	0.69	0.05	0.01	1.70E-05
LRRN1	rs3941888	6	109251208	A	G	0.31	-0.05	0.01	7.74E-06
LRRN1	rs1405212	6	117169501	C	T	0.63	-0.06	0.01	1.94E-10
LRRN1	rs11414746	9	33150411	A	T	0.42	0.05	0.01	3.23E-06
LRRN1	rs55861089	11	1762527	G	A	0.11	-0.11	0.01	4.59E-12
LRRN1	rs594144	11	75015783	T	C	0.47	0.05	0.01	1.14E-06
LRRN1	rs6496452	15	84829414	T	A	0.45	0.04	0.01	1.08E-05
LSP1	rs907612	11	1852991	T	C	0.38	0.15	0.01	3.55E-38
LSP1	rs12941811	17	40003082	C	T	0.58	-0.05	0.01	2.38E-06
LTA	rs2009581	2	111050100	A	G	0.27	-0.07	0.01	3.65E-11
LTA	rs58106596	2	231714669	A	G	0.22	0.06	0.01	1.39E-07
LTA	rs2229092	6	31572980	C	A	0.06	-1.49	0.01	0.00E+00
LTA	rs764024035	12	6395606	G	T	0.17	0.18	0.01	1.39E-46
LTA	rs3184504	12	111446804	C	T	0.52	-0.15	0.01	1.02E-55
LTA	rs186021206	17	7166093	A	G	0.01	0.32	0.04	9.68E-07
LTA	rs34557412	17	16948873	G	A	0.01	0.45	0.04	3.60E-15
LTA	rs8071789	17	39850080	T	C	0.49	-0.04	0.01	1.86E-05
LTA	rs344560	19	6665009	C	T	0.95	0.16	0.01	6.04E-15
LTA	rs6073290	20	44030119	G	A	0.70	0.05	0.01	8.87E-06
LTA	rs738409	22	43928847	G	C	0.22	0.06	0.01	1.17E-06
LTA4H	rs2540486	12	96037229	G	C	0.35	0.10	0.01	8.20E-18
LTBP2	rs4920605	1	16988930	A	G	0.57	0.07	0.01	6.73E-14
LTBP2	rs333947	1	109928142	A	G	0.15	-0.06	0.01	4.75E-06
LTBP2	rs61801010	1	161572445	G	A	0.11	0.08	0.01	3.67E-07
LTBP2	rs6973480	7	17938506	T	C	0.52	0.05	0.01	3.07E-07
LTBP2	rs748386792	10	63542085	T	G	0.46	0.05	0.01	5.64E-06
LTBP2	rs3751198	12	103753429	G	A	0.60	0.08	0.01	2.69E-17
LTBP2	rs888414	14	74638202	A	G	0.38	-0.10	0.01	9.59E-26
LTBP2	rs1036477	15	48622729	G	A	0.10	-0.08	0.01	1.53E-06
LTBP2	rs200489612	17	7203059	A	G	0.01	0.65	0.05	4.64E-20
LTBP3	rs746777507	2	53642581	T	A	0.20	0.06	0.01	1.67E-05
LTBP3	rs376096585	2	55867443	T	C	0.22	0.06	0.01	1.93E-06
LTBP3	rs6718159	2	203198007	A	T	0.56	-0.05	0.01	4.86E-06
LTBP3	rs140336797	5	96970610	T	A	0.43	0.06	0.01	6.22E-08
LTBP3	rs4099470	11	65552515	T	C	0.05	0.90	0.02	3.31E-295
LTBP3	rs28929474	14	94378610	T	C	0.02	0.26	0.02	2.46E-12

LTBP3	rs77303550	16	72045758	T	C	0.20	-0.07	0.01	4.77E-07
LTBP3	rs1801689	17	66214462	C	A	0.03	0.72	0.02	2.67E-121
LTBP3	rs73045269	19	41319286	T	C	0.17	0.15	0.01	6.48E-26
LTBR	rs61747728	1	179557079	T	C	0.04	0.20	0.02	7.99E-13
LTBR	rs1260326	2	27508073	C	T	0.61	0.07	0.01	1.25E-09
LTBR	rs75166367	2	162107791	A	G	0.06	-0.10	0.01	1.79E-05
LTBR	rs12613853	2	178132920	G	A	0.38	0.05	0.01	1.09E-05
LTBR	rs1434055	2	203222136	A	G	0.56	-0.06	0.01	2.44E-08
LTBR	rs27295	5	97022983	T	C	0.55	-0.05	0.01	1.18E-05
LTBR	-	6	31434889	T	C	0.31	0.07	0.01	1.92E-09
LTBR	rs10849449	12	6389496	G	A	0.65	-0.31	0.01	9.39E-154
LTBR	rs966541	12	29338595	G	A	0.28	0.08	0.01	2.70E-09
LTBR	rs28929474	14	94378610	T	C	0.02	0.95	0.03	1.57E-135
LTBR	rs77924615	16	20381010	A	G	0.20	-0.08	0.01	9.46E-08
LTBR	rs1801689	17	66214462	C	A	0.03	0.15	0.02	1.89E-06
LTBR	rs123698	19	807442	C	G	0.61	0.06	0.01	1.39E-08
LTBR	rs1137844	19	35852177	G	C	0.32	0.05	0.01	2.37E-05
LXN	rs6785524	3	158619442	A	C	0.53	-0.17	0.01	8.38E-49
LXN	rs55636108	3	186663473	C	T	0.21	0.08	0.01	5.60E-08
LY6D	rs61816761	1	152313385	A	G	0.02	0.32	0.02	1.22E-17
LY6D	rs2870238	4	76451926	T	C	0.50	0.05	0.01	8.30E-06
LY6D	rs2572925	8	142786489	T	C	0.40	-0.44	0.01	0.00E+00
LY6D	rs77924615	16	20381010	A	G	0.20	-0.07	0.01	3.12E-07
LY6D	rs761061860	19	38280727	G	C	0.17	-0.06	0.01	1.97E-05
LY6D	rs56035820	19	51016454	T	C	0.23	0.06	0.01	2.11E-06
LY75	rs72957586	2	159906176	A	G	0.56	0.93	0.01	0.00E+00
LY75	rs34794906	6	31270081	C	T	0.26	0.05	0.01	1.12E-07
LY75	rs1137827	9	133254328	T	C	0.06	0.10	0.01	1.29E-08
LY75	rs12574844	11	126403491	A	G	0.07	-0.10	0.01	2.06E-10
LY75	rs3184504	12	111446804	C	T	0.52	-0.06	0.01	2.85E-12
LY75	rs3213572	12	120767275	A	G	0.47	-0.03	0.01	2.72E-05
LY75	rs186021206	17	7166093	A	G	0.01	0.94	0.04	4.09E-67
LY9	rs188468174	1	24965206	T	C	0.01	-0.25	0.03	5.48E-07
LY9	rs37455	1	43828556	G	A	0.35	0.07	0.01	5.59E-09
LY9	rs12128261	1	160792994	G	T	0.22	0.60	0.01	0.00E+00
LY9	rs2009581	2	111050100	A	G	0.27	-0.06	0.01	1.53E-07
LY9	rs9824474	3	170027739	G	A	0.37	-0.06	0.01	9.79E-07
LY9	rs7634389	3	187020633	C	T	0.39	-0.07	0.01	9.45E-11
LY9	rs35829610	5	132488170	G	C	0.34	-0.06	0.01	1.06E-07
LY9	-	6	29866672	G	A	0.54	-0.05	0.01	1.27E-06
LY9	rs11185602	7	50259481	G	A	0.33	-0.05	0.01	8.12E-06
LY9	rs778224678	7	151256873	G	T	0.11	0.08	0.01	4.06E-06
LY9	rs505922	9	133273813	C	T	0.32	0.06	0.01	1.47E-06
LY9	rs75071241	11	126362291	A	G	0.06	-0.11	0.01	3.68E-06
LY9	rs3184504	12	111446804	C	T	0.52	-0.12	0.01	6.44E-32
LY9	rs34949455	12	120762594	C	T	0.50	-0.05	0.01	2.60E-05
LY9	rs76428106	13	28029870	C	T	0.01	0.27	0.03	8.48E-08
LY9	rs186021206	17	7166093	A	G	0.01	1.04	0.05	5.67E-47
LY9	rs2725405	17	81246424	C	G	0.44	0.08	0.01	8.53E-14
LY96	rs188468174	1	24965206	T	C	0.01	-0.48	0.03	9.66E-24
LY96	rs35668054	2	71827707	T	C	0.08	-0.12	0.01	1.98E-07
LY96	-	3	177083309	A	C	0.10	-0.08	0.01	8.52E-06
LY96	rs67612510	6	32558659	A	G	0.54	-0.08	0.01	1.06E-08
LY96	rs3804333	6	106279340	T	C	0.20	-0.06	0.01	1.06E-05
LY96	rs6472812	8	74004849	G	A	0.96	-0.20	0.02	2.58E-13
LY96	rs3181356	9	114930602	T	C	0.23	0.06	0.01	5.51E-06

LY96	rs7943728	11	61779596	A	G	0.19	0.08	0.01	2.23E-08
LY96	rs3184504	12	111446804	C	T	0.52	-0.06	0.01	1.60E-07
LY96	rs117775520	14	105707884	A	G	0.04	-0.19	0.02	3.78E-10
LY96	rs3803800	17	7559652	G	A	0.79	-0.08	0.01	5.24E-09
LY96	rs34557412	17	16948873	G	A	0.01	-0.47	0.04	1.27E-11
LY96	rs5763821	22	30153082	C	A	0.39	-0.06	0.01	7.72E-08
LYAR	rs11723339	4	4270159	T	C	0.62	-0.05	0.01	1.40E-05
LYN	rs1354034	3	56815721	C	T	0.60	0.06	0.01	1.60E-06
LYN	rs2055663	4	82639526	C	T	0.86	-0.09	0.01	5.56E-08
LYN	rs3847326	10	63572099	G	A	0.48	-0.05	0.01	1.80E-05
LYN	rs2186882	11	119297660	A	G	0.27	0.07	0.01	3.76E-08
LYN	rs1613662	19	55025227	A	G	0.83	0.07	0.01	2.28E-05
LYPD3	rs1260326	2	27508073	C	T	0.61	0.05	0.01	4.76E-06
LYPD3	rs1047891	2	210675783	A	C	0.31	0.05	0.01	5.07E-06
LYPD3	rs13107325	4	102267552	T	C	0.08	0.08	0.01	2.78E-05
LYPD3	rs2479016	6	2245111	G	A	0.43	-0.08	0.01	2.50E-13
LYPD3	rs112155110	6	32533640	T	C	0.40	-0.05	0.01	1.22E-06
LYPD3	rs7842080	8	133587086	G	A	0.22	0.18	0.01	1.54E-44
LYPD3	-	9	36291499	A	T	0.43	-0.06	0.01	6.01E-08
LYPD3	rs2519093	9	133266456	T	C	0.18	-0.15	0.01	3.13E-28
LYPD3	rs56278466	10	17833858	G	T	0.66	0.05	0.01	1.79E-05
LYPD3	rs139130389	11	72139110	A	C	0.07	-0.47	0.01	1.32E-124
LYPD3	rs10893502	11	126384123	C	T	0.59	-0.07	0.01	1.59E-11
LYPD3	rs2286783	12	519503	A	G	0.40	0.05	0.01	5.97E-06
LYPD3	-	12	123993829	T	C	0.31	0.06	0.01	2.48E-07
LYPD3	rs56990244	16	377999	G	C	0.59	-0.10	0.01	6.11E-21
LYPD3	rs62034713	16	3309124	G	A	0.25	0.16	0.01	2.22E-41
LYPD3	rs186021206	17	7166093	A	G	0.01	0.97	0.05	7.53E-43
LYPD3	rs276937	18	31031095	T	A	0.32	0.13	0.01	9.96E-32
LYPD3	rs708686	19	5840608	T	C	0.27	0.08	0.01	2.66E-11
LYPD3	rs7257767	19	43497193	T	C	0.11	-0.52	0.01	1.55E-198
LYPD3	rs601338	19	48703417	A	G	0.51	0.26	0.01	1.99E-123
LYPD3	rs383510	21	41486440	C	T	0.51	-0.08	0.01	4.27E-14
LYPD8	rs12729620	1	20691678	G	A	0.06	1.06	0.02	0.00E+00
LYPD8	rs12046713	1	55564598	T	C	0.09	0.84	0.01	0.00E+00
LYPD8	rs12022008	1	110298586	C	T	0.09	0.91	0.01	0.00E+00
LYPD8	rs28768088	1	133200121	C	T	0.09	0.84	0.01	0.00E+00
LYPD8	rs12044252	1	248771492	C	T	0.09	0.84	0.01	0.00E+00
LYPD8	rs4556017	7	100989509	T	C	0.85	-0.08	0.01	8.57E-07
LYPD8	-	9	133263362	G	A	0.18	-0.13	0.01	2.55E-22
LYPD8	rs11220520	11	126488013	T	C	0.19	0.10	0.01	1.56E-14
LYPD8	rs186021206	17	7166093	A	G	0.01	0.56	0.05	4.78E-14
LYPD8	rs73338360	17	49193845	C	G	0.10	0.12	0.01	7.51E-12
LYPD8	rs708686	19	5840608	T	C	0.27	0.17	0.01	2.20E-43
LYPD8	rs4760	19	43648948	G	A	0.16	-0.11	0.01	4.52E-14
MAD1L1	rs143579216	7	2225428	A	G	0.00	1.27	0.07	1.15E-28
MAGED1	rs1354034	3	56815721	C	T	0.60	0.06	0.01	3.96E-07
MAGED1	rs342293	7	106731773	G	C	0.46	-0.08	0.01	9.97E-11
MANF	rs11721253	3	51513203	G	A	0.86	0.09	0.01	2.05E-08
MANF	rs1354034	3	56815721	C	T	0.60	0.06	0.01	5.84E-08
MANF	rs3804749	3	123114156	T	C	0.59	-0.05	0.01	2.04E-05
MANF	rs342298	7	106733200	T	C	0.45	-0.06	0.01	5.37E-08
MANF	rs6993770	8	105569300	T	A	0.29	-0.06	0.01	9.84E-06
MANF	-	10	63156270	A	T	0.49	0.06	0.01	1.28E-06
MANF	rs892090	19	55027704	G	T	0.83	0.07	0.01	6.89E-06
MANSC1	rs3917932	1	36478315	G	C	0.58	-0.06	0.01	1.44E-06

MANSC1	rs61747728	1	179557079	T	C	0.04	0.18	0.02	1.22E-09
MANSC1	rs1260326	2	27508073	C	T	0.61	0.08	0.01	4.76E-12
MANSC1	rs75166367	2	162107791	A	G	0.06	-0.12	0.02	3.98E-07
MANSC1	rs11721168	3	58422952	A	G	0.38	0.06	0.01	4.22E-06
MANSC1	rs112926042	4	724801	T	C	0.31	0.06	0.01	8.15E-06
MANSC1	-	4	186236113	C	A	0.51	-0.07	0.01	4.32E-10
MANSC1	rs3130508	6	31181743	A	G	0.26	0.07	0.01	2.87E-07
MANSC1	rs11137085	8	7019946	C	G	0.38	0.06	0.01	3.32E-07
MANSC1	-	9	133263362	G	A	0.18	-0.10	0.01	8.82E-11
MANSC1	rs1059091	11	309127	G	A	0.32	-0.06	0.01	1.62E-06
MANSC1	rs73017394	11	126311952	A	C	0.24	-0.14	0.01	3.40E-25
MANSC1	rs3053800	12	12346197	G	C	0.50	0.05	0.01	1.32E-05
MANSC1	rs9506687	13	21461663	A	G	0.27	0.06	0.01	5.38E-06
MANSC1	rs72756918	15	64185880	A	G	0.12	0.08	0.01	1.16E-05
MANSC1	rs4997081	16	20353912	C	G	0.19	-0.07	0.01	2.60E-06
MANSC1	rs186021206	17	7166093	A	G	0.01	0.72	0.05	1.99E-20
MANSC1	rs6503533	17	40028327	T	C	0.62	-0.07	0.01	1.44E-08
MANSC1	rs55715868	17	81301025	G	A	0.54	-0.06	0.01	3.23E-06
MANSC1	-	19	836186	C	A	0.26	0.09	0.01	1.20E-11
MANSC1	rs36013629	19	49539610	G	T	0.25	0.06	0.01	1.99E-05
MAP2K6	rs342298	7	106733200	T	C	0.46	-0.06	0.01	9.68E-08
MAP2K6	rs3847326	10	63572099	G	A	0.48	-0.06	0.01	3.07E-07
MAP2K6	rs60822569	12	54323724	C	T	0.55	0.05	0.01	9.06E-06
MAP3K5	rs1354034	3	56815721	C	T	0.60	0.06	0.01	1.85E-06
MAP4K5	rs3811444	1	247876149	T	C	0.33	-0.06	0.01	7.73E-06
MAP4K5	rs342293	7	106731773	G	C	0.46	-0.05	0.01	2.57E-05
MAP4K5	rs17780143	14	50435050	A	G	0.06	-0.38	0.02	2.63E-54
MAPK9	rs3763131	5	180307312	C	T	0.20	0.14	0.01	8.64E-24
MARCO	rs12740374	1	109274968	T	G	0.22	-0.13	0.01	1.37E-22
MARCO	rs4672487	2	62268110	G	T	0.57	-0.09	0.01	1.12E-15
MARCO	rs35288880	2	118970896	T	C	0.16	-0.35	0.01	1.07E-117
MARCO	rs62165726	2	134208991	A	C	0.04	-0.36	0.02	4.48E-34
MARCO	rs2470750	3	98971748	T	A	0.39	-0.07	0.01	6.30E-10
MARCO	rs112125244	5	132477205	G	T	0.29	-0.06	0.01	1.35E-06
MARCO	-	6	105909664	C	T	0.52	-0.09	0.01	8.52E-18
MARCO	rs12667978	7	129137407	T	C	0.57	0.05	0.01	2.37E-06
MARCO	-	11	44570719	T	A	0.78	0.07	0.01	3.56E-07
MARCO	rs73338697	14	94516256	A	G	0.01	-0.31	0.04	3.35E-06
MARCO	rs11861563	16	75175892	A	G	0.59	-0.06	0.01	8.46E-08
MARCO	rs186021206	17	7166093	A	G	0.01	1.10	0.05	4.26E-50
MARCO	-	19	55313449	C	G	0.39	0.05	0.01	1.14E-05
MASP1	rs12136468	1	11039731	A	G	0.79	0.12	0.01	2.26E-20
MASP1	-	1	27373179	A	G	0.01	-1.17	0.03	4.30E-143
MASP1	rs3820897	2	3594771	C	T	0.82	0.19	0.01	5.42E-41
MASP1	rs1486236	2	179874723	A	C	0.37	0.32	0.01	2.00E-168
MASP1	rs698090	3	187246512	T	C	0.64	0.21	0.01	3.24E-77
MASP1	rs9987289	8	9325848	G	A	0.91	-0.09	0.01	3.98E-06
MASP1	rs41341748	8	16155085	A	G	0.01	0.27	0.03	4.14E-07
MASP1	rs10104003	8	54509880	T	C	0.21	0.10	0.01	2.69E-14
MASP1	rs7899547	10	52777079	G	T	0.65	0.13	0.01	1.70E-32
MASP1	rs4926	11	57614516	A	G	0.27	-0.09	0.01	1.65E-13
MASP1	rs215223	12	481093	A	G	0.40	-0.10	0.01	1.23E-19
MASP1	rs6118	14	94587526	T	C	0.10	0.10	0.01	4.63E-08
MASP1	rs704	17	28367840	A	G	0.47	-0.07	0.01	6.69E-11
MASP1	rs9944724	18	62517378	G	T	0.38	0.06	0.01	8.55E-08
MATN2	rs13159365	5	136053744	T	C	0.51	0.08	0.01	2.31E-14

MATN2	rs2685412	8	27953086	G	C	0.27	0.25	0.01	4.34E-93
MATN2	rs1869609	8	97978993	G	A	0.66	0.39	0.01	6.69E-252
MATN2	rs35157100	9	115138014	A	G	0.29	-0.17	0.01	1.75E-46
MATN2	rs5786398	10	79383631	T	C	0.38	0.05	0.01	1.98E-05
MATN2	rs2269434	11	47338861	C	T	0.35	0.05	0.01	5.64E-06
MATN2	rs77542162	17	69085137	G	A	0.02	0.16	0.02	1.09E-05
MATN3	rs11694716	2	20007858	A	G	0.64	-0.56	0.01	0.00E+00
MATN3	rs66530140	4	186240057	C	T	0.51	-0.06	0.01	6.83E-08
MATN3	rs2519093	9	133266456	T	C	0.18	-0.08	0.01	1.56E-10
MATN3	rs11657101	17	62559465	A	G	0.36	-0.12	0.01	1.11E-29
MAVS	rs1354034	3	56815721	C	T	0.60	0.05	0.01	2.78E-05
MAVS	rs7080386	10	63288546	A	C	0.41	0.05	0.01	3.29E-06
MAVS	rs45437096	20	3864282	T	C	0.14	-0.13	0.01	2.25E-15
MAX	rs17622656	5	132485305	A	G	0.39	-0.06	0.01	5.74E-07
MAX	rs342298	7	106733200	T	C	0.45	-0.05	0.01	7.39E-06
MAX	rs6993770	8	105569300	T	A	0.29	-0.06	0.01	2.59E-05
MAX	rs7896518	10	63344740	G	A	0.42	0.06	0.01	1.25E-06
MAX	rs60822569	12	54323724	C	T	0.55	0.05	0.01	2.49E-05
MAX	rs8005829	14	65109447	T	C	0.58	0.08	0.01	2.73E-11
MAX	rs12588296	14	106627735	C	A	0.25	-0.07	0.01	1.25E-06
MAX	rs200309755	17	29518756	T	C	0.54	0.05	0.01	1.87E-05
MB	rs12463674	2	178567458	G	A	0.32	0.07	0.01	1.72E-11
MB	rs2151597	9	100581119	A	G	0.44	0.05	0.01	9.57E-07
MB	rs17099139	10	119659975	G	C	0.27	0.05	0.01	1.08E-05
MB	rs7481951	11	22250324	T	A	0.59	0.07	0.01	2.72E-11
MB	rs1028883	13	73534450	G	T	0.57	0.06	0.01	1.96E-09
MB	rs4901541	14	54768564	T	C	0.56	-0.05	0.01	2.39E-07
MB	rs1799938	17	67056188	A	G	0.11	0.07	0.01	1.88E-05
MB	rs41155	22	30008558	C	A	0.59	0.05	0.01	1.01E-05
MCAM	rs1260326	2	27508073	C	T	0.61	0.07	0.01	6.45E-10
MCAM	rs13107325	4	102267552	T	C	0.08	0.11	0.01	5.25E-07
MCAM	rs41289902	6	112139163	T	C	0.01	0.49	0.03	5.23E-27
MCAM	rs8176693	9	133262254	T	C	0.06	0.23	0.01	3.56E-24
MCAM	rs34587557	11	119314967	C	T	0.05	-0.31	0.02	8.87E-37
MCAM	rs11220462	11	126374057	A	G	0.13	-0.10	0.01	1.07E-09
MCAM	rs7310615	12	111427245	G	C	0.52	0.06	0.01	5.21E-07
MCAM	rs186021206	17	7166093	A	G	0.01	0.96	0.05	1.31E-38
MCAM	rs2006987	22	39459077	C	T	0.75	-0.07	0.01	2.07E-08
MCFD2	rs6419615	2	46925152	C	G	0.71	-0.14	0.01	7.83E-30
MCFD2	rs6993770	8	105569300	T	A	0.29	-0.08	0.01	1.06E-09
MCFD2	rs112635299	14	94371805	T	G	0.02	0.34	0.03	6.03E-18
MCFD2	rs17696641	18	59348568	A	T	0.15	0.08	0.01	2.68E-07
MDGA1	rs9349050	6	37695848	C	T	0.51	-1.08	0.01	0.00E+00
MDGA1	rs9987289	8	9325848	G	A	0.91	-0.06	0.01	5.96E-07
MDGA1	rs35887873	11	126349501	C	T	0.23	0.07	0.01	1.21E-16
MDGA1	rs186021206	17	7166093	A	G	0.01	0.50	0.03	2.66E-24
MDGA1	rs77542162	17	69085137	G	A	0.02	0.14	0.02	6.80E-09
MDGA1	rs7287617	22	39501158	A	G	0.65	0.04	0.00	3.79E-07
MDK	rs6427756	1	199031598	G	A	0.73	-0.08	0.01	7.46E-10
MDK	rs7618405	3	18209017	A	C	0.20	-0.07	0.01	2.23E-06
MDK	rs1354034	3	56815721	C	T	0.60	-0.06	0.01	1.01E-06
MDK	rs6961069	7	80589645	T	C	0.40	0.05	0.01	1.29E-05
MDK	rs6993770	8	105569300	T	A	0.29	-0.08	0.01	6.45E-10
MDK	rs7080386	10	63288546	A	C	0.41	0.07	0.01	7.53E-10
MDK	rs541295226	11	46380789	G	A	0.00	-1.66	0.10	2.26E-26
MDK	rs117125830	11	56252294	T	C	0.01	-0.51	0.05	6.59E-12

MDK	rs3184504	12	111446804	C	T	0.52	-0.06	0.01	1.86E-08
MDK	rs12445050	16	81837364	T	C	0.14	0.08	0.01	4.42E-06
MDK	rs57839578	17	5365042	T	A	0.48	-0.05	0.01	8.94E-06
MDK	rs892090	19	55027704	G	T	0.83	0.13	0.01	1.61E-16
MDK	rs14142	20	45345427	T	C	0.37	-0.06	0.01	2.79E-07
MED18	rs12245149	10	63561387	A	C	0.49	-0.06	0.01	1.11E-06
MED18	rs200309755	17	29518756	T	C	0.54	0.06	0.01	5.39E-06
MEGF10	-	3	12807512	T	A	0.66	0.07	0.01	4.48E-10
MEGF10	rs766512750	3	98922306	T	C	0.18	0.06	0.01	1.45E-05
MEGF10	rs112394041	5	127292892	T	C	0.46	0.40	0.01	1.54E-284
MEGF10	rs200889152	6	117186819	C	A	0.37	-0.06	0.01	2.62E-09
MEGF10	rs4841132	8	9326086	G	A	0.91	-0.08	0.01	1.62E-05
MEGF10	rs10813959	9	33166236	C	T	0.42	0.08	0.01	2.06E-13
MEGF10	rs508196	10	17974944	T	C	0.44	-0.05	0.01	3.48E-07
MEGF10	rs7232	11	60173126	A	T	0.37	0.05	0.01	1.45E-06
MEGF10	rs2236653	11	126413890	T	C	0.43	0.05	0.01	1.88E-06
MEGF10	rs10849082	12	482922	C	T	0.47	-0.06	0.01	2.47E-08
MEGF10	rs632887	12	3283185	G	A	0.41	0.08	0.01	1.62E-14
MEGF10	-	12	48210711	A	T	0.56	-0.05	0.01	1.37E-06
MEGF10	rs199957765	14	35256291	A	C	0.40	0.05	0.01	1.61E-05
MEGF10	rs3007036	14	49639327	G	A	0.54	0.06	0.01	2.53E-08
MEGF10	rs186021206	17	7166093	A	G	0.01	0.67	0.05	6.29E-21
MEGF10	rs77542162	17	69085137	G	A	0.02	0.79	0.02	2.64E-118
MEGF10	rs6068599	20	53643079	C	T	0.68	-0.05	0.01	1.04E-06
MEGF10	-	22	19094017	T	A	0.19	-0.07	0.01	5.56E-07
MEGF9	rs646776	1	109275908	T	C	0.78	-0.08	0.01	1.23E-09
MEGF9	rs12732792	1	178669563	A	G	0.48	0.05	0.01	1.32E-06
MEGF9	-	3	136181921	A	T	0.70	0.06	0.01	1.76E-06
MEGF9	rs13107325	4	102267552	T	C	0.08	0.18	0.01	4.08E-17
MEGF9	rs80318131	7	129143002	C	T	0.31	0.06	0.01	1.04E-06
MEGF9	rs1461729	8	9329732	G	A	0.90	-0.09	0.01	1.01E-06
MEGF9	rs28601761	8	125487789	G	C	0.42	-0.08	0.01	2.03E-12
MEGF9	rs7849566	9	120698491	C	A	0.70	0.34	0.01	7.87E-172
MEGF9	rs9633740	10	80505515	G	A	0.80	0.08	0.01	3.14E-08
MEGF9	rs751229834	11	61808335	C	A	0.31	0.08	0.01	6.67E-10
MEGF9	rs11045819	12	21176879	A	C	0.16	0.15	0.01	1.85E-23
MEGF9	rs186021206	17	7166093	A	G	0.01	0.46	0.05	3.14E-09
MEGF9	rs77542162	17	69085137	G	A	0.02	0.26	0.02	5.39E-12
MEGF9	rs73066226	19	58496846	C	T	0.16	0.08	0.01	1.61E-07
MEP1B	rs75460349	1	26853597	C	A	0.02	-0.15	0.02	6.73E-10
MEP1B	rs1730862	1	107071381	A	G	0.66	0.04	0.01	5.86E-07
MEP1B	rs1260326	2	27508073	C	T	0.61	0.06	0.00	1.24E-13
MEP1B	rs370213063	3	136744673	C	T	0.31	-0.04	0.01	1.90E-06
MEP1B	-	4	105293951	T	C	0.41	0.03	0.00	1.04E-05
MEP1B	rs4895864	6	130043337	T	C	0.57	0.04	0.00	2.78E-07
MEP1B	rs6973520	7	129100389	T	C	0.51	0.04	0.00	3.04E-07
MEP1B	rs3735165	7	150371282	C	T	0.31	0.07	0.01	1.08E-19
MEP1B	rs112875651	8	125494452	A	G	0.39	0.04	0.00	5.71E-08
MEP1B	rs174533	11	61781553	A	G	0.35	0.05	0.01	1.96E-11
MEP1B	rs11045856	12	21197755	G	T	0.24	0.04	0.01	9.18E-07
MEP1B	rs7979473	12	120982457	G	A	0.62	-0.05	0.00	1.94E-11
MEP1B	rs17616063	16	51402971	G	A	0.07	-0.10	0.01	3.90E-13
MEP1B	rs16940186	16	85976134	C	T	0.15	0.13	0.01	8.90E-38
MEP1B	rs4564600	17	76609022	A	G	0.56	-0.04	0.00	7.37E-09
MEP1B	rs680321	18	32217995	C	T	0.46	-1.07	0.01	0.00E+00
MEP1B	rs1800961	20	44413724	T	C	0.03	0.10	0.01	1.74E-06

MEPE	rs181163165	1	92486835	T	C	0.00	-0.43	0.06	9.24E-07
MEPE	rs1393691	1	219601777	T	G	0.52	-0.08	0.01	2.39E-13
MEPE	rs6547692	2	27512105	A	G	0.56	0.05	0.01	2.30E-05
MEPE	rs77623237	2	229249783	C	T	0.18	-0.14	0.01	2.33E-24
MEPE	rs17013212	4	87832908	T	C	0.14	-0.19	0.01	8.14E-33
MEPE	rs12509937	4	186239096	T	C	0.51	-0.06	0.01	1.40E-08
MEPE	rs55712380	6	139288987	T	G	0.28	-0.05	0.01	1.96E-05
MEPE	rs757980	7	28685919	A	G	0.75	0.07	0.01	1.17E-07
MEPE	rs7842942	8	118996348	C	T	0.57	0.06	0.01	4.33E-09
MEPE	rs201265508	11	180231	G	A	0.29	0.07	0.01	1.52E-06
MEPE	rs3008070	13	22452312	G	T	0.22	-0.07	0.01	5.67E-07
MEPE	rs806292	13	50236895	G	A	0.53	-0.06	0.01	1.21E-07
MEPE	rs28929474	14	94378610	T	C	0.02	0.16	0.02	2.79E-05
MEPE	rs2614763	15	50850536	A	C	0.45	0.10	0.01	3.67E-21
MEPE	rs111791352	15	67280330	A	T	0.23	0.06	0.01	1.19E-06
MEPE	rs10521142	16	27118834	G	A	0.56	0.08	0.01	1.59E-12
MEPE	rs78005814	17	46047809	C	A	0.35	0.05	0.01	1.81E-05
MERTK	rs10779835	1	230164203	C	T	0.61	-0.09	0.01	6.86E-16
MERTK	rs10188642	2	111983522	A	G	0.63	-0.26	0.01	2.29E-125
MERTK	rs10935473	3	98698056	T	G	0.44	-0.07	0.01	4.61E-12
MERTK	rs35371668	6	32593861	T	C	0.18	-0.11	0.01	1.45E-13
MERTK	-	9	133263362	G	A	0.18	-0.16	0.01	6.51E-30
MERTK	rs11596680	10	19919554	G	A	0.33	0.07	0.01	6.53E-09
MERTK	rs7088799	10	63256414	G	T	0.41	0.07	0.01	3.03E-11
MERTK	-	11	61774534	G	A	0.35	0.07	0.01	6.67E-10
MERTK	rs494144	11	89465628	C	T	0.54	-0.08	0.01	8.23E-15
MERTK	-	12	21191379	G	A	0.24	0.16	0.01	4.19E-35
MERTK	rs9738226	12	120985856	G	A	0.62	-0.06	0.01	1.45E-08
MERTK	rs28929474	14	94378610	T	C	0.02	0.20	0.02	1.20E-07
MERTK	rs423135	16	89674465	A	G	0.52	0.07	0.01	2.44E-10
MERTK	rs186021206	17	7166093	A	G	0.01	0.61	0.05	7.69E-17
MERTK	rs12451766	17	47543895	G	A	0.50	0.06	0.01	3.91E-07
MERTK	rs58895965	19	35060524	A	C	0.18	0.07	0.01	1.65E-06
MERTK	rs111981233	19	49513222	G	T	0.08	0.09	0.01	9.29E-06
MESD	rs1354034	3	56815721	C	T	0.60	0.07	0.01	3.23E-09
MESD	rs17622656	5	132485305	A	G	0.39	-0.05	0.01	2.66E-05
MESD	rs342298	7	106733200	T	C	0.45	-0.05	0.01	2.38E-05
MESD	rs6993770	8	105569300	T	A	0.29	-0.09	0.01	1.90E-11
MET	rs79094524	1	39575699	A	G	0.23	-0.06	0.01	1.26E-05
MET	rs4855845	3	49649610	C	T	0.82	-0.06	0.01	9.38E-06
MET	rs5398	3	170998041	A	G	0.28	-0.09	0.01	4.03E-14
MET	rs1858830	7	116672385	G	C	0.44	-0.16	0.01	5.27E-44
MET	rs7799998	7	129098896	G	A	0.45	-0.07	0.01	3.50E-11
MET	rs507666	9	133273983	A	G	0.18	-0.33	0.01	1.22E-123
MET	rs10761779	10	63515167	G	A	0.47	0.07	0.01	4.25E-10
MET	rs10748526	10	80513323	C	T	0.79	0.12	0.01	6.69E-21
MET	rs174547	11	61803311	C	T	0.34	0.10	0.01	2.08E-19
MET	rs11045819	12	21176879	A	C	0.16	0.12	0.01	5.62E-16
MET	rs2464190	12	120977587	C	T	0.41	0.05	0.01	1.27E-06
MET	rs1041316	14	56633141	A	G	0.73	0.08	0.01	3.40E-11
MET	rs28929474	14	94378610	T	C	0.02	0.31	0.02	9.65E-17
MET	rs1982784	16	4616536	G	A	0.81	0.07	0.01	3.01E-06
MET	rs4850	16	21965441	A	G	0.06	0.15	0.02	7.34E-10
MET	rs186021206	17	7166093	A	G	0.01	0.56	0.05	6.32E-14
MET	rs34349423	17	49782701	C	A	0.63	-0.05	0.01	2.71E-05
MET	rs77542162	17	69085137	G	A	0.02	0.38	0.02	2.19E-26

MET	rs10401969	19	19296909	C	T	0.08	-0.11	0.01	2.55E-07
MET	rs112167630	19	42370749	A	G	0.03	0.26	0.02	1.77E-15
MET	rs914187	21	41247822	C	T	0.60	-0.05	0.01	1.11E-06
METAP1D	rs138869820	2	172066280	A	G	0.00	-0.81	0.09	1.51E-08
METAP1D	rs6993770	8	105569300	T	A	0.29	-0.06	0.01	1.57E-06
METAP1D	rs6580980	12	54298277	G	A	0.56	0.06	0.01	2.36E-07
METAP2	rs1354034	3	56815721	C	T	0.60	0.10	0.01	1.76E-17
METAP2	rs6993770	8	105569300	T	A	0.29	-0.07	0.01	9.03E-08
METAP2	-	10	63156270	A	T	0.49	0.05	0.01	9.44E-06
METAP2	rs11502185	11	180258	C	T	0.26	0.07	0.01	5.33E-06
METAP2	rs11170874	12	54332691	G	A	0.33	0.06	0.01	4.34E-06
METAP2	rs10405535	19	32581179	G	A	0.68	0.10	0.01	8.84E-15
MFAP5	rs4894803	3	172082466	G	A	0.41	0.07	0.01	6.68E-11
MFAP5	rs1918352	7	100165816	A	G	0.55	0.05	0.01	3.15E-06
MFAP5	rs532436	9	133274414	A	G	0.18	-0.08	0.01	3.28E-09
MFAP5	rs4132450	12	8662099	T	C	0.00	-1.70	0.06	4.23E-82
MFAP5	rs1126464	16	89637957	C	G	0.24	-0.06	0.01	1.56E-05
MFAP5	rs7412	19	44908822	T	C	0.08	0.09	0.01	7.04E-06
MFGE8	rs777231403	1	62634374	A	G	0.35	-0.06	0.01	2.16E-07
MFGE8	rs1260326	2	27508073	C	T	0.61	-0.06	0.01	6.34E-08
MFGE8	rs56210800	3	124753745	G	C	0.13	-0.08	0.01	8.90E-07
MFGE8	rs11134475	5	156972939	G	A	0.64	0.06	0.01	4.66E-07
MFGE8	rs521977	6	31869050	G	T	0.68	0.07	0.01	1.34E-08
MFGE8	rs17145750	7	73612048	T	C	0.16	-0.08	0.01	1.85E-07
MFGE8	rs200646600	8	19998347	A	T	0.23	-0.07	0.01	1.52E-07
MFGE8	rs2980888	8	125495066	C	T	0.70	-0.07	0.01	5.67E-08
MFGE8	rs7232	11	60173126	A	T	0.37	0.19	0.01	3.14E-60
MFGE8	rs964184	11	116778201	C	G	0.87	-0.16	0.01	5.90E-23
MFGE8	rs2141247	12	124366207	A	C	0.61	0.08	0.01	1.33E-12
MFGE8	rs6602913	13	113841152	C	A	0.37	0.06	0.01	6.73E-08
MFGE8	rs139566989	15	58434769	T	C	0.78	-0.08	0.01	3.01E-08
MFGE8	rs34239095	15	88912537	G	C	0.28	0.37	0.01	2.63E-203
MFGE8	rs34954997	19	44914381	G	C	0.24	0.06	0.01	6.17E-06
MFGE8	rs189448562	20	3724468	C	G	0.01	0.33	0.03	1.15E-09
MGLL	rs7578	3	127689396	A	G	0.56	0.06	0.01	3.47E-07
MGLL	rs6993770	8	105569300	T	A	0.29	-0.09	0.01	1.64E-12
MGLL	rs77303550	16	72045758	T	C	0.20	0.07	0.01	7.80E-06
MGMT	rs1354034	3	56815721	C	T	0.60	0.06	0.01	7.42E-07
MGMT	rs342298	7	106733200	T	C	0.46	-0.05	0.01	1.80E-06
MGMT	-	10	63156270	A	T	0.49	0.05	0.01	3.97E-06
MGMT	rs1008982	10	129647467	C	T	0.37	-0.41	0.01	2.86E-269
MIA	rs4257489	3	98914437	C	A	0.34	-0.05	0.01	1.28E-05
MIA	rs4917	3	186619924	C	T	0.64	0.06	0.01	4.88E-08
MIA	rs28817415	4	76480299	T	C	0.46	0.05	0.01	5.61E-08
MIA	rs60028714	4	85472147	T	C	0.62	-0.05	0.01	1.07E-06
MIA	rs3132568	6	31134140	G	A	0.26	-0.05	0.01	5.84E-06
MIA	rs77542162	17	69085137	G	A	0.02	0.27	0.02	4.18E-16
MIA	rs2233159	19	40777460	G	C	0.93	-1.37	0.01	0.00E+00
MICB_MICA	rs2009581	2	111050100	A	G	0.27	-0.03	0.00	1.11E-05
MICB_MICA	rs10935473	3	98698056	T	G	0.44	-0.10	0.00	5.46E-66
MICB_MICA	rs3132467	6	31413166	A	G	0.26	-1.10	0.01	0.00E+00
MICB_MICA	-	9	133263362	G	A	0.18	-0.09	0.00	2.37E-32
MICB_MICA	rs186862582	12	10352001	G	A	0.00	-0.34	0.04	8.80E-07
MICB_MICA	rs3184504	12	111446804	C	T	0.52	-0.03	0.00	1.06E-05
MICB_MICA	rs35715239	15	55332404	C	G	0.33	-0.05	0.00	4.41E-18
MICB_MICA	rs186021206	17	7166093	A	G	0.01	0.34	0.03	5.50E-18

MICB_MICA	rs4760	19	43648948	G	A	0.16	0.04	0.01	4.69E-07
MIF	rs1354034	3	56815721	C	T	0.60	0.07	0.01	9.23E-09
MIF	rs10733789	10	63188924	C	T	0.31	0.06	0.01	8.97E-06
MIF	rs11502185	11	180258	C	T	0.26	0.06	0.01	2.42E-05
MIF	rs60822569	12	54323724	C	T	0.55	0.05	0.01	7.61E-06
MIF	rs2330634	22	23908608	G	C	0.60	-0.11	0.01	1.85E-20
MILR1	rs37471	1	43811876	T	C	0.35	0.05	0.01	1.52E-05
MILR1	rs1260326	2	27508073	C	T	0.61	0.05	0.01	9.62E-06
MILR1	rs140655352	3	98831392	T	A	0.09	0.20	0.01	2.09E-25
MILR1	rs2524119	6	31261627	C	T	0.54	0.06	0.01	3.55E-08
MILR1	rs6984305	8	9320758	T	A	0.89	-0.07	0.01	2.50E-05
MILR1	rs4055121	11	126362442	T	C	0.13	-0.10	0.01	8.98E-11
MILR1	rs1168948	12	120892562	A	C	0.49	0.05	0.01	1.08E-06
MILR1	rs186021206	17	7166093	A	G	0.01	1.25	0.05	1.09E-57
MILR1	rs704	17	28367840	A	G	0.47	-0.06	0.01	2.26E-07
MILR1	rs138176943	17	64468504	A	G	0.06	0.79	0.02	7.29E-229
MITD1	rs13009147	2	99187535	G	T	0.65	-0.07	0.01	1.42E-09
MITD1	rs1354034	3	56815721	C	T	0.60	0.06	0.01	1.14E-06
MLN	rs870465	1	3323635	A	G	0.17	0.06	0.01	1.51E-05
MLN	rs826404	1	117622100	C	T	0.59	-0.06	0.01	3.14E-09
MLN	rs11240492	1	205492095	C	G	0.87	0.10	0.01	3.63E-11
MLN	rs16862260	2	221205237	A	G	0.21	-0.22	0.01	7.45E-71
MLN	rs147351977	3	184221870	A	G	0.03	0.14	0.02	1.18E-05
MLN	rs76405485	5	7338998	C	T	0.35	-0.07	0.01	1.72E-10
MLN	rs56092644	5	96439105	T	G	0.24	0.08	0.01	1.30E-11
MLN	rs67131976	6	20686647	T	C	0.17	0.17	0.01	2.95E-34
MLN	rs1547671	6	33809483	T	C	0.54	-0.41	0.01	0.00E+00
MLN	rs13215052	6	41714097	A	G	0.09	0.09	0.01	5.22E-06
MLN	rs688949	6	116927769	G	A	0.18	0.10	0.01	1.23E-13
MLN	rs12114906	8	103976556	C	T	0.25	-0.06	0.01	1.17E-07
MLN	rs10982164	9	114328787	A	G	0.07	0.18	0.01	1.16E-16
MLN	rs782134971	9	133264504	G	C	0.25	-0.07	0.01	5.48E-10
MLN	rs4309096	10	46458013	C	T	0.65	0.07	0.01	1.34E-09
MLN	rs5785928	10	69560898	T	C	0.69	-0.06	0.01	2.18E-07
MLN	rs11213809	11	111265020	G	A	0.66	-0.05	0.01	3.63E-06
MLN	rs2485287	13	37945144	C	A	0.47	0.08	0.01	8.79E-14
MLN	rs11634163	15	62038434	T	C	0.40	0.06	0.01	5.49E-09
MLN	rs566487891	17	336025	C	G	0.79	0.06	0.01	4.48E-06
MLN	rs17681738	17	9889923	T	C	0.33	0.05	0.01	7.37E-07
MLN	rs74175746	19	23301088	A	T	0.18	0.07	0.01	6.54E-07
MLN	rs2251034	19	48704535	A	G	0.53	-0.07	0.01	3.26E-13
MLN	rs2208150	20	16922822	C	T	0.59	0.08	0.01	3.06E-16
MME	rs1497406	1	16178825	G	A	0.58	0.08	0.01	2.24E-13
MME	rs4687657	3	52818522	T	G	0.26	0.58	0.01	0.00E+00
MME	rs79837905	3	155067802	G	A	0.08	-0.11	0.01	3.77E-07
MME	rs4835265	4	145900258	A	C	0.16	0.12	0.01	4.60E-17
MME	rs10075805	5	31021251	G	A	0.28	-0.05	0.01	2.50E-05
MME	rs112875651	8	125494452	A	G	0.39	-0.05	0.01	7.68E-06
MME	rs151068477	10	50882756	T	G	0.01	0.27	0.04	1.70E-05
MME	rs1650146	10	77928450	G	A	0.62	0.05	0.01	6.59E-06
MME	rs17674290	12	20845175	G	T	0.15	0.06	0.01	1.41E-05
MME	rs59643720	14	103098470	C	A	0.24	0.11	0.01	6.52E-20
MME	rs12373325	18	58420416	C	T	0.80	-0.10	0.01	6.48E-14
MME	rs58659609	19	3482461	A	C	0.18	0.07	0.01	1.48E-07
MME	rs189448562	20	3724468	C	G	0.01	0.28	0.03	2.86E-08
MME	rs12169946	22	17985339	G	A	0.26	-0.06	0.01	1.38E-06

MMP1	rs12136856	1	156503322	G	C	0.65	0.09	0.01	6.90E-18
MMP1	rs12622659	2	68419147	A	G	0.30	-0.06	0.01	5.40E-07
MMP1	rs76036957	2	112738876	A	G	0.01	0.31	0.04	2.66E-08
MMP1	rs28706788	5	160148021	A	G	0.54	-0.05	0.01	2.89E-07
MMP1	rs5879021	6	109314355	C	G	0.52	0.07	0.01	9.39E-11
MMP1	rs6961069	7	80589645	T	C	0.40	0.05	0.01	2.50E-05
MMP1	rs6993770	8	105569300	T	A	0.29	-0.16	0.01	3.65E-43
MMP1	rs10820606	9	96430637	C	A	0.23	0.08	0.01	2.77E-11
MMP1	rs150813342	9	132989126	T	C	0.01	-0.25	0.04	1.97E-05
MMP1	-	10	99528354	C	A	0.28	0.07	0.01	3.59E-08
MMP1	rs471994	11	102827000	A	G	0.36	-0.46	0.01	0.00E+00
MMP1	rs60822569	12	54323724	C	T	0.55	0.05	0.01	7.42E-07
MMP1	rs757848155	12	56757619	C	A	0.64	0.05	0.01	3.49E-06
MMP1	rs12445050	16	81837364	T	C	0.14	0.07	0.01	2.30E-06
MMP1	rs1654425	19	55027612	C	T	0.83	0.12	0.01	2.56E-17
MMP1	-	20	19334040	T	A	0.38	0.06	0.01	6.02E-08
MMP10	rs187265184	6	32557992	G	C	0.36	0.06	0.01	4.67E-06
MMP10	rs486055	11	102779693	T	C	0.16	-0.40	0.01	4.71E-146
MMP10	rs681343	19	48703205	T	C	0.51	0.13	0.01	5.95E-35
MMP12	rs2770	6	31354030	A	G	0.45	-0.05	0.01	1.28E-06
MMP12	rs17368814	11	102877965	G	A	0.13	-0.74	0.01	0.00E+00
MMP12	rs61729512	12	7485173	A	G	0.12	0.11	0.01	4.76E-12
MMP12	rs12986064	19	54251270	C	T	0.51	-0.08	0.01	1.53E-15
MMP13	rs562736	11	102956184	T	A	0.56	-0.14	0.01	4.35E-33
MMP3	rs41341748	8	16155085	A	G	0.01	0.25	0.03	5.97E-11
MMP3	rs678815	11	102843046	C	G	0.48	0.48	0.01	0.00E+00
MMP3	rs9895446	17	69473737	C	T	0.06	-0.07	0.01	2.59E-05
MMP3	rs2267372	22	38202227	G	A	0.60	0.05	0.01	4.57E-10
MMP7	rs6694034	1	202034859	C	A	0.45	0.11	0.01	2.00E-22
MMP7	rs11568819	11	102530902	A	G	0.06	0.57	0.01	7.00E-139
MMP7	rs61729512	12	7485173	A	G	0.12	0.08	0.01	5.68E-06
MMP7	rs77924615	16	20381010	A	G	0.20	-0.09	0.01	4.82E-11
MMP7	rs12975366	19	54255498	C	T	0.39	-0.06	0.01	3.13E-08
MMP8	-	11	47280489	T	A	0.11	-0.13	0.01	4.10E-13
MMP8	rs2511241	11	73234296	T	C	0.93	0.19	0.01	2.18E-18
MMP8	rs35231465	11	102713404	A	G	0.02	-1.25	0.02	1.02E-266
MMP8	-	17	39988115	T	A	0.54	-0.09	0.01	3.16E-16
MMP9	rs1886654	1	235942610	C	T	0.89	-0.08	0.01	5.06E-06
MMP9	rs28498283	2	43132926	T	A	0.25	0.06	0.01	9.37E-06
MMP9	rs4619875	4	152779978	T	C	0.40	-0.06	0.01	4.28E-07
MMP9	rs9325122	5	148823373	T	C	0.55	-0.05	0.01	2.00E-05
MMP9	-	6	31448099	T	G	0.30	0.07	0.01	3.32E-09
MMP9	rs2158799	7	28237488	G	C	0.61	0.05	0.01	5.05E-06
MMP9	rs2511241	11	73234296	T	C	0.93	0.38	0.01	5.94E-70
MMP9	rs2289511	14	87988566	A	G	0.58	-0.06	0.01	4.55E-07
MMP9	rs3826331	17	39994239	C	T	0.60	-0.08	0.01	1.25E-13
MMP9	rs17576	20	46011586	G	A	0.35	0.14	0.01	5.04E-34
MNDA	rs6503533	17	40028327	T	C	0.62	-0.05	0.01	1.30E-05
MOG	rs9461541	6	29657418	G	A	0.05	-0.45	0.02	1.87E-72
MOG	rs8021280	14	74823315	G	C	0.46	0.06	0.01	3.95E-07
MPHOSPH8	rs1354034	3	56815721	C	T	0.60	0.07	0.01	4.16E-09
MPHOSPH8	-	10	63156270	A	T	0.49	0.06	0.01	6.17E-07
MPHOSPH8	rs35424555	13	19647376	G	A	0.12	0.09	0.01	7.07E-07
MPI	rs1354034	3	56815721	C	T	0.60	0.07	0.01	2.66E-09
MPI	rs17622656	5	132485305	A	G	0.39	-0.05	0.01	1.14E-05
MPI	rs7080386	10	63288546	A	C	0.41	0.06	0.01	1.64E-07

MPI	rs11502185	11	180258	C	T	0.26	0.06	0.01	2.11E-05
MPI	rs4886632	15	74881584	T	G	0.58	0.20	0.01	1.37E-71
MPIG6B	rs6961069	7	80589645	T	C	0.40	0.06	0.01	2.22E-06
MPIG6B	rs6993770	8	105569300	T	A	0.29	-0.10	0.01	6.73E-15
MPIG6B	rs7896518	10	63344740	G	A	0.42	0.08	0.01	2.24E-10
MPIG6B	rs1362213	12	6173262	T	C	0.48	-0.06	0.01	2.99E-06
MPIG6B	rs892090	19	55027704	G	T	0.83	0.10	0.01	9.58E-11
MPO	rs2120782	1	229534642	C	G	0.21	0.09	0.01	7.74E-10
MPO	rs7748270	6	32480822	T	C	0.52	0.05	0.01	1.69E-05
MPO	rs13277237	8	129592317	A	G	0.57	-0.07	0.01	9.60E-11
MPO	rs757081	11	17330136	G	C	0.33	0.05	0.01	1.21E-05
MPO	rs12941811	17	40003082	C	T	0.57	-0.06	0.01	1.49E-06
MPO	rs34097845	17	58281068	T	C	0.06	-0.50	0.02	1.39E-90
MPO	rs3790320	20	17638809	A	G	0.39	0.07	0.01	6.56E-09
MSLN	rs72851462	2	25500169	C	G	0.12	-0.10	0.01	1.77E-10
MSLN	-	6	31099297	T	A	0.32	-0.07	0.01	9.01E-10
MSLN	rs1214761	6	43386693	G	A	0.68	-0.09	0.01	6.86E-17
MSLN	rs11788059	9	106717987	C	T	0.34	0.07	0.01	7.89E-10
MSLN	rs35705950	11	1219991	T	G	0.11	0.07	0.01	2.52E-05
MSLN	rs2235503	16	760593	A	C	0.19	0.75	0.01	0.00E+00
MSLN	rs4807630	19	1170446	T	C	0.31	0.06	0.01	1.18E-06
MSLN	rs34450188	19	9420770	G	A	0.56	0.08	0.01	1.63E-13
MSLN	rs492602	19	48703160	G	A	0.51	0.07	0.01	1.24E-10
MSMB	rs4687657	3	52818522	T	G	0.26	0.09	0.01	5.34E-24
MSMB	rs62406520	6	36952853	G	A	0.10	-0.11	0.01	6.20E-15
MSMB	rs73671500	8	23608303	A	T	0.17	0.08	0.01	2.77E-15
MSMB	rs10993994	10	46046326	C	T	0.61	0.93	0.01	0.00E+00
MSMB	rs36120341	19	45721713	T	C	0.36	-0.05	0.01	1.27E-09
MSR1	rs41271951	1	150764744	G	A	0.09	-0.10	0.01	9.10E-08
MSR1	rs755799544	1	196721823	C	C	0.36	-0.05	0.01	2.73E-05
MSR1	rs547373038	3	99058488	T	G	0.49	-0.08	0.01	1.63E-12
MSR1	rs2293330	3	143385000	G	T	0.83	0.07	0.01	8.95E-07
MSR1	rs1471401	4	87853188	G	A	0.66	0.05	0.01	9.47E-06
MSR1	rs115216147	5	71369699	A	T	0.07	0.09	0.01	1.86E-05
MSR1	rs509227	6	10535358	C	A	0.52	0.05	0.01	2.21E-07
MSR1	rs139299944	6	32634888	T	C	0.34	-0.09	0.01	1.13E-18
MSR1	rs10863	7	130505748	G	A	0.85	0.07	0.01	9.52E-07
MSR1	rs41341748	8	16155085	A	G	0.01	-1.54	0.03	1.98E-206
MSR1	rs2031902	9	33117526	T	C	0.59	-0.06	0.01	1.47E-07
MSR1	-	9	133274293	A	C	0.18	0.07	0.01	4.98E-08
MSR1	rs11220505	11	126464910	T	C	0.22	0.14	0.01	2.81E-31
MSR1	rs77303550	16	72045758	T	C	0.20	-0.06	0.01	2.19E-05
MSR1	rs186021206	17	7166093	A	G	0.01	1.38	0.05	2.84E-89
MSRA	rs1354034	3	56815721	C	T	0.60	0.06	0.01	2.70E-07
MSRA	rs12546887	8	10435366	T	C	0.59	-0.06	0.01	1.31E-06
MSRA	-	10	63156270	A	T	0.49	0.06	0.01	2.28E-06
MSRA	rs139130389	11	72139110	A	C	0.07	-0.15	0.01	1.71E-12
MSTN	rs10489588	1	3035270	A	G	0.21	0.06	0.01	1.06E-05
MSTN	rs143242500	2	190060135	G	A	0.00	0.78	0.09	4.69E-09
MSTN	rs9838614	3	38496180	G	T	0.39	-0.05	0.01	1.74E-05
MSTN	rs199910997	6	32517878	G	A	0.56	0.05	0.01	1.21E-05
MSTN	rs34950020	6	133479407	A	G	0.32	-0.08	0.01	3.45E-11
MSTN	rs1815739	11	66560624	C	T	0.56	0.06	0.01	3.28E-08
MSTN	rs8096658	18	79396537	G	C	0.49	0.06	0.01	1.86E-07
MUC13	rs1127233	3	124908177	G	T	0.26	0.24	0.01	1.26E-78
MUC13	rs4556017	7	100989509	T	C	0.85	-0.10	0.01	1.15E-10

MUC13	rs2519093	9	133266456	T	C	0.18	-0.25	0.01	7.34E-66
MUC13	rs12019136	19	5835666	A	G	0.04	0.13	0.02	4.93E-06
MUC16	rs62193070	2	241791173	G	A	0.16	-0.26	0.01	3.92E-60
MUC16	rs7205754	16	765413	A	C	0.04	-0.20	0.02	1.18E-11
MUC16	rs10421318	19	9010243	A	G	0.35	-0.07	0.01	9.27E-09
MVK	rs28934897	12	109596515	A	G	0.00	-1.02	0.09	4.34E-13
MYO9B	rs11392202	19	17202369	T	A	0.40	-0.05	0.01	1.86E-05
MYOC	rs171003	1	171647381	A	G	0.58	0.27	0.01	7.58E-130
MYOC	-	2	218856229	T	G	0.06	0.13	0.02	2.38E-06
MYOC	rs309743	4	176495651	C	A	0.26	0.05	0.01	2.21E-05
MYOC	rs3812049	5	128083158	G	C	0.74	0.08	0.01	1.40E-11
MYOC	rs1474348	7	22728289	G	C	0.57	0.05	0.01	8.11E-06
MYOC	rs56278466	10	17833858	G	T	0.66	0.10	0.01	3.78E-20
MYOC	rs58473820	11	122643695	T	C	0.38	0.05	0.01	1.47E-05
MYOC	rs215225	12	481379	G	A	0.43	0.05	0.01	1.71E-05
MYOC	rs7310615	12	111427245	G	C	0.52	0.06	0.01	1.26E-07
MYOC	rs11632195	15	65201150	T	C	0.64	-0.09	0.01	6.12E-15
MYOC	rs186021206	17	7166093	A	G	0.01	0.52	0.05	7.20E-13
MYOC	rs61739916	19	18786630	C	T	0.06	0.15	0.02	3.91E-10
MZB1	rs149547076	3	57571717	C	T	0.01	-0.56	0.04	7.60E-24
MZB1	rs11739135	5	132397705	C	G	0.42	0.05	0.01	1.43E-05
MZB1	rs140248321	5	139390083	T	A	0.06	0.25	0.02	3.41E-22
MZB1	rs778224678	7	151256873	G	T	0.11	0.10	0.01	7.27E-09
MZB1	rs61839660	10	6052734	T	C	0.10	0.10	0.01	3.79E-07
MZB1	-	11	17247874	C	A	0.44	-0.06	0.01	7.89E-08
MZB1	rs3803286	14	102780133	G	A	0.67	-0.06	0.01	2.10E-07
MZB1	rs10150206	14	105740363	A	G	0.66	0.15	0.01	1.45E-37
MZB1	rs3803800	17	7559652	G	A	0.79	-0.09	0.01	1.54E-10
MZB1	rs34562254	17	16939677	A	G	0.10	0.15	0.01	3.62E-16
MZB1	rs9624334	22	23824069	C	G	0.15	-0.09	0.01	5.78E-08
MZT1	rs1354034	3	56815721	C	T	0.60	0.06	0.01	1.21E-06
MZT1	rs11101690	10	133308228	C	T	0.13	-0.08	0.01	1.94E-05
NAAA	rs5014539	1	172516613	T	G	0.57	0.05	0.01	6.97E-07
NAAA	rs687339	3	136213517	T	C	0.77	0.06	0.01	1.26E-06
NAAA	rs112197434	4	75932965	T	A	0.23	-0.67	0.01	0.00E+00
NAAA	rs114816312	4	109717668	T	C	0.01	0.29	0.04	1.01E-05
NAAA	-	4	118809663	T	C	0.06	-0.12	0.02	9.51E-07
NAAA	rs77945361	4	153333382	A	G	0.15	-0.08	0.01	4.83E-07
NAAA	rs4471106	9	5629133	A	G	0.29	-0.05	0.01	1.65E-05
NAAA	rs1800866	9	34637693	G	T	0.17	0.07	0.01	2.39E-08
NAAA	rs772851187	10	63147821	T	G	0.47	0.06	0.01	1.17E-09
NAAA	rs7904738	10	72950726	C	G	0.09	-0.09	0.01	2.12E-07
NAAA	rs2862954	10	100152307	C	T	0.50	-0.05	0.01	1.39E-06
NAAA	rs7096937	10	112190660	C	T	0.73	-0.06	0.01	1.51E-08
NAAA	rs10860794	12	101823942	A	C	0.29	-0.36	0.01	2.30E-233
NAAA	rs150641790	13	41113708	G	T	0.07	0.10	0.01	6.97E-08
NAAA	rs71435622	14	39242324	T	A	0.29	0.07	0.01	2.89E-09
NAAA	rs145078947	14	93186629	T	G	0.00	0.79	0.06	9.64E-17
NAAA	rs62073233	17	46311546	C	G	0.21	0.06	0.01	2.61E-05
NAAA	rs200210321	19	19283081	G	A	0.07	0.12	0.01	6.82E-11
NAAA	rs429358	19	44908684	C	T	0.16	-0.12	0.01	6.79E-18
NAAA	rs626283	19	54173307	C	G	0.44	0.06	0.01	1.11E-09
NAAA	rs738408	22	43928850	T	C	0.22	0.06	0.01	3.18E-06
NADK	rs17162854	1	1762287	G	A	0.52	0.25	0.01	1.99E-112
NADK	rs71309976	3	56694053	T	C	0.01	0.29	0.04	4.56E-07
NADK	rs389883	6	31979683	T	G	0.68	0.06	0.01	1.78E-06

NADK	rs56077246	8	143909325	C	T	0.43	0.08	0.01	7.06E-13
NADK	rs59581534	12	7438940	A	T	0.04	0.37	0.02	1.16E-42
NBL1	rs12408663	1	19641564	C	T	0.23	-0.12	0.01	2.59E-20
NBL1	rs1260326	2	27508073	C	T	0.61	0.06	0.01	1.90E-06
NBL1	rs36060036	16	20350628	T	C	0.17	-0.08	0.01	1.05E-07
NBN	rs1354034	3	56815721	C	T	0.60	0.06	0.01	1.29E-07
NBN	rs143331722	5	132595276	A	G	0.01	-0.31	0.04	2.32E-08
NBN	rs74378230	8	90031136	G	A	0.00	-0.61	0.06	4.89E-12
NCAM1	rs333947	1	109928142	A	G	0.15	-0.06	0.01	1.44E-05
NCAM1	rs2301455	1	172449128	T	C	0.56	0.06	0.01	8.26E-08
NCAM1	rs66824127	3	52517249	C	T	0.56	0.06	0.01	4.24E-08
NCAM1	rs28383282	6	32618598	T	C	0.21	0.06	0.01	7.57E-06
NCAM1	rs11214489	11	113105212	T	C	0.18	-0.33	0.01	4.83E-123
NCAM1	rs705702	12	55996852	G	A	0.34	0.06	0.01	6.17E-07
NCAM1	rs9318188	13	73536279	G	A	0.54	-0.11	0.01	1.59E-26
NCAM1	rs2035645	15	92403096	G	T	0.56	0.11	0.01	7.98E-24
NCAM1	rs776023876	17	39675044	C	A	0.69	0.09	0.01	1.59E-14
NCAM2	rs199922514	8	9325592	G	A	0.91	-0.08	0.01	1.94E-05
NCAM2	rs2071916	16	376476	C	T	0.59	-0.19	0.01	2.31E-80
NCAM2	rs186021206	17	7166093	A	G	0.01	0.42	0.04	1.01E-09
NCAM2	rs77542162	17	69085137	G	A	0.02	0.19	0.02	4.75E-08
NCAM2	rs4760	19	43648948	G	A	0.16	0.12	0.01	3.56E-17
NCAM2	-	21	21467382	T	A	0.35	0.54	0.01	0.00E+00
NCAM2	rs760715	22	39466338	C	T	0.25	0.07	0.01	9.43E-08
NCAN	rs61804160	1	161650299	A	T	0.14	0.06	0.01	2.45E-05
NCAN	rs6786821	3	141280434	G	C	0.62	0.10	0.01	1.18E-18
NCAN	rs114964313	5	79137749	A	G	0.32	0.05	0.01	2.50E-05
NCAN	rs6913309	6	32372063	A	T	0.28	0.05	0.01	1.95E-05
NCAN	rs12523793	6	149652628	A	G	0.33	0.08	0.01	1.30E-13
NCAN	-	8	115606807	C	A	0.56	0.07	0.01	4.88E-10
NCAN	-	10	63156270	A	T	0.49	0.05	0.01	3.61E-06
NCAN	-	10	72028381	C	A	0.56	0.08	0.01	1.03E-12
NCAN	rs12801188	11	47366663	A	G	0.36	0.06	0.01	1.88E-07
NCAN	rs3751198	12	103753429	G	A	0.60	0.05	0.01	1.29E-06
NCAN	rs60111444	15	101089333	G	A	0.23	0.07	0.01	2.03E-08
NCAN	rs879292	16	17371057	T	C	0.34	-0.07	0.01	2.96E-10
NCAN	rs117345300	17	44852391	G	C	0.04	0.12	0.02	2.20E-05
NCAN	rs6504649	17	50360095	G	C	0.39	-0.07	0.01	9.76E-10
NCAN	rs74178612	18	51101919	T	C	0.37	-0.06	0.01	2.59E-08
NCAN	rs3761077	19	19215154	T	G	0.11	-0.24	0.01	4.26E-45
NCF2	rs35012521	1	183563229	A	T	0.01	-0.93	0.04	4.32E-67
NCF2	-	7	75189161	T	G	0.10	0.09	0.01	1.19E-05
NCF2	rs149698681	15	42359702	C	G	0.02	0.21	0.02	7.93E-08
NCF2	rs12941811	17	40003082	C	T	0.57	-0.05	0.01	8.18E-07
NCF2	rs4632248	19	53821741	T	G	0.21	0.16	0.01	4.58E-32
NCK2	rs11170874	12	54332691	G	A	0.33	0.05	0.01	2.84E-05
NCLN	rs9268228	6	32319342	A	G	0.63	0.06	0.01	3.24E-06
NCR1	rs181807963	1	155004195	T	G	0.01	-0.31	0.04	3.71E-06
NCR1	rs6693836	1	185442174	A	G	0.64	0.07	0.01	2.42E-08
NCR1	rs7532674	1	207853394	T	G	0.27	0.07	0.01	6.02E-08
NCR1	rs76529516	2	60356235	G	A	0.03	0.15	0.02	1.04E-05
NCR1	rs2009581	2	111050100	A	G	0.27	-0.13	0.01	1.32E-24
NCR1	rs13104208	4	141749489	C	T	0.16	0.08	0.01	3.59E-08
NCR1	rs2248116	5	132468655	A	C	0.57	-0.06	0.01	3.64E-08
NCR1	rs9260316	6	29949137	T	G	0.57	-0.06	0.01	2.83E-08
NCR1	rs78565598	8	59182922	G	T	0.17	0.07	0.01	6.11E-06

NCR1	rs7086174	10	5974436	A	C	0.33	0.09	0.01	2.18E-13
NCR1	rs2787147	10	8419168	T	A	0.34	0.05	0.01	2.51E-05
NCR1	rs1250569	10	79285450	C	T	0.45	0.06	0.01	2.63E-08
NCR1	rs6586167	10	88996389	G	T	0.50	0.05	0.01	1.30E-06
NCR1	rs2497318	10	92672243	T	C	0.45	0.06	0.01	3.73E-08
NCR1	rs113430316	11	128246346	A	T	0.28	-0.05	0.01	2.54E-05
NCR1	rs3003	12	10431012	T	C	0.84	-0.09	0.01	7.52E-08
NCR1	rs3782473	12	51362485	C	T	0.24	0.08	0.01	6.79E-09
NCR1	rs3184504	12	111446804	C	T	0.52	-0.18	0.01	2.31E-63
NCR1	rs906932	14	99318673	C	T	0.62	0.08	0.01	3.07E-13
NCR1	-	16	20343260	T	A	0.18	-0.07	0.01	4.82E-06
NCR1	rs9916257	17	35470352	T	G	0.44	-0.08	0.01	2.17E-13
NCR1	rs4065275	17	39924612	G	A	0.50	0.07	0.01	3.42E-10
NCR1	rs6504199	17	64083022	A	C	0.25	-0.06	0.01	1.48E-05
NCR1	rs2659007	17	81243678	A	G	0.45	0.05	0.01	2.94E-06
NCR1	rs7255591	19	54904812	C	G	0.09	-0.43	0.01	7.48E-107
NCR1	rs6103572	20	44029222	C	T	0.73	0.07	0.01	1.39E-07
NCS1	rs66927685	2	169074756	A	T	0.53	-0.05	0.01	1.45E-06
NCS1	rs11134475	5	156972939	G	A	0.64	0.05	0.01	8.04E-06
NCS1	-	6	24429112	C	T	0.00	-0.99	0.05	1.23E-34
NCS1	rs662	7	95308134	C	T	0.29	0.06	0.01	2.15E-07
NCS1	rs12342201	9	93132682	A	G	0.48	-0.07	0.01	1.65E-11
NCS1	rs1054879	9	130233489	A	G	0.51	0.12	0.01	1.62E-29
NCS1	rs7902343	10	63245639	T	C	0.47	0.05	0.01	6.15E-06
NCS1	rs9807819	19	45736340	T	C	0.36	-0.05	0.01	6.20E-07
NCS1	rs2868346	20	45919331	T	C	0.76	0.05	0.01	7.55E-06
NECTIN2	rs61747728	1	179557079	T	C	0.04	0.18	0.02	7.35E-10
NECTIN2	rs553340154	6	32558001	G	T	0.33	0.07	0.01	5.73E-06
NECTIN2	-	9	133263362	G	A	0.18	-0.10	0.01	3.94E-11
NECTIN2	rs777834943	16	20355409	T	C	0.18	-0.08	0.01	9.17E-08
NECTIN2	rs1137844	19	35852177	G	C	0.32	0.06	0.01	5.76E-07
NECTIN2	rs440277	19	44857967	A	G	0.31	-0.18	0.01	1.90E-48
NECTIN2	rs679574	19	48702851	G	C	0.51	-0.16	0.01	4.25E-43
NECTIN4	rs35434391	1	161079719	T	G	0.12	-0.45	0.01	1.26E-143
NECTIN4	rs61747728	1	179557079	T	C	0.04	0.15	0.02	5.22E-07
NECTIN4	rs1260326	2	27508073	C	T	0.61	0.07	0.01	3.98E-09
NECTIN4	rs77784890	10	69439539	C	G	0.14	0.10	0.01	1.28E-09
NECTIN4	rs77924615	16	20381010	A	G	0.20	-0.08	0.01	1.46E-08
NECTIN4	rs3814995	19	35851310	T	C	0.31	0.06	0.01	4.80E-06
NEFL	rs4235739	5	148987008	C	T	0.61	0.04	0.01	2.60E-05
NEFL	rs7197999	16	75245022	T	C	0.52	0.04	0.01	1.26E-05
NELL1	rs10890482	1	47480761	C	T	0.81	0.08	0.01	3.42E-08
NELL1	rs2990223	1	155215184	G	A	0.61	0.06	0.01	3.91E-08
NELL1	rs4344946	2	119432144	G	A	0.86	-0.10	0.01	6.99E-11
NELL1	rs3903119	2	176127715	C	T	0.66	-0.06	0.01	2.35E-07
NELL1	-	3	186932142	C	G	0.62	0.07	0.01	1.33E-10
NELL1	rs13107325	4	102267552	T	C	0.08	0.18	0.01	7.27E-18
NELL1	rs9501590	6	31396607	G	A	0.34	0.05	0.01	5.76E-06
NELL1	rs2207136	6	50842007	C	T	0.47	-0.05	0.01	9.69E-07
NELL1	rs11458788	6	100505171	C	A	0.51	0.05	0.01	3.11E-06
NELL1	rs62435145	7	1246931	T	G	0.69	0.05	0.01	2.74E-05
NELL1	rs2339228	8	90722254	A	G	0.50	0.06	0.01	7.54E-09
NELL1	rs56278466	10	17833858	G	T	0.66	0.06	0.01	4.01E-08
NELL1	rs7917848	10	28705933	G	C	0.65	-0.10	0.01	1.62E-18
NELL1	rs61652119	11	20933724	A	G	0.05	0.79	0.02	1.99E-211
NELL1	rs542695580	11	30738234	T	C	0.37	0.07	0.01	9.27E-08

NELL1	rs375125008	11	65739571	C	T	0.38	-0.06	0.01	5.90E-08
NELL1	rs2298475	11	126408308	C	T	0.08	-0.16	0.01	2.10E-14
NELL1	rs7298766	12	552490	G	A	0.29	0.23	0.01	1.11E-86
NELL1	rs141826184	12	57184132	T	C	0.00	0.82	0.05	1.57E-24
NELL1	rs111279508	12	132533176	T	C	0.14	0.13	0.01	5.42E-16
NELL1	-	14	65602338	A	C	0.68	-0.08	0.01	1.33E-12
NELL1	rs4541022	15	93623083	T	C	0.88	-0.08	0.01	2.50E-06
NELL1	rs186021206	17	7166093	A	G	0.01	0.64	0.05	3.32E-18
NELL1	rs72835417	17	49164280	A	G	0.11	0.14	0.01	6.70E-16
NELL2	rs17285324	3	46441489	T	A	0.17	-0.07	0.01	1.00E-06
NELL2	rs4282054	3	52532049	C	T	0.56	0.07	0.01	3.83E-09
NELL2	rs201147962	3	58477962	T	C	0.61	0.05	0.01	2.20E-06
NELL2	rs55818129	5	39424990	G	T	0.12	-0.08	0.01	7.27E-06
NELL2	rs155625	5	72911035	G	T	0.32	-0.05	0.01	6.66E-06
NELL2	rs113239947	6	31494179	T	A	0.24	-0.07	0.01	1.40E-07
NELL2	rs7849148	9	33138354	T	A	0.31	0.06	0.01	1.65E-07
NELL2	rs2519093	9	133266456	T	C	0.18	-0.19	0.01	3.05E-44
NELL2	rs56278466	10	17833858	G	T	0.66	0.06	0.01	5.86E-08
NELL2	rs76485191	11	21170687	T	G	0.34	0.05	0.01	6.68E-06
NELL2	rs36027301	11	68041801	T	C	0.06	-0.12	0.02	1.86E-07
NELL2	rs142457851	12	44999589	A	C	0.60	-0.18	0.01	8.59E-57
NELL2	rs540730	12	57413331	C	T	0.75	-0.07	0.01	4.13E-07
NELL2	rs7137828	12	111494996	T	C	0.52	-0.05	0.01	2.18E-05
NELL2	rs186021206	17	7166093	A	G	0.01	0.43	0.05	1.58E-08
NELL2	rs4804790	19	7732268	T	G	0.07	-0.13	0.01	9.79E-10
NELL2	rs9616810	22	50656498	T	C	0.22	0.06	0.01	1.22E-05
NFASC	rs6693024	1	204976361	A	C	0.10	0.59	0.01	2.40E-239
NFASC	rs115478735	9	133274295	T	A	0.18	-0.09	0.01	6.62E-11
NFASC	rs56278466	10	17833858	G	T	0.66	0.17	0.01	9.95E-57
NFASC	rs58534292	14	21737747	C	T	0.65	0.05	0.01	7.45E-07
NFASC	rs12895651	14	106038037	C	T	0.70	0.09	0.01	8.22E-14
NFASC	rs3747207	22	43928975	A	G	0.22	0.06	0.01	5.93E-07
NFATC1	rs342293	7	106731773	G	C	0.46	-0.05	0.01	2.38E-05
NFATC1	rs657693	18	79402462	G	A	0.36	-0.11	0.01	1.32E-22
NFATC3	rs74661798	6	32501898	A	T	0.16	0.13	0.01	3.42E-13
NFATC3	rs13263709	8	80374940	C	T	0.65	0.06	0.01	1.13E-06
NFATC3	-	19	1489737	G	C	0.06	-0.14	0.02	1.40E-08
NFKBIE	rs1354034	3	56815721	C	T	0.60	0.06	0.01	1.20E-06
NFKBIE	rs28362856	6	44266802	C	G	0.04	-0.52	0.02	6.16E-78
NID1	rs41267433	1	156904629	G	C	0.11	0.08	0.01	6.12E-06
NID1	rs76183323	1	236067017	A	G	0.01	-0.76	0.04	3.59E-39
NID1	rs13412535	2	224010157	A	G	0.23	-0.07	0.01	2.26E-07
NID1	rs11719450	3	58471084	T	C	0.37	0.06	0.01	4.12E-08
NID1	rs34716199	5	72897488	T	A	0.54	0.06	0.01	3.28E-07
NID1	rs4098923	6	31225979	G	A	0.45	-0.06	0.01	3.97E-08
NID1	rs342299	7	106733272	T	C	0.46	-0.05	0.01	2.49E-06
NID1	rs4734879	8	105570896	G	A	0.27	-0.06	0.01	4.65E-06
NID1	rs927826	10	19931917	T	G	0.71	0.18	0.01	1.09E-50
NID1	rs10761731	10	63267850	T	A	0.41	0.09	0.01	5.43E-15
NID1	rs12445050	16	81837364	T	C	0.14	0.09	0.01	1.17E-08
NID1	rs892090	19	55027704	G	T	0.83	0.13	0.01	8.91E-18
NID2	rs13412535	2	224010157	A	G	0.23	-0.06	0.01	2.03E-06
NID2	rs7618405	3	18209017	A	C	0.20	-0.06	0.01	2.12E-05
NID2	rs1354034	3	56815721	C	T	0.60	-0.06	0.01	1.85E-07
NID2	rs755492124	4	101856385	T	G	0.39	-0.06	0.01	4.52E-08
NID2	rs274555	5	132387259	T	C	0.40	0.05	0.01	2.71E-06

NID2	rs35173808	6	31358371	A	C	0.10	-0.10	0.01	1.10E-07
NID2	rs1917342	6	70640051	G	A	0.30	-0.05	0.01	4.41E-06
NID2	rs6961069	7	80589645	T	C	0.40	0.06	0.01	2.93E-08
NID2	rs6993770	8	105569300	T	A	0.29	-0.10	0.01	3.67E-17
NID2	rs10820606	9	96430637	C	A	0.23	-0.08	0.01	2.58E-09
NID2	rs10740118	10	63341447	C	G	0.41	0.09	0.01	2.44E-16
NID2	rs12767683	10	102556824	A	C	0.32	0.06	0.01	7.69E-07
NID2	rs35277580	12	6185368	A	G	0.49	-0.05	0.01	2.54E-06
NID2	rs2516600	14	52021048	C	T	0.54	0.57	0.01	0.00E+00
NID2	rs59001897	15	64868193	A	T	0.17	0.07	0.01	2.34E-06
NID2	rs12445050	16	81837364	T	C	0.14	0.10	0.01	1.64E-11
NID2	rs892090	19	55027704	G	T	0.83	0.15	0.01	3.29E-25
NID2	rs3827978	20	19300647	T	C	0.35	0.07	0.01	6.58E-10
NINJ1	rs9274442	6	32665454	C	G	0.28	-0.08	0.01	4.47E-09
NINJ1	rs11379524	9	93120463	C	A	0.63	0.24	0.01	8.74E-90
NME3	rs188468174	1	24965206	T	C	0.01	-0.35	0.03	2.54E-13
NME3	rs57008601	2	203110811	G	T	0.57	-0.05	0.01	2.23E-06
NME3	rs796157456	4	39636708	A	C	0.50	0.05	0.01	9.19E-06
NME3	rs34230118	5	96939216	T	C	0.42	0.08	0.01	5.13E-12
NME3	rs1014651	9	100214362	A	G	0.53	0.05	0.01	1.14E-05
NME3	rs2519093	9	133266456	T	C	0.18	-0.11	0.01	3.49E-15
NME3	rs10842845	12	9085616	C	G	0.35	-0.25	0.01	9.18E-104
NME3	rs2278093	12	29381276	A	C	0.29	-0.06	0.01	6.73E-06
NME3	rs12424638	12	48925017	A	G	0.08	-0.12	0.01	7.11E-08
NME3	rs55707100	15	43528519	T	C	0.02	-0.18	0.02	1.52E-06
NME3	rs749285440	16	1718075	A	G	0.00	3.07	0.11	4.78E-80
NME3	rs1801689	17	66214462	C	A	0.03	0.16	0.02	1.98E-06
NME3	rs140664781	19	3199725	G	C	0.44	0.10	0.01	2.83E-16
NME3	rs7599	19	35547488	G	A	0.63	-0.15	0.01	3.53E-39
NME3	rs12975366	19	54255498	C	T	0.39	-0.08	0.01	1.19E-11
NME3	rs5749077	22	30364829	C	A	0.23	0.06	0.01	7.41E-06
NMNAT1	rs180754466	1	9960737	A	C	0.01	0.67	0.04	8.00E-30
NMNAT1	rs9271573	6	32622724	C	A	0.60	0.06	0.01	8.73E-07
NMNAT1	rs58534292	14	21737747	C	T	0.65	-0.09	0.01	8.94E-14
NMNAT1	rs111266381	14	105998918	T	C	0.15	-0.19	0.01	3.22E-36
NMNAT1	rs704	17	28367840	A	G	0.47	-0.20	0.01	6.27E-77
NOMO1	rs111779744	5	180809824	A	C	0.15	0.08	0.01	4.13E-08
NOMO1	rs174549	11	61803910	A	G	0.31	0.07	0.01	4.38E-10
NOMO1	rs28929474	14	94378610	T	C	0.02	0.39	0.02	3.69E-26
NOMO1	rs3891245	16	16254430	G	T	0.23	-0.51	0.01	0.00E+00
NOMO1	rs1801689	17	66214462	C	A	0.03	0.18	0.02	7.81E-09
NOS1	rs12574283	11	22233940	A	G	0.71	0.07	0.01	3.03E-08
NOS1	rs10651018	12	117459359	G	A	0.46	-0.11	0.01	2.44E-25
NOS3	rs7572209	2	85862949	C	T	0.20	-0.08	0.01	1.51E-08
NOS3	rs6415128	6	32363117	A	G	0.56	0.05	0.01	1.15E-05
NOS3	-	9	133266234	C	A	0.34	0.07	0.01	8.08E-08
NOS3	rs189448562	20	3724468	C	G	0.01	0.24	0.04	2.31E-05
NOTCH1	rs1260326	2	27508073	C	T	0.61	0.06	0.01	8.42E-07
NOTCH1	rs4240624	8	9326721	A	G	0.91	-0.10	0.01	1.49E-07
NOTCH1	rs10793962	9	133253728	T	A	0.06	0.52	0.02	1.32E-113
NOTCH1	rs182330532	9	136506782	A	G	0.00	1.22	0.11	1.06E-13
NOTCH1	rs56278466	10	17833858	G	T	0.66	0.08	0.01	3.56E-11
NOTCH1	rs34434834	11	126437901	A	G	0.03	0.14	0.02	1.75E-05
NOTCH1	rs111338191	12	111388673	T	A	0.52	-0.05	0.01	3.80E-06
NOTCH1	rs186021206	17	7166093	A	G	0.01	0.63	0.05	1.14E-16
NOTCH3	rs61801010	1	161572445	G	A	0.11	0.10	0.01	7.47E-08

NOTCH3	rs1260326	2	27508073	C	T	0.61	0.07	0.01	8.70E-10
NOTCH3	rs10282122	7	2489989	T	C	0.67	0.07	0.01	3.65E-09
NOTCH3	rs774272255	7	95307072	T	A	0.28	0.06	0.01	1.48E-06
NOTCH3	rs507666	9	133273983	A	G	0.18	-0.07	0.01	1.59E-07
NOTCH3	rs12359178	10	17847614	A	G	0.14	0.08	0.01	4.41E-07
NOTCH3	-	12	57001714	G	A	0.08	0.11	0.01	3.03E-09
NOTCH3	rs17580	14	94380925	A	T	0.05	-0.15	0.02	3.96E-09
NOTCH3	rs139974673	15	43735687	C	T	0.02	-0.21	0.02	2.32E-09
NOTCH3	rs186021206	17	7166093	A	G	0.01	0.48	0.05	7.96E-11
NOTCH3	rs77542162	17	69085137	G	A	0.02	0.19	0.02	1.84E-07
NOTCH3	rs539420095	19	15417974	T	C	0.00	-1.17	0.06	1.05E-34
NOTCH3	rs28616221	19	35063575	A	G	0.18	-0.06	0.01	5.84E-06
NPDC1	rs13146355	4	76490987	A	G	0.46	0.05	0.01	4.46E-06
NPDC1	rs10265221	7	151717243	C	T	0.29	0.06	0.01	7.03E-07
NPDC1	-	9	137059328	C	G	0.90	0.18	0.01	2.16E-22
NPDC1	rs34749116	12	130796207	A	G	0.33	0.05	0.01	1.61E-05
NPDC1	rs548328000	14	91830655	A	C	0.58	-0.06	0.01	1.58E-08
NPDC1	rs36060036	16	20350628	T	C	0.17	-0.09	0.01	7.96E-09
NPDC1	rs11672900	19	48717066	G	A	0.54	0.06	0.01	8.75E-07
NPPB	rs198389	1	11859214	G	A	0.42	0.18	0.01	2.85E-56
NPPB	rs2234962	10	119670121	C	T	0.22	0.07	0.01	8.66E-07
NPPC	rs77806605	2	49134522	G	C	0.27	0.05	0.01	1.44E-05
NPPC	rs4859682	4	76489165	A	C	0.46	0.05	0.01	2.73E-06
NPPC	rs113878851	16	20353760	T	C	0.17	-0.06	0.01	1.65E-05
NPTX1	rs78265569	1	2214726	A	C	0.09	0.10	0.01	7.42E-09
NPTX1	-	1	27373179	A	G	0.01	-0.33	0.03	6.74E-15
NPTX1	rs12025349	1	213767438	T	C	0.60	0.10	0.01	1.77E-21
NPTX1	rs13064675	3	24188797	C	T	0.20	-0.06	0.01	3.13E-06
NPTX1	rs4693802	4	87058060	G	A	0.25	-0.06	0.01	8.67E-08
NPTX1	rs66833621	6	6923601	C	T	0.19	0.12	0.01	1.93E-20
NPTX1	rs16896742	6	29954963	G	A	0.36	-0.24	0.01	1.36E-116
NPTX1	rs10986338	9	124428953	A	G	0.65	0.05	0.01	5.68E-06
NPTX1	rs2519093	9	133266456	T	C	0.18	-0.10	0.01	1.22E-14
NPTX1	rs10786548	10	99496569	A	G	0.89	0.07	0.01	9.33E-06
NPTX1	rs7937122	11	126354553	G	C	0.39	0.08	0.01	3.74E-14
NPTX1	rs186021206	17	7166093	A	G	0.01	0.71	0.05	2.10E-21
NPTX1	rs62068268	17	80565290	T	C	0.09	0.82	0.01	0.00E+00
NPTX1	rs4806469	19	55186877	A	G	0.19	0.06	0.01	2.80E-06
NPTX1	rs7280064	21	15005329	T	C	0.15	0.07	0.01	9.85E-07
NPTXR	rs9273211	6	32645903	A	G	0.23	-0.06	0.01	2.84E-06
NPTXR	rs61007246	7	98654503	T	C	0.71	0.09	0.01	6.56E-16
NPTXR	rs12216891	9	133251979	T	C	0.06	0.10	0.01	2.36E-05
NPTXR	rs369705328	10	17806193	C	G	0.13	0.09	0.01	2.46E-08
NPTXR	rs186021206	17	7166093	A	G	0.01	0.31	0.05	1.63E-05
NPTXR	rs111577133	22	38845257	G	A	0.02	2.00	0.03	0.00E+00
NPY	rs765974791	1	62541132	T	C	0.64	0.07	0.01	1.36E-08
NPY	rs1499899	3	113192413	A	G	0.49	-0.05	0.01	2.11E-05
NPY	rs5030062	3	186736391	C	A	0.37	-0.10	0.01	2.00E-19
NPY	rs4861708	4	186236079	A	G	0.51	-0.19	0.01	6.41E-65
NPY	rs639933	5	135132061	A	C	0.63	0.06	0.01	2.44E-06
NPY	rs1801020	5	177409531	G	A	0.74	-0.09	0.01	1.87E-11
NPY	rs34713112	6	32640812	A	T	0.65	0.06	0.01	1.88E-06
NPY	rs56393506	6	160668275	T	C	0.17	0.07	0.01	8.47E-07
NPY	rs9718966	7	506159	C	G	0.51	0.06	0.01	6.94E-07
NPY	rs4722337	7	24269479	T	A	0.55	-0.16	0.01	1.61E-46
NPY	rs33951980	7	73615107	T	C	0.13	-0.08	0.01	1.98E-06

NPY	rs76722925	8	20073987	G	A	0.08	-0.09	0.01	1.95E-05
NPY	rs200235517	8	116618774	G	A	0.08	0.17	0.01	1.10E-17
NPY	-	8	125492141	C	T	0.60	-0.08	0.01	7.98E-10
NPY	-	11	116752497	T	A	0.86	-0.13	0.01	1.30E-15
NPY	rs1809844	16	86154405	T	C	0.50	-0.06	0.01	8.19E-07
NPY	rs77542162	17	69085137	G	A	0.02	0.30	0.02	6.57E-16
NPY	rs72654473	19	44911142	A	C	0.11	0.15	0.01	8.18E-17
NRCAM	rs61804210	1	161701037	T	C	0.10	0.10	0.01	4.05E-08
NRCAM	rs1260326	2	27508073	C	T	0.61	0.07	0.01	4.22E-09
NRCAM	rs34656207	6	32614824	T	C	0.36	0.06	0.01	1.61E-06
NRCAM	rs138528835	7	108346905	A	T	0.31	0.29	0.01	7.53E-127
NRCAM	rs532436	9	133274414	A	G	0.19	-0.11	0.01	4.20E-14
NRCAM	rs56278466	10	17833858	G	T	0.66	0.14	0.01	2.92E-32
NRCAM	rs1265564	12	111270654	C	A	0.44	0.05	0.01	5.17E-06
NRCAM	rs186021206	17	7166093	A	G	0.01	0.38	0.05	8.19E-07
NRP1	rs777231403	1	62634374	A	G	0.35	0.09	0.01	7.16E-16
NRP1	rs764992666	1	230159329	T	C	0.60	0.21	0.01	3.38E-81
NRP1	rs2090034	2	20172648	A	G	0.47	-0.05	0.01	1.91E-06
NRP1	rs1260326	2	27508073	C	T	0.61	0.07	0.01	3.40E-09
NRP1	rs35332062	7	73597712	A	G	0.13	0.09	0.01	1.73E-07
NRP1	rs2506150	10	33194380	A	G	0.36	-0.30	0.01	1.02E-151
NRP1	-	11	116752497	T	A	0.86	-0.10	0.01	6.21E-11
NRP1	rs186021206	17	7166093	A	G	0.01	0.60	0.05	2.21E-15
NRP1	rs77542162	17	69085137	G	A	0.02	0.21	0.02	1.29E-08
NRP2	rs12995151	2	62375303	G	A	0.20	0.06	0.01	1.59E-05
NRP2	rs2289023	2	205121597	G	A	0.23	-0.20	0.01	3.24E-51
NRP2	rs17285324	3	46441489	T	A	0.17	-0.08	0.01	2.75E-07
NRP2	rs13081028	3	52521300	A	G	0.56	0.06	0.01	5.85E-07
NRP2	rs55818129	5	39424990	G	T	0.12	-0.07	0.01	2.13E-05
NRP2	rs3132485	6	31275612	A	C	0.50	-0.07	0.01	6.72E-11
NRP2	rs949451	7	17818931	G	A	0.51	-0.06	0.01	3.46E-08
NRP2	-	11	44570719	T	A	0.78	-0.06	0.01	7.52E-06
NRP2	rs2137537	12	70719307	C	T	0.55	-0.05	0.01	2.67E-05
NRP2	rs1070073	12	103606541	G	T	0.67	0.08	0.01	5.94E-11
NRTN	rs36055559	19	5799422	A	G	0.17	-0.07	0.01	7.07E-06
NSFL1C	rs1354034	3	56815721	C	T	0.60	0.06	0.01	2.72E-08
NSFL1C	rs6033860	20	1467930	G	A	0.28	-0.09	0.01	1.48E-12
NT5C3A	rs1354034	3	56815721	C	T	0.60	0.06	0.01	1.31E-06
NT5C3A	rs17622656	5	132485305	A	G	0.39	-0.05	0.01	1.65E-05
NT5C3A	rs10252880	7	33071047	C	G	0.33	-0.11	0.01	5.64E-19
NT5C3A	rs6993770	8	105569300	T	A	0.29	-0.06	0.01	3.88E-07
NT5C3A	-	10	63156270	A	T	0.49	0.07	0.01	7.46E-09
NT5E	rs1497406	1	16178825	G	A	0.58	0.06	0.01	3.65E-09
NT5E	rs7429191	3	149395293	A	G	0.71	0.05	0.01	1.59E-06
NT5E	rs13107325	4	102267552	T	C	0.08	-0.09	0.01	1.61E-06
NT5E	rs4835265	4	145900258	A	C	0.16	0.12	0.01	1.56E-17
NT5E	rs10075805	5	31021251	G	A	0.28	-0.06	0.01	1.36E-06
NT5E	rs2229523	6	85489515	G	A	0.66	-0.59	0.01	0.00E+00
NT5E	rs174557	11	61813896	G	A	0.31	0.06	0.01	3.66E-08
NT5E	rs550999044	14	103101069	T	A	0.24	0.11	0.01	1.02E-21
NT5E	rs12373325	18	58420416	C	T	0.80	-0.08	0.01	5.27E-10
NT5E	rs601338	19	48703417	A	G	0.51	0.06	0.01	4.38E-08
NT5E	rs189448562	20	3724468	C	G	0.01	0.33	0.03	2.23E-11
NT5E	rs6076335	20	25334228	G	T	0.48	0.05	0.01	1.46E-06
NTF3	rs13402475	2	3592319	G	C	0.82	-0.08	0.01	4.65E-08
NTF3	rs10868235	9	84878840	T	C	0.53	-0.08	0.01	1.77E-13

NTF3	rs73039984	12	5440290	T	C	0.11	-0.23	0.01	1.42E-37
NTF3	rs117126605	15	88248996	C	T	0.02	-0.36	0.03	4.88E-17
NTF3	rs11151953	18	74456625	C	G	0.53	0.09	0.01	5.27E-14
NTF4	rs8002440	13	73617497	T	C	0.49	-0.05	0.01	2.02E-05
NTproBNP	rs198389	1	11859214	G	A	0.42	0.26	0.01	3.23E-131
NTproBNP	rs3800560	7	128821040	T	C	0.08	0.08	0.01	1.13E-05
NTproBNP	rs72671655	8	105335669	A	T	0.04	0.14	0.02	1.98E-07
NTproBNP	rs2234962	10	119670121	C	T	0.22	0.09	0.01	2.75E-12
NTRK2	rs4665972	2	27375230	C	T	0.61	0.06	0.01	3.27E-07
NTRK2	rs13107325	4	102267552	T	C	0.08	0.10	0.01	2.24E-06
NTRK2	rs77562929	9	84670452	T	G	0.01	0.47	0.05	5.21E-11
NTRK2	rs56278466	10	17833858	G	T	0.66	0.08	0.01	1.44E-12
NTRK2	rs2298475	11	126408308	C	T	0.07	-0.10	0.01	1.32E-06
NTRK2	rs186021206	17	7166093	A	G	0.01	0.52	0.05	1.82E-11
NTRK2	rs2008174	22	39464125	T	C	0.74	-0.07	0.01	6.04E-07
NTRK3	rs34324830	1	92622298	T	C	0.11	-0.09	0.01	6.99E-08
NTRK3	rs7340151	2	9525321	T	C	0.67	0.06	0.01	3.47E-06
NTRK3	rs35887873	11	126349501	C	T	0.23	0.06	0.01	5.50E-06
NTRK3	rs10777159	12	89385567	G	A	0.30	-0.05	0.01	2.75E-05
NTRK3	rs3184504	12	111446804	C	T	0.52	0.08	0.01	2.31E-12
NTRK3	rs79445108	14	103726687	A	G	0.07	-0.16	0.01	2.71E-13
NTRK3	rs28735437	15	87971624	C	G	0.14	0.27	0.01	2.29E-65
NTRK3	rs200489612	17	7203059	A	G	0.01	0.53	0.05	8.18E-11
NTRK3	rs914187	21	41247822	C	T	0.60	-0.11	0.01	8.23E-23
NTRK3	rs2413593	22	39476122	G	A	0.49	0.05	0.01	4.85E-06
NUB1	rs1354034	3	56815721	C	T	0.60	0.08	0.01	1.61E-12
NUB1	rs342298	7	106733200	T	C	0.46	-0.06	0.01	2.25E-06
NUB1	rs113920358	7	151341801	T	C	0.13	-0.27	0.01	1.19E-52
NUB1	rs3847326	10	63572099	G	A	0.48	-0.05	0.01	1.58E-05
NUB1	rs11502185	11	180258	C	T	0.26	0.07	0.01	1.37E-06
NUCB2	rs2524096	6	31268690	T	G	0.42	0.05	0.01	2.28E-05
NUCB2	rs6993770	8	105569300	T	A	0.29	-0.08	0.01	3.89E-10
NUCB2	rs214068	11	17284103	C	T	0.58	-0.40	0.01	6.61E-278
NUCB2	rs3184504	12	111446804	C	T	0.52	-0.05	0.01	4.41E-06
NUCB2	rs892090	19	55027704	G	T	0.83	0.08	0.01	7.35E-08
NUDC	rs1354034	3	56815721	C	T	0.60	0.08	0.01	2.69E-11
NUDC	rs342298	7	106733200	T	C	0.46	-0.06	0.01	6.92E-08
NUDT2	rs1354034	3	56815721	C	T	0.60	0.05	0.01	1.36E-05
NUDT2	rs10972064	9	34336683	T	A	0.15	0.33	0.01	1.46E-99
NUDT2	-	10	63156270	A	T	0.49	0.06	0.01	2.40E-07
NUDT5	rs1354034	3	56815721	C	T	0.60	0.07	0.01	1.54E-08
NUDT5	rs10508438	10	12202080	A	G	0.34	0.06	0.01	7.20E-07
NUDT5	rs60822569	12	54323724	C	T	0.55	0.06	0.01	1.02E-06
OBP2B	rs144623173	1	9202282	A	T	0.41	-0.05	0.01	5.89E-11
OBP2B	rs61685000	1	202212057	C	G	0.41	-0.04	0.01	8.02E-06
OBP2B	rs12621139	2	118875148	A	G	0.18	0.07	0.01	3.26E-11
OBP2B	-	5	2485913	C	G	0.40	0.04	0.01	7.80E-06
OBP2B	rs34320230	7	98156188	T	C	0.54	-0.06	0.01	7.87E-14
OBP2B	rs1825146	8	32228161	C	A	0.57	-0.05	0.01	1.36E-08
OBP2B	rs4962104	9	133211660	C	T	0.80	0.70	0.01	0.00E+00
OBP2B	rs11202728	10	88350500	G	C	0.29	0.04	0.01	1.88E-06
OBP2B	rs7305932	12	7528796	A	T	0.78	-0.05	0.01	4.43E-06
OBP2B	rs9543287	13	73240304	G	C	0.32	-0.05	0.01	1.86E-08
OBP2B	rs8047587	16	53764710	T	G	0.44	-0.04	0.01	9.26E-07
OBP2B	rs2304195	19	45059613	T	C	0.52	0.04	0.01	1.65E-05
OBP2B	rs12986064	19	54251270	C	T	0.51	-0.05	0.01	2.20E-08

OBP2B	rs11911496	21	39677545	G	A	0.49	-0.04	0.01	9.00E-07
OBP2B	rs1978060	22	19762002	G	A	0.61	0.04	0.01	9.21E-07
ODAM	rs76153614	4	70195601	C	T	0.05	-0.58	0.02	5.80E-110
ODAM	rs28544078	4	111864198	T	G	0.24	-0.06	0.01	3.90E-06
OGFR	rs6993770	8	105569300	T	A	0.29	-0.06	0.01	7.90E-06
OGFR	rs200108407	20	62812457	A	G	0.00	-1.72	0.10	1.97E-28
OGN	rs13146355	4	76490987	A	G	0.46	0.04	0.01	9.93E-06
OGN	rs7026361	9	92376282	T	G	0.37	0.59	0.01	0.00E+00
OGN	rs145678014	11	32906232	T	G	0.04	-0.11	0.02	2.08E-06
OGN	rs28640218	16	20347945	T	G	0.19	-0.06	0.01	6.55E-07
OLR1	rs61740470	3	46450495	T	C	0.01	0.28	0.04	7.91E-07
OLR1	rs2871960	3	141402972	C	A	0.45	-0.10	0.01	2.36E-18
OLR1	rs56388170	7	28684757	T	G	0.29	0.06	0.01	1.39E-05
OLR1	-	11	47280489	T	A	0.11	-0.16	0.01	2.58E-19
OLR1	rs2511241	11	73234296	T	C	0.93	0.21	0.01	1.67E-20
OLR1	rs587862	11	128725278	G	A	0.27	0.06	0.01	1.34E-05
OLR1	rs936964	12	10139536	A	C	0.28	0.11	0.01	1.60E-16
OLR1	rs11871747	17	40021029	C	T	0.39	0.07	0.01	2.71E-09
OMD	rs13081028	3	52521300	A	G	0.56	0.10	0.01	4.94E-19
OMD	rs1386625	4	38359499	G	A	0.90	-0.08	0.01	2.36E-05
OMD	rs4253281	4	186243195	A	G	0.51	-0.06	0.01	9.86E-08
OMD	-	8	119033245	C	T	0.51	0.07	0.01	2.38E-09
OMD	rs35209758	9	92225574	T	A	0.31	0.08	0.01	1.08E-10
OMD	rs117801489	12	104015054	C	T	0.02	-0.45	0.03	2.87E-27
OMD	rs548589580	13	42399147	A	T	0.48	0.06	0.01	2.72E-08
OMD	rs704	17	28367840	A	G	0.47	-0.09	0.01	4.12E-16
OMD	rs738408	22	43928850	T	C	0.22	0.06	0.01	7.04E-06
OMG	rs112168180	5	127059245	T	C	0.03	-0.17	0.02	1.11E-06
OMG	rs3134986	6	32676776	T	C	0.29	-0.07	0.01	6.51E-07
OMG	rs3757724	7	20355444	T	C	0.06	0.18	0.02	4.34E-15
OMG	rs35107030	7	73536765	G	C	0.04	0.18	0.02	1.13E-10
OMG	rs56278466	10	17833858	G	T	0.66	0.11	0.01	9.56E-22
OMG	rs200489612	17	7203059	A	G	0.01	0.52	0.05	3.21E-10
OMG	rs72813607	17	31134507	A	G	0.14	-0.13	0.01	4.11E-15
OMG	rs17196752	20	50270731	T	C	0.19	-0.07	0.01	2.66E-06
OPTC	rs139024490	1	203499664	C	T	0.00	-1.71	0.05	2.97E-97
OPTC	rs78011579	2	88398520	A	G	0.03	-0.25	0.02	3.44E-13
OPTC	rs10212330	3	25477342	T	A	0.77	-0.06	0.01	2.33E-05
OPTC	rs67449961	6	32529773	C	G	0.82	0.09	0.01	1.84E-09
OPTC	rs2108790	7	7215848	T	C	0.61	0.05	0.01	8.20E-06
OPTC	-	8	134250701	C	T	0.06	0.18	0.01	2.17E-15
OPTC	rs55851518	9	76662648	A	G	0.06	-0.16	0.02	1.90E-11
OPTC	rs10750291	11	124836729	A	G	0.32	-0.08	0.01	2.13E-10
OPTC	rs35320790	14	60642107	A	C	0.60	-0.06	0.01	7.84E-07
OPTC	rs35731193	14	106050118	A	G	0.44	-0.05	0.01	6.71E-06
OPTC	rs186021206	17	7166093	A	G	0.01	0.65	0.05	8.59E-18
OSCAR	-	2	118955049	G	A	0.17	0.07	0.01	3.33E-07
OSCAR	rs13063578	3	47046347	A	T	0.40	-0.05	0.01	6.14E-06
OSCAR	rs73401939	6	29772569	G	A	0.01	0.62	0.04	2.07E-21
OSCAR	rs4407843	8	129597122	C	A	0.57	-0.06	0.01	2.50E-09
OSCAR	rs7137828	12	111494996	T	C	0.52	-0.08	0.01	3.96E-17
OSCAR	rs76428106	13	28029870	C	T	0.01	0.33	0.03	2.76E-13
OSCAR	rs186021206	17	7166093	A	G	0.01	0.62	0.04	3.95E-20
OSCAR	rs2305482	17	39984674	C	A	0.54	-0.05	0.01	5.52E-06
OSCAR	rs2659005	17	81244914	T	C	0.44	0.04	0.01	1.83E-05
OSCAR	rs4442925	19	54051696	T	C	0.45	0.52	0.01	0.00E+00

OSM	rs28498283	2	43132926	T	A	0.26	0.07	0.01	7.82E-07
OSM	rs9325122	5	148823373	T	C	0.55	-0.05	0.01	1.00E-05
OSM	-	6	31448099	T	G	0.30	0.07	0.01	1.06E-08
OSM	rs34433840	10	934476	T	C	0.24	0.06	0.01	5.32E-06
OSM	rs2511241	11	73234296	T	C	0.93	0.36	0.01	1.75E-61
OSM	rs7310615	12	111427245	G	C	0.52	-0.07	0.01	6.54E-09
OSM	rs2289511	14	87988566	A	G	0.58	-0.06	0.01	4.45E-07
OSM	rs11871747	17	40021029	C	T	0.39	0.13	0.01	7.52E-29
OSM	rs4823082	22	30204116	C	G	0.02	-0.19	0.02	1.18E-06
OSMR	rs2228145	1	154454494	C	A	0.41	-0.05	0.01	6.28E-07
OSMR	rs9292723	5	38852470	T	C	0.28	-0.59	0.01	0.00E+00
OSMR	rs8176693	9	133262254	T	C	0.06	0.10	0.01	5.48E-07
OSMR	rs776516693	11	126371957	T	G	0.13	-0.08	0.01	1.69E-08
OSMR	rs56187480	15	63497280	A	G	0.34	0.06	0.01	4.92E-10
OSMR	rs186021206	17	7166093	A	G	0.01	0.60	0.04	4.13E-19
OXT	rs10929757	2	11562535	C	A	0.58	0.06	0.01	2.38E-07
OXT	rs11127048	2	27529596	A	G	0.62	-0.06	0.01	7.45E-07
OXT	rs4895864	6	130043337	T	C	0.57	0.05	0.01	2.85E-06
OXT	rs35332062	7	73597712	A	G	0.13	-0.10	0.01	3.41E-10
OXT	rs45446698	7	99735325	G	T	0.04	-0.12	0.02	5.51E-06
OXT	rs112875651	8	125494452	A	G	0.39	-0.07	0.01	1.89E-09
OXT	rs2068888	10	93079885	A	G	0.45	-0.05	0.01	1.07E-05
OXT	rs4782568	16	83946924	G	C	0.45	0.06	0.01	2.78E-07
OXT	rs877172	20	3069244	G	T	0.31	0.62	0.01	0.00E+00
OXT	rs1800961	20	44413724	T	C	0.03	-0.14	0.02	1.13E-05
P4HB	rs3910516	2	215438330	G	A	0.74	0.07	0.01	7.39E-07
P4HB	rs1354034	3	56815721	C	T	0.60	0.06	0.01	7.77E-07
P4HB	-	7	106727342	C	T	0.46	-0.05	0.01	6.50E-06
P4HB	rs112635299	14	94371805	T	G	0.02	0.19	0.03	1.55E-06
P4HB	rs113708033	17	81878054	T	G	0.19	0.08	0.01	1.45E-08
PADI2	rs2235913	1	17098582	A	G	0.62	0.12	0.01	3.02E-25
PADI4	rs2240336	1	17347907	T	C	0.41	-0.26	0.01	2.32E-110
PAEP	rs1130866	2	85666618	A	G	0.51	-0.04	0.01	2.72E-05
PAEP	rs9409964	9	135580082	A	G	0.19	0.87	0.01	0.00E+00
PAEP	rs4805162	19	36183403	G	A	0.52	-0.05	0.01	1.16E-08
PAG1	rs1354034	3	56815721	C	T	0.60	0.05	0.01	8.06E-06
PAG1	rs4436084	8	81125771	C	T	0.35	-0.08	0.01	6.80E-10
PAG1	rs7310615	12	111427245	G	C	0.52	-0.08	0.01	4.65E-11
PAG1	rs1532085	15	58391167	G	A	0.62	0.06	0.01	1.09E-06
PAG1	rs247617	16	56956804	A	C	0.32	-0.08	0.01	1.85E-10
PAG1	rs7208422	17	78134494	T	A	0.48	-0.08	0.01	5.82E-11
PAK4	rs1354034	3	56815721	C	T	0.60	0.05	0.01	1.82E-05
PAK4	rs60822569	12	54323724	C	T	0.55	0.06	0.01	2.55E-06
PAM	rs149802978	5	103011162	G	C	0.05	-1.09	0.02	0.00E+00
PAM	rs75901433	6	32531373	G	A	0.56	0.05	0.01	6.83E-07
PAM	rs56278466	10	17833858	G	T	0.66	0.11	0.01	2.17E-24
PAM	rs10509313	10	69512378	G	A	0.17	0.06	0.01	1.13E-05
PAM	rs12975366	19	54255498	C	T	0.40	-0.07	0.01	2.01E-11
PAMR1	rs1260326	2	27508073	C	T	0.61	-0.06	0.01	5.01E-08
PAMR1	rs1150753	6	32092090	G	A	0.13	-0.22	0.01	4.14E-47
PAMR1	rs61736408	11	35434513	C	T	0.16	-0.47	0.01	2.12E-220
PAMR1	rs4055121	11	126362442	T	C	0.14	-0.07	0.01	1.84E-05
PAPPA	rs967968	2	61428921	G	C	0.51	0.08	0.01	8.73E-12
PAPPA	rs34263006	2	102734543	A	C	0.22	-0.06	0.01	6.93E-06
PAPPA	rs4328821	3	128597592	G	A	0.11	-0.12	0.01	1.72E-11
PAPPA	rs13107325	4	102267552	T	C	0.07	0.10	0.01	9.84E-07

PAPPA	rs7808353	7	2407045	T	A	0.17	-0.08	0.01	4.63E-08
PAPPA	rs117448041	9	115580321	C	T	0.02	0.18	0.03	1.89E-05
PAPPA	rs9704782	11	46682556	T	G	0.26	-0.06	0.01	1.41E-05
PAPPA	rs147496186	11	49333270	C	G	0.04	-0.12	0.02	1.79E-05
PAPPA	rs550729	11	57391903	C	T	0.73	0.26	0.01	8.55E-98
PAPPA	rs56056911	15	101243641	C	G	0.14	-0.07	0.01	6.20E-06
PAPPA	rs74035509	16	88500925	T	C	0.08	0.11	0.01	2.82E-07
PAPPA	rs35617692	17	58195140	A	C	0.01	0.29	0.03	5.14E-08
PARK7	rs3766606	1	7962137	T	G	0.17	0.21	0.01	1.80E-44
PARK7	rs1354034	3	56815721	C	T	0.60	0.06	0.01	3.29E-07
PARK7	rs6993770	8	105569300	T	A	0.29	-0.05	0.01	1.72E-05
PARK7	rs10733789	10	63188924	C	T	0.31	0.05	0.01	2.30E-05
PARK7	rs11170874	12	54332691	G	A	0.33	0.05	0.01	1.07E-05
PARP1	rs1433574	1	226421638	C	A	0.16	-0.16	0.01	9.77E-25
PARP1	rs4632248	19	53821741	T	G	0.21	0.15	0.01	1.50E-27
PBLD	rs61854835	10	68279137	G	A	0.24	0.57	0.01	0.00E+00
PCDH1	rs4645358	5	141880520	T	C	0.57	0.08	0.01	2.92E-13
PCDH1	rs4077811	9	93078062	G	A	0.14	0.07	0.01	9.83E-06
PCDH1	rs587611953	9	133270004	A	C	0.16	0.17	0.01	1.62E-23
PCDH1	rs56278466	10	17833858	G	T	0.66	0.05	0.01	6.02E-06
PCDH1	-	10	80523403	A	T	0.73	0.06	0.01	9.76E-06
PCDH1	rs34937091	12	50652945	T	G	0.36	0.06	0.01	1.98E-06
PCDH1	rs58534292	14	21737747	C	T	0.65	-0.12	0.01	7.00E-26
PCDH1	rs60808632	14	106001848	A	C	0.63	-0.13	0.01	1.07E-27
PCDH1	rs77542162	17	69085137	G	A	0.02	0.17	0.02	6.77E-06
PCDH17	rs6789547	3	58440320	T	C	0.17	-0.07	0.01	5.49E-06
PCDH17	rs71536553	6	32560520	G	A	0.22	0.07	0.01	5.13E-07
PCDH17	rs8176749	9	133255801	T	C	0.06	0.64	0.01	5.68E-184
PCDH17	rs56278466	10	17833858	G	T	0.66	0.11	0.01	1.39E-23
PCDH17	rs11603123	11	126435600	A	G	0.03	0.17	0.02	1.06E-08
PCDH17	rs3184504	12	111446804	C	T	0.52	-0.08	0.01	1.67E-14
PCDH17	rs11148472	13	59446055	C	A	0.39	0.35	0.01	1.17E-211
PCDH17	rs186021206	17	7166093	A	G	0.01	0.35	0.05	1.94E-06
PCDH17	rs5757683	22	39454169	A	G	0.75	-0.07	0.01	5.22E-09
PCOLCE	rs9801017	7	100638579	A	G	0.62	0.17	0.01	8.56E-21
PCSK9	rs11591147	1	55039974	T	G	0.02	-1.14	0.03	6.08E-165
PCSK9	rs547186	2	21046369	A	T	0.50	0.07	0.01	1.90E-11
PCSK9	rs1260326	2	27508073	C	T	0.61	-0.07	0.01	7.25E-11
PCSK9	rs2001945	8	125465736	C	G	0.52	-0.06	0.01	1.56E-07
PCSK9	rs35473591	11	61818856	T	C	0.34	0.08	0.01	1.27E-11
PCSK9	rs673335	11	75739531	C	T	0.16	-0.07	0.01	1.22E-05
PCSK9	rs261342	15	58438954	C	G	0.78	-0.06	0.01	1.49E-06
PCSK9	rs8106503	19	11086210	C	T	0.12	-0.10	0.01	1.12E-08
PCSK9	rs58542926	19	19268740	T	C	0.08	-0.13	0.01	1.24E-09
PDCD1	rs37471	1	43811876	T	C	0.35	0.07	0.01	1.18E-09
PDCD1	rs2476601	1	113834946	G	A	0.90	-0.09	0.01	3.80E-06
PDCD1	rs376812565	2	122138	T	C	0.64	-0.06	0.01	6.95E-06
PDCD1	rs11888817	2	62303845	C	T	0.64	0.05	0.01	8.90E-06
PDCD1	rs3087243	2	203874196	A	G	0.45	-0.06	0.01	3.79E-07
PDCD1	-	2	241859600	C	G	0.20	-0.30	0.01	1.14E-101
PDCD1	rs9838308	3	170033504	C	T	0.37	-0.05	0.01	8.25E-06
PDCD1	rs3128981	6	31437843	G	A	0.30	0.06	0.01	1.51E-06
PDCD1	rs80318131	7	129143002	C	T	0.31	0.06	0.01	1.28E-05
PDCD1	rs12772559	10	69424600	C	G	0.26	0.08	0.01	7.03E-11
PDCD1	rs111338191	12	111388673	T	A	0.52	-0.10	0.01	3.79E-19
PDCD1	rs11460034	12	120807949	T	A	0.53	-0.05	0.01	2.46E-05

PDCD1	rs7161799	15	58478324	T	C	0.08	0.13	0.01	1.99E-09
PDCD1	rs186021206	17	7166093	A	G	0.01	0.60	0.05	1.99E-15
PDCD1	rs28394864	17	49373413	A	G	0.46	-0.05	0.01	2.11E-05
PDCD1	rs2659005	17	81244914	T	C	0.44	0.06	0.01	5.34E-08
PDCD1	rs753324368	19	10631005	A	C	0.74	-0.08	0.01	1.44E-09
PDCD1LG2	rs812490	1	43805985	C	T	0.45	-0.06	0.01	5.49E-09
PDCD1LG2	rs10935473	3	98698056	T	G	0.44	-0.46	0.01	0.00E+00
PDCD1LG2	rs2070725	5	132486096	T	C	0.34	-0.05	0.01	4.69E-06
PDCD1LG2	rs2381282	9	5514839	C	T	0.44	0.60	0.01	0.00E+00
PDCD1LG2	rs507666	9	133273983	A	G	0.18	-0.20	0.01	5.95E-46
PDCD1LG2	rs4055121	11	126362442	T	C	0.13	-0.08	0.01	5.44E-09
PDCD1LG2	rs3184504	12	111446804	C	T	0.52	-0.05	0.01	9.16E-09
PDCD1LG2	rs186021206	17	7166093	A	G	0.01	0.96	0.04	3.21E-50
PDCD5	rs1354034	3	56815721	C	T	0.60	0.07	0.01	3.70E-09
PDCD5	rs60822569	12	54323724	C	T	0.55	0.05	0.01	6.35E-06
PDCD5	rs10402931	19	32581163	A	G	0.68	0.38	0.01	6.53E-217
PDCD6	rs676705	1	56918624	A	G	0.35	-0.06	0.01	9.34E-11
PDCD6	rs9654451	5	299428	T	C	0.13	-1.48	0.01	0.00E+00
PDCD6	rs9495061	6	138380781	A	C	0.06	-0.08	0.01	2.60E-06
PDCD6	-	10	63399012	A	G	0.47	0.04	0.01	1.13E-06
PDCD6	rs10842898	12	9109693	T	G	0.35	-0.05	0.01	3.35E-10
PDCD6	rs112635299	14	94371805	T	G	0.02	0.15	0.02	8.32E-08
PDGFA	rs6671171	1	156901526	A	G	0.11	0.08	0.01	6.39E-06
PDGFA	rs3811444	1	247876149	T	C	0.33	-0.07	0.01	2.50E-09
PDGFA	rs7618405	3	18209017	A	C	0.21	-0.07	0.01	5.72E-06
PDGFA	rs1354034	3	56815721	C	T	0.60	-0.12	0.01	7.21E-26
PDGFA	rs2278668	3	123116385	C	T	0.59	-0.06	0.01	2.91E-06
PDGFA	rs4698839	4	101880784	T	C	0.40	-0.07	0.01	4.58E-09
PDGFA	rs10058074	5	132350453	A	G	0.48	-0.05	0.01	4.42E-06
PDGFA	rs2105957	6	31453863	G	C	0.08	-0.11	0.01	2.06E-07
PDGFA	rs146842698	6	70618570	A	T	0.31	-0.06	0.01	6.33E-06
PDGFA	rs2759391	6	163443194	C	A	0.34	-0.05	0.01	1.16E-05
PDGFA	rs147247048	7	592365	T	C	0.00	-0.44	0.07	2.20E-05
PDGFA	rs6961069	7	80589645	T	C	0.40	0.07	0.01	4.17E-09
PDGFA	rs6993770	8	105569300	T	A	0.29	-0.18	0.01	1.56E-47
PDGFA	rs9787438	10	63278270	C	G	0.41	0.12	0.01	1.01E-24
PDGFA	rs34377578	10	102576669	C	A	0.25	0.08	0.01	5.52E-10
PDGFA	rs5796220	12	6184174	T	C	0.49	-0.05	0.01	9.99E-06
PDGFA	rs59001897	15	64868193	A	T	0.17	0.08	0.01	1.64E-07
PDGFA	rs12445050	16	81837364	T	C	0.14	0.11	0.01	5.73E-12
PDGFA	rs1654425	19	55027612	C	T	0.83	0.17	0.01	1.62E-29
PDGFA	rs3827978	20	19300647	T	C	0.35	0.07	0.01	3.95E-10
PDGFB	rs75699653	1	156902203	T	C	0.02	0.16	0.02	2.03E-05
PDGFB	rs68066031	2	224015781	C	T	0.23	0.23	0.01	3.42E-64
PDGFB	rs1354034	3	56815721	C	T	0.60	-0.07	0.01	9.92E-11
PDGFB	rs755492124	4	101856385	T	G	0.39	-0.06	0.01	3.10E-08
PDGFB	rs274555	5	132387259	T	C	0.40	0.05	0.01	1.75E-05
PDGFB	rs2516471	6	31432515	A	G	0.06	-0.12	0.02	1.09E-06
PDGFB	rs1917342	6	70640051	G	A	0.30	-0.06	0.01	3.14E-06
PDGFB	rs6961069	7	80589645	T	C	0.40	0.07	0.01	1.40E-08
PDGFB	rs6993770	8	105569300	T	A	0.29	-0.14	0.01	6.44E-30
PDGFB	rs10820606	9	96430637	C	A	0.23	-0.08	0.01	4.52E-08
PDGFB	rs61469632	9	132986603	C	T	0.06	-0.10	0.02	1.72E-05
PDGFB	rs10740118	10	63341447	C	G	0.41	0.10	0.01	1.66E-19
PDGFB	rs34377578	10	102576669	C	A	0.25	0.08	0.01	6.70E-10
PDGFB	rs1362213	12	6173262	T	C	0.48	-0.05	0.01	4.05E-06

PDGFB	rs60128101	15	64868190	A	T	0.17	0.07	0.01	3.30E-06
PDGFB	rs11639051	15	101452793	A	C	0.30	0.09	0.01	2.62E-13
PDGFB	rs12445050	16	81837364	T	C	0.14	0.10	0.01	2.10E-10
PDGFB	rs1654425	19	55027612	C	T	0.83	0.17	0.01	6.48E-28
PDGFB	rs3827978	20	19300647	T	C	0.35	0.08	0.01	1.05E-10
PDGFC	rs12499706	4	156174698	G	C	0.27	-0.09	0.01	8.48E-13
PDGFC	rs4861708	4	186236079	A	G	0.51	-0.09	0.01	1.82E-13
PDGFC	rs75077631	5	177413083	C	G	0.74	-0.06	0.01	1.35E-05
PDGFC	rs150611042	9	114321523	A	C	0.07	-0.47	0.02	2.04E-92
PDGFC	rs28929474	14	94378610	T	C	0.02	0.35	0.03	2.37E-17
PDGFC	rs149394327	17	66232877	C	G	0.03	0.36	0.02	2.52E-25
PDGFC	rs1065853	19	44909976	T	G	0.08	0.18	0.01	8.84E-18
PDGFRA	rs61801010	1	161572445	G	A	0.11	0.15	0.01	3.21E-15
PDGFRA	rs56033718	3	58460671	G	A	0.37	0.08	0.01	2.03E-12
PDGFRA	rs4558798	3	98590071	G	A	0.67	-0.05	0.01	1.59E-05
PDGFRA	rs35597368	4	54273604	C	T	0.11	-0.42	0.01	2.29E-126
PDGFRA	rs9267947	6	32243441	G	A	0.48	0.05	0.01	1.29E-06
PDGFRA	rs142032527	7	150521013	T	A	0.24	-0.06	0.01	1.91E-05
PDGFRA	rs10761753	10	63402143	A	G	0.47	0.05	0.01	7.08E-06
PDGFRA	rs3751198	12	103753429	G	A	0.60	0.12	0.01	1.33E-27
PDGFRA	rs12148472	15	78939136	C	T	0.13	0.07	0.01	1.26E-05
PDGFRA	rs186021206	17	7166093	A	G	0.01	0.33	0.05	1.41E-05
PDGFRA	rs62217923	21	41167366	G	C	0.26	0.05	0.01	2.24E-05
PDGFRA	rs2294915	22	43945024	T	C	0.23	0.06	0.01	1.44E-06
PDGFRB	rs2304058	5	150128981	G	C	0.56	1.17	0.01	0.00E+00
PDGFRB	rs68055275	11	126369248	T	C	0.14	-0.07	0.01	4.61E-10
PDGFRB	rs186021206	17	7166093	A	G	0.01	0.42	0.03	1.42E-16
PDLIM7	rs1354034	3	56815721	C	T	0.60	0.05	0.01	5.17E-06
PDLIM7	rs342298	7	106733200	T	C	0.45	-0.08	0.01	8.09E-12
PDLIM7	rs11553699	12	121779004	G	A	0.14	-0.08	0.01	1.88E-06
PEAR1	rs4661012	1	156915699	G	T	0.36	-0.21	0.01	3.10E-72
PEAR1	rs1354034	3	56815721	C	T	0.60	-0.06	0.01	3.35E-08
PEAR1	rs13107325	4	102267552	T	C	0.08	0.16	0.01	7.44E-15
PEAR1	rs56293029	6	135097901	A	C	0.26	0.06	0.01	4.97E-06
PEAR1	rs296847	9	4793798	T	G	0.66	0.06	0.01	5.46E-07
PEAR1	rs8176741	9	133256074	A	G	0.06	0.43	0.02	7.20E-78
PEAR1	-	11	119269246	C	T	0.31	0.05	0.01	1.46E-05
PEAR1	rs3967200	11	126362490	T	C	0.13	-0.08	0.01	3.53E-06
PEAR1	rs370664797	12	89431434	C	T	0.25	-0.06	0.01	8.32E-06
PEAR1	rs3184504	12	111446804	C	T	0.52	-0.08	0.01	4.76E-12
PEAR1	rs186021206	17	7166093	A	G	0.01	0.74	0.05	1.59E-22
PEBP1	rs1354034	3	56815721	C	T	0.60	0.06	0.01	2.25E-07
PEBP1	rs10733789	10	63188924	C	T	0.31	0.05	0.01	1.93E-05
PEBP1	-	12	118261874	G	C	0.01	-0.48	0.04	1.21E-17
PECAM1	rs1354034	3	56815721	C	T	0.60	0.07	0.01	1.38E-11
PECAM1	rs10075801	5	132341949	G	A	0.49	-0.05	0.01	8.15E-06
PECAM1	rs342293	7	106731773	G	C	0.46	-0.06	0.01	2.46E-08
PECAM1	rs6993770	8	105569300	T	A	0.29	-0.05	0.01	1.96E-05
PECAM1	rs2519093	9	133266456	T	C	0.18	-0.58	0.01	0.00E+00
PECAM1	rs35166255	11	126431861	A	G	0.03	0.33	0.02	3.53E-28
PECAM1	rs3184504	12	111446804	C	T	0.52	-0.06	0.01	4.73E-07
PECAM1	rs186021206	17	7166093	A	G	0.01	0.61	0.05	1.54E-16
PFKFB2	rs1560	1	207077286	G	A	0.48	-0.19	0.01	4.37E-63
PFKFB2	rs1354034	3	56815721	C	T	0.60	0.06	0.01	2.62E-07
PFKFB2	rs6993770	8	105569300	T	A	0.29	-0.06	0.01	2.71E-06
PFKFB2	rs7080386	10	63288546	A	C	0.41	0.06	0.01	1.82E-06

PGF	rs9319430	13	28402948	A	T	0.53	0.08	0.01	1.00E-15
PGF	rs4902651	14	68805408	T	C	0.50	-0.05	0.01	1.06E-05
PGF	-	14	75029984	C	A	0.47	0.15	0.01	1.25E-50
PGF	rs704	17	28367840	A	G	0.47	-0.05	0.01	2.74E-06
PGLYRP1	rs3917932	1	36478315	G	C	0.58	-0.07	0.01	1.34E-09
PGLYRP1	rs113542380	2	43237679	A	G	0.07	0.14	0.01	3.46E-11
PGLYRP1	rs6436124	2	219176711	A	C	0.61	0.09	0.01	1.04E-13
PGLYRP1	rs2290547	3	47019693	A	G	0.18	0.06	0.01	1.65E-05
PGLYRP1	rs41266485	3	127671682	T	C	0.21	0.08	0.01	1.39E-08
PGLYRP1	rs218264	4	54542708	T	A	0.25	0.07	0.01	1.57E-07
PGLYRP1	rs141484466	6	31356775	C	G	0.04	0.31	0.02	1.10E-26
PGLYRP1	rs11775560	8	60747604	G	A	0.19	0.13	0.01	4.40E-21
PGLYRP1	rs4407843	8	129597122	C	A	0.57	-0.05	0.01	6.48E-06
PGLYRP1	rs2074038	11	44066439	T	G	0.10	0.12	0.01	2.44E-10
PGLYRP1	rs2511241	11	73234296	T	C	0.93	0.10	0.01	2.53E-06
PGLYRP1	rs371147134	12	31836996	A	G	0.52	-0.06	0.01	3.20E-07
PGLYRP1	rs7310615	12	111427245	G	C	0.52	-0.05	0.01	1.71E-05
PGLYRP1	-	17	39988115	T	A	0.54	-0.12	0.01	1.25E-28
PGLYRP1	-	19	4970712	A	G	0.23	-0.08	0.01	1.92E-09
PGLYRP1	rs2072563	19	46023390	A	G	0.33	-0.30	0.01	2.49E-136
PHOSPHO1	rs543343625	16	66956668	T	C	0.00	-1.83	0.14	3.48E-18
PI3	rs11679052	2	72029274	C	G	0.44	-0.06	0.01	5.94E-08
PI3	rs28550378	8	125596398	T	C	0.47	0.05	0.01	3.68E-06
PI3	rs7088444	10	79009948	T	C	0.48	0.06	0.01	8.25E-08
PI3	rs17333180	20	45175067	A	C	0.18	0.35	0.01	3.51E-138
PIGR	rs291102	1	206933133	A	G	0.02	-0.51	0.02	1.03E-43
PIGR	rs1200427	6	45558733	T	A	0.25	0.08	0.01	1.82E-09
PIGR	rs113810201	7	151229168	G	A	0.11	0.10	0.01	1.74E-08
PIGR	-	9	133255468	T	G	0.06	0.14	0.02	3.78E-09
PIGR	rs61882275	11	34482745	A	G	0.38	-0.06	0.01	2.49E-06
PIGR	rs708686	19	5840608	T	C	0.27	0.10	0.01	1.93E-15
PIGR	rs838136	19	48753131	C	T	0.28	-0.06	0.01	2.36E-05
PIK3AP1	rs11204523	1	247857254	C	G	0.76	-0.08	0.01	3.18E-09
PIK3AP1	rs1354034	3	56815721	C	T	0.60	0.10	0.01	4.50E-20
PIK3AP1	rs7695151	4	2770803	T	C	0.46	-0.05	0.01	1.29E-06
PIK3AP1	rs9264942	6	31306603	C	T	0.35	0.05	0.01	2.01E-05
PIK3AP1	rs78842280	6	47513892	G	T	0.27	-0.06	0.01	1.31E-06
PIK3AP1	rs342293	7	106731773	G	C	0.46	-0.05	0.01	4.32E-06
PIK3AP1	rs530506	9	133006062	A	G	0.55	0.05	0.01	2.19E-05
PIK3AP1	rs41317268	10	96709357	G	A	0.19	-0.23	0.01	3.89E-59
PIK3IP1	rs13146355	4	76490987	A	G	0.46	0.05	0.01	9.85E-06
PIK3IP1	rs28640218	16	20347945	T	G	0.19	-0.08	0.01	3.74E-08
PIK3IP1	rs2413028	22	31145921	C	T	0.17	-0.37	0.01	4.80E-147
PILRA	rs61747728	1	179557079	T	C	0.04	0.10	0.01	1.76E-05
PILRA	rs10085857	7	45216839	T	C	0.48	0.19	0.01	4.68E-58
PILRA	rs1859788	7	100374211	G	A	0.68	0.99	0.01	0.00E+00
PILRA	rs3184504	12	111446804	C	T	0.52	-0.06	0.01	4.70E-13
PILRA	rs186021206	17	7166093	A	G	0.01	0.55	0.04	2.84E-20
PILRA	rs55951667	17	76598422	T	C	0.37	-0.04	0.01	1.51E-05
PILRA	rs8077394	17	81284987	G	A	0.55	-0.05	0.01	1.01E-08
PILRB	rs10085857	7	45216839	T	C	0.48	0.22	0.01	1.58E-73
PILRB	rs1859788	7	100374211	G	A	0.68	1.14	0.01	0.00E+00
PILRB	rs7310615	12	111427245	G	C	0.52	-0.06	0.01	1.02E-12
PILRB	rs186021206	17	7166093	A	G	0.01	0.47	0.03	4.01E-18
PILRB	rs8077394	17	81284987	G	A	0.55	-0.05	0.01	1.01E-08
PKLR	rs77350683	1	155514639	C	T	0.07	-0.36	0.01	1.36E-58

PKLR	rs6592965	7	50360284	A	G	0.45	0.06	0.01	7.17E-08
PKLR	rs2361710	17	80138078	C	T	0.47	-0.05	0.01	5.18E-06
PKLR	rs737092	20	57415349	C	T	0.49	0.05	0.01	9.16E-06
PLA2G10	rs12136083	1	62697402	C	T	0.35	0.06	0.01	6.07E-07
PLA2G10	rs533617	2	21011100	C	T	0.04	0.13	0.02	4.48E-06
PLA2G10	rs1260326	2	27508073	C	T	0.61	0.05	0.01	3.53E-06
PLA2G10	rs149401529	3	52513395	C	G	0.01	-0.48	0.05	2.66E-11
PLA2G10	rs28367569	6	31268325	C	T	0.04	0.58	0.02	1.11E-106
PLA2G10	rs75278536	8	19963914	G	T	0.11	0.10	0.01	8.80E-09
PLA2G10	rs2976388	8	142678838	A	G	0.42	-0.26	0.01	3.17E-122
PLA2G10	-	9	133274293	A	C	0.18	-0.11	0.01	1.34E-15
PLA2G10	rs34481144	11	320836	T	C	0.50	-0.11	0.01	2.17E-23
PLA2G10	rs7232	11	60173126	A	T	0.37	0.07	0.01	6.35E-10
PLA2G10	rs964184	11	116778201	C	G	0.87	0.11	0.01	9.35E-13
PLA2G10	rs536340612	16	14564263	C	T	0.00	-1.42	0.06	1.91E-60
PLA2G10	rs186021206	17	7166093	A	G	0.01	0.38	0.05	6.75E-07
PLA2G10	rs112001035	17	68827664	A	G	0.06	0.16	0.02	5.04E-12
PLA2G10	rs1065853	19	44909976	T	G	0.08	0.21	0.01	3.53E-27
PLA2G10	rs516316	19	48702888	C	G	0.51	-0.07	0.01	1.76E-11
PLA2G10	-	20	45925083	T	A	0.19	-0.08	0.01	4.19E-09
PLA2G15	rs2187668	6	32638107	T	C	0.15	0.14	0.01	2.33E-19
PLA2G15	rs9346804	6	159979996	A	G	0.14	-0.08	0.01	1.69E-06
PLA2G15	rs139130389	11	72139110	A	C	0.07	-0.09	0.01	1.06E-05
PLA2G15	rs10778152	12	101831973	G	A	0.29	-0.23	0.01	8.38E-81
PLA2G15	rs7965471	12	113358761	C	A	0.48	0.09	0.01	4.69E-14
PLA2G15	rs145078947	14	93186629	T	G	0.00	1.25	0.07	1.69E-31
PLA2G15	rs3743588	16	11742652	A	G	0.29	0.06	0.01	8.92E-07
PLA2G15	rs7672	16	68260897	G	C	0.71	-0.25	0.01	6.97E-89
PLA2G15	rs186021206	17	7166093	A	G	0.01	0.47	0.05	8.02E-10
PLA2G15	-	17	46930667	T	A	0.64	-0.05	0.01	7.62E-06
PLA2G15	rs58542926	19	19268740	T	C	0.08	0.12	0.01	3.82E-09
PLA2G1B	rs35004807	1	50642739	A	T	0.34	0.06	0.01	1.82E-06
PLA2G1B	rs1260326	2	27508073	C	T	0.61	0.05	0.01	1.18E-05
PLA2G1B	rs17032925	2	67039351	C	T	0.08	-0.09	0.01	1.17E-05
PLA2G1B	rs376333472	2	240682394	A	G	0.02	0.17	0.02	2.30E-06
PLA2G1B	rs1361108	6	126446454	T	C	0.46	-0.08	0.01	6.41E-13
PLA2G1B	rs7003060	8	18872308	G	T	0.70	-0.06	0.01	5.75E-07
PLA2G1B	rs1372993	8	128553416	A	G	0.12	-0.16	0.01	1.01E-20
PLA2G1B	rs116842520	9	133078678	A	G	0.04	0.14	0.02	4.13E-06
PLA2G1B	rs2438988	10	48162047	G	A	0.64	-0.07	0.01	6.45E-09
PLA2G1B	rs4751995	10	116638373	G	A	0.52	-0.08	0.01	1.80E-12
PLA2G1B	rs61729512	12	7485173	A	G	0.12	0.08	0.01	6.40E-06
PLA2G1B	rs11065078	12	120310782	T	C	0.21	-0.07	0.01	3.61E-07
PLA2G1B	rs56994090	14	100840110	C	T	0.42	-0.05	0.01	7.61E-06
PLA2G1B	rs72802342	16	75200974	A	C	0.08	-0.20	0.01	1.05E-20
PLA2G1B	rs1673931	16	88910069	C	T	0.38	-0.13	0.01	2.03E-30
PLA2G1B	rs1468481	17	71143838	T	C	0.77	0.06	0.01	6.20E-06
PLA2G1B	rs6507586	18	44835125	G	A	0.08	0.09	0.01	9.96E-06
PLA2G1B	rs681343	19	48703205	T	C	0.51	0.10	0.01	4.63E-21
PLA2G1B	-	20	50218801	T	C	0.47	-0.05	0.01	6.90E-06
PLA2G1B	rs71327329	22	28931359	G	A	0.13	-0.08	0.01	5.10E-06
PLA2G2A	rs11573156	1	19979653	C	G	0.23	0.72	0.01	0.00E+00
PLA2G2A	rs6698842	1	65699049	T	C	0.63	0.08	0.01	9.63E-13
PLA2G2A	rs61729512	12	7485173	A	G	0.12	0.08	0.01	2.64E-07
PLA2G2A	rs34931250	17	68883786	T	C	0.06	0.18	0.01	2.84E-18
PLA2G2A	rs112896133	19	12318968	A	G	0.01	-0.21	0.03	4.37E-06

PLA2G2A	rs10405357	19	54255803	C	T	0.43	-0.05	0.01	6.17E-06
PLA2G4A	rs556306120	1	186967401	C	G	0.08	0.13	0.01	2.24E-09
PLA2G4A	rs1354034	3	56815721	C	T	0.60	0.06	0.01	1.16E-07
PLA2G4A	rs1149577	11	76834370	C	T	0.21	0.06	0.01	8.65E-06
PLA2G7	rs11591147	1	55039974	T	G	0.02	-0.22	0.03	2.36E-07
PLA2G7	rs7528419	1	109274570	G	A	0.22	-0.10	0.01	5.17E-14
PLA2G7	rs934197	2	21044589	A	G	0.34	0.05	0.01	1.07E-05
PLA2G7	rs6871667	5	75308917	A	G	0.40	0.05	0.01	5.71E-06
PLA2G7	rs574476364	6	46717995	T	C	0.00	-1.45	0.11	1.90E-17
PLA2G7	rs13702	8	19966981	C	T	0.29	-0.05	0.01	2.60E-05
PLA2G7	rs28601761	8	125487789	G	C	0.42	-0.06	0.01	7.43E-08
PLA2G7	rs562028	11	60110377	C	A	0.63	0.08	0.01	2.61E-11
PLA2G7	rs964184	11	116778201	C	G	0.87	-0.13	0.01	5.91E-16
PLA2G7	-	19	11085680	A	C	0.12	-0.13	0.01	2.55E-14
PLA2G7	rs1065853	19	44909976	T	G	0.08	-0.47	0.01	6.73E-123
PLAT	rs7765526	6	147392628	G	A	0.54	0.05	0.01	1.46E-05
PLAT	rs796629870	7	101126425	G	T	0.46	-0.06	0.01	4.26E-08
PLAT	rs2020921	8	42187447	A	G	0.03	-0.28	0.02	4.39E-16
PLAT	rs4576860	12	130788364	G	A	0.58	-0.05	0.01	9.47E-07
PLAT	rs8112983	19	48726268	C	T	0.54	0.07	0.01	5.18E-10
PLAU	rs11888817	2	62303845	C	T	0.64	0.05	0.01	2.67E-06
PLAU	rs776068721	2	159943114	T	G	0.51	-0.09	0.01	2.45E-15
PLAU	rs2470750	3	98971748	T	A	0.39	-0.07	0.01	6.50E-09
PLAU	rs6858148	4	99144766	C	T	0.36	0.05	0.01	6.12E-06
PLAU	rs8176741	9	133256074	A	G	0.06	0.17	0.01	2.25E-14
PLAU	rs369705328	10	17806193	C	G	0.13	0.08	0.01	9.10E-07
PLAU	rs55744193	10	73912301	A	G	0.01	-0.82	0.04	2.15E-38
PLAU	rs4055121	11	126362442	T	C	0.14	-0.15	0.01	2.24E-20
PLAU	rs186021206	17	7166093	A	G	0.01	1.30	0.05	2.26E-69
PLAU	rs9948409	18	27181964	G	A	0.70	0.11	0.01	1.15E-20
PLAU	rs36229204	19	43671830	T	C	0.03	-0.52	0.02	5.42E-64
PLAUR	rs72955755	2	159861821	G	C	0.46	-0.10	0.01	2.74E-20
PLAUR	rs9378247	6	31353648	A	G	0.18	-0.06	0.01	1.40E-05
PLAUR	rs8176672	9	133266772	T	C	0.06	0.11	0.01	2.35E-06
PLAUR	rs2688623	10	73929857	T	C	0.56	0.11	0.01	3.22E-22
PLAUR	rs2511241	11	73234296	T	C	0.93	0.14	0.01	4.04E-11
PLAUR	rs60843925	11	126368937	C	T	0.14	-0.10	0.01	5.10E-09
PLAUR	rs7137828	12	111494996	T	C	0.52	-0.05	0.01	9.84E-07
PLAUR	rs77924615	16	20381010	A	G	0.20	-0.06	0.01	2.13E-05
PLAUR	rs186021206	17	7166093	A	G	0.01	0.90	0.05	1.13E-33
PLAUR	rs2241244	17	39997301	C	G	0.54	-0.05	0.01	1.75E-05
PLAUR	rs2302524	19	43652320	C	T	0.17	-0.18	0.01	5.83E-34
PLIN1	rs150004289	15	89673215	A	G	0.00	-0.74	0.07	7.73E-12
PLIN3	rs6993770	8	105569300	T	A	0.29	-0.06	0.01	1.29E-06
PLIN3	rs262557	19	4872083	T	C	0.76	0.08	0.01	1.81E-08
PLPBP	rs1354034	3	56815721	C	T	0.60	0.07	0.01	8.06E-09
PLPBP	rs6993770	8	105569300	T	A	0.29	-0.06	0.01	1.02E-06
PLTP	rs34211178	3	98664718	A	G	0.44	-0.08	0.01	1.41E-13
PLTP	rs3916027	8	19967357	A	G	0.26	0.05	0.01	7.76E-06
PLTP	rs13284054	9	104906792	C	T	0.12	-0.07	0.01	8.21E-06
PLTP	rs174533	11	61781553	A	G	0.35	-0.06	0.01	5.45E-07
PLTP	rs78689694	11	126364925	C	G	0.13	-0.09	0.01	6.00E-09
PLTP	rs147233090	15	43735849	T	C	0.02	-0.18	0.02	6.09E-07
PLTP	rs35853021	15	58388444	T	G	0.36	0.09	0.01	7.55E-17
PLTP	rs247617	16	56956804	A	C	0.32	0.12	0.01	1.94E-27
PLTP	rs4986970	16	67942417	T	A	0.04	-0.13	0.02	1.72E-05

PLTP	rs186021206	17	7166093	A	G	0.01	0.98	0.05	4.41E-42
PLTP	rs6073958	20	45923216	C	T	0.20	-0.50	0.01	6.33E-290
PLXDC1	rs3775298	4	186229324	G	A	0.51	-0.07	0.01	4.79E-10
PLXDC1	rs11386832	5	96785666	A	T	0.74	0.13	0.01	7.05E-25
PLXDC1	rs885122	5	115995638	G	A	0.40	-0.06	0.01	3.39E-06
PLXDC1	rs79428469	6	32634257	C	T	0.22	0.06	0.01	1.62E-05
PLXDC1	rs142988850	7	100166617	G	A	0.00	0.50	0.05	8.57E-10
PLXDC1	rs62076601	17	39067156	T	C	0.05	-0.53	0.02	3.87E-86
PLXNA4	rs1354034	3	56815721	C	T	0.60	0.10	0.01	2.66E-17
PLXNA4	rs17622656	5	132485305	A	G	0.39	-0.06	0.01	9.74E-08
PLXNA4	rs6467443	7	132553252	T	G	0.22	0.10	0.01	1.42E-13
PLXNA4	rs6993770	8	105569300	T	A	0.29	-0.10	0.01	1.48E-16
PLXNA4	rs1671152	19	55014977	G	T	0.84	0.08	0.01	1.08E-06
PLXNB2	-	1	161704318	G	A	0.11	0.12	0.01	8.86E-15
PLXNB2	rs7523508	1	177968620	G	A	0.28	-0.05	0.01	2.76E-06
PLXNB2	rs1260326	2	27508073	C	T	0.61	0.05	0.01	1.32E-07
PLXNB2	rs1126735	3	58427681	G	A	0.37	0.05	0.01	2.35E-07
PLXNB2	rs9269965	6	32584442	G	A	0.39	-0.06	0.01	3.56E-07
PLXNB2	rs2519093	9	133266456	T	C	0.18	-0.12	0.01	8.90E-22
PLXNB2	rs56278466	10	17833858	G	T	0.66	0.19	0.01	3.00E-80
PLXNB2	rs10774624	12	111395984	A	G	0.51	-0.04	0.01	2.20E-05
PLXNB2	rs28929474	14	94378610	T	C	0.02	0.15	0.02	1.54E-05
PLXNB2	rs186021206	17	7166093	A	G	0.01	0.31	0.04	3.12E-06
PLXNB2	rs1801689	17	66214462	C	A	0.03	0.14	0.02	5.41E-07
PLXNB2	rs868876	19	7766340	T	A	0.30	-0.06	0.01	6.13E-08
PLXNB2	rs11547731	22	50283705	C	T	0.65	-0.70	0.01	0.00E+00
PLXNB3	rs9402686	6	135106679	A	G	0.27	0.06	0.01	1.10E-05
PLXNB3	rs6993770	8	105569300	T	A	0.29	-0.06	0.01	1.46E-05
PLXNB3	rs409801	9	4744743	C	T	0.51	0.06	0.01	3.05E-07
PLXNB3	rs4502008	11	126417859	T	G	0.52	-0.06	0.01	2.50E-07
PLXNB3	rs111338191	12	111388673	T	A	0.52	-0.07	0.01	1.45E-08
PLXNB3	rs1613662	19	55025227	A	G	0.83	0.08	0.01	1.72E-06
PM20D1	rs760077	1	155208991	T	A	0.60	-0.07	0.01	6.86E-10
PM20D1	rs112268616	1	205847765	C	T	0.34	-0.83	0.01	0.00E+00
PM20D1	rs11680123	2	60287613	G	A	0.59	0.12	0.01	1.84E-41
PM20D1	rs121908120	2	218890289	A	T	0.03	-0.12	0.02	1.17E-05
PM20D1	rs11707641	3	12114388	C	T	0.16	0.06	0.01	4.47E-06
PM20D1	rs12496730	3	98688089	C	G	0.44	-0.04	0.01	2.06E-05
PM20D1	rs2519093	9	133266456	T	C	0.18	-0.06	0.01	1.90E-06
PM20D1	-	10	95142972	G	A	0.58	-0.05	0.01	5.68E-07
PM20D1	rs36034702	15	90191395	T	C	0.17	-0.06	0.01	7.27E-07
PMVK	rs114951074	1	154931441	T	C	0.01	-0.67	0.05	4.95E-21
PMVK	rs1354034	3	56815721	C	T	0.60	0.06	0.01	1.76E-06
PMVK	rs342293	7	106731773	G	C	0.46	-0.07	0.01	3.85E-09
PMVK	rs6993770	8	105569300	T	A	0.29	-0.06	0.01	8.08E-07
PMVK	rs7896518	10	63344740	G	A	0.42	0.05	0.01	8.19E-06
PMVK	rs6580981	12	54329244	A	G	0.46	0.05	0.01	1.18E-05
PNLIPRP2	rs4364486	6	96086513	T	C	0.79	0.04	0.01	1.93E-05
PNLIPRP2	rs8176743	9	133256028	T	C	0.06	0.20	0.01	2.20E-41
PNLIPRP2	rs56278466	10	17833858	G	T	0.66	0.05	0.01	1.65E-05
PNLIPRP2	rs4751995	10	116638373	G	A	0.52	1.13	0.01	0.00E+00
PNLIPRP2	rs72802342	16	75200974	A	C	0.08	-0.11	0.01	4.30E-16
PNLIPRP2	rs2608894	19	5847989	C	T	0.76	-0.04	0.01	1.11E-05
PNLIPRP2	rs601338	19	48703417	A	G	0.51	0.16	0.00	4.49E-103
PNPT1	rs9394070	6	31398518	A	C	0.06	-0.14	0.02	5.49E-09
PNPT1	rs6001857	22	22335340	C	T	0.17	-0.08	0.01	6.07E-07

PODXL	rs767298	7	7234953	T	G	0.63	-0.16	0.01	3.51E-45
PODXL	rs3212298	7	131508980	T	C	0.04	-0.56	0.02	3.41E-96
PODXL	rs590666	9	76488450	C	T	0.52	-0.10	0.01	9.85E-18
PODXL	rs2519093	9	133266456	T	C	0.18	-0.55	0.01	0.00E+00
PODXL	rs34434834	11	126437901	A	G	0.03	0.33	0.02	3.45E-28
PODXL	rs186021206	17	7166093	A	G	0.01	0.86	0.05	5.46E-30
PODXL2	rs61744853	1	34988050	C	A	0.01	-0.25	0.04	1.39E-05
PODXL2	rs189026820	1	161616771	A	G	0.14	0.07	0.01	1.01E-05
PODXL2	rs61747728	1	179557079	T	C	0.04	0.13	0.02	1.03E-05
PODXL2	rs1047891	2	210675783	A	C	0.31	0.05	0.01	1.43E-05
PODXL2	rs13303	3	52523992	C	T	0.56	0.05	0.01	2.24E-05
PODXL2	-	3	127629383	T	C	0.01	-1.11	0.03	2.30E-114
PODXL2	rs781656	4	56912479	A	G	0.18	0.17	0.01	9.56E-33
PODXL2	rs9471974	6	42951246	A	T	0.45	-0.05	0.01	2.64E-05
PODXL2	-	9	133276163	T	A	0.19	0.07	0.01	8.27E-07
PODXL2	rs74841302	15	41792131	A	G	0.02	-0.19	0.03	8.40E-06
PODXL2	rs9922607	16	17476363	T	C	0.21	0.11	0.01	4.22E-17
PODXL2	rs6504649	17	50360095	G	C	0.39	-0.12	0.01	7.83E-25
PODXL2	rs55639531	18	394341	G	C	0.29	0.06	0.01	2.46E-06
PODXL2	rs45512696	19	35059974	T	C	0.18	-0.07	0.01	3.01E-06
POLR2F	rs7137828	12	111494996	T	C	0.52	-0.05	0.01	8.16E-06
PON2	rs149369377	1	196850349	G	A	0.20	0.07	0.01	3.16E-10
PON2	rs6961624	7	95408349	C	T	0.24	-0.90	0.01	0.00E+00
PON2	rs28929474	14	94378610	T	C	0.02	0.21	0.02	1.50E-10
PON2	rs58542926	19	19268740	T	C	0.08	0.15	0.01	1.31E-18
PON3	rs6677604	1	196717788	A	G	0.19	0.09	0.01	2.25E-10
PON3	rs34060476	7	73623626	G	A	0.13	0.07	0.01	2.03E-05
PON3	rs149867961	7	95396432	C	T	0.03	-1.05	0.02	1.07E-199
PON3	rs4240624	8	9326721	A	G	0.91	0.10	0.01	1.81E-07
PON3	rs3208305	8	19966137	T	A	0.29	0.06	0.01	1.28E-06
PON3	rs1077835	15	58431227	G	A	0.22	0.06	0.01	6.23E-06
PON3	rs247617	16	56956804	A	C	0.32	0.07	0.01	4.31E-10
PON3	rs149394327	17	66232877	C	G	0.03	0.18	0.02	1.13E-08
PON3	rs34415062	19	58511799	A	G	0.18	0.16	0.01	2.94E-31
PPCDC	rs1354034	3	56815721	C	T	0.60	0.05	0.01	7.65E-06
PPCDC	rs12591994	15	75027965	G	A	0.06	0.67	0.01	8.90E-193
PPIB	rs1354034	3	56815721	C	T	0.60	0.05	0.01	8.93E-06
PPIB	rs6993770	8	105569300	T	A	0.29	-0.07	0.01	1.00E-08
PPIB	rs1671152	19	55014977	G	T	0.84	0.07	0.01	4.11E-06
PPME1	rs3782123	11	205198	A	C	0.73	0.06	0.01	5.99E-06
PPP1R12A	rs68066031	2	224015781	C	T	0.23	0.10	0.01	2.87E-14
PPP1R12A	rs1354034	3	56815721	C	T	0.60	0.05	0.01	3.48E-06
PPP1R12A	rs17622656	5	132485305	A	G	0.39	-0.05	0.01	1.71E-05
PPP1R12A	rs342296	7	106732457	A	G	0.46	-0.06	0.01	1.67E-08
PPP1R2	rs1354034	3	56815721	C	T	0.60	0.08	0.01	1.57E-11
PPP1R2	rs4746204	10	63568758	T	C	0.48	-0.05	0.01	7.29E-06
PPP1R2	rs11502185	11	180258	C	T	0.26	0.07	0.01	1.28E-06
PPP1R2	rs1613662	19	55025227	A	G	0.83	0.07	0.01	1.52E-05
PPP1R9B	rs1354034	3	56815721	C	T	0.60	0.07	0.01	7.11E-09
PPP1R9B	rs342298	7	106733200	T	C	0.46	-0.05	0.01	1.55E-05
PPP3R1	rs188468174	1	24965206	T	C	0.01	-0.32	0.03	4.24E-11
PPP3R1	rs1354034	3	56815721	C	T	0.60	0.05	0.01	9.13E-06
PPP3R1	rs55768089	4	101822541	A	T	0.31	0.10	0.01	2.21E-17
PPP3R1	rs915651	6	31781407	A	G	0.13	-0.08	0.01	1.73E-06
PPP3R1	rs9414801	10	63389329	A	G	0.53	-0.07	0.01	9.06E-09
PPY	rs1493691	1	119989350	A	G	0.11	-0.10	0.01	1.33E-08

PPY	rs73174306	3	169476456	T	A	0.04	0.42	0.02	3.91E-52
PPY	rs6451770	5	44691293	G	T	0.38	-0.06	0.01	3.63E-07
PPY	rs9348441	6	20680447	A	T	0.26	-0.07	0.01	2.74E-07
PPY	rs9375435	6	126340712	T	C	0.45	-0.06	0.01	7.97E-09
PPY	rs184163936	7	156988778	C	T	0.01	-0.36	0.05	5.26E-07
PPY	rs2438988	10	48162047	G	A	0.64	-0.21	0.01	6.70E-71
PPY	rs375124982	10	117370918	T	G	0.31	-0.05	0.01	1.01E-05
PPY	rs139130389	11	72139110	A	C	0.07	-0.09	0.01	2.39E-05
PPY	rs9320010	18	55386666	G	A	0.40	0.05	0.01	6.07E-06
PRCP	rs3010061	1	183855735	T	C	0.20	0.06	0.01	2.06E-05
PRCP	rs11125916	2	62382243	A	C	0.19	0.07	0.01	2.36E-06
PRCP	rs13111888	4	76210141	A	G	0.18	0.06	0.01	2.48E-05
PRCP	rs142032527	7	150521013	T	A	0.24	-0.06	0.01	7.13E-06
PRCP	rs11250073	8	10774726	T	G	0.61	-0.07	0.01	3.04E-09
PRCP	rs10104003	8	54509880	T	C	0.21	0.16	0.01	3.64E-35
PRCP	rs10116966	9	33122372	A	T	0.31	-0.08	0.01	1.76E-12
PRCP	rs56278466	10	17833858	G	T	0.66	0.14	0.01	2.62E-37
PRCP	rs543233230	11	82795670	A	C	0.00	-1.48	0.08	2.21E-33
PRCP	rs540730	12	57413331	C	T	0.75	-0.08	0.01	1.80E-09
PRCP	rs4764823	12	101825988	A	G	0.29	-0.22	0.01	3.73E-73
PRCP	rs145078947	14	93186629	T	G	0.00	1.06	0.07	3.75E-24
PRCP	rs738409	22	43928847	G	C	0.22	0.06	0.01	2.77E-05
PRDX1	rs2356552	1	45543644	C	T	0.90	0.11	0.01	7.22E-09
PRDX1	rs6993770	8	105569300	T	A	0.29	-0.07	0.01	2.32E-07
PRDX3	rs7358070	10	119197216	C	G	0.32	-0.07	0.01	1.38E-07
PRDX5	rs3847326	10	63572099	G	A	0.48	-0.05	0.01	2.43E-05
PRDX5	rs77269065	11	64319797	A	G	0.01	0.99	0.05	3.00E-35
PRDX6	rs33951697	1	173489187	T	C	0.18	0.17	0.01	2.23E-33
PRELP	rs41313926	1	203483281	A	G	0.01	-2.26	0.03	0.00E+00
PRKAB1	rs1354034	3	56815721	C	T	0.60	0.05	0.01	9.49E-06
PRKAB1	rs9414801	10	63389329	A	G	0.53	-0.05	0.01	1.51E-05
PRKAB1	rs749140768	12	119671479	A	C	0.08	0.35	0.01	4.90E-60
PRKAR1A	rs68066031	2	224015781	C	T	0.23	0.22	0.01	2.34E-57
PRKAR1A	rs1354034	3	56815721	C	T	0.60	0.07	0.01	1.38E-08
PRKAR1A	rs342293	7	106731773	G	C	0.46	-0.05	0.01	2.90E-06
PRKAR1A	rs7896518	10	63344740	G	A	0.42	0.06	0.01	2.58E-07
PRKAR1A	rs1552950	15	101454028	G	C	0.29	0.07	0.01	9.46E-09
PRKRA	rs1354034	3	56815721	C	T	0.60	0.10	0.01	3.59E-18
PRKRA	rs11502185	11	180258	C	T	0.26	0.08	0.01	3.02E-07
PRKRA	rs60822569	12	54323724	C	T	0.55	0.07	0.01	2.07E-08
PRKRA	-	17	35559053	T	A	0.17	0.13	0.01	9.27E-19
PRL	rs4738	5	1461453	T	C	0.73	-0.06	0.01	1.52E-05
PRL	-	6	126421582	C	T	0.47	0.07	0.01	5.41E-10
PRL	rs2094622	9	5105679	A	C	0.50	-0.08	0.01	7.45E-12
PRL	rs11042976	11	2177029	A	G	0.48	-0.05	0.01	6.65E-06
PROC	rs599839	1	109279544	A	G	0.77	0.08	0.01	2.98E-11
PROC	rs1260326	2	27508073	C	T	0.61	-0.13	0.01	3.05E-33
PROC	rs1799810	2	127418464	T	A	0.43	-0.20	0.01	4.81E-77
PROC	rs13108218	4	3442204	G	A	0.62	-0.06	0.01	6.31E-07
PROC	-	4	82636278	T	A	0.19	-0.06	0.01	1.82E-05
PROC	rs9275239	6	32692940	A	G	0.48	0.05	0.01	8.53E-07
PROC	rs13226650	7	73602675	G	A	0.20	-0.08	0.01	1.36E-08
PROC	rs2393969	10	63380680	C	A	0.47	-0.05	0.01	5.93E-06
PROC	rs149394327	17	66232877	C	G	0.03	0.14	0.02	7.68E-06
PROC	-	20	35180847	G	T	0.09	0.78	0.01	0.00E+00
PROK1	rs1857512	1	110450727	A	G	0.16	0.48	0.01	0.00E+00

PROK1	rs4607429	6	32616054	C	G	0.14	-0.06	0.01	1.13E-07
PROK1	rs7497289	15	40041456	A	G	0.28	0.04	0.01	9.63E-06
PRSS2	rs2487583	1	119976376	A	G	0.11	-0.08	0.01	2.36E-05
PRSS2	rs34296809	4	39392695	C	T	0.21	0.07	0.01	1.43E-06
PRSS2	rs446145	6	29553010	C	G	0.49	0.05	0.01	6.99E-06
PRSS2	rs9482772	6	127125645	C	T	0.45	0.08	0.01	3.12E-12
PRSS2	rs3752404	7	142762725	G	A	0.42	-0.14	0.01	1.67E-33
PRSS2	rs748208	8	18855003	C	T	0.71	-0.07	0.01	5.81E-08
PRSS2	rs4733612	8	128557753	A	G	0.73	0.07	0.01	2.75E-07
PRSS2	rs12216891	9	133251979	T	C	0.06	0.10	0.02	2.69E-05
PRSS2	rs174580	11	61839170	G	A	0.35	0.06	0.01	1.28E-07
PRSS2	rs686056	11	100775958	A	G	0.32	-0.08	0.01	2.31E-10
PRSS2	rs72802342	16	75200974	A	C	0.08	-0.27	0.01	5.85E-35
PRSS2	rs533406	16	88908452	G	A	0.38	-0.10	0.01	1.67E-19
PRSS2	rs17138478	17	37713312	A	C	0.13	0.08	0.01	4.62E-06
PRSS2	rs7234846	18	44823175	A	T	0.08	0.11	0.01	4.47E-07
PRSS27	rs1036332	1	199043349	C	A	0.74	-0.06	0.01	4.23E-06
PRSS27	rs545694574	3	141399127	T	C	0.42	-0.09	0.01	2.50E-16
PRSS27	rs541115	3	187920304	A	T	0.34	0.05	0.01	2.35E-05
PRSS27	rs10010281	4	105184974	C	G	0.21	0.06	0.01	6.47E-07
PRSS27	rs2479005	6	2337266	G	A	0.47	-0.16	0.01	7.17E-52
PRSS27	rs6993770	8	105569300	T	A	0.29	0.05	0.01	2.38E-05
PRSS27	rs3824458	9	33144811	T	C	0.31	-0.06	0.01	9.41E-07
PRSS27	-	9	36291177	T	C	0.43	-0.12	0.01	6.11E-31
PRSS27	rs141129381	9	97929618	G	T	0.36	0.08	0.01	7.38E-13
PRSS27	rs635634	9	133279427	T	C	0.18	-0.06	0.01	9.33E-06
PRSS27	rs112972631	10	102598421	T	G	0.25	-0.06	0.01	1.78E-06
PRSS27	rs546202	11	65742553	C	T	0.55	0.06	0.01	2.53E-09
PRSS27	rs11220477	11	126405507	T	C	0.04	-0.52	0.02	1.23E-91
PRSS27	rs7306988	12	510642	A	G	0.17	-0.07	0.01	5.12E-07
PRSS27	rs79755767	12	54304624	A	G	0.10	0.08	0.01	1.30E-05
PRSS27	rs71386687	16	2717893	T	G	0.08	0.38	0.01	9.49E-85
PRSS27	rs708686	19	5840608	T	C	0.27	0.16	0.01	2.27E-36
PRSS27	rs681343	19	48703205	T	C	0.51	0.55	0.01	0.00E+00
PRSS27	rs1654439	19	55042279	G	T	0.83	0.08	0.01	6.94E-07
PRSS27	rs587935	21	39538813	T	C	0.67	0.06	0.01	3.94E-07
PRSS8	rs1260326	2	27508073	C	T	0.61	-0.07	0.01	2.64E-09
PRSS8	rs7714420	5	75044502	C	A	0.28	-0.05	0.01	1.49E-05
PRSS8	rs28366311	6	32593360	G	C	0.28	0.06	0.01	5.16E-07
PRSS8	rs59084494	6	130036899	A	G	0.58	0.05	0.01	1.41E-05
PRSS8	rs35332062	7	73597712	A	G	0.13	-0.11	0.01	4.91E-11
PRSS8	rs764066899	8	125480557	A	T	0.19	0.06	0.01	7.44E-06
PRSS8	rs62032958	16	365317	A	G	0.07	-0.20	0.01	6.37E-21
PRSS8	rs889555	16	31111250	T	C	0.28	0.11	0.01	3.51E-22
PRSS8	rs149460787	17	47594412	C	T	0.51	0.06	0.01	3.38E-08
PRSS8	rs57822461	19	38241943	T	C	0.18	-0.10	0.01	4.48E-12
PRTFDC1	rs17622656	5	132485305	A	G	0.39	-0.05	0.01	2.62E-05
PRTFDC1	rs143356584	10	24952573	T	C	0.01	-0.85	0.03	1.80E-67
PRTFDC1	rs4759076	12	54336088	C	T	0.46	0.06	0.01	1.99E-06
PRTG	rs13107325	4	102267552	T	C	0.08	0.10	0.01	2.96E-06
PRTG	rs28507026	6	32614708	G	A	0.16	0.07	0.01	7.75E-07
PRTG	rs8176746	9	133255935	T	G	0.06	0.30	0.01	1.05E-42
PRTG	rs10851591	15	55711507	G	A	0.69	0.45	0.01	4.47E-296
PRTG	rs7498431	16	88048298	G	C	0.61	0.09	0.01	3.62E-17
PRTG	rs186021206	17	7166093	A	G	0.01	0.57	0.05	7.27E-15
PRTG	rs704	17	28367840	A	G	0.47	-0.06	0.01	1.30E-07

PRTG	rs77542162	17	69085137	G	A	0.02	0.18	0.02	4.26E-07
PRTG	rs6565921	18	76845797	G	A	0.63	-0.06	0.01	7.71E-09
PRTN3	rs3917932	1	36478315	G	C	0.58	-0.07	0.01	1.01E-10
PRTN3	rs10443175	1	76079728	T	C	0.23	0.07	0.01	3.07E-07
PRTN3	rs112694524	2	43226582	A	G	0.07	0.10	0.01	9.83E-06
PRTN3	rs13135092	4	102276925	G	A	0.08	0.09	0.01	1.42E-05
PRTN3	rs41559422	6	31355890	A	G	0.04	0.24	0.02	3.12E-17
PRTN3	rs915125	6	81753659	T	C	0.28	0.09	0.01	9.81E-13
PRTN3	rs11155297	6	143503967	T	G	0.26	-0.06	0.01	3.01E-06
PRTN3	rs2977810	8	6957935	C	T	0.33	0.07	0.01	1.48E-08
PRTN3	rs10107630	8	129591389	T	C	0.57	-0.09	0.01	8.14E-16
PRTN3	rs118119317	9	79611770	A	G	0.03	-0.14	0.02	9.58E-06
PRTN3	rs73000965	11	114111599	A	T	0.32	0.06	0.01	1.28E-06
PRTN3	rs16942887	16	67894139	A	G	0.12	0.07	0.01	1.92E-05
PRTN3	rs6503533	17	40028327	T	C	0.62	-0.09	0.01	9.44E-18
PRTN3	rs56378716	17	58279141	G	A	0.01	0.24	0.03	3.65E-07
PRTN3	rs10425544	19	836043	C	T	0.71	0.49	0.01	6.35E-300
PSG1	rs7526027	1	59218322	C	T	0.29	0.05	0.01	1.38E-06
PSG1	rs78689694	11	126364925	C	G	0.13	-0.10	0.01	2.99E-13
PSG1	rs745680717	12	480706	C	G	0.40	0.04	0.01	1.53E-05
PSG1	rs909222	14	75443024	C	A	0.46	0.05	0.01	8.42E-07
PSG1	rs186021206	17	7166093	A	G	0.01	0.32	0.04	3.55E-07
PSG1	rs12986075	19	42868882	A	G	0.20	-0.96	0.01	0.00E+00
PSG1	rs61740142	20	2483466	C	T	0.04	-0.29	0.02	8.39E-35
PSG1	rs2426439	20	52383088	C	T	0.63	0.04	0.01	7.34E-06
PSIP1	rs1354034	3	56815721	C	T	0.60	0.13	0.01	1.73E-28
PSIP1	rs28383233	6	32616376	A	G	0.58	0.05	0.01	1.51E-05
PSIP1	rs7080536	10	113588287	A	G	0.04	-0.15	0.02	4.47E-08
PSIP1	rs111338191	12	111388673	T	A	0.52	-0.06	0.01	2.41E-07
PSMD9	-	12	121938985	T	A	0.29	-0.19	0.01	2.12E-57
PSME1	rs1354034	3	56815721	C	T	0.60	0.05	0.01	3.05E-06
PSME1	rs35350651	12	111469627	C	A	0.50	-0.05	0.01	2.62E-05
PSME1	rs2236352	14	24141524	C	T	0.26	0.09	0.01	6.22E-13
PSME2	rs2236352	14	24141524	C	T	0.26	0.10	0.01	2.12E-15
PSMG3	rs9503502	6	3260321	A	T	0.83	-0.20	0.01	3.83E-41
PSMG3	rs34407549	7	1569176	G	C	0.01	-0.29	0.04	1.13E-05
PSMG3	-	7	95305886	C	A	0.20	-0.06	0.01	1.10E-05
PSPN	rs4381790	2	18490529	A	C	0.55	-0.05	0.01	5.84E-08
PSPN	rs6542472	2	118571862	C	A	0.36	0.04	0.01	3.89E-06
PSPN	rs2102843	2	232303910	C	T	0.06	-0.10	0.01	1.94E-07
PSPN	rs13111751	4	119129607	G	C	0.34	-0.05	0.01	1.08E-06
PSPN	rs7488780	12	20426458	C	G	0.21	-0.08	0.01	7.47E-14
PSPN	rs9543374	13	73469980	T	C	0.26	-0.07	0.01	2.23E-13
PSPN	rs138903858	14	105168150	T	G	0.03	-0.14	0.02	1.89E-07
PSPN	rs147382204	17	46691231	G	T	0.33	0.04	0.01	1.09E-05
PSPN	rs749522465	18	51383818	A	G	0.31	0.05	0.01	5.31E-07
PSPN	rs58634535	20	3660895	A	G	0.16	-0.05	0.01	6.70E-06
PSPN	rs549768142	20	10669029	T	G	0.80	0.06	0.01	3.59E-09
PSPN	rs6517463	21	38380751	T	C	0.25	0.05	0.01	5.17E-07
PSRC1	rs34863121	1	109280836	T	C	0.01	0.71	0.03	8.43E-45
PSRC1	rs709049	20	32851058	G	T	0.55	-0.06	0.01	1.30E-07
PSRC1	rs6070696	20	59022590	G	A	0.18	0.10	0.01	1.63E-10
PTGDS	-	9	136964561	C	T	0.53	0.19	0.01	3.49E-72
PTGDS	rs12934455	16	20345959	T	C	0.17	-0.07	0.01	2.06E-06
PTGDS	rs704	17	28367840	A	G	0.47	0.05	0.01	1.23E-05
PTH1R	rs121434601	3	46898097	T	C	0.00	2.26	0.07	2.90E-95

PTH1R	rs428232	16	89647561	C	T	0.46	0.05	0.01	2.45E-05
PTK7	-	6	43069227	A	T	0.03	0.18	0.03	6.59E-06
PTN	rs28672722	6	32658760	T	G	0.22	0.06	0.01	6.07E-06
PTN	rs57122790	6	160729145	A	G	0.29	0.07	0.01	5.35E-09
PTN	rs10271620	7	137332456	A	G	0.43	0.15	0.01	1.24E-42
PTN	-	8	105561296	C	A	0.27	-0.06	0.01	9.81E-06
PTN	-	9	133274293	A	C	0.18	-0.10	0.01	3.01E-11
PTN	rs1654425	19	55027612	C	T	0.83	0.07	0.01	4.28E-06
PTPN1	rs1354034	3	56815721	C	T	0.60	0.08	0.01	3.35E-13
PTPN1	rs342293	7	106731773	G	C	0.46	-0.05	0.01	1.34E-05
PTPN1	rs7080536	10	113588287	A	G	0.04	-0.19	0.02	6.79E-12
PTPN6	rs1354034	3	56815721	C	T	0.60	0.06	0.01	1.15E-06
PTPN6	rs342298	7	106733200	T	C	0.45	-0.07	0.01	3.64E-10
PTPRF	rs2842194	1	43563291	A	G	0.22	-0.09	0.01	2.67E-11
PTPRF	rs2090034	2	20172648	A	G	0.47	0.06	0.01	6.19E-08
PTPRF	rs9873618	3	171015287	A	G	0.29	-0.06	0.01	1.44E-07
PTPRF	rs4860974	4	68598436	C	G	0.28	-0.06	0.01	1.62E-05
PTPRF	rs17039766	4	109085276	A	C	0.06	0.29	0.02	2.74E-34
PTPRF	rs2308527	6	31272044	T	G	0.59	-0.06	0.01	1.65E-07
PTPRF	rs581080	9	15305380	C	G	0.82	0.06	0.01	9.97E-06
PTPRF	rs507666	9	133273983	A	G	0.18	-0.08	0.01	5.00E-09
PTPRF	rs7088799	10	63256414	G	T	0.41	0.07	0.01	6.12E-10
PTPRF	rs9633740	10	80505515	G	A	0.80	0.14	0.01	1.12E-23
PTPRF	rs1535	11	61830500	G	A	0.35	0.05	0.01	4.63E-06
PTPRF	rs11045856	12	21197755	G	T	0.24	0.09	0.01	1.60E-13
PTPRF	rs28929474	14	94378610	T	C	0.02	0.33	0.02	2.93E-18
PTPRF	rs1801689	17	66214462	C	A	0.03	-0.22	0.02	2.06E-11
PTPRF	rs1065853	19	44909976	T	G	0.08	0.09	0.01	2.30E-05
PTPRF	rs679574	19	48702851	G	C	0.51	-0.10	0.01	1.75E-19
PTPRM	rs61804211	1	161701516	T	G	0.10	0.10	0.01	3.88E-09
PTPRM	rs13107325	4	102267552	T	C	0.07	0.11	0.01	1.43E-09
PTPRM	rs9270047	6	32585920	C	T	0.43	-0.05	0.01	3.14E-06
PTPRM	rs2519093	9	133266456	T	C	0.18	-0.70	0.01	0.00E+00
PTPRM	rs12359178	10	17847614	A	G	0.14	0.08	0.01	2.98E-08
PTPRM	-	10	80546526	A	T	0.33	-0.05	0.01	1.47E-05
PTPRM	rs11603123	11	126435600	A	G	0.03	0.18	0.02	9.93E-11
PTPRM	rs186021206	17	7166093	A	G	0.01	0.66	0.04	8.87E-23
PTPRM	rs72911294	18	8253604	T	C	0.27	-0.08	0.01	3.42E-11
PTPRM	rs5757659	22	39416404	G	T	0.69	-0.06	0.01	8.69E-08
PTPRN2	rs3800860	7	158110812	T	C	0.36	-0.15	0.01	8.11E-40
PTPRN2	rs113878851	16	20353760	T	C	0.17	-0.07	0.01	3.71E-06
PTPRS	rs1260326	2	27508073	C	T	0.61	0.09	0.01	8.26E-16
PTPRS	-	3	136181921	A	T	0.70	0.08	0.01	1.11E-11
PTPRS	rs545971	9	133267960	T	C	0.32	0.06	0.01	2.54E-06
PTPRS	rs144268704	15	63500022	G	A	0.34	0.08	0.01	1.40E-11
PTPRS	rs186021206	17	7166093	A	G	0.01	0.42	0.05	7.21E-08
PTPRS	rs77542162	17	69085137	G	A	0.02	0.16	0.02	2.75E-05
PTPRS	rs55763631	19	5293572	A	C	0.05	0.65	0.02	6.06E-120
PTS	rs183616935	3	52504351	C	A	0.01	0.47	0.05	4.49E-10
PTS	rs112875651	8	125494452	A	G	0.39	-0.05	0.01	7.07E-06
PTS	rs12796145	11	112205864	C	G	0.23	-0.24	0.01	1.63E-69
PTS	rs738408	22	43928850	T	C	0.22	0.08	0.01	1.22E-09
PTX3	rs678	3	52786965	T	A	0.36	-0.08	0.01	2.61E-10
PTX3	rs1056522	3	126542502	A	G	0.30	0.22	0.01	2.20E-74
PTX3	rs56025932	3	157438069	C	A	0.04	-0.16	0.02	6.61E-08
PTX3	rs41341748	8	16155085	A	G	0.01	0.33	0.03	1.09E-09

PTX3	rs7084817	10	7732395	G	C	0.05	-0.63	0.02	8.84E-136
PVALB	rs72642137	1	2298478	T	C	0.21	0.05	0.01	1.77E-05
PVALB	rs3811444	1	247876149	T	C	0.33	-0.07	0.01	1.98E-11
PVALB	rs10820606	9	96430637	C	A	0.23	0.05	0.01	8.76E-06
PVALB	rs7896518	10	63344740	G	A	0.42	0.06	0.01	1.51E-09
PVALB	rs6580981	12	54329244	A	G	0.46	0.05	0.01	2.33E-07
PVALB	rs11553699	12	121779004	G	A	0.14	-0.06	0.01	2.19E-05
PVALB	rs11865642	16	8956780	C	A	0.19	0.05	0.01	1.51E-05
PVALB	rs4821544	22	36862461	C	T	0.30	-0.81	0.01	0.00E+00
PVR	rs573252567	7	73589224	A	T	0.11	-0.08	0.01	5.68E-07
PVR	rs200489612	17	7203059	A	G	0.01	0.32	0.04	8.15E-06
PVR	rs2301274	19	44643102	C	T	0.24	-0.78	0.01	0.00E+00
PXN	rs3129754	6	32615269	G	A	0.42	0.05	0.01	5.52E-07
PXN	rs4767884	12	120224174	C	T	0.77	0.76	0.01	0.00E+00
PXN	rs4632248	19	53821741	T	G	0.21	0.07	0.01	1.08E-07
QDPR	rs1354034	3	56815721	C	T	0.60	0.06	0.01	1.03E-07
QDPR	-	4	17512134	C	G	0.25	-0.45	0.01	6.75E-254
QPCT	rs188468174	1	24965206	T	C	0.01	-0.38	0.03	1.30E-15
QPCT	rs61747728	1	179557079	T	C	0.04	0.13	0.02	1.50E-05
QPCT	rs4670696	2	37372820	C	A	0.02	-0.88	0.02	3.80E-123
QPCT	rs4634107	3	186623604	T	G	0.52	0.07	0.01	6.70E-10
QPCT	rs1130389	6	32664850	T	C	0.15	-0.08	0.01	1.65E-06
QPCT	rs111632177	7	151234719	A	G	0.11	0.09	0.01	1.11E-07
QPCT	rs13277237	8	129592317	A	G	0.57	-0.07	0.01	2.24E-09
QPCT	rs56278466	10	17833858	G	T	0.66	0.09	0.01	1.30E-13
QPCT	rs10896045	11	65788053	G	A	0.70	-0.15	0.01	7.33E-35
QPCT	rs3803800	17	7559652	G	A	0.79	-0.06	0.01	2.37E-05
QPCT	rs34562254	17	16939677	A	G	0.10	0.12	0.01	1.12E-10
QPCT	-	17	39988115	T	A	0.54	-0.06	0.01	1.09E-06
QPCT	rs2725405	17	81246424	C	G	0.44	0.05	0.01	8.58E-06
RAB6A	rs5030062	3	186736391	C	A	0.37	0.19	0.01	3.46E-68
RAB6A	rs4861708	4	186236079	A	G	0.51	0.39	0.01	1.68E-271
RAB6A	rs1801020	5	177409531	G	A	0.74	0.20	0.01	2.68E-62
RAB6A	rs35939600	11	73840098	T	A	0.15	0.07	0.01	3.29E-06
RAB6A	rs821840	16	56959974	G	A	0.33	0.07	0.01	1.34E-08
RABEPK	rs11788700	9	125220102	A	G	0.15	0.21	0.01	1.45E-35
RABGAP1L	rs6661868	1	174107629	G	A	0.20	0.09	0.01	1.48E-10
RABGAP1L	rs1354034	3	56815721	C	T	0.60	0.06	0.01	1.63E-06
RAD23B	rs1354034	3	56815721	C	T	0.60	0.05	0.01	1.52E-05
RAD23B	rs6993770	8	105569300	T	A	0.29	-0.06	0.01	5.12E-06
RAD23B	rs7035725	9	107287633	G	C	0.55	-0.06	0.01	5.86E-08
RARRES1	rs6441224	3	158732628	C	T	0.53	0.46	0.01	0.00E+00
RARRES2	rs780094	2	27518370	C	T	0.62	-0.05	0.01	1.80E-05
RARRES2	rs1265905	6	31942164	G	A	0.13	-0.10	0.01	1.16E-08
RARRES2	rs3735167	7	150342466	T	C	0.26	0.21	0.01	4.66E-64
RASSF2	rs1104902	20	4823485	A	C	0.57	0.09	0.01	5.90E-16
RASSF2	rs575652543	20	44990397	T	C	0.17	-0.07	0.01	1.83E-05
RBKS	rs140948699	2	27858572	G	C	0.01	-1.81	0.04	3.69E-214
RBP2	rs1375131	2	135197227	C	T	0.26	0.06	0.01	9.53E-06
RBP2	rs295469	3	139494058	A	G	0.38	-0.07	0.01	4.15E-09
RBP2	rs6472539	8	70717575	C	G	0.51	-0.06	0.01	1.61E-07
RBP2	rs11605127	11	268430	G	C	0.24	0.11	0.01	2.90E-16
RBP5	rs746441505	6	116455480	C	T	0.41	0.07	0.01	1.29E-10
RBP5	rs112875651	8	125494452	A	G	0.39	-0.05	0.01	3.93E-06
RBP5	rs1110236	9	93140313	A	G	0.13	-0.09	0.01	2.39E-08
RBP5	rs11370710	9	114396677	A	C	0.49	-0.05	0.01	1.89E-06

RBP5	rs76904513	12	7125522	G	A	0.09	-0.51	0.01	1.54E-154
RCOR1	rs7214731	17	66216248	T	C	0.28	0.06	0.01	1.25E-06
REG1A	rs11126696	2	79096762	G	A	0.62	0.25	0.01	6.66E-109
REG1A	rs9274437	6	32665397	T	C	0.25	0.06	0.01	1.02E-06
REG1A	rs529565	9	133274084	C	T	0.32	0.14	0.01	1.85E-33
REG1A	rs4421693	10	122546912	T	G	0.58	0.18	0.01	4.04E-63
REG1A	rs708686	19	5840608	T	C	0.27	0.10	0.01	1.71E-17
REG1A	rs679574	19	48702851	G	C	0.51	-0.19	0.01	3.16E-66
REG1B	rs11126696	2	79096762	G	A	0.62	0.34	0.01	1.61E-202
REG1B	rs529565	9	133274084	C	T	0.32	0.16	0.01	7.20E-43
REG1B	rs4421693	10	122546912	T	G	0.58	0.11	0.01	4.56E-26
REG1B	rs17802036	12	68273489	T	C	0.29	-0.05	0.01	2.24E-05
REG1B	rs72802342	16	75200974	A	C	0.08	-0.19	0.01	1.24E-19
REG1B	rs708686	19	5840608	T	C	0.27	0.09	0.01	1.10E-13
REG1B	rs492602	19	48703160	G	A	0.51	-0.18	0.01	1.89E-64
REG3A	rs147616162	2	79168385	A	T	0.06	0.40	0.02	6.82E-68
REG3A	rs11707109	3	40873097	G	A	0.14	-0.07	0.01	2.54E-06
REG3A	rs35846682	6	41714748	T	C	0.09	0.13	0.01	9.69E-12
REG3A	rs12201703	6	105954433	A	G	0.08	-0.16	0.01	9.07E-16
REG3A	rs11761603	7	1247276	C	T	0.70	0.05	0.01	2.73E-05
REG3A	rs146649678	8	6925915	A	G	0.00	0.47	0.07	1.14E-05
REG3A	rs2048528	8	23516167	A	G	0.32	-0.06	0.01	1.20E-06
REG3A	-	12	31958971	C	T	0.44	0.06	0.01	1.62E-08
REG3A	rs17338413	15	75182595	T	C	0.08	-0.35	0.01	6.03E-69
REG3A	rs11701433	21	38924487	T	C	0.67	-0.05	0.01	1.27E-05
REG4	rs147859708	1	8809045	A	G	0.03	-0.18	0.03	5.43E-06
REG4	rs79795228	1	119816663	A	C	0.02	0.63	0.03	2.32E-51
REG4	rs587654685	1	143975430	T	C	0.01	0.48	0.04	8.03E-14
REG4	rs137872268	1	149005418	C	T	0.02	0.26	0.03	9.04E-10
REG4	-	8	11807586	T	A	0.58	-0.05	0.01	9.91E-06
RELT	rs1398018	4	76451770	C	T	0.44	0.05	0.01	2.72E-06
RELT	rs56801796	11	73379220	G	A	0.21	0.40	0.01	7.60E-218
RELT	rs3184504	12	111446804	C	T	0.52	-0.06	0.01	3.90E-09
RELT	rs77924615	16	20381010	A	G	0.20	-0.07	0.01	3.05E-07
REN	rs112009694	1	163677855	T	C	0.55	-0.06	0.01	3.28E-07
REN	rs193280350	1	204179521	A	G	0.01	0.84	0.04	2.34E-38
REN	rs116219813	2	203216952	T	C	0.13	0.10	0.01	3.47E-09
RET	rs6747755	2	100961996	A	G	0.22	0.06	0.01	4.74E-06
RET	rs6762552	3	98712363	T	C	0.69	-0.08	0.01	1.71E-12
RET	rs7856189	9	98878916	C	T	0.61	-0.05	0.01	1.14E-05
RET	rs189906184	10	38695562	T	A	0.02	0.39	0.02	3.65E-25
RET	rs2795507	10	42857446	C	T	0.79	-0.44	0.01	9.50E-228
RET	rs35887873	11	126349501	C	T	0.23	0.09	0.01	1.92E-11
RET	rs186021206	17	7166093	A	G	0.01	0.45	0.05	1.09E-09
RET	rs9952412	18	48939170	C	A	0.52	0.05	0.01	2.33E-05
RETN	rs3917932	1	36478315	G	C	0.58	-0.06	0.01	1.43E-08
RETN	rs17405635	2	43128624	A	G	0.26	0.09	0.01	1.04E-13
RETN	rs2305637	3	47004356	T	C	0.18	0.07	0.01	5.67E-07
RETN	rs9880192	3	128578726	C	G	0.41	-0.05	0.01	1.24E-05
RETN	rs218264	4	54542708	T	A	0.25	0.06	0.01	1.42E-06
RETN	rs13107325	4	102267552	T	C	0.08	0.11	0.01	7.88E-07
RETN	rs149110519	6	144064640	T	C	0.04	0.39	0.02	5.21E-40
RETN	rs445	7	92779056	T	C	0.09	-0.11	0.01	1.09E-08
RETN	rs4407843	8	129597122	C	A	0.57	-0.08	0.01	4.09E-14
RETN	rs2074038	11	44066439	T	G	0.10	0.11	0.01	9.65E-10
RETN	rs11642657	16	85982722	T	C	0.01	0.31	0.04	4.22E-06

RETN	rs2241244	17	39997301	C	G	0.54	-0.11	0.01	8.20E-23
RETN	rs3219175	19	7668969	A	G	0.01	1.53	0.05	1.86E-106
RETN	rs6069597	20	38294325	C	T	0.53	-0.06	0.01	7.94E-08
RETN	rs243591	21	17783489	C	A	0.48	-0.06	0.01	4.43E-07
RGMA	rs147440039	1	146039631	G	C	0.03	-0.15	0.02	1.62E-06
RGMA	rs10800097	1	165414556	G	A	0.37	-0.05	0.01	2.25E-05
RGMA	rs1260326	2	27508073	C	T	0.61	0.08	0.01	2.97E-13
RGMA	rs6804368	3	30712081	G	A	0.44	-0.07	0.01	2.15E-10
RGMA	rs13147041	4	82951522	T	C	0.36	-0.05	0.01	6.79E-06
RGMA	rs13107325	4	102267552	T	C	0.08	-0.09	0.01	5.79E-06
RGMA	rs11956061	5	126910206	T	C	0.48	0.07	0.01	7.27E-11
RGMA	rs4841132	8	9326086	G	A	0.91	-0.09	0.01	8.30E-06
RGMA	rs1885372	9	35838033	G	A	0.41	-0.28	0.01	2.01E-142
RGMA	rs657152	9	133263862	A	C	0.34	0.05	0.01	2.40E-05
RGMA	rs7303452	12	50913366	C	T	0.66	-0.05	0.01	1.95E-05
RGMA	rs705702	12	55996852	G	A	0.34	0.06	0.01	1.55E-07
RGMA	rs68109100	13	73614638	G	A	0.50	0.05	0.01	1.91E-05
RGMA	rs4775282	15	60645725	T	A	0.35	-0.06	0.01	7.72E-07
RGMA	rs56058910	15	72833697	C	T	0.14	0.08	0.01	3.59E-07
RGMA	rs3752102	15	93072785	A	C	0.47	-0.23	0.01	9.17E-99
RGMA	rs71149134	16	20347025	T	C	0.17	-0.06	0.01	2.44E-05
RGMA	rs117353460	16	89240295	T	C	0.03	0.16	0.02	6.08E-08
RGMA	rs2904766	17	39692424	G	A	0.67	0.06	0.01	2.87E-07
RGMA	rs73597479	19	35054481	T	C	0.04	0.12	0.02	2.46E-05
RGMB	rs61747728	1	179557079	T	C	0.04	0.14	0.02	1.55E-06
RGMB	rs56870134	5	98437819	T	C	0.55	0.17	0.01	5.85E-53
RGMB	rs765574776	5	177371069	G	C	0.26	0.06	0.01	1.85E-05
RGMB	rs12359178	10	17847614	A	G	0.14	0.08	0.01	1.52E-07
RGMB	rs34262842	16	20344489	G	A	0.17	-0.09	0.01	6.79E-09
RGS8	rs34917868	14	106618321	A	G	0.14	0.24	0.01	7.65E-47
RHOC	rs1006298	1	112519337	C	G	0.48	0.08	0.01	1.73E-12
RHOC	rs1354034	3	56815721	C	T	0.60	0.06	0.01	9.28E-08
RHOC	rs342293	7	106731773	G	C	0.46	-0.05	0.01	7.77E-06
RILP	rs1354034	3	56815721	C	T	0.60	0.08	0.01	1.63E-12
RILP	rs6993770	8	105569300	T	A	0.29	-0.07	0.01	3.04E-08
RILP	rs58932608	11	265602	G	A	0.25	0.06	0.01	1.64E-05
RILP	rs183827902	17	1649225	T	C	0.02	0.26	0.03	4.03E-09
RNASE3	rs28498283	2	43132926	T	A	0.25	0.06	0.01	3.15E-06
RNASE3	rs75004383	2	64859095	A	G	0.04	0.13	0.02	1.03E-05
RNASE3	rs10208293	2	102349850	A	G	0.28	-0.05	0.01	2.07E-05
RNASE3	rs9269219	6	32482556	C	T	0.58	0.05	0.01	1.17E-05
RNASE3	-	9	6152128	C	A	0.32	0.06	0.01	5.30E-06
RNASE3	rs147307766	14	20917832	T	C	0.05	0.56	0.02	1.31E-111
RNASET2	rs61747728	1	179557079	T	C	0.04	0.13	0.02	2.36E-06
RNASET2	rs751785721	1	182160297	T	A	0.33	-0.05	0.01	4.83E-06
RNASET2	rs56193304	2	202779814	G	T	0.14	0.07	0.01	6.72E-06
RNASET2	rs13107325	4	102267552	T	C	0.08	0.12	0.01	8.41E-09
RNASET2	rs6914950	6	32622587	T	C	0.15	0.26	0.01	8.83E-64
RNASET2	rs3756838	6	166957763	A	G	0.21	0.46	0.01	1.22E-259
RNASET2	rs2721961	8	115645684	G	T	0.28	0.05	0.01	1.76E-05
RNASET2	rs12359178	10	17847614	A	G	0.14	0.07	0.01	1.40E-06
RNASET2	rs149871778	12	8946399	C	G	0.10	0.09	0.01	3.55E-07
RNASET2	rs11111026	12	101799025	T	G	0.18	-0.17	0.01	1.83E-37
RNASET2	rs111338191	12	111388673	T	A	0.52	-0.06	0.01	3.07E-09
RNASET2	rs145078947	14	93186629	T	G	0.00	1.32	0.07	3.30E-39
ROBO1	rs12752838	1	8853597	G	A	0.51	0.06	0.01	2.58E-07

ROBO1	-	1	161676997	A	C	0.08	0.09	0.01	1.78E-05
ROBO1	-	1	199025567	T	A	0.73	0.08	0.01	1.01E-10
ROBO1	rs3773244	3	78735620	A	G	0.20	0.23	0.01	2.16E-62
ROBO1	rs10935478	3	98709311	T	C	0.44	-0.06	0.01	3.94E-07
ROBO1	rs3849768	5	39418785	A	C	0.21	0.06	0.01	3.79E-06
ROBO1	rs9271325	6	32614736	G	C	0.47	-0.08	0.01	3.01E-13
ROBO1	rs9987289	8	9325848	G	A	0.91	-0.08	0.01	2.18E-05
ROBO1	-	9	133255468	T	G	0.06	0.15	0.02	5.33E-10
ROBO1	rs56278466	10	17833858	G	T	0.66	0.12	0.01	7.82E-25
ROBO1	-	11	61774534	G	A	0.35	0.05	0.01	1.91E-05
ROBO1	rs111338191	12	111388673	T	A	0.52	-0.06	0.01	9.55E-08
ROBO1	rs186021206	17	7166093	A	G	0.01	0.67	0.05	1.20E-18
ROBO1	rs77542162	17	69085137	G	A	0.02	0.19	0.02	5.62E-07
ROBO2	rs775722	3	77594465	C	T	0.45	-0.11	0.01	1.61E-22
ROBO2	rs67370636	6	32556186	T	C	0.33	0.08	0.01	2.14E-10
ROBO2	rs6984305	8	9320758	T	A	0.89	-0.10	0.01	1.41E-09
ROBO2	rs56278466	10	17833858	G	T	0.66	0.06	0.01	1.37E-07
ROBO2	rs186021206	17	7166093	A	G	0.01	0.49	0.05	1.79E-11
ROBO2	rs77542162	17	69085137	G	A	0.02	0.15	0.02	2.57E-05
ROR1	rs7532110	1	47496292	A	T	0.80	0.07	0.01	2.61E-06
ROR1	rs6588083	1	64148328	C	T	0.17	-0.39	0.01	3.86E-155
ROR1	rs150816167	1	179602727	C	T	0.04	0.20	0.02	6.10E-13
ROR1	rs1260326	2	27508073	C	T	0.61	0.06	0.01	4.94E-08
ROR1	rs75166367	2	162107791	A	G	0.06	-0.10	0.01	8.22E-06
ROR1	rs13107325	4	102267552	T	C	0.08	0.10	0.01	3.57E-06
ROR1	rs111239279	7	155877481	C	T	0.02	-0.18	0.03	1.01E-05
ROR1	rs2298475	11	126408308	C	T	0.07	-0.10	0.01	1.80E-06
ROR1	rs77924615	16	20381010	A	G	0.20	-0.08	0.01	1.68E-08
ROR1	rs186021206	17	7166093	A	G	0.01	0.46	0.05	5.46E-10
ROR1	rs2659005	17	81244914	T	C	0.44	0.06	0.01	6.37E-09
ROR1	rs33950747	19	35848345	T	C	0.08	0.13	0.01	1.16E-10
RP2	rs6479457	9	93148226	C	T	0.25	-0.06	0.01	2.11E-05
RP2	rs429358	19	44908684	C	T	0.16	0.14	0.01	2.59E-18
RRM2	-	6	32643672	G	T	0.18	-0.08	0.01	1.48E-06
RRM2	rs111338191	12	111388673	T	A	0.52	-0.06	0.01	2.44E-06
RRM2	rs7247412	19	51810615	C	T	0.27	-0.07	0.01	9.08E-07
RRM2B	rs1354034	3	56815721	C	T	0.60	0.09	0.01	1.89E-14
RRM2B	rs5893603	8	102238611	G	C	0.08	0.19	0.01	8.35E-19
RRM2B	-	10	63156270	A	T	0.49	0.06	0.01	1.75E-06
RRM2B	rs544626513	11	180302	C	G	0.23	0.06	0.01	2.02E-05
RSPO1	rs36043533	1	37613845	G	T	0.06	0.47	0.02	4.15E-86
RSPO1	rs13412535	2	224010157	A	G	0.23	-0.06	0.01	7.48E-06
RSPO1	rs11130124	3	47250693	C	T	0.59	-0.05	0.01	2.57E-05
RSPO1	rs1354034	3	56815721	C	T	0.60	-0.06	0.01	3.69E-07
RSPO1	rs6993770	8	105569300	T	A	0.29	-0.09	0.01	1.25E-12
RSPO1	rs61469632	9	132986603	C	T	0.06	-0.11	0.01	5.68E-06
RSPO1	rs3184504	12	111446804	C	T	0.52	-0.06	0.01	5.90E-09
RSPO1	rs12445050	16	81837364	T	C	0.14	0.08	0.01	3.46E-07
RSPO1	rs892090	19	55027704	G	T	0.83	0.10	0.01	2.76E-12
RSPO1	rs13651	20	45345439	C	T	0.51	-0.09	0.01	1.31E-14
RSPO3	rs1936800	6	127114919	T	C	0.53	0.16	0.01	1.88E-49
RSPO3	rs11751347	6	160671406	T	C	0.10	-0.12	0.01	1.20E-11
RSPO3	rs6993770	8	105569300	T	A	0.29	-0.06	0.01	1.88E-07
RSPO3	rs9543413	13	73609701	C	T	0.56	0.05	0.01	1.34E-05
RSPO3	rs8050794	16	3693045	C	T	0.70	0.06	0.01	6.00E-08
RSPO3	rs77924615	16	20381010	A	G	0.20	-0.06	0.01	2.40E-06

RSPO3	rs892090	19	55027704	G	T	0.83	0.08	0.01	1.21E-07
RSPO3	rs2741454	20	45345679	T	C	0.50	-0.06	0.01	3.43E-08
RTBDN	rs7687767	4	56958766	G	A	0.18	0.07	0.01	3.25E-06
RTBDN	rs7255045	19	12821455	A	G	0.26	-0.17	0.01	8.31E-43
RTBDN	rs1065853	19	44909976	T	G	0.08	0.09	0.01	1.44E-05
RTN4R	rs6658843	1	172429942	C	G	0.61	-0.06	0.01	5.75E-08
RTN4R	rs1260326	2	27508073	C	T	0.61	-0.06	0.01	4.59E-07
RTN4R	rs1047891	2	210675783	A	C	0.31	0.06	0.01	1.86E-07
RTN4R	rs7644541	3	136426924	G	A	0.77	0.06	0.01	7.71E-06
RTN4R	-	6	24429112	C	T	0.00	-0.39	0.05	7.57E-06
RTN4R	rs4355646	6	32418777	T	C	0.40	0.05	0.01	2.51E-05
RTN4R	rs1461729	8	9329732	G	A	0.90	-0.08	0.01	3.35E-06
RTN4R	rs28929474	14	94378610	T	C	0.02	0.31	0.02	1.05E-16
RTN4R	rs56332871	15	96171587	A	C	0.27	0.05	0.01	1.29E-05
RTN4R	rs1065853	19	44909976	T	G	0.08	0.12	0.01	2.03E-09
RTN4R	rs663353	22	20187189	A	G	0.75	-0.30	0.01	8.55E-122
RUVBL1	rs6003229	22	22697904	C	G	0.41	-0.24	0.01	5.70E-82
RWDD1	rs1354034	3	56815721	C	T	0.60	0.07	0.01	1.88E-10
RWDD1	rs147899431	6	116571518	C	G	0.04	0.30	0.02	3.59E-28
RWDD1	rs11502185	11	180258	C	T	0.26	0.06	0.01	2.34E-05
RWDD1	rs60822569	12	54323724	C	T	0.55	0.05	0.01	2.76E-06
S100A11	rs3122301	1	152038249	A	T	0.04	0.32	0.02	3.34E-32
S100A11	rs4632248	19	53821741	T	G	0.21	0.23	0.01	5.77E-62
S100A12	rs3014874	1	153365467	A	G	0.26	-0.23	0.01	2.00E-77
S100A12	rs6992333	8	143919209	G	A	0.43	0.05	0.01	1.93E-05
S100A12	rs10982156	9	114325784	A	T	0.07	0.14	0.01	1.68E-09
S100A12	-	17	39988115	T	A	0.54	-0.06	0.01	5.17E-08
S100A12	rs4632248	19	53821741	T	G	0.21	0.17	0.01	2.04E-34
S100A16	rs9700928	1	153612510	C	A	0.49	-0.10	0.01	5.43E-18
S100A16	rs56398830	13	103049340	A	G	0.01	-0.30	0.04	9.23E-08
S100A16	rs601338	19	48703417	A	G	0.51	-0.08	0.01	2.01E-10
S100A4	rs34557489	1	153548238	C	T	0.05	0.36	0.02	1.53E-45
S100A4	rs6993770	8	105569300	T	A	0.29	-0.06	0.01	1.05E-05
S100A4	rs150813342	9	132989126	T	C	0.01	-0.35	0.04	3.78E-08
S100A4	rs3817621	19	12887391	C	G	0.24	-0.06	0.01	2.43E-05
S100A4	rs892090	19	55027704	G	T	0.83	0.07	0.01	2.42E-05
S100P	rs28498283	2	43132926	T	A	0.25	0.07	0.01	1.90E-07
S100P	-	4	6685222	T	G	0.05	-0.56	0.02	5.55E-107
S100P	rs7846314	8	60738272	T	A	0.19	0.08	0.01	8.88E-09
S100P	-	8	143956732	T	C	0.36	0.09	0.01	8.59E-13
S100P	rs6503533	17	40028327	T	C	0.62	-0.06	0.01	1.47E-06
S100P	rs7412	19	44908822	T	C	0.08	0.19	0.01	3.37E-19
S100P	rs4632248	19	53821741	T	G	0.21	0.07	0.01	1.63E-06
SAMD9L	rs1354034	3	56815721	C	T	0.60	0.05	0.01	1.32E-05
SAMD9L	rs17165120	7	93147778	C	A	0.07	-0.56	0.01	2.46E-143
SCAMP3	rs1142287	1	155260340	T	C	0.26	-0.08	0.01	3.02E-10
SCAMP3	rs1354034	3	56815721	C	T	0.60	0.08	0.01	2.59E-13
SCAMP3	rs342293	7	106731773	G	C	0.46	-0.06	0.01	5.38E-07
SCAMP3	rs6993770	8	105569300	T	A	0.29	-0.06	0.01	7.90E-06
SCARA5	rs1260326	2	27508073	C	T	0.61	0.05	0.01	1.76E-05
SCARA5	rs73724776	6	18010537	G	A	0.05	0.11	0.02	5.49E-06
SCARA5	rs2726951	8	27948266	T	C	0.27	-0.34	0.01	6.24E-175
SCARA5	rs8176719	9	133257521	C	T	0.34	0.10	0.01	2.79E-21
SCARA5	rs112771035	11	126355981	G	C	0.07	-0.11	0.01	1.43E-08
SCARA5	rs597808	12	111535554	G	A	0.52	0.04	0.01	2.50E-05
SCARA5	rs186021206	17	7166093	A	G	0.01	0.38	0.05	1.11E-07

SCARB1	rs113075502	14	105891588	A	G	0.23	0.07	0.01	1.64E-05
SCARB2	rs10935473	3	98698056	T	G	0.44	-0.05	0.01	3.27E-06
SCARB2	rs28563976	4	76176220	C	A	0.15	-0.31	0.01	5.17E-97
SCARB2	rs1042133	6	33080829	C	G	0.16	0.08	0.01	9.05E-08
SCARB2	rs13192569	6	122806452	A	G	0.14	-0.07	0.01	8.28E-06
SCARB2	rs62018815	15	50766657	A	G	0.03	-0.21	0.02	1.72E-10
SCARF1	rs3811444	1	247876149	T	C	0.33	-0.05	0.01	6.06E-06
SCARF1	rs342298	7	106733200	T	C	0.46	-0.05	0.01	1.62E-05
SCARF1	rs6993770	8	105569300	T	A	0.29	-0.12	0.01	2.61E-23
SCARF1	-	9	4773337	C	T	0.54	-0.05	0.01	6.76E-06
SCARF1	rs579459	9	133278724	C	T	0.21	-0.06	0.01	4.27E-06
SCARF1	rs3184504	12	111446804	C	T	0.52	-0.06	0.01	3.74E-09
SCARF1	rs2272011	17	1638896	A	G	0.59	-0.38	0.01	5.78E-237
SCARF1	rs892090	19	55027704	G	T	0.83	0.08	0.01	1.91E-08
SCARF2	rs61747728	1	179557079	T	C	0.04	0.13	0.02	9.51E-07
SCARF2	rs1260326	2	27508073	C	T	0.61	0.04	0.01	1.55E-05
SCARF2	rs765335847	2	171568895	A	T	0.25	0.07	0.01	2.60E-09
SCARF2	rs507666	9	133273983	A	G	0.19	-0.12	0.01	2.94E-22
SCARF2	rs4055121	11	126362442	T	C	0.14	-0.08	0.01	6.58E-08
SCARF2	rs215225	12	481379	G	A	0.43	0.05	0.01	6.04E-08
SCARF2	rs77924615	16	20381010	A	G	0.20	-0.06	0.01	3.49E-06
SCARF2	rs186021206	17	7166093	A	G	0.01	0.61	0.04	3.05E-20
SCARF2	rs9610447	22	20414601	T	C	0.75	0.34	0.01	2.39E-195
SCG2	rs665401	6	116936380	C	T	0.32	-0.07	0.01	1.00E-07
SCG2	rs635634	9	133279427	T	C	0.18	-0.07	0.01	2.07E-06
SCG2	rs77924615	16	20381010	A	G	0.20	-0.07	0.01	1.71E-06
SCG2	rs681343	19	48703205	T	C	0.51	0.07	0.01	9.28E-10
SCG3	rs61804164	1	161653235	C	G	0.12	0.07	0.01	4.14E-06
SCG3	rs6798507	3	71497888	A	G	0.64	0.05	0.01	3.51E-07
SCG3	rs781657	4	56912519	G	A	0.18	0.11	0.01	5.54E-17
SCG3	-	5	134520666	C	T	0.42	-0.05	0.01	1.09E-05
SCG3	rs111540411	6	26249389	A	T	0.37	0.05	0.01	1.03E-06
SCG3	rs3751198	12	103753429	G	A	0.60	0.06	0.01	1.21E-07
SCG3	rs2606134	15	51681453	C	G	0.76	0.70	0.01	0.00E+00
SCG3	rs186021206	17	7166093	A	G	0.01	0.43	0.04	3.92E-10
SCGB1A1	rs3765766	1	3718096	C	T	0.88	-0.07	0.01	2.00E-05
SCGB1A1	rs761160638	2	8677620	A	T	0.08	-0.11	0.01	3.87E-07
SCGB1A1	rs3856521	2	61178588	A	G	0.15	-0.06	0.01	2.45E-05
SCGB1A1	rs9874923	3	20060071	T	C	0.42	0.05	0.01	1.34E-05
SCGB1A1	-	3	169161781	G	C	0.09	-0.15	0.01	7.45E-16
SCGB1A1	rs1515498	3	189790513	G	A	0.36	-0.10	0.01	9.09E-21
SCGB1A1	rs182548370	4	74794493	A	C	0.01	0.26	0.04	1.31E-05
SCGB1A1	-	5	1310990	G	A	0.57	0.05	0.01	6.85E-07
SCGB1A1	rs887373	5	127118033	C	T	0.50	-0.05	0.01	6.24E-07
SCGB1A1	rs3210176	6	32660073	C	T	0.37	-0.06	0.01	1.33E-06
SCGB1A1	rs7773147	6	73491411	G	A	0.68	0.05	0.01	8.04E-06
SCGB1A1	rs372203682	7	98174104	T	A	0.55	-0.05	0.01	2.61E-05
SCGB1A1	rs7040029	9	114856934	C	T	0.71	-0.05	0.01	1.19E-05
SCGB1A1	rs7072547	10	110222640	A	G	0.18	-0.07	0.01	1.77E-06
SCGB1A1	rs10501135	11	34789633	C	T	0.41	-0.14	0.01	3.46E-37
SCGB1A1	rs3741240	11	62419070	A	G	0.35	-0.34	0.01	9.92E-193
SCGB1A1	rs7962469	12	51954475	G	A	0.65	-0.09	0.01	1.60E-14
SCGB1A1	rs7963771	12	114905687	T	C	0.34	-0.06	0.01	1.97E-06
SCGB1A1	rs1766143	14	36227136	G	A	0.31	-0.06	0.01	4.99E-07
SCGB1A1	rs7182041	15	50014386	C	A	0.44	0.08	0.01	2.61E-14
SCGB1A1	rs55826010	17	45601827	A	G	0.24	-0.07	0.01	1.17E-08

SCGB1A1	rs1997814	20	10666023	G	T	0.68	-0.06	0.01	5.98E-07
SCGB3A2	rs72691756	1	117574557	A	G	0.12	0.19	0.01	1.03E-27
SCGB3A2	rs56241824	2	9929198	A	G	0.37	0.06	0.01	1.52E-06
SCGB3A2	rs58235267	2	63050708	G	C	0.49	-0.07	0.01	8.09E-11
SCGB3A2	rs1515498	3	189790513	G	A	0.36	-0.13	0.01	6.67E-30
SCGB3A2	rs17717320	5	147826351	G	C	0.20	-0.25	0.01	3.33E-70
SCGB3A2	rs9350191	6	19842430	T	C	0.85	-0.09	0.01	4.90E-08
SCGB3A2	rs6927418	6	24827684	T	C	0.56	-0.05	0.01	5.47E-06
SCGB3A2	rs62405419	6	50819746	T	G	0.12	-0.08	0.01	2.21E-05
SCGB3A2	rs12666406	7	98161679	A	G	0.54	-0.06	0.01	1.89E-08
SCGB3A2	-	8	70993166	A	T	0.08	-0.09	0.01	1.25E-05
SCGB3A2	rs12570742	10	973374	T	C	0.52	-0.06	0.01	1.27E-07
SCGB3A2	rs11499809	11	34769196	T	C	0.41	0.06	0.01	4.63E-08
SCGB3A2	rs3741240	11	62419070	A	G	0.35	0.14	0.01	3.43E-32
SCGB3A2	rs7962469	12	51954475	G	A	0.65	-0.09	0.01	5.75E-16
SCGB3A2	-	14	36790980	A	T	0.19	-0.07	0.01	5.11E-07
SCGB3A2	rs2414000	15	50020271	G	T	0.43	0.06	0.01	6.43E-07
SCGB3A2	rs173539	16	56954132	T	C	0.33	0.06	0.01	9.55E-08
SCGB3A2	rs756719	16	72933464	T	C	0.28	-0.05	0.01	2.01E-05
SCGB3A2	rs1572053	20	46898021	G	A	0.62	0.05	0.01	2.65E-05
SCGB3A2	-	21	34694224	C	G	0.20	-0.07	0.01	4.79E-07
SCGB3A2	rs12167717	22	19839117	A	G	0.24	-0.07	0.01	7.72E-08
SCGN	rs6859236	5	151156083	A	G	0.51	-0.10	0.01	1.77E-19
SCGN	rs10946788	6	25660282	A	G	0.28	-0.09	0.01	1.33E-12
SCGN	rs13253288	8	58426297	C	G	0.66	0.06	0.01	1.05E-06
SCGN	rs782134971	9	133264504	G	C	0.25	-0.07	0.01	9.64E-07
SCGN	rs11043076	11	2202244	T	C	0.13	0.09	0.01	7.91E-08
SCGN	rs601338	19	48703417	A	G	0.51	-0.13	0.01	1.63E-31
SCLY	rs62196005	2	238098489	T	A	0.27	0.19	0.01	1.27E-56
SCLY	rs4835265	4	145900258	A	C	0.16	0.07	0.01	1.55E-06
SCLY	rs10733608	9	114386150	T	G	0.49	-0.05	0.01	2.07E-05
SCLY	rs10883451	10	100164661	C	T	0.50	-0.05	0.01	2.95E-06
SCLY	rs58964858	11	94082917	T	C	0.11	-0.09	0.01	1.16E-06
SCLY	rs112635299	14	94371805	T	G	0.02	0.17	0.02	1.08E-05
SCLY	rs3747207	22	43928975	A	G	0.22	0.07	0.01	5.30E-08
SCP2	rs17200109	6	31410340	T	C	0.06	-0.11	0.02	1.45E-05
SCRN1	rs1434282	1	199041592	T	C	0.73	0.09	0.01	8.85E-13
SCRN1	rs17324153	7	29926525	C	T	0.04	0.27	0.02	2.38E-21
SDC1	rs907866	2	20171619	A	G	0.45	0.13	0.01	7.61E-32
SDC1	rs1260326	2	27508073	C	T	0.61	0.07	0.01	2.06E-10
SDC1	rs895893	3	136236762	T	C	0.77	0.06	0.01	2.70E-05
SDC1	rs13107325	4	102267552	T	C	0.08	0.33	0.01	3.02E-57
SDC1	rs28522260	7	1144328	A	C	0.17	-0.07	0.01	1.59E-06
SDC1	rs1461729	8	9329732	G	A	0.90	-0.11	0.01	7.06E-09
SDC1	rs17409194	8	19679679	G	A	0.42	-0.05	0.01	2.29E-05
SDC1	-	8	133585751	A	C	0.22	0.10	0.01	1.41E-14
SDC1	rs532436	9	133274414	A	G	0.19	-0.11	0.01	1.04E-13
SDC1	rs11438680	10	63414938	A	G	0.46	0.06	0.01	4.38E-08
SDC1	rs186021206	17	7166093	A	G	0.01	0.67	0.05	7.97E-19
SDC1	rs2204211	17	49196605	A	G	0.10	0.09	0.01	4.25E-07
SDC1	rs620982	18	32223308	C	T	0.46	-0.07	0.01	3.15E-11
SDC1	rs601338	19	48703417	A	G	0.51	0.07	0.01	4.15E-11
SDC1	rs2294915	22	43945024	T	C	0.23	0.06	0.01	1.57E-05
SDC4	rs6662263	1	171975102	G	A	0.80	0.07	0.01	1.45E-06
SDC4	rs1354034	3	56815721	C	T	0.60	-0.08	0.01	7.89E-12
SDC4	-	5	132328823	T	A	0.63	-0.06	0.01	2.97E-06

SDC4	rs2735095	6	29947989	A	C	0.32	-0.09	0.01	9.01E-13
SDC4	rs778641135	6	135097494	T	C	0.26	0.06	0.01	7.68E-06
SDC4	rs61177759	6	139526156	T	C	0.20	-0.06	0.01	1.28E-05
SDC4	rs11763765	7	1020874	T	C	0.15	-0.17	0.01	6.24E-28
SDC4	rs139141690	7	101856650	A	G	0.00	-0.44	0.05	1.05E-07
SDC4	rs6993770	8	105569300	T	A	0.29	-0.12	0.01	1.55E-22
SDC4	rs385893	9	4763176	C	T	0.52	0.09	0.01	6.83E-15
SDC4	rs61750929	9	88880220	T	C	0.06	-0.11	0.02	4.35E-06
SDC4	rs7861658	9	97917672	G	A	0.36	0.05	0.01	1.30E-05
SDC4	rs4962034	9	132991261	G	C	0.61	-0.05	0.01	4.47E-06
SDC4	rs58641111	10	80485200	T	C	0.47	-0.08	0.01	4.16E-13
SDC4	rs12762934	10	102600127	T	C	0.32	0.10	0.01	1.09E-17
SDC4	rs33972805	11	126418977	T	C	0.53	-0.05	0.01	2.65E-06
SDC4	rs11553699	12	121779004	G	A	0.14	0.17	0.01	1.25E-22
SDC4	rs12881760	14	100709998	C	G	0.67	0.05	0.01	8.05E-06
SDC4	rs34890846	16	8947636	G	A	0.19	-0.07	0.01	7.63E-07
SDC4	rs4783186	16	85382128	C	T	0.87	-0.08	0.01	4.70E-06
SDC4	rs7198986	16	88478414	G	C	0.35	-0.05	0.01	2.55E-05
SDC4	rs1654425	19	55027612	C	T	0.83	0.08	0.01	4.98E-07
SDC4	rs4355178	20	1950227	A	G	0.26	-0.06	0.01	6.35E-07
SDC4	rs2251577	20	45345931	C	G	0.51	-0.22	0.01	6.35E-87
SDC4	rs2229742	21	14966851	C	G	0.10	0.08	0.01	8.15E-06
SELE	rs1277930	1	109279521	A	G	0.77	0.07	0.01	1.69E-09
SELE	rs4987388	1	169689445	G	A	0.71	-0.10	0.01	2.51E-20
SELE	rs10935473	3	98698056	T	G	0.44	-0.09	0.01	2.03E-19
SELE	rs13135092	4	102276925	G	A	0.08	0.08	0.01	5.86E-06
SELE	rs2519093	9	133266456	T	C	0.18	-0.97	0.01	0.00E+00
SELE	-	9	137036493	A	G	0.64	-0.07	0.01	8.84E-10
SELE	rs35166255	11	126431861	A	G	0.03	0.32	0.02	2.19E-35
SELE	rs7979473	12	120982457	G	A	0.62	-0.05	0.01	3.96E-08
SELE	rs186021206	17	7166093	A	G	0.01	0.59	0.04	2.05E-19
SELE	rs17855739	19	5831829	T	C	0.04	0.17	0.02	5.25E-13
SELP	rs6136	1	169594713	G	T	0.11	-0.40	0.01	4.91E-114
SELP	rs56043070	1	247556467	A	G	0.07	0.11	0.01	3.82E-07
SELP	rs11242109	5	132341354	T	G	0.48	-0.06	0.01	3.36E-08
SELP	rs6961069	7	80589645	T	C	0.40	0.06	0.01	1.59E-06
SELP	rs6993770	8	105569300	T	A	0.29	-0.11	0.01	2.25E-20
SELP	rs385893	9	4763176	C	T	0.52	0.05	0.01	1.69E-05
SELP	rs2519093	9	133266456	T	C	0.18	-0.22	0.01	2.55E-51
SELP	rs7896518	10	63344740	G	A	0.42	0.07	0.01	1.37E-10
SELP	rs12445050	16	81837364	T	C	0.14	0.10	0.01	4.20E-10
SELP	rs892090	19	55027704	G	T	0.83	0.14	0.01	4.68E-20
SELPLG	rs343808	1	110787385	T	C	0.24	-0.07	0.01	1.17E-08
SELPLG	rs2990223	1	155215184	G	A	0.60	0.14	0.01	8.50E-39
SELPLG	rs10214273	5	35883884	G	T	0.27	-0.07	0.01	2.19E-08
SELPLG	rs4248814	6	31378978	A	G	0.20	0.07	0.01	4.01E-08
SELPLG	rs10093797	8	78660478	A	C	0.75	-0.06	0.01	6.03E-06
SELPLG	rs2142306	8	133458388	C	T	0.41	0.05	0.01	4.67E-06
SELPLG	rs536644	9	76530872	A	G	0.55	0.10	0.01	3.00E-20
SELPLG	rs10819317	9	127906678	G	A	0.17	0.10	0.01	2.06E-11
SELPLG	rs7294337	12	108618918	T	C	0.02	-1.97	0.03	0.00E+00
SELPLG	rs60005225	17	40011875	T	G	0.38	-0.05	0.01	6.78E-06
SELPLG	rs1840828	19	16331801	T	C	0.61	-0.05	0.01	1.89E-05
SELPLG	rs28823595	20	3724486	A	G	0.09	0.08	0.01	2.44E-05
SEMA3F	rs554375669	3	50143912	T	C	0.21	-0.17	0.01	5.19E-32
SEMA3F	rs4679317	3	126969428	A	G	0.69	-0.07	0.01	1.45E-09

SEMA3F	rs507666	9	133273983	A	G	0.18	-0.08	0.01	1.76E-08
SEMA3F	rs564699	11	89467705	T	C	0.54	-0.06	0.01	1.77E-08
SEMA3F	rs3184504	12	111446804	C	T	0.52	-0.06	0.01	4.03E-08
SEMA3F	rs1037117	15	101528455	A	G	0.25	0.06	0.01	1.00E-06
SEMA3F	rs8055162	16	89648874	A	G	0.57	-0.06	0.01	3.84E-08
SEMA4C	rs756513821	2	95553269	G	A	0.00	-1.00	0.12	2.88E-07
SEMA4C	rs537675308	2	99768494	C	G	0.00	-0.84	0.12	1.00E-05
SEMA4C	rs34794906	6	31270081	C	T	0.26	0.06	0.01	1.97E-06
SEMA4C	rs2519093	9	133266456	T	C	0.18	-0.26	0.01	7.53E-73
SEMA4C	rs564699	11	89467705	T	C	0.54	-0.06	0.01	1.28E-06
SEMA4C	rs186021206	17	7166093	A	G	0.01	0.49	0.05	3.38E-10
SEMA4C	rs909692	22	50272204	G	C	0.39	0.05	0.01	1.50E-05
SEMA4D	rs1354034	3	56815721	C	T	0.60	0.10	0.01	1.73E-18
SEMA4D	rs6993770	8	105569300	T	A	0.29	-0.05	0.01	2.36E-05
SEMA4D	rs409801	9	4744743	C	T	0.51	0.05	0.01	2.45E-05
SEMA4D	rs36020742	9	89436540	T	C	0.18	0.10	0.01	1.70E-12
SEMA4D	rs7080386	10	63288546	A	C	0.41	0.05	0.01	5.36E-06
SEMA4D	rs7949566	11	126415406	A	G	0.42	0.07	0.01	2.60E-08
SEMA4D	rs111338191	12	111388673	T	A	0.52	-0.09	0.01	3.13E-15
SEMA7A	rs61801010	1	161572445	G	A	0.11	0.09	0.01	2.27E-06
SEMA7A	rs7606173	2	60498316	C	G	0.43	-0.06	0.01	2.59E-08
SEMA7A	rs3864106	3	186938813	C	G	0.58	-0.18	0.01	5.82E-61
SEMA7A	rs28716466	4	207285	C	T	0.30	0.07	0.01	1.87E-08
SEMA7A	rs13135092	4	102276925	G	A	0.08	0.10	0.01	3.15E-06
SEMA7A	rs9263850	6	31188411	G	A	0.28	0.06	0.01	2.71E-06
SEMA7A	rs9489686	6	119328000	G	A	0.42	-0.06	0.01	9.15E-08
SEMA7A	rs2347784	7	6485212	G	C	0.27	-0.08	0.01	3.39E-11
SEMA7A	rs12554596	9	37002145	G	A	0.20	-0.06	0.01	7.39E-06
SEMA7A	rs10793962	9	133253728	T	A	0.06	0.22	0.01	1.16E-22
SEMA7A	rs56278466	10	17833858	G	T	0.66	0.16	0.01	1.28E-43
SEMA7A	rs73119306	12	57433199	G	A	0.25	0.06	0.01	8.12E-06
SEMA7A	rs3184504	12	111446804	C	T	0.52	-0.12	0.01	3.00E-26
SEMA7A	rs608394	12	120756250	T	C	0.51	-0.08	0.01	6.96E-12
SEMA7A	rs78994380	15	74400071	A	C	0.11	-0.23	0.01	1.27E-37
SEMA7A	rs200489612	17	7203059	A	G	0.01	0.68	0.05	1.33E-16
SEMA7A	rs34557412	17	16948873	G	A	0.01	0.56	0.04	6.15E-17
SEMA7A	rs485186	19	48703949	G	A	0.54	0.05	0.01	1.31E-05
SEMA7A	rs7258315	19	52904912	C	G	0.22	-0.06	0.01	1.99E-05
SEPTIN9	rs55645755	17	77456611	G	A	0.02	-0.37	0.03	5.29E-15
SEPTIN9	rs10432210	18	25281830	T	C	0.21	0.06	0.01	1.02E-05
SERPINA11	rs1260326	2	27508073	C	T	0.61	-0.07	0.01	1.73E-13
SERPINA11	-	3	186624022	C	A	0.51	0.06	0.01	1.24E-10
SERPINA11	rs13108218	4	3442204	G	A	0.62	-0.04	0.01	1.08E-05
SERPINA11	rs144861591	6	26072764	T	C	0.08	-0.07	0.01	1.65E-05
SERPINA11	-	8	10718441	A	T	0.60	0.06	0.01	9.32E-10
SERPINA11	rs3999089	10	63444048	G	A	0.47	0.06	0.01	9.41E-11
SERPINA11	rs7139079	12	120977490	A	G	0.59	-0.04	0.01	9.24E-07
SERPINA11	rs12898137	14	94444245	A	T	0.41	-0.61	0.01	0.00E+00
SERPINA11	rs7188155	16	88460014	G	C	0.31	0.04	0.01	1.05E-05
SERPINA11	rs8105397	19	45894381	G	A	0.12	-0.10	0.01	6.69E-13
SERPINA12	rs759945735	7	100056468	T	A	0.29	-0.05	0.01	3.24E-07
SERPINA12	rs1495743	8	18415790	C	G	0.78	-0.05	0.01	5.69E-06
SERPINA12	-	11	61853156	A	C	0.40	-0.05	0.01	5.69E-06
SERPINA12	rs17091005	14	94522070	C	T	0.22	0.77	0.01	0.00E+00
SERPINA12	rs339969	15	60591082	A	C	0.62	-0.10	0.01	7.98E-24
SERPINA12	rs72999033	19	19255823	T	C	0.06	0.09	0.01	3.74E-06

SERPINA12	rs738408	22	43928850	T	C	0.22	0.06	0.01	9.35E-07
SERPINA9	rs9264954	6	31307618	A	G	0.11	0.07	0.01	4.82E-06
SERPINA9	rs10849448	12	6384185	G	A	0.75	-0.05	0.01	1.62E-05
SERPINA9	rs11850199	14	94476326	A	C	0.27	-0.50	0.01	0.00E+00
SERPINB1	rs17622656	5	132485305	A	G	0.39	-0.05	0.01	1.83E-05
SERPINB1	rs6925281	6	2815043	T	C	0.18	0.11	0.01	2.22E-12
SERPINB1	rs6993770	8	105569300	T	A	0.29	-0.08	0.01	1.61E-10
SERPINB5	rs148729762	18	63493861	A	C	0.66	0.06	0.01	4.79E-06
SERPINB6	rs9405601	6	2972663	A	G	0.44	0.16	0.01	6.35E-46
SERPINB6	rs6993770	8	105569300	T	A	0.29	-0.06	0.01	1.28E-06
SERPINB8	rs3826616	18	63987229	G	A	0.58	-0.72	0.01	0.00E+00
SERPINB8	rs4632248	19	53821741	T	G	0.21	0.10	0.01	4.28E-19
SERPINB9	rs7751676	6	2931645	T	C	0.06	0.22	0.02	3.96E-21
SERPINB9	rs6993770	8	105569300	T	A	0.29	-0.07	0.01	5.95E-08
SERPINE1	rs2274319	1	156481081	C	T	0.65	-0.06	0.01	1.38E-06
SERPINE1	rs13412535	2	224010157	A	G	0.23	-0.06	0.01	6.21E-06
SERPINE1	rs78909033	2	240571486	A	G	0.14	-0.08	0.01	8.66E-07
SERPINE1	rs7618405	3	18209017	A	C	0.21	-0.06	0.01	1.50E-05
SERPINE1	rs1354034	3	56815721	C	T	0.60	-0.12	0.01	2.71E-25
SERPINE1	rs4698839	4	101880784	T	C	0.40	-0.06	0.01	6.20E-07
SERPINE1	rs114694170	5	88884379	C	T	0.06	0.14	0.02	4.11E-09
SERPINE1	rs11242109	5	132341354	T	G	0.48	-0.06	0.01	1.32E-06
SERPINE1	rs6961069	7	80589645	T	C	0.40	0.07	0.01	2.29E-08
SERPINE1	rs2227674	7	101132927	G	A	0.20	-0.12	0.01	3.31E-16
SERPINE1	rs6993770	8	105569300	T	A	0.29	-0.16	0.01	2.30E-38
SERPINE1	rs61469632	9	132986603	C	T	0.06	-0.14	0.02	3.90E-09
SERPINE1	rs34377578	10	102576669	C	A	0.25	0.07	0.01	3.39E-08
SERPINE1	rs6580981	12	54329244	A	G	0.46	0.06	0.01	2.35E-08
SERPINE1	rs60128101	15	64868190	A	T	0.18	0.07	0.01	6.14E-06
SERPINE1	rs12445050	16	81837364	T	C	0.14	0.10	0.01	1.57E-09
SERPINE1	rs1654425	19	55027612	C	T	0.83	0.16	0.01	2.93E-26
SERPINE1	rs6081565	20	19307260	A	G	0.35	0.07	0.01	3.48E-09
SESTD1	rs10170520	2	179229886	T	A	0.33	-0.10	0.01	1.77E-17
SESTD1	rs1354034	3	56815721	C	T	0.60	0.06	0.01	3.59E-06
SESTD1	rs10733789	10	63188924	C	T	0.31	0.06	0.01	9.58E-07
SETMAR	rs547990685	1	10631051	G	C	0.00	0.52	0.08	1.61E-05
SETMAR	rs35083095	3	4362134	C	T	0.49	-0.22	0.01	5.37E-88
SEZ6L	rs61801010	1	161572445	G	A	0.11	0.10	0.01	9.61E-08
SEZ6L	rs781657	4	56912519	G	A	0.18	0.06	0.01	5.79E-06
SEZ6L	rs9269915	6	32583676	T	G	0.72	0.06	0.01	1.13E-06
SEZ6L	rs10838702	11	47389337	T	G	0.39	0.05	0.01	3.62E-06
SEZ6L	rs60194243	11	126377404	T	C	0.06	-0.11	0.01	1.19E-06
SEZ6L	rs1070073	12	103606541	G	T	0.67	0.05	0.01	4.24E-06
SEZ6L	rs186021206	17	7166093	A	G	0.01	0.48	0.05	1.49E-10
SEZ6L	rs137203	22	26292865	T	G	0.02	-0.56	0.03	2.43E-46
SEZ6L2	rs1260326	2	27508073	C	T	0.61	0.05	0.01	2.09E-05
SEZ6L2	rs1961950	4	56954651	T	C	0.09	-0.18	0.01	9.18E-24
SEZ6L2	rs2523567	6	31362238	G	C	0.23	-0.07	0.01	3.74E-09
SEZ6L2	rs56287412	7	17939205	C	T	0.51	0.05	0.01	1.61E-05
SEZ6L2	rs56278466	10	17833858	G	T	0.66	0.06	0.01	2.72E-07
SEZ6L2	rs12372718	12	50777307	G	A	0.41	-0.08	0.01	4.56E-14
SEZ6L2	rs113443718	16	29880863	A	G	0.31	0.16	0.01	5.13E-48
SEZ6L2	rs186021206	17	7166093	A	G	0.01	0.66	0.05	1.42E-20
SF3B4	rs12078573	1	149931717	A	G	0.08	-0.10	0.01	7.05E-06
SF3B4	rs10067881	5	95826771	A	G	0.13	-0.11	0.01	6.14E-11
SF3B4	rs6993770	8	105569300	T	A	0.29	-0.06	0.01	5.87E-07

SF3B4	rs3782123	11	205198	A	C	0.73	0.06	0.01	2.53E-05
SFRP1	rs2318761	1	150285304	G	A	0.47	0.05	0.01	1.26E-06
SFRP1	rs3189152	6	32666564	G	A	0.42	-0.05	0.01	1.50E-05
SFRP1	rs72643819	8	41309674	T	G	0.39	-0.19	0.01	2.48E-64
SFRP1	rs6420185	8	143220901	A	G	0.39	-0.06	0.01	2.47E-08
SFRP1	-	9	133263362	G	A	0.18	-0.12	0.01	6.03E-17
SFRP1	rs2741450	20	45346489	A	T	0.51	-0.06	0.01	1.13E-08
SFTPA1	rs1965708	10	79557289	T	G	0.19	-0.28	0.01	4.36E-81
SFTPA2	rs11675149	2	203076319	A	C	0.57	-0.05	0.01	3.29E-06
SFTPA2	rs1004500	5	127132544	A	G	0.46	0.05	0.01	6.56E-07
SFTPA2	-	9	133255468	T	G	0.06	0.09	0.01	1.37E-05
SFTPA2	rs17886395	10	79558907	G	C	0.13	0.93	0.01	0.00E+00
SFTPA2	rs7962469	12	51954475	G	A	0.65	0.05	0.01	1.46E-06
SFTPA2	rs2753532	14	105893218	C	T	0.88	-0.10	0.01	1.30E-08
SFTPA2	rs117981338	17	73092704	T	C	0.01	0.25	0.03	8.11E-09
SFTPD	rs2075571	1	155204315	T	C	0.42	0.04	0.01	3.04E-06
SFTPD	rs56218657	1	207096104	A	G	0.04	-0.12	0.01	3.45E-07
SFTPD	rs751775696	4	57078846	A	T	0.53	-0.05	0.01	1.43E-07
SFTPD	rs2464520	4	88929868	G	C	0.82	-0.07	0.01	7.57E-10
SFTPD	-	5	95695443	C	A	0.55	-0.04	0.01	1.70E-05
SFTPD	rs4262138	5	129986982	T	A	0.14	0.06	0.01	5.21E-07
SFTPD	rs62395441	6	31379437	A	G	0.06	0.69	0.01	1.53E-275
SFTPD	rs2451951	9	106734349	C	T	0.49	-0.11	0.01	4.94E-37
SFTPD	rs721917	10	79946568	G	A	0.42	-0.75	0.01	0.00E+00
SFTPD	rs35705950	11	1219991	T	G	0.11	0.07	0.01	2.61E-06
SFTPD	rs4767280	12	114926148	T	C	0.40	0.04	0.01	1.65E-05
SFTPD	rs111345531	13	109265867	C	T	0.26	0.04	0.01	2.71E-05
SFTPD	rs58462329	16	84370147	C	G	0.15	-0.21	0.01	5.42E-67
SFTPD	rs199913382	17	46548500	C	A	0.22	-0.06	0.01	1.98E-07
SH2B3	rs11950562	5	132316836	C	A	0.48	-0.05	0.01	8.34E-06
SH2B3	rs342293	7	106731773	G	C	0.46	-0.06	0.01	1.57E-07
SH2B3	rs6993770	8	105569300	T	A	0.29	-0.09	0.01	5.91E-12
SH2B3	rs409801	9	4744743	C	T	0.51	0.05	0.01	1.48E-05
SH2B3	-	12	111473764	G	A	0.21	-0.08	0.01	1.59E-08
SH2D1A	rs3184504	12	111446804	C	T	0.52	-0.08	0.01	4.74E-12
SH2D1A	rs75763843	18	44482415	C	A	0.13	0.12	0.01	7.15E-13
SHMT1	rs1354034	3	56815721	C	T	0.60	0.05	0.01	1.30E-07
SHMT1	rs342296	7	106732457	A	G	0.46	-0.05	0.01	6.13E-06
SHMT1	rs6993770	8	105569300	T	A	0.29	-0.07	0.01	8.51E-12
SHMT1	rs60822569	12	54323724	C	T	0.55	0.04	0.01	2.10E-05
SHMT1	rs669340	17	18360643	C	G	0.63	0.71	0.01	0.00E+00
SIAE	rs114269697	1	64848529	A	C	0.01	-0.23	0.03	6.65E-06
SIAE	rs6993770	8	105569300	T	A	0.29	-0.06	0.01	3.15E-07
SIAE	rs56278466	10	17833858	G	T	0.66	0.08	0.01	7.95E-11
SIAE	rs9414801	10	63389329	A	G	0.53	-0.09	0.01	8.76E-16
SIAE	rs78778622	11	124660768	C	T	0.06	-0.44	0.02	1.27E-76
SIAE	rs2559850	12	101699681	A	G	0.59	0.07	0.01	4.10E-09
SIAE	rs3184504	12	111446804	C	T	0.52	-0.05	0.01	1.12E-05
SIAE	rs72701845	14	92750678	A	G	0.04	-0.15	0.02	9.70E-07
SIAE	rs892090	19	55027704	G	T	0.83	0.10	0.01	3.47E-10
SIGLEC1	rs17849502	1	183563445	T	G	0.05	0.11	0.02	3.50E-06
SIGLEC1	rs62165726	2	134208991	A	C	0.04	-0.40	0.02	1.37E-46
SIGLEC1	rs2111485	2	162254026	G	A	0.61	0.05	0.01	1.46E-05
SIGLEC1	rs115216147	5	71369699	A	T	0.07	0.11	0.01	5.38E-07
SIGLEC1	rs11185602	7	50259481	G	A	0.33	-0.06	0.01	1.24E-08
SIGLEC1	rs2031902	9	33117526	T	C	0.59	-0.07	0.01	6.43E-10

SIGLEC1	rs6590207	11	126446130	G	A	0.29	0.09	0.01	8.67E-14
SIGLEC1	rs3184504	12	111446804	C	T	0.52	-0.07	0.01	5.48E-11
SIGLEC1	rs186021206	17	7166093	A	G	0.01	1.32	0.05	6.36E-76
SIGLEC1	rs11668950	19	18172130	A	G	0.27	0.09	0.01	1.11E-13
SIGLEC1	rs189448562	20	3724468	C	G	0.01	-1.83	0.04	7.01E-259
SIGLEC10	rs1257169	2	134206291	A	C	0.50	-0.05	0.01	5.11E-06
SIGLEC10	rs4857410	3	98635069	T	C	0.15	-0.08	0.01	1.68E-07
SIGLEC10	rs634501	5	180791668	G	A	0.74	-0.06	0.01	1.22E-06
SIGLEC10	rs562289	6	32609269	T	C	0.23	0.07	0.01	9.67E-08
SIGLEC10	rs3184504	12	111446804	C	T	0.52	-0.11	0.01	2.16E-24
SIGLEC10	rs186021206	17	7166093	A	G	0.01	0.85	0.05	3.81E-31
SIGLEC10	rs34557412	17	16948873	G	A	0.01	0.44	0.04	1.94E-11
SIGLEC10	rs148783636	19	51407725	C	T	0.02	-1.57	0.03	1.27E-291
SIGLEC10	rs3747207	22	43928975	A	G	0.22	0.06	0.01	1.12E-05
SIGLEC5	rs7137828	12	111494996	T	C	0.52	-0.06	0.01	1.97E-10
SIGLEC5	rs186021206	17	7166093	A	G	0.01	0.45	0.04	1.37E-13
SIGLEC5	rs1106476	19	51627384	A	T	0.11	-1.21	0.01	0.00E+00
SIGLEC6	rs115427247	1	92412850	T	C	0.03	-0.16	0.02	2.95E-07
SIGLEC6	rs4951018	1	205667206	C	A	0.23	-0.07	0.01	3.43E-08
SIGLEC6	rs74227709	1	247559286	A	G	0.07	0.14	0.01	2.57E-12
SIGLEC6	rs3111414	2	8303729	G	C	0.80	-0.12	0.01	2.08E-20
SIGLEC6	rs7573683	2	60353959	C	T	0.54	-0.05	0.01	1.43E-05
SIGLEC6	rs4384720	2	144667834	G	C	0.92	0.11	0.01	1.81E-09
SIGLEC6	rs773890649	3	69801056	T	C	0.21	0.05	0.01	1.94E-05
SIGLEC6	rs1516527	3	148891917	C	T	0.95	0.22	0.02	1.35E-20
SIGLEC6	rs576666495	3	158217690	G	A	0.00	-0.44	0.06	1.49E-06
SIGLEC6	rs13107325	4	102267552	T	C	0.08	0.12	0.01	1.70E-09
SIGLEC6	rs4863714	4	139986183	G	A	0.64	0.05	0.01	9.69E-07
SIGLEC6	rs622076	6	31858928	A	G	0.16	0.12	0.01	4.73E-18
SIGLEC6	rs2299876	6	119216358	G	A	0.41	-0.08	0.01	9.80E-14
SIGLEC6	rs10441775	9	91719626	G	A	0.69	0.05	0.01	1.68E-05
SIGLEC6	rs56278466	10	17833858	G	T	0.66	0.05	0.01	4.27E-06
SIGLEC6	rs2155311	11	126421580	A	G	0.48	-0.11	0.01	4.47E-24
SIGLEC6	rs3184504	12	111446804	C	T	0.52	-0.07	0.01	3.64E-12
SIGLEC6	rs4454799	12	120879272	T	G	0.47	-0.06	0.01	2.47E-10
SIGLEC6	rs76428106	13	28029870	C	T	0.01	0.49	0.03	7.58E-26
SIGLEC6	rs2745083	16	1234173	A	C	0.51	-0.11	0.01	3.25E-28
SIGLEC6	rs62045817	16	88978825	T	C	0.15	-0.07	0.01	1.25E-06
SIGLEC6	rs146261845	17	7108935	T	C	0.01	0.42	0.04	3.03E-10
SIGLEC6	rs78744187	19	33263642	T	C	0.08	0.15	0.01	9.15E-13
SIGLEC6	rs62617068	19	51531295	A	C	0.01	-1.76	0.03	1.56E-271
SIGLEC7	rs1257169	2	134206291	A	C	0.50	-0.06	0.01	3.60E-07
SIGLEC7	rs11721064	3	98689982	T	G	0.60	-0.05	0.01	2.26E-06
SIGLEC7	rs4393849	3	194757682	G	A	0.31	0.05	0.01	9.44E-06
SIGLEC7	rs11220462	11	126374057	A	G	0.13	-0.08	0.01	2.49E-07
SIGLEC7	rs3184504	12	111446804	C	T	0.52	-0.07	0.01	8.72E-10
SIGLEC7	rs186021206	17	7166093	A	G	0.01	0.96	0.05	6.20E-39
SIGLEC7	rs2160725	17	68399585	C	A	0.49	-0.08	0.01	1.22E-13
SIGLEC7	rs60202083	19	47268927	A	T	0.42	-0.06	0.01	3.21E-08
SIGLEC7	rs140185670	19	51142883	C	G	0.07	-0.63	0.01	2.09E-170
SIGLEC9	rs61830291	1	220827800	C	A	0.10	-0.07	0.01	2.31E-05
SIGLEC9	rs2593704	2	134247706	C	G	0.78	-0.06	0.01	2.64E-08
SIGLEC9	rs10935473	3	98698056	T	G	0.44	-0.63	0.01	0.00E+00
SIGLEC9	rs1468906	3	186930328	G	A	0.34	-0.05	0.01	2.81E-06
SIGLEC9	rs9260	6	32642684	G	A	0.81	-0.11	0.01	5.76E-23
SIGLEC9	rs4841132	8	9326086	G	A	0.91	-0.09	0.01	1.50E-09

SIGLEC9	-	9	33140495	T	A	0.31	0.09	0.01	2.94E-19
SIGLEC9	rs507666	9	133273983	A	G	0.18	-0.24	0.01	8.40E-95
SIGLEC9	rs78689694	11	126364925	C	G	0.13	-0.20	0.01	3.37E-52
SIGLEC9	rs10774625	12	111472415	G	A	0.50	-0.04	0.01	2.53E-05
SIGLEC9	rs186021206	17	7166093	A	G	0.01	1.55	0.04	3.39E-144
SIGLEC9	rs2075803	19	51125272	G	A	0.55	-0.57	0.01	0.00E+00
SIRPA	rs61747728	1	179557079	T	C	0.04	0.07	0.01	6.97E-06
SIRPA	rs1260326	2	27508073	C	T	0.61	0.03	0.00	4.08E-07
SIRPA	rs34458031	3	99033191	A	G	0.09	0.07	0.01	9.19E-12
SIRPA	rs9269915	6	32583676	T	G	0.72	-0.03	0.00	9.20E-06
SIRPA	rs2519093	9	133266456	T	C	0.18	-0.08	0.00	1.26E-23
SIRPA	rs186021206	17	7166093	A	G	0.01	0.42	0.03	1.87E-25
SIRPA	rs2659005	17	81244914	T	C	0.44	0.03	0.00	2.54E-06
SIRPA	rs6136377	20	1915642	G	A	0.38	-1.22	0.01	0.00E+00
SIRPB1	rs61747728	1	179557079	T	C	0.04	0.13	0.02	2.46E-08
SIRPB1	rs1260326	2	27508073	C	T	0.61	0.04	0.01	8.71E-06
SIRPB1	rs7735073	5	132479665	A	G	0.33	-0.05	0.01	1.56E-06
SIRPB1	rs13277237	8	129592317	A	G	0.57	-0.05	0.01	1.40E-07
SIRPB1	rs3184504	12	111446804	C	T	0.52	-0.08	0.01	1.09E-18
SIRPB1	rs76428106	13	28029870	C	T	0.01	0.27	0.03	1.29E-10
SIRPB1	rs777834943	16	20355409	T	C	0.18	-0.06	0.01	4.23E-06
SIRPB1	rs186021206	17	7166093	A	G	0.01	0.36	0.04	1.60E-08
SIRPB1	rs6503533	17	40028327	T	C	0.62	-0.05	0.01	6.07E-08
SIRPB1	rs2777895	17	59802249	G	A	0.55	0.08	0.01	8.32E-18
SIRPB1	rs7224668	17	81261988	C	T	0.56	-0.05	0.01	2.60E-07
SIRPB1	rs79462675	20	1616529	A	C	0.13	-0.95	0.01	0.00E+00
SIRT2	rs1354034	3	56815721	C	T	0.60	0.06	0.01	5.37E-08
SIRT2	rs342293	7	106731773	G	C	0.46	-0.07	0.01	1.03E-08
SIRT2	-	10	63156270	A	T	0.49	0.06	0.01	2.94E-08
SIRT2	rs11502185	11	180258	C	T	0.26	0.06	0.01	1.44E-05
SIRT2	rs144373891	19	38889130	T	C	0.01	-0.70	0.04	3.96E-32
SIT1	rs3184504	12	111446804	C	T	0.52	-0.10	0.01	1.38E-17
SIT1	rs10468017	15	58386313	T	C	0.30	-0.06	0.01	9.37E-07
SIT1	rs71374020	16	30641303	T	A	0.30	-0.06	0.01	1.21E-05
SIT1	rs12446515	16	56953103	T	C	0.32	-0.08	0.01	2.84E-11
SIT1	rs4613118	17	7394133	G	A	0.86	-0.08	0.01	1.79E-06
SIT1	rs7208422	17	78134494	T	A	0.48	-0.08	0.01	1.44E-12
SIT1	rs10469472	19	17376117	G	T	0.12	0.10	0.01	9.14E-08
SKAP1	rs28718232	6	32658973	G	A	0.21	0.07	0.01	1.40E-06
SKAP1	rs17323934	7	26864711	G	C	0.23	-0.09	0.01	4.74E-12
SKAP1	rs3176789	12	9760134	T	C	0.28	-0.06	0.01	1.37E-05
SKAP1	rs7310615	12	111427245	G	C	0.52	-0.08	0.01	4.06E-11
SKAP1	rs71374020	16	30641303	T	A	0.30	-0.06	0.01	7.81E-06
SKAP1	rs5817082	16	56963437	A	C	0.27	0.06	0.01	1.08E-05
SKAP1	rs12603827	17	48250540	T	C	0.45	0.05	0.01	1.09E-05
SKAP1	rs7208422	17	78134494	T	A	0.48	-0.07	0.01	1.99E-09
SKAP1	rs74460666	19	17410292	G	T	0.12	0.09	0.01	4.65E-07
SKAP2	rs1354034	3	56815721	C	T	0.60	0.07	0.01	1.07E-08
SKAP2	rs10900809	5	132490630	A	G	0.39	-0.06	0.01	3.75E-06
SKAP2	rs6993770	8	105569300	T	A	0.29	-0.07	0.01	1.61E-07
SLAMF1	rs37454	1	43828739	A	C	0.35	0.06	0.01	2.24E-07
SLAMF1	rs7535367	1	160668460	T	G	0.14	-0.14	0.01	6.61E-17
SLAMF1	rs11710456	3	187008099	A	G	0.28	-0.06	0.01	2.99E-06
SLAMF1	rs9264669	6	31271904	T	A	0.57	0.07	0.01	2.44E-08
SLAMF1	rs35350651	12	111469627	C	A	0.50	-0.05	0.01	2.56E-05
SLAMF1	rs186021206	17	7166093	A	G	0.01	0.77	0.05	1.67E-22

SLAMF6	rs11265413	1	160490309	A	T	0.20	-0.21	0.01	2.75E-51
SLAMF6	rs3184504	12	111446804	C	T	0.52	-0.07	0.01	2.24E-08
SLAMF6	rs186021206	17	7166093	A	G	0.01	0.44	0.05	5.45E-08
SLAMF7	rs188468174	1	24965206	T	C	0.01	-0.55	0.03	1.64E-29
SLAMF7	rs11581248	1	160750284	T	C	0.14	-0.94	0.01	0.00E+00
SLAMF7	rs4972973	2	230873083	A	G	0.47	-0.06	0.01	5.09E-08
SLAMF7	rs7621161	3	187009382	A	C	0.28	-0.13	0.01	5.30E-31
SLAMF7	rs3094087	6	31093784	C	T	0.17	-0.10	0.01	4.89E-13
SLAMF7	rs3780480	9	33163488	T	G	0.25	-0.05	0.01	6.38E-06
SLAMF7	rs111338191	12	111388673	T	A	0.52	-0.06	0.01	1.57E-07
SLAMF7	rs186021206	17	7166093	A	G	0.01	0.95	0.05	9.82E-42
SLAMF7	rs34562254	17	16939677	A	G	0.10	0.09	0.01	1.46E-07
SLAMF7	rs8077394	17	81284987	G	A	0.55	-0.05	0.01	3.02E-07
SLAMF7	-	22	30037182	G	T	0.45	-0.05	0.01	2.84E-06
SLAMF8	rs34687326	1	159830120	A	G	0.12	-1.34	0.01	0.00E+00
SLAMF8	rs2190504	7	30467764	C	T	0.44	0.05	0.01	1.09E-06
SLAMF8	rs12667978	7	129137407	T	C	0.57	0.05	0.01	5.01E-06
SLAMF8	rs3184504	12	111446804	C	T	0.52	-0.10	0.01	5.12E-20
SLAMF8	rs186021206	17	7166093	A	G	0.01	0.45	0.05	1.65E-09
SLC16A1	rs6681552	1	112891643	A	G	0.53	-0.06	0.01	1.45E-07
SLC16A1	rs3811444	1	247876149	T	C	0.33	-0.10	0.01	5.67E-17
SLC16A1	rs13135472	4	143989465	C	T	0.69	0.07	0.01	8.15E-08
SLC16A1	rs590856	6	139523292	A	G	0.55	0.09	0.01	8.46E-16
SLC27A4	rs1354034	3	56815721	C	T	0.60	0.10	0.01	4.16E-16
SLC27A4	rs137948800	9	128307438	A	T	0.12	0.08	0.01	3.45E-06
SLC27A4	rs7080536	10	113588287	A	G	0.04	-0.19	0.02	9.16E-11
SLC27A4	rs58512066	11	266315	C	T	0.24	0.06	0.01	2.77E-05
SLC39A14	rs782470771	7	73608603	A	G	0.13	-0.08	0.01	1.15E-05
SLC39A14	rs35486529	8	22394355	T	C	0.26	-0.11	0.01	5.20E-16
SLC39A14	-	12	21191379	G	A	0.24	0.10	0.01	2.98E-12
SLC39A14	rs28929474	14	94378610	T	C	0.02	0.31	0.03	2.45E-14
SLC39A5	rs12065546	1	230159498	T	C	0.85	0.08	0.01	1.32E-06
SLC39A5	rs9266089	6	31352732	A	G	0.18	-0.09	0.01	1.49E-09
SLC39A5	rs7786376	7	73628284	G	A	0.28	-0.07	0.01	3.23E-08
SLC39A5	rs2272662	8	144414342	C	T	0.58	-0.06	0.01	2.30E-07
SLC39A5	rs147572362	12	56231330	C	T	0.01	-0.70	0.04	4.32E-28
SLC39A5	rs111349624	12	123288246	C	A	0.08	0.09	0.01	4.04E-06
SLC39A5	rs28929474	14	94378610	T	C	0.02	0.34	0.03	3.72E-18
SLC39A5	rs11078597	17	1715069	C	T	0.19	0.07	0.01	5.63E-06
SLC39A5	-	19	35062059	T	A	0.93	0.10	0.01	1.77E-05
SLC39A5	rs55953905	21	41510744	T	C	0.25	0.10	0.01	2.64E-14
SLIT2	rs1185160	4	20198728	T	G	0.70	-0.06	0.01	6.39E-07
SLITRK2	rs61830291	1	220827800	C	A	0.10	-0.09	0.01	9.55E-06
SLITRK2	rs62262391	3	123386272	T	C	0.22	0.07	0.01	7.22E-08
SLITRK2	rs68119427	4	56891864	G	C	0.35	0.05	0.01	1.28E-05
SLITRK2	rs13107325	4	102267552	T	C	0.08	0.26	0.01	5.44E-36
SLITRK2	rs78334147	5	109681535	T	C	0.10	0.11	0.01	1.51E-08
SLITRK2	rs2811708	9	21973423	T	G	0.26	0.07	0.01	4.96E-08
SLITRK2	rs1131773	9	93077974	G	A	0.14	0.07	0.01	8.05E-06
SLITRK2	rs56278466	10	17833858	G	T	0.66	0.07	0.01	1.88E-10
SLITRK2	rs10823396	10	69483567	G	C	0.46	0.05	0.01	9.72E-06
SLITRK2	rs12805381	11	11254541	T	G	0.45	0.08	0.01	3.17E-13
SLITRK2	rs774868556	12	89431449	A	A	0.31	-0.09	0.01	1.00E-11
SLITRK2	rs186021206	17	7166093	A	G	0.01	0.43	0.05	1.93E-08
SLITRK6	rs61747728	1	179557079	T	C	0.04	0.14	0.02	1.88E-06
SLITRK6	rs1260326	2	27508073	C	T	0.61	0.07	0.01	1.51E-08

SLITRK6	rs13107325	4	102267552	T	C	0.07	0.10	0.01	1.60E-06
SLITRK6	rs7712631	5	154230042	A	G	0.42	0.09	0.01	7.54E-15
SLITRK6	rs149556327	9	93046096	A	G	0.07	0.09	0.01	2.09E-05
SLITRK6	-	9	133274293	A	C	0.18	-0.07	0.01	1.17E-06
SLITRK6	rs36115375	10	7735836	A	C	0.06	-0.15	0.01	1.71E-10
SLITRK6	rs56278466	10	17833858	G	T	0.66	0.07	0.01	1.48E-08
SLITRK6	rs12863734	13	85796436	A	G	0.02	-1.40	0.03	1.03E-223
SMAD1	rs1260326	2	27508073	C	T	0.61	-0.05	0.01	1.28E-05
SMAD1	rs1354034	3	56815721	C	T	0.60	0.05	0.01	9.87E-06
SMAD1	rs342293	7	106731773	G	C	0.46	-0.06	0.01	1.01E-07
SMAD1	rs6993770	8	105569300	T	A	0.29	-0.07	0.01	3.28E-08
SMAD1	rs964184	11	116778201	C	G	0.87	-0.08	0.01	1.45E-05
SMAD5	rs4643790	4	153788702	A	G	0.20	-0.21	0.01	2.36E-52
SMAD5	rs3129777	6	32678407	A	C	0.21	-0.08	0.01	4.83E-08
SMAD5	-	9	133263362	G	A	0.18	-0.19	0.01	2.83E-39
SMOC1	rs2090034	2	20172648	A	G	0.47	-0.06	0.01	1.30E-07
SMOC1	rs35457250	3	186620775	T	C	0.01	0.31	0.04	4.41E-08
SMOC1	rs11755689	6	32618599	G	A	0.33	0.06	0.01	2.09E-07
SMOC1	rs2960834	7	1163320	C	G	0.45	0.05	0.01	1.73E-06
SMOC1	rs705379	7	95324583	A	G	0.48	0.08	0.01	3.26E-13
SMOC1	-	11	116752497	T	A	0.86	-0.08	0.01	3.80E-08
SMOC1	rs1958078	14	69888141	C	A	0.85	0.37	0.01	6.92E-139
SMOC1	rs28929474	14	94378610	T	C	0.02	0.18	0.02	2.18E-06
SMOC1	rs77542162	17	69085137	G	A	0.02	0.45	0.02	8.16E-40
SMOC1	rs584007	19	44913221	G	A	0.64	0.06	0.01	7.16E-08
SMOC1	rs3830725	20	45336067	T	C	0.24	-0.06	0.01	2.73E-06
SMOC1	rs4239651	20	47711852	C	T	0.79	-0.07	0.01	6.91E-08
SMOC2	rs4708467	6	168424579	T	C	0.28	-0.39	0.01	6.28E-247
SMOC2	rs56408342	8	22190977	A	G	0.05	-0.11	0.01	5.00E-07
SMOC2	rs4759276	12	57132863	A	G	0.39	0.05	0.01	1.27E-06
SMOC2	rs77924615	16	20381010	A	G	0.20	-0.06	0.01	7.00E-06
SMOC2	rs7247412	19	51810615	C	T	0.27	-0.12	0.01	6.53E-25
SMPD1	rs78444298	1	184702964	A	G	0.02	-0.21	0.02	1.01E-07
SMPD1	-	4	87292522	A	T	0.43	-0.05	0.01	1.94E-06
SMPD1	rs114816312	4	109717668	T	C	0.01	0.33	0.04	4.91E-08
SMPD1	rs2389688	4	118815641	C	A	0.44	-0.05	0.01	6.32E-06
SMPD1	rs77945361	4	153333382	A	G	0.15	-0.08	0.01	2.88E-07
SMPD1	-	6	130042457	C	T	0.57	0.05	0.01	6.69E-07
SMPD1	rs199922514	8	9325592	G	A	0.91	0.08	0.01	1.78E-05
SMPD1	rs11791806	9	34633532	T	C	0.17	0.06	0.01	4.62E-06
SMPD1	rs772851187	10	63147821	T	G	0.47	0.08	0.01	5.56E-15
SMPD1	rs12257692	10	72944091	C	T	0.04	-0.11	0.02	2.77E-05
SMPD1	rs2862954	10	100152307	C	T	0.50	-0.06	0.01	1.22E-07
SMPD1	rs1050239	11	6394233	A	G	0.24	-0.44	0.01	1.01E-270
SMPD1	rs10778152	12	101831973	G	A	0.29	-0.34	0.01	1.77E-188
SMPD1	rs150641790	13	41113708	G	T	0.07	0.12	0.01	3.42E-09
SMPD1	rs71435622	14	39242324	T	A	0.29	0.07	0.01	3.84E-08
SMPD1	rs112635299	14	94371805	T	G	0.02	0.34	0.02	1.23E-21
SMPD1	rs139974673	15	43735687	C	T	0.02	0.16	0.02	2.17E-06
SMPD1	-	15	60595685	G	A	0.62	0.07	0.01	9.96E-10
SMPD1	rs58542926	19	19268740	T	C	0.08	0.16	0.01	5.65E-16
SMPD1	rs429358	19	44908684	C	T	0.16	-0.10	0.01	2.15E-11
SMPD1	rs11668882	19	54171328	C	T	0.44	0.06	0.01	5.65E-08
SMPD1	rs3747207	22	43928975	A	G	0.22	0.07	0.01	3.37E-08
SMPDL3A	rs114165349	1	26695422	C	G	0.02	0.18	0.02	8.93E-09
SMPDL3A	rs2642438	1	220796686	G	A	0.70	0.04	0.01	1.57E-05

SMPDL3A	rs687339	3	136213517	T	C	0.77	0.09	0.01	1.27E-16
SMPDL3A	rs114816312	4	109717668	T	C	0.01	0.25	0.03	2.39E-06
SMPDL3A	rs114667001	4	118865819	C	T	0.06	-0.10	0.01	1.33E-06
SMPDL3A	rs41280463	4	153270074	A	G	0.17	-0.07	0.01	2.96E-09
SMPDL3A	rs2522063	5	132469899	T	G	0.18	0.05	0.01	8.06E-06
SMPDL3A	rs567092558	5	173120385	T	C	0.14	-0.06	0.01	3.58E-06
SMPDL3A	rs28385609	6	122801319	T	C	0.14	-1.01	0.01	0.00E+00
SMPDL3A	rs9414801	10	63389329	A	G	0.53	-0.06	0.01	4.37E-10
SMPDL3A	-	10	72768827	T	C	0.04	-0.13	0.01	2.01E-08
SMPDL3A	rs2862954	10	100152307	C	T	0.50	-0.06	0.01	9.47E-12
SMPDL3A	rs10787429	10	112189906	C	T	0.73	-0.06	0.01	4.60E-08
SMPDL3A	rs10128858	12	101827588	G	A	0.29	-0.41	0.01	0.00E+00
SMPDL3A	rs2464190	12	120977587	C	T	0.41	0.04	0.01	1.16E-05
SMPDL3A	rs150641790	13	41113708	G	T	0.07	0.13	0.01	4.91E-13
SMPDL3A	rs11622951	14	39253478	A	G	0.46	0.06	0.01	7.20E-12
SMPDL3A	rs145078947	14	93186629	T	G	0.00	0.79	0.06	1.53E-18
SMPDL3A	rs147233090	15	43735849	T	C	0.02	0.17	0.02	7.13E-08
SMPDL3A	-	15	60595685	G	A	0.62	0.05	0.01	8.41E-07
SMPDL3A	-	18	74259946	T	A	0.71	0.05	0.01	1.29E-05
SMPDL3A	rs58542926	19	19268740	T	C	0.08	0.19	0.01	2.74E-27
SMPDL3A	rs429358	19	44908684	C	T	0.16	-0.10	0.01	1.33E-15
SMPDL3A	rs626283	19	54173307	C	G	0.44	0.06	0.01	1.22E-09
SMPDL3A	rs738408	22	43928850	T	C	0.22	0.07	0.01	5.94E-10
SNAP23	rs1354034	3	56815721	C	T	0.60	0.11	0.01	8.43E-22
SNAP23	rs6993770	8	105569300	T	A	0.29	-0.06	0.01	7.17E-06
SNAP23	rs10820606	9	96430637	C	A	0.23	-0.08	0.01	2.07E-09
SNAP23	rs11502185	11	180258	C	T	0.26	0.08	0.01	1.82E-08
SNAP29	rs1354034	3	56815721	C	T	0.60	0.08	0.01	1.90E-11
SNAP29	rs11242109	5	132341354	T	G	0.48	-0.05	0.01	2.78E-06
SNAP29	rs6993770	8	105569300	T	A	0.29	-0.07	0.01	6.87E-09
SNAP29	rs892090	19	55027704	G	T	0.83	0.08	0.01	1.06E-06
SNAP29	rs5759874	22	20662861	G	A	0.09	-0.13	0.01	3.23E-10
SNCG	rs873110	10	86964862	T	C	0.24	-1.06	0.01	0.00E+00
SNX9	rs688167	6	157671616	G	C	0.71	-0.09	0.01	1.14E-12
SNX9	rs342298	7	106733200	T	C	0.46	-0.07	0.01	6.69E-10
SOD1	rs1354034	3	56815721	C	T	0.60	0.05	0.01	1.23E-05
SOD1	rs6993770	8	105569300	T	A	0.29	-0.11	0.01	1.02E-17
SOD1	rs7282332	21	31753926	A	G	0.10	0.09	0.01	1.04E-06
SOD2	rs1570805	1	156294857	T	C	0.11	0.09	0.01	1.84E-06
SOD2	rs3821884	3	45975107	G	T	0.53	-0.05	0.01	2.58E-05
SOD2	rs9272548	6	32639185	C	T	0.19	-0.07	0.01	1.31E-05
SOD2	rs732498	6	159670528	A	G	0.31	0.15	0.01	1.78E-38
SOD2	rs4541868	8	105578477	A	C	0.27	-0.05	0.01	1.61E-05
SOD2	rs9411378	9	133270015	A	C	0.22	0.06	0.01	2.00E-05
SOD2	rs117692263	12	7472418	C	T	0.08	0.16	0.01	1.55E-16
SOD2	rs2072694	16	1455428	G	C	0.52	0.05	0.01	6.93E-06
SOD2	rs12975366	19	54255498	C	T	0.40	-0.15	0.01	6.07E-43
SORCS2	rs4234798	4	7218206	G	T	0.62	-0.46	0.01	0.00E+00
SORCS2	rs4253238	4	186227233	T	C	0.51	-0.07	0.01	1.40E-10
SORCS2	rs1344684	12	89458895	G	A	0.71	0.05	0.01	4.65E-06
SORCS2	rs7203642	16	20355808	G	A	0.19	-0.07	0.01	8.42E-07
SORD	rs10733608	9	114386150	T	G	0.49	-0.05	0.01	2.26E-05
SORD	rs137936987	12	7394123	C	A	0.03	-0.28	0.02	6.35E-19
SORD	rs72722075	15	45031276	T	C	0.13	0.21	0.01	5.97E-35
SORD	rs3747207	22	43928975	A	G	0.22	0.07	0.01	3.61E-07
SORT1	rs61394658	1	109330668	A	G	0.23	-0.18	0.01	1.97E-42

SORT1	rs2079797	3	47300311	A	G	0.45	0.06	0.01	2.00E-06
SORT1	rs13303	3	52523992	C	T	0.56	0.07	0.01	5.76E-09
SORT1	-	3	58414616	G	A	0.37	0.06	0.01	1.47E-07
SORT1	rs139596721	4	153540161	T	C	0.03	0.16	0.02	1.29E-06
SORT1	rs4703589	5	72801524	C	T	0.53	0.05	0.01	6.76E-06
SORT1	rs4098923	6	31225979	G	A	0.45	-0.05	0.01	1.63E-05
SORT1	rs139141690	7	101856650	A	G	0.00	0.65	0.05	7.79E-15
SORT1	rs6993770	8	105569300	T	A	0.29	-0.18	0.01	1.05E-47
SORT1	rs385893	9	4763176	C	T	0.52	0.06	0.01	4.73E-07
SORT1	rs10984405	9	97928273	G	A	0.36	0.08	0.01	4.23E-11
SORT1	rs2519093	9	133266456	T	C	0.18	-0.09	0.01	3.06E-10
SORT1	rs774510679	10	63304076	A	C	0.41	0.09	0.01	2.74E-15
SORT1	rs17490626	10	69458890	C	G	0.13	0.16	0.01	6.52E-23
SORT1	rs3184504	12	111446804	C	T	0.52	-0.07	0.01	2.46E-10
SORT1	rs7176023	15	64808808	C	T	0.07	0.10	0.01	8.30E-06
SORT1	rs12445050	16	81837364	T	C	0.14	0.07	0.01	2.44E-05
SORT1	rs781416314	16	88477072	A	T	0.34	-0.05	0.01	2.34E-05
SORT1	rs892090	19	55027704	G	T	0.83	0.13	0.01	6.70E-18
SOST	rs4973180	2	229236796	C	T	0.18	-0.08	0.01	6.46E-08
SOST	rs17138646	5	116010548	G	T	0.12	0.11	0.01	5.86E-12
SOST	rs66642874	6	44793317	C	T	0.22	-0.07	0.01	1.61E-07
SOST	rs34115816	6	133047963	A	G	0.32	0.08	0.01	2.99E-13
SOST	rs73169672	7	151250992	T	A	0.11	0.09	0.01	1.07E-07
SOST	rs11995824	8	119000461	G	C	0.54	-0.10	0.01	1.62E-22
SOST	-	11	87170008	C	T	0.32	0.06	0.01	1.04E-06
SOST	rs215226	12	482134	G	A	0.40	-0.20	0.01	1.82E-74
SOST	rs7968637	12	53409849	C	T	0.18	0.07	0.01	2.87E-06
SOST	rs34001253	13	42509157	G	A	0.03	0.19	0.02	2.91E-10
SOST	rs7325334	13	110458327	G	C	0.60	0.05	0.01	2.60E-05
SOST	rs34369065	15	50898548	T	C	0.51	-0.06	0.01	1.68E-08
SOST	rs1513671	17	43729942	C	G	0.64	-0.06	0.01	4.14E-08
SOST	rs884205	18	62387624	C	A	0.75	0.07	0.01	1.62E-08
SOST	rs34952318	20	11196407	A	G	0.05	-0.14	0.02	8.58E-09
SOST	rs6127099	20	54114863	T	A	0.28	-0.06	0.01	5.42E-06
SPARC	-	1	156911262	T	C	0.10	0.09	0.01	6.12E-06
SPARC	rs13412535	2	224010157	A	G	0.23	-0.07	0.01	1.89E-06
SPARC	rs7618405	3	18209017	A	C	0.20	-0.06	0.01	1.65E-05
SPARC	rs1354034	3	56815721	C	T	0.60	-0.10	0.01	7.08E-19
SPARC	rs755492124	4	101856385	T	G	0.39	-0.06	0.01	1.12E-07
SPARC	rs11242109	5	132341354	T	G	0.48	-0.05	0.01	1.70E-05
SPARC	rs576037440	5	151683029	A	G	0.00	-0.70	0.06	1.20E-12
SPARC	rs138614581	6	31436393	T	A	0.08	-0.10	0.01	1.79E-06
SPARC	rs1608696	6	70619562	C	T	0.31	-0.05	0.01	2.79E-05
SPARC	rs6961069	7	80589645	T	C	0.40	0.07	0.01	2.28E-08
SPARC	rs6993770	8	105569300	T	A	0.29	-0.12	0.01	2.23E-20
SPARC	-	10	63433569	C	T	0.49	-0.06	0.01	9.88E-08
SPARC	rs34377578	10	102576669	C	A	0.25	0.10	0.01	3.52E-15
SPARC	rs4573747	12	6181464	G	A	0.48	-0.05	0.01	2.61E-05
SPARC	rs35785237	12	54328275	A	C	0.43	0.06	0.01	8.55E-08
SPARC	rs2127869	14	65327634	C	T	0.71	0.05	0.01	2.74E-05
SPARC	rs59001897	15	64868193	A	T	0.17	0.07	0.01	1.50E-06
SPARC	rs28734133	16	81840430	G	A	0.18	0.08	0.01	2.48E-08
SPARC	rs1654425	19	55027612	C	T	0.83	0.17	0.01	2.92E-27
SPARC	rs3790176	20	19281278	A	G	0.34	0.06	0.01	3.51E-06
SPARCL1	rs4693826	4	87510740	C	T	0.36	0.71	0.01	0.00E+00
SPARCL1	-	9	133263362	G	A	0.18	-0.16	0.01	7.88E-39

SPARCL1	rs217184	16	72072066	C	T	0.20	-0.15	0.01	2.44E-34
SPARCL1	rs77542162	17	69085137	G	A	0.02	0.21	0.02	4.10E-11
SPARCL1	rs55639531	18	394341	G	C	0.29	0.05	0.01	1.41E-05
SPINK1	rs28817415	4	76480299	T	C	0.46	0.05	0.01	9.22E-07
SPINK1	rs7725017	5	147838260	A	C	0.60	-0.40	0.01	1.08E-273
SPINK1	rs777834943	16	20355409	T	C	0.18	-0.07	0.01	6.66E-06
SPINK4	rs11129965	3	42476887	A	G	0.55	-0.06	0.01	1.46E-09
SPINK4	rs369940	5	40726145	T	A	0.68	-0.07	0.01	1.07E-12
SPINK4	rs4879679	9	33234021	T	C	0.84	-1.11	0.01	0.00E+00
SPINK4	rs12804877	11	1087248	G	A	0.22	0.05	0.01	4.25E-06
SPINK4	-	11	111599854	T	C	0.71	0.06	0.01	5.82E-09
SPINK4	rs28529068	18	22093044	G	A	0.24	0.11	0.01	3.22E-26
SPINK4	rs2834350	21	34024827	T	C	0.38	-0.05	0.01	1.24E-06
SPINK5	rs80293268	1	8147519	C	G	0.05	-0.12	0.02	1.24E-05
SPINK5	rs1260326	2	27508073	C	T	0.61	0.06	0.01	4.75E-09
SPINK5	rs11384422	2	60272726	G	A	0.58	0.05	0.01	1.33E-05
SPINK5	rs6883868	5	148121320	T	C	0.62	0.41	0.01	5.08E-280
SPINK5	rs2479016	6	2245111	G	A	0.43	-0.05	0.01	1.54E-05
SPINK5	rs587600964	9	133296749	G	C	0.12	-0.10	0.01	7.86E-09
SPINK5	rs11220477	11	126405507	T	C	0.04	-0.13	0.02	8.62E-07
SPINK5	rs186021206	17	7166093	A	G	0.01	0.87	0.05	1.60E-33
SPINK5	rs681343	19	48703205	T	C	0.51	0.20	0.01	8.68E-78
SPINK6	rs12717962	5	148186561	G	A	0.55	-0.36	0.01	9.80E-237
SPINT1	-	1	38885713	A	T	0.46	-0.09	0.01	7.10E-17
SPINT1	rs4671378	2	60267177	C	T	0.56	0.06	0.01	1.46E-07
SPINT1	rs13080057	3	98783709	A	G	0.09	0.13	0.01	2.54E-10
SPINT1	rs538174489	6	32540424	T	C	0.31	-0.08	0.01	8.20E-11
SPINT1	-	9	133255468	T	G	0.06	0.16	0.02	7.97E-12
SPINT1	rs56278466	10	17833858	G	T	0.66	0.09	0.01	1.17E-13
SPINT1	rs73415720	12	120906712	C	T	0.47	-0.05	0.01	3.10E-06
SPINT1	rs17658212	15	40853721	T	C	0.06	-0.67	0.02	7.05E-164
SPINT1	rs186021206	17	7166093	A	G	0.01	0.84	0.05	2.80E-28
SPINT1	rs708686	19	5840608	T	C	0.27	0.10	0.01	6.81E-15
SPINT1	rs584768	19	48710027	A	G	0.53	0.05	0.01	9.11E-06
SPINT2	rs13412535	2	224010157	A	G	0.23	-0.07	0.01	1.22E-07
SPINT2	rs1354034	3	56815721	C	T	0.60	-0.10	0.01	2.02E-17
SPINT2	rs5030062	3	186736391	C	A	0.37	-0.07	0.01	1.35E-08
SPINT2	rs11734099	4	6889708	A	G	0.17	0.07	0.01	1.13E-05
SPINT2	rs10016018	4	101873464	T	A	0.40	-0.06	0.01	6.02E-07
SPINT2	rs66530140	4	186240057	C	T	0.51	-0.15	0.01	4.21E-40
SPINT2	rs10058074	5	132350453	A	G	0.48	-0.06	0.01	1.10E-06
SPINT2	rs2545801	5	177414338	C	T	0.74	-0.07	0.01	4.72E-08
SPINT2	rs35173808	6	31358371	A	C	0.10	-0.09	0.01	2.25E-06
SPINT2	rs6961069	7	80589645	T	C	0.40	0.07	0.01	1.13E-09
SPINT2	rs139141690	7	101856650	A	G	0.00	0.45	0.05	7.50E-08
SPINT2	rs6993770	8	105569300	T	A	0.29	-0.13	0.01	1.66E-25
SPINT2	-	9	133263362	G	A	0.18	-0.08	0.01	2.00E-08
SPINT2	rs10761737	10	63292445	C	T	0.41	0.09	0.01	1.79E-16
SPINT2	rs111763415	10	102570539	G	T	0.43	-0.05	0.01	3.72E-06
SPINT2	rs111338191	12	111388673	T	A	0.52	-0.06	0.01	3.16E-07
SPINT2	rs12445050	16	81837364	T	C	0.14	0.09	0.01	2.02E-08
SPINT2	rs45437199	19	38304610	A	G	0.18	-0.19	0.01	6.33E-41
SPINT2	rs892090	19	55027704	G	T	0.83	0.13	0.01	1.71E-18
SPOCK1	rs34546909	1	47498019	T	G	0.80	0.15	0.01	4.68E-27
SPOCK1	rs4655581	1	68254752	T	C	0.09	-0.09	0.01	2.67E-06
SPOCK1	rs77736461	2	18499499	T	C	0.31	-0.06	0.01	2.76E-06

SPOCK1	rs62155324	2	67541208	A	C	0.04	-0.19	0.02	1.10E-09
SPOCK1	rs10000888	4	145888016	G	A	0.51	0.05	0.01	2.11E-05
SPOCK1	rs4835737	5	137559375	G	A	0.37	-0.13	0.01	5.24E-31
SPOCK1	rs7756288	6	15110481	C	T	0.26	-0.10	0.01	8.89E-15
SPOCK1	rs3129777	6	32678407	A	C	0.21	-0.09	0.01	1.09E-09
SPOCK1	rs2505507	10	43149376	T	C	0.27	0.06	0.01	4.88E-06
SPOCK1	rs703987	10	79179462	C	G	0.61	-0.06	0.01	7.17E-07
SPOCK1	rs149440923	11	71997603	G	A	0.02	0.24	0.03	2.90E-07
SPOCK1	rs36100715	12	128861663	G	T	0.11	-0.12	0.01	5.79E-11
SPOCK1	rs3916033	17	46812337	T	C	0.55	0.05	0.01	1.88E-06
SPON1	rs1270942	6	31951083	G	A	0.13	-0.10	0.01	7.07E-10
SPON1	rs8176741	9	133256074	A	G	0.06	0.16	0.01	7.90E-14
SPON1	rs10832164	11	14026933	T	C	0.52	-0.29	0.01	8.60E-158
SPON1	rs3082163	11	57190822	T	A	0.19	0.08	0.01	2.62E-08
SPON1	rs77542162	17	69085137	G	A	0.02	0.26	0.02	9.65E-14
SPON1	rs68013007	19	32447775	T	A	0.16	-0.08	0.01	9.58E-08
SPON1	rs33950747	19	35848345	T	C	0.08	0.09	0.01	2.41E-05
SPON1	rs516316	19	48702888	C	G	0.51	-0.08	0.01	2.31E-13
SPON2	rs28855974	4	1121121	G	A	0.12	0.31	0.01	7.75E-73
SPP1	rs35085476	1	159580314	C	T	0.02	-0.17	0.02	5.57E-06
SPP1	rs559637118	1	196710779	T	G	0.40	-0.05	0.01	2.40E-06
SPP1	rs9853056	3	52521941	C	T	0.56	0.05	0.01	5.93E-06
SPP1	rs56254643	4	87937733	C	T	0.19	-0.11	0.01	2.55E-16
SPP1	rs4253328	4	186257633	T	C	0.51	-0.06	0.01	1.03E-07
SPP1	rs11995824	8	119000461	G	C	0.54	0.06	0.01	2.48E-08
SPP1	-	10	63156270	A	T	0.48	0.06	0.01	1.36E-07
SPP1	rs548589580	13	42399147	A	T	0.48	0.06	0.01	1.35E-07
SPP1	rs117068593	14	92651884	T	C	0.19	-0.09	0.01	1.77E-09
SPP1	rs113374757	19	46753285	T	C	0.17	0.07	0.01	9.65E-06
SPP1	rs738408	22	43928850	T	C	0.22	0.06	0.01	7.11E-06
SPRY2	rs3811444	1	247876149	T	C	0.33	-0.05	0.01	1.95E-05
SPRY2	rs1354034	3	56815721	C	T	0.60	0.10	0.01	9.25E-17
SPRY2	rs342298	7	106733200	T	C	0.45	-0.07	0.01	1.36E-08
SPRY2	rs6993770	8	105569300	T	A	0.29	-0.08	0.01	6.32E-10
SPRY2	rs11502185	11	180258	C	T	0.26	0.07	0.01	2.87E-06
SPRY2	rs6580981	12	54329244	A	G	0.46	0.08	0.01	3.66E-12
SPRY2	rs504122	13	80337390	A	G	0.37	-0.08	0.01	3.63E-12
SPRY2	rs200309755	17	29518756	T	C	0.54	0.05	0.01	8.86E-06
SRC	rs17622656	5	132485305	A	G	0.39	-0.05	0.01	4.98E-06
SRP14	rs1354034	3	56815721	C	T	0.60	0.11	0.01	1.32E-21
SRP14	rs597808	12	111535554	G	A	0.52	-0.05	0.01	2.60E-05
SRP14	-	17	35559053	T	A	0.17	0.10	0.01	1.75E-12
SRPK2	rs1354034	3	56815721	C	T	0.60	0.11	0.01	1.96E-20
SRPK2	rs342298	7	106733200	T	C	0.46	-0.05	0.01	5.95E-06
SRPK2	rs7080386	10	63288546	A	C	0.41	0.06	0.01	5.54E-08
SRPK2	rs11502185	11	180258	C	T	0.26	0.06	0.01	1.58E-05
SRPK2	rs60822569	12	54323724	C	T	0.55	0.05	0.01	1.12E-05
SSC4D	rs12140070	1	28009070	G	A	0.31	-0.15	0.01	2.99E-42
SSC4D	rs165316	1	91067740	G	A	0.20	-0.07	0.01	6.63E-07
SSC4D	rs6908943	6	32667519	T	C	0.18	-0.07	0.01	2.71E-06
SSC4D	rs12540573	7	76409696	C	A	0.06	0.68	0.01	2.77E-213
SSC4D	rs11183272	12	45988473	A	C	0.49	0.05	0.01	9.18E-07
SSC4D	rs112635299	14	94371805	T	G	0.02	0.18	0.02	1.41E-06
SSC4D	rs2498786	14	104796031	G	C	0.62	-0.10	0.01	2.01E-20
SSC4D	rs339969	15	60591082	A	C	0.62	0.06	0.01	2.81E-07
SSC4D	rs35523349	17	47631920	A	C	0.46	0.05	0.01	9.04E-06

SSC4D	rs4803852	19	45735668	T	G	0.82	0.06	0.01	6.82E-06
SSC4D	rs1800961	20	44413724	T	C	0.03	-0.16	0.02	1.84E-07
SSC4D	rs2739344	22	23956106	C	A	0.58	-0.06	0.01	2.10E-07
SSC5D	rs56210800	3	124753745	G	C	0.13	-0.09	0.01	7.85E-09
SSC5D	rs13107325	4	102267552	T	C	0.08	0.15	0.01	1.00E-13
SSC5D	rs521977	6	31869050	G	T	0.68	0.05	0.01	1.94E-05
SSC5D	-	7	7252014	C	A	0.62	0.10	0.01	1.05E-19
SSC5D	rs10231604	7	100148578	A	G	0.56	0.06	0.01	2.23E-07
SSC5D	rs2142306	8	133458388	C	T	0.41	0.09	0.01	6.24E-17
SSC5D	rs7358384	11	11619477	A	T	0.38	0.10	0.01	2.20E-22
SSC5D	rs964184	11	116778201	C	G	0.87	-0.07	0.01	2.37E-06
SSC5D	rs10778118	12	101430728	T	C	0.79	0.06	0.01	2.74E-06
SSC5D	rs748853879	15	56843630	A	T	0.04	0.11	0.02	2.29E-05
SSC5D	rs200489612	17	7203059	A	G	0.01	0.68	0.05	1.92E-18
SSC5D	rs55799523	19	55488178	A	C	0.27	0.53	0.01	0.00E+00
SSC5D	rs181210490	20	3715330	A	G	0.01	0.83	0.03	1.79E-63
ST3GAL1	rs12740374	1	109274968	T	G	0.22	-0.10	0.01	4.59E-15
ST3GAL1	rs539657009	1	161658666	T	C	0.13	0.08	0.01	1.96E-06
ST3GAL1	rs4672497	2	62296430	G	C	0.22	0.18	0.01	7.27E-42
ST3GAL1	rs7633553	3	58454509	C	T	0.70	0.05	0.01	2.12E-05
ST3GAL1	-	6	135099929	C	T	0.27	-0.06	0.01	1.84E-06
ST3GAL1	rs9643300	8	133490905	T	C	0.56	-0.24	0.01	3.95E-100
ST3GAL1	rs782134971	9	133264504	G	C	0.25	0.14	0.01	1.36E-27
ST3GAL1	rs56278466	10	17833858	G	T	0.66	0.05	0.01	1.72E-05
ST3GAL1	rs28929474	14	94378610	T	C	0.02	0.24	0.02	9.33E-10
ST3GAL1	rs28873836	19	51811402	C	G	0.27	-0.10	0.01	6.02E-17
ST6GAL1	rs150110347	1	24982271	T	C	0.01	0.25	0.03	1.65E-06
ST6GAL1	rs4686837	3	187021889	A	G	0.31	-0.18	0.01	3.13E-50
ST6GAL1	rs6993770	8	105569300	T	A	0.29	-0.06	0.01	5.75E-06
ST6GAL1	rs9414801	10	63389329	A	G	0.53	-0.05	0.01	7.46E-06
ST6GAL1	rs142744740	11	116818075	C	A	0.81	-0.07	0.01	2.02E-05
ST6GAL1	rs71454159	12	89521106	T	A	0.72	0.06	0.01	1.37E-05
ST6GAL1	rs2695293	12	101693237	A	C	0.58	0.05	0.01	4.08E-06
ST6GAL1	rs28929474	14	94378610	T	C	0.02	0.21	0.03	6.59E-08
ST6GAL1	rs61658003	19	55032835	A	C	0.84	0.07	0.01	3.08E-06
STAMBP	rs559935910	2	73889990	A	G	0.00	-0.89	0.11	1.47E-07
STAMBP	rs1354034	3	56815721	C	T	0.60	0.06	0.01	5.90E-07
STAMBP	rs10733789	10	63188924	C	T	0.31	0.06	0.01	7.92E-06
STAT5B	rs1354034	3	56815721	C	T	0.60	0.05	0.01	1.63E-05
STAT5B	rs342293	7	106731773	G	C	0.46	-0.06	0.01	2.09E-08
STAT5B	rs6993770	8	105569300	T	A	0.29	-0.07	0.01	4.11E-07
STC1	rs57247992	8	23875381	A	G	0.08	0.12	0.01	3.99E-08
STC1	rs2519093	9	133266456	T	C	0.18	-0.08	0.01	1.69E-08
STC1	rs11812859	10	833279	C	A	0.08	0.11	0.01	9.62E-08
STC1	rs11830324	12	510452	A	T	0.17	-0.08	0.01	2.52E-07
STC1	rs478141	13	71771960	A	G	0.39	-0.08	0.01	1.92E-11
STC1	rs12460997	19	7693907	A	C	0.49	-0.11	0.01	4.45E-25
STC2	rs2894602	2	226385086	G	A	0.77	0.06	0.01	1.83E-06
STC2	-	5	173336651	T	A	0.39	0.08	0.01	1.22E-11
STC2	rs5896958	9	21981631	T	C	0.72	-0.06	0.01	1.96E-05
STC2	rs12380110	9	136194972	C	T	0.16	0.07	0.01	8.92E-06
STC2	rs10830278	11	89483257	A	T	0.80	0.06	0.01	1.11E-05
STC2	rs11362821	12	7514128	A	G	0.98	-0.16	0.02	7.59E-06
STC2	rs3184504	12	111446804	C	T	0.52	-0.06	0.01	1.64E-07
STC2	-	16	58767006	C	T	0.09	0.09	0.01	6.92E-06
STC2	rs1126464	16	89637957	C	G	0.24	-0.07	0.01	6.73E-08

STC2	rs12986064	19	54251270	C	T	0.51	-0.06	0.01	1.88E-08
STIP1	rs1354034	3	56815721	C	T	0.60	0.06	0.01	3.61E-08
STIP1	rs10739067	9	4684770	G	A	0.34	0.08	0.01	1.56E-10
STK11	rs1354034	3	56815721	C	T	0.60	0.07	0.01	2.12E-10
STK11	rs342293	7	106731773	G	C	0.46	-0.05	0.01	1.63E-05
STK11	rs6993770	8	105569300	T	A	0.29	-0.07	0.01	2.90E-08
STK11	rs7924036	10	63431885	T	G	0.50	0.05	0.01	4.99E-06
STK24	rs1354034	3	56815721	C	T	0.60	0.05	0.01	1.08E-05
STK24	rs6993770	8	105569300	T	A	0.29	-0.06	0.01	1.11E-05
STK4	rs1354034	3	56815721	C	T	0.60	0.07	0.01	6.40E-10
STK4	rs10900809	5	132490630	A	G	0.39	-0.05	0.01	2.72E-05
STK4	rs6993770	8	105569300	T	A	0.29	-0.08	0.01	2.43E-09
STK4	rs1894572	20	45056869	C	T	0.47	0.05	0.01	6.87E-06
STX16	rs218476	20	58662614	A	G	0.38	0.06	0.01	1.69E-06
STX4	rs7090758	10	63575555	C	T	0.48	-0.05	0.01	2.00E-05
STX4	rs12292693	11	65169248	C	A	0.26	-0.07	0.01	1.47E-08
STX6	rs139299944	6	32634888	T	C	0.34	0.05	0.01	2.73E-05
STX6	rs342293	7	106731773	G	C	0.46	-0.05	0.01	1.77E-05
STX8	rs1354034	3	56815721	C	T	0.60	0.05	0.01	6.68E-06
STX8	rs6993770	8	105569300	T	A	0.29	-0.06	0.01	4.69E-06
STX8	rs8077500	17	9440454	A	G	0.24	0.10	0.01	3.19E-14
SUGT1	rs1354034	3	56815721	C	T	0.60	0.06	0.01	6.99E-08
SUGT1	rs60822569	12	54323724	C	T	0.55	0.05	0.01	1.73E-05
SULT1A1	rs148788997	16	30311847	C	G	0.13	0.15	0.01	2.17E-17
SULT2A1	rs4861708	4	186236079	A	G	0.51	0.07	0.01	4.60E-11
SULT2A1	rs6894249	5	132461855	G	A	0.39	-0.05	0.01	2.01E-05
SULT2A1	-	7	64510228	T	A	0.64	-0.06	0.01	5.62E-07
SULT2A1	rs62129966	19	47871693	A	C	0.17	-0.37	0.01	6.91E-136
SULT2A1	rs3747207	22	43928975	A	G	0.22	0.07	0.01	8.15E-08
SUMF2	rs2642442	1	220800221	T	C	0.68	-0.05	0.01	2.47E-05
SUMF2	rs1289395	1	226854761	C	T	0.46	-0.05	0.01	4.94E-06
SUMF2	rs9852409	3	57637814	A	T	0.82	-0.19	0.01	4.92E-41
SUMF2	rs114082534	4	118813029	A	G	0.06	0.11	0.02	2.39E-06
SUMF2	rs62396264	6	31352885	G	C	0.04	0.25	0.02	9.56E-19
SUMF2	rs6796	7	6462736	C	T	0.28	0.07	0.01	6.82E-09
SUMF2	rs35384521	7	56078132	A	G	0.02	-1.53	0.03	1.03E-307
SUMF2	rs556893622	7	61107238	A	G	0.01	-0.54	0.04	8.54E-15
SUMF2	rs745346394	7	67894425	G	A	0.00	-1.02	0.15	1.63E-05
SUMF2	rs79499703	7	127485694	T	C	0.07	-0.19	0.01	9.91E-17
SUMF2	rs199922514	8	9325592	G	A	0.91	-0.22	0.01	9.15E-33
SUMF2	rs11999525	9	136476890	A	G	0.26	-0.06	0.01	2.61E-06
SUMF2	rs506596	11	32075869	C	T	0.28	-0.06	0.01	2.41E-07
SUMF2	rs1169288	12	120978847	C	A	0.31	-0.05	0.01	1.44E-05
SUMF2	rs2291256	12	132816737	T	C	0.09	-0.10	0.01	3.71E-07
SUMF2	rs438811	19	44913484	T	C	0.24	0.06	0.01	8.53E-07
SUMF2	rs4806498	19	54171009	T	C	0.43	-0.07	0.01	7.46E-11
SUSD1	rs2278668	3	123116385	C	T	0.59	-0.05	0.01	2.79E-05
SUSD1	rs2631360	5	132371737	A	G	0.52	0.06	0.01	6.61E-07
SUSD1	rs146879704	9	112124289	T	C	0.00	-1.28	0.07	2.68E-35
SUSD1	rs61744929	11	2304197	C	T	0.02	-0.43	0.03	1.25E-24
SUSD1	rs200309755	17	29518756	T	C	0.54	0.05	0.01	1.02E-05
SUSD2	rs62292583	3	186942006	T	C	0.13	-0.08	0.01	7.75E-08
SUSD2	rs9269502	6	32575344	G	A	0.36	-0.06	0.01	1.32E-06
SUSD2	rs7952602	11	126363774	C	G	0.14	-0.07	0.01	1.99E-05
SUSD2	rs200489612	17	7203059	A	G	0.01	0.59	0.05	7.42E-15
SUSD2	rs117587385	22	24181236	A	G	0.04	1.19	0.02	0.00E+00

TACC3	rs745543985	4	1739806	C	T	0.47	0.21	0.01	1.65E-73
TACC3	rs6993770	8	105569300	T	A	0.29	-0.06	0.01	8.08E-07
TACC3	rs7080386	10	63288546	A	C	0.41	0.08	0.01	5.42E-13
TACC3	rs8057254	16	8959132	A	T	0.19	0.07	0.01	7.10E-07
TACC3	rs13381663	18	25280795	C	T	0.21	0.09	0.01	1.13E-10
TACSTD2	rs7333	1	58575548	T	C	0.16	-0.91	0.01	0.00E+00
TACSTD2	rs12740374	1	109274968	T	G	0.22	-0.15	0.01	1.13E-25
TACSTD2	rs780093	2	27519736	C	T	0.62	0.05	0.01	2.40E-05
TACSTD2	rs34070949	3	98635347	A	C	0.47	-0.05	0.01	1.69E-07
TACSTD2	rs76507355	6	29939996	C	T	0.08	0.10	0.01	1.82E-07
TACSTD2	rs7824174	8	97800530	C	T	0.48	0.25	0.01	1.37E-136
TACSTD2	rs8176693	9	133262254	T	C	0.06	0.27	0.01	1.63E-38
TACSTD2	rs529700	10	17843438	C	T	0.51	0.07	0.01	2.35E-08
TACSTD2	rs1077834	15	58431280	C	T	0.22	0.07	0.01	4.03E-08
TACSTD2	rs56156922	16	56953457	C	T	0.32	0.07	0.01	1.12E-10
TACSTD2	rs72802342	16	75200974	A	C	0.08	-0.10	0.01	4.03E-07
TACSTD2	rs186021206	17	7166093	A	G	0.01	0.66	0.04	1.07E-21
TACSTD2	rs72835428	17	49183288	T	C	0.12	0.11	0.01	1.27E-13
TACSTD2	rs681343	19	48703205	T	C	0.51	0.07	0.01	4.67E-11
TACSTD2	rs2868346	20	45919331	T	C	0.76	0.06	0.01	1.02E-06
TAFA5	rs2870238	4	76451926	T	C	0.50	0.06	0.01	2.30E-08
TAFA5	rs113878851	16	20353760	T	C	0.17	-0.09	0.01	2.75E-08
TAFA5	rs79072	22	48371374	T	G	0.71	0.18	0.01	1.48E-43
TARBP2	rs1354034	3	56815721	C	T	0.60	0.07	0.01	1.67E-10
TARBP2	rs342293	7	106731773	G	C	0.46	-0.05	0.01	3.67E-06
TARBP2	rs7080386	10	63288546	A	C	0.41	0.05	0.01	2.66E-05
TBC1D17	rs746738	19	49883083	C	T	0.43	0.41	0.01	2.63E-299
TBC1D23	rs1036770	3	100299871	T	C	0.59	-0.15	0.01	3.29E-40
TBC1D23	rs342293	7	106731773	G	C	0.46	-0.05	0.01	1.53E-05
TBC1D23	rs6993770	8	105569300	T	A	0.29	-0.06	0.01	8.23E-07
TBC1D23	rs7090758	10	63575555	C	T	0.48	-0.05	0.01	1.06E-05
TBC1D5	rs1354034	3	56815721	C	T	0.60	0.06	0.01	6.43E-07
TBC1D5	rs11227136	11	65164493	A	G	0.24	-0.07	0.01	3.37E-07
TBCB	rs1354034	3	56815721	C	T	0.60	0.06	0.01	6.56E-08
TBCB	rs342298	7	106733200	T	C	0.45	-0.05	0.01	5.21E-06
TBCB	rs6993770	8	105569300	T	A	0.29	-0.06	0.01	2.09E-06
TBCB	rs7896518	10	63344740	G	A	0.42	0.06	0.01	3.35E-07
TBCC	rs1354034	3	56815721	C	T	0.60	0.06	0.01	2.46E-07
TBCC	rs2234026	6	42745880	G	A	0.99	0.59	0.03	3.04E-32
TBCC	rs342298	7	106733200	T	C	0.45	-0.06	0.01	1.50E-06
TBCC	rs3847326	10	63572099	G	A	0.48	-0.05	0.01	4.96E-06
TBCC	rs11502185	11	180258	C	T	0.26	0.06	0.01	1.91E-05
TBL1X	rs1354034	3	56815721	C	T	0.60	-0.12	0.01	1.19E-25
TBL1X	rs736801	5	132497907	T	C	0.39	-0.05	0.01	1.23E-05
TBL1X	rs139141690	7	101856650	A	G	0.00	-0.44	0.06	3.23E-07
TBL1X	rs6993770	8	105569300	T	A	0.29	-0.07	0.01	1.26E-07
TBL1X	rs12767683	10	102556824	A	C	0.32	0.08	0.01	4.01E-11
TBL1X	rs60822569	12	54323724	C	T	0.55	0.07	0.01	2.91E-08
TCL1A	rs301816	1	8444998	A	G	0.59	-0.05	0.01	8.86E-06
TCL1A	rs17361097	4	152080067	A	G	0.44	0.05	0.01	7.35E-06
TCL1A	rs10689049	5	88824262	T	C	0.31	0.06	0.01	9.69E-08
TCL1A	rs77018303	5	143812499	T	G	0.02	-0.22	0.03	2.27E-07
TCL1A	rs17056273	5	158822119	C	T	0.08	-0.20	0.01	3.35E-23
TCL1A	rs34907473	6	31344161	G	A	0.28	-0.08	0.01	6.75E-10
TCL1A	rs652951	6	44596271	A	C	0.49	-0.05	0.01	3.12E-06
TCL1A	rs9483788	6	135114363	C	T	0.26	-0.07	0.01	8.41E-09

TCL1A	-	7	26867540	T	A	0.27	-0.05	0.01	2.18E-05
TCL1A	-	7	39959783	A	T	0.36	-0.05	0.01	6.21E-06
TCL1A	rs116968179	7	50149587	A	G	0.03	-0.14	0.02	7.24E-06
TCL1A	rs1433577	8	129583635	A	G	0.27	-0.08	0.01	1.67E-11
TCL1A	rs4948492	10	61959980	T	C	0.66	-0.07	0.01	2.26E-10
TCL1A	rs12784975	10	96620380	C	T	0.19	-0.08	0.01	3.30E-08
TCL1A	rs112170428	11	14435383	A	G	0.26	-0.05	0.01	2.78E-05
TCL1A	rs76428106	13	28029870	C	T	0.01	0.22	0.03	1.49E-05
TCL1A	rs78986913	14	95696081	A	G	0.04	-0.71	0.02	4.31E-149
TCL1A	rs11853271	15	90482598	A	G	0.32	0.06	0.01	1.44E-06
TCL1A	rs13330176	16	85985481	A	T	0.24	0.09	0.01	1.80E-11
TCL1A	rs12947321	17	2816578	G	A	0.49	-0.06	0.01	4.59E-08
TCL1A	rs35222145	17	39851068	G	T	0.49	-0.10	0.01	3.43E-20
TCL1A	rs6065926	20	46107215	G	A	0.75	-0.06	0.01	1.80E-05
TCL1A	rs75743846	22	41929013	A	G	0.02	-0.20	0.03	3.73E-06
TCN2	-	9	133263362	G	A	0.18	-0.06	0.01	1.30E-07
TCN2	rs150384171	19	8305034	A	C	0.01	1.23	0.03	6.79E-197
TCN2	rs12986064	19	54251270	C	T	0.51	-0.07	0.01	1.28E-12
TCN2	rs740234	22	30612758	G	A	0.20	0.63	0.01	0.00E+00
TDGF1	rs112481213	3	46577748	A	T	0.24	1.32	0.01	0.00E+00
TDGF1	rs113354603	9	114323961	A	G	0.07	-0.07	0.01	2.33E-05
TDGF1	rs507666	9	133273983	A	G	0.18	-0.05	0.01	6.43E-08
TDRKH	rs4845556	1	151772382	A	G	0.48	0.31	0.01	9.14E-165
TDRKH	rs1354034	3	56815721	C	T	0.60	0.06	0.01	2.78E-07
TDRKH	rs6993770	8	105569300	T	A	0.29	-0.07	0.01	8.15E-08
TDRKH	rs7080386	10	63288546	A	C	0.41	0.05	0.01	4.07E-06
TEK	rs74816838	1	161673770	T	C	0.17	0.07	0.01	2.34E-06
TEK	rs10935473	3	98698056	T	G	0.44	-0.07	0.01	5.03E-13
TEK	rs13107325	4	102267552	T	C	0.08	0.12	0.01	7.89E-10
TEK	rs1050518	6	31356864	A	T	0.30	-0.22	0.01	3.47E-89
TEK	rs2134964	8	88385408	G	A	0.70	-0.11	0.01	1.36E-22
TEK	rs682632	9	27183465	C	A	0.96	-1.04	0.02	4.79E-296
TEK	rs8176759	9	133254260	A	G	0.06	0.81	0.02	4.57E-266
TEK	rs174530	11	61779120	G	A	0.37	0.04	0.01	2.69E-05
TEK	rs35166255	11	126431861	A	G	0.03	0.28	0.02	3.77E-25
TEK	rs1042704	14	22843385	A	G	0.22	-0.10	0.01	9.45E-16
TEK	rs186021206	17	7166093	A	G	0.01	0.47	0.04	1.01E-11
TFF1	rs760077	1	155208991	T	A	0.60	-0.10	0.01	2.18E-18
TFF1	rs62133344	2	68950137	A	C	0.06	0.41	0.02	2.23E-69
TFF1	rs2075842	11	1193830	C	G	0.42	0.07	0.01	1.39E-11
TFF1	rs536255	21	39638874	T	C	0.19	-0.08	0.01	7.18E-09
TFF1	rs3761376	21	42366929	A	G	0.24	-0.31	0.01	5.25E-128
TFF2	rs760077	1	155208991	T	A	0.60	-0.10	0.01	1.38E-19
TFF2	rs11379990	3	12482550	T	A	0.50	-0.08	0.01	6.80E-15
TFF2	-	4	48014421	G	A	0.38	-0.06	0.01	6.52E-08
TFF2	rs2978981	8	142677719	T	C	0.44	-0.05	0.01	6.96E-06
TFF2	rs2075842	11	1193830	C	G	0.42	0.11	0.01	2.33E-25
TFF2	rs2217142	13	48860499	T	C	0.58	-0.07	0.01	4.19E-11
TFF2	rs186021206	17	7166093	A	G	0.01	0.71	0.05	7.53E-22
TFF2	rs601338	19	48703417	A	G	0.51	-0.05	0.01	8.75E-06
TFF2	rs1534081	21	39651684	G	A	0.75	0.07	0.01	4.35E-08
TFF2	rs225344	21	42355775	A	G	0.73	0.12	0.01	5.74E-24
TFF3	rs9992101	4	76439278	A	G	0.41	0.05	0.01	1.52E-05
TFF3	rs2524277	6	31439802	A	G	0.06	0.60	0.02	9.30E-146
TFF3	rs118095917	21	42313518	T	C	0.01	-1.25	0.04	5.15E-74
TFPI	rs12127364	1	169499904	A	C	0.27	0.14	0.01	3.64E-33

TFPI	rs1260326	2	27508073	C	T	0.61	-0.05	0.01	1.22E-05
TFPI	rs7576066	2	187479054	A	G	0.29	0.35	0.01	1.76E-184
TFPI	rs562281690	3	90128763	T	G	0.00	-0.49	0.07	9.31E-06
TFPI	rs528128538	3	93868695	T	C	0.00	-0.45	0.06	7.27E-06
TFPI	rs28601761	8	125487789	G	C	0.42	-0.06	0.01	3.47E-08
TFPI	rs1111796	9	114323946	T	A	0.08	-0.17	0.01	4.42E-15
TFPI	-	10	19943243	C	T	0.58	0.08	0.01	1.24E-14
TFPI	rs7412	19	44908822	T	C	0.08	-0.15	0.01	1.10E-14
TFPI2	rs61804208	1	161691621	T	G	0.10	0.14	0.01	7.76E-15
TFPI2	rs68066031	2	224015781	C	T	0.23	0.07	0.01	5.39E-08
TFPI2	rs1056523	3	126542364	T	C	0.25	-0.14	0.01	2.65E-30
TFPI2	rs5879368	6	116269018	A	T	0.33	-0.06	0.01	2.64E-06
TFPI2	rs6924387	6	136761810	G	A	0.41	-0.06	0.01	2.97E-07
TFPI2	rs62466701	7	93956351	C	T	0.06	0.24	0.01	1.29E-24
TFPI2	rs10087526	8	54520591	G	T	0.20	0.09	0.01	6.43E-12
TFPI2	rs35719208	8	104954086	T	C	0.19	0.09	0.01	8.58E-12
TFPI2	rs5791751	11	47013006	A	T	0.67	-0.08	0.01	6.36E-13
TFPI2	rs10842452	12	25050552	A	C	0.88	-0.14	0.01	8.73E-17
TFPI2	rs2160725	17	68399585	C	A	0.48	-0.06	0.01	1.80E-07
TFRC	rs766000481	1	205275030	A	C	0.36	-0.05	0.01	5.93E-06
TFRC	rs3817672	3	196073940	T	C	0.56	-0.52	0.01	0.00E+00
TFRC	rs252152	5	142066209	G	A	0.66	0.08	0.01	1.68E-12
TFRC	rs1800562	6	26092913	A	G	0.08	-0.27	0.01	1.24E-44
TFRC	rs6592965	7	50360284	A	G	0.45	-0.06	0.01	1.29E-08
TFRC	rs3211931	7	80668857	T	C	0.43	-0.05	0.01	9.37E-06
TFRC	rs2075672	7	100642673	G	A	0.62	-0.07	0.01	3.53E-10
TFRC	rs550057	9	133271182	T	C	0.26	-0.10	0.01	1.95E-16
TFRC	rs560046267	10	45501143	T	G	0.31	0.05	0.01	4.33E-06
TFRC	rs11216316	11	117210784	C	A	0.10	0.23	0.01	1.43E-38
TFRC	rs74035509	16	88500925	T	C	0.08	0.09	0.01	2.07E-05
TFRC	rs59435462	17	45673555	A	T	0.76	-0.06	0.01	5.73E-07
TFRC	rs2072860	22	37074564	A	G	0.53	-0.14	0.01	2.16E-38
TGFA	rs17639251	2	70549149	C	T	0.12	0.17	0.01	1.28E-22
TGFA	rs7310615	12	111427245	G	C	0.52	-0.07	0.01	8.17E-11
TGFA	rs11871747	17	40021029	C	T	0.39	0.08	0.01	4.00E-13
TGFA	rs62143194	19	53816370	G	C	0.21	0.06	0.01	2.25E-05
TGFB1	rs3804749	3	123114156	T	C	0.59	-0.07	0.01	5.21E-10
TGFB1	rs4869313	5	96888176	G	T	0.55	-0.09	0.01	2.57E-14
TGFB1	rs6796	7	6462736	C	T	0.28	0.06	0.01	1.98E-06
TGFB1	rs2544221	7	134308262	C	G	0.46	0.06	0.01	2.86E-08
TGFB1	rs6993770	8	105569300	T	A	0.29	-0.14	0.01	4.02E-30
TGFB1	-	9	133276163	T	A	0.19	-0.11	0.01	9.31E-15
TGFB1	rs7080386	10	63288546	A	C	0.41	0.05	0.01	1.65E-05
TGFB1	rs12762934	10	102600127	T	C	0.32	0.06	0.01	3.27E-07
TGFB1	rs7136223	12	29284928	G	A	0.28	0.08	0.01	1.03E-09
TGFB1	rs73045269	19	41319286	T	C	0.17	0.29	0.01	1.97E-83
TGFB1	rs892090	19	55027704	G	T	0.83	0.12	0.01	3.53E-14
TGFBI	rs2478539	1	230709026	T	G	0.40	-0.25	0.01	1.01E-130
TGFBI	rs11127048	2	27529596	A	G	0.62	-0.06	0.01	5.85E-09
TGFBI	rs13159365	5	136053744	T	C	0.51	-0.54	0.01	0.00E+00
TGFBI	rs9268812	6	32456791	G	A	0.66	0.07	0.01	1.37E-10
TGFBI	rs10645152	7	77233094	T	G	0.74	-0.11	0.01	9.70E-23
TGFBI	rs28601761	8	125487789	G	C	0.42	-0.06	0.01	6.95E-09
TGFBI	rs10982156	9	114325784	A	T	0.07	-0.44	0.01	4.06E-99
TGFBI	rs56278466	10	17833858	G	T	0.66	0.06	0.01	7.39E-08
TGFBI	rs28929474	14	94378610	T	C	0.02	0.20	0.02	4.88E-08

TGFBI	rs116683462	17	59774420	T	A	0.54	0.08	0.01	9.31E-14
TGFBI	rs12975366	19	54255498	C	T	0.39	-0.06	0.01	9.49E-10
TGFBR2	rs61747728	1	179557079	T	C	0.04	0.19	0.02	1.32E-11
TGFBR2	rs1260326	2	27508073	C	T	0.61	0.05	0.01	1.73E-06
TGFBR2	rs75166367	2	162107791	A	G	0.06	-0.11	0.01	5.98E-06
TGFBR2	rs143048713	3	46851683	T	G	0.10	0.08	0.01	2.58E-05
TGFBR2	rs3129887	6	32442914	A	G	0.19	0.09	0.01	5.35E-10
TGFBR2	rs10265221	7	151717243	C	T	0.29	0.06	0.01	6.45E-06
TGFBR2	rs507666	9	133273983	A	G	0.18	-0.17	0.01	8.99E-34
TGFBR2	rs777834943	16	20355409	T	C	0.18	-0.08	0.01	1.30E-08
TGFBR2	rs2837988	21	41247617	A	C	0.36	0.05	0.01	1.25E-05
TGFBR3	rs11207423	1	59185157	C	G	0.28	0.10	0.01	9.48E-15
TGFBR3	rs2634023	1	91858593	T	C	0.08	-0.23	0.01	4.34E-29
TGFBR3	rs150816167	1	179602727	C	T	0.04	0.14	0.02	6.03E-07
TGFBR3	rs4253311	4	186253529	G	A	0.51	-0.08	0.01	2.10E-11
TGFBR3	rs181242111	10	17823665	A	G	0.12	0.10	0.01	3.21E-07
TGFBR3	rs4997081	16	20353912	C	G	0.19	-0.07	0.01	4.45E-07
TGFBR3	rs1236213	21	27105423	T	G	0.59	-0.16	0.01	4.01E-43
TGM2	rs539726870	6	32638754	G	A	0.12	-0.13	0.01	3.67E-12
TGM2	rs6592965	7	50360284	A	G	0.45	0.06	0.01	1.19E-07
TGM2	rs757944190	17	28833612	C	A	0.26	0.06	0.01	1.46E-06
TGM2	rs2235582	20	38165738	G	A	0.63	0.17	0.01	1.44E-50
THBD	rs7532110	1	47496292	A	T	0.80	0.07	0.01	1.67E-06
THBD	rs61747728	1	179557079	T	C	0.04	0.18	0.02	7.09E-10
THBD	rs1260326	2	27508073	C	T	0.61	0.05	0.01	6.06E-06
THBD	rs75166367	2	162107791	A	G	0.06	-0.11	0.01	2.35E-06
THBD	rs56387622	3	46846708	C	T	0.10	0.08	0.01	2.61E-05
THBD	rs638333	5	73123440	C	T	0.29	0.07	0.01	7.04E-09
THBD	rs9264666	6	31271519	C	G	0.55	-0.06	0.01	2.89E-08
THBD	rs1137827	9	133254328	T	C	0.06	0.33	0.01	8.61E-48
THBD	rs34434834	11	126437901	A	G	0.03	0.25	0.02	4.52E-16
THBD	rs12442366	15	101233358	A	G	0.38	0.05	0.01	4.47E-06
THBD	rs77924615	16	20381010	A	G	0.20	-0.08	0.01	1.33E-08
THBD	rs186021206	17	7166093	A	G	0.01	0.40	0.05	9.24E-08
THBD	rs2659005	17	81244914	T	C	0.44	0.07	0.01	2.58E-09
THBD	rs33950747	19	35848345	T	C	0.08	0.15	0.01	1.90E-12
THBD	rs1042579	20	23048087	A	G	0.19	0.20	0.01	1.85E-45
THBS2	rs2271708	5	40936439	C	T	0.01	-0.34	0.05	1.57E-06
THBS2	rs78109196	6	169224606	A	C	0.10	0.66	0.01	5.45E-265
THBS2	rs3967200	11	126362490	T	C	0.14	-0.12	0.01	2.10E-13
THBS2	rs531745895	14	68777300	T	G	0.34	-0.05	0.01	4.83E-06
THBS2	rs186021206	17	7166093	A	G	0.01	0.57	0.05	2.15E-14
THBS2	rs146385050	17	62559897	A	C	0.18	0.23	0.01	5.36E-63
THBS2	rs2111504	19	32426549	A	T	0.17	0.14	0.01	2.99E-23
THBS2	rs738409	22	43928847	G	C	0.22	0.07	0.01	5.99E-08
THBS4	rs2438632	5	80096370	T	G	0.40	0.24	0.01	1.66E-99
THBS4	rs2881756	7	7216809	T	C	0.41	0.07	0.01	5.47E-11
THBS4	rs8176719	9	133257521	C	T	0.34	0.05	0.01	1.49E-05
THBS4	rs56278466	10	17833858	G	T	0.66	0.09	0.01	1.00E-15
THBS4	rs112771035	11	126355981	G	C	0.07	-0.13	0.01	4.67E-10
THBS4	rs186021206	17	7166093	A	G	0.01	1.34	0.05	1.85E-70
THOP1	rs2741990	19	2796646	T	C	0.29	0.26	0.01	8.98E-107
THPO	rs2305637	3	47004356	T	C	0.18	-0.08	0.01	1.88E-07
THPO	rs113926195	3	58237678	C	G	0.23	-0.06	0.01	4.06E-06
THPO	rs6141	3	184372478	T	C	0.53	0.06	0.01	8.27E-08
THPO	rs11242109	5	132341354	T	G	0.48	-0.06	0.01	3.02E-07

THPO	rs10224886	7	38202565	A	T	0.25	-0.12	0.01	1.04E-20
THPO	rs6993770	8	105569300	T	A	0.29	-0.13	0.01	4.01E-24
THPO	rs10740118	10	63341447	C	G	0.41	0.08	0.01	6.09E-11
THPO	rs10762489	10	71891700	G	A	0.22	0.07	0.01	2.04E-06
THPO	rs56385468	11	1773446	G	T	0.09	-0.21	0.01	2.10E-25
THPO	rs1362213	12	6173262	T	C	0.48	-0.07	0.01	1.50E-08
THPO	rs2015599	12	29282547	A	G	0.46	-0.05	0.01	4.87E-06
THPO	rs6580981	12	54329244	A	G	0.46	0.06	0.01	2.16E-06
THPO	-	12	101891200	A	G	0.50	0.05	0.01	2.65E-05
THPO	rs28929474	14	94378610	T	C	0.02	0.35	0.03	7.94E-19
THPO	rs151234	16	28494339	C	G	0.13	0.08	0.01	4.23E-06
THPO	rs12445050	16	81837364	T	C	0.14	0.08	0.01	5.25E-06
THPO	rs149394327	17	66232877	C	G	0.03	0.16	0.02	4.55E-06
THPO	rs1654425	19	55027612	C	T	0.83	0.10	0.01	4.00E-11
THY1	rs603424	10	100315722	A	G	0.17	-0.08	0.01	1.08E-07
THY1	rs55933700	11	119423203	T	G	0.02	-1.08	0.02	3.06E-181
THY1	rs186021206	17	7166093	A	G	0.01	0.35	0.05	3.54E-06
TIA1	rs1354034	3	56815721	C	T	0.60	0.09	0.01	3.92E-13
TIA1	rs17622656	5	132485305	A	G	0.39	-0.05	0.01	2.59E-05
TIA1	-	10	63156270	A	T	0.49	0.05	0.01	2.52E-05
TIA1	rs11502185	11	180258	C	T	0.26	0.07	0.01	1.12E-06
TIA1	rs60822569	12	54323724	C	T	0.55	0.06	0.01	9.94E-07
TIE1	rs4660729	1	43302229	G	C	0.61	0.19	0.01	4.89E-68
TIE1	rs10935473	3	98698056	T	G	0.44	-0.22	0.01	1.04E-92
TIE1	rs2962006	5	58152249	C	G	0.39	-0.05	0.01	3.80E-06
TIE1	rs9271325	6	32614736	G	C	0.47	-0.06	0.01	3.39E-07
TIE1	rs4841133	8	9326154	G	A	0.91	-0.09	0.01	8.41E-07
TIE1	rs8176746	9	133255935	T	G	0.06	0.89	0.02	0.00E+00
TIE1	rs56278466	10	17833858	G	T	0.66	0.07	0.01	7.66E-09
TIE1	rs174564	11	61820833	G	A	0.35	0.06	0.01	2.48E-07
TIE1	rs34434834	11	126437901	A	G	0.03	0.16	0.02	2.06E-07
TIE1	rs2283464	16	100766	A	G	0.85	-0.14	0.01	2.72E-20
TIE1	rs186021206	17	7166093	A	G	0.01	0.52	0.05	1.86E-12
TIE1	rs4015	19	48750132	T	C	0.20	-0.07	0.01	1.16E-06
TIGAR	rs3217860	12	4289884	G	A	0.25	-0.07	0.01	2.10E-06
TIGAR	rs4632248	19	53821741	T	G	0.21	0.07	0.01	4.78E-07
TIMD4	rs1060622	1	93154836	A	G	0.62	0.07	0.01	1.22E-09
TIMD4	rs12145753	1	234960192	T	C	0.35	0.05	0.01	3.93E-06
TIMD4	rs10189685	2	202623726	A	G	0.30	0.12	0.01	1.51E-24
TIMD4	rs67833823	2	231361235	A	G	0.16	0.07	0.01	6.43E-06
TIMD4	rs11395592	3	69793190	T	A	0.43	-0.05	0.01	1.78E-05
TIMD4	rs371216559	3	155820016	T	C	0.27	-0.07	0.01	7.91E-08
TIMD4	rs115216147	5	71369699	A	T	0.07	0.11	0.01	1.36E-06
TIMD4	rs38029	5	97017519	G	C	0.56	-0.07	0.01	3.10E-09
TIMD4	rs4704826	5	156965071	A	C	0.64	-0.23	0.01	1.13E-93
TIMD4	rs9270270	6	32589431	C	T	0.84	-0.15	0.01	9.52E-23
TIMD4	rs10456852	6	105908171	T	C	0.13	0.10	0.01	1.57E-10
TIMD4	rs2737245	8	115646356	T	G	0.28	0.06	0.01	4.78E-06
TIMD4	rs2142306	8	133458388	C	T	0.41	0.06	0.01	9.67E-09
TIMD4	rs9411378	9	133270015	A	C	0.22	0.10	0.01	2.48E-14
TIMD4	rs2068888	10	93079885	A	G	0.45	0.06	0.01	3.28E-08
TIMD4	rs72823014	10	114026477	A	G	0.13	0.08	0.01	1.28E-06
TIMD4	-	11	44570719	T	A	0.78	0.07	0.01	7.50E-08
TIMD4	rs10769256	11	47356845	T	C	0.40	-0.06	0.01	1.34E-08
TIMD4	rs112771035	11	126355981	G	C	0.07	0.49	0.01	3.86E-120
TIMD4	rs34623398	12	29336225	T	C	0.28	0.08	0.01	6.10E-11

TIMD4	rs10778118	12	101430728	T	C	0.79	-0.07	0.01	1.42E-07
TIMD4	-	16	56967362	A	C	0.34	0.05	0.01	6.78E-06
TIMD4	rs34417180	16	72185857	A	C	0.17	-0.07	0.01	4.46E-06
TIMD4	rs4843861	16	85917370	T	A	0.27	-0.07	0.01	9.78E-09
TIMD4	rs186021206	17	7166093	A	G	0.01	1.14	0.05	1.07E-54
TIMD4	rs77542162	17	69085137	G	A	0.02	0.16	0.02	1.99E-05
TIMD4	rs601338	19	48703417	A	G	0.51	0.05	0.01	1.13E-05
TIMD4	-	20	35545566	T	A	0.14	0.11	0.01	1.10E-12
TIMD4	rs2143876	20	40490434	C	T	0.14	0.09	0.01	3.08E-08
TIMD4	rs66817580	21	41317710	T	G	0.15	0.16	0.01	1.29E-25
TIMP1	rs1775815	1	117606208	T	G	0.57	0.07	0.01	1.79E-08
TIMP1	rs12407843	1	156903935	A	G	0.11	0.09	0.01	3.43E-07
TIMP1	rs9380143	6	29834268	T	C	0.28	-0.10	0.01	7.04E-15
TIMP1	rs6993770	8	105569300	T	A	0.29	-0.11	0.01	8.67E-20
TIMP1	rs1654425	19	55027612	C	T	0.83	0.11	0.01	1.09E-13
TIMP3	rs2274319	1	156481081	C	T	0.65	-0.06	0.01	1.47E-08
TIMP3	rs13412535	2	224010157	A	G	0.23	-0.06	0.01	2.60E-06
TIMP3	rs1354034	3	56815721	C	T	0.60	-0.05	0.01	7.38E-06
TIMP3	rs11734099	4	6889708	A	G	0.17	0.08	0.01	4.56E-09
TIMP3	rs755492124	4	101856385	T	G	0.39	-0.05	0.01	3.73E-06
TIMP3	rs114694170	5	88884379	C	T	0.06	0.16	0.01	1.08E-13
TIMP3	-	5	132328823	T	A	0.63	-0.05	0.01	1.48E-05
TIMP3	rs11953411	5	151418773	A	G	0.61	-0.06	0.01	2.05E-08
TIMP3	rs12190479	6	132512815	C	G	0.20	-0.06	0.01	1.19E-05
TIMP3	rs6961069	7	80589645	T	C	0.40	0.05	0.01	5.33E-06
TIMP3	rs61469632	9	132986603	C	T	0.06	-0.13	0.01	2.48E-09
TIMP3	rs7896518	10	63344740	G	A	0.42	0.08	0.01	1.18E-14
TIMP3	rs17655730	11	270715	C	T	0.25	0.06	0.01	6.40E-06
TIMP3	rs1362213	12	6173262	T	C	0.48	-0.05	0.01	1.04E-05
TIMP3	rs12445050	16	81837364	T	C	0.14	0.09	0.01	6.19E-09
TIMP3	rs74035509	16	88500925	T	C	0.08	0.08	0.01	1.92E-05
TIMP3	rs892090	19	55027704	G	T	0.83	0.14	0.01	5.73E-23
TIMP3	rs6081569	20	19323142	C	G	0.37	0.05	0.01	7.96E-06
TIMP3	rs5749504	22	32766845	T	C	0.73	-0.65	0.01	0.00E+00
TIMP4	rs2943649	2	226240975	G	C	0.65	-0.06	0.01	1.82E-07
TIMP4	rs184262	3	12093240	G	A	0.83	0.45	0.01	6.31E-215
TIMP4	rs34579624	6	32662342	A	G	0.15	0.09	0.01	9.15E-08
TIMP4	rs1358980	6	43796814	T	C	0.48	-0.06	0.01	2.80E-07
TIMP4	rs1716403	12	124035299	C	T	0.68	-0.05	0.01	4.46E-06
TIMP4	rs12986064	19	54251270	C	T	0.51	-0.05	0.01	1.49E-05
TINAGL1	rs78265569	1	2214726	A	C	0.09	-0.09	0.01	2.47E-05
TINAGL1	rs2229474	1	21855622	A	G	0.01	0.61	0.03	1.36E-38
TINAGL1	rs945214	1	31581704	G	A	0.61	-0.09	0.01	4.14E-13
TINAGL1	rs7648959	3	159844708	G	A	0.30	0.09	0.01	2.61E-13
TINAGL1	rs1694889	3	192954103	C	G	0.51	0.05	0.01	1.65E-05
TINAGL1	rs56278466	10	17833858	G	T	0.66	0.06	0.01	2.46E-07
TINAGL1	rs7137828	12	111494996	T	C	0.52	-0.05	0.01	1.45E-06
TINAGL1	rs9583474	13	110243053	C	A	0.21	-0.08	0.01	5.25E-08
TINAGL1	rs6143024	20	62339173	G	A	0.24	-0.06	0.01	1.45E-06
TJAP1	rs1354034	3	56815721	C	T	0.60	0.06	0.01	3.48E-08
TJAP1	rs549884121	6	43547784	A	C	0.01	-0.32	0.05	1.32E-05
TLR3	rs61804206	1	161689031	G	A	0.11	0.04	0.01	1.12E-05
TLR3	rs3775291	4	186082920	T	C	0.30	-1.15	0.01	0.00E+00
TLR3	rs635634	9	133279427	T	C	0.18	-0.04	0.00	4.60E-08
TLR3	rs56278466	10	17833858	G	T	0.66	0.04	0.00	1.48E-12
TLR3	rs4760	19	43648948	G	A	0.16	0.08	0.01	1.48E-26

TLR3	rs738409	22	43928847	G	C	0.22	0.06	0.00	1.15E-15
TMPRSS15	rs11134475	5	156972939	G	A	0.64	0.08	0.01	1.20E-10
TMPRSS15	rs59922816	8	41520778	C	T	0.11	0.10	0.01	1.37E-07
TMPRSS15	rs550057	9	133271182	T	C	0.26	-0.21	0.01	3.35E-57
TMPRSS15	rs708686	19	5840608	T	C	0.27	0.11	0.01	4.29E-18
TMPRSS15	rs438811	19	44913484	T	C	0.24	0.06	0.01	2.33E-05
TMPRSS15	rs492602	19	48703160	G	A	0.51	-0.18	0.01	7.21E-58
TMPRSS15	rs2824805	21	18398417	C	G	0.17	0.13	0.01	6.79E-18
TMPRSS5	rs35383942	1	201468704	T	C	0.06	0.13	0.01	3.08E-11
TMPRSS5	rs12024555	1	221974503	A	G	0.19	0.05	0.01	8.73E-06
TMPRSS5	rs199927946	2	111993137	T	G	0.62	0.04	0.01	1.95E-05
TMPRSS5	rs1371687	3	98886146	G	A	0.34	-0.05	0.01	2.28E-07
TMPRSS5	rs67670470	3	152612950	A	C	0.34	0.05	0.01	1.10E-06
TMPRSS5	rs4686658	3	194005178	T	C	0.78	-0.05	0.01	2.30E-06
TMPRSS5	rs60028714	4	85472147	T	C	0.62	-0.06	0.01	1.05E-09
TMPRSS5	rs9386298	6	96039693	T	A	0.50	0.05	0.01	1.32E-06
TMPRSS5	-	7	43781353	C	T	0.21	0.05	0.01	1.37E-05
TMPRSS5	rs17326497	9	33111688	A	G	0.07	-0.14	0.01	1.20E-14
TMPRSS5	rs56278466	10	17833858	G	T	0.66	0.07	0.01	1.40E-11
TMPRSS5	rs10887793	10	88231148	T	G	0.45	0.05	0.01	7.10E-08
TMPRSS5	rs7114195	11	113690699	C	A	0.64	0.78	0.01	0.00E+00
TMPRSS5	rs68055275	11	126369248	T	C	0.14	-0.09	0.01	5.69E-08
TMPRSS5	rs28404455	13	50241222	C	T	0.09	0.08	0.01	5.10E-07
TMPRSS5	rs72681869	14	50188639	C	G	0.01	0.20	0.03	6.94E-06
TMPRSS5	rs3742780	14	75038305	C	G	0.48	-0.05	0.01	2.22E-08
TMPRSS5	rs12912843	15	50707136	G	T	0.12	0.08	0.01	1.53E-08
TMPRSS5	rs186021206	17	7166093	A	G	0.01	0.56	0.04	1.37E-18
TMPRSS5	rs504549	19	36946299	T	C	0.38	-0.05	0.01	8.98E-07
TMPRSS5	rs2836683	21	38825514	C	T	0.34	0.04	0.01	2.41E-05
TMSB10	rs1354034	3	56815721	C	T	0.60	0.06	0.01	1.84E-06
TMSB10	rs342293	7	106731773	G	C	0.46	-0.06	0.01	1.29E-06
TMSB10	rs6993770	8	105569300	T	A	0.29	-0.06	0.01	7.90E-06
TMSB10	rs4746204	10	63568758	T	C	0.48	-0.05	0.01	2.93E-06
TMSB10	rs11502185	11	180258	C	T	0.26	0.07	0.01	1.02E-06
TMSB10	rs10774624	12	111395984	A	G	0.51	-0.06	0.01	6.13E-07
TMSB10	rs4632248	19	53821741	T	G	0.21	0.13	0.01	4.99E-20
TNC	rs4655585	1	65689944	C	T	0.37	-0.05	0.01	5.67E-06
TNC	rs2646260	2	237369152	G	A	0.24	-0.06	0.01	7.61E-06
TNC	rs4686846	3	187054623	T	C	0.23	0.07	0.01	1.81E-07
TNC	rs13135092	4	102276925	G	A	0.08	0.08	0.01	2.11E-05
TNC	rs13159365	5	136053744	T	C	0.51	0.20	0.01	1.13E-75
TNC	rs113760175	6	22343363	A	G	0.07	0.10	0.01	4.14E-06
TNC	rs1990790	7	130393789	T	C	0.51	-0.05	0.01	2.04E-06
TNC	rs2685412	8	27953086	G	C	0.27	0.38	0.01	5.25E-204
TNC	rs4263799	8	71482756	T	C	0.48	0.08	0.01	2.68E-12
TNC	rs112286501	8	99015365	T	C	0.04	0.14	0.02	1.39E-07
TNC	rs7021057	9	115135714	C	T	0.29	-0.24	0.01	4.13E-94
TNC	rs7949566	11	126415406	A	G	0.42	0.08	0.01	1.27E-12
TNC	rs9668810	12	26273487	C	T	0.74	-0.08	0.01	3.92E-10
TNC	rs10411932	19	3470266	C	T	0.48	-0.05	0.01	2.10E-05
TNC	rs760715	22	39466338	C	T	0.25	0.11	0.01	5.37E-16
TNF	rs4645843	6	31576785	T	C	0.00	-1.42	0.11	8.52E-17
TNF	rs7161799	15	58478324	T	C	0.08	0.23	0.01	3.36E-27
TNF	rs3184504	12	111446804	C	T	0.52	-0.11	0.01	6.44E-21
TNFAIP8	rs1035376	5	119312555	A	G	0.88	0.12	0.01	3.45E-11
TNFRSF10A	rs79287178	3	172576710	A	G	0.03	0.25	0.02	2.69E-16

TNFRSF10A	rs1632984	6	29805615	A	T	0.85	0.06	0.01	8.53E-06
TNFRSF10A	rs13278062	8	23225458	T	G	0.50	-0.46	0.01	0.00E+00
TNFRSF10A	rs3072826	17	68456500	G	A	0.36	-0.05	0.01	3.48E-06
TNFRSF10B	rs231996	3	172555945	A	C	0.76	-0.06	0.01	2.38E-07
TNFRSF10B	rs1105944	8	23027596	G	A	0.90	0.54	0.01	2.27E-212
TNFRSF10B	rs3184504	12	111446804	C	T	0.52	-0.05	0.01	1.66E-06
TNFRSF10C	rs3917932	1	36478315	G	C	0.58	-0.04	0.01	1.26E-06
TNFRSF10C	rs3014874	1	153365467	A	G	0.26	-0.04	0.01	2.29E-05
TNFRSF10C	rs3762281	1	156334991	G	A	0.50	0.08	0.01	1.11E-18
TNFRSF10C	rs5030738	1	161629864	T	G	0.02	0.20	0.02	8.17E-09
TNFRSF10C	rs6723921	2	62304597	G	A	0.61	0.05	0.01	1.01E-06
TNFRSF10C	rs10182318	2	196928580	C	T	0.90	-0.09	0.01	5.89E-09
TNFRSF10C	rs12497115	3	47178851	T	C	0.18	0.05	0.01	2.15E-05
TNFRSF10C	rs3774315	3	172514196	G	A	0.27	0.08	0.01	5.41E-14
TNFRSF10C	rs4690299	4	747685	T	C	0.50	0.04	0.01	2.07E-05
TNFRSF10C	rs2249742	6	31272944	T	C	0.44	0.05	0.01	1.40E-09
TNFRSF10C	rs149110519	6	144064640	T	C	0.04	0.13	0.02	8.76E-08
TNFRSF10C	rs1010366	7	7190195	C	T	0.32	0.06	0.01	2.15E-11
TNFRSF10C	rs58609917	7	35626657	A	C	0.38	0.04	0.01	7.23E-06
TNFRSF10C	rs445	7	92779056	T	C	0.10	-0.09	0.01	1.71E-08
TNFRSF10C	-	8	23114758	G	T	0.01	-1.76	0.03	2.74E-256
TNFRSF10C	rs7846314	8	60738272	T	A	0.19	0.06	0.01	1.89E-07
TNFRSF10C	rs3124753	9	133384145	A	G	0.47	-0.07	0.01	3.70E-14
TNFRSF10C	rs2241244	17	39997301	C	G	0.54	-0.09	0.01	4.73E-25
TNFRSF10C	rs4760	19	43648948	G	A	0.16	-1.17	0.01	0.00E+00
TNFRSF11A	rs550677346	1	26552488	T	A	0.20	0.06	0.01	3.64E-06
TNFRSF11A	rs9269215	6	32482496	G	T	0.27	0.05	0.01	1.80E-05
TNFRSF11A	rs342293	7	106731773	G	C	0.46	0.06	0.01	2.59E-08
TNFRSF11A	rs141129381	9	97929618	G	T	0.36	0.05	0.01	5.91E-06
TNFRSF11A	-	9	133276163	T	A	0.19	-0.10	0.01	2.75E-15
TNFRSF11A	rs7080386	10	63288546	A	C	0.41	0.05	0.01	2.37E-07
TNFRSF11A	rs12946745	17	76487027	C	G	0.67	-0.05	0.01	7.86E-06
TNFRSF11A	rs113339733	18	62351096	A	G	0.19	-0.59	0.01	0.00E+00
TNFRSF11A	rs516316	19	48702888	C	G	0.51	-0.12	0.01	2.79E-32
TNFRSF11B	rs114165349	1	26695422	C	G	0.02	-0.23	0.02	2.42E-11
TNFRSF11B	rs79287178	3	172576710	A	G	0.03	0.20	0.02	1.22E-10
TNFRSF11B	rs748443516	8	119088959	C	T	0.50	0.20	0.01	5.60E-84
TNFRSF11B	rs1013495	10	79380497	T	C	0.45	0.05	0.01	2.69E-07
TNFRSF11B	rs7927191	11	16231436	C	T	0.27	-0.06	0.01	3.79E-07
TNFRSF11B	rs3825568	14	68793871	T	C	0.46	-0.05	0.01	5.41E-06
TNFRSF11B	rs200489612	17	7203059	A	G	0.01	0.36	0.05	4.45E-06
TNFRSF11B	rs704	17	28367840	A	G	0.47	-0.20	0.01	1.50E-84
TNFRSF11B	rs77542162	17	69085137	G	A	0.02	0.15	0.02	1.58E-05
TNFRSF11B	rs3747207	22	43928975	A	G	0.22	0.08	0.01	1.54E-09
TNFRSF12A	rs139130389	11	72139110	A	C	0.07	-0.28	0.01	5.18E-43
TNFRSF12A	rs964184	11	116778201	C	G	0.87	-0.08	0.01	1.74E-06
TNFRSF12A	-	16	2982315	T	A	0.29	0.07	0.01	9.06E-09
TNFRSF12A	rs77542162	17	69085137	G	A	0.02	0.22	0.02	6.50E-10
TNFRSF13B	rs41268860	6	31254884	C	A	0.13	0.08	0.01	5.62E-06
TNFRSF13B	rs10806425	6	90216893	A	C	0.41	-0.05	0.01	3.27E-06
TNFRSF13B	rs3804329	6	106238552	G	A	0.20	0.09	0.01	9.88E-10
TNFRSF13B	rs113745074	7	151245263	C	T	0.12	0.09	0.01	8.96E-07
TNFRSF13B	rs1483572	8	78723008	C	A	0.69	-0.05	0.01	2.23E-05
TNFRSF13B	rs75587749	12	7462344	T	C	0.09	0.09	0.01	4.18E-06
TNFRSF13B	rs111338191	12	111388673	T	A	0.52	-0.07	0.01	2.35E-10
TNFRSF13B	rs374039502	13	108308037	A	T	0.02	0.70	0.03	4.08E-62

TNFRSF13B	rs12588969	14	102764421	C	G	0.68	-0.06	0.01	2.49E-06
TNFRSF13B	rs34562254	17	16939677	A	G	0.10	-0.17	0.01	6.66E-20
TNFRSF13B	-	17	46220093	A	G	0.18	-0.07	0.01	1.43E-05
TNFRSF13B	rs12986064	19	54251270	C	T	0.51	-0.06	0.01	1.24E-06
TNFRSF13B	rs6053024	20	4995159	T	G	0.39	0.05	0.01	2.69E-06
TNFRSF13B	rs73165110	22	41895852	A	G	0.08	-0.19	0.01	1.95E-19
TNFRSF13C	rs17056298	5	158841671	G	C	0.08	-0.13	0.01	1.56E-10
TNFRSF13C	rs9264918	6	31305041	G	T	0.20	-0.07	0.01	1.27E-07
TNFRSF13C	rs2395904	8	129605573	G	T	0.27	-0.05	0.01	2.70E-05
TNFRSF13C	rs7087507	10	61985930	G	A	0.35	0.06	0.01	5.38E-07
TNFRSF13C	rs12784975	10	96620380	C	T	0.19	-0.08	0.01	5.95E-08
TNFRSF13C	rs774868556	12	89431449	A	A	0.31	-0.12	0.01	3.13E-20
TNFRSF13C	rs374039502	13	108308037	A	T	0.02	0.21	0.03	4.97E-07
TNFRSF13C	rs13330176	16	85985481	A	T	0.24	0.06	0.01	1.44E-05
TNFRSF13C	rs34557412	17	16948873	G	A	0.01	0.69	0.04	1.21E-25
TNFRSF13C	rs35222145	17	39851068	G	T	0.49	-0.07	0.01	2.87E-10
TNFRSF13C	rs17676949	18	63118405	A	G	0.10	-0.08	0.01	1.15E-05
TNFRSF13C	rs1967330	19	17548874	T	C	0.57	0.05	0.01	6.42E-06
TNFRSF13C	rs625372	20	3704082	C	T	0.63	0.05	0.01	2.13E-05
TNFRSF13C	rs763882049	22	41931492	A	T	0.09	-0.79	0.01	0.00E+00
TNFRSF14	rs2495366	1	2557491	A	G	0.49	0.11	0.01	1.18E-24
TNFRSF14	rs61747728	1	179557079	T	C	0.04	0.14	0.02	5.11E-06
TNFRSF14	rs11926707	3	46884049	C	T	0.62	-0.05	0.01	1.85E-05
TNFRSF14	rs342293	7	106731773	G	C	0.46	-0.05	0.01	1.62E-05
TNFRSF14	rs6993770	8	105569300	T	A	0.29	-0.06	0.01	2.05E-06
TNFRSF14	rs111338191	12	111388673	T	A	0.52	-0.06	0.01	7.70E-07
TNFRSF19	rs6605317	3	198029337	A	T	0.23	0.06	0.01	7.77E-07
TNFRSF19	rs13146355	4	76490987	A	G	0.46	0.05	0.01	4.16E-06
TNFRSF19	rs61947047	13	23579770	T	C	0.31	0.25	0.01	5.17E-106
TNFRSF19	rs12434499	14	105886314	T	C	0.16	0.09	0.01	1.43E-08
TNFRSF19	rs35830321	16	20341705	A	C	0.17	-0.08	0.01	3.17E-08
TNFRSF19	rs2252576	21	41243366	T	C	0.24	0.14	0.01	3.06E-31
TNFRSF1A	rs61747728	1	179557079	T	C	0.04	0.17	0.02	2.10E-09
TNFRSF1A	rs13019008	2	9553607	A	G	0.51	0.07	0.01	1.29E-10
TNFRSF1A	rs553340154	6	32558001	G	T	0.33	0.07	0.01	9.94E-07
TNFRSF1A	rs10982156	9	114325784	A	T	0.07	0.18	0.01	6.68E-15
TNFRSF1A	rs9411365	9	133242881	G	C	0.07	0.12	0.01	1.01E-07
TNFRSF1A	rs4149584	12	6333477	T	C	0.02	-0.31	0.03	7.67E-13
TNFRSF1A	-	16	20343260	T	A	0.18	-0.08	0.01	1.30E-07
TNFRSF1A	rs186021206	17	7166093	A	G	0.01	0.33	0.05	2.33E-05
TNFRSF1A	rs516316	19	48702888	C	G	0.51	-0.06	0.01	9.52E-07
TNFRSF1B	rs5746026	1	12193005	A	G	0.04	-0.39	0.02	1.36E-43
TNFRSF1B	rs550677346	1	26552488	T	A	0.20	0.06	0.01	9.12E-06
TNFRSF1B	rs61747728	1	179557079	T	C	0.04	0.19	0.02	7.40E-11
TNFRSF1B	rs3093974	6	31538433	A	G	0.36	0.09	0.01	9.34E-14
TNFRSF1B	rs3184504	12	111446804	C	T	0.52	-0.08	0.01	3.92E-12
TNFRSF1B	rs77924615	16	20381010	A	G	0.20	-0.07	0.01	4.46E-06
TNFRSF1B	rs3814995	19	35851310	T	C	0.32	0.06	0.01	1.56E-06
TNFRSF21	rs583104	1	109278685	T	G	0.77	0.07	0.01	1.13E-07
TNFRSF21	rs150816167	1	179602727	C	T	0.04	0.19	0.02	2.30E-11
TNFRSF21	rs1260326	2	27508073	C	T	0.61	0.06	0.01	1.73E-06
TNFRSF21	rs75166367	2	162107791	A	G	0.06	-0.11	0.02	7.58E-06
TNFRSF21	rs13107325	4	102267552	T	C	0.08	0.12	0.01	5.41E-08
TNFRSF21	rs17613643	6	32663641	G	A	0.22	0.08	0.01	1.76E-07
TNFRSF21	rs148598211	6	47286508	T	C	0.00	-1.86	0.06	4.12E-106
TNFRSF21	rs2298475	11	126408308	C	T	0.08	-0.18	0.01	4.10E-18

TNFRSF21	rs77924615	16	20381010	A	G	0.20	-0.07	0.01	1.92E-07
TNFRSF21	rs186021206	17	7166093	A	G	0.01	1.14	0.05	4.74E-52
TNFRSF21	rs10445407	17	81288009	A	C	0.48	-0.05	0.01	7.38E-06
TNFRSF21	rs2008174	22	39464125	T	C	0.74	-0.06	0.01	2.10E-05
TNFRSF4	-	1	1216707	C	T	0.04	-0.31	0.02	3.82E-28
TNFRSF4	rs13081778	3	46912231	G	A	0.37	-0.05	0.01	2.26E-05
TNFRSF4	rs2524137	6	31296805	T	C	0.69	-0.13	0.01	9.23E-26
TNFRSF4	rs4946811	6	107099312	C	A	0.36	0.06	0.01	1.41E-06
TNFRSF4	rs7137828	12	111494996	T	C	0.52	-0.12	0.01	8.80E-30
TNFRSF4	-	12	118073854	A	G	0.03	-0.15	0.02	3.38E-06
TNFRSF4	rs72778736	16	57267123	T	C	0.26	0.06	0.01	1.01E-05
TNFRSF4	rs186021206	17	7166093	A	G	0.01	0.34	0.05	1.53E-05
TNFRSF6B	rs139025277	6	32679082	G	A	0.02	0.20	0.03	2.14E-06
TNFRSF6B	-	9	133263362	G	A	0.18	-0.07	0.01	9.44E-07
TNFRSF6B	rs7137828	12	111494996	T	C	0.52	-0.07	0.01	2.32E-10
TNFRSF6B	rs77542162	17	69085137	G	A	0.02	0.20	0.02	2.84E-08
TNFRSF6B	rs6062497	20	63704906	T	C	0.67	-0.31	0.01	1.26E-153
TNFRSF8	rs35249183	1	12039288	G	A	0.10	0.35	0.01	1.08E-74
TNFRSF8	rs3094005	6	31497270	T	G	0.14	0.14	0.01	4.11E-17
TNFRSF8	rs10817685	9	114927025	T	C	0.43	-0.11	0.01	1.94E-23
TNFRSF8	rs3184504	12	111446804	C	T	0.52	-0.08	0.01	6.34E-13
TNFRSF8	rs76428106	13	28029870	C	T	0.01	0.22	0.03	2.37E-05
TNFRSF8	rs2695167	15	41736622	G	A	0.02	0.37	0.03	1.04E-17
TNFRSF8	rs186021206	17	7166093	A	G	0.01	0.38	0.05	9.74E-07
TNFRSF8	rs3814995	19	35851310	T	C	0.31	0.05	0.01	1.57E-05
TNFRSF9	rs2493214	1	7934360	G	A	0.19	-0.19	0.01	6.25E-44
TNFRSF9	rs3117574	6	31757453	A	G	0.13	0.13	0.01	1.37E-14
TNFRSF9	rs111338191	12	111388673	T	A	0.52	-0.08	0.01	1.15E-12
TNFRSF9	rs374039502	13	108308037	A	T	0.02	0.20	0.03	1.88E-06
TNFRSF9	rs34557412	17	16948873	G	A	0.01	0.51	0.04	2.96E-14
TNFRSF9	rs61750000	19	6534717	C	G	0.00	-0.39	0.05	1.62E-06
TNFRSF9	rs73165110	22	41895852	A	G	0.08	-0.09	0.01	1.29E-05
TNFSF10	rs7519758	1	196856157	T	C	0.19	0.13	0.01	1.72E-22
TNFSF10	rs79287178	3	172576710	A	G	0.03	-0.54	0.02	6.05E-61
TNFSF10	rs756671612	3	186735641	G	T	0.11	-0.14	0.01	1.52E-16
TNFSF10	rs10502	6	89329951	C	G	0.38	0.05	0.01	5.15E-06
TNFSF10	-	8	23114758	G	T	0.01	-0.35	0.04	7.00E-09
TNFSF10	rs174551	11	61806212	C	T	0.34	0.06	0.01	1.14E-08
TNFSF10	rs1027315	12	57640409	C	T	0.43	-0.06	0.01	6.41E-08
TNFSF10	rs3751198	12	103753429	G	A	0.60	0.05	0.01	2.50E-05
TNFSF10	rs28929474	14	94378610	T	C	0.02	0.85	0.02	9.61E-114
TNFSF10	rs4788460	16	72120610	T	C	0.29	0.09	0.01	5.30E-15
TNFSF10	rs241771	17	28265920	C	T	0.55	0.05	0.01	5.99E-06
TNFSF10	rs8178824	17	66228657	T	C	0.03	0.51	0.02	4.93E-60
TNFSF10	rs680321	18	32217995	C	T	0.46	-0.11	0.01	2.94E-24
TNFSF10	rs4760	19	43648948	G	A	0.16	-0.48	0.01	7.60E-227
TNFSF11	rs552693039	1	109274241	C	T	0.18	0.06	0.01	2.19E-05
TNFSF11	rs79287178	3	172576710	A	G	0.03	-0.66	0.02	3.19E-84
TNFSF11	rs6988146	8	23228573	A	T	0.75	0.18	0.01	1.35E-42
TNFSF11	rs2737212	8	115608987	T	C	0.55	-0.05	0.01	3.61E-06
TNFSF11	rs1156545	8	119068453	A	G	0.46	-0.22	0.01	6.68E-85
TNFSF11	rs7137828	12	111494996	T	C	0.52	-0.06	0.01	2.51E-07
TNFSF11	rs2062305	13	42478744	A	G	0.53	-0.11	0.01	2.12E-23
TNFSF11	rs7146217	14	68784198	C	T	0.53	0.05	0.01	6.83E-06
TNFSF11	rs2207132	20	40513876	A	G	0.03	-0.15	0.02	1.25E-06
TNFSF12	rs2228099	1	150836413	G	C	0.37	-0.06	0.01	1.07E-07

TNFSF12	rs1354034	3	56815721	C	T	0.60	-0.05	0.01	6.54E-06
TNFSF12	rs828596	3	98746469	G	C	0.41	-0.07	0.01	2.08E-09
TNFSF12	rs9842051	3	143303014	C	G	0.73	0.07	0.01	1.86E-08
TNFSF12	rs13135092	4	102276925	G	A	0.08	0.14	0.01	7.14E-12
TNFSF12	rs4098923	6	31225979	G	A	0.45	-0.05	0.01	3.44E-06
TNFSF12	rs139141690	7	101856650	A	G	0.00	0.36	0.05	1.66E-05
TNFSF12	rs6993770	8	105569300	T	A	0.29	-0.07	0.01	8.21E-09
TNFSF12	rs11784985	8	143960331	T	A	0.41	0.07	0.01	6.51E-11
TNFSF12	rs60988380	9	33117956	T	C	0.10	-0.10	0.01	4.88E-08
TNFSF12	-	9	133263362	G	A	0.18	-0.13	0.01	1.99E-19
TNFSF12	rs10761741	10	63306426	T	G	0.41	0.10	0.01	5.67E-18
TNFSF12	rs745680717	12	480706	C	G	0.40	-0.07	0.01	6.05E-09
TNFSF12	rs11158588	14	65333158	A	G	0.79	-0.07	0.01	1.91E-07
TNFSF12	rs11075045	16	8940859	G	A	0.18	0.06	0.01	2.41E-05
TNFSF12	rs151233	16	28495107	T	C	0.13	0.07	0.01	1.18E-05
TNFSF12	rs80067372	17	7549435	A	G	0.29	0.37	0.01	1.72E-198
TNFSF12	rs77542162	17	69085137	G	A	0.02	0.17	0.02	6.22E-06
TNFSF12	rs892090	19	55027704	G	T	0.83	0.10	0.01	1.72E-12
TNFSF13	rs12740374	1	109274968	T	G	0.22	-0.24	0.01	6.36E-78
TNFSF13	rs1354034	3	56815721	C	T	0.60	-0.05	0.01	6.66E-06
TNFSF13	-	6	31391651	A	G	0.07	-0.11	0.01	2.62E-07
TNFSF13	rs11570136	16	11964975	A	T	0.33	0.06	0.01	3.89E-08
TNFSF13	rs3803800	17	7559652	G	A	0.79	-0.32	0.01	1.90E-132
TNFSF13	rs16961828	17	16916910	C	G	0.10	-0.10	0.01	1.62E-07
TNFSF13	rs77542162	17	69085137	G	A	0.02	0.17	0.02	4.51E-06
TNFSF13	rs892090	19	55027704	G	T	0.83	0.08	0.01	4.01E-08
TNFSF13B	rs35675148	3	31614721	T	C	0.77	-0.08	0.01	6.41E-09
TNFSF13B	rs3129755	6	32615280	G	A	0.57	0.10	0.01	2.31E-19
TNFSF13B	rs6488394	12	7512550	C	T	0.91	-0.15	0.01	2.35E-15
TNFSF13B	-	13	108308032	T	G	0.02	0.76	0.03	3.34E-77
TNFSF13B	rs79874727	14	105904579	A	G	0.11	0.09	0.01	5.53E-06
TNFSF13B	rs34557412	17	16948873	G	A	0.01	0.61	0.04	4.96E-21
TNFSF13B	rs12986064	19	54251270	C	T	0.51	-0.12	0.01	3.69E-28
TNFSF13B	rs763882049	22	41931492	A	T	0.09	0.39	0.01	2.77E-97
TNFSF13B	rs74510325	22	49921734	G	C	0.03	0.20	0.02	8.74E-12
TNFSF14	rs11242109	5	132341354	T	G	0.48	-0.06	0.01	7.05E-08
TNFSF14	rs6993770	8	105569300	T	A	0.29	-0.05	0.01	1.78E-05
TNFSF14	rs12411442	10	102589774	G	A	0.43	-0.05	0.01	7.12E-06
TNFSF14	rs344560	19	6665009	C	T	0.95	0.55	0.02	2.81E-108
TNFSF14	rs892090	19	55027704	G	T	0.83	0.11	0.01	3.95E-12
TNR	rs2235256	1	175599911	G	A	0.44	-0.37	0.01	3.16E-254
TNR	rs2169305	3	159791591	C	T	0.36	0.08	0.01	3.82E-12
TNR	rs2507997	6	31347004	G	A	0.31	-0.07	0.01	6.52E-09
TNR	rs1111796	9	114323946	G	A	0.60	0.14	0.01	5.71E-37
TNR	rs11594905	10	75899975	A	G	0.14	0.08	0.01	3.06E-07
TNR	rs1727	10	89307012	A	C	0.72	-0.06	0.01	4.60E-06
TNR	rs7120175	11	30468906	C	G	0.45	-0.05	0.01	6.97E-07
TNR	rs11603123	11	126435600	A	G	0.03	0.34	0.02	5.42E-31
TNR	rs4981022	12	103756096	A	G	0.68	0.08	0.01	1.08E-11
TNR	rs3184504	12	111446804	C	T	0.52	0.06	0.01	2.42E-09
TNR	rs4767280	12	114926148	T	C	0.40	-0.11	0.01	1.77E-25
TNR	rs12879626	14	34251928	G	T	0.61	0.13	0.01	1.48E-33
TNR	rs10658607	15	57564948	T	A	0.32	-0.06	0.01	7.97E-07
TNR	rs2439430	15	66625506	T	G	0.50	0.07	0.01	6.45E-10
TNR	rs4886410	15	74773303	C	G	0.68	0.06	0.01	5.15E-07
TNR	rs3817428	15	88872016	G	C	0.27	0.15	0.01	3.50E-37

TNR	rs186021206	17	7166093	A	G	0.01	0.60	0.05	2.25E-16
TNXB	rs147650812	5	136063181	C	T	0.01	-0.28	0.04	6.14E-06
TNXB	rs9267797	6	32062923	T	C	0.01	1.66	0.03	3.03E-276
TNXB	rs181242111	10	17823665	A	G	0.12	0.09	0.01	3.11E-07
TNXB	rs78689694	11	126364925	C	G	0.13	-0.15	0.01	1.93E-23
TNXB	rs186021206	17	7166093	A	G	0.01	0.81	0.05	2.58E-30
TNXB	rs1236213	21	27105423	T	G	0.59	-0.05	0.01	1.35E-06
TP53	rs1641549	17	7671457	T	C	0.26	0.07	0.01	1.18E-06
TP53INP1	rs58534292	14	21737747	C	T	0.65	-0.05	0.01	2.12E-05
TP53INP1	rs56366203	14	106008121	G	C	0.50	-0.06	0.01	1.07E-06
TP53INP1	rs8192297	15	89801121	C	T	0.11	-0.15	0.01	8.19E-17
TPMT	rs115306927	6	18126707	T	A	0.05	-1.09	0.02	0.00E+00
TPMT	rs6580981	12	54329244	A	G	0.46	0.05	0.01	1.29E-05
TPP1	rs6848819	4	153530526	C	A	0.03	0.15	0.02	1.88E-06
TPP1	rs10455861	6	159980630	A	G	0.14	-0.10	0.01	3.60E-09
TPP1	rs17154155	7	80604927	T	G	0.41	0.06	0.01	9.94E-07
TPP1	rs6993770	8	105569300	T	A	0.29	-0.11	0.01	8.11E-20
TPP1	rs9414801	10	63389329	A	G	0.53	-0.08	0.01	7.55E-12
TPP1	rs140726254	11	6617713	A	G	0.00	2.90	0.08	1.68E-130
TPP1	rs149871778	12	8946399	C	G	0.10	0.18	0.01	2.80E-23
TPP1	rs10778152	12	101831973	G	A	0.29	-0.13	0.01	7.36E-25
TPP1	rs3184504	12	111446804	C	T	0.52	-0.06	0.01	3.19E-08
TPP1	rs145078947	14	93186629	T	G	0.00	0.87	0.07	7.46E-16
TPP1	rs1045693	16	5025225	A	G	0.53	-0.08	0.01	2.26E-12
TPP1	rs8064959	17	5374434	T	A	0.31	-0.05	0.01	1.83E-05
TPP1	-	17	35559053	T	A	0.17	0.06	0.01	2.14E-05
TPP1	rs200210321	19	19283081	G	A	0.07	0.12	0.01	5.69E-08
TPP1	rs1654425	19	55027612	C	T	0.83	0.09	0.01	6.64E-10
TPPP3	rs13334364	16	67298462	C	T	0.08	-0.12	0.01	2.13E-07
TPSAB1	rs115427247	1	92412850	T	C	0.03	-0.15	0.01	1.75E-11
TPSAB1	rs16856110	1	205662639	G	A	0.23	-0.06	0.01	1.15E-09
TPSAB1	rs6672987	1	234559442	T	C	0.49	-0.04	0.01	1.43E-06
TPSAB1	rs56043070	1	247556467	A	G	0.07	0.09	0.01	2.50E-10
TPSAB1	rs62252239	3	69844820	T	G	0.21	0.05	0.01	2.90E-06
TPSAB1	-	3	114862405	G	T	0.54	-0.05	0.01	1.31E-09
TPSAB1	rs28455789	4	159042382	A	G	0.17	-0.05	0.01	3.38E-06
TPSAB1	rs876606	5	78505260	A	G	0.26	0.05	0.01	4.85E-07
TPSAB1	rs73112996	7	50320300	T	C	0.14	0.05	0.01	1.61E-05
TPSAB1	rs56278466	10	17833858	G	T	0.66	0.04	0.01	1.42E-06
TPSAB1	rs7127546	11	61922259	A	G	0.37	0.05	0.01	6.72E-11
TPSAB1	rs8051930	16	1245817	A	T	0.48	0.83	0.01	0.00E+00
TPSAB1	rs17758695	18	63253621	T	C	0.03	-0.12	0.01	1.34E-07
TPSAB1	rs78744187	19	33263642	T	C	0.08	0.19	0.01	2.53E-41
TPSAB1	rs111402121	19	43777672	T	C	0.38	0.07	0.01	1.75E-18
TPSAB1	-	19	51797309	C	T	0.22	0.06	0.01	1.29E-11
TRAF2	rs1354034	3	56815721	C	T	0.60	0.07	0.01	3.00E-09
TRAF2	rs419788	6	31961022	C	T	0.67	0.05	0.01	1.32E-05
TRAF2	-	9	136927852	T	C	0.60	-0.05	0.01	3.53E-06
TRAF2	rs4239702	20	46120612	C	T	0.72	-0.06	0.01	2.00E-06
TREM2	rs10919543	1	161538827	G	A	0.32	0.05	0.01	5.21E-06
TREM2	rs1410996	1	196727803	A	G	0.40	-0.05	0.01	2.73E-07
TREM2	rs531950386	6	32518763	T	G	0.48	-0.07	0.01	9.77E-09
TREM2	rs143332484	6	41161469	T	C	0.01	-1.47	0.03	1.71E-186
TREM2	rs913914	9	99495968	G	C	0.37	-0.05	0.01	1.39E-06
TREM2	rs7232	11	60173126	A	T	0.37	0.40	0.01	5.24E-283
TREM2	rs11057840	12	124831509	C	A	0.14	0.10	0.01	1.83E-11

TREM2	rs738409	22	43928847	G	C	0.22	0.06	0.01	4.34E-07
TREML2	rs150816167	1	179602727	C	T	0.04	0.14	0.02	1.69E-06
TREML2	rs11759347	6	41199359	A	C	0.24	0.30	0.01	5.25E-118
TREML2	rs56293029	6	135097901	A	C	0.26	0.08	0.01	2.83E-10
TREML2	rs409801	9	4744743	C	T	0.51	0.06	0.01	2.71E-07
TREML2	rs7080386	10	63288546	A	C	0.41	0.06	0.01	3.57E-08
TREML2	rs12576289	11	126395946	G	A	0.22	-0.07	0.01	1.80E-06
TREML2	rs111338191	12	111388673	T	A	0.52	-0.12	0.01	2.09E-27
TREML2	rs186021206	17	7166093	A	G	0.01	0.46	0.05	1.74E-09
TREML2	rs2659005	17	81244914	T	C	0.44	0.05	0.01	1.20E-05
TRIAP1	rs148212596	1	193105381	G	A	0.02	-0.43	0.03	3.52E-28
TRIAP1	rs9856296	3	14113953	C	T	0.30	-0.08	0.01	3.35E-10
TRIAP1	rs1354034	3	56815721	C	T	0.60	0.09	0.01	3.07E-14
TRIAP1	rs62508038	8	42398746	G	C	0.00	-1.04	0.10	2.00E-12
TRIAP1	rs7896518	10	63344740	G	A	0.42	0.06	0.01	3.04E-06
TRIAP1	-	11	263868	C	A	0.24	0.06	0.01	2.77E-05
TRIAP1	rs60822569	12	54323724	C	T	0.55	0.08	0.01	5.04E-12
TRIAP1	rs10418046	19	53824615	G	T	0.21	0.09	0.01	2.33E-11
TRIM21	rs915652	6	31781365	A	G	0.13	-0.09	0.01	9.27E-08
TRIM21	rs4746204	10	63568758	T	C	0.48	-0.05	0.01	1.39E-05
TRIM21	rs199881122	11	4385377	T	A	0.00	-1.12	0.08	5.71E-22
TRIM5	rs11821656	11	5667914	C	G	0.14	-0.41	0.01	4.83E-145
TSHB	rs6426808	1	19508921	A	G	0.51	0.09	0.01	1.22E-14
TSHB	rs334713	1	61156615	C	A	0.96	0.13	0.02	4.04E-06
TSHB	rs12068854	1	107867043	C	A	0.09	0.09	0.01	6.36E-06
TSHB	rs2712172	2	216756010	A	G	0.26	-0.08	0.01	5.09E-09
TSHB	rs10028213	4	148731458	G	C	0.20	-0.11	0.01	1.18E-14
TSHB	rs2046045	5	77239986	G	T	0.40	0.15	0.01	1.34E-37
TSHB	rs556025	6	32603103	T	C	0.35	0.06	0.01	3.55E-06
TSHB	rs9472136	6	43842284	T	C	0.40	-0.07	0.01	2.38E-10
TSHB	rs2983511	6	165630471	C	G	0.31	-0.12	0.01	1.74E-22
TSHB	-	8	32509087	A	T	0.18	-0.07	0.01	3.37E-06
TSHB	rs7032019	9	97785862	A	G	0.67	0.05	0.01	1.75E-05
TSHB	rs7128207	11	45208510	T	G	0.56	0.07	0.01	1.58E-10
TSHB	rs61938844	12	96190080	A	G	0.03	0.16	0.02	1.11E-05
TSHB	rs116909374	14	36269155	T	C	0.04	-0.18	0.02	9.21E-10
TSHB	rs724170	14	80991596	A	G	0.49	-0.05	0.01	1.97E-05
TSHB	rs7142772	14	104734040	G	T	0.44	-0.05	0.01	1.74E-05
TSHB	rs200066768	15	49436680	T	A	0.18	-0.08	0.01	1.99E-07
TSHB	rs200293726	16	79720543	T	A	0.31	-0.08	0.01	5.41E-11
TSHB	-	19	7213358	A	C	0.20	0.07	0.01	1.60E-05
TSHB	rs1203944	20	22616241	C	T	0.79	0.06	0.01	1.70E-05
TSPAN1	rs34463133	1	46185497	A	G	0.00	-0.46	0.06	9.11E-07
TSPAN1	rs2268535	3	187039295	A	G	0.24	0.06	0.01	1.17E-06
TSPAN1	rs708686	19	5840608	T	C	0.27	0.06	0.01	6.49E-07
TST	rs4821544	22	36862461	C	T	0.30	-0.11	0.01	5.83E-17
TXLNA	rs1354034	3	56815721	C	T	0.60	0.10	0.01	9.84E-20
TXLNA	rs342293	7	106731773	G	C	0.46	-0.06	0.01	3.07E-07
TXLNA	rs6993770	8	105569300	T	A	0.29	-0.05	0.01	1.98E-05
TXLNA	rs11502185	11	180258	C	T	0.26	0.07	0.01	5.61E-06
TXNDC15	rs188468174	1	24965206	T	C	0.01	-0.51	0.03	2.81E-36
TXNDC15	rs115276619	1	184895998	A	T	0.02	0.21	0.03	1.08E-07
TXNDC15	rs6054	4	154568456	T	C	0.01	0.31	0.04	2.93E-06
TXNDC15	rs6876611	5	96907894	T	A	0.48	-0.05	0.01	4.16E-06
TXNDC15	rs3733897	5	134887903	G	A	0.13	1.17	0.01	0.00E+00
TXNDC15	rs2524096	6	31268690	T	G	0.42	-0.17	0.01	4.59E-69

TXNDC15	rs73015965	6	160706469	G	A	0.01	0.34	0.04	3.23E-07
TXNDC15	-	11	61824523	G	A	0.31	0.06	0.01	3.59E-08
TXNDC15	rs139130389	11	72139110	A	C	0.07	-0.19	0.01	6.61E-25
TXNDC15	rs28929474	14	94378610	T	C	0.02	0.23	0.02	1.31E-11
TXNDC15	rs12929396	16	11680769	T	C	0.49	-0.04	0.01	6.91E-06
TXNDC15	rs9302635	16	72110275	C	T	0.18	-0.09	0.01	4.24E-13
TXNDC15	rs1801689	17	66214462	C	A	0.03	0.50	0.02	2.76E-73
TXNDC5	rs1354034	3	56815721	C	T	0.60	0.07	0.01	8.65E-10
TXNDC5	rs11962800	6	7886672	G	A	0.11	-0.16	0.01	9.07E-18
TXNDC5	rs6993770	8	105569300	T	A	0.29	-0.07	0.01	2.27E-07
TXNDC5	rs6580981	12	54329244	A	G	0.46	0.06	0.01	5.23E-07
TXNRD1	rs1354034	3	56815721	C	T	0.60	0.06	0.01	1.78E-06
TXNRD1	rs6993770	8	105569300	T	A	0.29	-0.07	0.01	1.13E-07
TXNRD1	rs10743940	12	7498542	T	A	0.97	-0.17	0.02	1.78E-06
TXNRD1	rs60822569	12	54323724	C	T	0.55	0.05	0.01	6.85E-06
TXNRD1	rs201402862	12	104313269	A	G	0.00	-1.14	0.14	2.67E-07
TXNRD1	rs12975366	19	54255498	C	T	0.40	-0.06	0.01	3.14E-07
TYMP	rs10774624	12	111395984	A	G	0.51	-0.05	0.01	5.29E-06
TYMP	rs131805	22	50525724	C	T	0.78	0.22	0.01	4.83E-59
TYRO3	rs3791101	1	43900578	A	G	0.31	0.05	0.01	2.48E-05
TYRO3	rs10935473	3	98698056	T	G	0.44	-0.23	0.01	7.68E-119
TYRO3	rs13107325	4	102267552	T	C	0.08	0.08	0.01	1.61E-05
TYRO3	-	9	133263362	G	A	0.18	-0.22	0.01	3.57E-69
TYRO3	rs174530	11	61779120	G	A	0.37	0.05	0.01	1.47E-06
TYRO3	rs3967200	11	126362490	T	C	0.13	-0.07	0.01	5.88E-07
TYRO3	rs8024626	15	41570983	A	G	0.30	-0.64	0.01	0.00E+00
TYRO3	rs186021206	17	7166093	A	G	0.01	0.97	0.04	1.02E-47
TYRO3	rs2008174	22	39464125	T	C	0.74	-0.09	0.01	5.17E-15
UBAC1	-	3	49275118	A	T	0.14	0.11	0.01	1.27E-10
UBAC1	rs112505519	9	135938274	C	G	0.00	-1.01	0.10	1.39E-11
ULBP2	rs4703854	5	72397636	T	C	0.76	0.07	0.01	2.55E-08
ULBP2	rs6924387	6	136761810	G	A	0.41	-0.05	0.01	4.27E-06
ULBP2	rs60340208	6	149980059	G	A	0.15	-0.68	0.01	0.00E+00
ULBP2	rs1115867	8	10781240	A	T	0.59	-0.07	0.01	1.20E-10
ULBP2	rs10104997	8	54527508	T	C	0.21	0.11	0.01	1.62E-19
ULBP2	rs677355	9	133270615	A	G	0.32	0.14	0.01	2.33E-36
ULBP2	rs371539188	16	68534225	T	C	0.76	0.06	0.01	5.47E-06
ULBP2	rs4804181	19	12398722	A	C	0.78	-0.11	0.01	1.28E-19
UMOD	rs4846828	1	217256289	A	G	0.62	-0.04	0.01	6.90E-06
UMOD	rs12465018	2	9096314	A	G	0.17	-0.06	0.01	1.69E-06
UMOD	rs72819488	2	95281351	A	G	0.16	-0.06	0.01	5.54E-07
UMOD	rs72831838	2	113258824	T	C	0.16	0.05	0.01	6.35E-06
UMOD	rs6793835	3	136101092	A	G	0.27	0.05	0.01	6.36E-06
UMOD	-	3	186932142	C	G	0.62	0.04	0.01	5.00E-06
UMOD	rs56012466	7	151709702	A	G	0.27	0.07	0.01	1.53E-11
UMOD	rs13288823	9	6386584	T	C	0.05	-0.09	0.01	1.82E-06
UMOD	rs774882452	11	61827448	C	T	0.34	-0.04	0.01	4.44E-06
UMOD	rs5792371	11	65789645	G	T	0.35	-0.08	0.01	1.23E-18
UMOD	rs12368376	12	515011	G	C	0.17	-0.06	0.01	3.81E-08
UMOD	rs11172134	12	57252006	A	T	0.21	0.05	0.01	1.48E-06
UMOD	rs10851885	15	76012162	G	A	0.26	-0.05	0.01	1.63E-06
UMOD	rs12922822	16	20356323	T	C	0.18	-0.96	0.01	0.00E+00
UMOD	rs186021206	17	7166093	A	G	0.01	0.29	0.04	2.18E-06
UMOD	rs2934951	17	39677075	G	A	0.68	-0.04	0.01	7.76E-06
UMOD	rs72835417	17	49164280	A	G	0.11	0.24	0.01	1.97E-67
UMOD	rs1997596	21	15206128	T	C	0.34	0.04	0.01	4.68E-06

USO1	rs1354034	3	56815721	C	T	0.60	0.06	0.01	6.66E-08
USO1	rs342298	7	106733200	T	C	0.45	-0.06	0.01	2.31E-06
USO1	rs6993770	8	105569300	T	A	0.29	-0.07	0.01	2.02E-07
USP8	rs78909033	2	240571486	A	G	0.14	-0.19	0.01	2.56E-29
USP8	rs1354034	3	56815721	C	T	0.60	0.07	0.01	3.91E-09
USP8	rs342298	7	106733200	T	C	0.46	-0.06	0.01	1.11E-06
USP8	rs60822569	12	54323724	C	T	0.55	0.05	0.01	2.53E-05
USP8	rs4380013	15	50467231	A	G	0.20	0.09	0.01	1.03E-09
UXS1	rs660240	1	109275216	C	T	0.79	0.06	0.01	8.77E-06
UXS1	rs138588539	2	106188136	T	C	0.08	0.18	0.01	1.97E-17
UXS1	rs68066031	2	224015781	C	T	0.23	-0.15	0.01	3.72E-28
UXS1	rs710446	3	186742138	C	T	0.41	-0.06	0.01	1.98E-06
UXS1	rs4253282	4	186243245	T	C	0.51	-0.12	0.01	1.56E-27
UXS1	rs2731673	5	177412897	C	T	0.74	-0.06	0.01	2.08E-06
UXS1	rs75694075	11	76783716	C	T	0.18	-0.24	0.01	1.26E-57
VAMP5	rs6547623	2	85574841	A	T	0.30	0.08	0.01	2.51E-10
VAMP5	rs505922	9	133273813	C	T	0.32	0.06	0.01	6.05E-07
VASH1	rs1354034	3	56815721	C	T	0.60	0.10	0.01	5.54E-18
VASH1	rs114694170	5	88884379	C	T	0.06	0.13	0.02	1.29E-07
VASH1	rs17622656	5	132485305	A	G	0.39	-0.05	0.01	2.37E-05
VASH1	rs11502185	11	180258	C	T	0.26	0.07	0.01	3.99E-06
VASH1	rs60822569	12	54323724	C	T	0.55	0.05	0.01	1.29E-05
VASH1	rs11159226	14	76715089	G	T	0.06	0.21	0.02	1.32E-17
VASN	rs150816167	1	179602727	C	T	0.04	0.14	0.02	4.91E-07
VASN	rs1260326	2	27508073	C	T	0.61	0.06	0.01	1.01E-08
VASN	rs199922514	8	9325592	G	A	0.91	-0.10	0.01	3.25E-07
VASN	rs7896518	10	63344740	G	A	0.42	0.05	0.01	5.12E-06
VASN	rs35713275	16	4381359	A	G	0.06	-0.90	0.02	0.00E+00
VASN	rs77542162	17	69085137	G	A	0.02	0.19	0.02	2.45E-07
VAT1	rs1047891	2	210675783	A	C	0.31	-0.07	0.01	5.55E-08
VAT1	rs11781667	8	143966688	T	G	0.36	0.06	0.01	3.41E-06
VAT1	rs12342201	9	93132682	A	G	0.48	-0.07	0.01	4.95E-10
VAT1	rs4149307	9	104827463	T	C	0.15	0.09	0.01	1.52E-08
VAT1	rs174564	11	61820833	G	A	0.35	0.05	0.01	2.22E-05
VAT1	rs190543502	15	43464986	C	T	0.02	-0.22	0.02	8.67E-09
VAT1	rs173539	16	56954132	T	C	0.33	0.06	0.01	2.79E-06
VAT1	rs200561116	16	67938291	T	A	0.12	0.10	0.01	5.44E-08
VAT1	rs7210098	17	43141596	T	C	0.34	-0.14	0.01	3.03E-32
VAT1	rs2868346	20	45919331	T	C	0.76	-0.15	0.01	2.23E-31
VCAM1	rs139561173	1	100679634	C	A	0.21	-0.10	0.01	3.85E-13
VCAM1	rs61801010	1	161572445	G	A	0.11	0.12	0.01	3.41E-10
VCAM1	rs2009581	2	111050100	A	G	0.27	-0.06	0.01	2.91E-06
VCAM1	rs1257169	2	134206291	A	C	0.50	-0.11	0.01	9.97E-23
VCAM1	rs35723031	2	181459315	T	G	0.56	-0.06	0.01	4.73E-07
VCAM1	rs28418426	6	32651877	C	T	0.53	0.06	0.01	5.99E-07
VCAM1	rs8176693	9	133262254	T	C	0.06	0.15	0.02	2.14E-10
VCAM1	rs2723889	12	103606692	C	T	0.67	0.07	0.01	1.28E-09
VCAM1	rs3184504	12	111446804	C	T	0.52	-0.14	0.01	1.76E-37
VCAM1	rs186021206	17	7166093	A	G	0.01	0.86	0.05	3.11E-29
VCAM1	rs34557412	17	16948873	G	A	0.01	0.32	0.04	2.89E-06
VCAM1	rs3747207	22	43928975	A	G	0.22	0.06	0.01	2.24E-05
VCAN	rs3754053	1	161606054	G	C	0.58	0.05	0.01	3.62E-07
VCAN	rs34552760	2	55962368	G	A	0.62	-0.09	0.01	2.92E-19
VCAN	rs1229984	4	99318162	C	T	0.98	-0.27	0.02	1.58E-15
VCAN	rs13107325	4	102267552	T	C	0.08	0.15	0.01	2.52E-15
VCAN	rs309559	5	83537550	G	A	0.48	-0.65	0.01	0.00E+00

VCAN	rs1917368	7	17872129	T	G	0.39	-0.05	0.01	5.52E-07
VCAN	rs3736590	7	100153658	T	G	0.56	0.06	0.01	6.23E-09
VCAN	rs10503665	8	19762823	A	G	0.09	-0.21	0.01	2.05E-35
VCAN	rs11783413	8	133537462	G	C	0.57	0.05	0.01	3.05E-06
VCAN	rs2435381	10	43183348	T	C	0.27	0.09	0.01	1.52E-14
VCAN	rs3138189	12	91171917	C	T	0.06	-0.10	0.01	1.33E-06
VCAN	rs11419060	15	101242185	G	T	0.19	-0.07	0.01	1.30E-08
VCAN	rs200489612	17	7203059	A	G	0.01	0.39	0.05	1.08E-07
VCAN	rs2665397	17	59749370	A	C	0.54	0.06	0.01	1.64E-08
VCAN	rs55639531	18	394341	G	C	0.29	0.06	0.01	8.29E-08
VEGFA	rs2274319	1	156481081	C	T	0.65	-0.06	0.01	7.73E-08
VEGFA	rs114694170	5	88884379	C	T	0.06	0.16	0.01	2.56E-15
VEGFA	rs6921438	6	43957870	A	G	0.49	-0.68	0.01	0.00E+00
VEGFA	rs13236689	7	80606698	G	T	0.41	0.05	0.01	5.11E-07
VEGFA	rs6993770	8	105569300	T	A	0.29	-0.22	0.01	1.43E-96
VEGFA	rs2375981	9	2692583	G	C	0.44	-0.19	0.01	1.20E-82
VEGFA	rs7075195	10	63290899	G	A	0.41	0.12	0.01	1.44E-36
VEGFA	rs3184504	12	111446804	C	T	0.52	-0.04	0.01	5.61E-06
VEGFA	rs9630357	14	33929731	T	C	0.18	0.06	0.01	2.53E-05
VEGFA	rs12445050	16	81837364	T	C	0.14	0.07	0.01	3.73E-07
VEGFA	rs34416903	16	88492837	C	T	0.28	0.08	0.01	8.01E-14
VEGFA	rs892090	19	55027704	G	T	0.83	0.11	0.01	6.88E-16
VEGFA	rs3827978	20	19300647	T	C	0.35	0.06	0.01	7.10E-08
VEGFC	rs7618405	3	18209017	A	C	0.20	-0.07	0.01	5.64E-06
VEGFC	rs1354034	3	56815721	C	T	0.60	-0.07	0.01	5.34E-10
VEGFC	rs10016018	4	101873464	T	A	0.40	-0.07	0.01	2.89E-08
VEGFC	rs35029317	4	176832273	G	A	0.01	-0.81	0.05	1.66E-27
VEGFC	rs11242109	5	132341354	T	G	0.48	-0.05	0.01	1.06E-05
VEGFC	rs2516471	6	31432515	A	G	0.06	-0.12	0.02	4.73E-07
VEGFC	rs1917342	6	70640051	G	A	0.30	-0.05	0.01	1.51E-05
VEGFC	rs6961069	7	80589645	T	C	0.40	0.07	0.01	3.21E-08
VEGFC	rs6993770	8	105569300	T	A	0.29	-0.09	0.01	1.16E-12
VEGFC	rs13298270	9	114011185	T	C	0.34	-0.05	0.01	2.37E-05
VEGFC	-	10	63433569	C	T	0.49	-0.08	0.01	3.40E-11
VEGFC	rs71474568	10	102589091	G	T	0.34	0.08	0.01	3.68E-11
VEGFC	rs73000929	11	114082900	A	G	0.04	-0.14	0.02	2.97E-06
VEGFC	rs1362213	12	6173262	T	C	0.48	-0.05	0.01	1.27E-05
VEGFC	rs61978213	14	70187041	A	G	0.04	0.12	0.02	1.95E-05
VEGFC	rs59001897	15	64868193	A	T	0.17	0.07	0.01	2.06E-06
VEGFC	rs12445050	16	81837364	T	C	0.14	0.11	0.01	1.46E-10
VEGFC	rs1654425	19	55027612	C	T	0.83	0.17	0.01	6.98E-29
VEGFC	rs6081555	20	19265079	T	G	0.34	0.07	0.01	1.86E-08
VEGFD	rs710446	3	186742138	C	T	0.41	0.06	0.01	4.10E-07
VEGFD	rs12331618	4	186218785	G	A	0.51	0.14	0.01	6.50E-35
VEGFD	rs3217225	5	109855234	T	C	0.17	-0.08	0.01	7.13E-08
VEGFD	rs2731674	5	177412889	G	T	0.74	0.08	0.01	1.79E-09
VEGFD	rs10982164	9	114328787	A	G	0.07	-0.12	0.02	7.00E-07
VEGFD	rs56278466	10	17833858	G	T	0.66	0.07	0.01	9.34E-09
VEGFD	rs1037117	15	101528455	A	G	0.25	-0.08	0.01	2.57E-10
VMO1	rs117779442	8	19982198	T	C	0.07	-0.08	0.01	8.55E-06
VMO1	rs112875651	8	125494452	A	G	0.39	-0.04	0.01	2.16E-06
VMO1	rs964184	11	116778201	C	G	0.87	-0.08	0.01	4.05E-11
VMO1	rs3794465	14	105903347	G	A	0.14	-0.11	0.01	3.96E-18
VMO1	rs261290	15	58386521	C	T	0.66	-0.13	0.01	3.34E-43
VMO1	rs8078118	17	4786743	A	C	0.42	0.75	0.01	0.00E+00
VMO1	rs3826688	19	44915704	C	T	0.64	0.05	0.01	6.68E-08

VNN2	rs11125915	2	62308853	C	A	0.64	0.05	0.01	1.03E-05
VNN2	rs9493425	6	132753506	G	A	0.32	-0.70	0.01	0.00E+00
VNN2	rs56278466	10	17833858	G	T	0.66	0.07	0.01	3.29E-10
VNN2	rs603424	10	100315722	A	G	0.17	-0.06	0.01	4.37E-06
VNN2	rs2511241	11	73234296	T	C	0.93	0.18	0.01	5.03E-20
VNN2	rs73017399	11	126316417	C	G	0.24	0.07	0.01	1.93E-10
VNN2	rs7310615	12	111427245	G	C	0.52	-0.04	0.01	2.06E-05
VNN2	rs146261845	17	7108935	T	C	0.01	0.29	0.04	1.12E-05
VNN2	rs56922390	17	40000768	G	C	0.39	0.10	0.01	1.85E-25
VNN2	rs4760	19	43648948	G	A	0.16	-0.18	0.01	1.93E-42
VPS37A	rs1354034	3	56815721	C	T	0.60	0.05	0.01	3.24E-06
VPS37A	rs964184	11	116778201	C	G	0.87	-0.07	0.01	1.77E-05
VPS53	rs1354034	3	56815721	C	T	0.60	0.06	0.01	1.26E-06
VPS53	rs342293	7	106731773	G	C	0.46	-0.05	0.01	4.89E-06
VSIG4	rs514591	1	196671190	G	A	0.23	-0.06	0.01	2.68E-06
VSIG4	rs11130306	3	52507237	G	A	0.48	0.05	0.01	1.75E-05
VSIG4	rs13146355	4	76490987	A	G	0.46	0.05	0.01	1.69E-05
VSIG4	rs12667978	7	129137407	T	C	0.57	0.05	0.01	2.58E-05
VSIG4	rs10769256	11	47356845	T	C	0.40	-0.10	0.01	2.40E-20
VSIG4	rs540639423	12	111407152	T	C	0.01	0.28	0.04	1.17E-05
VSIG4	rs28640218	16	20347945	T	G	0.19	-0.06	0.01	5.95E-06
VSIG4	-	17	59924072	A	C	0.14	0.08	0.01	3.60E-06
VSIG4	rs2230199	19	6718376	C	G	0.22	0.07	0.01	5.34E-07
VSIR	rs2278668	3	123116385	C	T	0.59	-0.05	0.01	2.39E-05
VSIR	rs10016018	4	101873464	T	A	0.40	-0.06	0.01	2.12E-06
VSIR	rs11242109	5	132341354	T	G	0.48	-0.06	0.01	1.98E-07
VSIR	rs6961069	7	80589645	T	C	0.40	0.06	0.01	2.09E-06
VSIR	rs342298	7	106733200	T	C	0.46	-0.06	0.01	7.90E-07
VSIR	rs6993770	8	105569300	T	A	0.29	-0.12	0.01	2.16E-22
VSIR	rs10999992	10	71769141	C	T	0.15	0.43	0.01	1.48E-162
VSIR	rs7956289	12	6184907	G	A	0.49	-0.05	0.01	1.42E-05
VSIR	rs1654425	19	55027612	C	T	0.83	0.10	0.01	1.14E-11
VSTM1	rs60816814	6	29920825	A	C	0.04	0.18	0.02	1.64E-13
VSTM1	rs188743906	17	7087693	T	C	0.01	0.25	0.04	2.00E-05
VSTM1	rs2433724	19	54042277	C	T	0.43	-0.77	0.01	0.00E+00
VSTM1	rs189448562	20	3724468	C	G	0.01	0.22	0.03	1.12E-06
VSTM2L	rs646776	1	109275908	T	C	0.78	0.21	0.01	3.49E-50
VSTM2L	rs2273349	20	37996354	A	C	0.18	-0.15	0.01	8.81E-22
VTA1	rs1354034	3	56815721	C	T	0.60	0.08	0.01	1.00E-10
VTA1	rs10900809	5	132490630	A	G	0.39	-0.06	0.01	8.47E-07
VTA1	rs10733789	10	63188924	C	T	0.31	0.06	0.01	5.29E-06
VTA1	rs60822569	12	54323724	C	T	0.55	0.06	0.01	2.35E-06
VTCN1	rs56174814	1	117180685	A	T	0.16	0.11	0.01	1.11E-10
VTCN1	rs679574	19	48702851	G	C	0.51	0.08	0.01	2.42E-11
VWA1	rs528749486	1	1499128	T	C	0.01	-1.50	0.03	3.16E-202
VWA1	-	3	155824602	A	T	0.25	0.07	0.01	5.84E-07
VWA1	rs35457250	3	186620775	T	C	0.01	0.52	0.04	3.20E-18
VWA1	rs6054	4	154568456	T	C	0.01	0.41	0.05	6.91E-08
VWA1	rs10843403	12	29387376	C	T	0.61	0.05	0.01	1.32E-05
VWA1	rs28929474	14	94378610	T	C	0.02	0.22	0.03	1.34E-08
VWA1	rs1801689	17	66214462	C	A	0.03	0.26	0.02	1.43E-15
VWA1	rs7256200	19	44912678	T	G	0.13	-0.08	0.01	1.00E-06
VWC2	rs61747728	1	179557079	T	C	0.04	0.13	0.02	1.70E-06
VWC2	rs141154056	3	52823924	T	C	0.00	1.78	0.08	4.29E-49
VWC2	rs769604	7	49775794	G	C	0.46	-0.45	0.01	0.00E+00
VWF	rs9390463	6	147381011	G	A	0.50	0.06	0.01	4.18E-08

VWF	rs2726953	8	27943788	A	G	0.30	0.06	0.01	9.48E-06
VWF	rs505922	9	133273813	C	T	0.32	0.31	0.01	4.50E-149
VWF	rs68120218	12	6032283	A	G	0.35	0.10	0.01	3.38E-16
VWF	rs3751198	12	103753429	G	A	0.60	0.06	0.01	2.70E-07
VWF	rs10744481	12	130792726	A	G	0.68	-0.05	0.01	1.91E-05
WARS	rs7974006	12	7367230	G	A	0.10	0.08	0.01	2.57E-05
WARS	rs7137828	12	111494996	T	C	0.52	-0.07	0.01	6.28E-11
WARS	rs2273804	14	100376300	G	C	0.26	-0.26	0.01	5.89E-89
WAS	rs5030082	3	186741160	G	A	0.40	0.06	0.01	4.13E-07
WAS	rs4861708	4	186236079	A	G	0.51	0.11	0.01	4.55E-22
WAS	rs1801020	5	177409531	G	A	0.74	0.08	0.01	1.64E-09
WASF1	rs1354034	3	56815721	C	T	0.60	0.05	0.01	2.06E-05
WASF1	rs1980532	6	110399006	G	A	0.45	-0.29	0.01	1.04E-138
WASF1	rs7916868	10	63229171	T	A	0.50	0.05	0.01	7.49E-06
WASF3	rs1018123	13	26640777	C	T	0.57	0.13	0.01	1.37E-30
WFDC12	rs61816761	1	152313385	A	G	0.02	0.73	0.02	6.81E-117
WFDC12	rs12030654	1	156751240	A	T	0.33	0.06	0.01	2.53E-08
WFDC12	rs17350445	3	187914551	T	C	0.06	-0.12	0.01	6.51E-11
WFDC12	rs7613473	3	190303767	A	G	0.59	0.07	0.01	4.25E-15
WFDC12	-	11	34718151	A	G	0.54	0.07	0.01	3.18E-13
WFDC12	rs12816349	12	6600779	A	G	0.24	-0.06	0.01	1.40E-09
WFDC12	rs10483947	14	80308772	T	C	0.09	0.08	0.01	1.35E-06
WFDC12	rs8038032	15	35010668	G	A	0.32	-0.09	0.01	2.15E-18
WFDC12	rs35673728	15	40755579	C	T	0.06	-0.17	0.01	6.59E-21
WFDC12	rs562177063	16	31131513	T	C	0.00	-0.62	0.06	1.07E-12
WFDC12	rs6508781	19	38294678	C	A	0.17	-0.13	0.01	1.05E-28
WFDC12	rs75002084	20	45126666	G	C	0.43	-0.43	0.01	0.00E+00
WFDC12	rs714781	21	38806457	T	C	0.33	-0.06	0.01	3.54E-10
WFDC2	rs77924615	16	20381010	A	G	0.20	-0.07	0.01	2.01E-07
WFDC2	rs2638282	19	48710576	A	G	0.53	0.06	0.01	3.63E-08
WFDC2	rs973446	20	45465735	G	C	0.08	-0.14	0.01	4.94E-12
WFIKKN1	rs760192241	1	24768903	C	A	0.55	-0.06	0.01	3.96E-08
WFIKKN1	rs55944332	2	144969054	G	A	0.24	0.06	0.01	8.45E-06
WFIKKN1	rs11676298	2	226427015	G	C	0.19	-0.08	0.01	7.98E-10
WFIKKN1	rs76311404	3	123380282	A	G	0.22	0.07	0.01	5.21E-08
WFIKKN1	rs34250475	4	153508890	T	C	0.45	0.05	0.01	5.08E-06
WFIKKN1	rs27659	5	97007848	G	A	0.55	-0.05	0.01	1.45E-05
WFIKKN1	rs2647074	6	32606583	T	C	0.33	0.09	0.01	1.43E-15
WFIKKN1	rs3731211	9	21986848	A	T	0.72	-0.08	0.01	8.36E-12
WFIKKN1	rs183805019	9	99584725	C	T	0.00	-0.46	0.06	5.32E-07
WFIKKN1	rs4937333	11	128460625	C	T	0.53	0.05	0.01	1.77E-06
WFIKKN1	rs7955466	12	6381950	T	C	0.36	0.05	0.01	5.01E-06
WFIKKN1	rs11513729	12	111835695	T	C	0.41	0.07	0.01	6.53E-11
WFIKKN1	rs55798945	16	619708	G	T	0.30	0.27	0.01	1.06E-112
WFIKKN1	rs34557412	17	16948873	G	A	0.01	0.39	0.04	1.77E-09
WFIKKN1	rs6077396	20	8623946	A	G	0.51	0.11	0.01	1.10E-26
WFIKKN2	rs6664906	1	30881966	C	T	0.46	-0.05	0.01	1.49E-05
WFIKKN2	rs4546329	5	61293912	T	C	0.50	-0.05	0.01	4.86E-07
WFIKKN2	rs4591185	17	50832073	A	T	0.66	-0.67	0.01	0.00E+00
WFIKKN2	rs33950747	19	35848345	T	C	0.08	0.14	0.01	4.46E-13
WIF1	rs1414660	1	240423395	T	C	0.19	0.09	0.01	6.85E-10
WIF1	rs6768977	3	12466670	G	A	0.53	0.06	0.01	6.73E-07
WIF1	rs17613549	6	32662910	C	G	0.22	-0.07	0.01	2.26E-06
WIF1	rs28457828	8	54538582	T	C	0.21	-0.10	0.01	1.13E-13
WIF1	rs10963680	9	18629285	A	G	0.24	0.06	0.01	1.26E-05
WIF1	-	9	133263362	G	A	0.18	-0.14	0.01	5.77E-22

WIF1	-	10	24911876	A	C	0.37	-0.05	0.01	9.43E-06
WIF1	rs7896518	10	63344740	G	A	0.42	0.06	0.01	3.72E-08
WIF1	rs10741178	10	129104740	C	T	0.78	0.06	0.01	9.48E-06
WIF1	rs7949566	11	126415406	A	G	0.42	0.07	0.01	1.87E-09
WIF1	rs6489548	12	479438	G	A	0.60	-0.19	0.01	3.92E-63
WIF1	rs462010	12	64944365	A	G	0.44	-0.19	0.01	3.97E-67
WIF1	rs12894709	14	74975031	A	G	0.47	-0.05	0.01	1.25E-06
WIF1	rs117068593	14	92651884	T	C	0.19	-0.08	0.01	3.98E-08
WIF1	rs186021206	17	7166093	A	G	0.01	0.49	0.05	1.43E-10
WIF1	rs2145943	20	39934603	G	A	0.41	0.10	0.01	2.66E-19
WNT9A	rs6690181	1	227869167	C	T	0.38	-0.13	0.01	3.86E-31
WNT9A	rs9274370	6	32664750	A	C	0.14	-0.08	0.01	5.66E-07
WWP2	rs1354034	3	56815721	C	T	0.60	0.08	0.01	5.58E-13
WWP2	rs342293	7	106731773	G	C	0.46	-0.06	0.01	4.02E-07
WWP2	rs72783174	16	69775775	T	C	0.08	-0.31	0.01	8.87E-50
XCL1	rs4656599	1	168534148	T	C	0.19	0.83	0.01	0.00E+00
XCL1	rs2009581	2	111050100	A	G	0.27	-0.15	0.01	1.29E-42
XCL1	rs13063578	3	47046347	A	T	0.40	-0.09	0.01	2.69E-19
XCL1	rs2548993	5	132473177	A	G	0.72	0.07	0.01	2.36E-09
XCL1	rs9265813	6	31342763	T	A	0.17	0.08	0.01	1.82E-08
XCL1	rs705379	7	95324583	A	G	0.48	0.06	0.01	4.18E-10
XCL1	rs8177653	10	5973418	G	A	0.33	0.05	0.01	4.33E-07
XCL1	rs3184504	12	111446804	C	T	0.52	-0.10	0.01	2.03E-24
XCL1	-	17	39907727	C	A	0.51	-0.06	0.01	1.07E-09
XCL1	rs77542162	17	69085137	G	A	0.02	0.26	0.02	5.75E-16
XCL1	rs547843340	19	16398994	T	G	0.13	-0.06	0.01	2.72E-05
XCL1	rs17561351	19	44869072	G	A	0.06	-0.09	0.01	6.40E-06
XCL1	rs6031304	20	44028908	C	T	0.73	0.05	0.01	1.16E-05
XG	rs11178645	12	71129854	A	T	0.41	-0.05	0.01	1.45E-09
XPNPEP2	rs114165349	1	26695422	C	G	0.02	0.42	0.02	1.13E-29
XPNPEP2	rs1260326	2	27508073	C	T	0.61	-0.07	0.01	3.42E-09
XPNPEP2	rs1584300	2	61358866	C	A	0.15	0.08	0.01	2.61E-06
XPNPEP2	rs687339	3	136213517	T	C	0.77	0.06	0.01	1.65E-05
XPNPEP2	rs5860563	4	99126006	A	C	0.70	-0.08	0.01	4.06E-11
XPNPEP2	rs4323324	6	130061751	A	G	0.58	0.07	0.01	9.39E-11
XPNPEP2	rs10822143	10	63128096	T	C	0.50	-0.06	0.01	2.97E-07
XPNPEP2	rs11621792	14	24402720	T	C	0.45	0.08	0.01	2.52E-11
XPNPEP2	rs2498786	14	104796031	G	C	0.62	-0.08	0.01	1.25E-12
XPNPEP2	rs339969	15	60591082	A	C	0.62	0.09	0.01	2.58E-16
XPNPEP2	-	16	79720536	T	A	0.31	-0.16	0.01	2.06E-40
XPNPEP2	-	17	47500653	C	A	0.50	-0.09	0.01	4.97E-15
XPNPEP2	rs60018147	19	3375574	G	A	0.12	-0.09	0.01	3.36E-06
XPNPEP2	rs2207132	20	40513876	A	G	0.03	-0.22	0.02	1.57E-11
XPNPEP2	rs1800961	20	44413724	T	C	0.03	-0.23	0.02	7.92E-13
XRCC4	rs1805377	5	83353124	A	G	0.11	0.10	0.01	1.24E-07
YES1	rs1354034	3	56815721	C	T	0.60	0.06	0.01	2.40E-07
YES1	rs2741189	18	707511	C	G	0.23	0.09	0.01	1.76E-10
YTHDF3	rs1354034	3	56815721	C	T	0.60	0.09	0.01	4.69E-13
YTHDF3	rs11502185	11	180258	C	T	0.26	0.07	0.01	2.27E-06
ZBTB16	rs1354034	3	56815721	C	T	0.60	0.07	0.01	5.97E-09
ZBTB16	rs915652	6	31781365	A	G	0.13	-0.08	0.01	7.15E-06
ZBTB16	rs342292	7	106730198	G	C	0.46	-0.06	0.01	2.96E-07
ZBTB16	rs6993770	8	105569300	T	A	0.29	-0.06	0.01	2.02E-06
ZBTB16	rs12767683	10	102556824	A	C	0.32	0.08	0.01	1.91E-11
ZBTB16	rs73000929	11	114082900	A	G	0.04	0.85	0.02	8.05E-183
ZBTB16	rs7297265	12	54325905	G	A	0.44	0.08	0.01	2.89E-12

ZBTB17	rs7080536	10	113588287	A	G	0.04	-0.16	0.02	5.75E-08
ZBTB17	rs7260293	19	52726141	T	C	0.62	0.07	0.01	1.19E-09

Supplementary Table 2. Associations of genetically predicted proteins with severe COVID-19 using IVW method and cis+trans SNPs.

Protein	No. of SNPs	Beta	SE	Pval
AARSD1	3	0.10	0.10	3.03E-01
ABHD14B	4	-0.05	0.05	3.18E-01
ABL1	1	0.16	0.17	3.51E-01
ACAA1	3	-0.11	0.12	3.73E-01
ACAN	15	-0.02	0.05	6.58E-01
ACE2	9	-0.06	0.09	4.65E-01
ACOX1	1	-0.25	0.20	2.09E-01
ACP5	9	0.04	0.04	2.98E-01
ACP6	2	0.00	0.02	8.70E-01
ACTA2	1	0.40	0.26	1.19E-01
ACVRL1	3	0.05	0.04	2.27E-01
ACY1	3	0.08	0.09	3.79E-01
ADA	4	0.00	0.02	9.35E-01
ADA2	13	-0.10	0.05	4.38E-02
ADAM15	7	-0.02	0.02	1.87E-01
ADAM22	6	0.03	0.03	4.46E-01
ADAM23	5	0.02	0.03	4.50E-01
ADAM8	7	0.02	0.03	4.24E-01
ADAMTS13	11	0.03	0.04	4.61E-01
ADAMTS15	5	0.05	0.09	5.81E-01
ADAMTS16	1	0.03	0.12	8.11E-01
ADAMTS8	4	0.00	0.03	9.07E-01
ADGRB3	5	0.02	0.02	2.97E-01
ADGRE2	7	-0.01	0.05	7.84E-01
ADGRE5	8	-0.03	0.05	6.16E-01
ADGRG1	9	-0.27	0.07	1.36E-04
ADGRG2	6	0.57	0.11	3.73E-07
ADH4	6	-0.02	0.08	7.59E-01
AFP	21	-0.02	0.03	4.75E-01
AGER	15	0.08	0.06	1.60E-01
AGR2	4	0.16	0.10	1.03E-01
AGR3	3	0.16	0.15	2.91E-01
AGRN	6	0.03	0.05	4.96E-01
AGRP	8	-0.04	0.08	6.56E-01
AGXT	11	-0.04	0.04	3.52E-01
AHCY	1	-0.03	0.04	3.90E-01
AHSP	7	0.13	0.09	1.39E-01
AIF1	1	0.29	0.13	2.10E-02
AIFM1	2	0.01	0.19	9.48E-01
AKR1B1	2	-0.09	0.10	3.53E-01
AKR1C4	1	-0.18	0.28	5.22E-01
AKT1S1	3	-0.18	0.13	1.75E-01
AKT3	2	-0.42	0.22	5.13E-02
ALCAM	11	-0.04	0.06	5.78E-01
ALDH1A1	3	-0.05	0.16	7.40E-01
ALDH3A1	1	-0.02	0.03	5.68E-01
ALPP	10	0.08	0.03	1.03E-02
AMBP	8	-0.03	0.07	6.89E-01
AMIGO2	11	-0.09	0.04	2.43E-02
AMN	5	0.02	0.02	4.13E-01

AMY2A	8	-0.14	0.04	5.34E-04
AMY2B	9	-0.16	0.03	1.18E-06
ANG	3	0.04	0.03	1.84E-01
ANGPT1	17	0.05	0.04	2.59E-01
ANGPT2	6	0.15	0.07	2.76E-02
ANGPTL1	6	0.04	0.06	4.49E-01
ANGPTL2	5	0.12	0.09	2.14E-01
ANGPTL3	6	-0.07	0.09	4.75E-01
ANGPTL4	1	0.03	0.05	5.34E-01
ANGPTL7	6	0.02	0.07	7.37E-01
ANKRD54	1	0.05	0.24	8.33E-01
ANPEP	7	0.05	0.03	1.39E-01
ANXA11	3	-0.37	0.18	4.34E-02
ANXA3	5	-0.17	0.11	1.02E-01
ANXA4	2	-0.20	0.16	2.31E-01
ANXA5	1	0.23	0.13	7.71E-02
AOC1	1	0.01	0.06	8.49E-01
AOC3	10	0.03	0.03	2.46E-01
APBB1IP	5	0.01	0.05	8.04E-01
APEX1	3	-0.04	0.05	4.21E-01
APLP1	5	-0.01	0.09	8.77E-01
APOH	1	0.00	0.03	9.40E-01
APOM	9	0.12	0.04	3.64E-03
APP	17	0.06	0.05	2.37E-01
APRT	6	-0.01	0.09	9.31E-01
AREG	3	0.36	0.08	5.12E-06
ARG1	5	0.02	0.06	7.94E-01
ARHGAP1	2	-0.31	0.14	2.35E-02
ARHGAP25	2	0.10	0.14	4.89E-01
ARSA	7	0.02	0.02	4.32E-01
ARSB	11	0.07	0.05	1.33E-01
ART3	4	0.22	0.12	6.83E-02
ARTN	1	-0.43	0.30	1.46E-01
ASAH2	16	-0.03	0.02	2.79E-01
ASGR1	6	0.01	0.06	8.33E-01
ATF2	2	-0.08	0.13	5.10E-01
ATG4A	3	0.32	0.14	2.69E-02
ATOX1	5	0.15	0.09	1.13E-01
ATP5IF1	6	0.04	0.09	6.80E-01
ATP6V1F	1	0.21	0.22	3.51E-01
ATXN10	5	-0.14	0.09	1.15E-01
AXIN1	3	-0.08	0.16	6.24E-01
AXL	8	-0.07	0.06	2.02E-01
AZU1	2	-0.10	0.20	6.16E-01
B4GALT1	6	0.03	0.04	4.35E-01
B4GAT1	9	0.02	0.04	5.65E-01
BACH1	3	-0.08	0.16	5.94E-01
BAG3	4	0.22	0.11	4.64E-02
BAIAP2	5	-0.04	0.10	6.63E-01
BAMBI	1	-0.20	0.22	3.51E-01
BANK1	3	0.07	0.11	5.11E-01
BAX	2	-0.22	0.18	2.00E-01

BCAM	14	-0.01	0.03	7.35E-01
BCAN	7	0.11	0.05	4.24E-02
BCL2L11	10	0.02	0.04	5.30E-01
BCR	1	0.30	0.36	4.09E-01
BGN	11	0.02	0.06	6.95E-01
BID	2	-0.07	0.17	6.77E-01
BIN2	3	0.01	0.12	9.26E-01
BIRC2	4	-0.25	0.15	9.07E-02
BLMH	3	-0.02	0.03	4.57E-01
BLVRB	2	0.04	0.11	7.06E-01
BMP4	5	-0.08	0.11	4.90E-01
BMP6	15	-0.02	0.04	6.21E-01
BOC	8	-0.03	0.07	6.34E-01
BPIFB1	15	0.03	0.03	2.93E-01
BRK1	8	0.01	0.06	8.46E-01
BSG	10	-0.16	0.06	8.55E-03
BST1	4	-0.01	0.01	5.37E-01
BST2	11	-0.02	0.06	7.60E-01
BTC	3	-0.01	0.03	8.06E-01
BTN2A1	7	0.04	0.05	4.38E-01
BTN3A2	3	-0.04	0.02	1.25E-01
C19orf12	5	0.04	0.12	7.44E-01
C1QA	13	-0.01	0.04	7.39E-01
C1QTNF1	4	0.07	0.07	2.71E-01
C2	2	0.09	0.15	5.39E-01
C2CD2L	1	-0.20	0.28	4.73E-01
C4BPB	10	0.01	0.05	8.33E-01
CA1	4	0.04	0.08	6.40E-01
CA11	2	-0.10	0.11	4.00E-01
CA12	7	0.03	0.05	4.91E-01
CA13	3	-0.11	0.06	4.47E-02
CA14	5	-0.03	0.07	6.87E-01
CA2	2	-0.03	0.13	8.16E-01
CA3	6	-0.07	0.07	2.95E-01
CA4	9	0.08	0.04	1.71E-02
CA5A	5	-0.04	0.02	5.96E-02
CA6	12	-0.03	0.02	1.31E-01
CA9	5	0.07	0.08	3.83E-01
CALB1	3	0.00	0.12	9.78E-01
CALCA	7	-0.07	0.09	4.51E-01
CALCOCO1	4	-0.06	0.11	6.17E-01
CAMKK1	3	-0.02	0.05	6.29E-01
CANT1	7	0.16	0.07	2.70E-02
CAPG	2	0.02	0.02	2.36E-01
CARHSP1	1	0.17	0.18	3.29E-01
CASP1	1	-0.03	0.06	5.69E-01
CASP10	3	-0.02	0.08	8.33E-01
CASP2	2	0.16	0.14	2.62E-01
CASP3	2	0.04	0.14	7.54E-01
CASP8	3	0.05	0.06	3.96E-01
CBLIF	17	-0.09	0.03	2.71E-03
CBLN4	5	0.05	0.07	4.98E-01

CC2D1A	2	0.37	0.17	3.43E-02
CCDC80	5	0.20	0.08	1.07E-02
CCL11	9	-0.10	0.05	3.99E-02
CCL13	14	0.05	0.04	1.76E-01
CCL14	6	0.02	0.03	4.45E-01
CCL15	4	0.38	0.07	4.40E-08
CCL16	8	0.02	0.02	4.34E-01
CCL17	14	0.01	0.03	7.18E-01
CCL18	4	0.04	0.03	2.22E-01
CCL19	5	-0.05	0.09	5.84E-01
CCL2	8	0.08	0.04	6.40E-02
CCL20	5	-0.14	0.09	1.28E-01
CCL21	10	-0.13	0.07	7.47E-02
CCL22	8	-0.17	0.07	1.73E-02
CCL23	6	-0.04	0.04	2.78E-01
CCL24	11	0.01	0.02	6.12E-01
CCL25	9	0.06	0.02	1.84E-03
CCL26	7	-0.01	0.05	9.10E-01
CCL27	7	-0.07	0.06	2.11E-01
CCL28	18	-0.08	0.04	7.55E-02
CCL3	6	0.10	0.11	3.67E-01
CCL4	5	-0.14	0.04	1.26E-04
CCL5	9	-0.01	0.05	9.04E-01
CCL7	4	-0.05	0.04	2.83E-01
CCL8	10	0.02	0.02	1.86E-01
CCN1	11	-0.05	0.06	3.85E-01
CCN2	16	0.04	0.04	2.82E-01
CCN3	3	-0.07	0.06	2.27E-01
CCN4	12	-0.05	0.03	1.33E-01
CCN5	1	-0.02	0.29	9.43E-01
CCS	1	0.02	0.03	4.85E-01
CD109	6	-0.43	0.07	1.02E-10
CD14	6	0.02	0.05	6.88E-01
CD160	13	-0.02	0.03	4.96E-01
CD163	18	-0.03	0.04	4.21E-01
CD164	10	0.01	0.06	8.21E-01
CD177	3	-0.01	0.03	7.91E-01
CD1C	18	-0.10	0.04	1.86E-02
CD200	8	-0.06	0.06	3.02E-01
CD200R1	9	0.00	0.02	7.95E-01
CD207	15	-0.02	0.03	4.64E-01
CD209	8	0.11	0.02	3.79E-11
CD22	20	-0.04	0.04	3.72E-01
CD244	13	-0.08	0.04	6.60E-02
CD27	6	0.00	0.05	9.80E-01
CD274	7	0.02	0.04	6.14E-01
CD276	7	-0.01	0.02	6.34E-01
CD28	10	0.05	0.05	2.56E-01
CD2AP	1	0.21	0.22	3.51E-01
CD300C	14	0.03	0.04	4.19E-01
CD300E	10	-0.06	0.04	1.20E-01
CD300LF	9	0.01	0.02	5.74E-01

CD300LG	12	0.05	0.03	1.27E-01
CD302	6	-0.01	0.04	8.55E-01
CD33	7	0.00	0.01	7.21E-01
CD34	3	-0.23	0.04	1.39E-09
CD38	2	-0.04	0.04	3.40E-01
CD4	4	-0.12	0.04	2.11E-03
CD40	9	-0.07	0.03	2.89E-02
CD40LG	12	0.18	0.06	1.84E-03
CD46	7	-0.17	0.08	3.91E-02
CD48	13	-0.02	0.02	2.45E-01
CD5	8	-0.06	0.06	2.95E-01
CD55	6	0.00	0.03	8.88E-01
CD58	12	-0.17	0.03	2.20E-08
CD59	4	0.02	0.06	7.40E-01
CD6	11	-0.01	0.03	8.17E-01
CD63	9	-0.01	0.06	8.86E-01
CD69	6	-0.02	0.08	7.69E-01
CD70	3	0.00	0.02	9.57E-01
CD74	6	-0.03	0.06	6.37E-01
CD79B	17	-0.12	0.04	3.59E-03
CD83	13	-0.06	0.05	2.49E-01
CD84	11	-0.05	0.05	2.62E-01
CD8A	5	-0.01	0.04	8.35E-01
CD93	5	-0.21	0.10	3.24E-02
CD99	4	0.15	0.10	1.24E-01
CD99L2	9	0.02	0.06	7.51E-01
CDC27	2	0.08	0.15	5.88E-01
CDC37	2	-0.01	0.17	9.60E-01
CDCP1	8	-0.14	0.05	4.87E-03
CDH1	6	0.05	0.03	4.24E-02
CDH15	4	0.30	0.05	1.03E-10
CDH17	12	-0.01	0.02	6.36E-01
CDH2	13	0.01	0.06	9.22E-01
CDH3	7	-0.12	0.09	1.75E-01
CDH5	10	0.01	0.02	7.85E-01
CDH6	6	0.05	0.03	4.92E-02
CDHR1	4	0.04	0.06	5.29E-01
CDHR2	8	0.06	0.10	5.15E-01
CDHR5	8	-0.06	0.03	2.30E-02
CDKN1A	5	-0.07	0.08	3.43E-01
CDKN2D	4	0.09	0.09	3.14E-01
CDNF	1	0.01	0.04	8.16E-01
CDON	4	0.11	0.11	3.29E-01
CDSN	16	-0.07	0.05	2.07E-01
CEACAM1	6	-0.02	0.05	6.32E-01
CEACAM21	2	-0.43	0.30	1.52E-01
CEACAM3	1	-0.20	0.24	4.07E-01
CEACAM5	11	-0.11	0.03	1.50E-04
CEACAM8	12	-0.06	0.05	2.37E-01
CEBPB	2	0.36	0.19	5.33E-02
CELA3A	15	-0.10	0.03	1.76E-03
CEP20	4	0.08	0.10	4.23E-01

CEP43	6	-0.12	0.10	2.29E-01
CEP85	2	0.04	0.11	7.05E-01
CERT	4	0.09	0.12	4.22E-01
CES1	18	0.07	0.04	6.51E-02
CES2	2	-0.15	0.15	3.18E-01
CES3	9	-0.10	0.06	9.23E-02
CETN2	3	0.03	0.12	8.37E-01
CFC1	8	0.12	0.09	1.74E-01
CGA	1	-0.47	0.38	2.11E-01
CGREF1	3	0.04	0.02	1.00E-01
CHAC2	2	0.41	0.14	3.24E-03
CHGB	6	-0.05	0.02	4.81E-02
CHI3L1	6	0.08	0.03	1.88E-02
CHIT1	4	-0.03	0.02	1.07E-01
CHL1	6	0.00	0.04	9.89E-01
CHMP1A	4	0.07	0.12	5.62E-01
CHRDL1	4	-0.06	0.15	6.91E-01
CHRDL2	4	-0.02	0.15	8.76E-01
CIAPIN1	4	-0.20	0.12	1.04E-01
CKAP4	4	-0.03	0.04	4.87E-01
MT1A_CKMT	6	0.38	0.09	1.75E-05
CLC	9	-0.03	0.06	5.81E-01
CLEC10A	9	0.02	0.02	4.97E-01
CLEC11A	7	-0.08	0.05	9.93E-02
CLEC14A	7	-0.17	0.04	9.24E-05
CLEC1A	6	0.01	0.03	7.70E-01
CLEC1B	9	0.10	0.05	5.66E-02
CLEC4A	11	0.00	0.03	9.21E-01
CLEC4C	25	0.02	0.02	3.10E-01
CLEC4D	10	-0.01	0.02	8.13E-01
CLEC4G	10	0.19	0.04	6.24E-07
CLEC5A	10	0.03	0.04	4.20E-01
CLEC6A	13	0.01	0.03	7.62E-01
CLEC7A	2	0.01	0.02	4.23E-01
CLIP2	2	0.06	0.10	5.89E-01
CLMP	4	0.16	0.06	7.11E-03
CLPS	7	-0.03	0.04	3.54E-01
CLSPN	1	0.06	0.15	6.88E-01
CLSTN2	3	0.03	0.06	6.25E-01
CLTA	1	0.06	0.12	6.09E-01
CLUL1	8	0.00	0.03	9.77E-01
CNDP1	8	-0.06	0.03	4.51E-02
CNPY2	1	-0.06	0.12	6.28E-01
CNPY4	3	-0.05	0.07	5.37E-01
CNST	4	-0.03	0.14	8.08E-01
CNTN1	7	0.09	0.04	2.52E-02
CNTN2	5	0.20	0.12	1.03E-01
CNTN3	16	-0.01	0.04	7.14E-01
CNTN4	10	-0.01	0.05	8.85E-01
CNTN5	6	-0.04	0.04	4.22E-01
CNTNAP2	4	0.03	0.04	4.19E-01
COL18A1	4	-0.07	0.08	3.98E-01

COL1A1	9	-0.10	0.07	1.57E-01
COL4A1	9	0.01	0.04	8.13E-01
COL6A3	4	0.01	0.14	9.15E-01
COL9A1	3	0.24	0.14	8.16E-02
COLEC12	4	0.30	0.15	4.13E-02
COMP	6	0.05	0.06	3.99E-01
COMT	2	-0.04	0.03	1.63E-01
CORO1A	3	0.06	0.15	6.97E-01
COX5B	4	-0.20	0.14	1.56E-01
CPA1	21	-0.06	0.04	1.66E-01
CPA2	4	0.03	0.04	5.23E-01
CPB1	13	-0.05	0.04	2.33E-01
CPE	7	-0.15	0.06	1.10E-02
CPM	11	0.01	0.07	9.20E-01
CPPED1	1	-0.01	0.02	8.12E-01
CPVL	12	0.02	0.02	3.23E-01
CPXM1	18	-0.01	0.03	6.92E-01
CR2	23	0.01	0.04	7.86E-01
CRACR2A	4	-0.04	0.09	6.50E-01
CRADD	4	-0.22	0.12	6.81E-02
CREG1	11	0.01	0.05	8.13E-01
CRELD2	11	0.00	0.04	9.36E-01
CRH	23	-0.03	0.03	3.73E-01
CRHBP	5	0.02	0.03	4.55E-01
CRIM1	4	-0.40	0.14	3.37E-03
CRIP2	4	0.17	0.09	5.83E-02
CRISP2	13	-0.02	0.03	5.56E-01
CRKL	1	-0.10	0.23	6.57E-01
CRLF1	7	-0.07	0.11	5.08E-01
CRNN	3	0.04	0.02	6.15E-02
CRTAC1	2	0.00	0.02	9.13E-01
CRTAM	14	-0.06	0.04	1.36E-01
CSF1	3	-0.08	0.07	2.16E-01
CSF2RA	1	0.10	0.32	7.58E-01
CSF3	3	-0.11	0.10	2.46E-01
CST3	6	-0.03	0.07	6.76E-01
CST5	13	0.02	0.02	4.04E-01
CST6	15	0.02	0.05	7.00E-01
CST7	11	0.00	0.02	9.73E-01
CTF1	2	-0.03	0.22	8.84E-01
CTRB1	12	-0.05	0.03	9.08E-02
CTRC	11	-0.03	0.02	2.51E-01
CTSB	7	0.03	0.03	4.12E-01
CTSC	6	0.02	0.02	4.07E-01
CTSD	6	0.03	0.04	4.25E-01
CTSF	15	0.02	0.04	6.37E-01
CTSH	1	0.06	0.16	7.27E-01
CTSL	17	-0.03	0.05	5.83E-01
CTSO	10	-0.03	0.05	5.83E-01
CTSS	7	-0.24	0.08	2.68E-03
CTSV	17	0.00	0.04	9.75E-01
CTSZ	9	0.01	0.04	7.38E-01

CX3CL1	7	-0.56	0.09	3.62E-10
CXADR	5	0.23	0.06	3.02E-05
CXCL1	4	0.00	0.03	9.45E-01
CXCL10	4	-0.04	0.06	5.63E-01
CXCL11	8	-0.05	0.06	3.98E-01
CXCL12	9	-0.11	0.07	1.16E-01
CXCL13	7	-0.12	0.10	2.49E-01
CXCL14	2	0.13	0.17	4.22E-01
CXCL16	13	0.05	0.05	3.02E-01
CXCL17	5	-0.36	0.12	2.73E-03
CXCL3	3	-0.07	0.12	5.55E-01
CXCL5	11	-0.02	0.03	4.59E-01
CXCL6	7	-0.02	0.03	4.70E-01
CXCL8	8	0.12	0.06	5.88E-02
CXCL9	5	-0.02	0.07	7.37E-01
DAB2	3	-0.43	0.15	3.31E-03
DAG1	3	0.43	0.16	7.70E-03
DAPP1	2	0.19	0.12	1.22E-01
DARS1	5	-0.08	0.11	4.51E-01
DBI	3	0.13	0.05	1.67E-02
DBNL	4	0.02	0.13	8.80E-01
DCBLD2	16	-0.04	0.04	3.75E-01
DCN	1	-0.33	0.23	1.43E-01
DCTN1	1	-0.08	0.22	7.09E-01
DCTN2	2	0.00	0.20	9.90E-01
DCTPP1	5	0.02	0.09	8.42E-01
DCXR	6	0.06	0.09	5.04E-01
DDAH1	5	0.07	0.07	3.32E-01
DDC	7	0.00	0.04	9.88E-01
DDR1	12	-0.05	0.03	1.47E-01
DDX58	1	0.04	0.08	6.09E-01
DECR1	3	0.09	0.14	5.07E-01
EFA1_DEFA1	15	0.05	0.06	4.30E-01
EFB4A_DEFB4	3	0.09	0.15	5.45E-01
DFFA	1	0.21	0.22	3.51E-01
DIABLO	3	-0.21	0.16	1.86E-01
DKK1	10	0.05	0.05	3.22E-01
DKK3	7	-0.07	0.04	5.78E-02
DKK4	6	0.04	0.05	4.61E-01
DKKL1	5	-0.01	0.01	6.30E-01
DLK1	6	-0.01	0.03	7.24E-01
DLL1	6	0.05	0.07	4.30E-01
DNAJA2	1	-0.22	0.23	3.38E-01
DNAJB1	6	0.01	0.10	9.15E-01
DNAJB8	2	0.18	0.16	2.58E-01
DNER	7	0.00	0.05	9.53E-01
DNMBP	3	0.07	0.13	6.01E-01
DNPH1	1	-0.06	0.05	2.59E-01
DOK2	4	-0.14	0.11	2.10E-01
DPEP1	7	0.00	0.02	9.70E-01
DPEP2	3	-0.03	0.09	7.12E-01
DPP10	5	-0.10	0.05	3.79E-02

DPP4	5	0.06	0.06	3.50E-01
DPP6	7	-0.10	0.05	3.80E-02
DPP7	7	-0.02	0.06	7.19E-01
DPT	1	0.03	0.04	5.42E-01
DPY30	2	-0.24	0.18	1.95E-01
DRAXIN	17	-0.11	0.05	1.40E-02
DRG2	3	0.20	0.10	5.00E-02
DSC2	9	0.04	0.05	3.66E-01
DSG2	10	0.00	0.04	9.07E-01
DSG3	17	0.07	0.04	7.71E-02
DSG4	22	-0.05	0.04	2.44E-01
DTX3	3	0.01	0.07	8.70E-01
DUSP3	1	-0.58	0.23	1.10E-02
EBAG9	5	0.09	0.10	3.65E-01
EBI3_IL27	19	-0.02	0.04	5.86E-01
ECE1	4	-0.04	0.10	6.74E-01
EDA2R	3	0.43	0.18	1.71E-02
EDAR	23	0.03	0.03	3.18E-01
EDIL3	3	0.07	0.10	5.11E-01
EFEMP1	4	-0.08	0.07	2.34E-01
EFNA1	4	0.07	0.04	8.09E-02
EFNA4	5	-0.02	0.10	8.10E-01
EGF	11	0.09	0.06	1.11E-01
EGFL7	18	0.00	0.04	9.42E-01
EGFR	6	0.09	0.09	3.40E-01
EGLN1	4	-0.10	0.08	2.42E-01
EIF4B	3	-0.13	0.12	3.06E-01
EIF4G1	3	0.01	0.12	9.56E-01
ELOA	2	-0.02	0.10	8.37E-01
ENAH	1	0.15	0.14	2.80E-01
ENG	8	-0.12	0.03	6.73E-06
ENO1	4	0.17	0.11	1.18E-01
ENO2	5	-0.07	0.08	3.61E-01
ENPP2	8	0.01	0.05	8.86E-01
ENPP5	7	-0.03	0.03	2.31E-01
ENPP7	6	0.01	0.02	8.11E-01
ENTPD2	2	0.10	0.24	6.77E-01
ENTPD5	9	0.01	0.03	7.84E-01
ENTPD6	7	0.02	0.02	3.79E-01
EPCAM	14	0.13	0.04	1.94E-04
EPHA1	7	-0.12	0.06	3.70E-02
EPHA2	6	0.25	0.09	4.87E-03
EPHB4	10	-0.16	0.04	1.70E-05
EPHB6	4	0.09	0.06	1.43E-01
EPO	9	0.06	0.06	2.89E-01
EPS8L2	3	0.00	0.05	9.77E-01
ERBB2	9	0.03	0.06	5.97E-01
ERBB3	9	0.09	0.05	6.89E-02
ERBB4	16	-0.27	0.05	1.04E-08
ERBIN	3	-0.13	0.16	3.92E-01
EREG	10	0.04	0.07	5.79E-01
ERP44	6	0.04	0.03	1.87E-01

ESAM	8	0.11	0.06	8.48E-02
ESM1	11	-0.12	0.06	4.74E-02
EZR	2	-0.07	0.17	6.63E-01
F11R	11	0.12	0.07	1.08E-01
F2R	7	-0.08	0.07	2.23E-01
F3	9	0.04	0.03	2.65E-01
F7	7	0.05	0.03	4.95E-02
F9	2	0.24	0.12	4.87E-02
FABP1	2	0.01	0.06	8.53E-01
FABP2	5	0.12	0.08	1.44E-01
FABP4	2	0.17	0.14	2.09E-01
FABP5	2	0.22	0.18	2.36E-01
FABP6	4	0.06	0.06	2.97E-01
FABP9	16	0.06	0.03	4.53E-02
FADD	3	-0.10	0.14	4.80E-01
FAM3B	6	-0.07	0.02	4.15E-03
FAM3C	2	0.02	0.17	8.89E-01
FAP	6	-0.08	0.05	1.32E-01
FAS	4	0.09	0.05	1.10E-01
FASLG	21	0.03	0.03	3.54E-01
FBP1	5	0.02	0.06	7.83E-01
FCAR	14	-0.06	0.02	1.10E-03
FCER2	14	-0.04	0.04	3.65E-01
FCGR2A	5	0.00	0.01	9.13E-01
FCGR2B	4	-0.10	0.03	3.54E-04
FCGR3B	10	-0.04	0.05	3.59E-01
FCN2	11	-0.04	0.02	2.56E-02
FCRL1	26	-0.05	0.03	7.80E-02
FCRL2	16	0.04	0.02	1.12E-01
FCRL3	5	-0.02	0.01	9.56E-02
FCRL5	9	0.09	0.07	1.82E-01
FCRL6	5	-0.02	0.02	3.46E-01
FCRLB	7	0.03	0.03	3.35E-01
FEN1	1	-0.13	0.28	6.36E-01
FES	1	0.06	0.10	5.79E-01
FETUB	4	-0.02	0.05	5.90E-01
FGF19	5	0.30	0.05	5.26E-09
FGF2	6	0.00	0.02	9.15E-01
FGF21	7	0.07	0.06	3.08E-01
FGF23	5	-0.10	0.08	2.04E-01
FGF5	2	-0.02	0.03	5.60E-01
FGFBP1	13	-0.07	0.06	2.08E-01
FGFR2	8	0.22	0.09	1.40E-02
FGR	1	-0.12	0.17	4.75E-01
FHIT	2	-0.03	0.14	8.16E-01
FIS1	4	-0.11	0.08	1.33E-01
FKBP1B	4	0.02	0.06	6.83E-01
FKBP4	5	-0.04	0.10	7.13E-01
FKBP5	5	-0.05	0.06	4.83E-01
FLI1	3	-0.19	0.14	1.59E-01
FLRT2	11	0.02	0.03	5.16E-01
FLT1	8	-0.08	0.05	9.16E-02

FLT3	5	-0.16	0.10	1.18E-01
FLT3LG	20	0.01	0.04	7.07E-01
FLT4	11	-0.03	0.02	1.23E-01
FOLR1	8	-0.13	0.07	7.12E-02
FOLR2	14	-0.02	0.04	6.10E-01
FOLR3	10	-0.10	0.07	1.86E-01
FOXO1	4	0.02	0.09	8.35E-01
FRZB	4	-0.02	0.03	5.73E-01
FST	3	0.09	0.08	2.46E-01
FSTL3	2	0.01	0.15	9.27E-01
FUCA1	5	-0.01	0.01	3.44E-01
FURIN	3	0.00	0.07	9.83E-01
FUS	2	0.02	0.15	8.97E-01
FUT3_FUT5	14	-0.01	0.02	6.81E-01
FUT8	12	-0.01	0.02	7.73E-01
FXN	3	-0.17	0.15	2.65E-01
FXYD5	2	-0.08	0.15	5.84E-01
FYB1	2	0.35	0.16	3.05E-02
GAL	21	0.00	0.04	9.78E-01
GALNT10	9	0.01	0.05	7.73E-01
GALNT2	7	0.03	0.05	4.78E-01
GALNT3	7	0.02	0.03	5.80E-01
GALNT7	6	-0.05	0.04	2.54E-01
GAS6	15	0.00	0.03	9.67E-01
GBP2	1	-0.03	0.14	8.17E-01
GBP4	1	0.05	0.02	3.20E-02
GCG	5	0.03	0.12	8.02E-01
GCNT1	7	0.02	0.03	4.38E-01
GDF15	1	0.07	0.05	1.69E-01
GDF2	14	0.02	0.05	7.64E-01
GDNF	1	0.01	0.06	9.04E-01
GFAP	7	-0.10	0.10	3.22E-01
GFER	1	0.01	0.22	9.52E-01
GFRA1	3	0.09	0.05	9.16E-02
GFRA2	7	0.06	0.04	1.57E-01
GFRA3	21	0.01	0.04	7.85E-01
GGH	9	-0.02	0.03	5.06E-01
GGT1	7	-0.08	0.06	2.18E-01
GGT5	4	0.07	0.05	2.02E-01
GHRL	10	-0.01	0.06	9.31E-01
GKN1	4	0.26	0.12	2.36E-02
GLB1	9	0.04	0.04	2.86E-01
GLO1	1	-0.04	0.06	4.81E-01
GLOD4	2	0.08	0.19	6.88E-01
GLRX	2	-0.04	0.03	2.20E-01
GLT8D2	1	-0.07	0.09	4.11E-01
GMPR	2	-0.01	0.06	8.01E-01
GNE	3	-0.05	0.10	6.28E-01
GNLY	12	0.01	0.02	7.47E-01
GOLM2	9	0.19	0.04	4.95E-06
GOPC	2	0.08	0.15	6.02E-01
GP1BA	16	0.06	0.05	2.20E-01

GP2	8	0.10	0.02	1.10E-05
GP6	6	0.09	0.04	5.00E-02
GPA33	13	0.09	0.03	5.01E-03
GPC1	17	-0.11	0.04	4.61E-03
GPC5	10	-0.01	0.02	5.08E-01
GPNMB	6	0.03	0.04	4.05E-01
GPR37	10	0.00	0.02	8.07E-01
GRAP2	3	-0.10	0.14	4.91E-01
GRK5	1	0.04	0.06	4.50E-01
GRN	12	-0.04	0.02	4.48E-02
GRPEL1	3	-0.09	0.12	4.70E-01
GSAP	1	0.20	0.22	3.67E-01
GSTA1	9	-0.05	0.05	3.13E-01
GSTA3	5	-0.09	0.06	1.24E-01
GSTP1	1	0.08	0.09	3.73E-01
GUCA2A	7	-0.06	0.08	4.31E-01
GUSB	12	0.03	0.04	5.32E-01
GYS1	3	-0.01	0.06	8.99E-01
GZMA	14	0.01	0.06	8.41E-01
GZMB	10	-0.05	0.05	2.65E-01
GZMH	8	-0.06	0.06	3.16E-01
HAGH	1	-0.02	0.23	9.20E-01
HAO1	8	-0.03	0.08	7.59E-01
HARS1	2	0.02	0.16	9.18E-01
HAVCR1	7	0.06	0.03	2.15E-02
HAVCR2	8	-0.04	0.06	4.74E-01
HBEGF	23	0.02	0.04	6.31E-01
HBQ1	2	-0.03	0.04	5.00E-01
HCLS1	4	-0.05	0.10	6.45E-01
HDGF	2	-0.04	0.01	8.41E-04
HEBP1	1	-0.08	0.12	4.96E-01
HEXIM1	4	0.05	0.09	5.53E-01
HGF	5	-0.13	0.08	8.95E-02
HGS	4	0.01	0.13	9.12E-01
HLA-DRA	2	-0.13	0.16	4.20E-01
HLA-E	4	0.10	0.05	2.95E-02
HMBS	1	0.01	0.12	8.98E-01
HMOX1	18	0.00	0.04	9.23E-01
HMOX2	4	0.00	0.09	9.66E-01
HNMT	4	0.02	0.02	2.81E-01
HNRNPK	1	-0.11	0.24	6.57E-01
HPGDS	2	0.02	0.03	5.78E-01
HS3ST3B1	10	0.01	0.04	8.73E-01
HS6ST1	15	-0.05	0.05	2.59E-01
HSD11B1	12	0.01	0.04	9.06E-01
HSPA1A	3	-0.10	0.14	4.47E-01
HSPB1	5	-0.12	0.10	2.34E-01
HSPB6	4	-0.23	0.15	1.16E-01
HSPG2	4	0.02	0.08	8.10E-01
HTRA2	1	-0.12	0.28	6.57E-01
HYAL1	3	-0.01	0.05	7.94E-01
HYOU1	13	0.09	0.05	7.75E-02

ICA1	2	-0.02	0.07	7.36E-01
ICAM1	6	-0.19	0.03	3.42E-09
ICAM2	10	-0.05	0.01	3.25E-04
ICAM3	21	-0.11	0.03	2.47E-04
ICAM4	14	0.11	0.05	1.83E-02
ICAM5	6	-0.12	0.02	2.14E-11
ICOSLG	3	0.01	0.14	9.40E-01
IDI2	1	0.00	0.04	9.38E-01
IDS	3	-0.12	0.06	6.74E-02
IDUA	13	0.00	0.02	8.75E-01
IFNG	2	-0.65	0.22	2.36E-03
IFNGR1	10	-0.01	0.05	9.02E-01
IFNGR2	9	0.00	0.01	9.13E-01
IFNL1	5	-0.06	0.10	5.69E-01
IFNLR1	3	-0.02	0.05	7.20E-01
IGF1R	9	-0.21	0.03	2.42E-10
IGF2R	14	-0.01	0.03	7.32E-01
IGFBP1	2	0.04	0.14	7.68E-01
IGFBP2	4	-0.16	0.12	1.90E-01
IGFBP3	5	0.00	0.03	8.81E-01
IGFBP4	3	-0.02	0.17	9.18E-01
IGFBP6	3	-0.01	0.16	9.53E-01
IGFBP7	3	0.02	0.05	6.46E-01
IGFBPL1	2	0.01	0.04	7.08E-01
IGSF3	5	-0.09	0.09	3.44E-01
IGSF8	5	0.09	0.08	2.34E-01
IKBKG	1	0.27	0.29	3.51E-01
IKZF2	2	0.03	0.14	8.45E-01
IL10	5	-0.01	0.06	8.62E-01
IL10RA	1	0.03	0.10	7.77E-01
IL10RB	6	0.04	0.04	3.21E-01
IL11	1	-0.27	0.54	6.16E-01
IL12A_IL12B	17	-0.03	0.02	1.42E-01
IL12B	20	-0.03	0.02	1.56E-01
IL12RB1	5	-0.02	0.03	5.54E-01
IL13	1	-0.26	0.21	2.25E-01
IL13RA1	2	0.03	0.16	8.26E-01
IL15	21	0.07	0.04	1.12E-01
IL15RA	1	0.02	0.06	7.71E-01
IL16	1	-0.05	0.22	8.21E-01
IL17C	3	-0.07	0.11	5.60E-01
IL17D	3	-0.09	0.04	2.55E-02
IL17F	1	0.10	0.28	7.18E-01
IL17RA	3	0.00	0.03	8.80E-01
IL17RB	10	-0.04	0.02	4.67E-02
IL18	9	-0.06	0.04	7.33E-02
IL18BP	9	-0.12	0.07	7.20E-02
IL18R1	12	-0.02	0.02	3.16E-01
IL18RAP	3	0.12	0.10	2.48E-01
IL19	4	-0.14	0.03	1.54E-06
IL1A	1	0.02	0.14	8.69E-01
IL1B	2	-0.04	0.06	5.01E-01

IL1R1	13	-0.01	0.03	7.68E-01
IL1R2	13	0.04	0.03	1.34E-01
IL1RAP	4	-0.04	0.02	9.89E-03
IL1RL1	13	-0.02	0.02	3.91E-01
IL1RL2	12	0.01	0.03	6.52E-01
IL1RN	2	-0.02	0.07	8.23E-01
IL20RA	1	0.02	0.28	9.49E-01
IL22RA1	2	0.16	0.05	9.98E-04
IL2RA	8	0.01	0.03	7.20E-01
IL32	7	0.02	0.06	7.78E-01
IL33	1	0.32	0.56	5.65E-01
IL34	1	-0.01	0.03	7.24E-01
IL3RA	4	-0.12	0.12	3.31E-01
IL4R	5	-0.06	0.04	1.19E-01
IL5RA	23	0.04	0.03	1.71E-01
IL6	3	-0.18	0.08	2.26E-02
IL6R	5	-0.03	0.01	1.03E-02
IL6ST	10	-0.07	0.04	9.86E-02
IL7	3	-0.15	0.11	1.99E-01
IL7R	12	-0.06	0.02	5.01E-04
ILKAP	1	-0.11	0.25	6.57E-01
IMPA1	2	-0.07	0.07	3.26E-01
ING1	1	-0.12	0.27	6.57E-01
INHBC	6	0.03	0.04	3.98E-01
INPP1	3	-0.20	0.14	1.40E-01
IPCEF1	2	0.10	0.14	4.98E-01
IQGAP2	1	-0.01	0.13	9.65E-01
IRAG2	2	-0.36	0.17	3.92E-02
IRAK1	1	0.23	0.25	3.51E-01
ISLR2	8	-0.14	0.02	1.50E-08
ISM1	2	-0.06	0.08	4.55E-01
ITGA11	5	0.04	0.06	4.51E-01
ITGA5	10	-0.05	0.06	4.38E-01
ITGA6	7	-0.11	0.05	1.67E-02
ITGAM	9	-0.09	0.06	1.14E-01
ITGAV	5	-0.09	0.08	2.81E-01
ITGB1	7	-0.14	0.07	5.42E-02
ITGB1BP2	4	-0.01	0.11	9.40E-01
ITGB2	10	-0.14	0.05	1.14E-02
ITGB5	5	0.04	0.10	6.92E-01
ITGB6	9	-0.09	0.05	5.59E-02
ITGB7	18	-0.01	0.03	7.92E-01
ITIH3	7	-0.06	0.04	1.58E-01
ITM2A	2	-0.07	0.07	2.61E-01
JAM2	6	-0.03	0.07	6.39E-01
JCHAIN	14	-0.01	0.07	8.94E-01
KAZALD1	6	-0.03	0.02	1.20E-01
KDR	11	-0.03	0.03	3.63E-01
KEL	16	-0.14	0.03	1.42E-07
KIFBP	3	0.07	0.10	4.52E-01
KIR2DL3	4	0.04	0.03	1.45E-01
KIR3DL1	1	0.71	0.41	8.38E-02

KIRREL2	8	0.00	0.03	9.73E-01
KIT	22	-0.02	0.04	6.64E-01
KITLG	15	-0.03	0.04	4.60E-01
KLB	9	0.00	0.02	8.98E-01
KLK1	13	-0.09	0.02	8.62E-09
KLK10	3	0.02	0.03	4.36E-01
KLK11	5	-0.05	0.04	2.22E-01
KLK12	5	-0.04	0.02	3.24E-02
KLK13	10	0.00	0.03	9.64E-01
KLK14	9	0.01	0.04	7.19E-01
KLK4	3	-0.01	0.05	7.80E-01
KLK6	2	-0.03	0.08	7.15E-01
KLK8	4	0.06	0.04	7.49E-02
KLRB1	10	-0.14	0.06	1.91E-02
KLRD1	12	0.01	0.03	6.30E-01
KRT18	9	-0.13	0.07	7.17E-02
KRT19	3	0.24	0.09	1.09E-02
KRT5	4	0.04	0.08	6.15E-01
KYAT1	4	0.12	0.13	3.50E-01
KYNU	4	0.03	0.05	5.97E-01
L1CAM	6	-0.06	0.07	4.05E-01
LACTB2	3	0.20	0.10	5.17E-02
LAG3	12	-0.12	0.05	7.98E-03
LAIR1	4	0.07	0.05	1.45E-01
LAIR2	5	0.00	0.02	8.67E-01
LAMA4	11	0.00	0.04	9.10E-01
LAMP2	2	0.30	0.22	1.61E-01
LAMP3	10	-0.41	0.06	1.88E-10
LAT	6	0.04	0.09	6.50E-01
LAT2	1	0.21	0.23	3.51E-01
LAYN	4	-0.04	0.04	2.39E-01
LBP	2	-0.02	0.02	5.04E-01
LBR	6	-0.06	0.07	3.58E-01
LCN2	17	0.05	0.06	4.14E-01
LDLR	16	0.02	0.04	6.19E-01
LEFTY2	4	-0.63	0.14	8.97E-06
LEP	1	0.93	0.32	3.35E-03
LEPR	9	0.03	0.05	4.73E-01
LGALS1	2	-0.02	0.07	8.02E-01
LGALS3	5	-0.07	0.04	8.46E-02
LGALS4	3	-0.19	0.03	3.53E-11
ALS7_LGALS	7	0.14	0.04	7.56E-05
LGALS8	6	-0.16	0.03	5.75E-08
LGALS9	4	-0.06	0.04	2.05E-01
LGMN	18	0.02	0.04	6.88E-01
LHB	2	0.30	0.18	9.27E-02
LIFR	11	-0.17	0.03	1.10E-06
LILRA2	10	-0.14	0.07	6.08E-02
LILRA5	9	-0.07	0.06	2.15E-01
LILRB1	9	0.04	0.04	2.59E-01
LILRB2	10	-0.02	0.02	3.73E-01
LILRB4	4	-0.03	0.09	7.02E-01

LILRB5	3	0.00	0.01	9.64E-01
LPCAT2	1	-0.29	0.12	1.67E-02
LPL	8	-0.07	0.04	4.63E-02
LPO	12	-0.03	0.03	3.33E-01
LRIG1	10	-0.01	0.02	6.45E-01
LRP1	11	-0.01	0.02	6.31E-01
LRP11	6	0.04	0.02	6.67E-02
LRPAP1	7	-0.01	0.02	7.24E-01
LRRC25	13	-0.02	0.03	4.55E-01
LRRN1	7	0.01	0.02	6.15E-01
LSP1	2	-0.13	0.10	1.73E-01
LTA	10	0.00	0.02	8.44E-01
LTA4H	1	-0.17	0.15	2.63E-01
LTBP2	7	-0.25	0.08	2.50E-03
LTBP3	6	-0.01	0.03	5.97E-01
LTBR	13	0.00	0.04	9.84E-01
LXN	2	-0.09	0.08	2.59E-01
LY6D	5	-0.03	0.05	5.80E-01
LY75	6	-0.01	0.01	6.64E-01
LY9	14	-0.01	0.03	7.91E-01
LY96	10	-0.07	0.08	4.03E-01
LYAR	1	-0.01	0.29	9.69E-01
LYN	5	-0.03	0.12	7.98E-01
LYPD3	17	-0.10	0.03	1.31E-04
LYPD8	7	-0.01	0.04	7.59E-01
MAD1L1	1	-0.14	0.14	3.02E-01
MAGED1	2	-0.02	0.14	8.70E-01
MANF	6	0.05	0.11	6.57E-01
MANSC1	17	-0.11	0.06	5.23E-02
MAP2K6	2	-0.46	0.16	4.75E-03
MAP3K5	1	0.24	0.26	3.51E-01
MAP4K5	3	0.06	0.08	4.34E-01
MAPK9	1	-0.22	0.12	6.58E-02
MARCO	9	-0.10	0.04	1.88E-02
MASP1	13	0.00	0.03	9.44E-01
MATN2	5	0.00	0.03	9.94E-01
MATN3	4	-0.02	0.03	5.43E-01
MAVS	3	-0.05	0.12	6.49E-01
MAX	5	-0.07	0.10	5.03E-01
MB	8	0.00	0.10	9.85E-01
MCAM	8	0.06	0.05	2.36E-01
MCFD2	4	-0.12	0.08	1.70E-01
MDGA1	6	-0.01	0.01	5.46E-01
MDK	11	0.02	0.07	7.31E-01
MED18	1	-0.76	0.24	1.72E-03
MEGF10	13	-0.03	0.03	3.71E-01
MEGF9	11	0.10	0.05	3.04E-02
MEP1B	15	0.02	0.01	1.25E-01
MEPE	14	-0.12	0.05	3.34E-02
MERTK	13	-0.03	0.05	4.94E-01
MESD	4	0.09	0.11	4.28E-01
MET	20	-0.18	0.04	5.06E-07

METAP1D	2	-0.06	0.18	7.33E-01
METAP2	4	-0.04	0.10	6.51E-01
MFAP5	5	-0.34	0.12	4.19E-03
MFGE8	11	0.00	0.03	9.19E-01
MGLL	3	-0.11	0.12	3.71E-01
MGMT	3	0.01	0.04	6.93E-01
MIA	7	-0.05	0.03	4.49E-02
MICB_MICA	8	-0.03	0.01	2.07E-02
MIF	3	-0.04	0.12	7.34E-01
MILR1	9	-0.02	0.05	6.00E-01
MITD1	2	0.21	0.16	1.87E-01
MLN	17	0.03	0.03	3.09E-01
MME	13	0.01	0.03	7.50E-01
MMP1	12	0.02	0.03	5.28E-01
MMP10	2	-0.17	0.05	2.68E-04
MMP12	2	-0.02	0.03	4.98E-01
MMP13	1	-0.12	0.14	3.67E-01
MMP3	4	0.03	0.04	4.65E-01
MMP7	5	-0.01	0.06	8.77E-01
MMP8	2	-0.03	0.04	4.63E-01
MMP9	9	-0.14	0.06	2.57E-02
MNDA	1	-1.37	0.29	2.72E-06
MOG	2	0.16	0.07	1.80E-02
MPHOSPH8	2	0.08	0.15	6.09E-01
MPI	4	0.06	0.06	3.35E-01
MPIG6B	5	0.08	0.09	3.96E-01
MPO	6	-0.15	0.06	7.35E-03
MSLN	8	-0.07	0.03	3.81E-02
MSMB	5	-0.03	0.02	1.72E-01
MSR1	12	-0.02	0.04	5.71E-01
MSRA	2	0.01	0.17	9.52E-01
MSTN	5	0.08	0.11	4.53E-01
MUC13	4	-0.19	0.05	3.08E-05
MUC16	3	-0.07	0.08	3.41E-01
MYO9B	1	0.64	0.48	1.82E-01
MYOC	9	0.02	0.09	8.70E-01
MZB1	7	0.05	0.07	5.25E-01
MZT1	2	0.01	0.18	9.53E-01
NAAA	15	0.00	0.02	9.30E-01
NADK	5	-0.01	0.05	8.86E-01
NBL1	3	-0.07	0.11	5.26E-01
NBN	3	0.18	0.15	2.26E-01
NCAM1	7	0.01	0.05	7.73E-01
NCAM2	5	0.11	0.06	7.78E-02
NCAN	11	-0.02	0.06	7.93E-01
NCF2	4	-0.12	0.08	1.43E-01
NCK2	1	0.54	0.43	2.13E-01
NCR1	22	-0.14	0.04	1.27E-04
NCS1	8	-0.07	0.09	3.97E-01
NECTIN2	4	0.16	0.06	6.39E-03
NECTIN4	6	-0.01	0.06	8.23E-01
NEFL	2	-0.22	0.23	3.24E-01

NELL1	18	0.04	0.03	1.65E-01
NELL2	14	-0.22	0.06	1.31E-04
NFASC	6	0.00	0.04	9.49E-01
NFATC1	2	-0.11	0.12	3.88E-01
NFATC3	1	0.11	0.26	6.81E-01
NFKBIE	2	0.09	0.09	3.23E-01
NID1	10	0.00	0.05	9.99E-01
NID2	15	0.07	0.03	9.79E-03
NME3	12	-0.10	0.04	1.96E-02
NMNAT1	5	0.00	0.06	9.54E-01
NOMO1	3	-0.05	0.11	6.59E-01
NOS1	2	-0.08	0.14	5.74E-01
NOS3	2	-0.33	0.17	4.95E-02
NOTCH1	6	-0.04	0.05	4.45E-01
NOTCH3	10	-0.22	0.08	6.17E-03
NPDC1	5	-0.15	0.13	2.32E-01
NPPB	2	-0.07	0.08	3.37E-01
NPPC	2	0.14	0.21	5.03E-01
NPTX1	13	-0.04	0.03	2.17E-01
NPTXR	4	-0.01	0.02	5.61E-01
NPY	12	-0.01	0.05	8.84E-01
NRCAM	6	-0.39	0.09	1.19E-05
NRP1	6	-0.07	0.04	1.20E-01
NRP2	9	0.13	0.06	3.71E-02
NRTN	1	0.15	0.28	6.02E-01
NSFL1C	2	0.30	0.14	3.68E-02
NT5C3A	4	0.17	0.10	1.03E-01
NT5E	10	-0.03	0.02	1.65E-01
NTF3	4	-0.03	0.07	6.71E-01
NTF4	1	-0.03	0.27	9.14E-01
NTproBNP	4	-0.03	0.05	5.35E-01
NTRK2	7	0.15	0.10	1.61E-01
NTRK3	10	-0.03	0.05	6.36E-01
NUB1	4	-0.14	0.07	5.23E-02
NUCB2	5	0.03	0.04	3.44E-01
NUDC	2	0.02	0.14	9.05E-01
NUDT2	2	0.08	0.06	1.66E-01
NUDT5	2	0.04	0.17	8.15E-01
OBP2B	12	0.00	0.03	8.59E-01
ODAM	2	-0.06	0.06	3.40E-01
OGFR	1	-0.12	0.28	6.57E-01
OGN	4	0.02	0.02	3.91E-01
OLR1	6	0.04	0.08	6.14E-01
OMD	6	0.04	0.07	6.00E-01
OMG	7	0.03	0.09	7.30E-01
OPTC	7	0.01	0.08	9.15E-01
OSCAR	9	0.00	0.03	9.91E-01
OSM	8	-0.21	0.06	8.46E-04
OSMR	5	-0.01	0.03	7.50E-01
OXT	9	0.08	0.04	2.52E-02
P4HB	4	0.10	0.13	4.52E-01
PADI2	1	-0.05	0.12	6.63E-01

PADI4	1	-0.05	0.05	3.87E-01
PAEP	3	-0.01	0.02	6.14E-01
PAG1	6	-0.08	0.09	3.31E-01
PAK4	1	0.26	0.28	3.51E-01
PAM	4	0.04	0.03	1.71E-01
PAMR1	4	0.03	0.04	4.81E-01
PAPPA	11	0.03	0.05	4.86E-01
PARK7	5	0.03	0.10	7.87E-01
PARP1	2	-0.06	0.09	5.12E-01
PBLD	1	0.02	0.03	4.92E-01
PCDH1	6	-0.04	0.09	6.46E-01
PCDH17	8	0.00	0.03	9.97E-01
PCOLCE	1	0.35	0.13	4.86E-03
PCSK9	9	0.03	0.05	6.17E-01
PDCD1	12	0.00	0.07	9.70E-01
PDCD1LG2	8	-0.03	0.02	1.32E-01
PDCD5	2	-0.10	0.06	7.74E-02
PDCD6	4	-0.03	0.02	6.77E-02
PDGFA	17	0.04	0.04	3.71E-01
PDGFB	17	0.04	0.04	3.34E-01
PDGFC	7	0.01	0.05	8.73E-01
PDGFRA	9	0.02	0.04	6.82E-01
PDGFRB	2	-0.02	0.01	1.56E-01
PDLIM7	3	0.00	0.13	9.91E-01
PEAR1	9	0.00	0.04	9.79E-01
PEBP1	2	-0.17	0.18	3.65E-01
PECAM1	8	-0.17	0.03	2.20E-09
PFKFB2	4	-0.06	0.07	3.49E-01
PGF	3	-0.03	0.13	8.28E-01
PGLYRP1	12	0.01	0.04	7.97E-01
PI3	4	0.02	0.05	6.99E-01
PIGR	6	0.12	0.07	6.32E-02
PIK3AP1	7	0.00	0.06	9.87E-01
PIK3IP1	3	-0.13	0.05	6.02E-03
PILRA	6	-0.01	0.02	6.38E-01
PILRB	4	-0.01	0.01	6.65E-01
PKLR	4	-0.29	0.07	8.11E-05
PLA2G10	15	0.04	0.03	2.37E-01
PLA2G15	9	0.00	0.04	9.38E-01
PLA2G1B	18	-0.09	0.05	6.47E-02
PLA2G2A	6	-0.07	0.02	3.51E-03
PLA2G4A	3	-0.06	0.15	6.71E-01
PLA2G7	9	-0.07	0.05	1.50E-01
PLAT	4	-0.36	0.11	1.49E-03
PLAU	10	-0.06	0.05	2.12E-01
PLAUR	11	0.02	0.06	6.89E-01
PLIN1	1	0.57	0.32	7.04E-02
PLIN3	2	0.24	0.17	1.44E-01
PLPBP	2	0.07	0.16	6.70E-01
PLTP	11	0.00	0.03	8.77E-01
PLXDC1	5	-0.03	0.06	5.80E-01
PLXNA4	5	0.09	0.08	2.86E-01

PLXNB2	11	-0.01	0.02	6.37E-01
PLXNB3	5	0.38	0.12	1.85E-03
PM20D1	8	-0.02	0.03	5.66E-01
PMVK	6	-0.11	0.10	2.77E-01
PNLIPRP2	7	-0.04	0.01	4.44E-04
PNPT1	2	0.29	0.18	1.06E-01
PODXL	6	-0.14	0.03	6.56E-08
PODXL2	12	-0.02	0.06	8.00E-01
POLR2F	1	-0.49	0.27	6.81E-02
PON2	3	-0.04	0.03	1.61E-01
PON3	9	0.07	0.04	5.49E-02
PPCDC	2	0.06	0.04	1.45E-01
PPIB	3	0.13	0.14	3.64E-01
PPME1	1	0.06	0.27	8.33E-01
PPP1R12A	4	0.17	0.12	1.44E-01
PPP1R2	3	0.15	0.14	2.82E-01
PPP1R9B	2	0.03	0.17	8.67E-01
PPP3R1	5	-0.17	0.11	1.18E-01
PPY	8	-0.07	0.07	3.03E-01
PRCP	12	0.06	0.05	2.17E-01
PRDX1	2	-0.07	0.19	7.05E-01
PRDX3	1	-0.31	0.23	1.71E-01
PRDX5	2	-0.22	0.16	1.54E-01
PRDX6	1	0.00	0.11	9.65E-01
PRELP	1	0.00	0.03	9.96E-01
PRKAB1	2	0.07	0.23	7.69E-01
PRKAR1A	5	0.02	0.08	8.37E-01
PRKRA	1	0.13	0.14	3.51E-01
PRL	3	0.22	0.13	1.03E-01
PROC	8	0.02	0.05	6.78E-01
PROK1	2	0.08	0.04	4.54E-02
PRSS2	13	-0.06	0.05	2.60E-01
PRSS27	18	-0.11	0.02	1.06E-07
PRSS8	8	0.08	0.08	3.03E-01
PRTFDC1	3	0.05	0.10	6.55E-01
PRTG	9	0.04	0.03	2.21E-01
PRTN3	14	-0.05	0.04	2.27E-01
PSG1	6	-0.07	0.04	5.42E-02
PSIP1	3	0.00	0.10	9.78E-01
PSME1	3	-0.10	0.14	4.63E-01
PSME2	1	-0.10	0.17	5.46E-01
PSMG3	1	-0.10	0.42	8.05E-01
PSPN	9	0.09	0.10	3.91E-01
PSRC1	2	0.01	0.09	9.17E-01
PTGDS	2	0.21	0.20	2.79E-01
PTN	4	0.08	0.08	3.19E-01
PTPN1	3	-0.14	0.12	2.39E-01
PTPN6	2	-0.03	0.15	8.59E-01
PTPRF	14	-0.01	0.05	8.35E-01
PTPRM	8	-0.14	0.02	5.67E-09
PTPRN2	1	0.00	0.09	9.79E-01
PTPRS	5	0.00	0.05	9.87E-01

PTS	4	0.00	0.09	9.78E-01
PTX3	4	0.03	0.04	4.34E-01
PVALB	8	0.00	0.02	9.31E-01
PVR	2	-0.01	0.02	5.60E-01
PXN	3	-0.01	0.02	5.84E-01
QDPR	1	0.22	0.23	3.51E-01
QPCT	11	-0.02	0.04	6.32E-01
RAB6A	3	-0.02	0.04	5.37E-01
RABEPK	1	-0.13	0.10	1.85E-01
RABGAP1L	2	0.18	0.18	3.28E-01
RAD23B	3	0.18	0.15	2.04E-01
RARRES1	1	0.08	0.03	8.00E-03
RARRES2	3	0.05	0.09	5.82E-01
RASSF2	1	-0.10	0.24	6.69E-01
RBKS	1	-0.06	0.07	3.91E-01
RBP2	4	0.12	0.11	2.88E-01
RBP5	3	0.06	0.05	1.94E-01
RCOR1	1	0.34	0.24	1.58E-01
REG1A	5	0.29	0.04	1.47E-14
REG1B	7	0.23	0.03	8.86E-12
REG3A	8	0.06	0.06	3.19E-01
REG4	1	0.15	0.09	9.89E-02
RELT	4	0.06	0.04	1.56E-01
REN	2	-0.19	0.12	1.03E-01
RET	7	0.09	0.04	1.34E-02
RETN	15	0.03	0.05	4.75E-01
RGMA	17	0.02	0.04	6.17E-01
RGMB	4	-0.10	0.07	1.75E-01
RGS8	1	0.02	0.10	8.54E-01
RHOC	3	-0.05	0.14	7.18E-01
RILP	4	0.05	0.11	6.53E-01
RNASE3	4	0.04	0.05	4.36E-01
RNASET2	9	0.10	0.04	1.41E-02
ROBO1	9	-0.01	0.06	9.15E-01
ROBO2	5	0.02	0.10	8.12E-01
ROR1	12	0.00	0.04	9.10E-01
RP2	2	-0.02	0.17	9.04E-01
RRM2	1	0.09	0.25	7.20E-01
RRM2B	2	0.21	0.13	9.69E-02
RSPO1	10	0.01	0.05	8.49E-01
RSPO3	8	-0.04	0.06	4.89E-01
RTBDN	3	-0.01	0.09	8.99E-01
RTN4R	10	-0.11	0.05	1.89E-02
RWDD1	2	0.12	0.10	2.43E-01
S100A11	2	0.01	0.07	9.19E-01
S100A12	3	-0.02	0.06	7.07E-01
S100A16	3	0.28	0.11	1.10E-02
S100A4	5	0.03	0.10	7.80E-01
S100P	5	-0.24	0.10	1.33E-02
SAMD9L	2	-0.01	0.05	8.19E-01
SCAMP3	4	-0.19	0.10	6.95E-02
SCARA5	7	0.15	0.06	1.62E-02

SCARB1	1	-0.40	0.33	2.24E-01
SCARB2	4	0.07	0.06	2.07E-01
SCARF1	7	0.01	0.03	8.36E-01
SCARF2	8	-0.14	0.04	1.32E-03
SCG2	4	-0.82	0.12	3.08E-12
SCG3	5	-0.03	0.02	2.08E-01
SCGB1A1	17	0.06	0.04	2.04E-01
SCGB3A2	17	0.05	0.04	2.35E-01
SCGN	5	0.35	0.07	4.55E-07
SCLY	7	0.06	0.08	4.19E-01
SCP2	1	0.49	0.29	9.58E-02
SCRN1	2	0.10	0.11	3.69E-01
SDC1	13	0.04	0.05	4.35E-01
SDC4	20	0.04	0.04	2.60E-01
SELE	9	-0.10	0.02	1.92E-08
SELP	10	-0.09	0.04	2.71E-02
SELPLG	10	0.02	0.03	4.56E-01
SEMA3F	5	-0.39	0.10	1.37E-04
SEMA4C	4	-0.41	0.06	7.00E-11
SEMA4D	6	-0.02	0.08	7.92E-01
SEMA7A	18	-0.04	0.04	4.02E-01
SEPTIN9	2	0.35	0.18	5.47E-02
SERPINA11	8	-0.01	0.02	5.87E-01
SERPINA12	5	-0.01	0.02	6.90E-01
SERPINA9	3	0.02	0.03	5.42E-01
SERPINB1	3	0.03	0.12	8.30E-01
SERPINB6	2	0.07	0.11	5.63E-01
SERPINB8	2	-0.02	0.02	4.73E-01
SERPINB9	2	-0.11	0.11	3.14E-01
SERPINE1	17	0.03	0.04	5.11E-01
SESTD1	3	-0.01	0.11	9.40E-01
SETMAR	1	0.05	0.09	5.40E-01
SEZ6L	6	0.03	0.07	6.29E-01
SEZ6L2	8	0.05	0.07	4.04E-01
SF3B4	4	-0.05	0.12	6.58E-01
SFRP1	5	0.04	0.08	5.82E-01
SFTPA1	1	-0.31	0.09	3.05E-04
SFTPA2	6	0.09	0.02	5.01E-05
SFTPD	11	-0.08	0.02	1.94E-06
SH2B3	4	0.04	0.12	7.16E-01
SH2D1A	2	-0.06	0.12	6.04E-01
SHMT1	4	-0.03	0.03	2.54E-01
SIAE	9	0.09	0.06	1.35E-01
SIGLEC1	10	-0.14	0.06	1.54E-02
SIGLEC10	8	0.00	0.03	8.96E-01
SIGLEC5	3	0.04	0.02	2.83E-02
SIGLEC6	20	0.03	0.03	4.18E-01
SIGLEC7	9	0.03	0.06	5.35E-01
SIGLEC9	10	-0.01	0.02	4.29E-01
SIRPA	7	0.02	0.02	2.55E-01
SIRPB1	11	-0.02	0.02	2.64E-01
SIRT2	3	0.06	0.11	5.97E-01

SIT1	6	-0.13	0.08	1.26E-01
SKAP1	8	0.00	0.09	9.73E-01
SKAP2	3	0.16	0.15	2.72E-01
SLAMF1	5	-0.03	0.09	7.49E-01
SLAMF6	3	-0.19	0.10	5.66E-02
SLAMF7	9	0.03	0.02	1.21E-01
SLAMF8	5	0.00	0.02	9.34E-01
SLC16A1	4	-0.10	0.09	2.29E-01
SLC27A4	3	0.00	0.11	9.69E-01
SLC39A14	2	-0.15	0.14	2.83E-01
SLC39A5	8	0.02	0.08	8.17E-01
SLIT2	1	0.50	0.33	1.30E-01
SLITRK2	10	0.22	0.07	1.12E-03
SLITRK6	8	0.00	0.04	9.50E-01
SMAD1	5	-0.02	0.11	8.86E-01
SMAD5	1	0.12	0.08	1.35E-01
SMOC1	10	-0.03	0.04	4.71E-01
SMOC2	5	0.02	0.04	5.62E-01
SMPD1	15	0.00	0.03	9.94E-01
SMPDL3A	20	0.02	0.02	2.98E-01
SNAP23	3	0.10	0.11	3.50E-01
SNAP29	5	0.16	0.10	1.12E-01
SNCG	1	-0.01	0.02	3.69E-01
SNX9	2	-0.26	0.14	5.57E-02
SOD1	3	0.03	0.12	7.96E-01
SOD2	7	-0.16	0.06	5.40E-03
SORCS2	4	0.01	0.04	7.98E-01
SORD	4	-0.10	0.10	2.98E-01
SORT1	14	0.02	0.04	6.17E-01
SOST	14	0.13	0.05	6.50E-03
SPARC	14	0.03	0.05	5.42E-01
SPARCL1	4	0.01	0.02	7.15E-01
SPINK1	2	0.00	0.05	9.72E-01
SPINK4	5	0.01	0.02	6.73E-01
SPINK5	7	-0.14	0.04	3.30E-04
SPINK6	1	-0.07	0.05	1.66E-01
SPINT1	8	-0.05	0.04	2.04E-01
SPINT2	15	0.05	0.05	3.05E-01
SPOCK1	12	-0.02	0.06	7.51E-01
SPON1	6	0.01	0.05	7.95E-01
SPON2	1	0.01	0.07	9.18E-01
SPP1	6	-0.15	0.11	1.73E-01
SPRY2	6	0.02	0.08	8.29E-01
SRC	1	0.64	0.27	1.72E-02
SRP14	2	0.01	0.12	9.09E-01
SRPK2	3	-0.07	0.11	5.13E-01
SSC4D	10	-0.01	0.04	7.41E-01
SSC5D	10	0.12	0.06	3.90E-02
ST3GAL1	7	0.04	0.05	4.06E-01
ST6GAL1	7	0.00	0.08	9.85E-01
STAMBP	2	-0.18	0.18	3.17E-01
STAT5B	3	-0.06	0.14	6.90E-01

STC1	5	-0.23	0.09	1.29E-02
STC2	5	-0.06	0.13	6.59E-01
STIP1	2	0.08	0.15	6.11E-01
STK11	4	-0.07	0.12	5.53E-01
STK24	2	0.07	0.19	7.25E-01
STK4	4	0.03	0.12	8.05E-01
STX16	1	-0.24	0.27	3.72E-01
STX4	2	-0.60	0.22	6.18E-03
STX6	2	-0.22	0.21	2.81E-01
STX8	3	0.09	0.13	4.84E-01
SUGT1	1	0.21	0.22	3.51E-01
SULT1A1	1	-0.15	0.16	3.39E-01
SULT2A1	4	-0.08	0.05	1.18E-01
SUMF2	13	-0.04	0.04	2.52E-01
SUSD1	4	0.00	0.09	9.71E-01
SUSD2	4	0.06	0.03	7.14E-02
TACC3	4	0.02	0.11	8.38E-01
TACSTD2	13	-0.05	0.02	5.60E-03
TAFA5	2	0.01	0.09	9.33E-01
TARBP2	3	-0.12	0.13	3.92E-01
TBC1D17	1	0.01	0.03	7.96E-01
TBC1D23	4	-0.07	0.08	4.03E-01
TBC1D5	2	-0.01	0.17	9.68E-01
TBCB	4	-0.16	0.12	1.97E-01
TBCC	4	-0.08	0.09	3.72E-01
TBL1X	5	-0.07	0.09	4.23E-01
TCL1A	21	-0.05	0.04	2.26E-01
TCN2	2	0.01	0.03	7.66E-01
TDGF1	1	-2.13	0.34	2.07E-10
TDRKH	4	-0.06	0.04	1.41E-01
TEK	9	0.03	0.03	3.08E-01
TFF1	5	-0.06	0.05	1.97E-01
TFF2	8	0.03	0.06	6.52E-01
TFF3	3	-0.06	0.05	1.83E-01
TFPI	6	0.00	0.04	9.47E-01
TFPI2	9	-0.07	0.06	2.69E-01
TFRC	11	-0.07	0.02	6.47E-03
TGFA	4	-0.27	0.09	2.43E-03
TGFB1	10	-0.04	0.05	4.34E-01
TGFBI	8	-0.02	0.02	4.74E-01
TGFBR2	8	-0.27	0.07	2.44E-04
TGFBR3	7	-0.03	0.06	6.18E-01
TGM2	2	0.00	0.08	9.79E-01
THBD	14	0.00	0.05	9.26E-01
THBS2	7	-0.06	0.03	7.84E-02
THBS4	6	0.12	0.05	1.90E-02
THOP1	1	0.01	0.09	9.53E-01
THPO	17	-0.04	0.05	3.54E-01
THY1	3	0.02	0.04	6.65E-01
TIA1	2	0.29	0.14	4.49E-02
TIE1	12	0.03	0.03	3.21E-01
TIGAR	2	-0.11	0.19	5.68E-01

TIMD4	24	-0.04	0.03	1.99E-01
TIMP1	5	0.21	0.08	9.33E-03
TIMP3	17	0.03	0.02	2.40E-01
TIMP4	4	-0.16	0.04	1.05E-04
TINAGL1	8	-0.03	0.08	6.51E-01
TJAP1	2	0.14	0.21	5.04E-01
TLR3	6	0.00	0.01	7.41E-01
TMPRSS15	7	0.03	0.05	4.95E-01
TMPRSS5	18	0.04	0.02	3.36E-02
TMSB10	6	-0.14	0.09	1.23E-01
TNC	15	0.02	0.03	4.77E-01
TNF	2	-0.09	0.08	2.92E-01
TNFAIP8	1	0.18	0.19	3.49E-01
TNFRSF10A	4	-0.05	0.03	1.01E-01
TNFRSF10B	3	0.04	0.05	3.57E-01
TNFRSF10C	16	0.00	0.02	8.10E-01
TNFRSF11A	6	0.08	0.04	2.95E-02
TNFRSF11B	9	-0.02	0.06	7.62E-01
TNFRSF12A	2	-0.32	0.18	7.75E-02
TNFRSF13B	10	-0.09	0.06	1.22E-01
TNFRSF13C	12	-0.01	0.08	8.98E-01
TNFRSF14	5	-0.06	0.09	5.14E-01
TNFRSF19	4	0.03	0.05	5.47E-01
TNFRSF1A	6	0.22	0.10	3.28E-02
TNFRSF1B	6	-0.17	0.07	1.63E-02
TNFRSF21	11	0.05	0.06	3.70E-01
TNFRSF4	6	0.01	0.08	9.43E-01
TNFRSF6B	3	-0.06	0.05	2.44E-01
TNFRSF8	8	-0.09	0.05	9.78E-02
TNFRSF9	5	0.18	0.12	1.41E-01
TNFSF10	12	0.01	0.03	8.30E-01
TNFSF11	8	0.02	0.04	5.85E-01
TNFSF12	16	0.05	0.04	2.02E-01
TNFSF13	7	-0.06	0.04	1.64E-01
TNFSF13B	6	0.06	0.09	4.86E-01
TNFSF14	5	0.07	0.05	1.32E-01
TNR	16	0.06	0.03	5.97E-02
TNXB	6	0.05	0.03	1.03E-01
TP53	1	0.01	0.24	9.59E-01
TP53INP1	3	-0.03	0.12	7.71E-01
TPMT	2	-0.03	0.03	3.57E-01
TPP1	11	0.07	0.06	2.23E-01
TPPP3	1	-0.21	0.22	3.48E-01
TPSAB1	13	-0.01	0.03	7.46E-01
TRAF2	3	0.06	0.14	6.82E-01
TREM2	7	0.06	0.03	4.61E-02
TREML2	8	-0.03	0.06	6.39E-01
TRIAP1	5	-0.08	0.09	3.41E-01
TRIM21	2	-0.55	0.21	1.05E-02
TRIM5	1	0.00	0.05	9.36E-01
TSHB	16	0.00	0.05	9.38E-01
TSPAN1	3	0.03	0.20	8.67E-01

TST	1	-0.03	0.20	8.94E-01
TXLNA	3	0.02	0.11	8.86E-01
TXNDC15	11	0.03	0.02	1.11E-01
TXNDC5	4	0.09	0.10	3.50E-01
TXNRD1	4	0.00	0.12	9.76E-01
TYMP	2	-0.07	0.07	3.55E-01
TYRO3	8	0.03	0.02	1.81E-01
UBAC1	1	0.08	0.32	8.00E-01
ULBP2	7	0.04	0.03	1.39E-01
UMOD	16	-0.01	0.02	5.86E-01
USO1	3	-0.03	0.14	8.40E-01
USP8	4	0.06	0.09	5.02E-01
UXS1	7	-0.05	0.05	3.27E-01
VAMP5	2	0.76	0.15	5.60E-07
VASH1	4	0.07	0.09	3.98E-01
VASN	5	-0.06	0.03	8.65E-02
VAT1	9	-0.02	0.06	7.58E-01
VCAM1	10	-0.14	0.06	1.67E-02
VCAN	14	0.00	0.02	8.44E-01
VEGFA	13	0.00	0.02	8.83E-01
VEGFC	16	0.15	0.05	3.54E-03
VEGFD	6	-0.06	0.07	3.78E-01
VMO1	7	-0.03	0.02	1.52E-01
VNN2	9	0.02	0.02	3.19E-01
VPS37A	2	-0.01	0.19	9.41E-01
VPS53	2	0.00	0.18	9.78E-01
VSIG4	8	0.19	0.10	5.09E-02
VSIR	8	0.10	0.04	1.15E-02
VSTM1	2	0.00	0.02	8.05E-01
VSTM2L	2	-0.13	0.09	1.39E-01
VTA1	3	0.01	0.14	9.21E-01
VTCN1	2	-0.81	0.15	6.74E-08
VWA1	7	0.03	0.06	6.59E-01
VWC2	3	0.03	0.03	2.99E-01
VWF	6	0.25	0.04	1.77E-08
WARS	3	-0.12	0.06	4.05E-02
WAS	3	-0.10	0.12	4.35E-01
WASF1	3	-0.03	0.05	5.43E-01
WASF3	1	0.05	0.10	6.05E-01
WFDC12	12	0.03	0.04	3.57E-01
WFDC2	3	-0.37	0.12	2.52E-03
WFIKKN1	14	-0.08	0.05	1.19E-01
WFIKKN2	4	0.02	0.03	4.66E-01
WIF1	12	-0.09	0.05	4.48E-02
WNT9A	1	0.19	0.20	3.32E-01
WWP2	3	-0.04	0.07	5.66E-01
XCL1	10	0.00	0.02	9.26E-01
XG	1	0.23	0.26	3.84E-01
XPNPEP2	12	0.00	0.06	9.81E-01
XRCC4	1	0.21	0.22	3.40E-01
YES1	2	0.05	0.15	7.18E-01
YTHDF3	1	0.16	0.17	3.51E-01

ZBTB16	7	-0.05	0.07	4.68E-01
ZBTB17	2	-0.10	0.16	5.36E-01

Supplementary Table 3. Associations of genetically predicted proteins with COVID-19 hospitalization using IVW method and cis+trans SNPs.

Protein	No. of SNPs	Beta	SE	Pval
AARSD1	3	0.00	0.07	9.83E-01
ABHD14B	4	-0.04	0.04	2.41E-01
ABL1	1	-0.03	0.12	8.04E-01
ACAA1	3	0.12	0.08	1.29E-01
ACAN	15	-0.01	0.03	8.49E-01
ACE2	9	-0.01	0.05	9.15E-01
ACOX1	1	-0.07	0.14	5.87E-01
ACP5	9	0.02	0.03	4.21E-01
ACP6	2	-0.01	0.01	4.02E-01
ACTA2	1	0.17	0.18	3.62E-01
ACVRL1	3	0.03	0.03	3.11E-01
ACY1	3	-0.01	0.06	8.91E-01
ADA	4	-0.01	0.02	4.45E-01
ADA2	13	-0.05	0.03	1.16E-01
ADAM15	8	0.02	0.01	1.96E-01
ADAM22	6	-0.02	0.02	3.02E-01
ADAM23	5	0.02	0.02	2.58E-01
ADAM8	7	0.04	0.02	4.95E-02
ADAMTS13	11	0.01	0.03	8.00E-01
ADAMTS15	5	-0.05	0.06	3.68E-01
ADAMTS16	1	-0.09	0.08	2.83E-01
ADAMTS8	4	0.01	0.02	7.19E-01
ADGRB3	5	0.02	0.01	1.21E-01
ADGRE2	8	-0.02	0.03	4.42E-01
ADGRE5	9	0.02	0.03	5.35E-01
ADGRG1	9	-0.21	0.05	3.04E-06
ADGRG2	6	0.47	0.07	1.91E-11
ADH4	6	-0.05	0.05	3.13E-01
ADM	1	0.21	0.32	5.08E-01
AFP	22	-0.03	0.02	1.39E-01
AGER	15	0.03	0.04	3.92E-01
AGR2	4	0.04	0.07	5.52E-01
AGR3	3	0.05	0.10	6.36E-01
AGRN	6	0.01	0.03	7.85E-01
AGRP	8	0.03	0.06	6.46E-01
AGXT	11	-0.04	0.03	2.46E-01
AHCY	1	-0.01	0.03	6.16E-01
AHSP	7	0.06	0.06	2.45E-01
AIF1	1	0.24	0.09	5.95E-03
AIFM1	2	-0.04	0.13	7.67E-01
AKR1B1	2	-0.08	0.07	2.45E-01
AKR1C4	1	0.06	0.19	7.52E-01
AKT1S1	3	-0.07	0.09	4.48E-01
AKT3	2	-0.20	0.14	1.53E-01
ALCAM	11	0.00	0.04	9.32E-01
ALDH1A1	3	-0.11	0.09	2.21E-01
ALDH3A1	1	-0.01	0.02	7.48E-01
ALPP	10	0.06	0.02	9.97E-03
AMBP	9	-0.03	0.04	5.01E-01
AMIGO2	11	-0.07	0.03	5.94E-03
AMN	5	0.00	0.01	9.88E-01
AMY2A	8	-0.14	0.03	8.73E-08
AMY2B	9	-0.11	0.02	3.03E-07
ANG	3	0.04	0.02	8.39E-02
ANGPT1	17	0.02	0.03	5.74E-01
ANGPT2	6	0.11	0.05	1.72E-02
ANGPTL1	6	0.01	0.03	6.87E-01
ANGPTL2	5	0.02	0.06	6.87E-01
ANGPTL3	6	-0.08	0.06	2.05E-01

ANGPTL4	1	-0.04	0.03	2.75E-01
ANGPTL7	6	0.04	0.03	1.98E-01
ANKRD54	1	0.00	0.17	9.82E-01
ANPEP	7	0.06	0.02	1.25E-02
ANXA11	3	-0.23	0.11	3.66E-02
ANXA3	5	-0.09	0.07	2.17E-01
ANXA4	2	-0.08	0.11	4.62E-01
ANXA5	1	0.28	0.09	1.51E-03
AOC1	1	-0.03	0.04	4.02E-01
AOC3	10	0.02	0.02	3.05E-01
APBB1IP	5	0.01	0.04	8.34E-01
APEX1	3	-0.01	0.03	7.88E-01
APLP1	5	-0.01	0.06	8.94E-01
APOH	1	0.02	0.02	3.36E-01
APOM	9	0.03	0.03	2.52E-01
APP	17	0.04	0.03	2.46E-01
APRT	6	-0.11	0.06	7.30E-02
AREG	3	0.21	0.05	9.95E-05
ARG1	5	-0.02	0.04	6.92E-01
ARHGAP1	2	-0.13	0.09	1.50E-01
ARHGAP25	2	0.05	0.09	6.28E-01
ARSA	7	0.01	0.01	4.89E-01
ARSB	11	0.04	0.03	1.25E-01
ART3	4	0.13	0.08	1.36E-01
ARTN	1	0.15	0.21	4.79E-01
ASAH2	16	-0.01	0.02	5.90E-01
ASGR1	6	0.01	0.04	8.88E-01
ATG4A	3	0.12	0.10	2.40E-01
ATOX1	4	0.08	0.08	3.05E-01
ATP5IF1	6	0.06	0.06	3.39E-01
ATP6V1F	1	-0.04	0.15	8.04E-01
ATXN10	5	-0.02	0.06	7.49E-01
AXIN1	3	-0.05	0.11	6.03E-01
AXL	8	0.01	0.04	7.62E-01
AZU1	2	-0.30	0.13	1.70E-02
B4GALT1	7	0.05	0.03	4.58E-02
B4GAT1	9	-0.04	0.03	1.36E-01
BACH1	3	-0.04	0.11	6.97E-01
BAG3	4	-0.04	0.07	5.75E-01
BAIAP2	5	0.01	0.07	9.37E-01
BAMBI	1	0.04	0.15	8.04E-01
BANK1	3	-0.01	0.07	8.81E-01
BAX	2	-0.16	0.12	1.71E-01
BCAM	14	0.02	0.02	4.59E-01
BCAN	7	0.08	0.04	4.37E-02
BCL2L11	10	-0.03	0.02	1.61E-01
BCR	1	0.14	0.21	5.06E-01
BGN	11	0.03	0.04	3.99E-01
BID	2	0.01	0.11	9.49E-01
BIN2	3	-0.04	0.08	6.03E-01
BIRC2	4	-0.08	0.10	4.14E-01
BLMH	3	0.00	0.02	9.23E-01
BLVRB	2	0.03	0.06	5.59E-01
BMP4	5	-0.01	0.07	9.16E-01
BMP6	15	-0.02	0.03	3.76E-01
BOC	8	0.06	0.04	1.98E-01
BPIFB1	15	-0.02	0.02	3.20E-01
BRK1	8	0.00	0.04	9.19E-01
BSG	10	-0.15	0.04	4.08E-04
BST1	4	0.00	0.01	9.75E-01
BST2	11	0.11	0.04	1.02E-02

BTC	3	0.01	0.02	5.97E-01
BTN2A1	7	0.05	0.03	1.54E-01
BTN3A2	3	-0.02	0.02	1.32E-01
C19orf12	5	-0.06	0.07	3.74E-01
C1QA	13	0.01	0.02	6.90E-01
C1QTNF1	4	0.06	0.04	1.41E-01
C2	2	0.01	0.10	9.11E-01
C2CD2L	1	0.02	0.19	9.05E-01
C4BPB	10	-0.01	0.03	6.35E-01
CA1	4	0.05	0.06	4.04E-01
CA11	2	-0.04	0.06	5.65E-01
CA12	7	0.08	0.03	9.61E-03
CA13	3	-0.08	0.04	4.29E-02
CA14	5	0.04	0.05	3.69E-01
CA2	2	-0.05	0.09	6.10E-01
CA3	6	0.05	0.04	2.29E-01
CA4	9	0.12	0.02	3.11E-07
CA5A	5	-0.03	0.02	3.18E-02
CA6	12	-0.02	0.01	1.21E-01
CA9	5	0.07	0.05	1.88E-01
CALB1	3	-0.05	0.08	5.08E-01
CALCA	7	-0.02	0.06	7.67E-01
CALCOCO1	4	-0.09	0.07	1.93E-01
CAMKK1	3	0.00	0.03	9.13E-01
CANT1	7	0.05	0.05	2.84E-01
CAPG	2	0.02	0.01	1.17E-01
CARHSP1	1	0.13	0.12	2.84E-01
CASP1	1	0.00	0.04	9.91E-01
CASP10	3	-0.08	0.05	1.30E-01
CASP2	2	-0.02	0.09	7.95E-01
CASP3	2	0.08	0.08	3.44E-01
CASP8	3	-0.04	0.04	3.83E-01
CBLIF	17	-0.07	0.02	1.71E-04
CBLN4	5	0.01	0.05	8.40E-01
CC2D1A	2	0.12	0.12	3.35E-01
CCDC80	5	0.18	0.05	3.04E-04
CCL11	9	-0.03	0.03	3.03E-01
CCL13	14	0.01	0.03	6.67E-01
CCL14	6	0.04	0.02	2.38E-02
CCL15	4	0.25	0.05	1.54E-07
CCL16	8	0.01	0.01	3.31E-01
CCL17	14	-0.01	0.02	6.24E-01
CCL18	4	0.03	0.02	1.57E-01
CCL19	5	-0.03	0.06	6.53E-01
CCL2	8	0.06	0.03	2.63E-02
CCL20	5	-0.16	0.06	8.00E-03
CCL21	10	-0.03	0.05	5.49E-01
CCL22	8	-0.10	0.05	2.83E-02
CCL23	8	-0.04	0.02	1.36E-01
CCL24	11	0.01	0.01	3.96E-01
CCL25	9	0.04	0.01	2.51E-03
CCL26	7	0.01	0.03	7.41E-01
CCL27	7	-0.04	0.04	3.47E-01
CCL28	18	-0.09	0.03	1.58E-03
CCL3	6	0.09	0.07	2.03E-01
CCL4	5	-0.10	0.03	5.25E-05
CCL5	9	-0.01	0.03	8.46E-01
CCL7	4	-0.04	0.03	1.83E-01
CCL8	10	0.02	0.01	6.55E-02
CCN1	11	-0.02	0.04	5.70E-01
CCN2	17	0.03	0.02	1.66E-01

CCN3	3	-0.02	0.04	5.86E-01
CCN4	12	-0.03	0.02	2.12E-01
CCN5	1	0.04	0.20	8.39E-01
CCS	1	0.01	0.02	5.64E-01
CD109	6	-0.31	0.04	9.04E-13
CD14	6	-0.04	0.03	2.26E-01
CD160	14	-0.01	0.02	6.04E-01
CD163	18	-0.02	0.02	4.98E-01
CD164	10	-0.04	0.04	3.93E-01
CD177	3	0.01	0.02	5.15E-01
CD1C	18	-0.05	0.03	7.13E-02
CD200	8	0.02	0.04	6.32E-01
CD200R1	9	-0.01	0.01	2.32E-01
CD207	15	-0.02	0.02	1.90E-01
CD209	8	0.10	0.01	1.52E-20
CD22	21	0.01	0.03	8.39E-01
CD244	14	-0.05	0.03	4.65E-02
CD27	6	0.00	0.03	9.77E-01
CD274	7	0.02	0.03	4.35E-01
CD276	7	-0.01	0.01	4.91E-01
CD28	10	0.03	0.03	3.81E-01
CD2AP	1	-0.04	0.15	8.04E-01
CD300C	14	0.03	0.02	1.26E-01
CD300E	10	-0.02	0.03	4.20E-01
CD300LF	9	0.01	0.01	2.08E-01
CD300LG	12	0.01	0.02	5.52E-01
CD302	7	-0.01	0.03	6.69E-01
CD33	7	0.02	0.01	4.25E-02
CD34	3	-0.19	0.02	2.28E-14
CD38	2	-0.03	0.03	3.35E-01
CD4	4	-0.04	0.03	1.33E-01
CD40	9	-0.02	0.02	4.91E-01
CD40LG	12	0.11	0.04	4.05E-03
CD46	7	-0.20	0.06	3.18E-04
CD48	13	0.00	0.01	7.97E-01
CD5	8	0.01	0.04	8.40E-01
CD55	6	0.02	0.02	2.74E-01
CD58	12	-0.13	0.02	3.22E-10
CD59	5	0.03	0.04	4.43E-01
CD6	11	-0.02	0.01	2.54E-01
CD63	10	0.01	0.04	8.76E-01
CD69	6	-0.05	0.05	3.34E-01
CD70	3	-0.01	0.01	4.86E-01
CD74	6	-0.03	0.04	4.16E-01
CD79B	17	-0.12	0.03	1.28E-05
CD83	14	-0.05	0.03	1.31E-01
CD84	11	0.02	0.03	5.93E-01
CD8A	5	0.02	0.03	5.21E-01
CD93	5	-0.08	0.07	2.59E-01
CD99	4	0.13	0.07	5.71E-02
CD99L2	9	-0.01	0.04	7.98E-01
CDC27	2	0.00	0.10	9.84E-01
CDC37	2	-0.03	0.11	7.79E-01
CDCP1	8	-0.04	0.03	2.18E-01
CDH1	6	0.01	0.02	6.40E-01
CDH15	4	0.16	0.03	9.08E-08
CDH17	12	-0.04	0.02	2.20E-02
CDH2	13	0.04	0.04	3.66E-01
CDH3	7	-0.13	0.06	3.09E-02
CDH5	12	0.03	0.01	6.51E-02
CDH6	6	0.01	0.02	7.60E-01

CDHR1	4	0.07	0.04	7.11E-02
CDHR2	8	0.18	0.06	3.32E-03
CDHR5	8	-0.03	0.02	1.26E-01
CDKN1A	5	-0.01	0.05	7.90E-01
CDKN2D	4	0.05	0.06	4.65E-01
CDNF	1	0.00	0.03	9.23E-01
CDON	4	0.09	0.07	1.80E-01
CDSN	16	-0.04	0.04	3.30E-01
CEACAM1	6	0.00	0.03	8.79E-01
CEACAM21	2	-0.11	0.20	5.62E-01
CEACAM3	1	-0.11	0.18	5.48E-01
CEACAM5	11	-0.05	0.02	4.30E-03
CEACAM8	12	-0.01	0.03	8.03E-01
CEBPB	3	0.19	0.11	8.04E-02
CELA3A	15	-0.04	0.02	7.07E-02
CEP20	4	0.01	0.06	8.58E-01
CEP43	6	-0.09	0.07	1.81E-01
CEP85	2	-0.02	0.07	7.66E-01
CERT	4	-0.03	0.08	6.64E-01
CES1	18	0.03	0.02	1.10E-01
CES2	2	0.04	0.10	6.61E-01
CES3	9	-0.05	0.04	2.29E-01
CETN2	3	0.00	0.08	9.63E-01
CFC1	8	0.01	0.06	9.07E-01
CGA	1	-0.30	0.26	2.50E-01
CGREF1	3	0.02	0.02	1.90E-01
CHAC2	2	0.05	0.08	5.48E-01
CHGB	6	-0.04	0.02	2.22E-02
CHI3L1	6	0.00	0.02	9.02E-01
CHIT1	4	-0.01	0.01	3.04E-01
CHL1	6	0.01	0.03	7.33E-01
CHMP1A	4	0.02	0.08	7.50E-01
CHRDL1	4	0.00	0.10	9.96E-01
CHRDL2	4	0.02	0.09	8.44E-01
CIAPIN1	4	-0.06	0.08	4.72E-01
CKAP4	4	0.01	0.03	6.30E-01
CKMT1A_CKMT1B	6	0.22	0.06	1.18E-04
CLC	9	0.02	0.04	5.45E-01
CLEC10A	9	0.01	0.02	4.38E-01
CLEC11A	7	-0.06	0.03	7.21E-02
CLEC14A	8	-0.15	0.03	6.25E-07
CLEC1A	7	0.04	0.02	5.21E-02
CLEC1B	9	0.06	0.04	1.14E-01
CLEC4A	12	-0.01	0.02	6.04E-01
CLEC4C	25	0.01	0.01	2.56E-01
CLEC4D	10	-0.01	0.02	6.53E-01
CLEC4G	10	0.17	0.03	1.78E-11
CLEC5A	10	0.05	0.03	9.00E-02
CLEC6A	13	0.00	0.02	9.70E-01
CLEC7A	3	0.01	0.01	2.05E-01
CLIP2	2	0.02	0.07	7.35E-01
CLMP	4	0.07	0.03	3.85E-02
CLPS	7	-0.03	0.02	2.00E-01
CLSPN	1	0.01	0.10	8.84E-01
CLSTN2	3	0.01	0.04	7.87E-01
CLTA	1	-0.07	0.08	4.21E-01
CLUL1	9	0.00	0.02	8.64E-01
CNDP1	8	-0.05	0.02	3.59E-02
CNPY2	1	0.07	0.08	4.31E-01
CNPY4	3	-0.08	0.05	1.16E-01
CNST	4	-0.06	0.09	5.24E-01

CNTN1	7	0.05	0.03	8.42E-02
CNTN2	6	0.04	0.02	1.63E-02
CNTN3	16	-0.06	0.03	2.19E-02
CNTN4	10	0.02	0.03	6.14E-01
CNTN5	6	0.02	0.02	4.54E-01
CNTNAP2	4	0.04	0.02	5.01E-02
COL18A1	4	-0.03	0.05	5.95E-01
COL1A1	10	-0.12	0.04	5.75E-03
COL4A1	9	-0.01	0.03	8.21E-01
COL6A3	4	0.02	0.08	8.47E-01
COL9A1	4	0.13	0.07	6.68E-02
COLEC12	4	0.09	0.09	2.95E-01
COMP	6	0.11	0.04	5.98E-03
COMT	2	-0.05	0.02	2.84E-02
CORO1A	3	0.13	0.10	1.77E-01
COX5B	4	-0.13	0.08	1.29E-01
CPA1	21	-0.03	0.03	3.22E-01
CPA2	4	0.02	0.03	4.13E-01
CPB1	13	-0.03	0.03	3.00E-01
CPE	7	-0.11	0.04	2.28E-03
CPM	11	-0.02	0.04	6.03E-01
CPPED1	1	-0.02	0.02	2.47E-01
CPVL	12	0.00	0.01	8.88E-01
CPXM1	18	-0.01	0.02	6.36E-01
CR2	23	0.00	0.03	9.72E-01
CRACR2A	4	-0.03	0.06	6.75E-01
CRADD	4	-0.05	0.08	5.54E-01
CREG1	11	0.04	0.04	2.82E-01
CRELD2	11	-0.01	0.03	6.06E-01
CRH	23	0.01	0.02	6.42E-01
CRHBP	5	-0.01	0.02	7.28E-01
CRIM1	4	-0.18	0.08	2.67E-02
CRIP2	4	0.20	0.06	1.07E-03
CRISP2	13	0.00	0.02	8.26E-01
CRKL	1	-0.03	0.15	8.35E-01
CRLF1	7	-0.04	0.07	5.67E-01
CRNN	3	0.03	0.01	3.01E-02
CRTAC1	2	0.01	0.01	5.87E-01
CRTAM	14	-0.01	0.03	6.48E-01
CSF1	3	-0.07	0.05	1.47E-01
CSF2RA	1	0.13	0.19	4.88E-01
CSF3	3	0.05	0.06	4.24E-01
CST3	6	-0.02	0.04	6.20E-01
CST5	13	-0.01	0.01	6.61E-01
CST6	15	0.04	0.03	1.50E-01
CST7	11	0.00	0.01	7.73E-01
CTF1	2	0.00	0.14	9.93E-01
CTRB1	12	-0.02	0.02	1.95E-01
CTRC	11	0.00	0.02	8.30E-01
CTSB	7	0.06	0.02	3.59E-03
CTSC	7	0.03	0.02	4.74E-02
CTSD	6	0.06	0.03	2.15E-02
CTSF	15	0.00	0.02	9.19E-01
CTSH	1	0.00	0.11	9.71E-01
CTSL	17	0.00	0.03	8.85E-01
CTSO	10	0.01	0.03	7.25E-01
CTSS	7	-0.26	0.05	2.40E-06
CTSV	17	-0.05	0.03	1.19E-01
CTSZ	9	0.00	0.02	9.01E-01
CX3CL1	7	-0.42	0.06	8.37E-13
CXADR	5	0.17	0.04	1.75E-05

CXCL1	4	0.03	0.02	1.19E-01
CXCL10	4	0.04	0.03	2.54E-01
CXCL11	8	-0.05	0.04	2.23E-01
CXCL12	9	-0.05	0.04	2.08E-01
CXCL13	8	-0.16	0.07	1.32E-02
CXCL14	2	0.15	0.11	1.91E-01
CXCL16	13	0.02	0.03	5.63E-01
CXCL17	5	-0.30	0.08	1.40E-04
CXCL3	3	0.02	0.08	7.83E-01
CXCL5	11	0.01	0.02	7.71E-01
CXCL6	7	-0.01	0.02	6.59E-01
CXCL8	8	0.06	0.04	1.35E-01
CXCL9	5	0.01	0.05	7.74E-01
DAB2	3	-0.15	0.10	1.22E-01
DAG1	3	0.03	0.11	7.83E-01
DAPP1	2	0.01	0.08	9.00E-01
DARS1	5	0.00	0.07	9.52E-01
DBI	3	0.05	0.04	1.87E-01
DBNL	4	0.04	0.09	6.67E-01
DCBLD2	16	-0.01	0.03	8.54E-01
DCN	1	0.02	0.16	9.22E-01
DCTN1	1	0.09	0.15	5.61E-01
DCTN2	2	-0.04	0.14	7.69E-01
DCTPP1	5	0.03	0.06	5.88E-01
DCXR	6	-0.02	0.06	7.10E-01
DDAH1	5	0.10	0.05	4.40E-02
DDC	7	-0.01	0.02	6.16E-01
DDR1	12	-0.01	0.02	6.21E-01
DDX58	1	-0.01	0.04	7.57E-01
DECR1	3	-0.02	0.09	8.14E-01
DEFA1_DEFA1B	15	0.02	0.04	6.30E-01
DEFB4A_DEFB4B	3	-0.10	0.09	2.88E-01
DFFA	1	-0.04	0.15	8.04E-01
DIABLO	3	-0.11	0.11	2.95E-01
DKK1	10	0.02	0.03	5.71E-01
DKK3	7	-0.03	0.03	1.85E-01
DKK4	6	0.03	0.03	4.02E-01
DKKL1	5	0.00	0.01	9.52E-01
DLK1	6	-0.01	0.02	5.32E-01
DLL1	6	0.00	0.04	9.24E-01
DNAJA2	1	-0.02	0.16	8.79E-01
DNAJB1	6	-0.03	0.06	6.37E-01
DNAJB8	2	0.05	0.11	6.83E-01
DNER	7	0.08	0.03	2.62E-02
DNMBP	3	-0.01	0.08	8.57E-01
DNPH1	1	-0.08	0.04	2.94E-02
DOK2	4	-0.06	0.08	4.20E-01
DPEP1	7	-0.02	0.01	3.07E-01
DPEP2	3	0.02	0.05	7.27E-01
DPP10	5	-0.07	0.03	1.98E-02
DPP4	5	0.01	0.04	8.66E-01
DPP6	7	-0.12	0.03	3.45E-04
DPP7	7	0.01	0.04	7.94E-01
DPT	2	-0.02	0.03	4.93E-01
DPY30	2	-0.10	0.13	4.35E-01
DRAXIN	17	-0.07	0.03	1.23E-02
DRG2	3	0.09	0.07	1.86E-01
DSC2	9	0.01	0.03	6.80E-01
DSG2	10	0.03	0.03	2.44E-01
DSG3	17	0.05	0.03	3.44E-02
DSG4	22	-0.05	0.03	9.54E-02

DTX3	3	-0.12	0.05	1.65E-02
DUSP3	1	-0.14	0.15	3.69E-01
EBAG9	5	0.05	0.07	4.56E-01
EBI3_IL27	19	-0.03	0.03	2.68E-01
ECE1	4	0.01	0.06	8.35E-01
EDA2R	3	0.16	0.12	2.09E-01
EDAR	23	0.01	0.02	4.93E-01
EDIL3	3	0.06	0.06	3.24E-01
EFEMP1	4	0.03	0.05	4.81E-01
EFNA1	4	0.13	0.03	3.89E-06
EFNA4	5	-0.01	0.07	8.90E-01
EGF	11	0.05	0.04	2.18E-01
EGFL7	18	0.00	0.03	9.91E-01
EGFR	6	-0.05	0.06	3.72E-01
EGLN1	4	-0.07	0.06	2.46E-01
EIF4B	3	-0.06	0.08	4.89E-01
EIF4G1	3	-0.03	0.08	7.24E-01
ELOA	2	0.04	0.07	5.84E-01
ENAH	1	0.03	0.10	7.43E-01
ENG	8	-0.12	0.02	4.58E-11
ENO1	4	0.13	0.07	4.69E-02
ENO2	5	-0.01	0.05	8.31E-01
ENPP2	8	-0.07	0.04	5.87E-02
ENPP5	7	0.00	0.01	8.02E-01
ENPP7	6	-0.01	0.01	6.88E-01
ENTPD2	2	0.21	0.16	1.86E-01
ENTPD5	9	-0.02	0.02	2.16E-01
ENTPD6	7	0.04	0.02	1.53E-02
EPCAM	14	0.08	0.02	9.68E-04
EPHA1	7	-0.05	0.04	2.53E-01
EPHA2	6	0.18	0.06	1.56E-03
EPHB4	10	-0.13	0.03	4.98E-07
EPHB6	4	0.01	0.04	8.04E-01
EPO	9	0.04	0.04	2.47E-01
EPS8L2	3	0.02	0.03	5.16E-01
ERBB2	9	0.03	0.04	4.40E-01
ERBB3	9	0.02	0.03	6.33E-01
ERBB4	16	-0.16	0.03	2.16E-07
ERBIN	3	0.07	0.11	4.79E-01
EREG	10	0.01	0.05	8.59E-01
ERP44	6	0.00	0.02	9.92E-01
ESAM	8	0.07	0.04	8.24E-02
ESM1	11	-0.06	0.04	1.34E-01
EZR	2	-0.03	0.12	8.29E-01
F11R	11	0.06	0.05	2.54E-01
F2R	7	-0.09	0.04	4.75E-02
F3	9	0.00	0.02	8.31E-01
F7	7	0.02	0.02	2.40E-01
F9	2	0.07	0.08	3.71E-01
FABP1	2	-0.01	0.04	7.23E-01
FABP2	5	0.11	0.05	1.51E-02
FABP4	2	0.12	0.09	2.15E-01
FABP5	2	0.09	0.11	4.21E-01
FABP6	4	-0.02	0.04	5.82E-01
FABP9	16	0.03	0.02	1.46E-01
FADD	3	-0.02	0.09	8.73E-01
FAM3B	6	-0.03	0.02	8.34E-02
FAM3C	2	-0.06	0.10	5.64E-01
FAP	7	-0.01	0.03	8.82E-01
FAS	4	-0.04	0.04	2.48E-01
FASLG	21	0.00	0.02	9.05E-01

FBP1	5	0.01	0.04	7.55E-01
FCAR	14	-0.04	0.01	5.22E-03
FCER2	14	0.00	0.03	8.64E-01
FCGR2A	5	-0.01	0.01	4.55E-01
FCGR2B	4	-0.06	0.02	1.42E-03
FCGR3B	11	-0.02	0.03	5.40E-01
FCN2	11	-0.03	0.01	3.07E-02
FCRL1	26	-0.05	0.02	9.49E-03
FCRL2	16	0.02	0.02	1.73E-01
FCRL3	5	-0.03	0.01	9.45E-03
FCRL5	9	0.02	0.04	6.14E-01
FCRL6	5	-0.02	0.01	2.21E-01
FCRLB	7	-0.01	0.02	4.65E-01
FEN1	1	0.04	0.19	8.31E-01
FES	1	0.03	0.07	6.27E-01
FETUB	4	-0.02	0.03	5.67E-01
FGF19	5	0.18	0.03	1.01E-07
FGF2	6	-0.01	0.01	4.90E-01
FGF21	7	-0.01	0.04	8.03E-01
FGF23	5	-0.09	0.06	1.29E-01
FGF5	2	-0.02	0.02	3.89E-01
FGFBP1	13	0.00	0.04	9.83E-01
FGFR2	8	0.21	0.06	2.28E-04
FGR	1	-0.14	0.13	2.83E-01
FHIT	2	-0.05	0.10	5.80E-01
FIS1	4	-0.07	0.05	1.75E-01
FKBP1B	4	0.08	0.04	5.34E-02
FKBP4	5	-0.01	0.07	9.09E-01
FKBP5	5	-0.01	0.04	8.49E-01
FLI1	3	-0.13	0.09	1.65E-01
FLRT2	11	-0.01	0.02	7.20E-01
FLT1	8	-0.05	0.03	4.67E-02
FLT3	6	-0.04	0.06	5.58E-01
FLT3LG	20	-0.02	0.02	5.14E-01
FLT4	11	-0.03	0.01	4.91E-03
FOLR1	8	-0.03	0.05	4.79E-01
FOLR2	14	-0.01	0.03	6.52E-01
FOLR3	10	-0.03	0.05	4.80E-01
FOXO1	4	0.03	0.06	5.67E-01
FOXO3	1	0.05	0.26	8.46E-01
FRZB	4	-0.01	0.02	5.66E-01
FST	3	-0.01	0.05	7.99E-01
FSTL3	2	0.01	0.10	9.19E-01
FUCA1	5	-0.01	0.01	3.18E-01
FURIN	3	-0.04	0.05	3.27E-01
FUS	2	0.00	0.10	9.69E-01
FUT3_FUT5	14	-0.02	0.02	1.37E-01
FUT8	12	0.01	0.01	4.41E-01
FXN	3	-0.08	0.10	4.41E-01
FXYD5	2	-0.06	0.10	5.26E-01
FYB1	2	0.00	0.11	9.86E-01
GAL	21	0.01	0.03	6.56E-01
GALNT10	9	0.00	0.03	8.74E-01
GALNT2	7	0.01	0.03	6.79E-01
GALNT3	8	0.01	0.02	6.21E-01
GALNT7	6	-0.02	0.03	5.22E-01
GAS6	15	-0.01	0.02	6.60E-01
GBP2	1	0.02	0.09	8.38E-01
GBP4	1	0.00	0.01	8.49E-01
GCG	5	0.02	0.08	8.16E-01
GCNT1	7	0.03	0.02	2.11E-01

GDF15	1	0.07	0.04	4.92E-02
GDF2	14	0.06	0.03	6.60E-02
GDNF	1	-0.02	0.04	6.35E-01
GFAP	8	-0.06	0.06	3.03E-01
GFER	1	-0.07	0.15	6.33E-01
GFRA1	4	0.09	0.04	1.29E-02
GFRA2	7	0.05	0.03	8.18E-02
GFRA3	21	-0.02	0.03	5.71E-01
GGH	9	-0.01	0.02	5.62E-01
GGT1	7	-0.02	0.04	5.39E-01
GGT5	4	-0.01	0.04	6.95E-01
GHRL	10	0.03	0.04	3.73E-01
GKN1	4	0.25	0.08	1.09E-03
GLB1	9	0.04	0.03	1.40E-01
GLO1	1	-0.03	0.03	3.20E-01
GLOD4	2	-0.04	0.13	7.46E-01
GLRX	2	-0.01	0.02	7.12E-01
GLT8D2	1	0.01	0.06	8.77E-01
GMPR	2	-0.03	0.04	5.01E-01
GNE	3	-0.07	0.07	2.75E-01
GNLY	12	0.00	0.02	9.81E-01
GOLM2	9	0.19	0.03	1.25E-12
GOPC	2	-0.03	0.11	7.47E-01
GP1BA	16	-0.01	0.03	8.45E-01
GP2	8	0.11	0.02	1.60E-12
GP6	6	0.01	0.03	7.74E-01
GPA33	13	0.06	0.02	4.68E-03
GPC1	17	-0.06	0.02	1.98E-02
GPC5	10	-0.01	0.01	5.03E-01
GPNMB	6	-0.03	0.03	1.97E-01
GPR37	10	0.00	0.01	9.39E-01
GRAP2	3	-0.06	0.10	5.18E-01
GRK5	1	-0.03	0.04	4.59E-01
GRN	12	-0.02	0.01	1.01E-01
GRPEL1	3	0.12	0.08	1.56E-01
GSAP	1	-0.06	0.15	7.14E-01
GSTA1	9	0.00	0.03	9.58E-01
GSTA3	5	0.01	0.03	8.07E-01
GSTP1	1	0.00	0.05	9.81E-01
GUCA2A	7	-0.05	0.05	3.63E-01
GUSB	12	0.00	0.03	9.82E-01
GYS1	3	0.03	0.04	4.10E-01
GZMA	15	-0.03	0.03	3.92E-01
GZMB	10	-0.03	0.03	2.78E-01
GZMH	8	-0.08	0.04	2.62E-02
HAGH	1	-0.12	0.14	3.70E-01
HAO1	8	0.01	0.06	9.25E-01
HARS1	2	-0.05	0.11	6.67E-01
HAVCR1	7	0.03	0.02	1.41E-01
HAVCR2	8	0.02	0.04	5.59E-01
HBEGF	23	0.01	0.03	6.14E-01
HBQ1	2	-0.03	0.03	2.86E-01
HCLS1	4	-0.03	0.07	6.97E-01
HDGF	2	-0.02	0.01	5.07E-03
HEBP1	1	0.01	0.08	9.17E-01
HEXIM1	4	-0.01	0.06	9.07E-01
HGF	5	-0.12	0.05	2.46E-02
HGS	4	-0.06	0.09	4.55E-01
HLA-DRA	3	-0.02	0.03	4.19E-01
HLA-E	4	0.05	0.03	4.91E-02
HMBS	1	0.03	0.08	7.02E-01

HMOX1	18	-0.03	0.03	2.66E-01
HMOX2	4	-0.06	0.06	3.79E-01
HNMT	4	0.00	0.01	8.49E-01
HNRNPK	1	-0.03	0.16	8.35E-01
HPGDS	2	0.03	0.02	2.41E-01
HS3ST3B1	10	0.00	0.03	8.56E-01
HS6ST1	15	0.00	0.03	9.99E-01
HSD11B1	12	0.04	0.03	1.45E-01
HSPA1A	3	-0.03	0.09	7.41E-01
HSPB1	5	0.02	0.06	7.68E-01
HSPB6	5	-0.17	0.09	7.01E-02
HSPG2	4	0.07	0.05	1.91E-01
HTRA2	1	-0.04	0.19	8.35E-01
HYAL1	3	0.02	0.04	5.00E-01
HYOU1	13	0.00	0.03	9.40E-01
ICA1	2	-0.13	0.05	4.06E-03
ICAM1	6	-0.09	0.02	3.16E-05
ICAM2	10	-0.05	0.01	9.22E-09
ICAM3	21	-0.04	0.02	2.76E-02
ICAM4	14	0.06	0.03	5.74E-02
ICAM5	7	-0.08	0.01	8.06E-11
ICOSLG	4	0.04	0.04	2.86E-01
IDI2	1	0.01	0.03	7.89E-01
IDS	3	-0.13	0.04	2.50E-03
IDUA	13	0.01	0.02	5.63E-01
IFNG	2	-0.41	0.15	5.58E-03
IFNGR1	10	0.04	0.03	2.65E-01
IFNGR2	9	-0.02	0.01	1.01E-01
IFNL1	5	0.00	0.07	9.72E-01
IFNLR1	3	-0.02	0.04	5.50E-01
IGF1R	9	-0.17	0.02	3.84E-14
IGF2R	14	-0.03	0.02	1.45E-01
IGFBP1	2	-0.07	0.10	4.50E-01
IGFBP2	4	-0.03	0.09	7.54E-01
IGFBP3	5	0.01	0.02	8.11E-01
IGFBP4	3	-0.02	0.11	8.21E-01
IGFBP6	3	-0.02	0.10	8.32E-01
IGFBP7	3	0.01	0.03	7.76E-01
IGFBPL1	2	0.02	0.02	3.19E-01
IGSF3	5	0.04	0.06	4.83E-01
IGSF8	5	0.03	0.05	5.48E-01
IKBKG	1	-0.05	0.20	8.04E-01
IKZF2	2	-0.10	0.09	2.72E-01
IL10	5	0.00	0.04	9.51E-01
IL10RA	1	0.02	0.07	7.39E-01
IL10RB	6	0.01	0.02	7.06E-01
IL11	1	-0.02	0.26	9.54E-01
IL12A_IL12B	17	-0.04	0.02	1.04E-02
IL12B	20	-0.04	0.02	9.39E-03
IL12RB1	5	0.00	0.02	9.70E-01
IL13	1	-0.09	0.17	5.99E-01
IL13RA1	2	0.02	0.11	8.29E-01
IL15	21	0.01	0.03	7.81E-01
IL15RA	1	0.02	0.04	6.87E-01
IL16	1	-0.14	0.15	3.40E-01
IL17C	3	-0.12	0.08	1.08E-01
IL17D	3	-0.06	0.03	3.70E-02
IL17F	1	0.15	0.21	4.54E-01
IL17RA	3	0.01	0.01	5.03E-01
IL17RB	11	-0.01	0.01	3.31E-01
IL18	9	-0.05	0.02	3.25E-02

IL18BP	9	-0.06	0.04	2.03E-01
IL18R1	12	-0.01	0.01	2.92E-01
IL18RAP	3	0.12	0.07	9.54E-02
IL19	4	-0.07	0.02	9.35E-04
IL1A	1	0.07	0.08	3.64E-01
IL1B	2	0.00	0.04	9.45E-01
IL1R1	13	-0.02	0.02	4.97E-01
IL1R2	14	0.02	0.02	2.36E-01
IL1RAP	4	-0.01	0.01	1.84E-01
IL1RL1	13	0.00	0.01	8.37E-01
IL1RL2	12	0.01	0.02	6.90E-01
IL1RN	2	0.01	0.05	8.95E-01
IL20RA	1	0.03	0.19	8.87E-01
IL22RA1	2	0.10	0.03	2.08E-03
IL2RA	8	0.00	0.02	8.21E-01
IL32	7	-0.02	0.04	6.81E-01
IL33	1	-0.04	0.31	8.90E-01
IL34	1	-0.02	0.02	2.17E-01
IL3RA	4	-0.03	0.08	6.67E-01
IL4R	5	-0.03	0.03	2.87E-01
IL5RA	23	0.06	0.02	6.46E-03
IL6	4	-0.07	0.04	1.38E-01
IL6R	5	-0.02	0.01	5.44E-02
IL6ST	10	-0.09	0.03	8.23E-04
IL7	3	-0.11	0.08	1.43E-01
IL7R	12	-0.04	0.01	5.39E-05
ILKAP	1	-0.04	0.17	8.35E-01
IMPA1	2	-0.05	0.05	3.39E-01
ING1	1	-0.04	0.18	8.35E-01
INHBC	6	-0.01	0.02	4.83E-01
INPP1	3	-0.15	0.09	9.42E-02
IPCEF1	2	-0.01	0.10	8.81E-01
IQGAP2	1	0.02	0.07	8.22E-01
IRAG2	2	-0.27	0.12	2.23E-02
IRAK1	1	-0.04	0.17	8.04E-01
ISLR2	8	-0.12	0.02	6.66E-13
ISM1	2	-0.04	0.06	5.06E-01
ITGA11	5	0.07	0.04	7.52E-02
ITGA5	10	0.00	0.04	8.98E-01
ITGA6	7	-0.06	0.03	4.50E-02
ITGAM	9	-0.03	0.04	4.41E-01
ITGAV	5	-0.03	0.05	6.09E-01
ITGB1	7	-0.12	0.05	1.02E-02
ITGB1BP2	4	0.05	0.07	5.22E-01
ITGB2	10	-0.07	0.04	6.59E-02
ITGB5	5	0.06	0.06	3.38E-01
ITGB6	9	-0.12	0.03	1.45E-04
ITGB7	18	0.03	0.02	1.05E-01
ITIH3	7	-0.03	0.03	1.97E-01
ITM2A	2	0.06	0.05	2.11E-01
JAM2	6	0.09	0.04	4.18E-02
JCHAIN	14	0.04	0.05	3.99E-01
KAZALD1	6	-0.02	0.01	2.26E-01
KDR	12	-0.01	0.02	6.06E-01
KEL	17	-0.09	0.02	2.79E-08
KIFBP	3	0.05	0.07	4.44E-01
KIR2DL3	4	0.02	0.02	4.75E-01
KIR3DL1	1	0.66	0.28	1.79E-02
KIRREL2	8	0.03	0.02	1.87E-01
KIT	23	-0.01	0.03	6.59E-01
KITLG	15	-0.03	0.03	2.73E-01

KLB	9	0.02	0.01	1.29E-01
KLK1	13	-0.05	0.01	7.58E-07
KLK10	4	0.02	0.02	1.68E-01
KLK11	5	-0.03	0.03	3.36E-01
KLK12	5	0.01	0.01	2.18E-01
KLK13	10	0.04	0.02	3.11E-02
KLK14	10	0.00	0.02	8.71E-01
KLK4	3	-0.02	0.03	5.60E-01
KLK6	2	-0.03	0.06	6.48E-01
KLK8	4	0.04	0.03	1.66E-01
KLRB1	11	-0.06	0.04	1.39E-01
KLRD1	13	-0.01	0.02	7.06E-01
KRT18	9	-0.12	0.05	1.72E-02
KRT19	3	0.19	0.06	3.11E-03
KRT5	4	0.08	0.05	1.46E-01
KYAT1	4	-0.06	0.08	4.80E-01
KYNU	4	-0.05	0.04	1.42E-01
L1CAM	6	0.03	0.05	4.88E-01
LACTB2	3	0.09	0.07	1.79E-01
LAG3	12	-0.08	0.03	7.40E-03
LAIR1	4	0.02	0.02	3.26E-01
LAIR2	5	-0.01	0.01	6.66E-01
LAMA4	11	0.01	0.03	8.34E-01
LAMP2	2	0.09	0.14	5.24E-01
LAMP3	10	-0.17	0.04	9.72E-06
LAT	6	0.03	0.06	6.02E-01
LAT2	1	-0.04	0.16	8.04E-01
LAYN	4	0.01	0.02	5.54E-01
LBP	2	-0.03	0.02	4.71E-02
LBR	6	-0.08	0.04	9.01E-02
LCN2	17	-0.01	0.04	7.80E-01
LDLR	16	0.02	0.03	4.98E-01
LEFTY2	4	-0.56	0.09	8.93E-10
LEP	1	0.41	0.22	6.02E-02
LEPR	9	0.02	0.03	5.48E-01
LGALS1	2	0.01	0.04	8.20E-01
LGALS3	5	-0.01	0.02	5.65E-01
LGALS4	3	-0.18	0.02	4.66E-22
LGALS7_LGALS7B	7	0.16	0.02	6.22E-11
LGALS8	6	-0.13	0.02	2.58E-10
LGALS9	4	-0.06	0.03	1.51E-02
LGMN	19	-0.01	0.03	6.20E-01
LHB	1	0.27	0.18	1.35E-01
LIFR	11	-0.15	0.02	1.32E-11
LILRA2	11	-0.06	0.02	4.10E-03
LILRA5	9	-0.04	0.04	3.00E-01
LILRB1	9	0.00	0.02	9.12E-01
LILRB2	10	-0.02	0.01	4.82E-02
LILRB4	4	-0.03	0.05	5.91E-01
LILRB5	3	0.00	0.01	8.65E-01
LPCAT2	1	-0.01	0.09	8.65E-01
LPL	8	-0.04	0.02	1.28E-01
LPO	12	0.01	0.02	5.98E-01
LRIG1	11	-0.01	0.01	6.62E-01
LRP1	11	-0.02	0.01	1.87E-01
LRP11	6	0.01	0.02	4.47E-01
LRPAP1	7	0.00	0.01	7.33E-01
LRRC25	13	0.00	0.02	8.97E-01
LRRN1	7	0.00	0.01	9.21E-01
LSP1	2	-0.07	0.07	3.22E-01
LTA	10	0.01	0.01	3.78E-01

LTA4H	1	-0.07	0.10	4.89E-01
LTBP2	7	-0.06	0.06	3.16E-01
LTBP3	6	-0.02	0.02	3.91E-01
LTBR	13	0.00	0.02	9.86E-01
LXN	2	0.01	0.05	7.82E-01
LY6D	5	0.01	0.03	6.07E-01
LY75	6	-0.01	0.01	2.77E-01
LY9	14	0.01	0.02	6.86E-01
LY96	11	0.01	0.05	8.30E-01
LYAR	1	-0.16	0.20	4.20E-01
LYN	5	-0.18	0.08	2.16E-02
LYPD3	17	-0.04	0.02	2.58E-02
LYPD8	7	0.01	0.02	7.63E-01
MAD1L1	1	-0.07	0.10	4.94E-01
MAGED1	2	-0.03	0.10	7.76E-01
MANF	6	-0.05	0.07	4.50E-01
MANSC1	17	-0.03	0.04	4.97E-01
MAP2K6	2	-0.15	0.11	1.71E-01
MAP3K5	1	-0.04	0.18	8.04E-01
MAP4K5	3	0.06	0.05	2.58E-01
MAPK9	1	-0.11	0.08	1.77E-01
MARCO	9	-0.07	0.03	5.47E-03
MASP1	13	0.00	0.02	9.99E-01
MATN2	5	-0.01	0.02	5.60E-01
MATN3	4	-0.02	0.02	2.77E-01
MAVS	3	-0.01	0.08	9.26E-01
MAX	5	0.09	0.07	2.20E-01
MB	8	0.02	0.06	7.18E-01
MCAM	8	0.11	0.04	2.68E-03
MCFD2	4	0.04	0.06	5.02E-01
MDGA1	6	0.00	0.01	6.27E-01
MDK	11	-0.01	0.05	9.13E-01
MED18	1	-0.35	0.17	3.59E-02
MEGF10	13	-0.01	0.02	6.88E-01
MEGF9	11	0.11	0.03	1.30E-04
MEP1B	15	0.02	0.01	5.82E-02
MEPE	14	-0.01	0.04	7.35E-01
MERTK	13	0.04	0.03	2.14E-01
MESD	4	0.06	0.08	4.10E-01
MET	20	-0.13	0.02	3.01E-08
METAP1D	2	-0.13	0.12	2.66E-01
METAP2	4	-0.06	0.07	3.42E-01
MFAP5	6	-0.09	0.06	9.18E-02
MFGE8	11	-0.01	0.02	5.60E-01
MGLL	3	0.06	0.09	4.93E-01
MGMT	3	0.00	0.02	9.31E-01
MIA	7	-0.02	0.01	2.35E-01
MICB_MICA	8	-0.02	0.01	1.26E-02
MIF	3	0.06	0.08	3.98E-01
MILR1	9	0.00	0.03	8.81E-01
MITD1	2	0.03	0.11	7.78E-01
MLN	18	0.02	0.02	3.52E-01
MME	13	0.00	0.02	7.77E-01
MMP1	12	0.00	0.02	9.38E-01
MMP10	2	-0.11	0.03	3.26E-04
MMP12	2	-0.01	0.02	6.94E-01
MMP13	1	0.01	0.08	8.70E-01
MMP3	4	0.02	0.02	3.51E-01
MMP7	5	-0.03	0.03	3.92E-01
MMP8	2	0.00	0.02	9.98E-01
MMP9	9	-0.07	0.04	8.13E-02

MNDA	1	-0.75	0.20	1.32E-04
MOG	2	0.06	0.04	2.02E-01
MPHOSPH8	2	-0.06	0.10	5.42E-01
MPI	4	-0.01	0.04	8.70E-01
MPIG6B	5	0.01	0.06	9.23E-01
MPO	7	-0.11	0.04	4.48E-03
MSLN	8	-0.04	0.02	1.36E-02
MSMB	5	-0.01	0.01	2.52E-01
MSR1	12	-0.01	0.03	5.94E-01
MSRA	2	-0.04	0.12	7.12E-01
MSTN	5	0.10	0.08	2.07E-01
MUC13	4	-0.16	0.03	5.88E-07
MUC16	3	0.00	0.05	9.33E-01
MYO9B	1	0.31	0.26	2.20E-01
MYOC	10	0.05	0.04	1.84E-01
MZB1	7	0.02	0.05	7.30E-01
MZT1	2	-0.23	0.12	6.07E-02
NAAA	15	0.00	0.01	9.39E-01
NADK	5	-0.01	0.03	6.63E-01
NBL1	3	0.05	0.08	4.97E-01
NBN	3	0.03	0.10	7.32E-01
NCAM1	7	-0.05	0.03	8.88E-02
NCAM2	5	0.04	0.04	3.78E-01
NCAN	12	-0.05	0.04	2.39E-01
NCF2	4	-0.04	0.05	4.31E-01
NCK2	1	0.04	0.24	8.55E-01
NCR1	22	-0.05	0.02	6.01E-02
NCS1	8	-0.06	0.06	2.94E-01
NECTIN2	4	0.09	0.04	1.74E-02
NECTIN4	6	0.05	0.04	1.51E-01
NEFL	2	-0.28	0.16	6.99E-02
NELL1	18	0.02	0.02	2.46E-01
NELL2	14	-0.17	0.04	1.29E-05
NFASC	5	-0.05	0.03	3.71E-02
NFATC1	2	0.06	0.08	4.53E-01
NFATC3	1	0.14	0.18	4.26E-01
NFKBIE	2	0.05	0.05	3.48E-01
NID1	10	0.01	0.03	6.85E-01
NID2	15	0.02	0.02	3.31E-01
NME3	13	-0.08	0.03	3.51E-03
NMNAT1	4	-0.05	0.04	2.42E-01
NOMO1	3	0.05	0.07	5.24E-01
NOS1	2	-0.03	0.10	7.61E-01
NOS3	2	-0.29	0.12	1.20E-02
NOTCH1	6	0.04	0.03	2.09E-01
NOTCH3	10	-0.10	0.05	4.40E-02
NPDC1	5	-0.10	0.08	2.19E-01
NPPB	2	0.02	0.05	6.50E-01
NPPC	2	0.08	0.15	5.91E-01
NPTX1	14	-0.04	0.02	2.77E-02
NPTXR	4	0.00	0.02	8.80E-01
NPY	12	-0.06	0.03	5.45E-02
NRCAM	7	-0.26	0.06	4.28E-06
NRP1	6	-0.03	0.03	3.14E-01
NRP2	9	0.11	0.04	6.76E-03
NRTN	1	0.23	0.20	2.50E-01
NSFL1C	2	0.09	0.10	3.64E-01
NT5C3A	4	0.11	0.07	1.21E-01
NT5E	10	-0.01	0.02	5.02E-01
NTF3	4	0.02	0.05	6.42E-01
NTF4	1	-0.10	0.19	5.89E-01

NTproBNP	4	0.03	0.04	3.94E-01
NTRK2	7	0.11	0.07	9.97E-02
NTRK3	10	-0.02	0.04	5.17E-01
NUB1	4	-0.04	0.05	4.09E-01
NUCB2	5	0.02	0.02	4.94E-01
NUDC	2	-0.03	0.10	7.73E-01
NUDT2	2	0.04	0.04	2.65E-01
NUDT5	2	-0.15	0.11	1.86E-01
OBP2B	12	-0.01	0.02	4.69E-01
ODAM	2	-0.03	0.04	4.09E-01
OGFR	2	0.10	0.10	3.12E-01
OGN	4	0.01	0.02	5.28E-01
OLR1	6	-0.03	0.06	6.32E-01
OMD	6	-0.08	0.05	1.32E-01
OMG	8	-0.04	0.05	5.12E-01
OPTC	7	0.08	0.05	9.96E-02
OSCAR	9	0.01	0.02	6.70E-01
OSM	8	-0.12	0.04	3.80E-03
OSMR	5	0.02	0.02	3.57E-01
OXT	10	0.00	0.02	8.48E-01
P4HB	4	0.07	0.09	4.47E-01
PADI2	1	-0.08	0.08	3.41E-01
PADI4	1	0.03	0.04	3.55E-01
PAEP	3	-0.01	0.01	5.81E-01
PAG1	6	-0.01	0.06	8.10E-01
PAK4	1	-0.05	0.19	8.04E-01
PAM	4	-0.01	0.02	8.08E-01
PAMR1	4	-0.02	0.03	3.56E-01
PAPPA	11	0.04	0.03	2.63E-01
PARK7	5	-0.02	0.06	7.52E-01
PARP1	2	-0.01	0.06	8.36E-01
PBLD	1	0.02	0.02	2.61E-01
PCDH1	5	-0.13	0.07	7.20E-02
PCDH17	8	0.03	0.02	1.24E-01
PCOLCE	1	0.13	0.07	6.92E-02
PCSK9	9	0.00	0.03	9.56E-01
PDCD1	12	-0.05	0.05	2.80E-01
PDCD1LG2	8	-0.04	0.01	2.20E-03
PDCD5	2	-0.03	0.03	3.10E-01
PDCD6	4	-0.02	0.01	5.18E-02
PDGFA	17	0.03	0.03	2.59E-01
PDGFB	17	0.02	0.03	5.37E-01
PDGFC	7	-0.04	0.03	2.31E-01
PDGFRA	9	0.02	0.03	4.24E-01
PDGFRB	2	-0.01	0.01	4.10E-01
PDLIM7	3	0.04	0.09	6.85E-01
PEAR1	9	0.04	0.03	1.34E-01
PEBP1	2	-0.10	0.12	4.41E-01
PECAM1	8	-0.14	0.02	1.46E-13
PFKFB2	4	-0.02	0.05	7.45E-01
PGF	3	-0.04	0.09	6.58E-01
PGLYRP1	12	-0.01	0.03	7.39E-01
PI3	4	0.01	0.03	7.27E-01
PIGR	6	0.12	0.04	4.15E-03
PIK3AP1	7	-0.08	0.04	4.53E-02
PIK3IP1	3	-0.04	0.03	1.80E-01
PILRA	6	0.00	0.01	8.82E-01
PILRB	4	0.00	0.01	9.06E-01
PKLR	4	-0.19	0.05	1.53E-04
PLA2G10	15	0.04	0.02	6.13E-02
PLA2G15	9	0.01	0.03	6.11E-01

PLA2G1B	18	-0.08	0.03	1.37E-02
PLA2G2A	6	-0.03	0.02	1.20E-01
PLA2G4A	3	-0.17	0.10	1.00E-01
PLA2G7	10	-0.01	0.03	8.17E-01
PLAT	4	-0.18	0.08	2.05E-02
PLAU	10	0.02	0.03	4.83E-01
PLAUR	11	0.01	0.04	7.83E-01
PLIN1	1	-0.09	0.19	6.36E-01
PLIN3	2	0.18	0.11	1.21E-01
PLPBP	2	-0.04	0.11	7.46E-01
PLTP	11	0.01	0.02	5.30E-01
PLXDC1	5	-0.05	0.04	1.62E-01
PLXNA4	5	-0.01	0.05	8.05E-01
PLXNB2	11	-0.02	0.01	1.06E-01
PLXNB3	5	0.07	0.08	3.99E-01
PM20D1	8	-0.01	0.02	4.93E-01
PMVK	6	-0.07	0.06	2.92E-01
PNLIPRP2	7	-0.02	0.01	4.54E-02
PNPT1	2	0.09	0.12	4.26E-01
PODXL	6	-0.13	0.02	2.84E-13
PODXL2	12	0.02	0.04	6.84E-01
POLR2F	1	-0.25	0.18	1.68E-01
PON2	3	-0.02	0.01	2.56E-01
PON3	9	0.04	0.03	1.60E-01
PPCDC	2	0.01	0.03	7.58E-01
PPIB	3	-0.05	0.10	6.00E-01
PPME1	1	0.04	0.19	8.22E-01
PPP1R12A	4	0.06	0.08	4.62E-01
PPP1R2	3	-0.04	0.09	6.61E-01
PPP1R9B	2	-0.03	0.12	7.72E-01
PPP3R1	5	-0.11	0.07	1.19E-01
PPY	8	-0.04	0.04	3.44E-01
PRCP	12	0.02	0.03	5.46E-01
PRDX1	2	-0.07	0.12	5.32E-01
PRDX3	1	-0.14	0.15	3.81E-01
PRDX5	2	-0.09	0.09	3.00E-01
PRDX6	1	0.05	0.07	4.75E-01
PRELP	1	-0.01	0.02	7.83E-01
PRKAB1	2	0.01	0.15	9.57E-01
PRKAR1A	5	-0.01	0.05	7.58E-01
PRKRA	1	-0.02	0.10	8.04E-01
PRL	3	0.03	0.09	7.71E-01
PROC	8	0.00	0.04	9.02E-01
PROK1	2	0.03	0.03	3.00E-01
PRSS2	13	-0.01	0.04	6.74E-01
PRSS27	18	-0.08	0.01	2.89E-08
PRSS8	8	-0.04	0.05	3.83E-01
PRTFDC1	3	-0.02	0.06	7.87E-01
PRTG	9	0.05	0.02	2.26E-02
PRTN3	15	0.00	0.03	9.05E-01
PSG1	7	-0.02	0.02	4.07E-01
PSIP1	3	-0.10	0.07	1.18E-01
PSME1	3	-0.08	0.09	3.81E-01
PSME2	1	-0.06	0.12	6.05E-01
PSMG3	1	0.33	0.26	2.08E-01
PSPN	10	0.04	0.07	5.30E-01
PSRC1	2	-0.07	0.06	2.45E-01
PTGDS	2	0.20	0.13	1.29E-01
PTH1R	1	0.13	0.06	4.09E-02
PTN	4	-0.02	0.06	6.76E-01
PTPN1	3	-0.13	0.08	1.05E-01

PTPN6	2	-0.03	0.10	7.85E-01
PTPRF	14	0.01	0.03	8.78E-01
PTPRM	8	-0.12	0.02	2.93E-14
PTPRN2	1	0.07	0.06	2.59E-01
PTPRS	5	0.09	0.03	8.13E-03
PTS	4	-0.02	0.05	6.69E-01
PTX3	5	0.03	0.03	3.50E-01
PVALB	8	-0.02	0.01	1.16E-01
PVR	2	0.01	0.01	6.06E-01
PXN	3	-0.01	0.01	3.46E-01
QDPR	1	-0.04	0.16	8.04E-01
QPCT	11	-0.02	0.03	5.74E-01
RAB6A	3	0.03	0.02	1.90E-01
RABEPK	1	-0.01	0.07	9.37E-01
RABGAP1L	2	0.10	0.11	3.54E-01
RAD23B	3	0.06	0.10	5.27E-01
RARRES1	1	0.01	0.02	5.55E-01
RARRES2	3	0.02	0.05	7.27E-01
RASSF2	1	-0.21	0.13	1.11E-01
RBKS	1	-0.05	0.03	1.40E-01
RBP2	4	0.16	0.07	3.01E-02
RBP5	3	0.03	0.03	4.24E-01
RCOR1	1	0.08	0.17	6.25E-01
REG1A	5	0.22	0.03	9.72E-18
REG1B	7	0.18	0.02	9.56E-15
REG3A	9	0.05	0.04	1.96E-01
REG4	1	0.06	0.06	3.60E-01
RELT	4	0.01	0.03	8.49E-01
REN	2	-0.07	0.07	3.34E-01
RET	8	0.05	0.02	2.73E-02
RETN	15	0.04	0.03	1.84E-01
RGMA	17	-0.03	0.02	2.03E-01
RGMB	4	-0.04	0.05	4.26E-01
RGS8	1	0.01	0.07	9.11E-01
RHOC	3	-0.06	0.09	5.35E-01
RILP	4	0.03	0.08	6.80E-01
RNASE3	4	0.00	0.04	9.63E-01
RNASET2	9	0.04	0.02	1.21E-01
ROBO1	9	-0.04	0.04	2.52E-01
ROBO2	5	0.03	0.07	6.40E-01
ROR1	12	0.02	0.03	4.93E-01
RP2	2	-0.17	0.10	9.57E-02
RRM2	1	-0.13	0.17	4.67E-01
RRM2B	2	-0.02	0.08	8.29E-01
RSPO1	10	0.00	0.03	9.30E-01
RSPO3	8	0.03	0.04	5.42E-01
RTBDN	3	0.03	0.06	6.63E-01
RTN4R	10	-0.05	0.03	1.06E-01
RWDD1	2	0.02	0.07	7.88E-01
S100A11	2	0.04	0.05	3.67E-01
S100A12	4	0.02	0.04	5.77E-01
S100A16	3	0.16	0.07	2.79E-02
S100A4	5	-0.01	0.06	8.35E-01
S100P	5	-0.17	0.06	8.01E-03
SAMD9L	2	-0.03	0.03	3.62E-01
SCAMP3	4	-0.07	0.07	2.93E-01
SCARA5	7	0.10	0.04	2.94E-03
SCARB1	1	-0.03	0.26	9.19E-01
SCARB2	4	0.01	0.04	7.34E-01
SCARF1	7	0.01	0.02	6.43E-01
SCARF2	8	-0.08	0.03	5.58E-03

SCG2	4	-0.52	0.08	1.19E-10
SCG3	5	-0.02	0.02	1.53E-01
SCGB1A1	17	0.05	0.03	7.71E-02
SCGB3A2	17	0.00	0.03	9.47E-01
SCGN	5	0.21	0.05	1.02E-05
SCLY	7	0.02	0.05	6.52E-01
SCP2	1	0.36	0.20	6.84E-02
SCRN1	2	0.04	0.07	6.02E-01
SDC1	13	0.02	0.03	4.92E-01
SDC4	20	0.04	0.03	1.16E-01
SELE	9	-0.09	0.01	2.32E-13
SELP	10	-0.08	0.03	2.81E-03
SELPLG	10	-0.01	0.02	3.90E-01
SEMA3F	5	-0.34	0.07	1.47E-06
SEMA4C	5	-0.31	0.04	1.60E-13
SEMA4D	6	-0.03	0.06	5.88E-01
SEMA7A	18	0.01	0.03	6.37E-01
SEPTIN9	2	0.21	0.11	6.50E-02
SERPINA11	8	0.01	0.02	6.73E-01
SERPINA12	5	0.02	0.02	2.64E-01
SERPINA9	3	0.00	0.02	9.90E-01
SERPINB1	3	0.14	0.08	8.45E-02
SERPINB6	2	0.04	0.07	5.47E-01
SERPINB8	2	-0.02	0.02	3.17E-01
SERPINB9	2	-0.13	0.08	9.52E-02
SERPINE1	17	0.02	0.03	4.60E-01
SESTD1	3	0.00	0.08	9.92E-01
SETMAR	1	-0.02	0.05	7.46E-01
SEZ6L	6	-0.01	0.05	7.81E-01
SEZ6L2	8	0.05	0.04	2.61E-01
SF3B4	4	0.03	0.08	7.22E-01
SFRP1	5	0.00	0.05	9.88E-01
SFTPA1	1	-0.14	0.05	3.15E-03
SFTPA2	6	0.07	0.02	2.65E-05
SFTPD	11	-0.07	0.01	8.06E-09
SH2B3	4	0.08	0.08	3.00E-01
SH2D1A	2	-0.12	0.08	1.49E-01
SHMT1	4	-0.02	0.02	1.41E-01
SIAE	9	0.02	0.04	6.56E-01
SIGLEC1	10	-0.07	0.04	4.44E-02
SIGLEC10	8	-0.01	0.02	7.51E-01
SIGLEC5	3	0.02	0.01	1.64E-01
SIGLEC6	21	-0.02	0.02	3.43E-01
SIGLEC7	9	0.05	0.03	1.13E-01
SIGLEC9	10	-0.02	0.01	2.82E-02
SIRPA	7	0.01	0.01	6.10E-01
SIRPB1	11	-0.01	0.01	3.25E-01
SIRT2	3	0.05	0.07	4.75E-01
SIT1	6	-0.03	0.05	5.99E-01
SKAP1	8	0.00	0.06	9.51E-01
SKAP2	3	0.04	0.10	6.62E-01
SLAMF1	5	0.06	0.06	3.08E-01
SLAMF6	3	-0.01	0.06	8.75E-01
SLAMF7	9	0.04	0.02	1.49E-02
SLAMF8	5	0.01	0.01	6.63E-01
SLC16A1	4	-0.12	0.06	5.04E-02
SLC27A4	3	-0.06	0.08	3.96E-01
SLC39A14	2	0.00	0.09	9.58E-01
SLC39A5	8	0.09	0.05	8.98E-02
SLIT2	1	0.17	0.18	3.64E-01
SLITRK2	11	0.16	0.04	1.89E-04

SLITRK6	8	0.03	0.02	2.96E-01
SMAD1	5	-0.02	0.08	7.51E-01
SMAD5	1	0.05	0.06	3.36E-01
SMOC1	10	-0.01	0.03	6.93E-01
SMOC2	5	-0.01	0.03	7.01E-01
SMPD1	15	-0.01	0.02	7.76E-01
SMPDL3A	20	0.01	0.01	4.69E-01
SNAP23	3	-0.02	0.07	7.49E-01
SNAP29	5	0.06	0.07	3.88E-01
SNCG	1	-0.01	0.01	3.12E-01
SNX9	2	-0.04	0.09	6.80E-01
SOD1	3	-0.01	0.08	9.36E-01
SOD2	8	-0.03	0.04	4.67E-01
SORCS2	4	0.03	0.02	2.61E-01
SORD	4	-0.06	0.06	3.47E-01
SORT1	14	-0.04	0.03	2.29E-01
SOST	14	0.07	0.03	2.60E-02
SPARC	14	0.02	0.04	6.35E-01
SPARCL1	4	0.00	0.01	9.74E-01
SPINK1	2	0.01	0.03	8.04E-01
SPINK4	5	0.00	0.01	9.09E-01
SPINK5	7	-0.06	0.02	9.58E-03
SPINK6	1	0.00	0.03	9.69E-01
SPINT1	8	-0.03	0.03	2.97E-01
SPINT2	15	0.00	0.03	9.96E-01
SPOCK1	12	-0.02	0.04	5.72E-01
SPON1	6	0.03	0.03	3.32E-01
SPON2	1	0.00	0.05	9.35E-01
SPP1	7	-0.11	0.07	8.34E-02
SPRY2	6	-0.01	0.05	8.00E-01
SRC	1	0.53	0.18	3.68E-03
SRP14	2	-0.06	0.08	4.37E-01
SRPK2	3	-0.03	0.07	6.77E-01
SSC4D	11	0.03	0.02	2.22E-01
SSC5D	10	0.11	0.04	3.97E-03
ST3GAL1	7	-0.01	0.03	7.08E-01
ST6GAL1	8	0.06	0.05	2.00E-01
STAMBP	2	-0.10	0.12	4.30E-01
STAT5B	3	-0.03	0.09	7.24E-01
STC1	5	-0.23	0.06	1.05E-04
STC2	5	0.02	0.08	7.68E-01
STIP1	2	0.00	0.10	9.65E-01
STK11	4	-0.05	0.08	5.68E-01
STK24	2	-0.04	0.13	7.47E-01
STK4	4	0.03	0.08	7.48E-01
STX16	1	-0.14	0.19	4.49E-01
STX4	2	-0.21	0.13	1.20E-01
STX6	2	-0.12	0.14	4.20E-01
STX8	3	0.04	0.09	6.38E-01
SUGT1	1	-0.04	0.15	8.04E-01
SULT1A1	1	-0.13	0.11	2.45E-01
SULT2A1	4	-0.04	0.03	2.89E-01
SUMF2	14	-0.02	0.02	4.69E-01
SUSD1	4	0.00	0.06	9.76E-01
SUSD2	4	0.02	0.02	3.30E-01
TACC3	4	0.10	0.07	1.40E-01
TACSTD2	14	-0.03	0.01	2.25E-02
TAFA5	2	0.02	0.06	7.75E-01
TARBP2	3	-0.04	0.09	6.71E-01
TBC1D17	1	0.01	0.02	6.81E-01
TBC1D23	4	-0.03	0.05	6.19E-01

TBC1D5	2	-0.10	0.12	3.96E-01
TBCB	4	-0.06	0.08	4.94E-01
TBCC	4	-0.04	0.06	4.90E-01
TBL1X	5	0.02	0.06	6.81E-01
TCL1A	21	-0.03	0.03	2.30E-01
TCN2	2	0.00	0.02	8.64E-01
TDGF1	1	-1.83	0.22	3.62E-16
TDRKH	4	-0.04	0.03	1.27E-01
TEK	9	0.05	0.02	4.19E-03
TFF1	5	-0.01	0.03	6.76E-01
TFF2	8	0.10	0.04	1.36E-02
TFF3	3	0.01	0.03	7.95E-01
TFPI	8	-0.03	0.02	2.63E-01
TFPI2	9	-0.11	0.04	8.45E-03
TFRC	11	-0.05	0.02	2.83E-03
TGFA	4	-0.17	0.06	3.20E-03
TGFB1	10	-0.08	0.03	2.02E-02
TGFBI	10	0.01	0.02	6.00E-01
TGFBR2	8	-0.30	0.05	8.27E-10
TGFBR3	7	-0.05	0.04	2.46E-01
TGM2	2	0.02	0.06	7.16E-01
THBD	14	0.03	0.03	2.79E-01
THBS2	7	-0.03	0.02	1.86E-01
THBS4	6	0.12	0.03	3.57E-04
THOP1	1	-0.04	0.05	4.43E-01
THPO	17	0.02	0.03	5.39E-01
THY1	3	0.00	0.03	8.74E-01
TIA1	2	0.12	0.10	2.05E-01
TIE1	12	0.02	0.02	1.46E-01
TIGAR	2	-0.03	0.13	8.14E-01
TIMD4	25	-0.02	0.02	3.77E-01
TIMP1	5	0.13	0.05	1.86E-02
TIMP3	17	0.01	0.02	3.36E-01
TIMP4	4	-0.10	0.03	6.18E-04
TINAGL1	9	0.02	0.05	6.99E-01
TJAP1	2	-0.02	0.14	8.60E-01
TLR3	6	0.00	0.01	8.51E-01
TMPRSS15	7	-0.06	0.03	4.02E-02
TMPRSS5	18	0.01	0.01	3.54E-01
TMSB10	6	-0.05	0.06	4.26E-01
TNC	15	0.03	0.02	1.47E-01
TNF	2	-0.06	0.06	2.91E-01
TNFAIP8	1	0.00	0.13	9.82E-01
TNFRSF10A	4	-0.01	0.02	7.30E-01
TNFRSF10B	3	0.01	0.03	6.73E-01
TNFRSF10C	16	0.01	0.01	2.50E-01
TNFRSF11A	6	0.08	0.02	3.61E-04
TNFRSF11B	9	-0.04	0.04	3.00E-01
TNFRSF12A	2	-0.20	0.13	1.18E-01
TNFRSF13B	11	-0.05	0.04	1.59E-01
TNFRSF13C	12	0.01	0.05	8.86E-01
TNFRSF14	5	-0.05	0.06	4.02E-01
TNFRSF19	4	-0.03	0.04	4.16E-01
TNFRSF1A	7	0.20	0.06	7.94E-04
TNFRSF1B	6	-0.10	0.05	2.72E-02
TNFRSF21	12	0.04	0.03	2.45E-01
TNFRSF4	6	-0.02	0.05	6.53E-01
TNFRSF6B	3	-0.02	0.03	6.39E-01
TNFRSF8	8	-0.06	0.04	8.82E-02
TNFRSF9	6	0.04	0.06	5.02E-01
TNFSF10	12	0.01	0.02	5.28E-01

TNFSF11	8	-0.02	0.03	4.24E-01
TNFSF12	16	0.04	0.02	7.09E-02
TNFSF13	7	-0.05	0.03	6.69E-02
TNFSF13B	6	-0.07	0.06	2.39E-01
TNFSF14	5	0.00	0.03	9.02E-01
TNR	16	0.03	0.02	1.79E-01
TNXB	6	0.06	0.02	1.42E-03
TP53	1	-0.10	0.17	5.40E-01
TP53INP1	1	0.08	0.09	3.66E-01
TPMT	2	-0.02	0.02	4.10E-01
TPP1	12	-0.01	0.03	8.35E-01
TPPP3	1	-0.15	0.15	3.10E-01
TPSAB1	13	0.01	0.01	4.72E-01
TRAF2	3	-0.11	0.10	2.82E-01
TREM2	7	0.03	0.02	1.41E-01
TREML2	8	-0.05	0.04	1.50E-01
TRIAP1	6	-0.10	0.05	6.38E-02
TRIM21	3	-0.18	0.11	1.05E-01
TRIM5	1	0.01	0.03	8.09E-01
TSHB	17	-0.05	0.03	1.35E-01
TSPAN1	3	0.05	0.12	7.15E-01
TST	1	-0.18	0.11	9.17E-02
TXLNA	3	-0.03	0.07	7.22E-01
TXNDC15	11	0.01	0.01	4.04E-01
TXNDC5	4	0.05	0.07	4.70E-01
TXNRD1	4	-0.04	0.08	5.91E-01
TYMP	2	-0.08	0.05	8.94E-02
TYRO3	8	0.03	0.01	4.87E-02
UBAC1	1	0.24	0.21	2.51E-01
ULBP2	7	0.05	0.02	2.07E-02
UMOD	16	0.00	0.01	7.71E-01
USO1	3	-0.03	0.09	7.23E-01
USP8	4	0.03	0.06	6.20E-01
UXS1	7	-0.07	0.03	3.57E-02
VAMP5	2	0.77	0.10	2.34E-13
VASH1	4	0.05	0.06	4.35E-01
VASN	5	-0.05	0.02	4.31E-02
VAT1	9	-0.06	0.04	1.47E-01
VCAM1	10	-0.05	0.04	1.80E-01
VCAN	15	0.01	0.01	2.95E-01
VEGFA	13	0.01	0.01	7.15E-01
VEGFC	16	0.06	0.03	7.06E-02
VEGFD	6	0.03	0.05	5.37E-01
VMO1	7	-0.01	0.01	4.27E-01
VNN2	10	-0.01	0.01	6.21E-01
VPS37A	2	-0.02	0.13	8.71E-01
VPS53	2	-0.04	0.12	7.68E-01
VSIG4	8	0.11	0.06	6.73E-02
VSIR	8	0.04	0.03	1.84E-01
VSTM1	2	0.01	0.01	3.83E-01
VSTM2L	2	-0.07	0.05	1.70E-01
VTA1	3	0.01	0.09	9.21E-01
VTCN1	2	-0.42	0.10	1.46E-05
VWA1	7	-0.02	0.04	5.83E-01
VWC2	3	0.02	0.02	4.19E-01
VWF	6	0.26	0.03	2.05E-18
WARS	3	-0.06	0.04	1.65E-01
WAS	3	0.08	0.08	2.93E-01
WASF1	3	0.02	0.03	5.24E-01
WASF3	1	0.04	0.07	6.23E-01
WFDC12	12	-0.02	0.02	4.15E-01

WFDC2	3	-0.16	0.08	6.26E-02
WFIKKN1	14	-0.03	0.03	3.50E-01
WFIKKN2	4	0.00	0.02	9.26E-01
WIF1	13	-0.02	0.03	5.81E-01
WNT9A	1	-0.15	0.11	1.44E-01
WWP2	3	-0.10	0.05	5.01E-02
XCL1	10	0.00	0.01	8.33E-01
XG	1	-0.29	0.18	1.03E-01
XPNPEP2	12	0.00	0.04	9.44E-01
XRCC4	1	0.13	0.15	3.97E-01
YES1	2	-0.03	0.10	7.51E-01
YTHDF3	1	-0.03	0.11	8.04E-01
ZBTB16	7	0.01	0.04	8.85E-01
ZBTB17	2	-0.07	0.11	5.10E-01

Supplementary Table 4. Associations of genetically predicted proteins with COVID-19 infection using IVW method and cis+trans SNPs.

Protein	No. of SNPs	Beta	SE	Pval
AARSD1	3	0.02	0.03	4.52E-01
ABHD14B	4	-0.02	0.02	4.50E-01
ABL1	1	-0.01	0.06	8.82E-01
ACAA1	3	0.02	0.04	5.58E-01
ACAN	15	0.04	0.02	2.77E-02
ACE2	9	-0.05	0.02	4.47E-02
ACOX1	1	-0.07	0.07	3.05E-01
ACP5	10	0.01	0.01	3.53E-01
ACP6	2	-0.01	0.01	2.61E-01
ACTA2	1	0.08	0.09	3.65E-01
ACVRL1	4	-0.01	0.01	6.30E-01
ACY1	3	-0.01	0.02	7.79E-01
ADA	4	-0.02	0.01	6.27E-03
ADA2	13	-0.02	0.01	1.35E-01
ADAM15	8	0.03	0.01	3.16E-09
ADAM22	6	0.00	0.01	7.15E-01
ADAM23	5	0.02	0.01	7.62E-02
ADAM8	7	-0.01	0.01	2.96E-01
ADAMTS13	13	0.01	0.01	3.75E-01
ADAMTS15	5	0.05	0.03	6.23E-02
ADAMTS16	1	-0.04	0.04	2.77E-01
ADAMTS8	4	0.00	0.01	6.61E-01
ADGRB3	5	0.01	0.01	1.89E-01
ADGRE2	9	-0.01	0.01	4.96E-01
ADGRE5	10	-0.02	0.02	1.17E-01
ADGRG1	10	-0.14	0.02	1.91E-12
ADGRG2	6	0.24	0.03	2.38E-14
ADH4	6	-0.02	0.02	4.03E-01
ADM	2	0.00	0.04	9.82E-01
AFP	23	0.00	0.01	9.90E-01
AGER	15	0.01	0.02	5.34E-01
AGR2	5	0.08	0.03	1.30E-02
AGR3	3	-0.01	0.05	8.98E-01
AGRN	6	0.01	0.01	3.88E-01
AGRP	8	-0.01	0.03	8.23E-01
AGXT	11	0.00	0.01	9.61E-01
AHCY	1	0.00	0.01	8.07E-01
AHSP	9	0.01	0.02	6.93E-01
AIF1	1	0.02	0.04	6.30E-01
AIFM1	2	0.02	0.06	6.95E-01
AKR1B1	2	-0.01	0.03	6.78E-01
AKR1C4	1	0.07	0.09	4.41E-01
AKT1S1	3	-0.01	0.04	8.99E-01
AKT3	2	-0.13	0.06	3.87E-02
ALCAM	11	-0.02	0.02	1.86E-01
ALDH1A1	3	-0.01	0.04	7.23E-01
ALDH3A1	1	-0.02	0.01	2.92E-02
ALPP	11	0.01	0.01	3.08E-01
AMBP	9	-0.01	0.02	6.43E-01
AMIGO2	12	-0.07	0.01	1.37E-07
AMN	5	-0.01	0.01	4.28E-01
AMY2A	8	-0.10	0.01	9.69E-15
AMY2B	9	-0.07	0.01	3.08E-12
ANG	3	0.03	0.01	8.59E-03
ANGPT1	20	-0.02	0.01	2.13E-01
ANGPT2	6	0.03	0.02	1.15E-01
ANGPTL1	6	0.03	0.01	4.30E-02
ANGPTL2	6	0.03	0.02	2.20E-01

ANGPTL3	6	-0.02	0.03	4.03E-01
ANGPTL4	1	-0.02	0.02	3.19E-01
ANGPTL7	6	0.01	0.01	4.06E-01
ANKRD54	1	0.11	0.08	1.67E-01
ANPEP	7	0.03	0.01	2.23E-02
ANXA11	3	-0.06	0.05	1.92E-01
ANXA3	5	-0.06	0.04	7.03E-02
ANXA4	3	-0.07	0.05	1.78E-01
ANXA5	1	0.05	0.04	2.72E-01
AOC1	1	0.00	0.02	8.60E-01
AOC3	10	0.00	0.01	9.55E-01
APBB1IP	5	-0.03	0.02	1.06E-01
APEX1	3	0.02	0.01	1.95E-01
APLP1	5	0.06	0.03	3.22E-02
APOH	1	0.00	0.01	9.96E-01
APOM	9	0.02	0.01	7.80E-02
APP	18	-0.02	0.02	3.23E-01
APRT	6	-0.04	0.03	1.19E-01
AREG	3	0.11	0.03	6.34E-06
ARG1	5	-0.01	0.02	5.19E-01
ARHGAP1	2	-0.09	0.04	4.05E-02
ARHGAP25	2	-0.02	0.04	5.89E-01
ARSA	7	0.01	0.01	1.61E-01
ARSB	11	-0.01	0.01	3.54E-01
ART3	4	0.01	0.04	8.78E-01
ARTN	1	0.04	0.11	7.14E-01
ASAH2	16	-0.01	0.01	1.47E-01
ASGR1	7	-0.02	0.02	3.85E-01
ATG4A	3	0.04	0.05	3.43E-01
ATOX1	4	0.03	0.04	4.47E-01
ATP5IF1	6	0.01	0.03	6.94E-01
ATP6V1F	1	-0.01	0.07	8.82E-01
ATXN10	5	-0.02	0.03	5.22E-01
AXIN1	3	-0.08	0.05	1.27E-01
AXL	9	-0.02	0.02	3.24E-01
AZU1	3	-0.08	0.05	1.17E-01
B4GALT1	7	0.03	0.01	2.22E-02
B4GAT1	9	0.02	0.01	1.99E-01
BACH1	3	-0.01	0.05	8.11E-01
BAG3	4	0.07	0.03	3.80E-02
BAIAP2	6	0.00	0.03	9.13E-01
BAMBI	1	0.01	0.07	8.82E-01
BANK1	3	-0.05	0.03	1.72E-01
BAX	2	-0.07	0.06	2.44E-01
BCAM	15	0.01	0.01	2.06E-01
BCAN	7	0.02	0.02	2.48E-01
BCL2L11	10	0.02	0.01	1.29E-01
BCR	1	-0.07	0.09	3.97E-01
BGN	11	0.05	0.02	7.55E-03
BID	2	0.03	0.05	5.07E-01
BIN2	3	-0.04	0.04	3.52E-01
BIRC2	4	-0.01	0.05	9.10E-01
BLMH	3	0.00	0.01	7.19E-01
BLVRB	2	0.00	0.02	8.86E-01
BMP4	6	0.01	0.03	7.47E-01
BMP6	16	-0.04	0.01	3.84E-03
BOC	8	0.04	0.02	2.55E-02
BPIFB1	15	-0.01	0.01	1.32E-01
BRK1	8	0.01	0.02	7.00E-01
BSG	10	-0.14	0.02	5.50E-13

BST1	4	-0.01	0.00	2.51E-01
BST2	11	0.11	0.02	3.25E-09
BTC	5	0.00	0.01	7.70E-01
BTN2A1	7	-0.01	0.02	4.16E-01
BTN3A2	3	-0.01	0.01	1.52E-01
C19orf12	6	-0.05	0.03	7.30E-02
C1QA	13	0.00	0.01	7.01E-01
C1QTNF1	4	0.01	0.02	7.43E-01
C2	2	0.04	0.04	3.47E-01
C2CD2L	1	-0.12	0.09	1.81E-01
C4BPB	11	0.00	0.01	8.23E-01
CA1	4	-0.03	0.03	2.17E-01
CA11	2	0.00	0.03	9.13E-01
CA12	7	0.03	0.01	4.72E-02
CA13	3	-0.01	0.02	6.35E-01
CA14	5	0.03	0.02	1.47E-01
CA2	2	0.01	0.04	7.42E-01
CA3	6	0.00	0.02	8.24E-01
CA4	11	0.09	0.01	1.18E-14
CA5A	5	0.01	0.01	4.56E-01
CA6	12	-0.02	0.01	2.05E-03
CA9	5	0.07	0.02	2.60E-03
CALB1	3	-0.01	0.03	6.72E-01
CALCA	7	0.01	0.03	6.18E-01
CALCOCO1	4	0.00	0.03	9.75E-01
CAMKK1	3	0.02	0.02	3.00E-01
CANT1	7	0.04	0.02	1.22E-01
CAPG	2	-0.01	0.01	2.49E-01
CARHSP1	1	0.01	0.06	9.10E-01
CASP1	1	0.00	0.02	8.94E-01
CASP10	3	-0.07	0.02	5.18E-03
CASP2	2	-0.01	0.05	9.02E-01
CASP3	2	0.05	0.04	2.09E-01
CASP8	4	-0.03	0.02	1.81E-01
CBLIF	17	-0.06	0.01	1.25E-09
CBLN4	5	0.04	0.02	4.60E-02
CC2D1A	2	-0.04	0.06	4.79E-01
CCDC80	5	0.07	0.02	1.33E-03
CCL11	10	-0.01	0.02	5.92E-01
CCL13	15	0.01	0.01	2.23E-01
CCL14	6	0.02	0.01	7.76E-02
CCL15	4	0.14	0.02	1.41E-09
CCL16	9	-0.01	0.01	3.38E-02
CCL17	14	0.01	0.01	3.56E-01
CCL18	4	0.00	0.01	7.71E-01
CCL19	5	-0.03	0.03	3.84E-01
CCL2	9	0.04	0.01	3.36E-03
CCL20	5	-0.06	0.03	2.42E-02
CCL21	11	-0.01	0.02	6.83E-01
CCL22	8	-0.02	0.02	4.22E-01
CCL23	9	-0.02	0.01	3.46E-02
CCL24	12	0.01	0.01	2.94E-02
CCL25	9	0.02	0.01	1.95E-04
CCL26	7	-0.01	0.02	5.36E-01
CCL27	7	-0.02	0.02	3.63E-01
CCL28	18	-0.09	0.01	1.12E-10
CCL3	7	0.03	0.02	8.44E-02
CCL4	5	-0.03	0.01	3.98E-03
CCL5	9	-0.03	0.02	4.50E-02
CCL7	5	-0.02	0.01	7.78E-02

CCL8	11	0.00	0.01	9.38E-01
CCN1	11	-0.02	0.02	2.25E-01
CCN2	18	-0.02	0.01	1.41E-01
CCN3	3	0.00	0.02	9.15E-01
CCN4	12	-0.02	0.01	3.03E-02
CCN5	1	0.06	0.10	5.64E-01
CCS	1	0.00	0.01	6.06E-01
CD109	6	-0.25	0.02	4.95E-34
CD14	6	-0.04	0.01	7.71E-03
CD160	14	-0.01	0.01	6.28E-01
CD163	18	0.01	0.01	2.27E-01
CD164	10	-0.01	0.02	5.22E-01
CD177	3	0.01	0.01	4.79E-01
CD1C	18	-0.01	0.01	5.24E-01
CD200	8	0.03	0.02	1.23E-01
CD200R1	10	0.00	0.01	9.76E-01
CD207	15	-0.01	0.01	2.65E-01
CD209	8	0.10	0.01	6.46E-73
CD22	20	-0.01	0.01	4.39E-01
CD244	14	-0.03	0.01	2.61E-02
CD27	7	0.00	0.02	7.70E-01
CD274	7	0.00	0.01	8.96E-01
CD276	7	0.00	0.01	9.73E-01
CD28	12	0.05	0.01	5.07E-04
CD2AP	1	-0.01	0.07	8.82E-01
CD300C	14	0.02	0.01	1.03E-01
CD300E	10	-0.02	0.01	1.66E-01
CD300LF	9	0.01	0.00	8.62E-02
CD300LG	12	0.03	0.01	9.17E-03
CD302	7	0.01	0.01	4.16E-01
CD33	7	0.00	0.00	5.99E-01
CD34	3	-0.16	0.01	6.89E-43
CD38	2	-0.03	0.01	3.10E-02
CD4	4	-0.01	0.01	3.50E-01
CD40	10	-0.01	0.01	1.93E-01
CD40LG	12	0.01	0.02	7.13E-01
CD46	7	-0.13	0.03	4.56E-07
CD48	13	-0.01	0.01	2.03E-01
CD5	8	-0.03	0.02	1.37E-01
CD55	6	0.02	0.01	4.20E-02
CD58	12	-0.11	0.01	2.68E-31
CD59	5	0.04	0.02	3.39E-02
CD6	11	-0.01	0.01	1.36E-01
CD63	10	-0.02	0.02	1.93E-01
CD69	7	-0.05	0.02	3.39E-02
CD70	4	0.00	0.01	5.46E-01
CD74	6	-0.03	0.02	9.59E-02
CD79B	17	-0.11	0.01	5.08E-16
CD83	14	-0.04	0.02	1.74E-02
CD84	11	0.00	0.02	9.35E-01
CD8A	5	0.01	0.01	5.27E-01
CD93	6	0.00	0.03	9.92E-01
CD99	4	0.03	0.03	3.06E-01
CD99L2	9	0.01	0.02	7.79E-01
CDC27	2	-0.03	0.05	5.63E-01
CDC37	3	0.04	0.05	4.62E-01
CDCP1	8	-0.03	0.02	4.52E-02
CDH1	6	-0.01	0.01	2.84E-01
CDH15	4	0.03	0.01	4.84E-02
CDH17	12	-0.05	0.01	1.11E-10

CDH2	13	-0.01	0.02	5.24E-01
CDH3	7	-0.05	0.03	4.75E-02
CDH5	12	0.02	0.01	3.64E-04
CDH6	6	0.00	0.01	7.34E-01
CDHR1	4	0.02	0.02	2.61E-01
CDHR2	8	0.06	0.03	2.68E-02
CDHR5	8	-0.02	0.01	1.68E-02
CDKN1A	6	0.01	0.02	5.48E-01
CDKN2D	5	0.02	0.03	5.29E-01
CDNF	1	0.00	0.01	7.75E-01
CDON	4	0.01	0.03	6.65E-01
CDSN	18	0.00	0.01	7.25E-01
CEACAM1	6	-0.01	0.02	3.38E-01
CEACAM21	2	-0.19	0.09	3.32E-02
CEACAM3	1	0.03	0.09	7.47E-01
CEACAM5	13	-0.02	0.01	7.01E-02
CEACAM8	12	0.00	0.02	8.97E-01
CEBPB	3	-0.06	0.05	2.09E-01
CELA3A	16	0.00	0.01	7.19E-01
CEP20	5	0.00	0.03	9.11E-01
CEP43	6	-0.03	0.03	4.08E-01
CEP85	3	0.01	0.03	7.78E-01
CERT	4	0.01	0.04	6.88E-01
CES1	18	0.01	0.01	3.93E-01
CES2	2	-0.07	0.04	1.33E-01
CES3	9	-0.01	0.02	5.62E-01
CETN2	3	0.01	0.04	7.74E-01
CFC1	7	0.00	0.03	9.74E-01
CGA	1	-0.07	0.12	5.32E-01
CGREF1	3	0.02	0.01	3.61E-02
CHAC2	2	0.02	0.04	6.35E-01
CHGB	6	-0.01	0.01	4.28E-01
CHI3L1	7	0.01	0.01	3.49E-01
CHIT1	5	0.00	0.01	5.72E-01
CHL1	6	-0.01	0.01	4.79E-01
CHMP1A	4	-0.08	0.04	2.98E-02
CHRDL1	4	-0.01	0.05	7.96E-01
CHRDL2	5	0.02	0.02	4.56E-01
CIAPIN1	4	-0.02	0.04	6.93E-01
CKAP4	4	-0.03	0.01	1.85E-02
CKMT1A_CKMT1B	8	0.14	0.02	6.18E-10
CLC	10	0.06	0.02	3.29E-03
CLEC10A	9	0.00	0.01	6.16E-01
CLEC11A	8	0.01	0.02	5.51E-01
CLEC14A	9	-0.15	0.01	6.02E-29
CLEC1A	7	0.02	0.01	1.87E-02
CLEC1B	9	0.00	0.02	7.85E-01
CLEC4A	12	0.01	0.01	1.84E-01
CLEC4C	25	0.00	0.01	9.89E-01
CLEC4D	10	0.00	0.01	5.62E-01
CLEC4G	12	0.15	0.01	4.94E-40
CLEC5A	11	0.01	0.01	4.41E-01
CLEC6A	13	0.00	0.01	6.86E-01
CLEC7A	4	0.00	0.01	7.17E-01
CLIP2	2	-0.01	0.03	7.52E-01
CLMP	4	0.02	0.01	1.52E-01
CLPS	7	-0.02	0.01	6.69E-03
CLSPN	1	0.02	0.05	6.24E-01
CLSTN2	4	0.04	0.01	6.82E-03
CLTA	1	0.00	0.04	9.55E-01

CLUL1	9	0.01	0.01	2.63E-01
CNDP1	8	-0.02	0.01	7.99E-02
CNPY2	1	-0.06	0.04	1.03E-01
CNPY4	3	0.01	0.02	7.52E-01
CNST	4	-0.07	0.05	1.11E-01
CNTN1	8	0.03	0.01	3.92E-02
CNTN2	6	0.02	0.01	2.12E-02
CNTN3	16	-0.01	0.01	3.51E-01
CNTN4	10	-0.01	0.01	6.27E-01
CNTN5	7	-0.01	0.01	3.06E-01
CNTNAP2	4	0.02	0.01	7.03E-02
COL18A1	4	-0.02	0.02	3.26E-01
COL1A1	10	-0.05	0.02	7.61E-03
COL4A1	9	0.00	0.01	9.54E-01
COL6A3	4	-0.03	0.04	3.65E-01
COL9A1	4	0.00	0.03	9.79E-01
COLEC12	4	-0.02	0.04	6.11E-01
COMP	6	0.00	0.02	8.21E-01
COMT	2	-0.01	0.01	1.87E-01
CORO1A	3	0.03	0.05	5.51E-01
COX5B	4	-0.05	0.04	1.94E-01
CPA1	21	0.02	0.01	5.06E-02
CPA2	4	0.00	0.01	8.93E-01
CPB1	14	0.00	0.01	8.67E-01
CPE	7	-0.05	0.02	1.20E-03
CPM	11	-0.02	0.02	4.01E-01
CPPED1	1	0.00	0.01	8.81E-01
CPVL	12	0.01	0.01	1.25E-01
CPXM1	19	-0.01	0.01	1.61E-01
CR2	24	-0.01	0.01	5.76E-01
CRACR2A	4	-0.03	0.03	3.45E-01
CRADD	5	-0.02	0.04	5.68E-01
CREG1	12	0.04	0.02	1.53E-02
CRELD2	12	0.00	0.01	9.39E-01
CRH	22	0.01	0.01	3.88E-01
CRHBP	5	-0.01	0.01	4.82E-01
CRIM1	4	-0.14	0.04	9.95E-05
CRIP2	4	0.18	0.03	1.74E-11
CRISP2	13	-0.01	0.01	3.75E-01
CRKL	1	-0.08	0.07	2.63E-01
CRLF1	8	-0.04	0.03	2.40E-01
CRNN	3	0.01	0.01	7.08E-02
CRTAC1	2	0.00	0.01	5.12E-01
CRTAM	14	-0.01	0.01	6.78E-01
CSF1	3	-0.05	0.02	3.49E-02
CSF2RA	1	0.03	0.09	7.34E-01
CSF3	3	0.09	0.03	4.82E-04
CST3	6	0.01	0.02	6.09E-01
CST5	13	0.01	0.01	3.93E-01
CST6	15	-0.01	0.01	4.18E-01
CST7	11	0.00	0.01	4.84E-01
CTF1	2	0.00	0.06	9.93E-01
CTRB1	13	-0.02	0.01	6.93E-03
CTRC	12	0.01	0.01	3.60E-01
CTSB	7	0.00	0.01	7.42E-01
CTSC	7	0.00	0.01	5.69E-01
CTSD	6	0.00	0.01	8.53E-01
CTSF	15	0.00	0.01	9.41E-01
CTSH	1	0.00	0.05	9.63E-01
CTSL	17	0.01	0.01	3.26E-01

CTSO	10	0.01	0.02	5.02E-01
CTSS	7	-0.27	0.03	1.74E-25
CTSV	18	-0.04	0.01	2.85E-03
CTSZ	11	-0.01	0.01	3.10E-01
CX3CL1	8	-0.30	0.03	1.35E-29
CXADR	5	0.11	0.02	1.21E-08
CXCL1	4	0.01	0.01	2.35E-01
CXCL10	4	0.00	0.01	8.23E-01
CXCL11	9	-0.04	0.02	2.17E-02
CXCL12	10	-0.05	0.02	1.20E-02
CXCL13	8	-0.02	0.03	4.51E-01
CXCL14	2	0.00	0.05	9.45E-01
CXCL16	12	0.02	0.02	2.78E-01
CXCL17	6	-0.06	0.03	7.27E-02
CXCL3	3	0.05	0.04	2.34E-01
CXCL5	11	0.00	0.01	9.84E-01
CXCL6	7	-0.02	0.01	3.48E-02
CXCL8	9	0.03	0.02	1.31E-01
CXCL9	5	-0.01	0.02	7.76E-01
DAB2	4	-0.06	0.04	1.36E-01
DAG1	3	-0.02	0.05	7.73E-01
DAPP1	2	0.02	0.04	5.71E-01
DARS1	6	-0.01	0.03	7.41E-01
DBI	4	0.03	0.02	8.42E-02
DBNL	4	-0.01	0.04	8.42E-01
DCBLD2	16	-0.03	0.01	6.08E-02
DCN	1	-0.04	0.08	6.28E-01
DCTN1	2	0.01	0.06	9.17E-01
DCTN2	2	0.03	0.07	6.67E-01
DCTPP1	6	-0.01	0.03	7.63E-01
DCXR	6	0.01	0.03	6.92E-01
DDAH1	5	-0.01	0.02	8.08E-01
DDC	7	0.00	0.01	9.79E-01
DDR1	12	0.00	0.01	8.64E-01
DDX58	1	-0.02	0.02	2.73E-01
DECR1	3	-0.05	0.04	2.28E-01
DEFA1_DEFA1B	16	0.01	0.02	4.50E-01
DEFB4A_DEFB4B	4	-0.02	0.04	5.69E-01
DFFA	1	-0.01	0.07	8.82E-01
DIABLO	3	-0.13	0.05	1.41E-02
DKK1	12	-0.01	0.02	4.01E-01
DKK3	8	-0.04	0.01	1.04E-03
DKK4	6	0.01	0.02	3.98E-01
DKKL1	5	0.00	0.00	3.29E-01
DLK1	7	0.00	0.01	7.15E-01
DLL1	7	0.05	0.02	8.26E-03
DNAJA2	1	0.06	0.08	4.52E-01
DNAJB1	7	-0.02	0.03	5.66E-01
DNAJB8	2	-0.05	0.05	3.41E-01
DNER	7	-0.01	0.02	5.13E-01
DNMBP	4	-0.04	0.03	2.36E-01
DNPH1	1	-0.01	0.02	5.96E-01
DOK2	4	-0.03	0.04	3.93E-01
DPEP1	8	0.01	0.01	6.38E-02
DPEP2	3	-0.01	0.02	7.95E-01
DPP10	5	-0.08	0.02	5.31E-08
DPP4	5	0.00	0.02	8.14E-01
DPP6	7	-0.07	0.01	7.91E-07
DPP7	7	0.00	0.02	8.58E-01
DPT	2	0.00	0.01	7.65E-01

DPY30	2	-0.01	0.06	8.96E-01
DRAXIN	17	-0.06	0.01	5.43E-06
DRG2	4	0.01	0.03	6.87E-01
DSC2	9	0.02	0.01	1.46E-01
DSG2	10	0.03	0.01	2.34E-02
DSG3	17	0.00	0.01	8.77E-01
DSG4	22	-0.04	0.01	8.05E-03
DTX3	3	-0.03	0.02	2.91E-01
DUSP3	1	-0.06	0.07	4.49E-01
EBAG9	5	0.02	0.03	5.28E-01
EBI3_IL27	21	-0.02	0.01	7.75E-02
ECE1	5	0.02	0.03	4.13E-01
EDA2R	3	0.05	0.06	3.89E-01
EDAR	23	0.00	0.01	8.14E-01
EDIL3	3	-0.01	0.03	8.45E-01
EFEMP1	4	-0.02	0.02	3.42E-01
EFNA1	4	0.09	0.01	3.67E-11
EFNA4	5	-0.03	0.03	2.61E-01
EGF	11	-0.02	0.02	2.51E-01
EGFL7	18	-0.02	0.01	1.91E-01
EGFR	6	0.00	0.02	9.66E-01
EGLN1	4	0.03	0.03	2.68E-01
EIF4B	4	-0.02	0.04	6.37E-01
EIF4G1	3	-0.01	0.04	8.05E-01
ELOA	2	0.01	0.03	6.91E-01
ENAH	1	-0.01	0.04	7.75E-01
ENG	8	-0.09	0.01	3.68E-25
ENO1	4	0.04	0.03	1.41E-01
ENO2	5	-0.02	0.02	3.07E-01
ENPP2	8	-0.03	0.02	1.29E-01
ENPP5	7	0.00	0.01	8.79E-01
ENPP7	6	0.00	0.01	9.48E-01
ENTPD2	2	0.20	0.07	4.16E-03
ENTPD5	9	-0.02	0.01	3.81E-02
ENTPD6	9	0.02	0.01	5.92E-05
EPCAM	14	0.05	0.01	3.90E-05
EPHA1	7	-0.03	0.02	1.71E-01
EPHA2	6	0.04	0.02	7.30E-02
EPHB4	10	-0.12	0.01	2.51E-23
EPHB6	4	-0.03	0.02	1.02E-01
EPO	9	0.05	0.02	5.81E-03
EPS8L2	4	0.01	0.02	3.67E-01
ERBB2	9	0.03	0.02	2.03E-01
ERBB3	9	0.00	0.01	8.94E-01
ERBB4	17	-0.09	0.01	3.41E-11
ERBIN	3	0.05	0.05	2.80E-01
EREG	10	-0.03	0.02	1.58E-01
ERP44	6	0.00	0.01	6.44E-01
ESAM	8	-0.01	0.02	6.33E-01
ESM1	11	-0.04	0.02	5.18E-02
EZR	3	-0.05	0.05	2.35E-01
F11R	12	0.00	0.02	9.92E-01
F2R	8	-0.10	0.02	1.01E-06
F3	9	-0.03	0.01	7.32E-03
F7	7	0.00	0.01	7.15E-01
F9	2	0.02	0.04	5.80E-01
FABP1	2	0.00	0.02	9.11E-01
FABP2	5	0.07	0.02	8.88E-04
FABP4	2	0.03	0.05	5.27E-01
FABP5	2	0.00	0.04	9.72E-01

FABP6	4	0.02	0.02	1.89E-01
FABP9	17	0.01	0.01	3.90E-01
FADD	4	-0.01	0.04	8.02E-01
FAM3B	7	-0.02	0.01	8.44E-03
FAM3C	2	-0.06	0.05	2.33E-01
FAP	7	0.00	0.02	7.61E-01
FAS	4	0.00	0.02	9.02E-01
FASLG	21	-0.01	0.01	3.14E-01
FBP1	5	0.00	0.02	8.51E-01
FCAR	14	-0.01	0.01	1.31E-01
FCER2	14	0.00	0.01	7.86E-01
FCGR2A	5	0.00	0.00	5.78E-01
FCGR2B	4	-0.07	0.01	1.94E-12
FCGR3B	11	-0.01	0.01	5.51E-01
FCN2	11	0.00	0.01	7.92E-01
FCRL1	26	-0.02	0.01	1.36E-02
FCRL2	16	-0.01	0.01	4.83E-01
FCRL3	5	-0.01	0.00	8.68E-02
FCRL5	10	0.05	0.02	1.56E-02
FCRL6	6	-0.01	0.01	1.44E-01
FCRLB	7	-0.01	0.01	3.34E-01
FEN1	1	0.04	0.09	6.61E-01
FES	1	-0.02	0.03	6.57E-01
FETUB	4	-0.01	0.01	3.15E-01
FGF19	5	0.14	0.02	5.58E-17
FGF2	6	0.00	0.01	5.53E-01
FGF21	7	-0.02	0.02	4.08E-01
FGF23	6	0.12	0.02	2.42E-06
FGF5	2	-0.01	0.01	2.80E-01
FGFBP1	13	0.01	0.02	5.45E-01
FGFR2	8	0.14	0.03	4.51E-08
FGR	1	-0.02	0.06	7.43E-01
FHIT	2	-0.07	0.04	1.12E-01
FIS1	5	-0.03	0.02	2.13E-01
FKBP1B	5	-0.01	0.02	7.48E-01
FKBP4	6	-0.05	0.03	1.30E-01
FKBP5	5	-0.04	0.02	8.35E-02
FLI1	3	-0.08	0.04	7.14E-02
FLRT2	11	0.00	0.01	7.75E-01
FLT1	9	-0.06	0.01	1.87E-07
FLT3	6	0.01	0.03	8.14E-01
FLT3LG	20	0.02	0.01	1.30E-01
FLT4	12	-0.03	0.01	4.83E-07
FOLR1	8	-0.12	0.02	2.35E-08
FOLR2	14	0.02	0.01	1.40E-01
FOLR3	10	0.00	0.02	9.57E-01
FOXO1	4	0.03	0.03	2.13E-01
FOXO3	1	0.06	0.09	4.98E-01
FRZB	4	0.00	0.01	8.05E-01
FST	3	0.01	0.02	6.51E-01
FSTL3	2	-0.06	0.05	1.75E-01
FUCA1	5	0.00	0.00	7.28E-01
FURIN	3	0.01	0.02	7.47E-01
FUS	2	0.01	0.05	7.79E-01
FUT3_FUT5	14	0.00	0.01	8.42E-01
FUT8	12	0.01	0.01	8.08E-02
FXN	4	-0.07	0.05	1.16E-01
FXYD5	3	-0.02	0.02	3.16E-01
FYB1	2	-0.02	0.05	7.19E-01
GAL	22	0.02	0.01	1.30E-01

GALNT10	9	-0.01	0.01	2.51E-01
GALNT2	6	0.03	0.01	6.10E-02
GALNT3	9	0.01	0.01	4.82E-01
GALNT7	8	0.00	0.01	7.45E-01
GAS6	17	-0.02	0.01	9.18E-02
GBP2	1	-0.08	0.04	7.81E-02
GBP4	1	0.00	0.01	8.83E-01
GCG	5	0.03	0.04	3.83E-01
GCNT1	8	0.03	0.01	6.31E-03
GDF15	1	0.02	0.02	2.43E-01
GDF2	14	0.01	0.01	4.39E-01
GDNF	2	0.03	0.02	1.83E-01
GFAP	8	-0.03	0.03	2.70E-01
GFER	1	-0.05	0.08	4.98E-01
GFRA1	4	0.06	0.02	4.13E-04
GFRA2	7	0.03	0.01	3.39E-02
GFRA3	22	0.00	0.01	8.48E-01
GGH	11	0.00	0.01	8.72E-01
GGT1	7	-0.01	0.02	5.87E-01
GGT5	4	0.00	0.02	7.97E-01
GHRL	11	0.00	0.01	7.69E-01
GKN1	4	0.27	0.04	4.64E-14
GLB1	10	0.00	0.01	9.15E-01
GLO1	1	-0.01	0.01	6.46E-01
GLOD4	2	-0.05	0.06	3.88E-01
GLRX	2	0.00	0.01	7.16E-01
GLT8D2	1	-0.02	0.03	3.77E-01
GMPR	2	0.00	0.02	8.86E-01
GNE	3	-0.04	0.03	2.66E-01
GNLY	12	0.00	0.01	5.46E-01
GOLM2	8	0.15	0.01	2.00E-33
GOPC	2	-0.04	0.05	4.46E-01
GP1BA	17	-0.04	0.01	8.60E-03
GP2	8	0.11	0.01	1.29E-46
GP6	6	-0.01	0.01	3.31E-01
GPA33	14	0.04	0.01	1.84E-04
GPC1	17	-0.02	0.01	1.27E-01
GPC5	10	-0.01	0.01	3.15E-01
GPNMB	6	-0.01	0.01	4.53E-01
GPR37	10	0.00	0.01	7.18E-01
GRAP2	3	-0.01	0.05	7.58E-01
GRK5	1	0.02	0.02	2.64E-01
GRN	12	-0.01	0.01	2.17E-01
GRPEL1	3	0.07	0.04	8.46E-02
GSAP	1	0.01	0.07	9.01E-01
GSTA1	9	0.00	0.01	7.98E-01
GSTA3	5	0.00	0.01	7.98E-01
GSTP1	1	-0.01	0.02	5.53E-01
GUCA2A	7	0.00	0.02	8.96E-01
GUSB	14	0.00	0.01	7.50E-01
GYS1	3	0.03	0.02	1.49E-01
GZMA	16	-0.03	0.01	3.52E-02
GZMB	10	-0.01	0.01	7.01E-01
GZMH	9	-0.02	0.02	3.59E-01
HAGH	1	-0.03	0.05	5.40E-01
HAO1	9	0.03	0.03	2.74E-01
HARS1	3	-0.01	0.05	8.52E-01
HAVCR1	8	-0.01	0.01	4.50E-01
HAVCR2	8	0.00	0.02	8.70E-01
HBEGF	24	-0.01	0.01	5.84E-01

HBQ1	2	0.02	0.01	1.14E-01
HCLS1	4	0.01	0.03	8.80E-01
HDGF	2	0.00	0.00	2.54E-01
HEBP1	1	-0.01	0.04	7.68E-01
HEXIM1	5	-0.04	0.03	1.80E-01
HGF	5	-0.07	0.02	4.96E-03
HGS	4	-0.01	0.04	8.56E-01
HLA-DRA	3	0.00	0.01	9.51E-01
HLA-E	4	0.00	0.01	9.17E-01
HMBS	1	-0.05	0.04	2.08E-01
HMOX1	19	-0.02	0.01	6.06E-02
HMOX2	4	-0.06	0.03	2.50E-02
HNMT	4	0.01	0.01	1.39E-01
HNRNPK	1	-0.09	0.08	2.63E-01
HPCAL1	1	-0.03	0.05	4.84E-01
HPGDS	2	0.00	0.01	7.71E-01
HS3ST3B1	10	-0.01	0.01	2.23E-01
HS6ST1	17	0.00	0.01	9.99E-01
HSD11B1	12	0.00	0.01	7.87E-01
HSPA1A	3	-0.03	0.04	4.59E-01
HSPB1	5	0.02	0.03	4.90E-01
HSPB6	5	-0.02	0.04	6.23E-01
HSPG2	4	-0.01	0.03	7.31E-01
HTRA2	1	-0.10	0.09	2.63E-01
HYAL1	4	-0.01	0.02	3.97E-01
HYOU1	13	-0.02	0.02	2.16E-01
ICA1	3	-0.04	0.02	7.54E-02
ICAM1	6	-0.03	0.01	4.61E-03
ICAM2	10	-0.05	0.00	3.93E-23
ICAM3	21	-0.02	0.01	6.73E-03
ICAM4	14	0.01	0.01	7.23E-01
ICAM5	7	-0.05	0.01	1.22E-14
ICOSLG	4	0.00	0.01	9.89E-01
IDI2	1	0.01	0.01	3.96E-01
IDS	3	-0.11	0.02	1.06E-07
IDUA	13	0.02	0.01	3.17E-02
IFNG	2	-0.12	0.07	8.11E-02
IFNGR1	10	0.02	0.01	1.24E-01
IFNGR2	10	0.00	0.00	7.67E-01
IFNL1	6	-0.06	0.03	6.37E-02
IFNLR1	3	0.01	0.02	4.55E-01
IGF1R	9	-0.14	0.01	5.89E-40
IGF2R	14	0.00	0.01	7.13E-01
IGFBP1	2	0.01	0.05	7.60E-01
IGFBP2	4	0.00	0.04	9.52E-01
IGFBP3	5	-0.01	0.01	6.09E-01
IGFBP4	3	0.04	0.05	4.71E-01
IGFBP6	4	0.01	0.04	8.23E-01
IGFBP7	4	0.00	0.01	7.73E-01
IGFBPL1	2	-0.01	0.01	2.75E-01
IGSF3	5	0.05	0.03	2.81E-02
IGSF8	6	0.02	0.02	4.38E-01
IKBKG	1	-0.01	0.10	8.82E-01
IKZF2	2	-0.08	0.04	6.07E-02
IL10	5	-0.02	0.02	2.48E-01
IL10RA	1	0.00	0.03	8.91E-01
IL10RB	6	-0.01	0.01	2.67E-01
IL11	1	0.08	0.11	4.26E-01
IL12A_IL12B	17	-0.01	0.01	2.45E-01
IL12B	20	-0.01	0.01	2.96E-01

IL12RB1	5	0.00	0.01	7.45E-01
IL13	1	0.06	0.09	5.29E-01
IL13RA1	2	0.04	0.05	4.04E-01
IL15	21	-0.01	0.01	6.55E-01
IL15RA	2	0.01	0.02	7.07E-01
IL16	2	-0.02	0.01	1.51E-01
IL17C	3	-0.03	0.03	3.83E-01
IL17D	3	0.00	0.01	7.89E-01
IL17F	1	0.04	0.11	7.35E-01
IL17RA	3	0.00	0.01	3.94E-01
IL17RB	11	0.00	0.01	6.93E-01
IL18	10	-0.03	0.01	9.15E-03
IL18BP	9	-0.03	0.02	8.11E-02
IL18R1	12	-0.01	0.01	1.65E-01
IL18RAP	3	-0.03	0.03	4.45E-01
IL19	5	-0.05	0.01	1.71E-08
IL1A	1	0.04	0.04	2.94E-01
IL1B	2	-0.01	0.02	6.60E-01
IL1R1	13	0.01	0.01	4.87E-01
IL1R2	14	0.01	0.01	5.27E-01
IL1RAP	4	0.00	0.00	9.72E-01
IL1RL1	13	0.00	0.01	6.80E-01
IL1RL2	12	0.02	0.01	5.72E-02
IL1RN	2	-0.05	0.02	2.48E-02
IL20RA	1	-0.05	0.08	5.81E-01
IL22RA1	2	0.06	0.02	1.92E-04
IL2RA	8	-0.02	0.01	1.29E-02
IL32	9	-0.01	0.02	4.10E-01
IL33	1	-0.04	0.13	7.42E-01
IL34	1	-0.01	0.01	7.53E-02
IL3RA	4	0.02	0.03	5.62E-01
IL4R	5	-0.04	0.01	6.27E-03
IL5RA	23	0.02	0.01	6.93E-02
IL6	4	-0.01	0.02	5.74E-01
IL6R	6	-0.01	0.00	1.22E-01
IL6ST	12	-0.09	0.01	1.05E-12
IL7	4	-0.04	0.04	2.73E-01
IL7R	12	-0.03	0.00	1.01E-09
ILKAP	2	-0.12	0.07	9.88E-02
IMPA1	2	-0.01	0.02	7.04E-01
ING1	1	-0.10	0.09	2.63E-01
INHBC	6	0.00	0.01	8.86E-01
INPP1	3	-0.05	0.04	2.73E-01
IPCEF1	2	-0.04	0.05	3.83E-01
IQGAP2	1	0.01	0.03	8.55E-01
IRAG2	2	-0.13	0.06	1.95E-02
IRAK1	1	-0.01	0.08	8.82E-01
IRAK4	1	-0.09	0.10	3.61E-01
ISLR2	8	-0.10	0.01	1.62E-40
ISM1	3	-0.03	0.03	1.72E-01
ITGA11	5	0.02	0.02	2.01E-01
ITGA5	11	0.00	0.02	9.83E-01
ITGA6	7	-0.09	0.01	4.00E-09
ITGAM	10	-0.03	0.02	7.33E-02
ITGAV	6	-0.01	0.02	7.05E-01
ITGB1	7	-0.12	0.02	3.98E-09
ITGB1BP2	5	0.04	0.03	2.07E-01
ITGB2	10	-0.04	0.02	1.38E-02
ITGB5	6	-0.02	0.03	4.88E-01
ITGB6	9	-0.04	0.01	5.92E-03

ITGB7	19	0.02	0.01	2.95E-03
ITIH3	7	0.00	0.01	9.35E-01
ITM2A	3	-0.01	0.02	5.61E-01
JAM2	6	0.06	0.02	6.07E-03
JCHAIN	14	0.00	0.02	8.26E-01
KAZALD1	6	0.00	0.01	7.36E-01
KDR	12	0.01	0.01	3.38E-01
KEL	17	-0.07	0.01	2.31E-19
KIFBP	3	0.03	0.03	4.11E-01
KIR2DL3	4	0.01	0.01	2.68E-01
KIR3DL1	3	0.01	0.01	3.40E-01
KIRREL2	8	0.03	0.01	1.26E-02
KIT	26	-0.01	0.01	5.88E-01
KITLG	15	-0.01	0.01	2.89E-01
KLB	9	0.00	0.01	8.11E-01
KLK1	13	-0.04	0.01	3.77E-14
KLK10	5	0.01	0.01	2.65E-01
KLK11	5	0.00	0.01	9.19E-01
KLK12	5	0.00	0.00	8.17E-01
KLK13	10	0.02	0.01	3.57E-02
KLK14	11	-0.02	0.01	5.03E-02
KLK4	3	0.01	0.01	4.25E-01
KLK6	3	-0.02	0.02	3.44E-01
KLK8	5	0.01	0.01	2.65E-01
KLRB1	11	-0.02	0.02	1.81E-01
KLRD1	14	0.00	0.01	8.22E-01
KRT18	10	-0.04	0.02	1.41E-01
KRT19	3	0.09	0.03	2.07E-03
KRT5	5	0.04	0.02	1.11E-01
KYAT1	6	-0.07	0.04	6.97E-02
KYNU	5	-0.03	0.02	9.01E-02
L1CAM	6	-0.02	0.02	3.44E-01
LACTB2	3	-0.02	0.03	5.91E-01
LAG3	12	-0.03	0.01	7.51E-02
LAIR1	4	-0.01	0.01	3.69E-01
LAIR2	5	0.00	0.01	6.44E-01
LAMA4	12	-0.01	0.01	6.90E-01
LAMP2	2	0.04	0.06	5.58E-01
LAMP3	11	-0.06	0.02	5.87E-04
LAT	6	-0.03	0.03	3.81E-01
LAT2	1	-0.01	0.08	8.82E-01
LAYN	4	0.02	0.01	5.01E-02
LBP	2	0.00	0.01	8.64E-01
LBR	6	-0.03	0.02	1.28E-01
LCN2	17	0.01	0.02	5.87E-01
LDLR	17	-0.02	0.01	1.79E-01
LEFTY2	3	-0.45	0.04	1.80E-23
LEP	1	0.04	0.10	7.23E-01
LEPR	9	0.01	0.02	6.47E-01
LGALS1	2	0.00	0.02	8.24E-01
LGALS3	7	-0.03	0.01	2.35E-03
LGALS4	3	-0.16	0.01	3.21E-75
LGALS7_LGALS7B	7	0.15	0.01	2.58E-40
LGALS8	7	-0.11	0.01	8.95E-32
LGALS9	4	-0.03	0.01	1.55E-02
LGMN	19	-0.03	0.01	3.61E-02
LHB	1	0.06	0.08	4.45E-01
LIFR	13	-0.12	0.01	1.29E-29
LILRA2	11	-0.01	0.01	3.18E-01
LILRA5	9	-0.02	0.02	1.78E-01

LILRB1	9	0.00	0.01	7.60E-01
LILRB2	10	-0.01	0.01	6.28E-02
LILRB4	4	-0.02	0.02	2.87E-01
LILRB5	3	0.00	0.00	2.72E-01
LPCAT2	1	-0.11	0.04	1.15E-02
LPL	9	0.00	0.01	7.18E-01
LPO	14	-0.02	0.01	9.85E-02
LRIG1	11	0.00	0.01	5.11E-01
LRP1	11	-0.01	0.01	1.09E-01
LRP11	6	0.01	0.01	4.45E-01
LRPAP1	7	-0.01	0.01	5.87E-02
LRRC25	13	-0.01	0.01	1.72E-01
LRRN1	7	-0.01	0.01	3.64E-02
LSP1	2	0.00	0.03	9.46E-01
LTA	10	0.00	0.01	5.41E-01
LTA4H	1	0.04	0.05	4.25E-01
LTBP2	8	-0.01	0.03	6.79E-01
LTBP3	6	0.01	0.01	3.22E-01
LTBR	13	-0.03	0.01	7.31E-03
LXN	2	0.00	0.03	9.09E-01
LY6D	5	0.01	0.01	4.70E-01
LY75	7	0.00	0.00	4.20E-01
LY9	14	0.00	0.01	5.82E-01
LY96	11	-0.01	0.02	7.18E-01
LYAR	1	-0.20	0.09	2.77E-02
LYN	5	-0.03	0.04	4.87E-01
LYPD3	17	-0.04	0.01	1.34E-07
LYPD8	7	0.01	0.01	4.34E-01
MAD1L1	1	-0.13	0.05	8.54E-03
MAGED1	2	0.02	0.05	6.18E-01
MANF	6	-0.03	0.03	3.52E-01
MANSC1	18	-0.04	0.02	2.85E-02
MAP2K6	3	-0.05	0.05	3.08E-01
MAP3K5	1	-0.01	0.08	8.82E-01
MAP4K5	3	0.06	0.03	1.43E-02
MAPK9	1	-0.09	0.04	1.64E-02
MARCO	9	-0.01	0.01	2.95E-01
MASP1	13	-0.01	0.01	2.32E-01
MATN2	5	-0.01	0.01	2.35E-01
MATN3	4	0.00	0.01	7.30E-01
MAVS	3	-0.01	0.04	7.57E-01
MAX	8	0.00	0.03	9.23E-01
MB	8	0.03	0.03	2.79E-01
MCAM	9	0.04	0.02	2.79E-02
MCFD2	4	-0.03	0.03	2.68E-01
MDGA1	6	0.00	0.00	7.21E-01
MDK	11	-0.03	0.02	1.79E-01
MED18	2	-0.14	0.07	4.58E-02
MEGF10	13	-0.01	0.01	4.22E-01
MEGF9	11	0.01	0.01	2.83E-01
MEP1B	15	0.00	0.00	7.25E-01
MEPE	15	0.00	0.02	7.76E-01
MERTK	14	0.01	0.01	3.55E-01
MESD	4	0.02	0.04	5.68E-01
MET	20	-0.10	0.01	6.77E-21
METAP1D	3	-0.11	0.05	2.95E-02
METAP2	4	-0.01	0.03	7.33E-01
MFAP5	6	-0.10	0.02	1.58E-05
MFGE8	12	-0.01	0.01	3.24E-01
MGLL	3	-0.06	0.04	1.69E-01

MGMT	3	0.01	0.01	5.75E-01
MIA	7	0.00	0.01	9.67E-01
MICB_MICA	8	0.00	0.00	9.08E-01
MIF	4	0.01	0.03	7.09E-01
MILR1	9	-0.01	0.01	5.15E-01
MITD1	2	0.01	0.05	8.94E-01
MLN	22	0.02	0.01	7.65E-02
MME	14	0.01	0.01	4.70E-01
MMP1	13	-0.01	0.01	1.91E-01
MMP10	2	-0.06	0.01	7.42E-05
MMP12	3	0.01	0.01	2.05E-01
MMP13	1	-0.01	0.03	7.07E-01
MMP3	4	0.01	0.01	4.15E-01
MMP7	5	-0.01	0.01	4.61E-01
MMP8	2	0.01	0.01	6.66E-01
MMP9	9	-0.04	0.02	1.20E-02
MNDA	1	-0.42	0.09	7.24E-06
MOG	2	0.02	0.02	4.65E-01
MPHOSPH8	2	-0.01	0.05	8.39E-01
MPI	4	-0.05	0.02	2.16E-02
MPIG6B	5	-0.06	0.03	6.33E-02
MPO	7	-0.03	0.02	6.80E-02
MSLN	8	-0.01	0.01	3.73E-01
MSMB	5	0.00	0.01	6.08E-01
MSR1	13	0.02	0.01	1.84E-01
MSRA	2	0.00	0.06	9.36E-01
MSTN	6	0.01	0.03	8.47E-01
MUC13	4	-0.16	0.01	4.39E-26
MUC16	3	0.01	0.02	8.20E-01
MVK	1	0.10	0.07	2.01E-01
MYO9B	1	0.10	0.12	4.17E-01
MYOC	11	0.04	0.02	1.81E-02
MZB1	7	0.03	0.02	2.14E-01
MZT1	2	-0.03	0.06	6.70E-01
NAAA	17	0.01	0.01	1.97E-01
NADK	5	-0.04	0.02	8.48E-03
NBL1	3	-0.02	0.03	5.93E-01
NBN	3	-0.03	0.05	5.73E-01
NCAM1	8	-0.03	0.01	3.95E-02
NCAM2	5	0.03	0.02	2.03E-01
NCAN	12	0.00	0.02	8.36E-01
NCF2	4	-0.02	0.02	3.89E-01
NCK2	1	-0.08	0.10	4.17E-01
NCLN	1	0.03	0.13	8.41E-01
NCR1	24	-0.03	0.01	2.05E-02
NCS1	7	-0.01	0.03	6.50E-01
NECTIN2	4	0.07	0.02	1.89E-04
NECTIN4	6	-0.01	0.02	6.68E-01
NEFL	2	-0.09	0.07	2.01E-01
NELL1	20	0.01	0.01	2.40E-01
NELL2	15	-0.17	0.02	3.52E-20
NFASC	4	-0.04	0.01	8.31E-04
NFATC1	2	0.02	0.04	6.62E-01
NFATC3	1	0.08	0.08	3.22E-01
NFKBIE	2	0.00	0.02	9.24E-01
NID1	12	0.01	0.02	6.18E-01
NID2	17	-0.01	0.01	3.24E-01
NINJ1	2	0.01	0.03	8.15E-01
NME3	14	-0.05	0.01	3.53E-05
NMNAT1	4	-0.02	0.02	2.14E-01

NOMO1	3	-0.04	0.03	2.55E-01
NOS1	2	0.01	0.04	9.06E-01
NOS3	3	-0.08	0.05	1.33E-01
NOTCH1	7	0.05	0.01	5.46E-04
NOTCH3	11	-0.09	0.02	1.57E-04
NPDC1	5	-0.07	0.04	7.78E-02
NPPB	2	0.00	0.02	8.58E-01
NPPC	2	0.06	0.07	3.79E-01
NPTX1	14	-0.03	0.01	1.85E-04
NPTXR	6	0.00	0.01	8.00E-01
NPY	13	0.01	0.02	6.39E-01
NRCAM	7	-0.20	0.02	1.97E-16
NRP1	6	-0.03	0.01	1.61E-02
NRP2	9	0.04	0.02	2.33E-02
NRTN	1	0.15	0.09	9.18E-02
NSFL1C	2	0.07	0.05	1.48E-01
NT5C3A	4	0.04	0.03	2.87E-01
NT5E	11	-0.01	0.01	2.72E-01
NTF3	5	0.03	0.02	2.30E-01
NTF4	1	-0.03	0.09	7.72E-01
NTproBNP	4	0.01	0.02	7.31E-01
NTRK2	7	0.04	0.03	2.10E-01
NTRK3	10	0.03	0.02	7.95E-02
NUB1	4	-0.03	0.02	2.56E-01
NUCB2	5	0.00	0.01	6.99E-01
NUDC	2	0.02	0.05	7.15E-01
NUDT2	2	0.02	0.02	3.84E-01
NUDT5	3	-0.06	0.05	2.70E-01
OBP2B	13	0.00	0.01	7.19E-01
ODAM	2	-0.02	0.02	3.64E-01
OGFR	2	0.01	0.04	8.59E-01
OGN	4	0.02	0.01	3.38E-02
OLR1	7	-0.04	0.02	7.91E-02
OMD	6	0.00	0.02	8.33E-01
OMG	8	0.01	0.02	5.94E-01
OPTC	8	0.00	0.02	9.25E-01
OSCAR	9	0.01	0.01	2.58E-01
OSM	8	-0.06	0.02	1.81E-03
OSMR	5	0.01	0.01	1.18E-01
OXT	10	-0.01	0.01	5.14E-01
P4HB	4	-0.07	0.04	7.39E-02
PADI2	1	-0.01	0.04	8.60E-01
PADI4	1	0.00	0.02	9.09E-01
PAEP	3	0.00	0.01	5.36E-01
PAG1	6	-0.08	0.03	2.68E-03
PAK4	2	-0.07	0.08	3.68E-01
PAM	4	0.00	0.01	8.36E-01
PAMR1	4	-0.01	0.01	5.33E-01
PAPPA	11	0.03	0.02	3.64E-02
PARK7	5	-0.03	0.02	2.28E-01
PARP1	2	-0.04	0.03	1.63E-01
PBLD	1	0.02	0.01	1.19E-01
PCDH1	6	0.10	0.03	1.63E-03
PCDH17	9	0.02	0.01	7.70E-03
PCOLCE	1	0.08	0.03	5.86E-03
PCSK9	9	-0.01	0.01	6.68E-01
PDCD1	12	-0.01	0.02	7.82E-01
PDCD1LG2	8	-0.01	0.01	1.79E-02
PDCD5	3	0.01	0.01	3.94E-01
PDCD6	5	0.00	0.00	9.99E-01

PDGFA	19	-0.01	0.01	5.90E-01
PDGFB	18	-0.01	0.01	5.08E-01
PDGFC	7	-0.02	0.02	1.37E-01
PDGFRA	11	0.04	0.01	2.10E-03
PDGFRB	2	0.00	0.00	8.62E-01
PDLIM7	3	0.04	0.04	3.68E-01
PEAR1	9	0.03	0.01	1.59E-02
PEBP1	2	-0.04	0.06	5.46E-01
PECAM1	8	-0.12	0.01	2.21E-41
PFKFB2	4	-0.02	0.02	2.73E-01
PGF	3	-0.02	0.04	6.27E-01
PGLYRP1	13	0.01	0.01	3.64E-01
PI3	4	0.00	0.02	7.64E-01
PIGR	6	0.07	0.02	3.20E-04
PIK3AP1	7	-0.04	0.02	7.34E-02
PIK3IP1	3	-0.01	0.02	7.42E-01
PILRA	6	0.00	0.00	3.63E-01
PILRB	4	0.00	0.00	4.12E-01
PKLR	4	-0.03	0.02	1.91E-01
PLA2G10	15	0.02	0.01	8.20E-02
PLA2G15	9	0.00	0.01	8.55E-01
PLA2G1B	18	-0.04	0.02	1.81E-02
PLA2G2A	6	0.00	0.01	7.27E-01
PLA2G4A	3	-0.04	0.05	4.39E-01
PLA2G7	10	-0.01	0.02	3.86E-01
PLAT	4	-0.16	0.04	1.50E-05
PLAU	10	0.01	0.01	3.51E-01
PLAUR	11	0.01	0.02	5.38E-01
PLIN1	1	-0.10	0.08	2.29E-01
PLIN3	2	0.00	0.05	9.94E-01
PLPBP	2	-0.04	0.05	3.99E-01
PLTP	11	0.01	0.01	2.83E-01
PLXDC1	6	0.03	0.02	9.15E-02
PLXNA4	5	-0.01	0.03	6.29E-01
PLXNB2	11	-0.01	0.01	2.77E-02
PLXNB3	5	-0.02	0.04	5.58E-01
PM20D1	8	0.00	0.01	9.21E-01
PMVK	6	-0.02	0.03	5.26E-01
PNLIPRP2	7	0.00	0.00	3.29E-01
PNPT1	2	0.04	0.05	4.44E-01
PODXL	6	-0.10	0.01	7.81E-37
PODXL2	12	0.01	0.02	5.34E-01
POLR2F	1	-0.24	0.09	7.35E-03
PON2	3	-0.01	0.01	1.32E-01
PON3	9	0.02	0.01	5.26E-02
PPCDC	2	0.00	0.01	7.26E-01
PPIB	3	-0.06	0.05	2.06E-01
PPME1	1	-0.01	0.09	9.22E-01
PPP1R12A	4	0.06	0.04	1.12E-01
PPP1R2	3	-0.04	0.04	3.64E-01
PPP1R9B	2	0.02	0.06	7.34E-01
PPP3R1	5	-0.01	0.03	6.45E-01
PPY	8	0.00	0.02	9.39E-01
PRCP	12	0.01	0.01	3.86E-01
PRDX1	2	-0.08	0.05	1.34E-01
PRDX3	1	-0.16	0.07	2.98E-02
PRDX5	2	0.01	0.04	8.50E-01
PRDX6	1	0.02	0.03	4.84E-01
PRELP	1	0.01	0.01	2.71E-01
PRKAB1	2	-0.04	0.07	5.93E-01

PRKAR1A	5	0.00	0.02	9.11E-01
PRKRA	2	-0.03	0.04	5.12E-01
PRL	3	0.00	0.04	9.18E-01
PROC	8	0.01	0.02	5.65E-01
PROK1	3	0.01	0.01	3.68E-01
PRSS2	13	0.04	0.02	2.09E-02
PRSS27	18	-0.05	0.01	6.78E-12
PRSS8	8	0.05	0.02	4.50E-02
PRTFDC1	3	0.03	0.02	1.84E-01
PRTG	9	0.00	0.01	8.96E-01
PRTN3	15	0.00	0.01	9.57E-01
PSG1	7	-0.01	0.01	3.97E-01
PSIP1	3	-0.04	0.03	1.82E-01
PSME1	3	-0.01	0.04	7.76E-01
PSME2	1	0.00	0.05	9.82E-01
PSMG3	2	0.00	0.04	9.80E-01
PSPN	10	0.05	0.03	8.75E-02
PSRC1	2	-0.08	0.03	4.60E-03
PTGDS	2	0.09	0.06	1.43E-01
PTH1R	2	0.05	0.03	8.52E-02
PTN	4	-0.04	0.03	1.41E-01
PTPN1	3	-0.05	0.04	1.95E-01
PTPN6	2	0.03	0.05	6.13E-01
PTPRF	15	-0.03	0.02	5.74E-02
PTPRM	8	-0.11	0.01	6.61E-43
PTPRN2	1	0.07	0.03	3.34E-02
PTPRS	5	0.06	0.02	3.48E-04
PTS	4	-0.02	0.02	3.34E-01
PTX3	5	0.01	0.01	6.50E-01
PVALB	8	-0.01	0.01	1.13E-01
PVR	3	-0.01	0.01	7.88E-02
PXN	3	-0.01	0.01	4.53E-01
QDPR	1	-0.01	0.08	8.82E-01
QPCT	11	-0.01	0.01	6.34E-01
RAB6A	4	-0.01	0.01	2.29E-01
RABEPK	1	0.00	0.03	9.94E-01
RABGAP1L	2	0.06	0.05	2.20E-01
RAD23B	3	0.00	0.05	9.63E-01
RARRES1	1	0.01	0.01	1.79E-01
RARRES2	3	-0.01	0.02	6.98E-01
RASSF2	1	-0.06	0.05	2.61E-01
RBKS	1	0.00	0.01	7.82E-01
RBP2	4	0.05	0.03	1.02E-01
RBP5	3	0.01	0.02	7.33E-01
RCOR1	1	0.20	0.08	1.27E-02
REG1A	6	0.14	0.01	1.74E-32
REG1B	7	0.11	0.01	7.94E-26
REG3A	9	0.00	0.02	9.64E-01
REG4	1	0.05	0.03	1.07E-01
RELT	4	-0.02	0.01	2.35E-01
REN	2	-0.01	0.03	6.51E-01
RET	8	0.00	0.01	7.40E-01
RETN	15	0.01	0.01	6.22E-01
RGMA	18	0.00	0.01	7.50E-01
RGMB	4	-0.03	0.02	1.66E-01
RGS8	1	0.01	0.03	8.39E-01
RHOC	3	0.03	0.06	5.71E-01
RILP	4	0.00	0.04	9.48E-01
RNASE3	5	-0.02	0.02	1.84E-01
RNASET2	9	0.01	0.01	2.35E-01

ROBO1	9	0.01	0.02	7.08E-01
ROBO2	6	-0.01	0.03	6.82E-01
ROR1	12	-0.02	0.01	1.48E-01
RP2	2	-0.11	0.04	1.30E-02
RRM2	1	-0.04	0.08	6.37E-01
RRM2B	3	-0.01	0.04	7.99E-01
RSPO1	10	0.00	0.02	9.49E-01
RSPO3	8	0.01	0.02	6.66E-01
RTBDN	3	0.05	0.03	8.73E-02
RTN4R	10	-0.01	0.01	3.55E-01
RWDD1	3	-0.02	0.03	5.35E-01
S100A11	2	0.02	0.02	3.45E-01
S100A12	4	-0.02	0.02	2.52E-01
S100A16	3	0.15	0.03	4.27E-06
S100A4	5	0.03	0.03	3.43E-01
S100P	5	-0.06	0.03	4.02E-02
SAMD9L	2	0.01	0.02	4.56E-01
SCAMP3	4	0.01	0.03	7.56E-01
SCARA5	7	0.04	0.01	2.90E-03
SCARB1	1	-0.16	0.14	2.76E-01
SCARB2	5	0.02	0.02	2.48E-01
SCARF1	7	-0.03	0.01	1.22E-02
SCARF2	8	-0.09	0.01	8.92E-11
SCG2	4	-0.47	0.04	2.42E-35
SCG3	6	0.00	0.01	7.22E-01
SCGB1A1	18	0.03	0.01	2.93E-03
SCGB3A2	18	0.00	0.01	8.30E-01
SCGN	4	0.13	0.02	1.36E-08
SCLY	7	0.00	0.02	9.25E-01
SCP2	1	0.19	0.09	3.61E-02
SCRN1	2	0.02	0.03	6.52E-01
SDC1	13	-0.04	0.01	9.79E-03
SDC4	21	0.00	0.01	7.88E-01
SELE	9	-0.08	0.01	3.14E-41
SELP	10	-0.08	0.01	1.25E-11
SELPLG	12	-0.01	0.01	3.27E-01
SEMA3F	6	-0.24	0.03	5.26E-14
SEMA4C	6	-0.25	0.02	2.00E-39
SEMA4D	6	-0.01	0.03	6.39E-01
SEMA7A	19	0.00	0.01	7.31E-01
SEPTIN9	2	-0.04	0.05	4.75E-01
SERPINA11	8	0.00	0.01	8.21E-01
SERPINA12	5	0.01	0.01	1.62E-01
SERPINA9	3	0.00	0.01	9.87E-01
SERPINB1	3	0.05	0.04	1.57E-01
SERPINB6	2	0.02	0.03	6.30E-01
SERPINB8	2	-0.01	0.01	1.95E-01
SERPINB9	2	-0.05	0.04	2.02E-01
SERPINE1	18	-0.02	0.01	2.06E-01
SESTD1	3	0.00	0.04	9.49E-01
SETMAR	2	-0.01	0.02	6.24E-01
SEZ6L	8	0.02	0.02	3.02E-01
SEZ6L2	8	0.04	0.02	6.22E-02
SF3B4	4	-0.01	0.04	8.58E-01
SFRP1	5	0.01	0.02	5.83E-01
SFTPA1	1	-0.02	0.02	2.34E-01
SFTPA2	5	0.02	0.01	1.16E-03
SFTPD	12	-0.03	0.01	3.38E-06
SH2B3	4	0.03	0.04	4.61E-01
SH2D1A	2	-0.12	0.04	1.97E-03

SHMT1	5	0.01	0.01	2.49E-01
SIAE	9	0.00	0.02	9.58E-01
SIGLEC1	11	-0.01	0.01	6.73E-01
SIGLEC10	9	0.00	0.01	6.27E-01
SIGLEC5	3	0.00	0.01	7.75E-01
SIGLEC6	23	0.00	0.01	6.74E-01
SIGLEC7	9	-0.01	0.01	5.47E-01
SIGLEC9	11	-0.02	0.01	7.34E-04
SIRPA	8	0.00	0.00	4.42E-01
SIRPB1	11	-0.01	0.01	2.22E-01
SIRT2	3	0.01	0.03	6.30E-01
SIT1	6	-0.09	0.03	4.82E-04
SKAP1	8	-0.12	0.03	6.99E-06
SKAP2	3	0.02	0.04	6.59E-01
SLAMF1	6	-0.01	0.03	7.99E-01
SLAMF6	3	-0.07	0.02	6.29E-03
SLAMF7	9	0.00	0.01	5.45E-01
SLAMF8	5	-0.01	0.01	3.41E-01
SLC16A1	4	-0.01	0.03	6.69E-01
SLC27A4	3	-0.05	0.04	2.14E-01
SLC39A14	2	-0.10	0.04	1.03E-02
SLC39A5	8	0.01	0.02	6.14E-01
SLIT2	1	-0.06	0.08	4.50E-01
SLITRK2	11	0.08	0.02	2.77E-05
SLITRK6	8	0.01	0.01	2.59E-01
SMAD1	5	-0.02	0.04	6.03E-01
SMAD5	2	0.01	0.03	7.31E-01
SMOC1	11	-0.02	0.01	2.36E-01
SMOC2	5	0.00	0.01	9.44E-01
SMPD1	16	0.00	0.01	6.68E-01
SMPDL3A	21	0.00	0.01	9.40E-01
SNAP23	3	-0.02	0.04	5.49E-01
SNAP29	5	0.00	0.03	9.53E-01
SNCG	1	0.00	0.01	8.76E-01
SNX9	2	0.04	0.04	3.59E-01
SOD1	3	-0.04	0.04	3.23E-01
SOD2	9	0.01	0.02	4.50E-01
SORCS2	4	0.01	0.01	2.85E-01
SORD	4	0.02	0.03	4.37E-01
SORT1	16	-0.05	0.01	1.63E-04
SOST	14	0.01	0.02	5.87E-01
SPARC	15	-0.02	0.02	2.79E-01
SPARCL1	4	0.00	0.01	5.03E-01
SPINK1	2	-0.01	0.01	5.21E-01
SPINK4	5	-0.01	0.01	8.35E-02
SPINK5	7	-0.06	0.01	3.25E-07
SPINK6	1	-0.01	0.01	3.49E-01
SPINT1	8	-0.01	0.01	2.93E-01
SPINT2	16	0.01	0.01	4.87E-01
SPOCK1	13	0.00	0.02	8.05E-01
SPON1	7	0.04	0.01	4.36E-03
SPON2	1	0.01	0.02	7.33E-01
SPP1	7	-0.01	0.03	7.15E-01
SPRY2	7	0.00	0.02	9.97E-01
SRC	1	0.23	0.09	7.76E-03
SRP14	2	-0.05	0.04	2.02E-01
SRPK2	4	-0.02	0.03	5.18E-01
SSC4D	11	0.01	0.01	5.94E-01
SSC5D	11	0.02	0.01	1.67E-01
ST3GAL1	8	0.01	0.01	5.86E-01

ST6GAL1	9	-0.04	0.02	1.02E-01
STAMBP	3	-0.04	0.05	4.22E-01
STAT5B	3	-0.01	0.05	8.08E-01
STC1	6	-0.13	0.03	5.97E-07
STC2	7	0.00	0.04	9.41E-01
STIP1	2	-0.03	0.05	4.77E-01
STK11	4	-0.03	0.04	4.21E-01
STK24	2	-0.06	0.06	3.69E-01
STK4	4	0.00	0.04	9.76E-01
STX16	1	-0.06	0.08	4.46E-01
STX4	2	-0.09	0.06	1.48E-01
STX6	2	-0.04	0.07	5.23E-01
STX8	3	-0.03	0.04	5.20E-01
SUGT1	2	-0.05	0.07	4.53E-01
SULT1A1	1	-0.01	0.05	7.83E-01
SULT2A1	4	-0.01	0.02	5.30E-01
SUMF2	15	-0.01	0.01	1.26E-01
SUSD1	5	0.00	0.03	9.56E-01
SUSD2	4	0.01	0.01	3.24E-01
TACC3	4	-0.02	0.03	5.58E-01
TACSTD2	14	-0.01	0.01	1.83E-01
TAFA5	2	0.05	0.03	9.79E-02
TARBP2	3	-0.01	0.04	8.10E-01
TBC1D17	1	0.00	0.01	9.63E-01
TBC1D23	4	-0.05	0.03	5.49E-02
TBC1D5	2	-0.03	0.06	5.53E-01
TBCB	4	-0.04	0.04	3.31E-01
TBCC	4	0.00	0.03	9.85E-01
TBL1X	6	0.00	0.03	8.95E-01
TCL1A	21	-0.02	0.01	1.06E-01
TCN2	3	0.00	0.01	9.47E-01
TDGF1	2	-1.35	0.10	1.18E-44
TDRKH	4	-0.01	0.01	5.35E-01
TEK	11	0.03	0.01	6.09E-05
TFF1	5	0.02	0.01	1.32E-01
TFF2	8	0.08	0.02	6.27E-05
TFF3	3	0.02	0.01	1.61E-01
TFPI	8	-0.01	0.01	3.79E-01
TFPI2	9	-0.01	0.02	7.78E-01
TFRC	10	-0.02	0.01	3.85E-02
TGFA	4	-0.09	0.03	6.50E-04
TGFB1	10	-0.02	0.02	1.39E-01
TGFBI	10	0.00	0.01	8.11E-01
TGFBR2	8	-0.25	0.02	8.33E-27
TGFBR3	7	0.00	0.02	9.05E-01
TGM2	2	0.02	0.03	5.01E-01
THBD	15	0.03	0.01	5.26E-02
THBS2	7	-0.01	0.01	1.64E-01
THBS4	6	0.05	0.01	1.87E-03
THOP1	1	-0.02	0.02	2.61E-01
THPO	17	-0.02	0.02	1.45E-01
THY1	3	-0.02	0.01	1.55E-01
TIA1	3	0.03	0.04	4.92E-01
TIE1	12	0.03	0.01	5.52E-04
TIGAR	2	0.06	0.06	2.72E-01
TIMD4	25	0.00	0.01	6.49E-01
TIMP1	5	-0.01	0.03	6.62E-01
TIMP3	17	-0.01	0.01	3.37E-01
TIMP4	6	-0.03	0.01	9.30E-03
TINAGL1	9	-0.04	0.02	7.55E-02

TJAP1	2	-0.01	0.07	9.19E-01
TLR3	6	-0.01	0.00	9.43E-02
TMPRSS15	7	-0.09	0.01	3.19E-10
TMPRSS5	18	0.01	0.01	2.47E-01
TMSB10	6	-0.05	0.03	8.39E-02
TNC	15	0.02	0.01	8.04E-02
TNF	3	-0.05	0.02	5.19E-02
TNFAIP8	1	0.02	0.06	7.87E-01
TNFRSF10A	4	0.02	0.01	1.01E-01
TNFRSF10B	3	0.00	0.01	9.43E-01
TNFRSF10C	18	0.00	0.01	8.78E-01
TNFRSF11A	7	0.02	0.01	1.98E-02
TNFRSF11B	9	0.00	0.02	9.21E-01
TNFRSF12A	2	-0.08	0.06	1.56E-01
TNFRSF13B	12	0.00	0.02	8.40E-01
TNFRSF13C	12	0.01	0.02	6.26E-01
TNFRSF14	5	-0.04	0.03	1.35E-01
TNFRSF19	4	0.00	0.02	8.16E-01
TNFRSF1A	7	0.11	0.03	7.20E-05
TNFRSF1B	6	-0.06	0.02	7.13E-03
TNFRSF21	12	0.00	0.02	9.84E-01
TNFRSF4	6	-0.04	0.02	1.05E-01
TNFRSF6B	4	-0.02	0.02	2.77E-01
TNFRSF8	8	-0.01	0.02	4.30E-01
TNFRSF9	6	0.05	0.03	5.70E-02
TNFSF10	12	-0.01	0.01	9.68E-02
TNFSF11	8	-0.04	0.01	2.39E-03
TNFSF12	17	0.01	0.01	2.03E-01
TNFSF13	7	0.00	0.01	9.39E-01
TNFSF13B	6	0.01	0.03	6.11E-01
TNFSF14	5	0.01	0.02	5.79E-01
TNR	17	0.02	0.01	8.67E-02
TNXB	6	0.01	0.01	3.39E-01
TP53	1	-0.06	0.08	4.36E-01
TP53INP1	2	-0.02	0.04	6.77E-01
TPMT	2	-0.01	0.01	2.56E-01
TPP1	13	-0.01	0.02	3.33E-01
TPPP3	1	-0.01	0.07	8.37E-01
TPSAB1	14	-0.01	0.01	4.04E-01
TRAF2	3	-0.01	0.05	7.57E-01
TREM2	7	-0.01	0.01	5.21E-01
TREML2	8	-0.01	0.02	6.59E-01
TRIAP1	7	-0.05	0.02	5.46E-02
TRIM21	3	-0.05	0.05	3.23E-01
TRIM5	1	0.02	0.02	1.98E-01
TSHB	18	0.00	0.02	8.53E-01
TSPAN1	3	0.10	0.05	7.69E-02
TST	1	-0.08	0.05	1.14E-01
TXLNA	3	-0.01	0.04	8.37E-01
TXNDC15	10	0.00	0.01	4.26E-01
TXNDC5	4	0.04	0.03	2.24E-01
TXNRD1	5	0.02	0.04	6.26E-01
TYMP	2	-0.03	0.02	2.58E-01
TYRO3	8	0.01	0.01	1.64E-01
UBAC1	1	-0.03	0.09	7.22E-01
ULBP2	8	0.06	0.01	2.88E-08
UMOD	16	0.00	0.01	7.34E-01
USO1	3	-0.02	0.04	7.32E-01
USP8	5	-0.01	0.03	8.24E-01
UXS1	7	0.00	0.02	8.49E-01

VAMP5	2	0.80	0.05	8.56E-50
VASH1	5	0.01	0.03	6.18E-01
VASN	5	-0.03	0.01	2.38E-02
VAT1	9	-0.03	0.02	6.57E-02
VCAM1	12	-0.03	0.02	1.37E-01
VCAN	15	0.01	0.01	4.44E-01
VEGFA	13	0.00	0.01	8.91E-01
VEGFC	17	0.01	0.02	4.28E-01
VEGFD	7	-0.05	0.02	2.06E-02
VMO1	7	-0.02	0.01	1.10E-02
VNN2	10	0.01	0.01	3.56E-01
VPS37A	2	-0.05	0.06	3.79E-01
VPS53	2	0.02	0.06	6.81E-01
VSIG4	8	-0.03	0.03	3.05E-01
VSIR	9	0.00	0.01	8.80E-01
VSTM1	4	0.01	0.01	3.82E-01
VSTM2L	2	-0.01	0.02	6.46E-01
VTA1	4	0.01	0.04	7.67E-01
VTCN1	2	-0.30	0.04	4.18E-12
VWA1	7	0.01	0.02	3.96E-01
VWC2	3	0.02	0.01	5.41E-02
VWF	6	0.23	0.01	9.16E-62
WARS	3	-0.05	0.02	4.94E-03
WAS	3	-0.06	0.04	1.25E-01
WASF1	3	-0.01	0.02	6.10E-01
WASF3	1	0.00	0.03	9.46E-01
WFDC12	12	0.01	0.01	5.88E-01
WFDC2	3	-0.14	0.04	4.01E-04
WFIKKN1	14	-0.01	0.01	5.28E-01
WFIKKN2	4	0.02	0.01	3.01E-02
WIF1	14	-0.02	0.01	6.75E-02
WNT9A	2	-0.07	0.04	1.33E-01
WWP2	3	-0.03	0.02	2.25E-01
XCL1	11	0.00	0.01	9.96E-01
XG	1	0.00	0.09	9.56E-01
XPNPEP2	12	0.00	0.02	8.65E-01
XRCC4	1	0.04	0.07	5.27E-01
YES1	2	-0.09	0.05	7.74E-02
YTHDF3	1	-0.01	0.06	8.82E-01
ZBTB16	7	-0.01	0.02	3.18E-01
ZBTB17	2	-0.09	0.05	6.16E-02

Supplementary Table 5. Function of proteins affecting COVID-19 risk in MR analysis.

Protein	function
ADAM15	Disintegrin and metalloproteinase domain-containing protein 15; Active metalloproteinase with gelatinolytic and collagenolytic activity. Plays a role in the wound healing process. Mediates both heterotypic intraepithelial cell/T-cell interactions and homotypic T-cell aggregation. Inhibits beta-1 integrin-mediated cell adhesion and migration of airway smooth muscle cells. Suppresses cell motility on or towards fibronectin possibly by driving alpha-v/beta-1 integrin (ITAGV-ITGB1) cell surface expression via ERK1/2 inactivation. Cleaves E-cadherin in response to growth factor deprivation. [...]
ADGRG1	Adhesion G-protein coupled receptor G1; Receptor involved in cell adhesion and probably in cell- cell interactions. Mediates cell matrix adhesion in developing neurons and hematopoietic stem cells. Receptor for collagen III/COL3A1 in the developing brain and involved in regulation of cortical development, specifically in maintenance of the pial basement membrane integrity and in cortical lamination (By similarity). Binding to the COL3A1 ligand inhibits neuronal migration and activates the RhoA pathway by coupling to GNA13 and possibly GNA12. Plays a role in the maintenance of hematopoi [...]
ADGRG2	Adhesion G-protein coupled receptor G2; Orphan receptor. Could be involved in a signal transduction pathway controlling epididymal function and male fertility. May regulate fluid exchange within epididymis
AMY2A	Pancreatic alpha-amylase; Amylase, alpha 2A
AMY2B	Amylase, alpha 2B; Belongs to the glycosyl hydrolase 13 family
BST2	Bone marrow stromal antigen 2; IFN-induced antiviral host restriction factor which efficiently blocks the release of diverse mammalian enveloped viruses by directly tethering nascent virions to the membranes of infected cells. Acts as a direct physical tether, holding virions to the cell membrane and linking virions to each other. The tethered virions can be internalized by endocytosis and subsequently degraded or they can remain on the cell surface. In either case, their spread as cell-free virions is restricted. Its target viruses belong to diverse families, including retroviridae: h [...]
CA4	Carbonic anhydrase 4; Reversible hydration of carbon dioxide. May stimulate the sodium/bicarbonate transporter activity of SLC4A4 that acts in pH homeostasis. It is essential for acid overload removal from the retina and retina epithelium, and acid release in the choriocapillaris in the choroid; Belongs to the alpha-carbonic anhydrase family
CCL15	C-C motif chemokine 15; Chemotactic factor that attracts T-cells and monocytes, but not neutrophils, eosinophils, or B-cells. Acts mainly via CC chemokine receptor CCR1. Also binds to CCR3. CCL15(22-92), CCL15(25-92) and CCL15(29-92) are more potent chemoattractants than the small-inducible cytokine A15; Belongs to the intercrine beta (chemokine CC) family
CCL28	C-C motif chemokine 28; Chemotactic activity for resting CD4, CD8 T-cells and eosinophils. Binds to CCR3 and CCR10 and induces calcium mobilization in a dose-dependent manner; Chemokine ligands

CD109	CD109 antigen; Modulates negatively TGFB1 signaling in keratinocytes; Belongs to the protease inhibitor I39 (alpha-2-macroglobulin) family
CD209	CD209 antigen; Pathogen-recognition receptor expressed on the surface of immature dendritic cells (DCs) and involved in initiation of primary immune response. Thought to mediate the endocytosis of pathogens which are subsequently degraded in lysosomal compartments. The receptor returns to the cell membrane surface and the pathogen-derived antigens are presented to resting T-cells via MHC class II proteins to initiate the adaptive immune response; C-type lectin domain containing
CD34	Hematopoietic progenitor cell antigen CD34; Possible adhesion molecule with a role in early hematopoiesis by mediating the attachment of stem cells to the bone marrow extracellular matrix or directly to stromal cells. Could act as a scaffold for the attachment of lineage specific glycans, allowing stem cells to bind to lectins expressed by stromal cells or other marrow components. Presents carbohydrate ligands to selectins; CD molecules
CD58	Lymphocyte function-associated antigen 3; Ligand of the T-lymphocyte CD2 glycoprotein. This interaction is important in mediating thymocyte interactions with thymic epithelial cells, antigen-independent and -dependent interactions of T-lymphocytes with target cells and antigen- presenting cells and the T-lymphocyte rosetting with erythrocytes. In addition, the LFA-3/CD2 interaction may prime response by both the CD2+ and LFA-3+ cells
CDH15	Cadherin-15; Cadherins are calcium-dependent cell adhesion proteins. They preferentially interact with themselves in a homophilic manner in connecting cells; cadherins may thus contribute to the sorting of heterogeneous cell types. M-cadherin is part of the myogenic program and may provide a trigger for terminal muscle differentiation
CDH17	Cadherin-17; Cadherins are calcium-dependent cell adhesion proteins. They preferentially interact with themselves in a homophilic manner in connecting cells; cadherins may thus contribute to the sorting of heterogeneous cell types. LI-cadherin may have a role in the morphological organization of liver and intestine. Involved in intestinal peptide transport
CLEC14A	C-type lectin domain family 14 member a; C-type lectin domain containing 14A
CTSS	Cathepsin S; Thiol protease. Key protease responsible for the removal of the invariant chain from MHC class II molecules. The bond-specificity of this proteinase is in part similar to the specificities of cathepsin L and cathepsin N; Cathepsins

CX3CL1	C-X3-C motif chemokine ligand 1; Fractalkine; Acts as a ligand for both CX3CR1 and integrins. Binds to CX3CR1. Binds to integrins ITGAV:ITGB3 and ITGA4:ITGB1. Can activate integrins in both a CX3CR1-dependent and CX3CR1-independent manner. In the presence of CX3CR1, activates integrins by binding to the classical ligand-binding site (site 1) in integrins. In the absence of CX3CR1, binds to a second site (site 2) in integrins which is distinct from site 1 and enhances the binding of other integrin ligands to site 1. The soluble form is chemotactic for T-cells and monocytes and not for n [...]
DPP10	Inactive dipeptidyl peptidase 10; Promotes cell surface expression of the potassium channel KCND2. Modulates the activity and gating characteristics of the potassium channel KCND2. Has no dipeptidyl aminopeptidase activity; DASH family
DRAXIN	Draxin; Chemorepulsive axon guidance protein required for the development of spinal cord and forebrain commissures. Acts as a chemorepulsive guidance protein for commissural axons during development. Able to inhibit or repel neurite outgrowth from dorsal spinal cord. Inhibits the stabilization of cytosolic beta- catenin (CTNNB1) via its interaction with LRP6, thereby acting as an antagonist of Wnt signaling pathway; Belongs to the draxin family
EFNA1	Ephrin-A1; Cell surface GPI-bound ligand for Eph receptors, a family of receptor tyrosine kinases which are crucial for migration, repulsion and adhesion during neuronal, vascular and epithelial development. Binds promiscuously Eph receptors residing on adjacent cells, leading to contact-dependent bidirectional signaling into neighboring cells. Plays an important role in angiogenesis and tumor neovascularization. The recruitment of VAV2, VAV3 and PI3-kinase p85 subunit by phosphorylated EPHA2 is critical for EFNA1-induced RAC1 GTPase activation and vascular endothelial cell migration a [...]
ERBB4	Receptor tyrosine-protein kinase erbB-4; Tyrosine-protein kinase that plays an essential role as cell surface receptor for neuregulins and EGF family members and regulates development of the heart, the central nervous system and the mammary gland, gene transcription, cell proliferation, differentiation, migration and apoptosis. Required for normal cardiac muscle differentiation during embryonic development, and for postnatal cardiomyocyte proliferation. Required for normal development of the embryonic central nervous system, especially for normal neural crest cell migration and normal [...]
F2R	Coagulation factor ii (thrombin) receptor; Proteinase-activated receptor 1; High affinity receptor for activated thrombin coupled to G proteins that stimulate phosphoinositide hydrolysis. May play a role in platelets activation and in vascular development; F2R receptors

FCGR2B	Low affinity immunoglobulin gamma Fc region receptor II-b; Receptor for the Fc region of complexed or aggregated immunoglobulins gamma. Low affinity receptor. Involved in a variety of effector and regulatory functions such as phagocytosis of immune complexes and modulation of antibody production by B-cells. Binding to this receptor results in down-modulation of previous state of cell activation triggered via antigen receptors on B-cells (BCR), T-cells (TCR) or via another Fc receptor. Isoform IIB1 fails to mediate endocytosis or phagocytosis. Isoform IIB2 does not trigger phagocytosis [...]
FGF19	Fibroblast growth factor 19; Involved in the suppression of bile acid biosynthesis through down-regulation of CYP7A1 expression, following positive regulation of the JNK and ERK1/2 cascades. Stimulates glucose uptake in adipocytes. Activity requires the presence of KLB and FGFR4; Belongs to the heparin-binding growth factors family
FGFR2	Fibroblast growth factor receptor 2; Tyrosine-protein kinase that acts as cell-surface receptor for fibroblast growth factors and plays an essential role in the regulation of cell proliferation, differentiation, migration and apoptosis, and in the regulation of embryonic development. Required for normal embryonic patterning, trophoblast function, limb bud development, lung morphogenesis, osteogenesis and skin development. Plays an essential role in the regulation of osteoblast differentiation, proliferation and apoptosis, and is required for normal skeleton development. Promotes cell p [...]
FLT4	Vascular endothelial growth factor receptor 3; Tyrosine-protein kinase that acts as a cell-surface receptor for VEGFC and VEGFD, and plays an essential role in adult lymphangiogenesis and in the development of the vascular network and the cardiovascular system during embryonic development. Promotes proliferation, survival and migration of endothelial cells, and regulates angiogenic sprouting. Signaling by activated FLT4 leads to enhanced production of VEGFC, and to a lesser degree VEGFA, thereby creating a positive feedback loop that enhances FLT4 signaling. Modulates KDR signaling by [...]
FOLR1	Folate receptor alpha; Binds to folate and reduced folic acid derivatives and mediates delivery of 5-methyltetrahydrofolate and folate analogs into the interior of cells. Has high affinity for folate and folic acid analogs at neutral pH. Exposure to slightly acidic pH after receptor endocytosis triggers a conformation change that strongly reduces its affinity for folates and mediates their release. Required for normal embryonic development and normal cell proliferation
GKN1	Gastrokine-1; Has mitogenic activity and may be involved in maintaining the integrity of the gastric mucosal epithelium; BRICHOS domain containing
GOLM2	Golgi membrane protein 2; Protein CASC4; Cancer susceptibility 4; Belongs to the GOLM1/CASC4 family
GP2	Pancreatic secretory granule membrane major glycoprotein GP2; Glycoprotein 2

ICAM2	Intercellular adhesion molecule 2; ICAM proteins are ligands for the leukocyte adhesion protein LFA-1 (integrin alpha-L/beta-2). ICAM2 may play a role in lymphocyte recirculation by blocking LFA-1-dependent cell adhesion. It mediates adhesive interactions important for antigen- specific immune response, NK-cell mediated clearance, lymphocyte recirculation, and other cellular interactions important for immune response and surveillance; CD molecules
ICAM5	Intercellular adhesion molecule 5; ICAM proteins are ligands for the leukocyte adhesion protein LFA-1 (integrin alpha-L/beta-2); Ig-like cell adhesion molecule family
IDS	Iduronate 2-sulfatase; Required for the lysosomal degradation of heparan sulfate and dermatan sulfate; Sulfatases
ISLR2	Immunoglobulin superfamily containing leucine-rich repeat protein 2; Required for axon extension during neural development; Immunoglobulin like domain containing
ITGA6	Integrin subunit alpha 6; Integrin alpha-6; Integrin alpha-6/beta-1 is a receptor for laminin on platelets. Integrin alpha-6/beta-4 is a receptor for laminin in epithelial cells and it plays a critical structural role in the hemidesmosome (By similarity). ITGA6:ITGB4 binds to NRG1 (via EGF domain) and this binding is essential for NRG1-ERBB signaling. ITGA6:ITGB4 binds to IGF1 and this binding is essential for IGF1 signaling
ITGB1	Integrin beta-1; Integrins alpha-1/beta-1, alpha-2/beta-1, alpha-10/beta- 1 and alpha-11/beta-1 are receptors for collagen. Integrins alpha- 1/beta-1 and alpha-2/beta-2 recognize the proline-hydroxylated sequence G-F-P-G-E-R in collagen. Integrins alpha-2/beta-1, alpha- 3/beta-1, alpha-4/beta-1, alpha-5/beta-1, alpha-8/beta-1, alpha- 10/beta-1, alpha-11/beta-1 and alpha-V/beta-1 are receptors for fibronectin. Alpha-4/beta-1 recognizes one or more domains within the alternatively spliced CS-1 and CS-5 regions of fibronectin. Integrin alpha-5/beta-1 is a receptor for fibrinogen. Integrin [...]
KEL	Kell blood group glycoprotein; Zinc endopeptidase with endothelin-3-converting enzyme activity. Cleaves EDN1, EDN2 and EDN3, with a marked preference for EDN3; Blood group antigens
KLK1	Kallikrein-1; Glandular kallikreins cleave Met-Lys and Arg-Ser bonds in kininogen to release Lys-bradykinin; Belongs to the peptidase S1 family. Kallikrein subfamily
LAMP3	Lysosome-associated membrane glycoprotein 3; May play a role in dendritic cell function and in adaptive immunity; CD molecules
LEFTY2	Left-right determination factor 2; Required for left-right (L-R) asymmetry determination of organ systems in mammals. May play a role in endometrial bleeding
LGALS4	Galectin-4; Galectin that binds lactose and a related range of sugars. May be involved in the assembly of adherens junctions; Galectins
LGALS8	Galectin 8; Galectins

MET	Hepatocyte growth factor receptor; Receptor tyrosine kinase that transduces signals from the extracellular matrix into the cytoplasm by binding to hepatocyte growth factor/HGF ligand. Regulates many physiological processes including proliferation, scattering, morphogenesis and survival. Ligand binding at the cell surface induces autophosphorylation of MET on its intracellular domain that provides docking sites for downstream signaling molecules. Following activation by ligand, interacts with the PI3-kinase subunit PIK3R1, PLCG1, SRC, GRB2, STAT3 or the adapter GAB1. Recruitment of thes [...]
MNDA	Myeloid cell nuclear differentiation antigen; May act as a transcriptional activator/repressor in the myeloid lineage. Plays a role in the granulocyte/monocyte cell- specific response to interferon. Stimulates the DNA binding of the transcriptional repressor protein YY1; Pyrin and HIN domain family
MUC13	Mucin-13; Epithelial and hemopoietic transmembrane mucin that may play a role in cell signaling; Mucins
NELL2	Protein kinase C-binding protein NELL2; Required for neuron survival through the modulation of MAPK pathways (By similarity). Involved in the regulation of hypothalamic GNRH secretion and the control of puberty (By similarity)
NME3	Nucleoside diphosphate kinase 3; Major role in the synthesis of nucleoside triphosphates other than ATP. The ATP gamma phosphate is transferred to the NDP beta phosphate via a ping-pong mechanism, using a phosphorylated active-site intermediate. Probably has a role in normal hematopoiesis by inhibition of granulocyte differentiation and induction of apoptosis; NME/NM23 family
NRCAM	Neuronal cell adhesion molecule; Cell adhesion protein that is required for normal responses to cell-cell contacts in brain and in the peripheral nervous system. Plays a role in neurite outgrowth in response to contactin binding. Plays a role in mediating cell-cell contacts between Schwann cells and axons. Plays a role in the formation and maintenance of the nodes of Ranvier on myelinated axons. Nodes of Ranvier contain clustered sodium channels that are crucial for the saltatory propagation of action potentials along myelinated axons. During development, nodes of Ranvier are formed by [...]
PECAM1	Platelet endothelial cell adhesion molecule; Cell adhesion molecule which is required for leukocyte transendothelial migration (TEM) under most inflammatory conditions. Tyr-690 plays a critical role in TEM and is required for efficient trafficking of PECAM1 to and from the lateral border recycling compartment (LBRC) and is also essential for the LBRC membrane to be targeted around migrating leukocytes. Heterophilic interaction with CD177 plays a role in transendothelial migration of neutrophils. Homophilic ligation of PECAM1 prevents macrophage-mediated phagocytosis of neighboring viab [...]

PLAT	Plasminogen activator, tissue type; Tissue-type plasminogen activator; Converts the abundant, but inactive, zymogen plasminogen to plasmin by hydrolyzing a single Arg-Val bond in plasminogen. By controlling plasmin-mediated proteolysis, it plays an important role in tissue remodeling and degradation, in cell migration and many other physiopathological events. Plays a direct role in facilitating neuronal migration
PODXL	Podocalyxin; Involved in the regulation of both adhesion and cell morphology and cancer progression. Function as an anti-adhesive molecule that maintains an open filtration pathway between neighboring foot processes in the podocyte by charge repulsion. Acts as a pro-adhesive molecule, enhancing the adherence of cells to immobilized ligands, increasing the rate of migration and cell- cell contacts in an integrin-dependent manner. Induces the formation of apical actin-dependent microvilli. Involved in the formation of a preapical plasma membrane subdomain to set up inital epithelial pola [...]
PRSS27	Serine protease 27
PTPRM	Receptor-type tyrosine-protein phosphatase mu; Involved in cell-cell adhesion through homophilic interactions. May play a key role in signal transduction and growth control; Fibronectin type III domain containing
REG1A	Lithostathine-1-alpha; Might act as an inhibitor of spontaneous calcium carbonate precipitation. May be associated with neuronal sprouting in brain, and with brain and pancreas regeneration; C-type lectin domain containing
REG1B	Lithostathine-1-beta; Might act as an inhibitor of spontaneous calcium carbonate precipitation. May be associated with neuronal sprouting in brain, and with brain and pancreas regeneration; C-type lectin domain containing
S100A16	Protein S100-A16; Calcium-binding protein. Binds one calcium ion per monomer. Can promote differentiation of adipocytes (in vitro) (By similarity). Overexpression in preadipocytes increases their proliferation, enhances adipogenesis and reduces insulin-stimulated glucose uptake (By similarity); Belongs to the S-100 family
SCARF2	Scavenger receptor class F member 2; Probable adhesion protein, which mediates homophilic and heterophilic interactions. In contrast to SCARF1, it poorly mediates the binding and degradation of acetylated low density lipoprotein (Ac-LDL) (By similarity); Scavenger receptors
SCG2	Secretogranin-2; Secretogranin-2 is a neuroendocrine secretory granule protein, which is the precursor for biologically active peptides; Granins
SCGN	Secretagogin, EF-hand calcium binding protein; EF-hand domain containing
SELE	E-selectin; Cell-surface glycoprotein having a role in immunoadhesion. Mediates in the adhesion of blood neutrophils in cytokine-activated endothelium through interaction with PSGL1/SELPLG. May have a role in capillary morphogenesis; Belongs to the selectin/LECAM family
SEMA3F	Semaphorin-3F; May play a role in cell motility and cell adhesion; Immunoglobulin like domain containing

SEMA4C	Semaphorin-4C; Cell surface receptor for PLXNB2 that plays an important role in cell-cell signaling. PLXNB2 binding promotes downstream activation of RHOA and phosphorylation of ERBB2 at 'Tyr-1248'. Required for normal brain development, axon guidance and cell migration (By similarity). Probable signaling receptor which may play a role in myogenic differentiation through activation of the stress-activated MAPK cascade; Belongs to the semaphorin family
SFTPA2	Pulmonary surfactant-associated protein A2; In presence of calcium ions, it binds to surfactant phospholipids and contributes to lower the surface tension at the air-liquid interface in the alveoli of the mammalian lung and is essential for normal respiration; C-type lectin domain containing
SFTPD	Pulmonary surfactant-associated protein D; Contributes to the lung's defense against inhaled microorganisms, organic antigens and toxins. Interacts with compounds such as bacterial lipopolysaccharides, oligosaccharides and fatty acids and modulates leukocyte action in immune response. May participate in the extracellular reorganization or turnover of pulmonary surfactant. Binds strongly maltose residues and to a lesser extent other alpha-glucosyl moieties; C-type lectin domain containing
SLITRK2	Slit and ntrk like family member 2; SLIT and NTRK-like protein 2; It is involved in synaptogenesis and promotes excitatory synapse differentiation. Suppresses neurite outgrowth (By similarity)
SPINK5	Serine protease inhibitor Kazal-type 5; Serine protease inhibitor, probably important for the anti-inflammatory and/or antimicrobial protection of mucous epithelia. Contribute to the integrity and protective barrier function of the skin by regulating the activity of defense- activating and desquamation-involved proteases. Inhibits KLK5, it's major target, in a pH-dependent manner. Inhibits KLK7, KLK14 CASP14, and trypsin; Serine peptidase inhibitors, Kazal type
STC1	Stanniocalcin-1; Stimulates renal phosphate reabsorption, and could therefore prevent hypercalcemia
TDGF1	Teratocarcinoma-derived growth factor 1; GPI-anchored cell membrane protein involved in Nodal signaling. Cell-associated TDGF1 acts as a Nodal coreceptor in cis. Shedding of TDGF1 by TMEM8A modulates Nodal signaling by allowing soluble TDGF1 to act as a Nodal coreceptor on other cells. Could play a role in the determination of the epiblastic cells that subsequently give rise to the mesoderm
TGFBR2	Transforming growth factor beta receptor 2; TGF-beta receptor type-2; Transmembrane serine/threonine kinase forming with the TGF-beta type I serine/threonine kinase receptor, TGFBR1, the non-promiscuous receptor for the TGF-beta cytokines TGFB1, TGFB2 and TGFB3. Transduces the TGFB1, TGFB2 and TGFB3 signal from the cell surface to the cytoplasm and is thus regulating a plethora of physiological and pathological processes including cell cycle arrest in epithelial and hematopoietic cells, control of mesenchymal cell proliferation and differentiation, wound healing, extracellular matrix [...]
ULBP2	UL16-binding protein 2; Binds and activates the KLRK1/NKG2D receptor, mediating natural killer cell cytotoxicity; Belongs to the MHC class I family

VAMP5	Vesicle-associated membrane protein 5; May participate in trafficking events that are associated with myogenesis, such as myoblast fusion and/or GLUT4 trafficking; Belongs to the synaptobrevin family
VTCN1	V-set domain-containing T-cell activation inhibitor 1; Negatively regulates T-cell-mediated immune response by inhibiting T-cell activation, proliferation, cytokine production and development of cytotoxicity. When expressed on the cell surface of tumor macrophages, plays an important role, together with regulatory T-cells (Treg), in the suppression of tumor- associated antigen-specific T-cell immunity. Involved in promoting epithelial cell transformation; Immunoglobulin like domain containing

Supplementary Table 15. Associations of genetically predicted proteins with healthspan using IVW method and cis+trans SNPs.

Protein	No. of SNPs	Beta	SE	Pval
AARSD1	3	-0.08	0.33	8.06E-01
ABHD14B	3	-0.11	0.50	8.33E-01
ABL1	1	-0.04	0.59	9.44E-01
ACAA1	3	-0.36	0.39	3.56E-01
ACAN	13	-0.65	0.20	1.14E-03
ACE2	8	0.22	0.25	3.80E-01
ACOX1	1	0.94	0.69	1.76E-01
ACP5	10	-0.06	0.13	6.40E-01
ACP6	2	0.09	0.05	7.53E-02
ACVRL1	3	-0.03	0.15	8.60E-01
ACY1	3	-0.22	0.21	2.93E-01
ADA	4	-0.03	0.08	7.36E-01
ADA2	13	-0.39	0.15	8.35E-03
ADAM15	8	0.05	0.06	4.49E-01
ADAM22	6	0.02	0.08	8.58E-01
ADAM23	5	0.04	0.09	6.66E-01
ADAM8	7	-0.22	0.10	3.24E-02
ADAMTS13	10	0.10	0.12	3.81E-01
ADAMTS15	4	0.22	0.32	4.78E-01
ADAMTS16	1	0.13	0.43	7.66E-01
ADAMTS8	3	0.25	0.11	2.18E-02
ADGRB3	4	0.00	0.05	9.54E-01
ADGRE2	9	-0.38	0.14	5.33E-03
ADGRE5	9	-0.47	0.18	8.21E-03
ADGRG1	9	-0.14	0.21	5.07E-01
ADGRG2	5	-0.83	0.33	1.24E-02
ADH4	6	-0.48	0.26	6.73E-02
ADM	1	-1.04	1.03	3.14E-01
AFP	19	0.27	0.11	1.43E-02
AGER	12	0.21	0.17	2.26E-01
AGR2	4	-0.65	0.34	5.13E-02
AGR3	2	-0.65	0.53	2.20E-01
AGRN	6	-0.04	0.12	7.77E-01
AGRP	9	0.13	0.25	6.03E-01
AGXT	11	-0.26	0.15	7.57E-02
AHCY	1	0.01	0.14	9.46E-01
AHSP	8	-0.32	0.25	1.96E-01
AIF1	1	0.25	0.44	5.66E-01
AIFM1	2	0.19	0.66	7.73E-01
AKR1B1	3	-0.03	0.31	9.13E-01
AKR1C4	1	0.95	0.94	3.10E-01
AKT1S1	3	-0.15	0.45	7.43E-01
AKT3	2	0.12	0.66	8.59E-01
ALCAM	11	0.07	0.19	7.09E-01
ALDH1A1	3	0.02	0.38	9.51E-01
ALDH3A1	1	-0.05	0.11	6.17E-01
ALPP	10	0.07	0.10	5.26E-01
AMBP	9	0.25	0.18	1.76E-01
AMIGO2	12	0.34	0.13	9.47E-03
AMN	5	0.03	0.07	6.07E-01
AMY2A	7	0.28	0.12	2.25E-02
AMY2B	8	0.14	0.11	2.15E-01

ANG	3	0.02	0.11	8.69E-01
ANGPT1	17	0.22	0.14	1.26E-01
ANGPT2	6	-0.15	0.20	4.54E-01
ANGPTL1	6	0.16	0.16	2.92E-01
ANGPTL2	5	-0.10	0.29	7.25E-01
ANGPTL3	5	0.59	0.33	7.59E-02
ANGPTL4	1	-0.16	0.18	3.75E-01
ANGPTL7	6	-0.19	0.11	1.03E-01
ANKRD54	1	-1.01	0.73	1.66E-01
ANPEP	7	-0.03	0.12	8.15E-01
ANXA11	2	-0.33	0.60	5.76E-01
ANXA3	5	-0.22	0.33	5.04E-01
ANXA4	2	-0.18	0.57	7.57E-01
ANXA5	1	-1.10	0.45	1.43E-02
AOC1	1	0.12	0.18	5.06E-01
AOC3	10	0.04	0.09	6.46E-01
APBB1IP	5	-0.13	0.17	4.46E-01
APEX1	3	0.08	0.12	4.89E-01
APLP1	4	-0.79	0.34	2.19E-02
APOH	1	0.08	0.10	4.01E-01
APOM	8	0.12	0.15	4.15E-01
APP	15	0.27	0.16	9.42E-02
APRT	5	0.16	0.31	6.12E-01
AREG	3	0.03	0.26	9.02E-01
ARG1	5	0.16	0.21	4.28E-01
ARHGAP1	2	0.11	0.47	8.20E-01
ARHGAP25	2	-0.22	0.45	6.29E-01
ARSA	7	0.00	0.07	9.84E-01
ARSB	10	-0.14	0.14	2.92E-01
ART3	4	-0.23	0.42	5.82E-01
ARTN	1	-0.29	0.76	7.04E-01
ASAH2	15	0.00	0.08	9.93E-01
ASGR1	6	0.20	0.19	2.93E-01
ATF2	1	-0.13	0.41	7.59E-01
ATG4A	3	0.62	0.49	2.09E-01
ATOX1	3	0.50	0.49	3.13E-01
ATP5IF1	5	0.35	0.33	2.81E-01
ATP6V1F	1	-0.05	0.77	9.44E-01
ATXN10	5	0.10	0.31	7.59E-01
AXIN1	3	0.35	0.52	5.03E-01
AXL	6	-0.33	0.18	6.58E-02
AZU1	2	-0.83	0.61	1.71E-01
B4GALT1	7	-0.03	0.13	8.11E-01
B4GAT1	8	-0.05	0.14	7.22E-01
BACH1	3	0.41	0.53	4.40E-01
BAG3	4	0.69	0.35	4.58E-02
BAIAP2	5	-0.01	0.36	9.78E-01
BAMBI	1	0.05	0.75	9.44E-01
BANK1	3	0.20	0.33	5.52E-01
BAX	2	0.04	0.61	9.42E-01
BCAM	14	-0.16	0.10	1.19E-01
BCAN	7	-0.10	0.17	5.53E-01
BCL2L11	10	0.21	0.11	6.80E-02
BCR	1	0.88	0.78	2.55E-01

BGN	10	-0.23	0.21	2.76E-01
BID	2	-0.40	0.48	3.98E-01
BIN2	2	0.00	0.58	9.98E-01
BIRC2	4	0.57	0.45	2.03E-01
BLMH	3	0.01	0.09	8.96E-01
BLVRB	2	0.03	0.24	9.07E-01
BMP4	5	0.07	0.31	8.25E-01
BMP6	14	0.26	0.14	6.40E-02
BOC	8	0.13	0.21	5.61E-01
BPIFB1	14	0.00	0.10	1.00E+00
BRK1	6	-0.11	0.21	5.81E-01
BSG	10	-0.01	0.20	9.71E-01
BST1	4	-0.05	0.05	3.28E-01
BST2	11	-0.43	0.20	3.58E-02
BTC	3	0.02	0.12	8.55E-01
BTN2A1	6	-0.29	0.15	5.23E-02
BTN3A2	3	0.10	0.07	1.95E-01
C19orf12	5	0.42	0.32	1.87E-01
C1QA	13	-0.03	0.11	7.55E-01
C1QTNF1	4	0.22	0.18	2.29E-01
C2	2	-0.57	0.41	1.62E-01
C2CD2L	1	-1.11	0.90	2.19E-01
C4BPB	9	-0.03	0.14	8.45E-01
CA1	4	0.05	0.29	8.59E-01
CA11	2	-0.85	0.29	3.73E-03
CA12	6	-0.42	0.15	6.11E-03
CA13	3	-0.14	0.18	4.24E-01
CA14	4	0.16	0.24	5.10E-01
CA2	2	0.12	0.44	7.82E-01
CA3	6	-0.12	0.20	5.61E-01
CA4	9	0.08	0.13	5.15E-01
CA5A	5	0.01	0.08	9.05E-01
CA6	12	-0.03	0.07	6.47E-01
CA9	5	-0.55	0.23	1.40E-02
CALB1	3	-0.35	0.32	2.78E-01
CALCA	4	-0.05	0.35	8.88E-01
CALCOCO1	4	0.17	0.34	6.13E-01
CAMKK1	3	0.01	0.17	9.53E-01
CANT1	6	-0.66	0.24	5.86E-03
CAPG	2	-0.04	0.06	5.38E-01
CARHSP1	1	0.35	0.62	5.68E-01
CASP1	1	-0.11	0.19	5.64E-01
CASP10	3	0.46	0.23	4.97E-02
CASP2	2	-0.38	0.44	3.90E-01
CASP3	2	-0.02	0.37	9.53E-01
CASP8	3	0.37	0.21	7.82E-02
CBLIF	11	0.15	0.10	1.47E-01
CBLN4	5	0.10	0.21	6.47E-01
CC2D1A	2	-0.27	0.54	6.18E-01
CCDC80	5	-0.04	0.22	8.37E-01
CCL11	8	0.26	0.17	1.21E-01
CCL13	13	0.09	0.13	4.99E-01
CCL14	6	0.18	0.10	7.80E-02
CCL15	4	0.34	0.23	1.49E-01

CCL16	7	-0.06	0.06	3.71E-01
CCL17	13	0.28	0.10	7.70E-03
CCL18	3	-0.13	0.10	2.06E-01
CCL19	4	-0.37	0.33	2.72E-01
CCL2	8	0.13	0.15	3.74E-01
CCL20	5	-0.35	0.28	2.02E-01
CCL21	10	-0.24	0.21	2.53E-01
CCL22	7	-0.24	0.19	2.10E-01
CCL23	8	0.03	0.12	7.68E-01
CCL24	10	0.03	0.06	5.98E-01
CCL25	7	-0.10	0.06	1.21E-01
CCL26	6	0.02	0.16	9.02E-01
CCL27	7	0.27	0.19	1.43E-01
CCL28	18	0.41	0.14	3.13E-03
CCL3	6	-0.80	0.34	1.98E-02
CCL4	5	-0.09	0.12	4.36E-01
CCL5	9	0.15	0.17	3.96E-01
CCL7	4	0.26	0.15	7.75E-02
CCL8	9	-0.07	0.06	2.17E-01
CCN1	11	-0.58	0.21	5.02E-03
CCN2	17	0.18	0.12	1.25E-01
CCN3	3	0.14	0.20	4.88E-01
CCN4	12	-0.08	0.11	4.41E-01
CCN5	1	2.00	1.03	5.13E-02
CCS	1	-0.07	0.10	4.87E-01
CD109	6	0.16	0.21	4.46E-01
CD14	5	-0.23	0.16	1.40E-01
CD160	14	-0.02	0.10	8.20E-01
CD163	18	-0.23	0.10	3.18E-02
CD164	9	-0.14	0.20	4.98E-01
CD177	3	0.13	0.10	2.11E-01
CD1C	17	-0.35	0.13	8.19E-03
CD200	7	-0.27	0.19	1.56E-01
CD200R1	9	-0.04	0.06	4.65E-01
CD207	15	0.01	0.09	9.40E-01
CD209	8	-0.16	0.06	4.76E-03
CD22	19	-0.04	0.12	7.23E-01
CD244	13	-0.07	0.10	4.82E-01
CD27	7	-0.37	0.17	2.88E-02
CD274	7	-0.01	0.15	9.44E-01
CD276	7	-0.13	0.06	3.54E-02
CD28	9	-0.07	0.15	6.15E-01
CD2AP	1	-0.05	0.78	9.44E-01
CD300C	13	-0.24	0.10	2.16E-02
CD300E	10	-0.01	0.12	9.52E-01
CD300LF	9	-0.02	0.05	6.99E-01
CD300LG	12	0.28	0.11	1.14E-02
CD302	6	-0.25	0.15	9.23E-02
CD33	6	-0.19	0.39	6.22E-01
CD34	3	0.32	0.13	1.29E-02
CD38	2	0.05	0.14	7.12E-01
CD4	4	0.10	0.14	4.48E-01
CD40	9	0.17	0.12	1.64E-01
CD40LG	12	0.10	0.19	6.18E-01

CD46	7	0.36	0.28	2.05E-01
CD48	10	-0.10	0.07	1.43E-01
CD5	8	0.10	0.20	6.09E-01
CD55	6	0.01	0.11	8.91E-01
CD58	11	0.10	0.10	3.15E-01
CD59	5	0.22	0.19	2.55E-01
CD6	10	0.01	0.06	8.40E-01
CD63	8	0.07	0.19	7.03E-01
CD69	5	0.10	0.27	7.19E-01
CD70	3	-0.12	0.06	5.24E-02
CD74	6	-0.86	0.19	5.59E-06
CD79B	16	0.11	0.14	4.14E-01
CD83	12	-0.09	0.17	5.93E-01
CD84	11	-0.11	0.16	5.16E-01
CD8A	3	-0.08	0.18	6.65E-01
CD93	5	-0.37	0.35	2.84E-01
CD99	4	-0.14	0.34	6.74E-01
CD99L2	8	-0.06	0.20	7.59E-01
CDC27	2	-0.59	0.51	2.46E-01
CDC37	2	0.00	0.58	9.94E-01
CDCP1	7	-0.09	0.17	5.83E-01
CDH1	6	0.16	0.09	8.48E-02
CDH15	3	-0.12	0.16	4.24E-01
CDH17	11	0.17	0.08	3.51E-02
CDH2	13	0.24	0.19	2.26E-01
CDH3	7	-0.34	0.27	2.14E-01
CDH5	12	-0.03	0.08	7.01E-01
CDH6	6	-0.13	0.09	1.74E-01
CDHR1	4	-0.14	0.19	4.71E-01
CDHR2	7	-0.02	0.30	9.38E-01
CDHR5	8	-0.06	0.09	5.18E-01
CDKN1A	5	0.17	0.26	5.12E-01
CDKN2D	4	0.13	0.32	6.87E-01
CDNF	1	-0.06	0.12	6.42E-01
CDON	4	-0.57	0.31	7.15E-02
CDSN	18	-0.25	0.17	1.45E-01
CEACAM1	6	-0.20	0.16	2.31E-01
CEACAM21	2	-1.33	0.91	1.45E-01
CEACAM3	2	-0.50	0.41	2.25E-01
CEACAM5	10	-0.08	0.09	3.58E-01
CEACAM8	10	-0.27	0.17	1.14E-01
CEBPB	3	0.37	0.48	4.41E-01
CELA3A	14	-0.01	0.12	9.09E-01
CEP20	4	-0.15	0.30	6.20E-01
CEP43	6	0.39	0.34	2.46E-01
CEP85	2	0.07	0.37	8.58E-01
CERT	4	0.32	0.38	4.06E-01
CES1	16	-0.15	0.19	4.26E-01
CES2	2	-0.03	0.35	9.43E-01
CES3	9	-0.33	0.17	4.95E-02
CETN2	3	-0.16	0.40	6.86E-01
CFC1	6	1.07	0.34	1.42E-03
CGA	1	1.03	1.26	4.15E-01
CGREF1	3	0.12	0.08	1.33E-01

CHAC2	2	0.83	0.34	1.54E-02
CHGB	5	0.08	0.08	3.54E-01
CHI3L1	5	-0.15	0.09	7.56E-02
CHIT1	3	0.06	0.06	2.98E-01
CHL1	6	-0.02	0.14	8.66E-01
CHMP1A	4	-0.48	0.38	1.99E-01
CHRDL1	3	0.07	0.51	8.92E-01
CHRDL2	3	0.33	0.41	4.18E-01
CIAPIN1	4	0.08	0.43	8.43E-01
CKAP4	4	0.10	0.11	3.84E-01
KMT1A_CKMT1I	6	-0.32	0.27	2.40E-01
CLC	8	0.40	0.20	4.64E-02
CLEC10A	9	-0.17	0.09	4.31E-02
CLEC11A	5	0.28	0.16	8.27E-02
CLEC14A	8	0.20	0.15	1.72E-01
CLEC1A	7	0.00	0.10	9.70E-01
CLEC1B	9	0.04	0.19	8.17E-01
CLEC4A	12	-0.02	0.09	7.77E-01
CLEC4C	25	0.06	0.05	2.60E-01
CLEC4D	10	-0.03	0.08	6.74E-01
CLEC4G	11	-0.27	0.13	3.53E-02
CLEC5A	10	0.12	0.14	4.07E-01
CLEC6A	12	-0.15	0.09	7.51E-02
CLEC7A	3	-0.09	0.06	1.16E-01
CLIP2	2	0.14	0.36	6.92E-01
CLMP	4	-0.19	0.14	1.71E-01
CLPS	7	-0.03	0.09	7.45E-01
CLSPN	1	-0.30	0.48	5.42E-01
CLSTN2	3	0.11	0.12	3.87E-01
CLTA	1	0.01	0.43	9.87E-01
CLUL1	9	0.05	0.09	5.82E-01
CNDP1	7	-0.27	0.11	1.43E-02
CNPY2	1	0.49	0.36	1.77E-01
CNPY4	3	0.04	0.26	8.92E-01
CNST	4	0.26	0.47	5.89E-01
CNTN1	6	-0.14	0.13	2.77E-01
CNTN2	6	-0.08	0.06	1.86E-01
CNTN3	15	0.14	0.13	2.86E-01
CNTN4	9	0.13	0.15	3.82E-01
CNTN5	6	-0.01	0.11	9.32E-01
CNTNAP2	4	-0.02	0.09	8.52E-01
COL18A1	4	0.13	0.26	6.17E-01
COL1A1	9	0.36	0.22	1.07E-01
COL4A1	9	0.28	0.14	4.40E-02
COL6A3	4	-0.06	0.36	8.67E-01
COL9A1	4	-0.12	0.26	6.39E-01
COLEC12	4	0.20	0.30	5.01E-01
COMP	6	-0.43	0.17	1.26E-02
COMT	2	0.02	0.11	8.47E-01
CORO1A	3	-0.11	0.47	8.20E-01
COX5B	5	0.23	0.29	4.31E-01
CPA1	17	-0.07	0.14	6.01E-01
CPA2	4	-0.17	0.13	1.79E-01
CPB1	10	0.08	0.15	5.70E-01

CPE	7	0.30	0.17	7.59E-02
CPM	9	-0.67	0.24	4.69E-03
CPPED1	1	-0.04	0.08	6.46E-01
CPVL	11	-0.09	0.07	1.81E-01
CPXM1	18	0.18	0.11	1.09E-01
CR2	22	-0.07	0.13	6.02E-01
CRACR2A	4	-0.35	0.30	2.48E-01
CRADD	4	0.29	0.42	4.90E-01
CREG1	10	-0.03	0.17	8.60E-01
CRELD2	11	-0.21	0.13	1.23E-01
CRH	20	0.00	0.11	9.64E-01
CRHBP	5	-0.12	0.08	1.49E-01
CRIM1	4	0.61	0.36	8.68E-02
CRIP2	3	-0.26	0.28	3.45E-01
CRISP2	12	-0.06	0.09	5.42E-01
CRKL	1	0.06	0.78	9.34E-01
CRLF1	6	-0.11	0.34	7.47E-01
CRNN	3	-0.04	0.08	6.56E-01
CRTAC1	2	-0.01	0.06	9.22E-01
CRTAM	12	0.00	0.13	9.98E-01
CSF1	3	-0.26	0.24	2.72E-01
CSF2RA	1	0.49	0.64	4.47E-01
CSF3	2	-0.01	0.26	9.82E-01
CST3	6	-0.03	0.17	8.74E-01
CST5	13	-0.02	0.06	7.66E-01
CST6	14	0.11	0.15	4.78E-01
CST7	10	-0.01	0.07	8.25E-01
CTF1	2	0.16	0.66	8.13E-01
CTRB1	11	0.00	0.09	9.59E-01
CTRC	11	0.00	0.08	9.87E-01
CTSB	7	0.12	0.09	1.79E-01
CTSC	7	-0.07	0.08	4.10E-01
CTSD	6	-0.15	0.14	2.81E-01
CTSF	14	-0.13	0.13	2.92E-01
CTSH	1	-0.32	0.57	5.78E-01
CTSL	16	-0.30	0.15	4.80E-02
CTSO	9	-0.43	0.17	1.34E-02
CTSS	7	0.19	0.27	4.90E-01
CTSV	17	0.28	0.14	5.08E-02
CTSZ	9	0.07	0.10	4.59E-01
CX3CL1	5	0.15	0.33	6.46E-01
CXADR	5	0.08	0.20	6.88E-01
CXCL1	4	0.04	0.08	6.11E-01
CXCL10	3	-0.13	0.16	4.22E-01
CXCL11	6	-0.32	0.20	9.97E-02
CXCL12	9	-0.16	0.20	4.30E-01
CXCL13	7	-0.60	0.29	3.87E-02
CXCL14	2	-0.58	0.56	2.98E-01
CXCL16	11	-0.12	0.16	4.76E-01
CXCL17	5	-0.04	0.36	9.20E-01
CXCL3	3	0.88	0.40	2.92E-02
CXCL5	9	-0.04	0.10	7.01E-01
CXCL6	7	0.00	0.09	9.54E-01
CXCL8	7	0.15	0.20	4.48E-01

CXCL9	5	-0.49	0.24	4.21E-02
DAB2	3	0.15	0.48	7.55E-01
DAG1	3	0.41	0.55	4.56E-01
DAPP1	2	-1.02	0.42	1.56E-02
DARS1	5	0.28	0.33	4.02E-01
DBI	3	-0.18	0.18	3.18E-01
DBNL	4	-0.47	0.44	2.91E-01
DCBLD2	15	-0.15	0.14	2.75E-01
DCN	1	-1.40	0.81	8.33E-02
DCTN1	1	-1.60	0.73	2.92E-02
DCTN2	2	0.22	0.69	7.54E-01
DCTPP1	6	-0.35	0.27	2.03E-01
DCXR	6	-0.43	0.30	1.53E-01
DDAH1	5	-0.38	0.24	1.14E-01
DDC	6	0.08	0.13	5.02E-01
DDR1	11	0.12	0.11	3.02E-01
DDX58	1	0.00	0.19	9.94E-01
DECR1	3	0.34	0.33	2.96E-01
DEFA1_DEFA1B	13	-0.24	0.19	2.05E-01
DEFB4A_DEFB4B	3	0.25	0.35	4.70E-01
DFFA	2	0.54	0.57	3.39E-01
DIABLO	3	-0.43	0.54	4.28E-01
DKK1	9	0.10	0.17	5.59E-01
DKK3	7	0.09	0.12	4.69E-01
DKK4	6	0.25	0.16	1.14E-01
DKKL1	4	-0.05	0.04	2.20E-01
DLK1	5	0.01	0.10	9.31E-01
DLL1	6	-0.12	0.22	5.94E-01
DNAJA2	1	0.04	0.81	9.56E-01
DNAJB1	5	-0.37	0.34	2.76E-01
DNAJB8	1	-1.45	0.61	1.79E-02
DNER	7	0.20	0.16	2.26E-01
DNMBP	3	-0.45	0.37	2.20E-01
DNPH1	1	0.04	0.17	8.08E-01
DOK2	4	0.45	0.39	2.48E-01
DPEP1	5	-0.22	0.28	4.25E-01
DPEP2	3	-0.04	0.17	7.96E-01
DPP10	5	0.04	0.16	7.78E-01
DPP4	5	-0.40	0.19	4.09E-02
DPP6	7	-0.15	0.15	3.18E-01
DPP7	6	0.02	0.19	9.26E-01
DPT	2	0.07	0.15	6.32E-01
DPY30	2	0.30	0.64	6.42E-01
DRAXIN	15	-0.27	0.13	4.33E-02
DRG2	3	0.62	0.35	7.86E-02
DSC2	9	-0.31	0.16	5.04E-02
DSG2	10	-0.01	0.13	9.68E-01
DSG3	17	-0.19	0.12	1.16E-01
DSG4	22	0.27	0.14	5.65E-02
DTX3	3	0.42	0.26	1.01E-01
DUSP3	2	-0.65	0.53	2.23E-01
EBAG9	5	0.13	0.34	7.02E-01
EBI3_IL27	17	-0.29	0.14	3.43E-02
ECE1	5	-0.61	0.23	7.32E-03

EDA2R	3	1.36	0.56	1.56E-02
EDAR	21	-0.02	0.09	8.61E-01
EDIL3	3	0.01	0.28	9.62E-01
EFEMP1	4	-0.23	0.24	3.32E-01
EFNA1	4	0.25	0.15	9.35E-02
EFNA4	5	-1.11	0.28	7.09E-05
EGF	8	0.30	0.23	1.86E-01
EGFL7	18	-0.13	0.14	3.68E-01
EGFR	5	-0.11	0.35	7.54E-01
EGLN1	4	0.00	0.28	9.87E-01
EIF4B	3	-0.21	0.42	6.21E-01
EIF4G1	3	0.00	0.42	9.92E-01
ELOA	2	-0.14	0.34	6.82E-01
ENAH	1	-0.28	0.47	5.49E-01
ENG	8	0.16	0.09	8.59E-02
ENO1	4	0.04	0.27	8.82E-01
ENO2	5	-0.10	0.26	7.03E-01
ENPP2	8	0.10	0.17	5.47E-01
ENPP5	6	0.01	0.07	8.24E-01
ENPP7	6	-0.04	0.05	4.90E-01
ENTPD2	2	-1.32	0.68	5.19E-02
ENTPD5	9	0.14	0.09	1.35E-01
ENTPD6	8	-0.18	0.05	1.23E-03
EPCAM	13	0.11	0.13	4.02E-01
EPHA1	7	0.07	0.19	7.12E-01
EPHA2	6	0.06	0.26	8.18E-01
EPHB4	9	0.28	0.13	3.37E-02
EPHB6	4	-0.10	0.16	5.39E-01
EPO	9	0.11	0.19	5.41E-01
EPS8L2	3	0.21	0.16	2.06E-01
ERBB2	8	-0.04	0.22	8.59E-01
ERBB3	8	-0.06	0.12	6.42E-01
ERBB4	14	-0.12	0.16	4.41E-01
ERBIN	3	0.51	0.54	3.40E-01
EREG	8	-0.12	0.24	6.16E-01
ERP44	6	0.11	0.11	3.32E-01
ESAM	7	0.26	0.16	1.11E-01
ESM1	11	0.08	0.19	6.89E-01
EZR	2	0.38	0.59	5.22E-01
F11R	11	0.66	0.25	7.93E-03
F2R	7	-0.04	0.22	8.72E-01
F3	9	0.21	0.12	7.59E-02
F7	7	0.07	0.09	4.55E-01
F9	2	0.34	0.40	4.04E-01
FABP1	2	0.46	0.22	3.67E-02
FABP2	5	0.09	0.21	6.47E-01
FABP4	1	1.66	0.72	2.10E-02
FABP5	2	-0.20	0.42	6.25E-01
FABP6	3	0.00	0.19	9.88E-01
FABP9	17	-0.09	0.10	3.45E-01
FADD	3	-0.28	0.46	5.46E-01
FAM3B	6	-0.18	0.08	2.92E-02
FAM3C	1	0.07	0.82	9.35E-01
FAP	5	0.01	0.18	9.47E-01

FAS	3	-0.17	0.19	3.53E-01
FASLG	20	-0.27	0.11	1.71E-02
FBP1	5	0.06	0.15	6.72E-01
FCAR	14	0.10	0.07	1.46E-01
FCER2	11	0.10	0.13	4.78E-01
FCGR2A	5	0.08	0.05	1.10E-01
FCGR2B	4	0.02	0.10	8.65E-01
FCGR3B	12	-0.08	0.15	5.95E-01
FCN2	10	-0.05	0.07	4.78E-01
FCRL1	24	-0.05	0.10	5.95E-01
FCRL2	13	0.07	0.08	3.36E-01
FCRL3	5	-0.02	0.05	7.45E-01
FCRL5	9	0.06	0.19	7.48E-01
FCRL6	5	0.02	0.07	7.60E-01
FCRLB	7	-0.04	0.07	5.90E-01
FEN1	1	-0.62	0.93	5.05E-01
FES	1	0.12	0.37	7.47E-01
FETUB	4	-0.11	0.12	3.61E-01
FGF19	5	0.08	0.17	6.63E-01
FGF2	6	-0.10	0.07	1.50E-01
FGF21	7	0.09	0.20	6.47E-01
FGF23	5	0.32	0.27	2.42E-01
FGF5	2	-0.16	0.08	4.05E-02
FGFBP1	11	-0.33	0.23	1.50E-01
FGFR2	7	0.03	0.27	9.22E-01
FGR	1	-0.23	0.53	6.73E-01
FHIT	2	-0.48	0.46	3.01E-01
FIS1	4	0.41	0.27	1.26E-01
FKBP1B	4	0.18	0.20	3.79E-01
FKBP4	5	-0.17	0.35	6.37E-01
FKBP5	5	-0.13	0.22	5.55E-01
FLI1	3	0.07	0.47	8.79E-01
FLRT2	11	0.09	0.08	2.63E-01
FLT1	9	0.09	0.12	4.63E-01
FLT3	5	-0.84	0.30	5.10E-03
FLT3LG	19	0.10	0.12	3.93E-01
FLT4	11	0.08	0.06	1.85E-01
FOLR1	7	-0.44	0.24	6.31E-02
FOLR2	14	-0.28	0.14	5.37E-02
FOLR3	9	-0.19	0.25	4.44E-01
FOXO1	3	-0.29	0.31	3.50E-01
FOXO3	1	3.75	0.89	2.46E-05
FRZB	2	0.04	0.11	7.35E-01
FST	3	0.59	0.27	2.68E-02
FSTL3	2	-0.21	0.47	6.61E-01
FUCA1	5	0.05	0.04	2.15E-01
FURIN	3	-0.14	0.23	5.28E-01
FUS	2	-0.29	0.49	5.49E-01
FUT3_FUT5	12	0.10	0.09	2.61E-01
FUT8	11	-0.04	0.06	4.97E-01
FXN	3	-0.28	0.51	5.93E-01
FXYD5	2	-0.13	0.52	8.00E-01
FYB1	2	0.22	0.55	6.92E-01
GAL	19	0.01	0.12	9.53E-01

GALNT10	9	-0.09	0.12	4.61E-01
GALNT2	6	0.27	0.14	5.86E-02
GALNT3	8	-0.07	0.11	5.49E-01
GALNT7	6	0.04	0.15	7.63E-01
GAS6	15	-0.05	0.11	6.59E-01
GBP2	1	-0.65	0.48	1.75E-01
GCG	5	0.11	0.39	7.85E-01
GCNT1	5	-0.03	0.11	7.81E-01
GDF15	1	-0.03	0.18	8.71E-01
GDF2	13	-0.05	0.16	7.42E-01
GDNF	1	-0.05	0.21	8.07E-01
GFAP	6	0.16	0.28	5.57E-01
GFRA1	4	0.01	0.18	9.74E-01
GFRA2	6	0.02	0.14	8.97E-01
GFRA3	20	0.24	0.14	8.26E-02
GGH	9	0.04	0.11	7.10E-01
GGT1	7	0.02	0.16	8.82E-01
GGT5	4	0.10	0.16	5.39E-01
GHRL	8	0.09	0.18	6.23E-01
GKN1	4	0.45	0.37	2.18E-01
GLB1	8	-0.29	0.13	2.46E-02
GLO1	1	-0.36	0.15	1.61E-02
GLOD4	2	0.00	0.65	9.97E-01
GLRX	2	0.00	0.10	9.66E-01
GLT8D2	1	0.12	0.27	6.63E-01
GMPR	2	0.70	0.19	3.19E-04
GNE	3	0.02	0.33	9.44E-01
GNLY	9	-0.01	0.08	9.42E-01
GOLM2	9	-0.27	0.13	3.40E-02
GOPC	2	-0.01	0.53	9.91E-01
GP1BA	15	-0.03	0.17	8.50E-01
GP2	8	-0.22	0.08	5.23E-03
GP6	6	0.10	0.15	4.98E-01
GPA33	12	0.16	0.12	1.77E-01
GPC1	15	-0.23	0.12	6.05E-02
GPC5	10	-0.01	0.06	8.83E-01
GPNMB	6	0.55	0.13	3.60E-05
GPR37	10	0.00	0.07	9.54E-01
GRAP2	3	-0.44	0.47	3.48E-01
GRK5	1	0.25	0.18	1.75E-01
GRN	10	-0.25	0.07	5.32E-04
GRPEL1	3	-0.49	0.42	2.37E-01
GSAP	1	0.48	0.80	5.53E-01
GSTA1	9	-0.19	0.14	1.62E-01
GSTA3	5	-0.21	0.15	1.62E-01
GSTP1	1	-0.01	0.24	9.55E-01
GUCA2A	7	0.21	0.19	2.56E-01
GUSB	11	-0.03	0.14	8.09E-01
GYS1	3	0.09	0.19	6.20E-01
GZMA	13	0.00	0.14	9.84E-01
GZMB	8	-0.32	0.24	1.81E-01
GZMH	7	0.01	0.19	9.74E-01
HAGH	1	-0.49	0.41	2.35E-01
HAO1	8	-0.59	0.28	3.26E-02

HARS1	2	0.07	0.55	8.99E-01
HAVCR1	7	-0.21	0.10	2.98E-02
HAVCR2	8	-0.35	0.16	3.02E-02
HBEGF	21	0.28	0.14	4.09E-02
HBQ1	2	-0.15	0.13	2.45E-01
HCLS1	4	0.04	0.35	9.17E-01
HDGF	2	0.00	0.04	9.64E-01
HEBP1	1	-0.04	0.40	9.18E-01
HEXIM1	4	0.01	0.30	9.71E-01
HGF	5	-0.04	0.25	8.88E-01
HGS	4	0.11	0.43	7.98E-01
HLA-DRA	3	-0.42	0.09	2.85E-06
HLA-E	4	-0.20	0.13	1.13E-01
HMBS	1	-0.15	0.38	6.98E-01
HMOX1	16	0.08	0.15	5.72E-01
HMOX2	4	-1.03	0.30	5.58E-04
HNMT	4	-0.08	0.07	2.40E-01
HNRNPK	1	0.07	0.83	9.34E-01
HPGDS	1	-0.74	0.76	3.32E-01
HS3ST3B1	9	0.13	0.13	3.08E-01
HS6ST1	14	-0.23	0.14	1.05E-01
HSD11B1	9	0.11	0.15	4.51E-01
HSPA1A	3	-0.14	0.37	7.01E-01
HSPB1	5	-0.58	0.31	6.33E-02
HSPB6	5	0.11	0.43	7.91E-01
HSPG2	4	-0.07	0.27	7.91E-01
HTRA2	1	0.08	0.94	9.34E-01
HYAL1	4	-0.31	0.16	5.56E-02
HYOU1	13	-0.42	0.16	8.93E-03
ICA1	2	-0.15	0.23	5.08E-01
ICAM1	6	0.14	0.11	2.25E-01
ICAM2	9	0.11	0.05	2.90E-02
ICAM3	21	0.10	0.09	2.99E-01
ICAM4	14	-0.08	0.07	2.48E-01
ICAM5	6	0.16	0.06	8.78E-03
ICOSLG	4	0.09	0.13	4.86E-01
IDI2	1	0.00	0.15	9.86E-01
IDS	3	0.07	0.22	7.70E-01
IDUA	13	0.00	0.08	9.74E-01
IFNG	2	0.16	0.64	8.01E-01
IFNGR1	10	-0.01	0.15	9.39E-01
IFNGR2	8	-0.08	0.05	1.12E-01
IFNL1	4	-0.04	0.33	9.02E-01
IFNLR1	2	-0.79	0.49	1.12E-01
IGF1R	9	0.27	0.11	1.31E-02
IGF2R	14	-0.10	0.11	3.86E-01
IGFBP1	2	-0.53	0.50	2.88E-01
IGFBP2	3	-1.28	0.47	6.82E-03
IGFBP3	5	0.04	0.10	7.15E-01
IGFBP4	3	0.27	0.53	6.08E-01
IGFBP6	3	0.13	0.51	7.91E-01
IGFBP7	3	-0.09	0.15	5.62E-01
IGFBPL1	2	0.14	0.12	2.69E-01
IGSF3	5	-0.58	0.25	1.86E-02

IGSF8	5	0.19	0.25	4.60E-01
IKBKG	1	-0.07	0.99	9.44E-01
IKZF2	1	0.14	0.48	7.64E-01
IL10	5	-0.10	0.20	6.27E-01
IL10RA	1	0.47	0.35	1.76E-01
IL10RB	6	-0.05	0.09	5.57E-01
IL11	1	-0.65	0.90	4.68E-01
IL12A_IL12B	16	-0.08	0.08	3.08E-01
IL12B	17	-0.07	0.08	3.27E-01
IL12RB1	4	0.01	0.10	9.49E-01
IL13	1	0.66	0.54	2.27E-01
IL13RA1	2	0.13	0.53	8.12E-01
IL15	22	-0.06	0.14	6.78E-01
IL15RA	1	-0.03	0.21	8.66E-01
IL16	1	-0.52	0.76	4.97E-01
IL17C	2	-0.07	0.37	8.48E-01
IL17D	2	0.06	0.14	6.51E-01
IL17F	1	-0.01	0.78	9.88E-01
IL17RA	3	-0.03	0.06	6.12E-01
IL17RB	10	-0.05	0.06	4.07E-01
IL18	8	0.18	0.12	1.54E-01
IL18BP	9	-0.40	0.20	4.87E-02
IL18R1	12	-0.02	0.06	7.20E-01
IL18RAP	3	-0.16	0.32	6.18E-01
IL19	3	0.06	0.10	5.74E-01
IL1A	1	-1.48	0.44	7.21E-04
IL1B	2	-0.10	0.19	5.94E-01
IL1R1	13	0.00	0.11	9.89E-01
IL1R2	13	-0.14	0.09	1.02E-01
IL1RAP	4	-0.08	0.05	1.08E-01
IL1RL1	12	-0.06	0.07	3.72E-01
IL1RL2	12	-0.04	0.10	6.80E-01
IL1RN	2	-0.37	0.23	9.99E-02
IL20RA	1	0.35	0.68	6.10E-01
IL22RA1	2	0.04	0.17	7.98E-01
IL2RA	8	-0.10	0.09	2.53E-01
IL32	7	0.22	0.17	1.88E-01
IL33	1	-1.75	0.90	5.14E-02
IL34	1	0.03	0.07	6.59E-01
IL3RA	4	-0.49	0.32	1.32E-01
IL4R	5	0.24	0.14	7.30E-02
IL5RA	23	0.02	0.10	8.52E-01
IL6	4	0.35	0.19	7.17E-02
IL6R	5	0.10	0.04	2.55E-02
IL6ST	11	0.24	0.13	6.13E-02
IL7	3	0.20	0.39	6.02E-01
IL7R	12	0.10	0.05	5.60E-02
ILKAP	1	0.07	0.85	9.34E-01
IMPA1	2	0.47	0.24	4.97E-02
ING1	1	0.08	0.93	9.34E-01
INHBC	6	0.11	0.08	1.93E-01
INPP1	3	-0.25	0.36	4.83E-01
IPCEF1	2	-0.79	0.47	9.54E-02
IQGAP2	1	0.52	0.34	1.25E-01

IRAG2	2	0.02	0.58	9.73E-01
IRAK1	1	-0.06	0.86	9.44E-01
IRAK4	1	-0.53	0.67	4.27E-01
ISLR2	7	0.19	0.09	2.53E-02
ISM1	2	-0.63	0.27	1.98E-02
ITGA11	5	-0.31	0.18	8.17E-02
ITGA5	10	0.00	0.18	9.92E-01
ITGA6	5	-0.05	0.17	7.61E-01
ITGAM	9	0.15	0.17	3.82E-01
ITGAV	6	-0.08	0.22	7.29E-01
ITGB1	6	0.38	0.22	8.31E-02
ITGB1BP2	4	-0.55	0.35	1.20E-01
ITGB2	9	0.01	0.16	9.41E-01
ITGB5	5	-0.10	0.31	7.49E-01
ITGB6	9	0.26	0.15	9.28E-02
ITGB7	18	0.06	0.08	4.55E-01
ITIH3	7	0.12	0.13	3.65E-01
ITM2A	1	0.12	0.23	5.97E-01
JAM2	5	0.02	0.24	9.18E-01
JCHAIN	12	-0.06	0.22	7.80E-01
KAZALD1	5	-0.02	0.07	8.16E-01
KDR	11	-0.02	0.09	7.94E-01
KEL	16	0.09	0.08	2.74E-01
KIFBP	3	0.02	0.33	9.54E-01
KIR2DL3	3	-0.25	0.14	7.11E-02
KIR3DL1	2	0.04	0.05	4.18E-01
KIRREL2	7	0.00	0.12	1.00E+00
KIT	22	0.68	0.12	4.99E-08
KITLG	15	-0.03	0.13	7.98E-01
KLB	7	0.00	0.07	9.42E-01
KLK1	13	-0.02	0.05	7.57E-01
KLK10	3	-0.09	0.08	3.07E-01
KLK11	4	0.09	0.11	4.21E-01
KLK12	5	0.03	0.04	4.40E-01
KLK13	10	-0.03	0.10	7.62E-01
KLK14	9	-0.01	0.12	9.16E-01
KLK4	3	-0.16	0.12	1.84E-01
KLK6	2	-0.23	0.26	3.69E-01
KLK8	4	-0.11	0.11	3.27E-01
KLRB1	10	-0.25	0.19	1.77E-01
KLRD1	11	0.10	0.09	2.74E-01
KRT18	8	-0.50	0.26	5.55E-02
KRT19	3	-0.01	0.30	9.63E-01
KRT5	4	0.04	0.24	8.81E-01
KYAT1	4	0.05	0.35	8.87E-01
KYNU	3	-0.19	0.19	3.07E-01
L1CAM	6	-0.26	0.20	1.82E-01
LACTB2	3	0.37	0.25	1.37E-01
LAG3	10	-0.34	0.16	2.66E-02
LAIR1	3	-0.84	0.58	1.51E-01
LAIR2	3	-0.20	0.07	3.22E-03
LAMA4	11	0.00	0.15	9.75E-01
LAMP2	2	-0.47	0.62	4.53E-01
LAMP3	9	-0.03	0.18	8.44E-01

LAT	6	0.40	0.31	1.91E-01
LAT2	1	-0.05	0.79	9.44E-01
LAYN	3	0.14	0.13	2.61E-01
LBP	2	-0.02	0.07	8.33E-01
LBR	6	0.15	0.22	4.80E-01
LCN2	16	0.00	0.17	9.78E-01
LDLR	15	-0.30	0.17	7.72E-02
LEFTY2	3	1.98	0.48	4.02E-05
LEP	1	-2.17	1.12	5.37E-02
LEPR	9	-0.09	0.16	5.87E-01
LGALS1	2	-0.17	0.18	3.48E-01
LGALS3	4	-0.71	0.35	4.37E-02
LGALS4	3	0.30	0.10	2.47E-03
GALS7_LGALS7I	7	-0.36	0.12	3.39E-03
LGALS8	6	0.18	0.10	7.55E-02
LGALS9	3	-0.82	0.36	2.11E-02
LGMN	19	-0.03	0.14	8.22E-01
LHB	1	-0.24	0.56	6.73E-01
LIFR	11	0.20	0.12	7.66E-02
LILRA2	11	-0.03	0.08	6.72E-01
LILRA5	9	0.10	0.20	6.14E-01
LILRB1	7	0.06	0.08	4.78E-01
LILRB2	10	0.03	0.05	5.07E-01
LILRB4	2	-0.42	0.57	4.65E-01
LILRB5	2	0.00	0.04	9.45E-01
LPCAT2	1	0.03	0.45	9.43E-01
LPL	7	0.28	0.12	1.98E-02
LPO	13	-0.06	0.10	5.80E-01
LRIG1	11	-0.07	0.06	2.33E-01
LRP1	11	-0.02	0.08	8.33E-01
LRP11	6	0.04	0.08	6.08E-01
LRPAP1	7	0.07	0.07	2.73E-01
LRRC25	13	-0.08	0.10	4.23E-01
LRRN1	6	-0.02	0.06	7.92E-01
LSP1	2	-0.80	0.32	1.16E-02
LTA	9	0.11	0.06	9.27E-02
LTA4H	1	-0.47	0.51	3.51E-01
LTBP2	8	0.09	0.25	7.13E-01
LTBP3	5	-0.06	0.10	5.66E-01
LTBR	13	-0.06	0.11	6.06E-01
LXN	1	0.60	0.29	4.12E-02
LY6D	5	-0.21	0.11	4.93E-02
LY75	6	0.07	0.05	1.48E-01
LY9	13	-0.14	0.09	1.08E-01
LY96	11	-0.29	0.21	1.53E-01
LYAR	1	-0.77	0.94	4.13E-01
LYN	5	0.23	0.38	5.48E-01
LYPD3	17	-0.04	0.09	6.65E-01
LYPD8	7	-0.09	0.09	3.38E-01
MAD1L1	1	0.06	0.38	8.80E-01
MAGED1	2	0.18	0.50	7.20E-01
MANF	6	0.14	0.34	6.77E-01
MANSC1	12	-0.18	0.20	3.46E-01
MAP2K6	2	0.10	0.57	8.60E-01

MAP3K5	1	-0.06	0.88	9.44E-01
MAP4K5	3	-0.27	0.26	3.04E-01
MAPK9	1	-0.39	0.43	3.63E-01
MARCO	9	-0.25	0.13	5.39E-02
MASP1	12	0.28	0.11	8.17E-03
MATN2	5	-0.13	0.11	2.28E-01
MATN3	4	-0.05	0.09	5.95E-01
MAVS	3	-0.30	0.42	4.73E-01
MAX	5	0.76	0.36	3.56E-02
MB	8	0.12	0.32	7.02E-01
MCAM	8	0.03	0.17	8.62E-01
MCFD2	4	0.33	0.26	2.06E-01
MDGA1	6	0.08	0.04	9.34E-02
MDK	13	0.15	0.19	4.38E-01
MED18	1	0.23	0.85	7.88E-01
MEGF10	11	0.35	0.16	2.38E-02
MEGF9	11	-0.13	0.12	2.98E-01
MEP1B	14	-0.01	0.05	8.49E-01
MEPE	12	-0.09	0.18	6.30E-01
MERTK	12	0.16	0.14	2.50E-01
MESD	4	0.18	0.39	6.43E-01
MET	18	0.27	0.12	2.32E-02
METAP1D	3	0.48	0.47	3.00E-01
METAP2	4	-0.24	0.32	4.43E-01
MFAP5	5	0.14	0.21	4.84E-01
MFGE8	11	0.06	0.11	5.85E-01
MGLL	3	0.31	0.43	4.76E-01
MGMT	3	-0.07	0.12	5.47E-01
MIA	6	0.05	0.07	4.99E-01
MICB_MICA	8	0.01	0.05	8.49E-01
MIF	3	0.08	0.36	8.17E-01
MILR1	9	-0.07	0.11	5.40E-01
MITD1	2	-0.16	0.54	7.70E-01
MLN	15	-0.33	0.15	3.11E-02
MME	13	-0.12	0.09	1.53E-01
MMP1	10	-0.04	0.10	6.67E-01
MMP10	2	0.11	0.15	4.74E-01
MMP12	2	-0.06	0.10	5.09E-01
MMP13	1	0.20	0.35	5.65E-01
MMP3	4	0.01	0.10	8.81E-01
MMP7	5	-0.07	0.16	6.38E-01
MMP8	2	-0.10	0.12	4.21E-01
MMP9	9	0.11	0.17	5.20E-01
MNDA	1	-1.66	0.99	9.52E-02
MOG	2	-0.09	0.24	7.04E-01
MPHOSPH8	2	-0.13	0.54	8.15E-01
MPI	4	-0.42	0.21	5.02E-02
MPIG6B	4	0.14	0.33	6.76E-01
MPO	7	-0.23	0.18	1.95E-01
MSLN	7	-0.02	0.08	7.81E-01
MSMB	5	0.16	0.05	2.36E-03
MSR1	11	-0.27	0.11	1.95E-02
MSRA	2	0.18	0.59	7.67E-01
MSTN	6	0.93	0.34	6.72E-03

MUC13	4	0.38	0.16	1.85E-02
MUC16	2	-0.06	0.24	7.92E-01
MVK	1	-0.34	0.57	5.57E-01
MYOC	10	-0.08	0.15	5.85E-01
MZB1	7	-0.34	0.24	1.59E-01
MZT1	2	-0.18	0.64	7.80E-01
NAAA	15	-0.08	0.07	2.71E-01
NADK	5	0.17	0.16	2.80E-01
NBL1	3	-0.12	0.37	7.42E-01
NBN	3	0.28	0.42	5.11E-01
NCAM1	7	0.17	0.15	2.63E-01
NCAM2	5	-0.30	0.21	1.52E-01
NCAN	11	0.09	0.20	6.39E-01
NCF2	4	-0.11	0.21	5.78E-01
NCK2	1	0.28	0.99	7.77E-01
NCR1	22	-0.26	0.11	2.60E-02
NCS1	7	-0.22	0.28	4.27E-01
NECTIN2	4	0.20	0.20	3.06E-01
NECTIN4	6	-0.25	0.15	1.01E-01
NEFL	2	-1.67	0.78	3.26E-02
NELL1	16	-0.04	0.10	7.16E-01
NELL2	13	0.26	0.20	1.79E-01
NFASC	4	-0.09	0.12	4.49E-01
NFATC1	2	0.10	0.41	7.99E-01
NFATC3	1	0.28	0.85	7.42E-01
NFKBIE	2	-0.16	0.22	4.57E-01
NID1	10	-0.12	0.17	4.71E-01
NID2	13	0.17	0.08	3.61E-02
NINJ1	1	1.29	0.71	6.97E-02
NME3	12	0.10	0.12	4.10E-01
NMNAT1	4	0.08	0.17	6.60E-01
NOMO1	3	0.72	0.34	3.24E-02
NOS1	1	0.54	0.79	4.96E-01
NOS3	2	0.13	0.58	8.26E-01
NOTCH1	7	-0.25	0.16	1.15E-01
NOTCH3	11	-0.12	0.21	5.72E-01
NPDC1	5	-0.27	0.39	4.86E-01
NPPB	2	0.20	0.26	4.39E-01
NPPC	2	0.58	0.74	4.32E-01
NPTX1	12	0.05	0.09	5.29E-01
NPTXR	4	0.07	0.09	4.14E-01
NPY	11	-0.17	0.18	3.51E-01
NRCAM	7	-0.66	0.24	6.28E-03
NRP1	6	0.05	0.15	7.35E-01
NRP2	9	0.38	0.21	7.28E-02
NRTN	1	-0.05	0.90	9.59E-01
NSFL1C	2	0.53	0.48	2.72E-01
NT5C3A	4	0.17	0.35	6.32E-01
NT5E	8	-0.10	0.08	2.27E-01
NTF3	4	-0.26	0.23	2.59E-01
NTF4	1	-0.71	0.95	4.57E-01
NTproBNP	4	0.17	0.18	3.36E-01
NTRK2	7	-0.86	0.28	2.29E-03
NTRK3	9	0.06	0.18	7.37E-01

NUB1	4	-0.02	0.24	9.35E-01
NUCB2	5	0.12	0.12	3.22E-01
NUDC	2	-0.01	0.49	9.85E-01
NUDT2	2	-0.02	0.20	9.17E-01
NUDT5	2	-1.10	0.56	4.99E-02
OBP2B	11	-0.08	0.08	3.58E-01
ODAM	2	-0.22	0.19	2.39E-01
OGFR	2	-0.30	0.36	4.06E-01
OGN	4	0.02	0.08	8.19E-01
OLR1	6	-0.04	0.26	8.79E-01
OMD	6	0.08	0.24	7.46E-01
OMG	8	-0.06	0.23	7.90E-01
OPTC	7	-0.15	0.17	3.70E-01
OSCAR	8	-0.09	0.09	3.16E-01
OSM	8	-0.13	0.18	4.95E-01
OSMR	5	0.11	0.09	2.13E-01
OXT	9	0.04	0.09	6.57E-01
P4HB	4	0.28	0.41	4.88E-01
PADI2	1	-0.31	0.40	4.37E-01
PADI4	1	-0.11	0.19	5.76E-01
PAEP	3	0.13	0.07	6.17E-02
PAG1	6	-0.31	0.29	2.79E-01
PAK4	1	-0.07	0.95	9.44E-01
PAM	4	-0.03	0.10	7.26E-01
PAMR1	4	0.15	0.13	2.39E-01
PAPPA	11	-0.23	0.16	1.68E-01
PARK7	5	0.19	0.26	4.72E-01
PARP1	2	-0.44	0.29	1.28E-01
PCDH1	5	-0.33	0.28	2.49E-01
PCDH17	8	-0.01	0.10	8.87E-01
PCOLCE	1	-0.27	0.30	3.54E-01
PCSK9	8	-0.29	0.14	4.17E-02
PDCD1	11	0.31	0.24	1.83E-01
PDCD1LG2	8	0.05	0.06	3.95E-01
PDCD5	2	-0.20	0.14	1.43E-01
PDCD6	4	0.11	0.05	2.34E-02
PDGFA	16	0.15	0.14	2.96E-01
PDGFB	15	0.09	0.14	5.10E-01
PDGFC	4	-0.01	0.16	9.73E-01
PDGFRA	10	0.07	0.15	6.48E-01
PDGFRB	2	0.01	0.04	8.91E-01
PDLIM7	3	-0.83	0.45	6.51E-02
PEAR1	8	-0.20	0.14	1.60E-01
PEBP1	2	-0.43	0.63	4.97E-01
PECAM1	7	0.23	0.10	2.12E-02
PFKFB2	4	0.00	0.23	9.94E-01
PGF	3	0.44	0.46	3.38E-01
PGLYRP1	12	-0.04	0.14	7.77E-01
PI3	4	0.02	0.17	9.10E-01
PIGR	6	0.11	0.22	6.27E-01
PIK3AP1	6	-0.05	0.21	8.17E-01
PIK3IP1	3	0.14	0.17	4.18E-01
PILRA	6	0.01	0.05	8.50E-01
PILRB	4	0.01	0.05	7.70E-01

PKLR	4	-0.21	0.24	3.80E-01
PLA2G10	13	0.32	0.11	2.75E-03
PLA2G15	9	-0.09	0.13	4.91E-01
PLA2G1B	17	0.12	0.16	4.48E-01
PLA2G2A	6	0.04	0.08	6.50E-01
PLA2G4A	2	-0.24	0.60	6.96E-01
PLA2G7	8	-1.20	0.27	6.21E-06
PLAT	4	-0.39	0.37	2.93E-01
PLAU	9	0.11	0.14	4.29E-01
PLAUR	10	-0.42	0.18	1.69E-02
PLIN1	1	-0.51	0.60	3.93E-01
PLIN3	2	-0.48	0.56	3.93E-01
PLPBP	2	0.00	0.56	1.00E+00
PLTP	11	-0.03	0.10	7.82E-01
PLXDC1	4	0.04	0.19	8.54E-01
PLXNA4	5	0.45	0.27	9.24E-02
PLXNB2	11	0.03	0.07	6.47E-01
PLXNB3	5	0.26	0.39	5.09E-01
PM20D1	7	0.17	0.29	5.70E-01
PMVK	6	0.05	0.29	8.57E-01
PNLIPRP2	7	-0.09	0.04	3.14E-02
PNPT1	2	-0.04	0.54	9.34E-01
PODXL	6	0.18	0.09	4.39E-02
PODXL2	11	-0.40	0.20	4.42E-02
POLR2F	1	-1.36	0.95	1.52E-01
PON2	3	0.02	0.06	7.58E-01
PON3	9	0.12	0.13	3.67E-01
PPCDC	2	0.01	0.15	9.19E-01
PPIB	3	0.39	0.48	4.15E-01
PPME1	1	-0.21	0.94	8.28E-01
PPP1R12A	4	-0.23	0.37	5.38E-01
PPP1R2	3	0.20	0.46	6.58E-01
PPP1R9B	2	-0.01	0.59	9.82E-01
PPP3R1	4	0.40	0.34	2.38E-01
PPY	7	0.47	0.17	5.60E-03
PRCP	12	-0.28	0.14	4.12E-02
PRDX1	2	-0.22	0.54	6.80E-01
PRDX3	1	-0.83	0.78	2.88E-01
PRDX5	2	-0.32	0.33	3.31E-01
PRDX6	1	0.23	0.36	5.31E-01
PRELP	1	0.06	0.11	5.91E-01
PRKAB1	1	-0.07	0.93	9.44E-01
PRKAR1A	5	-0.20	0.22	3.67E-01
PRKRA	1	-0.03	0.49	9.44E-01
PRL	3	-0.15	0.46	7.46E-01
PROC	7	-0.28	0.18	1.22E-01
PROK1	2	-0.25	0.14	7.83E-02
PRSS2	11	0.10	0.18	5.61E-01
PRSS27	17	-0.07	0.07	3.07E-01
PRSS8	8	-0.23	0.24	3.26E-01
PRTFDC1	3	0.01	0.23	9.79E-01
PRTG	8	0.02	0.10	8.75E-01
PRTN3	12	-0.07	0.21	7.37E-01
PSG1	6	0.00	0.31	9.93E-01

PSIP1	3	0.18	0.33	5.88E-01
PSME1	2	0.85	0.50	8.93E-02
PSME2	1	1.14	0.55	3.76E-02
PSMG3	1	-0.84	1.00	4.00E-01
PSPN	8	-0.18	0.35	6.15E-01
PSRC1	2	0.08	0.28	7.70E-01
PTGDS	2	0.13	0.68	8.44E-01
PTH1R	1	-0.27	0.21	1.91E-01
PTN	3	0.35	0.29	2.26E-01
PTPN1	3	0.39	0.39	3.18E-01
PTPN6	2	0.00	0.53	1.00E+00
PTPRF	13	0.53	0.17	1.31E-03
PTPRM	8	0.19	0.09	2.70E-02
PTPRN2	1	0.01	0.33	9.85E-01
PTPRS	5	0.03	0.17	8.46E-01
PTS	4	-0.13	0.21	5.37E-01
PTX3	5	-0.32	0.13	1.61E-02
PVALB	6	-0.10	0.06	1.39E-01
PVR	2	0.04	0.07	5.46E-01
PXN	3	-0.07	0.08	3.49E-01
QDPR	1	-0.06	0.81	9.44E-01
QPCT	12	-0.02	0.13	9.07E-01
RAB6A	2	-0.27	0.19	1.47E-01
RABEPK	1	0.17	0.33	6.16E-01
RABGAP1L	2	0.45	0.52	3.91E-01
RAD23B	3	0.08	0.50	8.72E-01
RARRES1	1	-0.10	0.11	3.62E-01
RARRES2	3	0.15	0.24	5.15E-01
RASSF2	1	0.07	0.53	9.00E-01
RBKS	1	0.19	0.14	1.60E-01
RBP2	4	-0.30	0.35	3.83E-01
RBP5	3	0.10	0.16	5.44E-01
RCOR1	1	0.05	0.84	9.54E-01
REG1A	6	-0.30	0.12	1.62E-02
REG1B	7	-0.29	0.11	8.04E-03
REG3A	8	-0.01	0.16	9.26E-01
REG4	2	0.26	0.28	3.55E-01
RELT	4	-0.05	0.15	7.21E-01
REN	2	0.37	0.32	2.46E-01
RET	8	-0.11	0.12	3.51E-01
RETN	15	-0.10	0.13	4.41E-01
RGMA	17	-0.05	0.12	6.53E-01
RGMB	4	-0.11	0.25	6.68E-01
RGS8	1	0.22	0.29	4.48E-01
RHOC	3	0.21	0.58	7.23E-01
RILP	4	-0.09	0.37	8.13E-01
RNASE3	4	-0.05	0.18	7.68E-01
RNASET2	7	-0.30	0.11	8.35E-03
ROBO1	8	-0.25	0.18	1.62E-01
ROBO2	5	-0.36	0.30	2.28E-01
ROR1	12	-0.22	0.14	1.10E-01
RP2	2	-1.04	0.43	1.56E-02
RRM2	1	0.30	0.83	7.20E-01
RRM2B	1	-0.04	0.55	9.44E-01

RSPO1	10	0.02	0.17	8.99E-01
RSPO3	8	-0.09	0.22	7.02E-01
RTBDN	2	-0.10	0.30	7.29E-01
RTN4R	9	-0.21	0.16	1.82E-01
RUVBL1	1	-0.18	0.22	4.06E-01
RWDD1	2	0.50	0.34	1.40E-01
S100A11	2	-0.13	0.22	5.38E-01
S100A12	4	0.05	0.19	7.96E-01
S100A16	3	0.14	0.34	6.76E-01
S100A4	4	0.02	0.46	9.61E-01
S100P	5	0.45	0.31	1.48E-01
SAMD9L	2	-0.04	0.17	8.24E-01
SCAMP3	4	0.25	0.36	4.82E-01
SCARA5	6	-0.08	0.15	5.98E-01
SCARB1	1	-0.07	0.93	9.40E-01
SCARB2	4	-0.33	0.20	1.05E-01
SCARF1	7	0.07	0.12	5.77E-01
SCARF2	8	-0.09	0.14	5.42E-01
SCG2	4	0.58	0.40	1.47E-01
SCG3	5	0.00	0.08	9.54E-01
SCGB1A1	16	0.17	0.12	1.42E-01
SCGB3A2	16	0.13	0.14	3.37E-01
SCGN	3	0.11	0.26	6.60E-01
SCLY	7	-0.31	0.23	1.68E-01
SCP2	1	0.70	0.93	4.50E-01
SCRN1	2	0.22	0.36	5.38E-01
SDC1	13	-0.08	0.16	6.10E-01
SDC4	20	0.17	0.13	1.66E-01
SELE	9	0.13	0.06	3.33E-02
SELP	10	0.29	0.13	2.52E-02
SELPLG	10	0.07	0.09	4.59E-01
SEMA3F	5	0.66	0.35	5.61E-02
SEMA4C	4	0.78	0.21	2.36E-04
SEMA4D	6	0.06	0.29	8.44E-01
SEMA7A	18	-0.18	0.12	1.47E-01
SEPTIN9	2	0.07	0.46	8.87E-01
SERPINA11	8	0.01	0.08	9.45E-01
SERPINA12	5	-0.01	0.08	8.99E-01
SERPINA9	3	-0.06	0.11	5.81E-01
SERPINB1	3	0.22	0.41	5.85E-01
SERPINB6	1	0.07	0.87	9.34E-01
SERPINB8	2	0.08	0.07	2.24E-01
SERPINB9	2	0.04	0.39	9.13E-01
SERPINE1	17	0.29	0.15	5.00E-02
SESTD1	3	-0.29	0.38	4.50E-01
SETMAR	2	-0.18	0.22	4.18E-01
SEZ6L	8	-0.14	0.22	5.26E-01
SEZ6L2	8	-0.16	0.20	4.18E-01
SF3B4	4	0.14	0.41	7.22E-01
SFRP1	5	0.26	0.22	2.46E-01
SFTPA1	1	-0.03	0.22	8.99E-01
SFTPA2	6	-0.14	0.08	6.24E-02
SFTPD	10	0.08	0.06	1.56E-01
SH2B3	4	0.35	0.39	3.67E-01

SH2D1A	2	-0.15	0.42	7.22E-01
SHMT1	4	0.01	0.07	9.03E-01
SIAE	8	-0.30	0.18	9.88E-02
SIGLEC1	9	0.00	0.16	9.83E-01
SIGLEC10	8	-0.04	0.11	7.02E-01
SIGLEC5	3	0.02	0.06	7.70E-01
SIGLEC6	21	-0.10	0.10	3.25E-01
SIGLEC7	8	-0.20	0.13	1.22E-01
SIGLEC9	11	0.09	0.05	1.08E-01
SIRPA	8	0.02	0.04	6.39E-01
SIRPB1	11	-0.09	0.07	2.23E-01
SIRT2	3	-0.06	0.30	8.46E-01
SIT1	6	-0.32	0.27	2.42E-01
SKAP1	7	0.01	0.29	9.84E-01
SKAP2	3	0.23	0.47	6.28E-01
SLAMF1	4	-0.28	0.28	3.27E-01
SLAMF6	3	-0.23	0.25	3.51E-01
SLAMF7	9	-0.01	0.07	9.26E-01
SLAMF8	5	-0.06	0.05	2.54E-01
SLC16A1	4	0.06	0.30	8.46E-01
SLC27A4	3	0.20	0.37	5.96E-01
SLC39A14	2	0.38	0.37	3.07E-01
SLC39A5	8	0.27	0.22	2.21E-01
SLIT2	1	-0.63	0.82	4.40E-01
SLITRK2	11	-0.44	0.20	3.00E-02
SLITRK6	7	-0.25	0.13	4.47E-02
SMAD1	5	0.20	0.38	5.95E-01
SMAD5	1	0.26	0.29	3.58E-01
SMOC1	9	0.26	0.14	7.23E-02
SMOC2	5	0.05	0.13	6.78E-01
SMPD1	15	-0.06	0.09	5.35E-01
SMPDL3A	19	-0.10	0.06	8.35E-02
SNAP23	2	-0.01	0.40	9.78E-01
SNAP29	5	0.39	0.33	2.35E-01
SNCG	1	-0.01	0.05	8.13E-01
SNX9	2	0.06	0.45	9.01E-01
SOD1	3	0.19	0.40	6.31E-01
SOD2	9	0.04	0.19	8.40E-01
SORCS2	4	-0.02	0.11	8.67E-01
SORD	3	0.24	0.31	4.45E-01
SORT1	12	0.00	0.15	9.95E-01
SOST	14	-0.07	0.16	6.55E-01
SPARC	13	0.41	0.18	1.94E-02
SPARCL1	4	-0.24	0.07	6.21E-04
SPINK1	2	-0.10	0.12	4.34E-01
SPINK4	5	-0.04	0.06	4.80E-01
SPINK5	6	-0.20	0.19	2.90E-01
SPINK6	1	0.02	0.14	8.81E-01
SPINT1	8	-0.15	0.13	2.55E-01
SPINT2	15	0.22	0.14	1.21E-01
SPOCK1	11	-0.09	0.21	6.50E-01
SPON1	6	0.25	0.15	9.08E-02
SPON2	1	0.08	0.24	7.37E-01
SPP1	7	-0.29	0.30	3.36E-01

SPRY2	6	0.18	0.26	5.00E-01
SRC	1	0.99	0.91	2.79E-01
SRP14	2	-0.28	0.41	4.93E-01
SRPK2	3	-0.07	0.37	8.38E-01
SSC4D	11	0.03	0.12	7.80E-01
SSC5D	10	-0.18	0.18	3.12E-01
ST3GAL1	7	-0.06	0.15	6.99E-01
ST6GAL1	6	-0.03	0.24	9.10E-01
STAMBP	3	-0.34	0.50	4.98E-01
STAT5B	3	0.16	0.48	7.33E-01
STC1	5	0.90	0.28	1.36E-03
STC2	5	-0.80	0.40	4.60E-02
STIP1	2	-0.05	0.50	9.25E-01
STK11	4	0.16	0.40	6.94E-01
STK24	2	0.01	0.66	9.93E-01
STK4	4	0.37	0.40	3.46E-01
STX16	1	-0.46	0.86	5.95E-01
STX4	2	-0.45	0.60	4.50E-01
STX6	1	0.49	0.95	6.08E-01
STX8	3	-0.05	0.42	9.11E-01
SUGT1	1	-0.05	0.77	9.44E-01
SULT1A1	1	0.34	0.50	5.02E-01
SULT2A1	3	0.11	0.17	5.11E-01
SUMF2	14	0.04	0.10	6.93E-01
SUSD1	4	0.47	0.24	4.78E-02
SUSD2	4	0.10	0.11	3.33E-01
TACC3	4	0.30	0.36	3.97E-01
TACSTD2	13	-0.08	0.06	2.04E-01
TAFA5	2	0.08	0.29	7.80E-01
TARBP2	3	0.02	0.47	9.65E-01
TBC1D17	1	-0.16	0.12	1.88E-01
TBC1D23	4	0.43	0.28	1.17E-01
TBC1D5	2	-0.53	0.60	3.72E-01
TBCB	4	-0.06	0.42	8.85E-01
TBCC	4	-0.15	0.29	6.03E-01
TBL1X	5	0.51	0.28	6.77E-02
TCL1A	18	-0.15	0.12	2.25E-01
TCN2	1	0.13	0.10	1.71E-01
TDGF1	2	0.03	0.04	4.53E-01
TDRKH	4	0.32	0.15	3.10E-02
TEK	9	-0.12	0.08	1.58E-01
TFF1	5	0.02	0.14	8.78E-01
TFF2	8	0.56	0.20	4.91E-03
TFF3	3	0.42	0.14	2.16E-03
TFPI	8	-0.18	0.12	1.51E-01
TFPI2	8	-0.25	0.21	2.23E-01
TFRC	10	0.12	0.08	1.48E-01
TGFA	4	-0.62	0.30	3.48E-02
TGFB1	7	-0.05	0.17	7.85E-01
TGFBI	8	-0.09	0.08	2.17E-01
TGFBR2	7	0.09	0.25	7.30E-01
TGFBR3	6	0.01	0.22	9.70E-01
TGM2	2	0.07	0.28	8.10E-01
THBD	14	-0.17	0.16	2.95E-01

THBS2	7	0.08	0.11	4.25E-01
THBS4	5	-0.21	0.14	1.46E-01
THOP1	1	-0.02	0.20	9.10E-01
THPO	15	0.20	0.16	2.25E-01
THY1	3	0.14	0.15	3.44E-01
TIA1	2	0.24	0.49	6.23E-01
TIE1	11	-0.01	0.09	8.88E-01
TIGAR	2	0.53	0.59	3.67E-01
TIMD4	23	-0.02	0.10	8.48E-01
TIMP1	5	-0.16	0.27	5.55E-01
TIMP3	15	0.02	0.08	8.42E-01
TIMP4	4	0.21	0.14	1.35E-01
TINAGL1	9	-0.32	0.22	1.32E-01
TJAP1	1	-0.05	0.78	9.44E-01
TLR3	6	0.01	0.05	7.66E-01
TMPRSS15	7	0.25	0.16	1.11E-01
TMPRSS5	17	-0.07	0.06	2.85E-01
TMSB10	6	-0.24	0.30	4.32E-01
TNC	15	-0.02	0.10	8.24E-01
TNF	3	0.22	0.26	4.04E-01
TNFAIP8	1	-1.09	0.62	7.96E-02
TNFRSF10A	3	0.01	0.10	9.03E-01
TNFRSF10B	3	-0.04	0.15	7.72E-01
TNFRSF10C	17	0.05	0.06	3.62E-01
TNFRSF11A	5	0.00	0.10	9.81E-01
TNFRSF11B	9	0.27	0.19	1.59E-01
TNFRSF12A	2	0.09	0.57	8.71E-01
TNFRSF13B	10	0.07	0.23	7.45E-01
TNFRSF13C	9	-0.08	0.25	7.32E-01
TNFRSF14	5	0.01	0.31	9.80E-01
TNFRSF19	5	-0.05	0.17	7.91E-01
TNFRSF1A	7	-0.08	0.27	7.70E-01
TNFRSF1B	6	-0.41	0.22	6.57E-02
TNFRSF21	12	-0.34	0.14	1.26E-02
TNFRSF4	6	-0.66	0.25	6.75E-03
TNFRSF6B	3	0.55	0.16	4.72E-04
TNFRSF8	7	-0.30	0.18	9.64E-02
TNFRSF9	5	-0.56	0.23	1.62E-02
TNFSF10	11	0.13	0.09	1.50E-01
TNFSF11	8	0.15	0.13	2.57E-01
TNFSF12	16	0.00	0.12	9.85E-01
TNFSF13	7	-0.31	0.14	2.73E-02
TNFSF13B	4	0.60	0.30	4.81E-02
TNFSF14	5	0.15	0.17	3.81E-01
TNR	16	0.10	0.10	2.75E-01
TNXB	6	-0.26	0.11	2.09E-02
TP53	1	-0.38	0.83	6.45E-01
TP53INP1	2	-0.01	0.43	9.78E-01
TPMT	2	-0.05	0.10	6.01E-01
TPP1	11	-0.30	0.13	2.38E-02
TPPP3	1	-0.26	0.79	7.42E-01
TPSAB1	13	0.03	0.06	5.97E-01
TRAF2	3	0.43	0.49	3.86E-01
TREM2	7	0.08	0.10	3.96E-01

TREML2	7	-0.03	0.16	8.54E-01
TRIAP1	6	0.25	0.23	2.79E-01
TRIM21	3	0.36	0.35	3.12E-01
TRIM5	1	0.05	0.17	7.46E-01
TSHB	15	0.25	0.16	1.30E-01
TSPAN1	3	-1.32	0.53	1.26E-02
TST	1	-0.65	0.50	1.92E-01
TXLNA	3	0.08	0.38	8.33E-01
TXNDC15	10	-0.09	0.06	1.25E-01
TXNDC5	4	0.04	0.33	8.93E-01
TXNRD1	4	0.06	0.42	8.93E-01
TYMP	2	-0.09	0.25	7.29E-01
TYRO3	8	-0.17	0.07	2.01E-02
UBAC1	1	0.33	0.68	6.31E-01
ULBP2	6	-0.71	0.23	1.94E-03
UMOD	15	0.00	0.06	9.67E-01
USO1	3	0.02	0.47	9.72E-01
USP8	4	0.07	0.29	8.14E-01
UXS1	6	0.12	0.18	4.92E-01
VAMP5	1	-2.64	0.83	1.38E-03
VASH1	4	-0.18	0.30	5.45E-01
VASN	5	0.11	0.11	3.29E-01
VAT1	9	0.05	0.20	8.01E-01
VCAM1	10	-0.24	0.18	1.83E-01
VCAN	14	0.03	0.07	6.81E-01
VEGFA	13	0.00	0.06	9.67E-01
VEGFC	15	0.22	0.17	2.00E-01
VEGFD	5	-0.60	0.25	1.84E-02
VMO1	6	-0.05	0.06	4.37E-01
VNN2	10	-0.06	0.07	3.70E-01
VPS37A	2	-0.53	0.66	4.26E-01
VPS53	2	0.19	0.63	7.64E-01
VSIG4	8	-0.13	0.29	6.57E-01
VSIR	8	-0.10	0.14	4.59E-01
VSTM1	2	-0.03	0.06	6.16E-01
VSTM2L	2	-0.69	0.24	3.62E-03
VTA1	3	-0.02	0.45	9.72E-01
VTCN1	2	-0.27	0.44	5.39E-01
VWA1	6	0.19	0.27	4.82E-01
VWC2	3	-0.09	0.10	3.76E-01
VWF	6	-0.45	0.15	2.65E-03
WARS	3	-0.28	0.20	1.54E-01
WAS	2	-0.77	0.53	1.43E-01
WASF1	3	0.06	0.17	7.20E-01
WASF3	1	0.88	0.36	1.60E-02
WFDC12	11	-0.43	0.16	8.36E-03
WFDC2	3	-0.49	0.44	2.60E-01
WFIKKN1	13	-0.03	0.15	8.46E-01
WFIKKN2	4	0.14	0.08	5.81E-02
WIF1	13	0.29	0.14	3.73E-02
WWP2	3	0.06	0.25	8.25E-01
XCL1	9	0.00	0.07	9.97E-01
XG	1	0.90	0.93	3.32E-01
XPNPEP2	12	-0.07	0.18	7.16E-01

XRCC4	1	-0.32	0.77	6.73E-01
YES1	2	-0.88	0.52	8.75E-02
YTHDF3	1	-0.04	0.58	9.44E-01
ZBTB16	7	0.10	0.14	4.45E-01
ZBTB17	2	0.84	0.52	1.03E-01

Supplementary Table 16. Associations of genetically predicted proteins with father's attained age using IVW method and cis+trans SNPs.

Protein	No. of SNPs	Beta	SE	Pval
AARSD1	3	0.04	0.30	9.03E-01
ABHD14B	3	-0.38	0.45	3.92E-01
ABL1	1	-0.10	0.53	8.52E-01
ACAA1	3	0.31	0.35	3.73E-01
ACAN	13	-0.29	0.18	1.06E-01
ACE2	7	0.28	0.23	2.22E-01
ACOX1	1	0.04	0.62	9.44E-01
ACP5	10	-0.12	0.12	2.99E-01
ACP6	2	0.06	0.05	2.05E-01
ACVRL1	3	0.00	0.14	9.87E-01
ACY1	3	0.16	0.19	3.96E-01
ADA	4	0.00	0.07	9.74E-01
ADA2	13	-0.15	0.13	2.53E-01
ADAM15	8	0.06	0.05	2.69E-01
ADAM22	6	0.11	0.08	1.58E-01
ADAM23	4	0.01	0.08	9.39E-01
ADAM8	7	-0.27	0.09	3.66E-03
ADAMTS13	10	0.11	0.11	2.92E-01
ADAMTS15	2	1.12	0.38	3.25E-03
ADAMTS16	1	0.58	0.38	1.29E-01
ADAMTS8	3	-0.02	0.10	8.24E-01
ADGRB3	4	0.10	0.05	2.99E-02
ADGRE2	8	-0.08	0.13	5.26E-01
ADGRE5	9	-0.24	0.16	1.29E-01
ADGRG1	9	-0.11	0.19	5.72E-01
ADGRG2	5	-0.23	0.30	4.46E-01
ADH4	6	0.47	0.23	4.37E-02
ADM	1	-0.90	0.92	3.30E-01
AFP	19	0.11	0.10	2.43E-01
AGER	12	0.35	0.16	2.49E-02
AGR2	4	0.27	0.30	3.78E-01
AGR3	2	-1.02	0.47	3.12E-02
AGRN	6	0.14	0.11	2.01E-01
AGRP	9	-1.08	0.22	1.41E-06
AGXT	10	-0.28	0.22	2.00E-01
AHCY	1	0.08	0.13	5.18E-01
AHSP	7	-0.62	0.23	7.11E-03
AIF1	1	0.71	0.40	7.49E-02
AIFM1	2	-0.48	0.59	4.12E-01
AKR1B1	3	-0.23	0.28	3.99E-01
AKR1C4	1	1.49	0.84	7.71E-02
AKT1S1	3	-0.41	0.41	3.18E-01
AKT3	2	-0.19	0.60	7.49E-01
ALCAM	11	0.01	0.17	9.60E-01
ALDH1A1	3	0.19	0.34	5.76E-01
ALDH3A1	1	0.03	0.10	7.39E-01
ALPP	9	0.69	0.22	1.65E-03
AMBP	9	-0.04	0.16	8.08E-01
AMIGO2	11	-0.03	0.12	7.98E-01
AMN	5	0.03	0.06	6.34E-01
AMY2A	7	0.01	0.11	9.62E-01
AMY2B	8	0.08	0.10	4.49E-01
ANG	3	-0.06	0.10	5.35E-01
ANGPT1	16	-0.17	0.13	1.88E-01
ANGPT2	6	-0.02	0.18	9.04E-01
ANGPTL1	6	0.12	0.14	3.96E-01
ANGPTL2	4	-0.50	0.28	7.63E-02
ANGPTL3	5	0.79	0.30	7.98E-03

ANGPTL4	1	0.55	0.16	5.52E-04
ANGPTL7	6	-0.01	0.10	9.46E-01
ANKRD54	1	-0.14	0.66	8.35E-01
ANPEP	7	-0.05	0.11	6.74E-01
ANXA11	2	0.19	0.54	7.29E-01
ANXA3	5	-0.20	0.29	4.98E-01
ANXA4	2	-0.36	0.51	4.78E-01
ANXA5	1	0.26	0.40	5.13E-01
AOC1	1	0.00	0.16	9.97E-01
AOC3	10	0.11	0.08	1.73E-01
APBB1IP	5	0.03	0.15	8.64E-01
APEX1	3	0.15	0.11	1.79E-01
APLP1	3	-0.06	0.34	8.50E-01
APOH	1	0.25	0.09	4.21E-03
APOM	8	0.05	0.13	7.02E-01
APP	14	-0.09	0.15	5.50E-01
APRT	5	0.39	0.27	1.56E-01
AREG	3	0.44	0.24	6.33E-02
ARG1	5	0.40	0.19	3.28E-02
ARHGAP1	2	-0.07	0.42	8.62E-01
ARHGAP25	2	0.20	0.40	6.22E-01
ARSA	7	0.08	0.06	2.10E-01
ARSB	10	-0.29	0.12	1.78E-02
ART3	4	-0.32	0.37	3.86E-01
ARTN	1	-0.56	0.68	4.13E-01
ASAH2	15	-0.05	0.07	4.57E-01
ASGR1	6	0.15	0.17	4.00E-01
ATF2	1	0.05	0.37	8.86E-01
ATG4A	3	-0.41	0.44	3.53E-01
ATOX1	3	-0.87	0.44	4.90E-02
ATP5IF1	5	-0.45	0.29	1.19E-01
ATP6V1F	1	-0.13	0.69	8.52E-01
ATXN10	5	0.02	0.28	9.43E-01
AXIN1	3	-0.46	0.46	3.15E-01
AXL	6	0.16	0.16	3.00E-01
AZU1	2	-0.30	0.55	5.77E-01
B4GALT1	7	-0.09	0.12	4.15E-01
B4GAT1	8	0.31	0.12	1.18E-02
BACH1	3	0.14	0.48	7.73E-01
BAG3	1	-0.76	0.71	2.90E-01
BAIAP2	5	0.22	0.32	4.87E-01
BAMBI	1	0.13	0.67	8.52E-01
BANK1	3	-0.28	0.30	3.44E-01
BAX	2	0.06	0.55	9.09E-01
BCAM	13	-0.26	0.09	5.02E-03
BCAN	7	0.45	0.15	3.62E-03
BCL2L11	6	-0.10	0.18	5.80E-01
BCR	1	-1.15	0.70	1.00E-01
BGN	9	-0.17	0.19	3.88E-01
BID	2	0.64	0.43	1.36E-01
BIN2	2	-0.17	0.52	7.45E-01
BIRC2	4	-0.20	0.40	6.16E-01
BLMH	2	0.04	0.08	6.05E-01
BLVRB	2	-0.01	0.21	9.48E-01
BMP4	5	0.48	0.28	8.55E-02
BMP6	11	0.25	0.14	7.12E-02
BOC	8	0.00	0.19	9.98E-01
BPIFB1	13	-0.03	0.09	7.53E-01
BRK1	6	0.02	0.19	9.01E-01
BSG	9	-0.16	0.19	3.79E-01

BST1	4	0.01	0.04	7.93E-01
BST2	11	-0.05	0.18	8.02E-01
BTC	3	0.03	0.11	7.69E-01
BTN2A1	6	-0.33	0.13	1.27E-02
BTN3A2	3	0.01	0.07	8.44E-01
C19orf12	4	0.89	0.31	3.81E-03
C1QA	12	-0.09	0.10	3.98E-01
C1QTNF1	4	0.35	0.16	2.95E-02
C2	2	0.30	0.37	4.18E-01
C2CD2L	1	-0.60	0.81	4.58E-01
C4BPB	9	-0.39	0.13	2.48E-03
CA1	4	-0.81	0.26	2.01E-03
CA11	2	-1.55	0.26	3.52E-09
CA12	6	-0.02	0.14	8.85E-01
CA13	3	0.15	0.16	3.47E-01
CA14	4	0.05	0.22	8.28E-01
CA2	2	0.40	0.39	3.00E-01
CA3	6	-0.37	0.18	4.19E-02
CA4	8	0.15	0.11	1.88E-01
CA5A	5	-0.06	0.07	3.43E-01
CA6	11	-0.09	0.06	1.70E-01
CA9	5	0.43	0.20	3.48E-02
CALB1	3	0.02	0.29	9.55E-01
CALCA	4	0.16	0.32	6.23E-01
CALCOCO1	4	-0.36	0.30	2.32E-01
CAMKK1	3	-0.20	0.15	1.94E-01
CANT1	5	-0.10	0.23	6.58E-01
CAPG	2	0.02	0.05	7.66E-01
CARHSP1	1	-0.29	0.56	6.07E-01
CASP1	1	0.18	0.17	2.71E-01
CASP10	3	-0.37	0.21	7.53E-02
CASP2	2	0.04	0.40	9.14E-01
CASP3	1	-0.18	0.63	7.80E-01
CASP8	3	0.26	0.19	1.72E-01
CBLIF	9	0.29	0.09	2.08E-03
CBLN4	5	-0.07	0.19	7.07E-01
CC2D1A	2	-0.77	0.49	1.17E-01
CCDC80	5	0.02	0.20	9.05E-01
CCL11	8	0.00	0.15	9.85E-01
CCL13	12	0.02	0.12	8.32E-01
CCL14	5	0.01	0.09	8.85E-01
CCL15	4	0.69	0.21	1.05E-03
CCL16	7	-0.05	0.06	3.63E-01
CCL17	12	0.03	0.09	7.50E-01
CCL18	3	0.11	0.09	2.06E-01
CCL19	4	-0.79	0.30	8.54E-03
CCL2	8	0.15	0.14	2.70E-01
CCL20	4	-0.64	0.29	2.90E-02
CCL21	10	-0.64	0.19	7.47E-04
CCL22	6	-0.23	0.17	1.88E-01
CCL23	7	0.15	0.11	1.73E-01
CCL24	10	-0.09	0.05	6.66E-02
CCL25	6	0.10	0.06	7.56E-02
CCL26	5	-0.06	0.15	6.97E-01
CCL27	7	0.01	0.17	9.50E-01
CCL28	16	-0.01	0.14	9.60E-01
CCL3	6	-1.13	0.31	2.29E-04
CCL4	5	-0.14	0.11	2.08E-01
CCL5	9	-0.06	0.16	7.12E-01
CCL7	4	0.05	0.13	6.89E-01

CCL8	8	0.04	0.05	4.71E-01
CCN1	11	-0.77	0.18	3.10E-05
CCN2	16	-0.13	0.11	2.18E-01
CCN3	3	-0.12	0.18	4.94E-01
CCN4	11	0.13	0.10	1.68E-01
CCN5	1	3.08	0.92	8.12E-04
CCS	1	-0.04	0.09	6.37E-01
CD109	6	0.65	0.19	7.64E-04
CD14	5	0.26	0.14	6.69E-02
CD160	14	0.00	0.09	9.99E-01
CD163	17	-0.23	0.09	1.40E-02
CD164	9	-0.17	0.18	3.53E-01
CD177	3	-0.08	0.09	3.62E-01
CD1C	17	-0.45	0.12	1.73E-04
CD200	7	-0.28	0.17	9.61E-02
CD200R1	9	-0.12	0.05	3.44E-02
CD207	14	0.08	0.08	3.29E-01
CD209	8	-0.15	0.05	3.91E-03
CD22	18	0.11	0.12	3.41E-01
CD244	13	-0.16	0.09	8.28E-02
CD27	7	-0.74	0.15	1.21E-06
CD274	6	0.04	0.13	7.86E-01
CD276	7	0.04	0.05	4.31E-01
CD28	8	-0.02	0.14	8.72E-01
CD2AP	1	-0.13	0.70	8.52E-01
CD300C	12	0.14	0.09	1.31E-01
CD300E	10	-0.31	0.11	5.37E-03
CD300LF	9	0.03	0.04	5.56E-01
CD300LG	10	0.17	0.10	1.13E-01
CD302	5	-0.30	0.13	2.49E-02
CD33	7	-0.03	0.04	4.94E-01
CD34	3	0.25	0.12	3.14E-02
CD38	2	0.14	0.12	2.50E-01
CD4	4	-0.18	0.12	1.55E-01
CD40	9	-0.16	0.11	1.40E-01
CD40LG	12	-0.14	0.17	4.22E-01
CD46	7	-0.09	0.25	7.15E-01
CD48	8	-0.04	0.06	4.84E-01
CD5	8	-0.61	0.18	5.01E-04
CD55	6	0.08	0.10	4.15E-01
CD58	11	0.09	0.09	3.40E-01
CD59	5	0.11	0.17	5.24E-01
CD6	10	-0.01	0.06	8.95E-01
CD63	8	-0.33	0.17	5.72E-02
CD69	5	-0.23	0.24	3.45E-01
CD70	3	-0.11	0.06	5.33E-02
CD74	6	-0.74	0.17	1.23E-05
CD79B	13	0.16	0.13	2.32E-01
CD83	12	-0.11	0.15	4.78E-01
CD84	11	0.05	0.15	7.12E-01
CD8A	3	-0.35	0.16	2.62E-02
CD93	5	-0.80	0.31	1.05E-02
CD99	4	0.23	0.31	4.52E-01
CD99L2	8	-0.10	0.18	5.75E-01
CDC27	2	0.15	0.46	7.41E-01
CDC37	2	-0.48	0.52	3.55E-01
CDCP1	7	0.14	0.15	3.34E-01
CDH1	6	0.37	0.08	8.70E-06
CDH15	3	0.23	0.14	1.02E-01
CDH17	10	0.21	0.07	3.43E-03

CDH2	13	-0.16	0.17	3.54E-01
CDH3	6	0.09	0.28	7.54E-01
CDH5	11	-0.07	0.07	3.04E-01
CDH6	6	-0.10	0.08	2.37E-01
CDHR1	4	0.05	0.17	7.89E-01
CDHR2	6	1.36	0.34	6.20E-05
CDHR5	7	0.05	0.08	4.83E-01
CDKN1A	5	-0.12	0.24	6.18E-01
CDKN2D	4	-0.51	0.29	7.92E-02
CDNF	1	-0.07	0.11	5.20E-01
CDON	4	0.21	0.28	4.65E-01
CDSN	17	0.36	0.16	1.99E-02
CEACAM1	6	-0.05	0.15	7.50E-01
CEACAM21	2	-3.51	0.82	1.79E-05
CEACAM3	1	-1.12	0.63	7.57E-02
CEACAM5	10	-0.16	0.08	4.90E-02
CEACAM8	9	-0.12	0.16	4.70E-01
CEBPB	3	-0.67	0.43	1.25E-01
CELA3A	14	-0.25	0.11	1.74E-02
CEP20	4	-0.35	0.27	1.90E-01
CEP43	6	0.14	0.30	6.40E-01
CEP85	2	-0.21	0.33	5.19E-01
CERT	4	0.09	0.34	7.83E-01
CES1	16	0.45	0.17	8.93E-03
CES2	2	-0.63	0.32	4.90E-02
CES3	9	0.04	0.15	7.89E-01
CETN2	3	-0.22	0.36	5.29E-01
CFC1	6	0.04	0.30	9.06E-01
CGA	1	-1.10	1.13	3.31E-01
CGREF1	3	0.18	0.07	1.37E-02
CHAC2	2	0.60	0.31	4.92E-02
CHGB	5	0.01	0.07	8.73E-01
CHI3L1	5	-0.19	0.08	1.36E-02
CHIT1	3	0.02	0.05	7.35E-01
CHL1	6	0.06	0.12	6.25E-01
CHMP1A	4	-0.40	0.34	2.33E-01
CHRDL1	3	0.12	0.46	7.97E-01
CHRDL2	2	0.20	0.41	6.28E-01
CIAPIN1	4	-0.35	0.38	3.59E-01
CKAP4	4	-0.08	0.10	4.65E-01
CKMT1A_CKMT1B	6	0.60	0.24	1.34E-02
CLC	8	-0.29	0.18	1.11E-01
CLEC10A	9	-0.05	0.08	4.89E-01
CLEC11A	4	0.06	0.15	6.98E-01
CLEC14A	8	0.21	0.13	1.11E-01
CLEC1A	7	-0.25	0.09	3.93E-03
CLEC1B	9	-0.02	0.17	8.89E-01
CLEC4A	11	-0.01	0.08	8.68E-01
CLEC4C	25	0.02	0.05	6.13E-01
CLEC4D	10	0.07	0.07	3.42E-01
CLEC4G	10	-0.37	0.11	1.18E-03
CLEC5A	9	0.04	0.13	7.61E-01
CLEC6A	12	-0.05	0.08	5.31E-01
CLEC7A	3	-0.05	0.05	3.34E-01
CLIP2	2	0.15	0.32	6.34E-01
CLMP	4	0.00	0.13	9.76E-01
CLPS	7	-0.02	0.08	8.25E-01
CLSPN	1	0.48	0.43	2.72E-01
CLSTN2	3	0.14	0.11	1.98E-01
CLTA	1	-0.36	0.38	3.50E-01

CLUL1	8	0.05	0.08	5.32E-01
CNDP1	7	-0.16	0.10	1.01E-01
CNPY2	1	-0.15	0.33	6.57E-01
CNPY4	3	-0.15	0.24	5.37E-01
CNST	4	-0.37	0.43	3.89E-01
CNTN1	6	0.18	0.12	1.22E-01
CNTN2	6	0.02	0.05	7.54E-01
CNTN3	15	0.03	0.12	8.24E-01
CNTN4	8	0.29	0.13	3.20E-02
CNTN5	6	-0.03	0.10	7.40E-01
CNTNAP2	4	-0.03	0.08	6.98E-01
COL18A1	4	0.40	0.23	8.73E-02
COL1A1	9	0.51	0.20	1.11E-02
COL4A1	9	0.39	0.12	1.95E-03
COL6A3	3	0.09	0.44	8.44E-01
COL9A1	4	-0.48	0.23	4.12E-02
COLEC12	4	0.03	0.27	9.01E-01
COMP	6	0.33	0.15	3.10E-02
COMT	2	0.01	0.10	8.96E-01
CORO1A	3	0.45	0.42	2.78E-01
COX5B	5	0.03	0.26	9.01E-01
CPA1	16	0.05	0.13	6.62E-01
CPA2	4	0.19	0.11	9.03E-02
CPB1	10	0.14	0.13	2.86E-01
CPE	6	1.50	0.25	2.76E-09
CPM	9	0.35	0.21	9.53E-02
CPPED1	1	-0.11	0.07	1.07E-01
CPVL	8	0.04	0.06	5.43E-01
CPXM1	15	0.00	0.10	9.70E-01
CR2	20	0.00	0.12	9.68E-01
CRACR2A	4	0.12	0.27	6.50E-01
CRADD	4	-0.33	0.37	3.76E-01
CREG1	10	0.35	0.15	1.92E-02
CRELD2	11	-0.05	0.12	7.00E-01
CRH	19	-0.10	0.10	3.04E-01
CRHBP	4	-0.02	0.08	7.46E-01
CRIM1	4	0.14	0.32	6.55E-01
CRIP2	3	-0.01	0.25	9.67E-01
CRISP2	12	0.00	0.08	9.84E-01
CRKL	1	-0.19	0.69	7.80E-01
CRLF1	5	-1.02	0.33	2.17E-03
CRNN	3	-0.17	0.07	1.57E-02
CRTAC1	1	0.03	0.07	6.33E-01
CRTAM	11	-0.13	0.12	3.09E-01
CSF1	3	-0.21	0.21	3.27E-01
CSF2RA	1	0.23	0.58	6.89E-01
CSF3	2	-0.19	0.23	4.16E-01
CST3	6	-0.19	0.15	1.97E-01
CST5	13	0.01	0.06	8.83E-01
CST6	12	0.22	0.14	1.16E-01
CST7	10	-0.08	0.06	1.67E-01
CTF1	2	-0.10	0.59	8.66E-01
CTRB1	11	0.21	0.08	7.69E-03
CTRC	11	-0.09	0.07	2.29E-01
CTSB	7	0.07	0.08	4.15E-01
CTSC	7	-0.03	0.07	6.39E-01
CTSD	6	0.01	0.12	9.11E-01
CTSF	14	-0.10	0.11	3.94E-01
CTSH	1	0.30	0.51	5.60E-01
CTSL	15	0.06	0.14	6.76E-01

CTSO	9	-0.10	0.16	5.31E-01
CTSS	7	-0.18	0.24	4.58E-01
CTSV	16	0.10	0.13	4.65E-01
CTSZ	9	0.17	0.09	5.44E-02
CX3CL1	5	-0.10	0.29	7.28E-01
CXADR	5	0.62	0.18	4.17E-04
CXCL1	4	0.02	0.08	7.44E-01
CXCL10	3	-0.25	0.14	8.03E-02
CXCL11	6	-0.70	0.18	7.72E-05
CXCL12	9	-0.36	0.18	4.58E-02
CXCL13	7	-1.57	0.26	1.92E-09
CXCL14	2	-0.28	0.50	5.73E-01
CXCL16	11	0.21	0.15	1.52E-01
CXCL17	5	-0.24	0.32	4.50E-01
CXCL3	3	-0.02	0.36	9.64E-01
CXCL5	8	-0.12	0.09	1.98E-01
CXCL6	6	0.03	0.08	7.15E-01
CXCL8	6	-0.12	0.20	5.49E-01
CXCL9	5	-1.32	0.22	1.17E-09
DAB2	3	-0.17	0.43	6.91E-01
DAG1	3	0.06	0.49	9.08E-01
DAPP1	2	-0.33	0.38	3.90E-01
DARS1	4	-0.20	0.34	5.60E-01
DBI	3	-0.33	0.16	4.44E-02
DBNL	4	-0.27	0.40	5.05E-01
DCBLD2	15	-0.20	0.12	1.11E-01
DCN	1	0.45	0.72	5.31E-01
DCTN1	1	0.72	0.66	2.75E-01
DCTN2	2	-0.53	0.62	3.92E-01
DCTPP1	6	-0.14	0.24	5.77E-01
DCXR	6	0.10	0.27	7.15E-01
DDAH1	5	-0.26	0.22	2.34E-01
DDC	6	0.22	0.11	5.21E-02
DDR1	11	0.11	0.10	2.79E-01
DDX58	1	0.34	0.17	4.03E-02
DECR1	3	0.45	0.29	1.26E-01
DEFA1_DEFA1B	13	-0.50	0.17	3.23E-03
DEFB4A_DEFB4B	3	1.19	0.31	1.45E-04
DFFA	2	0.14	0.51	7.79E-01
DIABLO	3	0.38	0.48	4.32E-01
DKK1	9	-0.03	0.15	8.35E-01
DKK3	7	0.42	0.11	9.61E-05
DKK4	5	-0.02	0.14	8.73E-01
DKKL1	4	0.04	0.04	3.52E-01
DLK1	5	-0.08	0.09	3.73E-01
DLL1	6	-0.67	0.20	7.00E-04
DNAJA2	1	-0.80	0.73	2.71E-01
DNAJB1	5	-0.38	0.31	2.23E-01
DNAJB8	1	-1.05	0.55	5.50E-02
DNER	6	0.09	0.29	7.42E-01
DNMBP	3	-0.03	0.33	9.32E-01
DNPH1	1	-0.43	0.15	5.39E-03
DOK2	4	-0.08	0.35	8.15E-01
DPEP1	5	0.43	0.25	7.88E-02
DPEP2	3	-0.16	0.15	2.69E-01
DPP10	5	0.14	0.14	3.23E-01
DPP4	5	0.01	0.17	9.69E-01
DPP6	7	0.20	0.14	1.47E-01
DPP7	5	-0.04	0.18	8.43E-01
DPT	2	0.12	0.13	3.87E-01

DPY30	2	0.91	0.58	1.16E-01
DRAXIN	15	-0.06	0.12	5.89E-01
DRG2	3	-0.38	0.32	2.30E-01
DSC2	9	0.07	0.14	6.52E-01
DSG2	9	0.06	0.14	6.53E-01
DSG3	15	0.09	0.11	4.01E-01
DSG4	20	0.09	0.13	5.12E-01
DTX3	3	-0.36	0.23	1.23E-01
DUSP3	2	-0.55	0.48	2.54E-01
EBAG9	5	-0.47	0.31	1.26E-01
EBI3_IL27	14	-0.29	0.14	3.48E-02
ECE1	5	-0.09	0.21	6.77E-01
EDA2R	3	-0.55	0.50	2.75E-01
EDAR	20	0.02	0.08	8.20E-01
EDIL3	3	-0.01	0.25	9.77E-01
EFEMP1	3	0.16	0.22	4.70E-01
EFNA1	4	0.13	0.13	3.08E-01
EFNA4	4	-0.98	0.27	2.63E-04
EGF	8	-0.11	0.20	5.83E-01
EGFL7	17	-0.24	0.13	6.54E-02
EGFR	5	-0.24	0.31	4.48E-01
EGLN1	3	-0.34	0.26	1.96E-01
EIF4B	3	-0.37	0.38	3.32E-01
EIF4G1	3	-0.30	0.38	4.29E-01
ELOA	2	-0.06	0.31	8.48E-01
ENAH	1	0.83	0.42	4.77E-02
ENG	7	0.14	0.08	1.07E-01
ENO1	4	-0.09	0.25	7.19E-01
ENO2	5	-0.22	0.23	3.32E-01
ENPP2	8	0.18	0.15	2.37E-01
ENPP5	6	0.13	0.06	2.21E-02
ENPP7	6	-0.02	0.05	6.44E-01
ENTPD2	2	1.01	0.61	9.73E-02
ENTPD5	8	0.01	0.09	9.42E-01
ENTPD6	8	-0.15	0.05	2.46E-03
EPCAM	11	0.35	0.12	2.84E-03
EPHA1	7	-0.22	0.17	2.07E-01
EPHA2	6	-0.01	0.23	9.52E-01
EPHB4	9	0.07	0.12	5.53E-01
EPHB6	4	-0.13	0.14	3.68E-01
EPO	9	0.20	0.17	2.22E-01
EPS8L2	3	0.11	0.15	4.45E-01
ERBB2	8	-0.42	0.20	3.53E-02
ERBB3	8	-0.10	0.11	3.66E-01
ERBB4	14	0.10	0.14	4.93E-01
ERBIN	3	-1.12	0.48	1.95E-02
EREG	8	-0.21	0.22	3.30E-01
ERP44	5	-0.08	0.12	4.80E-01
ESAM	7	-0.13	0.14	3.71E-01
ESM1	10	-0.13	0.17	4.53E-01
EZR	2	-0.27	0.53	6.14E-01
F11R	11	-0.37	0.22	9.91E-02
F2R	7	-0.17	0.20	3.91E-01
F3	8	0.44	0.10	3.30E-05
F7	7	-0.02	0.08	7.71E-01
F9	2	-0.24	0.36	5.16E-01
FABP1	2	-0.18	0.20	3.50E-01
FABP2	5	0.19	0.18	2.92E-01
FABP4	1	-0.84	0.65	1.97E-01
FABP5	2	-0.03	0.37	9.41E-01

FABP6	3	0.24	0.17	1.64E-01
FABP9	15	0.14	0.09	1.07E-01
FADD	3	0.62	0.41	1.33E-01
FAM3B	6	-0.21	0.07	4.37E-03
FAM3C	1	-0.65	0.73	3.73E-01
FAP	5	-0.18	0.16	2.69E-01
FAS	3	-0.14	0.17	4.00E-01
FASLG	18	-0.42	0.10	3.26E-05
FBP1	5	0.14	0.14	2.93E-01
FCAR	13	-0.15	0.06	9.69E-03
FCER2	10	0.11	0.12	3.49E-01
FCGR2A	5	0.04	0.05	4.11E-01
FCGR2B	4	-0.11	0.09	2.04E-01
FCGR3B	12	-0.03	0.13	8.07E-01
FCN2	10	0.07	0.06	2.66E-01
FCRL1	22	-0.12	0.09	1.94E-01
FCRL2	12	-0.09	0.07	2.25E-01
FCRL3	5	-0.05	0.05	2.34E-01
FCRL5	9	0.32	0.17	5.84E-02
FCRL6	5	-0.02	0.06	7.05E-01
FCRLB	6	0.01	0.07	8.86E-01
FEN1	1	0.96	0.83	2.50E-01
FES	1	1.78	0.33	4.80E-08
FETUB	3	-0.53	0.29	6.88E-02
FGF19	4	0.54	0.16	8.31E-04
FGF2	6	-0.11	0.06	7.85E-02
FGF21	7	-0.25	0.18	1.79E-01
FGF23	4	-0.05	0.26	8.57E-01
FGF5	2	-0.22	0.07	1.62E-03
FGFBP1	10	0.09	0.22	6.86E-01
FGFR2	7	-0.20	0.24	4.02E-01
FGR	1	-0.54	0.48	2.57E-01
FHIT	2	0.02	0.41	9.66E-01
FIS1	4	-0.04	0.24	8.54E-01
FKBP1B	4	0.26	0.18	1.46E-01
FKBP4	5	-0.87	0.32	5.97E-03
FKBP5	5	-0.16	0.20	4.13E-01
FLI1	3	-0.10	0.42	8.12E-01
FLRT2	11	0.10	0.07	1.70E-01
FLT1	9	-0.11	0.11	2.97E-01
FLT3	5	0.24	0.27	3.68E-01
FLT3LG	19	0.03	0.11	7.67E-01
FLT4	10	-0.06	0.06	2.68E-01
FOLR1	7	-0.45	0.21	3.42E-02
FOLR2	14	-0.13	0.13	3.12E-01
FOLR3	9	0.07	0.22	7.68E-01
FOXO1	3	0.15	0.28	5.87E-01
FOXO3	1	1.31	0.79	9.80E-02
FRZB	2	-0.13	0.10	1.82E-01
FST	3	-0.06	0.24	7.91E-01
FSTL3	2	-0.02	0.42	9.69E-01
FUCA1	5	0.00	0.04	9.64E-01
FURIN	3	-1.05	0.20	2.19E-07
FUS	2	0.38	0.44	3.83E-01
FUT3_FUT5	11	0.11	0.08	1.80E-01
FUT8	11	-0.01	0.06	8.13E-01
FXN	3	-0.28	0.46	5.45E-01
FXYD5	2	-0.28	0.47	5.55E-01
FYB1	2	-0.22	0.50	6.56E-01
GAL	17	0.00	0.11	9.68E-01

GALNT10	9	-0.16	0.11	1.42E-01
GALNT2	6	0.24	0.13	6.05E-02
GALNT3	8	0.04	0.10	6.89E-01
GALNT7	5	0.01	0.14	9.54E-01
GAS6	15	0.02	0.10	7.99E-01
GBP2	1	0.00	0.43	9.96E-01
GCG	5	0.34	0.35	3.27E-01
GCNT1	4	-1.13	0.26	1.01E-05
GDF15	1	-0.21	0.16	1.87E-01
GDF2	11	0.16	0.17	3.48E-01
GDNF	1	-0.20	0.19	2.91E-01
GFAP	7	-0.86	0.23	1.42E-04
GFRA1	4	-0.06	0.16	6.91E-01
GFRA2	6	-0.10	0.13	4.09E-01
GFRA3	20	0.17	0.13	1.81E-01
GGH	9	0.07	0.10	4.60E-01
GGT1	7	0.36	0.14	1.16E-02
GGT5	4	0.49	0.15	9.64E-04
GHRL	8	0.25	0.16	1.11E-01
GKN1	4	0.82	0.33	1.27E-02
GLB1	8	-0.04	0.11	7.14E-01
GLO1	1	-0.21	0.13	1.15E-01
GLOD4	2	-0.19	0.58	7.45E-01
GLRX	2	0.11	0.09	2.04E-01
GLT8D2	1	0.21	0.25	3.93E-01
GMPR	2	0.30	0.17	8.39E-02
GNE	3	-0.02	0.30	9.40E-01
GNLY	8	-0.04	0.07	5.93E-01
GOLM2	8	0.08	0.12	4.75E-01
GOPC	2	-0.15	0.48	7.55E-01
GP1BA	14	-0.71	0.15	4.34E-06
GP2	8	-0.18	0.07	1.30E-02
GP6	6	-0.26	0.13	4.69E-02
GPA33	10	0.23	0.11	3.41E-02
GPC1	14	-0.26	0.18	1.54E-01
GPC5	10	-0.12	0.06	2.79E-02
GPNMB	5	0.18	0.12	1.27E-01
GPR37	10	-0.10	0.06	1.16E-01
GRAP2	3	0.25	0.42	5.58E-01
GRK5	1	0.04	0.17	7.87E-01
GRN	10	-0.37	0.07	1.73E-08
GRPEL1	3	0.00	0.37	9.98E-01
GSAP	1	0.50	0.72	4.83E-01
GSTA1	9	0.12	0.12	3.31E-01
GSTA3	5	0.09	0.13	4.90E-01
GSTP1	1	-0.10	0.21	6.25E-01
GUCA2A	7	-0.29	0.17	8.18E-02
GUSB	10	0.04	0.12	7.36E-01
GYS1	3	-0.05	0.17	7.86E-01
GZMA	13	-0.12	0.13	3.38E-01
GZMB	8	-0.92	0.21	1.28E-05
GZMH	7	0.42	0.17	1.47E-02
HAGH	1	-0.24	0.37	5.13E-01
HAO1	8	-0.30	0.25	2.31E-01
HARS1	2	-0.35	0.49	4.84E-01
HAVCR1	6	-0.21	0.09	1.79E-02
HAVCR2	8	-0.14	0.14	3.27E-01
HBEGF	20	-0.12	0.12	3.46E-01
HBQ1	2	-0.24	0.12	4.39E-02
HCLS1	3	0.61	0.38	1.01E-01

HDGF	2	0.06	0.04	1.10E-01
HEBP1	1	0.26	0.36	4.62E-01
HEXIM1	4	-0.11	0.27	6.87E-01
HGF	5	-0.23	0.22	2.99E-01
HGS	4	-0.48	0.39	2.20E-01
HLA-DRA	3	-0.28	0.08	6.33E-04
HLA-E	4	-0.26	0.11	2.53E-02
HMOX1	15	0.23	0.15	1.34E-01
HMOX2	4	-1.28	0.27	1.80E-06
HNMT	4	-0.04	0.06	5.42E-01
HNRNPK	1	-0.21	0.74	7.80E-01
HPGDS	1	-0.44	0.68	5.16E-01
HS3ST3B1	7	-0.15	0.12	1.96E-01
HS6ST1	14	-0.02	0.12	8.67E-01
HSD11B1	7	-0.08	0.14	5.90E-01
HSPA1A	3	-0.36	0.33	2.78E-01
HSPB1	5	0.00	0.28	9.96E-01
HSPB6	4	0.74	0.45	9.90E-02
HSPG2	4	0.07	0.24	7.85E-01
HTRA2	1	-0.24	0.84	7.80E-01
HYAL1	4	-0.14	0.15	3.57E-01
HYOU1	13	-0.33	0.14	2.39E-02
ICA1	2	0.07	0.21	7.42E-01
ICAM1	6	0.05	0.10	6.37E-01
ICAM2	9	0.01	0.04	8.77E-01
ICAM3	20	0.02	0.09	7.89E-01
ICAM4	14	-0.13	0.06	3.25E-02
ICAM5	5	0.19	0.06	6.60E-04
ICOSLG	4	0.07	0.11	5.30E-01
IDI2	1	0.28	0.14	3.57E-02
IDS	3	0.18	0.20	3.58E-01
IDUA	13	0.10	0.07	1.56E-01
IFNG	2	0.11	0.57	8.54E-01
IFNGR1	10	-0.14	0.14	2.93E-01
IFNGR2	8	-0.12	0.04	4.56E-03
IFNL1	3	0.13	0.36	7.24E-01
IFNLR1	2	-1.03	0.44	1.98E-02
IGF1R	9	0.17	0.10	8.89E-02
IGF2R	14	-0.01	0.10	9.01E-01
IGFBP1	2	-1.86	0.44	2.73E-05
IGFBP2	2	0.21	0.53	6.97E-01
IGFBP3	5	-0.03	0.09	7.73E-01
IGFBP4	3	-0.05	0.47	9.21E-01
IGFBP6	3	-0.27	0.46	5.56E-01
IGFBP7	3	0.39	0.13	2.76E-03
IGFBPL1	2	0.14	0.11	2.16E-01
IGSF3	5	0.00	0.22	9.92E-01
IGSF8	5	-0.40	0.23	8.13E-02
IKBKG	1	-0.17	0.89	8.52E-01
IKZF2	1	0.09	0.43	8.25E-01
IL10	5	-0.44	0.18	1.54E-02
IL10RA	1	-0.51	0.31	1.01E-01
IL10RB	6	0.07	0.08	4.07E-01
IL11	1	-0.88	0.81	2.82E-01
IL12A_IL12B	15	-0.12	0.08	1.11E-01
IL12B	16	-0.06	0.07	4.32E-01
IL12RB1	3	-0.06	0.09	4.98E-01
IL13	1	-0.30	0.49	5.33E-01
IL13RA1	2	-0.40	0.47	4.02E-01
IL15	21	0.29	0.13	2.27E-02

IL15RA	1	-0.01	0.19	9.74E-01
IL16	1	-0.74	0.68	2.77E-01
IL17C	2	0.23	0.33	4.85E-01
IL17D	2	-0.10	0.12	3.93E-01
IL17F	1	-1.52	0.70	2.87E-02
IL17RA	3	-0.03	0.05	5.36E-01
IL17RB	9	-0.01	0.05	8.17E-01
IL18	8	0.16	0.11	1.42E-01
IL18BP	9	-0.70	0.18	1.28E-04
IL18R1	12	-0.07	0.06	2.05E-01
IL18RAP	3	-0.22	0.28	4.43E-01
IL19	3	-0.21	0.09	2.07E-02
IL1A	1	-0.61	0.39	1.16E-01
IL1B	2	0.17	0.17	3.26E-01
IL1R1	13	-0.22	0.10	3.32E-02
IL1R2	13	0.05	0.08	5.25E-01
IL1RAP	3	-0.03	0.53	9.62E-01
IL1RL1	12	0.01	0.06	8.51E-01
IL1RL2	12	-0.04	0.09	6.67E-01
IL1RN	2	-0.19	0.20	3.42E-01
IL20RA	1	1.28	0.61	3.58E-02
IL22RA1	2	0.26	0.15	8.23E-02
IL2RA	8	-0.18	0.08	1.80E-02
IL32	7	0.03	0.15	8.26E-01
IL33	1	-2.28	0.84	6.52E-03
IL34	1	0.02	0.06	7.41E-01
IL3RA	4	-0.39	0.29	1.77E-01
IL4R	5	-0.07	0.12	5.51E-01
IL5RA	22	0.13	0.09	1.42E-01
IL6	4	0.25	0.17	1.57E-01
IL6R	5	0.08	0.04	3.72E-02
IL6ST	11	0.09	0.11	4.07E-01
IL7	3	-0.29	0.35	4.04E-01
IL7R	10	0.10	0.05	2.39E-02
ILKAP	1	-0.21	0.76	7.80E-01
IMPA1	2	-0.21	0.22	3.23E-01
ING1	1	-0.23	0.83	7.80E-01
INHBC	6	-0.14	0.08	6.50E-02
INPP1	3	0.15	0.33	6.51E-01
IPCEF1	2	-0.44	0.42	2.98E-01
IQGAP2	1	0.32	0.30	2.96E-01
IRAG2	1	1.07	0.67	1.07E-01
IRAK1	1	-0.14	0.77	8.52E-01
IRAK4	1	0.26	0.61	6.66E-01
ISLR2	7	0.22	0.08	3.44E-03
ISM1	1	-0.04	0.25	8.66E-01
ITGA11	4	0.00	0.16	9.81E-01
ITGA5	9	-0.44	0.18	1.40E-02
ITGA6	3	0.22	0.17	1.99E-01
ITGAM	10	0.04	0.15	7.94E-01
ITGAV	6	0.18	0.20	3.67E-01
ITGB1	6	0.32	0.20	9.82E-02
ITGB1BP2	4	-0.81	0.32	1.04E-02
ITGB2	10	-0.06	0.14	6.85E-01
ITGB5	5	-0.04	0.28	8.76E-01
ITGB6	9	0.26	0.14	6.00E-02
ITGB7	16	0.04	0.08	6.22E-01
ITIH3	7	0.01	0.12	9.55E-01
ITM2A	1	-0.06	0.20	7.53E-01
JAM2	4	0.71	0.25	4.91E-03

JCHAIN	12	-0.04	0.20	8.30E-01
KAZALD1	3	-0.21	0.45	6.41E-01
KDR	11	-0.03	0.08	7.00E-01
KEL	14	0.18	0.09	3.88E-02
KIFBP	3	0.05	0.30	8.63E-01
KIR2DL3	2	-1.98	0.46	1.44E-05
KIR3DL1	2	0.02	0.05	6.05E-01
KIRREL2	7	-0.24	0.11	2.63E-02
KIT	18	-0.02	0.12	8.94E-01
KITLG	15	0.28	0.11	1.22E-02
KLB	7	-0.04	0.06	4.73E-01
KLK1	12	-0.14	0.05	4.01E-03
KLK10	3	0.06	0.08	3.92E-01
KLK11	2	0.40	0.49	4.16E-01
KLK12	5	0.01	0.04	7.57E-01
KLK13	10	0.00	0.09	9.62E-01
KLK14	9	0.11	0.10	2.77E-01
KLK4	3	0.05	0.11	6.50E-01
KLK6	2	-0.05	0.23	8.15E-01
KLK8	3	-0.05	0.10	5.93E-01
KLRB1	10	-0.40	0.17	1.67E-02
KLRD1	10	-0.04	0.08	6.39E-01
KRT18	6	-0.06	0.30	8.53E-01
KRT19	3	0.70	0.27	9.74E-03
KRT5	4	0.18	0.22	4.11E-01
KYAT1	4	0.00	0.32	1.00E+00
KYNU	3	0.15	0.17	3.70E-01
L1CAM	6	0.06	0.18	7.39E-01
LACTB2	3	-0.04	0.22	8.41E-01
LAG3	10	-0.66	0.14	2.06E-06
LAIR1	3	-2.67	0.52	2.97E-07
LAIR2	3	-0.03	0.06	5.91E-01
LAMA4	11	0.15	0.14	2.56E-01
LAMP2	2	-1.05	0.56	6.00E-02
LAMP3	8	-0.77	0.27	3.64E-03
LAT	5	-0.66	0.29	2.47E-02
LAT2	1	-0.13	0.71	8.52E-01
LAYN	3	0.15	0.11	1.84E-01
LBP	2	0.04	0.07	5.84E-01
LBR	5	0.01	0.21	9.49E-01
LCN2	16	0.15	0.15	3.27E-01
LDLR	15	-0.96	0.15	1.91E-10
LEFTY2	3	3.03	0.43	2.41E-12
LEP	1	-2.50	1.01	1.32E-02
LEPR	9	-0.22	0.14	1.27E-01
LGALS1	2	0.06	0.16	6.95E-01
LGALS3	4	-0.91	0.31	3.77E-03
LGALS4	3	0.19	0.09	2.70E-02
LGALS7_LGALS7B	6	-0.22	0.11	4.70E-02
LGALS8	6	0.03	0.09	7.15E-01
LGALS9	3	-1.67	0.32	1.65E-07
LGMN	19	-0.18	0.12	1.40E-01
LHB	1	-0.49	0.50	3.31E-01
LIFR	10	0.26	0.10	1.40E-02
LILRA2	11	0.00	0.07	9.66E-01
LILRA5	9	-0.12	0.18	5.10E-01
LILRB1	7	0.12	0.07	1.13E-01
LILRB2	9	0.05	0.05	2.97E-01
LILRB4	2	-2.18	0.51	2.12E-05
LILRB5	2	-0.02	0.04	5.49E-01

LPCAT2	1	-0.68	0.40	8.96E-02
LPL	6	0.31	0.11	4.23E-03
LPO	13	-0.20	0.09	2.79E-02
LRIG1	10	-0.01	0.05	7.95E-01
LRP1	10	-0.56	0.17	1.07E-03
LRP11	6	0.04	0.07	5.98E-01
LRPAP1	7	0.05	0.06	4.22E-01
LRRC25	12	0.07	0.09	4.09E-01
LRRN1	6	-0.02	0.06	7.42E-01
LSP1	2	-0.97	0.28	6.70E-04
LTA	8	-0.08	0.06	1.83E-01
LTA4H	1	0.07	0.45	8.75E-01
LTBP2	8	0.00	0.22	9.96E-01
LTBP3	5	-0.22	0.09	1.16E-02
LTBR	13	-0.24	0.10	1.40E-02
LXN	1	0.42	0.26	1.10E-01
LY6D	5	-0.13	0.10	1.83E-01
LY75	6	0.07	0.05	1.21E-01
LY9	11	-0.05	0.08	4.83E-01
LY96	10	-0.06	0.19	7.49E-01
LYAR	1	-1.39	0.84	9.86E-02
LYN	5	-0.50	0.34	1.45E-01
LYPD3	17	0.05	0.08	5.03E-01
LYPD8	7	0.11	0.08	1.60E-01
MAD1L1	1	-0.48	0.35	1.76E-01
MAGED1	2	-0.41	0.45	3.59E-01
MANF	6	-0.34	0.31	2.63E-01
MANSC1	12	-0.05	0.18	7.93E-01
MAP2K6	2	-0.27	0.51	6.04E-01
MAP3K5	1	-0.15	0.80	8.52E-01
MAP4K5	3	-0.08	0.23	7.48E-01
MARCO	9	-0.05	0.11	6.57E-01
MASP1	10	0.20	0.11	6.67E-02
MATN2	5	0.12	0.10	2.19E-01
MATN3	3	0.01	0.08	9.23E-01
MAVS	3	-0.57	0.38	1.28E-01
MAX	5	-0.31	0.32	3.32E-01
MB	8	0.16	0.28	5.75E-01
MCAM	8	0.16	0.15	3.10E-01
MCFD2	4	-0.04	0.23	8.72E-01
MDGA1	6	0.10	0.04	1.09E-02
MDK	13	-0.33	0.17	4.79E-02
MED18	1	0.06	0.76	9.33E-01
MEGF10	11	0.20	0.14	1.55E-01
MEGF9	11	0.17	0.11	1.24E-01
MEP1B	14	0.00	0.04	9.22E-01
MEPE	11	0.01	0.18	9.64E-01
MERTK	12	-0.14	0.13	2.67E-01
MESD	4	-0.67	0.35	5.50E-02
MET	17	0.14	0.11	2.08E-01
METAP1D	3	0.68	0.42	1.04E-01
METAP2	4	-0.26	0.28	3.57E-01
MFAP5	4	0.56	0.19	3.90E-03
MFGE8	11	-0.10	0.10	3.38E-01
MGLL	3	0.20	0.39	6.00E-01
MGMT	3	0.10	0.11	3.62E-01
MIA	5	0.02	0.06	7.71E-01
MICB_MICA	8	-0.05	0.05	2.33E-01
MIF	3	-0.09	0.32	7.86E-01
MILR1	8	0.17	0.10	9.58E-02

MITD1	2	-0.09	0.49	8.45E-01
MLN	13	0.16	0.17	3.37E-01
MME	13	-0.15	0.08	5.22E-02
MMP1	10	-0.07	0.09	4.17E-01
MMP10	2	0.11	0.13	4.01E-01
MMP12	2	-0.08	0.09	3.76E-01
MMP13	1	0.30	0.32	3.35E-01
MMP3	4	0.14	0.09	1.33E-01
MMP7	5	-0.06	0.14	6.89E-01
MMP8	2	-0.12	0.11	2.74E-01
MMP9	9	0.10	0.15	5.03E-01
MNDA	1	-2.02	0.89	2.40E-02
MOG	2	-0.32	0.22	1.35E-01
MPHOSPH8	2	-0.47	0.48	3.34E-01
MPI	4	-0.03	0.19	8.80E-01
MPIG6B	4	-0.19	0.29	5.25E-01
MPO	6	-0.62	0.29	3.40E-02
MSLN	7	-0.07	0.07	3.23E-01
MSMB	5	0.06	0.05	2.27E-01
MSR1	11	-0.04	0.10	7.02E-01
MSRA	2	0.12	0.53	8.25E-01
MSTN	6	0.48	0.31	1.22E-01
MUC13	4	0.33	0.14	2.34E-02
MUC16	1	1.09	0.65	9.56E-02
MVK	1	-0.85	0.52	1.02E-01
MYOC	9	0.52	0.13	1.09E-04
MZB1	7	0.01	0.22	9.69E-01
MZT1	2	-0.04	0.57	9.40E-01
NAAA	15	0.03	0.06	6.08E-01
NADK	5	0.03	0.14	8.20E-01
NBL1	3	-0.26	0.33	4.30E-01
NBN	3	0.54	0.38	1.53E-01
NCAM1	7	-0.08	0.14	5.82E-01
NCAM2	5	0.19	0.19	3.22E-01
NCAN	9	-0.19	0.18	2.89E-01
NCF2	4	0.09	0.18	6.16E-01
NCK2	1	0.80	0.88	3.67E-01
NCR1	22	-0.29	0.10	4.69E-03
NCS1	7	0.06	0.25	8.07E-01
NECTIN2	4	0.30	0.18	8.87E-02
NECTIN4	6	-0.10	0.14	4.85E-01
NEFL	2	-1.45	0.70	3.77E-02
NELL1	16	0.02	0.09	8.27E-01
NELL2	13	0.11	0.18	5.28E-01
NFASC	4	0.00	0.11	1.00E+00
NFATC1	2	-0.01	0.36	9.77E-01
NFATC3	1	1.94	0.76	1.03E-02
NFKBIE	2	-0.03	0.20	8.89E-01
NID1	9	-0.12	0.15	4.35E-01
NID2	12	0.06	0.07	3.62E-01
NINJ1	1	0.05	0.63	9.38E-01
NME3	12	0.12	0.11	2.46E-01
NMNAT1	4	-0.02	0.16	8.79E-01
NOMO1	3	-0.22	0.30	4.65E-01
NOS1	1	-0.35	0.71	6.23E-01
NOS3	2	-0.28	0.52	5.96E-01
NOTCH1	7	-0.08	0.14	5.74E-01
NOTCH3	11	0.35	0.19	6.35E-02
NPDC1	5	-0.63	0.35	7.46E-02
NPPB	2	0.64	0.23	6.26E-03

NPPC	2	2.24	0.66	7.24E-04
NPTX1	12	0.17	0.08	2.87E-02
NPTXR	3	0.01	0.08	8.66E-01
NPY	9	-0.21	0.18	2.55E-01
NRCAM	7	-0.04	0.22	8.63E-01
NRP1	6	0.21	0.13	1.10E-01
NRP2	9	0.01	0.19	9.45E-01
NRTN	1	0.73	0.81	3.63E-01
NSFL1C	2	-0.06	0.43	8.85E-01
NT5C3A	4	-0.19	0.31	5.35E-01
NT5E	8	-0.07	0.08	3.30E-01
NTF3	4	0.05	0.21	8.19E-01
NTF4	1	-2.67	0.85	1.76E-03
NTproBNP	4	0.42	0.16	7.48E-03
NTRK2	7	0.20	0.25	4.22E-01
NTRK3	9	0.38	0.16	1.55E-02
NUB1	4	0.00	0.21	9.96E-01
NUCB2	5	-0.10	0.11	3.37E-01
NUDC	2	-0.37	0.44	4.01E-01
NUDT2	2	-0.13	0.18	4.73E-01
NUDT5	2	0.33	0.50	5.13E-01
OBP2B	11	0.02	0.08	8.30E-01
ODAM	2	-0.05	0.17	7.81E-01
OGFR	2	-0.33	0.33	3.17E-01
OGN	4	0.14	0.08	6.50E-02
OLR1	6	-0.04	0.24	8.49E-01
OMD	5	-0.06	0.23	7.93E-01
OMG	8	0.50	0.20	1.42E-02
OPTC	7	-0.04	0.15	8.10E-01
OSCAR	8	-0.03	0.08	7.23E-01
OSM	8	-0.38	0.16	2.08E-02
OSMR	5	0.02	0.08	8.44E-01
OXT	9	-0.10	0.08	2.19E-01
P4HB	3	-0.15	0.42	7.30E-01
PADI2	1	-0.55	0.36	1.27E-01
PADI4	1	0.13	0.17	4.59E-01
PAEP	3	-0.05	0.06	4.12E-01
PAG1	6	-1.17	0.26	5.81E-06
PAK4	1	-0.16	0.86	8.52E-01
PAM	4	0.13	0.09	1.35E-01
PAMR1	4	0.16	0.11	1.65E-01
PAPPA	11	0.04	0.15	7.65E-01
PARK7	5	-0.07	0.23	7.72E-01
PARP1	2	0.21	0.26	4.28E-01
PCDH1	5	-0.09	0.25	7.32E-01
PCDH17	8	-0.25	0.09	4.77E-03
PCOLCE	1	-0.01	0.26	9.69E-01
PCSK9	8	-0.63	0.13	6.42E-07
PDCD1	10	-0.11	0.22	6.04E-01
PDCD1LG2	8	-0.10	0.06	7.41E-02
PDCD5	2	-0.23	0.12	6.06E-02
PDCD6	4	0.01	0.04	8.46E-01
PDGFA	16	-0.15	0.13	2.27E-01
PDGFB	13	0.01	0.15	9.24E-01
PDGFC	4	-0.14	0.15	3.46E-01
PDGFRA	10	0.16	0.13	2.26E-01
PDGFRB	2	0.01	0.04	8.40E-01
PDLIM7	3	-0.65	0.40	1.07E-01
PEAR1	7	-0.38	0.16	1.65E-02
PEBP1	2	-0.30	0.57	5.99E-01

PECAM1	7	0.06	0.09	4.78E-01
PFKFB2	4	-0.48	0.21	2.07E-02
PGF	3	-0.46	0.41	2.63E-01
PGLYRP1	11	-0.28	0.13	2.44E-02
PI3	4	0.02	0.15	8.93E-01
PIGR	6	0.08	0.20	7.00E-01
PIK3AP1	6	-0.01	0.19	9.72E-01
PIK3IP1	3	0.29	0.15	5.13E-02
PILRA	6	0.05	0.05	2.70E-01
PILRB	4	0.06	0.04	1.68E-01
PKLR	4	0.03	0.21	9.02E-01
PLA2G10	13	0.40	0.10	2.87E-05
PLA2G15	8	0.27	0.12	2.33E-02
PLA2G1B	17	-0.05	0.14	7.16E-01
PLA2G2A	6	-0.02	0.07	7.74E-01
PLA2G4A	2	0.27	0.54	6.20E-01
PLA2G7	9	-1.41	0.21	3.69E-11
PLAT	4	-0.85	0.33	1.08E-02
PLAU	8	-0.06	0.12	6.27E-01
PLAUR	9	-0.31	0.16	5.37E-02
PLIN1	1	-0.86	0.53	1.07E-01
PLIN3	2	-0.72	0.50	1.55E-01
PLPBP	2	-0.16	0.50	7.46E-01
PLTP	11	0.03	0.09	7.39E-01
PLXDC1	3	-0.03	0.18	8.49E-01
PLXNA4	5	-0.40	0.24	1.01E-01
PLXNB2	11	-0.01	0.06	9.25E-01
PLXNB3	5	-0.04	0.35	8.98E-01
PM20D1	6	0.39	0.27	1.46E-01
PMVK	6	0.05	0.26	8.43E-01
PNLIPRP2	7	-0.12	0.04	1.85E-03
PNPT1	2	0.09	0.48	8.56E-01
PODXL	6	0.18	0.08	2.08E-02
PODXL2	11	-0.30	0.18	9.31E-02
POLR2F	1	-5.94	0.85	3.11E-12
PON2	3	-0.02	0.06	7.25E-01
PON3	9	0.03	0.11	7.64E-01
PPCDC	2	0.16	0.13	2.24E-01
PPIB	3	-0.18	0.43	6.85E-01
PPME1	1	0.28	0.84	7.41E-01
PPP1R12A	3	-1.01	0.44	2.27E-02
PPP1R2	3	-0.07	0.41	8.73E-01
PPP1R9B	2	-0.43	0.53	4.17E-01
PPP3R1	4	0.34	0.30	2.63E-01
PPY	6	0.28	0.19	1.37E-01
PRCP	12	-0.02	0.12	8.76E-01
PRDX1	2	-0.04	0.48	9.41E-01
PRDX3	1	-1.01	0.70	1.49E-01
PRDX5	2	0.14	0.29	6.30E-01
PRDX6	1	0.31	0.32	3.40E-01
PRELP	1	-0.06	0.10	5.48E-01
PRKAB1	1	-0.16	0.84	8.52E-01
PRKAR1A	4	-0.11	0.35	7.50E-01
PRKRA	1	-0.08	0.44	8.52E-01
PRL	3	0.48	0.41	2.41E-01
PROC	7	-0.18	0.16	2.67E-01
PROK1	2	0.12	0.12	3.49E-01
PRSS2	11	-0.08	0.16	6.25E-01
PRSS27	16	-0.10	0.06	1.14E-01
PRSS8	7	-0.02	0.22	9.20E-01

PRTFDC1	3	0.05	0.21	8.06E-01
PRTG	8	0.01	0.09	8.74E-01
PRTN3	12	-0.27	0.19	1.56E-01
PSG1	6	0.11	0.28	7.00E-01
PSIP1	2	-0.17	0.33	5.99E-01
PSME1	2	-0.26	0.45	5.64E-01
PSME2	1	-0.28	0.49	5.70E-01
PSMG3	1	-0.19	0.88	8.27E-01
PSPN	7	-1.03	0.33	2.11E-03
PSRC1	2	0.14	0.25	5.61E-01
PTGDS	2	-0.21	0.61	7.31E-01
PTH1R	1	-0.01	0.19	9.41E-01
PTN	3	0.19	0.26	4.59E-01
PTPN1	2	-0.31	0.45	4.89E-01
PTPN6	2	-0.47	0.47	3.27E-01
PTPRF	12	0.47	0.15	1.97E-03
PTPRM	8	0.13	0.08	8.44E-02
PTPRN2	1	0.10	0.29	7.29E-01
PTPRS	5	0.16	0.15	2.70E-01
PTS	4	0.09	0.19	6.44E-01
PTX3	5	-0.02	0.12	8.81E-01
PVALB	6	-0.03	0.06	5.55E-01
PVR	2	-0.07	0.06	2.80E-01
PXN	3	0.01	0.07	8.99E-01
QDPR	1	-0.14	0.73	8.52E-01
QPCT	11	0.28	0.12	1.82E-02
RABEPK	1	0.03	0.30	9.09E-01
RABGAP1L	2	0.01	0.47	9.82E-01
RAD23B	3	-0.10	0.44	8.27E-01
RARRES1	1	-0.05	0.10	6.07E-01
RARRES2	3	0.34	0.21	1.07E-01
RASSF2	1	0.33	0.47	4.82E-01
RBKS	1	0.10	0.12	4.33E-01
RBP2	4	0.20	0.31	5.13E-01
RBP5	3	-0.11	0.14	4.35E-01
RCOR1	1	-1.91	0.75	1.11E-02
REG1A	6	0.09	0.11	4.10E-01
REG1B	7	0.04	0.10	7.04E-01
REG3A	8	-0.05	0.14	7.33E-01
REG4	2	0.13	0.25	6.02E-01
RELT	4	0.18	0.13	1.73E-01
REN	2	-0.07	0.28	8.18E-01
RET	8	-0.21	0.11	4.64E-02
RETN	15	0.00	0.12	9.72E-01
RGMA	17	0.05	0.10	6.01E-01
RGMB	4	-0.25	0.22	2.63E-01
RGS8	1	0.31	0.26	2.32E-01
RHOC	3	-0.47	0.52	3.66E-01
RILP	3	-0.21	0.39	5.92E-01
RNASE3	4	0.01	0.16	9.60E-01
RNASET2	7	-0.05	0.10	6.25E-01
ROBO1	8	0.03	0.16	8.54E-01
ROBO2	5	0.35	0.27	1.95E-01
ROR1	12	0.18	0.12	1.38E-01
RP2	2	-3.85	0.39	2.29E-23
RRM2	1	0.42	0.74	5.75E-01
RRM2B	1	-0.09	0.49	8.52E-01
RSPO1	8	-0.16	0.15	2.88E-01
RSPO3	8	-0.14	0.20	4.70E-01
RTBDN	2	0.09	0.27	7.34E-01

RTN4R	9	-0.02	0.14	9.01E-01
RUVBL1	1	-0.33	0.20	8.98E-02
RWDD1	2	-0.03	0.30	9.23E-01
S100A11	2	0.07	0.19	7.29E-01
S100A12	4	0.19	0.17	2.50E-01
S100A16	3	0.61	0.31	4.63E-02
S100A4	4	-0.67	0.41	1.06E-01
S100P	5	1.13	0.28	4.99E-05
SAMD9L	2	-0.06	0.15	7.04E-01
SCAMP3	4	0.08	0.32	8.03E-01
SCARA5	6	-0.09	0.14	5.27E-01
SCARB1	1	-0.75	0.83	3.69E-01
SCARB2	4	-0.38	0.18	3.43E-02
SCARF1	7	-0.12	0.11	2.57E-01
SCARF2	8	0.15	0.13	2.52E-01
SCG2	4	-0.83	0.36	2.09E-02
SCG3	5	-0.10	0.07	1.70E-01
SCGB1A1	15	-0.10	0.11	3.76E-01
SCGB3A2	15	0.30	0.13	2.17E-02
SCGN	3	0.70	0.23	2.51E-03
SCLY	6	0.14	0.34	6.88E-01
SCP2	1	0.03	0.84	9.68E-01
SCRN1	2	-0.06	0.32	8.56E-01
SDC1	13	0.19	0.14	1.74E-01
SDC4	20	0.02	0.11	8.49E-01
SELE	9	0.05	0.06	3.32E-01
SELP	10	0.04	0.12	7.32E-01
SELPLG	10	-0.17	0.08	3.14E-02
SEMA3F	5	-0.25	0.31	4.27E-01
SEMA4C	5	0.41	0.19	2.86E-02
SEMA4D	6	0.11	0.26	6.67E-01
SEMA7A	17	-0.28	0.12	2.32E-02
SEPTIN9	2	0.92	0.41	2.56E-02
SERPINA11	8	0.07	0.07	3.03E-01
SERPINA12	5	-0.12	0.07	7.32E-02
SERPINA9	3	-0.13	0.10	1.88E-01
SERPINB1	3	-0.73	0.36	4.37E-02
SERPINB6	1	-0.22	0.78	7.80E-01
SERPINB8	2	0.05	0.06	3.88E-01
SERPINB9	2	0.56	0.34	1.05E-01
SERPINE1	14	-0.07	0.14	6.06E-01
SESTD1	3	-0.15	0.35	6.73E-01
SETMAR	2	-0.43	0.20	2.81E-02
SEZ6L	7	-0.34	0.21	9.70E-02
SEZ6L2	8	0.44	0.18	1.33E-02
SF3B4	4	0.50	0.36	1.70E-01
SFRP1	5	-0.16	0.20	4.11E-01
SFTPA1	1	-0.10	0.20	6.01E-01
SFTPA2	6	-0.01	0.07	8.63E-01
SFTPD	10	0.03	0.05	5.71E-01
SH2B3	4	-0.57	0.35	1.04E-01
SH2D1A	2	-1.46	0.38	1.08E-04
SHMT1	4	-0.07	0.06	2.77E-01
SIAE	8	0.12	0.16	4.64E-01
SIGLEC1	9	-0.01	0.15	9.55E-01
SIGLEC10	7	-0.09	0.10	3.37E-01
SIGLEC5	3	-0.06	0.06	2.54E-01
SIGLEC6	21	-0.11	0.09	2.04E-01
SIGLEC7	7	0.10	0.12	4.04E-01
SIGLEC9	10	-0.04	0.05	4.13E-01

SIRPA	7	0.09	0.04	1.39E-02
SIRPB1	11	-0.02	0.07	7.60E-01
SIRT2	3	-0.56	0.27	3.57E-02
SIT1	6	-1.01	0.24	3.24E-05
SKAP1	7	-0.65	0.26	1.19E-02
SKAP2	3	-0.79	0.42	5.87E-02
SLAMF1	3	0.51	0.27	5.72E-02
SLAMF6	3	-0.41	0.22	6.74E-02
SLAMF7	7	0.05	0.06	4.63E-01
SLAMF8	5	0.03	0.05	4.85E-01
SLC16A1	4	0.25	0.27	3.51E-01
SLC27A4	2	-0.19	0.41	6.48E-01
SLC39A14	2	-0.60	0.34	7.41E-02
SLC39A5	7	0.04	0.21	8.67E-01
SLIT2	1	-0.77	0.73	2.95E-01
SLITRK2	11	0.08	0.18	6.80E-01
SLITRK6	7	0.03	0.11	7.56E-01
SMAD1	5	-0.72	0.34	3.77E-02
SMAD5	1	0.11	0.26	6.75E-01
SMOC1	8	-0.18	0.13	1.79E-01
SMOC2	5	-0.21	0.12	7.41E-02
SMPD1	14	0.16	0.08	5.22E-02
SMPDL3A	19	0.03	0.05	5.54E-01
SNAP23	2	-0.10	0.36	7.73E-01
SNAP29	5	-0.50	0.29	8.97E-02
SNCG	1	-0.02	0.05	6.85E-01
SNX9	2	-0.50	0.41	2.14E-01
SOD1	3	-0.05	0.35	8.84E-01
SOD2	8	0.23	0.20	2.54E-01
SORCS2	3	0.16	0.10	1.01E-01
SORD	3	0.76	0.28	6.58E-03
SORT1	12	-0.26	0.13	5.30E-02
SOST	12	-0.03	0.15	8.41E-01
SPARC	12	-0.05	0.16	7.55E-01
SPARCL1	4	0.03	0.06	6.35E-01
SPINK1	2	0.16	0.11	1.50E-01
SPINK4	5	0.04	0.05	4.75E-01
SPINK5	6	-0.23	0.17	1.75E-01
SPINK6	1	-0.08	0.12	5.23E-01
SPINT1	8	0.40	0.12	6.35E-04
SPINT2	11	-0.09	0.15	5.41E-01
SPOCK1	11	0.12	0.19	5.28E-01
SPON1	5	0.07	0.14	6.25E-01
SPON2	1	-0.15	0.21	4.94E-01
SPP1	7	-0.39	0.27	1.50E-01
SPRY2	6	-0.14	0.24	5.43E-01
SRC	1	-2.49	0.82	2.33E-03
SRP14	2	-1.12	0.37	2.64E-03
SRPK2	3	-0.35	0.33	2.94E-01
SSC4D	11	-0.22	0.11	3.81E-02
SSC5D	10	-0.36	0.16	2.36E-02
ST3GAL1	7	-0.19	0.14	1.70E-01
ST6GAL1	5	-0.69	0.36	5.70E-02
STAMBP	3	0.09	0.44	8.43E-01
STAT5B	3	-0.39	0.43	3.62E-01
STC1	5	0.96	0.25	1.45E-04
STC2	4	-1.49	0.40	1.83E-04
STIP1	2	-0.04	0.45	9.36E-01
STK11	4	-0.27	0.36	4.44E-01
STK24	2	-0.19	0.59	7.42E-01

STK4	4	-0.68	0.35	5.42E-02
STX16	1	-0.30	0.77	7.01E-01
STX4	2	0.92	0.54	8.69E-02
STX6	1	-0.88	0.86	3.06E-01
STX8	3	-0.20	0.38	5.99E-01
SUGT1	1	-0.13	0.69	8.52E-01
SULT1A1	1	-0.17	0.45	7.12E-01
SULT2A1	3	-0.05	0.15	7.19E-01
SUMF2	14	-0.12	0.09	1.83E-01
SUSD1	4	0.20	0.21	3.45E-01
SUSD2	3	0.04	0.10	6.42E-01
TACC3	4	0.05	0.32	8.68E-01
TACSTD2	13	-0.12	0.06	3.12E-02
TAFA5	2	-0.06	0.26	8.13E-01
TARBP2	3	-0.48	0.42	2.55E-01
TBC1D17	1	0.13	0.11	2.25E-01
TBC1D23	4	-0.29	0.25	2.36E-01
TBC1D5	2	0.82	0.54	1.29E-01
TBCB	4	-0.42	0.38	2.60E-01
TBCC	4	-0.14	0.26	6.00E-01
TBL1X	5	-0.19	0.25	4.41E-01
TCL1A	17	-0.33	0.11	3.16E-03
TCN2	1	0.18	0.09	3.48E-02
TDGF1	2	0.02	0.04	6.24E-01
TDRKH	4	-0.04	0.13	7.64E-01
TEK	9	0.02	0.07	8.02E-01
TFF1	5	-0.03	0.13	8.38E-01
TFF2	8	0.40	0.18	2.37E-02
TFF3	3	0.11	0.12	3.64E-01
TFPI	8	0.21	0.11	6.05E-02
TFPI2	7	0.04	0.19	8.54E-01
TFRC	9	0.24	0.15	1.09E-01
TGFA	4	-0.75	0.26	4.27E-03
TGFB1	7	-0.42	0.15	5.09E-03
TGFBI	8	0.02	0.07	7.78E-01
TGFBR2	7	-0.08	0.22	7.31E-01
TGFBR3	5	-0.12	0.21	5.67E-01
TGM2	2	0.11	0.25	6.55E-01
THBD	14	-0.40	0.14	5.05E-03
THBS2	7	0.22	0.09	1.91E-02
THBS4	5	0.24	0.13	5.99E-02
THOP1	1	0.23	0.18	1.98E-01
THPO	14	-0.28	0.15	5.84E-02
THY1	3	0.11	0.13	4.27E-01
TIA1	2	-0.76	0.44	8.58E-02
TIE1	11	-0.16	0.08	4.76E-02
TIGAR	2	0.63	0.53	2.34E-01
TIMD4	22	-0.03	0.09	7.36E-01
TIMP1	5	-0.40	0.24	9.33E-02
TIMP3	13	0.00	0.07	9.44E-01
TIMP4	3	0.30	0.13	1.71E-02
TINAGL1	8	-0.69	0.20	5.38E-04
TJAP1	1	-0.13	0.70	8.52E-01
TLR3	5	0.28	0.45	5.42E-01
TMPRSS15	6	0.12	0.14	4.10E-01
TMPRSS5	16	-0.11	0.06	5.38E-02
TMSB10	6	-0.54	0.27	4.47E-02
TNC	13	0.22	0.09	1.55E-02
TNF	3	-0.95	0.24	5.04E-05
TNFAIP8	1	0.38	0.56	4.95E-01

TNFRSF10A	2	-0.31	0.09	9.99E-04
TNFRSF10B	2	-0.13	0.13	3.07E-01
TNFRSF10C	15	0.08	0.05	1.26E-01
TNFRSF11A	5	0.08	0.09	3.77E-01
TNFRSF11B	8	0.02	0.18	8.92E-01
TNFRSF12A	2	-1.06	0.51	3.84E-02
TNFRSF13B	9	0.13	0.21	5.56E-01
TNFRSF13C	9	-0.39	0.22	7.89E-02
TNFRSF14	5	-0.66	0.28	1.87E-02
TNFRSF19	4	0.22	0.16	1.72E-01
TNFRSF1A	7	-0.31	0.24	2.03E-01
TNFRSF1B	6	-1.00	0.20	6.51E-07
TNFRSF21	12	-0.10	0.12	4.08E-01
TNFRSF4	6	-0.83	0.22	1.69E-04
TNFRSF6B	3	0.06	0.14	6.58E-01
TNFRSF8	7	-0.58	0.16	2.85E-04
TNFRSF9	5	-0.33	0.21	1.17E-01
TNFSF10	9	-0.04	0.09	6.82E-01
TNFSF11	7	0.07	0.14	6.16E-01
TNFSF12	16	-0.05	0.10	6.04E-01
TNFSF13	7	-0.43	0.12	5.03E-04
TNFSF13B	4	-0.15	0.27	5.74E-01
TNFSF14	5	-0.09	0.15	5.54E-01
TNR	16	0.12	0.09	1.50E-01
TNXB	6	0.13	0.10	1.82E-01
TP53	1	-0.78	0.75	2.97E-01
TP53INP1	2	0.20	0.38	6.04E-01
TPMT	2	0.02	0.09	8.01E-01
TPP1	11	-0.20	0.12	9.60E-02
TPPP3	1	-0.49	0.71	4.92E-01
TPSAB1	13	-0.08	0.05	1.36E-01
TRAF2	3	0.31	0.44	4.85E-01
TREM2	7	-0.13	0.09	1.42E-01
TREML2	7	-0.10	0.15	5.00E-01
TRIAP1	6	-0.09	0.21	6.78E-01
TRIM21	3	0.78	0.32	1.57E-02
TRIM5	1	0.12	0.15	4.32E-01
TSHB	14	0.04	0.15	7.89E-01
TSPAN1	2	-0.18	0.58	7.62E-01
TST	1	-0.20	0.45	6.47E-01
TXLNA	3	-0.25	0.34	4.68E-01
TXNDC15	10	-0.08	0.05	1.35E-01
TXNDC5	4	0.29	0.30	3.35E-01
TXNRD1	4	-0.28	0.38	4.53E-01
TYMP	2	-0.65	0.23	4.39E-03
TYRO3	8	-0.06	0.07	3.84E-01
UBAC1	1	-0.57	0.61	3.52E-01
ULBP2	6	-0.55	0.21	7.34E-03
UMOD	15	-0.09	0.06	1.08E-01
USO1	3	-0.37	0.42	3.87E-01
USP8	3	-0.45	0.39	2.39E-01
UXS1	2	0.01	0.23	9.68E-01
VAMP5	1	-1.66	0.74	2.49E-02
VASH1	4	-0.33	0.27	2.30E-01
VASN	5	-0.15	0.10	1.39E-01
VAT1	9	0.37	0.18	3.66E-02
VCAM1	10	-0.74	0.16	3.46E-06
VCAN	13	0.02	0.06	7.97E-01
VEGFA	13	0.11	0.06	4.41E-02
VEGFC	14	-0.02	0.17	9.15E-01

VEGFD	2	0.05	0.45	9.11E-01
VMO1	6	0.01	0.06	8.68E-01
VNN2	10	0.06	0.06	3.55E-01
VPS37A	2	-1.18	0.59	4.64E-02
VPS53	2	-0.47	0.57	4.02E-01
VSIG4	8	0.46	0.26	7.45E-02
VSIR	8	-0.01	0.12	9.05E-01
VSTM1	2	-0.05	0.06	4.13E-01
VSTM2L	2	-1.07	0.21	4.00E-07
VTA1	3	-0.84	0.40	3.73E-02
VTCN1	2	-1.14	0.40	4.10E-03
VWA1	5	0.50	0.30	9.83E-02
VWC2	3	-0.11	0.09	2.44E-01
VWF	6	-0.12	0.13	3.55E-01
WARS	3	-0.50	0.18	4.98E-03
WASF1	3	0.17	0.15	2.62E-01
WASF3	1	-0.07	0.33	8.34E-01
WFDC12	9	0.29	0.15	5.93E-02
WFDC2	3	-0.57	0.39	1.44E-01
WFIKKN1	12	-0.27	0.13	4.59E-02
WFIKKN2	4	0.06	0.07	3.61E-01
WIF1	13	0.07	0.13	6.02E-01
WWP2	3	0.14	0.23	5.49E-01
XCL1	9	-0.13	0.06	3.50E-02
XG	1	-1.33	0.83	1.09E-01
XPNPEP2	12	-0.26	0.16	1.10E-01
XRCC4	1	-0.85	0.69	2.15E-01
YES1	2	-0.81	0.46	8.06E-02
YTHDF3	1	-0.10	0.52	8.52E-01
ZBTB16	7	0.03	0.12	8.19E-01
ZBTB17	1	0.97	0.62	1.16E-01

Supplementary Table 17. Associations of genetically predicted proteins with mother's attained age using IVW method and cis+trans SNPs.

Protein	No. of SNPs	Beta	SE	Pval
AARSD1	3	-0.08	0.30	8.01E-01
ABHD14B	3	0.06	0.45	8.89E-01
ABL1	1	-0.02	0.54	9.73E-01
ACAA1	3	0.77	0.35	2.63E-02
ACAN	13	-0.22	0.18	2.16E-01
ACE2	7	0.14	0.23	5.41E-01
ACOX1	1	-0.25	0.62	6.88E-01
ACP5	10	0.18	0.12	1.14E-01
ACP6	2	0.02	0.05	7.13E-01
ACVRL1	3	-0.01	0.14	9.45E-01
ACY1	3	0.25	0.19	1.76E-01
ADA	4	-0.08	0.07	2.78E-01
ADA2	13	-0.27	0.13	3.86E-02
ADAM15	8	0.06	0.05	2.80E-01
ADAM22	6	0.05	0.08	5.28E-01
ADAM23	4	0.12	0.08	1.59E-01
ADAM8	7	-0.27	0.09	3.21E-03
ADAMTS13	10	0.08	0.11	4.44E-01
ADAMTS15	2	0.37	0.38	3.32E-01
ADAMTS16	1	0.32	0.38	3.96E-01
ADAMTS8	3	0.10	0.10	3.27E-01
ADGRB3	4	0.06	0.05	2.51E-01
ADGRE2	8	-0.04	0.13	7.40E-01
ADGRE5	9	0.00	0.16	9.90E-01
ADGRG1	9	0.46	0.19	1.36E-02
ADGRG2	5	0.32	0.30	2.91E-01
ADH4	6	0.25	0.24	2.89E-01
ADM	1	-2.74	0.93	3.18E-03
AFP	19	0.01	0.10	8.81E-01
AGER	12	0.22	0.16	1.60E-01
AGR2	4	0.68	0.30	2.57E-02
AGR3	2	-0.31	0.48	5.14E-01
AGRN	6	0.22	0.11	5.37E-02
AGRP	9	-0.30	0.22	1.87E-01
AGXT	10	-0.05	0.22	8.21E-01
AHCY	1	0.05	0.13	6.85E-01
AHSP	7	-0.60	0.23	1.05E-02
AIF1	1	0.37	0.40	3.61E-01
AIFM1	2	0.84	0.59	1.56E-01
AKR1B1	3	-0.53	0.28	5.64E-02
AKR1C4	1	0.01	0.85	9.94E-01
AKT1S1	3	0.56	0.41	1.72E-01
AKT3	2	0.13	0.60	8.27E-01
ALCAM	11	-0.03	0.17	8.40E-01
ALDH1A1	3	-0.18	0.34	5.98E-01
ALDH3A1	1	0.03	0.10	7.37E-01
ALPP	9	0.42	0.22	5.52E-02
AMBP	9	0.26	0.16	1.17E-01
AMIGO2	11	0.15	0.12	2.13E-01
AMN	5	0.02	0.06	6.89E-01
AMY2A	7	-0.02	0.11	8.29E-01
AMY2B	8	0.10	0.10	3.24E-01
ANG	3	0.07	0.10	4.84E-01
ANGPT1	16	-0.21	0.13	1.13E-01
ANGPT2	6	-0.31	0.18	9.80E-02
ANGPTL1	6	-0.10	0.14	4.57E-01
ANGPTL2	4	-0.45	0.29	1.15E-01

ANGPTL3	5	0.12	0.30	6.82E-01
ANGPTL4	1	-0.08	0.16	6.17E-01
ANGPTL7	6	0.01	0.11	9.45E-01
ANKRD54	1	0.94	0.66	1.58E-01
ANPEP	7	0.05	0.11	6.44E-01
ANXA11	2	0.23	0.54	6.75E-01
ANXA3	5	-0.03	0.30	9.22E-01
ANXA4	2	0.46	0.51	3.71E-01
ANXA5	1	-0.51	0.40	2.06E-01
AOC1	1	0.04	0.16	8.14E-01
AOC3	10	0.02	0.08	7.53E-01
APBB1IP	5	-0.25	0.15	9.52E-02
APEX1	3	0.13	0.11	2.49E-01
APLP1	3	0.46	0.35	1.82E-01
APOH	1	0.21	0.09	1.74E-02
APOM	8	0.45	0.13	7.09E-04
APP	14	-0.18	0.15	2.25E-01
APRT	5	0.02	0.28	9.40E-01
AREG	3	0.41	0.24	8.75E-02
ARG1	5	0.32	0.19	8.52E-02
ARHGAP1	2	-0.07	0.42	8.64E-01
ARHGAP25	2	0.05	0.41	8.96E-01
ARSA	7	0.09	0.06	1.65E-01
ARSB	10	-0.07	0.12	5.63E-01
ART3	4	-0.44	0.37	2.45E-01
ARTN	1	0.77	0.68	2.57E-01
ASAH2	15	-0.09	0.07	2.41E-01
ASGR1	6	-0.17	0.17	3.36E-01
ATF2	1	0.03	0.37	9.32E-01
ATG4A	3	-0.39	0.44	3.79E-01
ATOX1	3	-0.60	0.44	1.77E-01
ATP5IF1	5	0.28	0.29	3.41E-01
ATP6V1F	1	-0.02	0.69	9.73E-01
ATXN10	5	0.34	0.28	2.32E-01
AXIN1	3	-0.14	0.46	7.68E-01
AXL	6	-0.03	0.16	8.59E-01
AZU1	2	-0.12	0.55	8.27E-01
B4GALT1	7	-0.11	0.12	3.62E-01
B4GAT1	8	0.14	0.12	2.67E-01
BACH1	3	0.02	0.48	9.68E-01
BAG3	1	-0.30	0.72	6.72E-01
BAIAP2	5	1.00	0.32	1.86E-03
BAMBI	1	0.02	0.68	9.73E-01
BANK1	3	0.42	0.30	1.63E-01
BAX	2	0.25	0.55	6.48E-01
BCAM	13	-0.11	0.09	2.23E-01
BCAN	7	0.06	0.15	6.75E-01
BCL2L11	6	-0.26	0.18	1.41E-01
BCR	1	0.79	0.70	2.63E-01
BGN	9	-0.14	0.20	4.61E-01
BID	2	0.18	0.43	6.77E-01
BIN2	2	-0.29	0.52	5.79E-01
BIRC2	4	0.19	0.40	6.33E-01
BLMH	2	0.03	0.08	7.36E-01
BLVRB	2	-0.36	0.21	8.60E-02
BMP4	5	0.48	0.28	8.66E-02
BMP6	11	0.23	0.14	9.41E-02
BOC	8	0.11	0.19	5.63E-01
BPIFB1	13	-0.01	0.09	9.32E-01

BRK1	6	0.05	0.19	8.02E-01
BSG	9	0.25	0.19	1.79E-01
BST1	4	0.09	0.04	3.17E-02
BST2	11	-0.33	0.18	6.89E-02
BTC	3	0.05	0.11	6.62E-01
BTN2A1	6	-0.01	0.13	9.26E-01
BTN3A2	3	-0.02	0.07	7.70E-01
C19orf12	4	0.66	0.31	3.30E-02
C1QA	12	-0.02	0.10	8.57E-01
C1QTNF1	4	-0.03	0.16	8.62E-01
C2	2	0.46	0.37	2.12E-01
C2CD2L	1	-0.07	0.82	9.28E-01
C4BPB	9	-0.47	0.13	3.42E-04
CA1	4	-0.61	0.27	2.11E-02
CA11	2	-0.71	0.26	6.92E-03
CA12	6	-0.03	0.14	8.13E-01
CA13	3	-0.15	0.16	3.66E-01
CA14	4	0.39	0.22	7.63E-02
CA2	2	0.06	0.39	8.85E-01
CA3	6	-0.19	0.18	2.91E-01
CA4	8	0.34	0.11	3.01E-03
CA5A	5	-0.06	0.07	4.14E-01
CA6	11	0.03	0.06	6.89E-01
CA9	5	0.50	0.20	1.34E-02
CALB1	3	-0.05	0.29	8.53E-01
CALCA	4	1.17	0.32	2.24E-04
CALCOCO1	4	-0.03	0.30	9.26E-01
CAMKK1	3	-0.02	0.15	8.71E-01
CANT1	5	0.07	0.23	7.60E-01
CAPG	2	0.02	0.05	6.63E-01
CARHSP1	1	-0.45	0.56	4.23E-01
CASP1	1	0.00	0.17	9.91E-01
CASP10	3	-0.23	0.21	2.74E-01
CASP2	2	0.06	0.40	8.90E-01
CASP3	1	-0.50	0.63	4.27E-01
CASP8	3	0.24	0.19	2.12E-01
CBLIF	9	0.16	0.09	1.02E-01
CBLN4	5	0.14	0.19	4.63E-01
CC2D1A	2	-0.35	0.49	4.79E-01
CCDC80	5	0.20	0.20	3.03E-01
CCL11	8	0.20	0.15	2.05E-01
CCL13	12	0.14	0.12	2.23E-01
CCL14	5	0.01	0.09	8.95E-01
CCL15	4	0.87	0.21	3.90E-05
CCL16	7	0.06	0.06	2.87E-01
CCL17	12	-0.08	0.09	3.94E-01
CCL18	3	0.20	0.09	2.81E-02
CCL19	4	-0.96	0.30	1.37E-03
CCL2	8	0.24	0.14	7.36E-02
CCL20	4	-0.27	0.29	3.50E-01
CCL21	10	-0.30	0.19	1.15E-01
CCL22	6	-0.22	0.17	2.02E-01
CCL23	7	0.07	0.11	5.39E-01
CCL24	10	-0.01	0.05	8.07E-01
CCL25	6	0.12	0.06	2.96E-02
CCL26	5	-0.11	0.15	4.68E-01
CCL27	7	0.15	0.17	3.72E-01
CCL28	16	0.10	0.14	4.48E-01
CCL3	6	-1.15	0.31	1.88E-04

CCL4	5	-0.22	0.11	4.54E-02
CCL5	9	-0.09	0.16	5.47E-01
CCL7	4	-0.09	0.13	4.80E-01
CCL8	8	-0.03	0.05	5.98E-01
CCN1	11	-0.40	0.19	2.95E-02
CCN2	16	-0.06	0.11	5.86E-01
CCN3	3	0.10	0.18	5.88E-01
CCN4	11	-0.25	0.10	1.09E-02
CCN5	1	2.49	0.92	7.15E-03
CCS	1	0.00	0.09	9.60E-01
CD109	6	0.75	0.19	1.13E-04
CD14	5	0.28	0.14	4.99E-02
CD160	14	-0.12	0.09	1.82E-01
CD163	17	-0.12	0.09	2.02E-01
CD164	9	0.05	0.18	7.90E-01
CD177	3	0.07	0.09	4.17E-01
CD1C	17	-0.38	0.12	1.68E-03
CD200	7	-0.07	0.17	7.00E-01
CD200R1	9	0.05	0.06	3.49E-01
CD207	14	0.06	0.08	4.58E-01
CD209	8	-0.15	0.05	3.62E-03
CD22	18	-0.04	0.12	7.56E-01
CD244	13	0.03	0.09	7.46E-01
CD27	7	-0.43	0.15	5.57E-03
CD274	6	0.03	0.14	8.49E-01
CD276	7	0.07	0.05	1.92E-01
CD28	8	0.00	0.14	9.81E-01
CD2AP	1	-0.02	0.70	9.73E-01
CD300C	12	-0.04	0.09	6.27E-01
CD300E	10	-0.25	0.11	2.64E-02
CD300LF	9	-0.05	0.04	2.57E-01
CD300LG	10	0.19	0.11	7.58E-02
CD302	5	-0.26	0.14	5.49E-02
CD33	7	-0.05	0.04	1.92E-01
CD34	3	0.45	0.12	1.16E-04
CD38	2	0.03	0.12	7.89E-01
CD4	4	-0.14	0.12	2.52E-01
CD40	9	0.12	0.11	2.68E-01
CD40LG	12	-0.25	0.17	1.41E-01
CD46	7	0.68	0.25	6.75E-03
CD48	8	-0.05	0.06	4.26E-01
CD5	8	-0.66	0.18	1.81E-04
CD55	6	0.10	0.10	2.80E-01
CD58	11	0.23	0.09	1.06E-02
CD59	5	0.13	0.17	4.42E-01
CD6	10	-0.13	0.06	1.61E-02
CD63	8	-0.32	0.18	6.50E-02
CD69	5	-0.09	0.24	6.94E-01
CD70	3	-0.11	0.06	4.92E-02
CD74	6	-0.40	0.17	1.93E-02
CD79B	13	0.28	0.13	3.86E-02
CD83	12	-0.20	0.15	2.04E-01
CD84	11	-0.25	0.15	8.41E-02
CD8A	3	-0.19	0.16	2.40E-01
CD93	5	0.08	0.31	7.89E-01
CD99	4	-0.07	0.31	8.19E-01
CD99L2	8	-0.35	0.18	5.16E-02
CDC27	2	-0.08	0.46	8.62E-01
CDC37	2	0.77	0.52	1.41E-01

CDCP1	7	0.07	0.15	6.58E-01
CDH1	6	0.52	0.08	4.07E-10
CDH15	3	0.26	0.14	5.92E-02
CDH17	10	0.37	0.07	4.48E-07
CDH2	13	0.02	0.18	9.24E-01
CDH3	6	-0.04	0.28	8.76E-01
CDH5	11	-0.01	0.07	8.32E-01
CDH6	6	0.10	0.08	2.18E-01
CDHR1	4	-0.05	0.17	7.57E-01
CDHR2	6	1.95	0.34	9.33E-09
CDHR5	7	-0.18	0.08	2.08E-02
CDKN1A	5	0.23	0.24	3.39E-01
CDKN2D	4	-0.08	0.29	7.86E-01
CDNF	1	-0.02	0.11	8.29E-01
CDON	4	0.14	0.28	6.25E-01
CDSN	17	-0.20	0.16	2.09E-01
CEACAM1	6	-0.26	0.15	7.61E-02
CEACAM21	2	-2.10	0.82	1.07E-02
CEACAM3	1	-0.58	0.63	3.58E-01
CEACAM5	10	-0.16	0.08	6.04E-02
CEACAM8	9	-0.08	0.16	6.19E-01
CEBPB	3	0.42	0.44	3.36E-01
CELA3A	14	-0.15	0.11	1.55E-01
CEP20	4	0.13	0.27	6.43E-01
CEP43	6	0.26	0.30	3.93E-01
CEP85	2	0.33	0.33	3.24E-01
CERT	4	0.28	0.34	4.12E-01
CES1	16	0.61	0.17	4.06E-04
CES2	2	-0.11	0.32	7.40E-01
CES3	9	-0.03	0.15	8.29E-01
CETN2	3	0.66	0.36	6.40E-02
CFC1	6	0.95	0.30	1.65E-03
CGA	1	-1.02	1.13	3.67E-01
CGREF1	3	0.12	0.07	9.63E-02
CHAC2	2	0.03	0.31	9.28E-01
CHGB	5	-0.09	0.07	2.00E-01
CHI3L1	5	0.03	0.08	6.63E-01
CHIT1	3	-0.18	0.05	4.46E-04
CHL1	6	-0.02	0.13	8.94E-01
CHMP1A	4	0.34	0.34	3.10E-01
CHRDL1	3	0.36	0.46	4.40E-01
CHRDL2	2	0.94	0.41	2.38E-02
CIAPIN1	4	0.59	0.38	1.22E-01
CKAP4	4	-0.10	0.10	3.22E-01
CKMT1A_CKMT1B	6	0.54	0.24	2.83E-02
CLC	8	-0.27	0.18	1.33E-01
CLEC10A	9	0.03	0.08	7.09E-01
CLEC11A	4	0.18	0.15	2.32E-01
CLEC14A	8	0.43	0.13	1.33E-03
CLEC1A	7	-0.06	0.09	5.06E-01
CLEC1B	9	-0.02	0.17	9.13E-01
CLEC4A	11	0.28	0.08	6.04E-04
CLEC4C	25	-0.01	0.05	7.77E-01
CLEC4D	10	-0.03	0.07	7.11E-01
CLEC4G	10	-0.26	0.11	2.39E-02
CLEC5A	9	0.15	0.13	2.47E-01
CLEC6A	12	-0.24	0.08	2.05E-03
CLEC7A	3	-0.03	0.05	6.18E-01
CLIP2	2	-0.20	0.33	5.47E-01

CLMP	4	0.04	0.13	7.78E-01
CLPS	7	0.06	0.08	4.31E-01
CLSPN	1	0.07	0.44	8.81E-01
CLSTN2	3	-0.15	0.11	1.80E-01
CLTA	1	0.20	0.39	6.04E-01
CLUL1	8	0.05	0.08	5.11E-01
CNDP1	7	0.13	0.10	1.99E-01
CNPY2	1	-0.65	0.33	4.87E-02
CNPY4	3	0.52	0.24	2.77E-02
CNST	4	-0.16	0.43	7.05E-01
CNTN1	6	-0.02	0.12	8.91E-01
CNTN2	6	0.02	0.05	6.69E-01
CNTN3	15	-0.02	0.12	8.77E-01
CNTN4	8	0.07	0.13	6.26E-01
CNTN5	6	0.06	0.10	5.50E-01
CNTNAP2	4	-0.02	0.08	7.75E-01
COL18A1	4	0.32	0.23	1.71E-01
COL1A1	9	0.66	0.20	9.54E-04
COL4A1	9	0.21	0.13	8.73E-02
COL6A3	3	0.44	0.44	3.17E-01
COL9A1	4	-0.81	0.23	5.84E-04
COLEC12	4	0.10	0.27	7.14E-01
COMP	6	0.31	0.15	4.25E-02
COMT	2	-0.11	0.10	2.64E-01
CORO1A	3	1.03	0.42	1.39E-02
COX5B	5	-0.03	0.26	9.21E-01
CPA1	16	0.27	0.13	3.30E-02
CPA2	4	0.18	0.11	1.23E-01
CPB1	10	0.19	0.13	1.57E-01
CPE	6	1.23	0.25	1.35E-06
CPM	9	-0.23	0.21	2.81E-01
CPPED1	1	-0.07	0.07	3.35E-01
CPVL	8	0.17	0.06	7.03E-03
CPXM1	15	-0.07	0.10	5.03E-01
CR2	20	-0.08	0.12	4.93E-01
CRACR2A	4	0.38	0.27	1.68E-01
CRADD	4	0.68	0.38	7.16E-02
CREG1	10	0.55	0.15	2.75E-04
CRELD2	11	-0.12	0.12	3.27E-01
CRH	19	-0.16	0.10	8.89E-02
CRHBP	4	-0.12	0.08	1.11E-01
CRIM1	4	0.44	0.32	1.74E-01
CRIP2	3	0.03	0.25	9.05E-01
CRISP2	12	-0.13	0.08	1.09E-01
CRKL	1	-0.55	0.70	4.27E-01
CRLF1	5	-0.78	0.33	1.87E-02
CRNN	3	0.00	0.07	9.74E-01
CRTAC1	1	-0.02	0.07	8.15E-01
CRTAM	11	-0.12	0.12	3.26E-01
CSF1	3	-0.32	0.22	1.33E-01
CSF2RA	1	1.62	0.58	5.17E-03
CSF3	2	0.02	0.24	9.18E-01
CST3	6	0.00	0.15	9.91E-01
CST5	13	-0.09	0.06	1.06E-01
CST6	12	-0.22	0.14	1.17E-01
CST7	10	-0.14	0.06	1.71E-02
CTF1	2	0.15	0.60	8.00E-01
CTRB1	11	0.13	0.08	9.30E-02
CTRC	11	0.05	0.07	5.23E-01

CTSB	7	0.02	0.08	8.34E-01
CTSC	7	-0.03	0.07	7.24E-01
CTSD	6	0.17	0.12	1.75E-01
CTSF	14	0.23	0.11	4.20E-02
CTSH	1	-0.11	0.51	8.23E-01
CTSL	15	-0.07	0.14	6.24E-01
CTSO	9	-0.15	0.16	3.27E-01
CTSS	7	-0.24	0.25	3.22E-01
CTSV	16	0.20	0.13	1.36E-01
CTSZ	9	0.11	0.09	2.00E-01
CX3CL1	5	0.04	0.30	8.85E-01
CXADR	5	0.79	0.18	8.33E-06
CXCL1	4	0.03	0.08	6.97E-01
CXCL10	3	-0.20	0.14	1.65E-01
CXCL11	6	-0.47	0.18	8.29E-03
CXCL12	9	-0.24	0.18	1.91E-01
CXCL13	7	-1.11	0.26	2.27E-05
CXCL14	2	-1.19	0.51	1.83E-02
CXCL16	11	0.37	0.15	1.30E-02
CXCL17	5	0.15	0.32	6.49E-01
CXCL3	3	0.76	0.36	3.67E-02
CXCL5	8	0.10	0.09	3.05E-01
CXCL6	6	0.03	0.08	6.65E-01
CXCL8	6	0.15	0.20	4.69E-01
CXCL9	5	-1.04	0.22	1.73E-06
DAB2	3	0.41	0.43	3.41E-01
DAG1	3	-0.68	0.49	1.65E-01
DAPP1	2	-0.17	0.38	6.50E-01
DARS1	4	0.73	0.34	3.10E-02
DBI	3	-0.40	0.16	1.52E-02
DBNL	4	0.13	0.40	7.36E-01
DCBLD2	15	-0.11	0.12	3.72E-01
DCN	1	-0.57	0.73	4.32E-01
DCTN1	1	-0.13	0.66	8.43E-01
DCTN2	2	0.94	0.62	1.31E-01
DCTPP1	6	0.10	0.24	6.88E-01
DCXR	6	0.06	0.27	8.35E-01
DDAH1	5	-0.06	0.22	7.74E-01
DDC	6	0.31	0.11	5.81E-03
DDR1	11	0.05	0.10	6.46E-01
DDX58	1	0.38	0.17	2.38E-02
DECR1	3	0.07	0.30	8.23E-01
DEFA1_DEFA1B	13	-0.12	0.17	4.97E-01
DEFB4A_DEFB4B	3	0.58	0.31	6.49E-02
DFFA	2	0.17	0.51	7.41E-01
DIABLO	3	0.58	0.48	2.32E-01
DKK1	9	-0.13	0.15	3.83E-01
DKK3	7	0.05	0.11	6.26E-01
DKK4	5	0.00	0.14	9.87E-01
DKKL1	4	-0.03	0.04	4.24E-01
DLK1	5	-0.04	0.09	6.86E-01
DLL1	6	-0.03	0.20	8.95E-01
DNAJA2	1	1.50	0.73	3.91E-02
DNAJB1	5	0.34	0.31	2.77E-01
DNAJB8	1	-0.12	0.55	8.27E-01
DNER	6	-0.17	0.29	5.65E-01
DNMBP	3	-0.05	0.33	8.68E-01
DNPH1	1	0.05	0.15	7.64E-01
DOK2	4	0.63	0.35	7.41E-02

DPEP1	5	0.60	0.25	1.50E-02
DPEP2	3	0.24	0.15	1.05E-01
DPP10	5	0.16	0.14	2.62E-01
DPP4	5	-0.07	0.18	6.76E-01
DPP6	7	0.24	0.14	7.80E-02
DPP7	5	-0.09	0.18	6.14E-01
DPT	2	0.37	0.13	5.46E-03
DPY30	2	0.14	0.58	8.03E-01
DRAXIN	15	-0.02	0.12	8.76E-01
DRG2	3	-0.03	0.32	9.28E-01
DSC2	9	0.27	0.14	5.94E-02
DSG2	9	0.18	0.14	1.90E-01
DSG3	15	0.06	0.11	6.03E-01
DSG4	20	0.08	0.13	5.40E-01
DTX3	3	-0.15	0.23	5.20E-01
DUSP3	2	0.43	0.48	3.77E-01
EBAG9	5	-0.09	0.31	7.80E-01
EBI3_IL27	14	-0.42	0.14	1.81E-03
ECE1	5	0.43	0.21	3.95E-02
EDA2R	3	-0.47	0.51	3.55E-01
EDAR	20	0.04	0.08	6.33E-01
EDIL3	3	0.24	0.25	3.39E-01
EFEMP1	3	0.36	0.22	9.80E-02
EFNA1	4	0.03	0.13	8.34E-01
EFNA4	4	-0.86	0.27	1.60E-03
EGF	8	-0.16	0.20	4.34E-01
EGFL7	17	-0.08	0.13	5.50E-01
EGFR	5	0.43	0.31	1.68E-01
EGLN1	3	-0.07	0.26	8.05E-01
EIF4B	3	0.46	0.38	2.29E-01
EIF4G1	3	0.23	0.38	5.44E-01
ELOA	2	-0.46	0.31	1.32E-01
ENAH	1	-0.02	0.42	9.71E-01
ENG	7	0.13	0.08	1.35E-01
ENO1	4	0.21	0.25	4.07E-01
ENO2	5	-0.08	0.23	7.19E-01
ENPP2	8	0.32	0.16	4.21E-02
ENPP5	6	0.03	0.06	6.42E-01
ENPP7	6	-0.11	0.05	2.40E-02
ENTPD2	2	0.16	0.61	7.91E-01
ENTPD5	8	-0.19	0.09	3.09E-02
ENTPD6	8	-0.09	0.05	8.65E-02
EPCAM	11	0.38	0.12	1.33E-03
EPHA1	7	-0.05	0.17	7.54E-01
EPHA2	6	0.51	0.23	3.04E-02
EPHB4	9	0.27	0.12	2.07E-02
EPHB6	4	0.08	0.15	5.65E-01
EPO	9	0.54	0.17	1.34E-03
EPS8L2	3	-0.09	0.15	5.51E-01
ERBB2	8	-0.44	0.20	2.83E-02
ERBB3	8	-0.08	0.11	4.86E-01
ERBB4	14	0.10	0.14	4.73E-01
ERBIN	3	0.29	0.48	5.47E-01
EREG	8	-0.11	0.22	6.15E-01
ERP44	5	-0.10	0.12	3.95E-01
ESAM	7	0.10	0.14	4.92E-01
ESM1	10	-0.38	0.17	2.87E-02
EZR	2	0.21	0.53	6.89E-01
F11R	11	-0.29	0.22	2.00E-01

F2R	7	0.21	0.20	2.81E-01
F3	8	0.63	0.11	1.81E-09
F7	7	-0.10	0.08	2.44E-01
F9	2	-0.01	0.36	9.77E-01
FABP1	2	0.20	0.20	3.24E-01
FABP2	5	0.17	0.18	3.59E-01
FABP4	1	0.51	0.65	4.35E-01
FABP5	2	-0.64	0.38	8.99E-02
FABP6	3	-0.06	0.17	7.37E-01
FABP9	15	0.11	0.09	2.12E-01
FADD	3	0.87	0.41	3.57E-02
FAM3B	6	-0.14	0.07	5.16E-02
FAM3C	1	-1.22	0.74	9.71E-02
FAP	5	0.01	0.16	9.44E-01
FAS	3	-0.04	0.17	8.23E-01
FASLG	18	-0.27	0.10	8.35E-03
FBP1	5	-0.11	0.14	4.26E-01
FCAR	13	-0.09	0.06	1.13E-01
FCER2	10	-0.02	0.12	8.85E-01
FCGR2A	5	0.11	0.05	1.89E-02
FCGR2B	4	0.00	0.09	9.76E-01
FCGR3B	12	0.00	0.13	9.75E-01
FCN2	10	0.03	0.06	6.58E-01
FCRL1	22	-0.11	0.09	2.39E-01
FCRL2	12	-0.03	0.07	7.05E-01
FCRL3	5	-0.03	0.05	5.24E-01
FCRL5	9	-0.08	0.17	6.51E-01
FCRL6	5	0.03	0.06	5.90E-01
FCRLB	6	0.05	0.07	4.12E-01
FEN1	1	-0.11	0.84	8.99E-01
FES	1	1.09	0.33	9.52E-04
FETUB	3	-0.46	0.29	1.17E-01
FGF19	4	0.60	0.16	2.10E-04
FGF2	6	-0.09	0.06	1.83E-01
FGF21	7	-0.27	0.18	1.44E-01
FGF23	4	-0.18	0.27	4.99E-01
FGF5	2	-0.14	0.07	4.56E-02
FGFBP1	10	-0.32	0.22	1.38E-01
FGFR2	7	-0.10	0.24	6.91E-01
FGR	1	-0.22	0.48	6.50E-01
FHIT	2	0.11	0.42	7.88E-01
FIS1	4	0.59	0.24	1.45E-02
FKBP1B	4	0.13	0.18	4.82E-01
FKBP4	5	-0.51	0.32	1.09E-01
FKBP5	5	0.11	0.20	5.93E-01
FLI1	3	-0.05	0.42	9.14E-01
FLRT2	11	-0.14	0.07	5.16E-02
FLT1	9	0.24	0.11	2.32E-02
FLT3	5	0.59	0.27	2.92E-02
FLT3LG	19	0.03	0.11	8.13E-01
FLT4	10	0.10	0.06	7.73E-02
FOLR1	7	-0.18	0.21	3.88E-01
FOLR2	14	-0.41	0.13	1.45E-03
FOLR3	9	-0.50	0.22	2.50E-02
FOXO1	3	-0.10	0.28	7.23E-01
FOXO3	1	3.90	0.80	1.08E-06
FRZB	2	-0.04	0.10	6.94E-01
FST	3	0.04	0.24	8.56E-01
FSTL3	2	-0.07	0.43	8.72E-01

FUCA1	5	-0.06	0.04	1.32E-01
FURIN	3	-0.46	0.20	2.38E-02
FUS	2	-0.06	0.44	9.00E-01
FUT3_FUT5	11	0.00	0.08	9.88E-01
FUT8	11	-0.07	0.06	2.20E-01
FXN	3	-0.12	0.46	7.97E-01
FXYD5	2	0.30	0.47	5.18E-01
FYB1	2	-0.27	0.50	5.90E-01
GAL	17	0.07	0.11	5.30E-01
GALNT10	9	-0.05	0.11	6.53E-01
GALNT2	6	0.04	0.13	7.56E-01
GALNT3	8	0.30	0.10	2.79E-03
GALNT7	5	-0.05	0.14	7.25E-01
GAS6	15	0.06	0.10	5.02E-01
GBP2	1	0.04	0.43	9.26E-01
GCG	5	-0.19	0.35	5.89E-01
GCNT1	4	-0.70	0.26	6.93E-03
GDF15	1	0.11	0.16	4.88E-01
GDF2	11	-0.18	0.17	2.67E-01
GDNF	1	0.26	0.19	1.77E-01
GFAP	7	-1.73	0.23	2.20E-14
GFRA1	4	-0.31	0.16	5.17E-02
GFRA2	6	0.02	0.13	8.55E-01
GFRA3	20	0.04	0.13	7.45E-01
GGH	9	-0.09	0.10	3.67E-01
GGT1	7	0.05	0.14	7.31E-01
GGT5	4	-0.05	0.15	7.53E-01
GHRL	8	0.19	0.16	2.40E-01
GKN1	4	0.84	0.33	1.18E-02
GLB1	8	0.06	0.11	6.29E-01
GLO1	1	0.02	0.13	9.08E-01
GLOD4	2	-0.33	0.59	5.77E-01
GLRX	2	0.07	0.09	4.26E-01
GLT8D2	1	0.34	0.25	1.73E-01
GMPR	2	0.23	0.18	1.84E-01
GNE	3	-0.03	0.30	9.11E-01
GNLY	8	-0.05	0.07	5.19E-01
GOLM2	8	-0.11	0.12	3.52E-01
GOPC	2	-0.23	0.48	6.31E-01
GP1BA	14	-0.51	0.16	9.97E-04
GP2	8	-0.19	0.07	8.60E-03
GP6	6	-0.23	0.13	7.79E-02
GPA33	10	0.34	0.11	1.85E-03
GPC1	14	-0.15	0.18	4.12E-01
GPC5	10	0.05	0.06	3.38E-01
GPNMB	5	0.12	0.12	3.08E-01
GPR37	10	-0.08	0.06	1.83E-01
GRAP2	3	-0.36	0.42	3.98E-01
GRK5	1	0.00	0.17	9.83E-01
GRN	10	-0.10	0.07	1.13E-01
GRPEL1	3	0.57	0.38	1.26E-01
GSAP	1	-0.55	0.72	4.43E-01
GSTA1	9	-0.05	0.12	6.96E-01
GSTA3	5	-0.12	0.13	3.64E-01
GSTP1	1	0.15	0.21	4.80E-01
GUCA2A	7	-0.12	0.17	4.66E-01
GUSB	10	0.09	0.12	4.79E-01
GYS1	3	0.43	0.17	1.12E-02
GZMA	13	0.02	0.13	8.67E-01

GZMB	8	-0.81	0.21	1.48E-04
GZMH	7	0.04	0.17	8.17E-01
HAGH	1	-0.23	0.38	5.34E-01
HAO1	8	0.01	0.25	9.68E-01
HARS1	2	0.61	0.50	2.18E-01
HAVCR1	6	0.01	0.09	8.68E-01
HAVCR2	8	-0.19	0.15	1.81E-01
HBEGF	20	0.08	0.13	5.32E-01
HBQ1	2	-0.14	0.12	2.25E-01
HCLS1	3	-0.23	0.38	5.37E-01
HDGF	2	0.06	0.04	1.29E-01
HEBP1	1	0.24	0.36	5.13E-01
HEXIM1	4	-0.06	0.27	8.17E-01
HGF	5	-0.53	0.22	1.87E-02
HGS	4	0.37	0.39	3.40E-01
HLA-DRA	3	-0.26	0.08	1.19E-03
HLA-E	4	-0.42	0.11	2.81E-04
HMOX1	15	0.02	0.15	8.83E-01
HMOX2	4	-1.68	0.27	4.84E-10
HNMT	4	0.09	0.06	1.51E-01
HNRNPK	1	-0.59	0.75	4.27E-01
HPGDS	1	0.20	0.69	7.68E-01
HS3ST3B1	7	-0.20	0.12	9.30E-02
HS6ST1	14	0.10	0.13	4.42E-01
HSD11B1	7	-0.21	0.14	1.45E-01
HSPA1A	3	0.34	0.34	3.13E-01
HSPB1	5	0.44	0.28	1.18E-01
HSPB6	4	-0.54	0.45	2.30E-01
HSPG2	4	-0.02	0.24	9.29E-01
HTRA2	1	-0.67	0.85	4.27E-01
HYAL1	4	-0.23	0.15	1.27E-01
HYOU1	13	-0.34	0.15	2.06E-02
ICA1	2	0.07	0.21	7.25E-01
ICAM1	6	-0.13	0.10	1.90E-01
ICAM2	9	0.12	0.04	6.01E-03
ICAM3	20	-0.06	0.09	4.90E-01
ICAM4	14	0.01	0.06	9.28E-01
ICAM5	5	0.09	0.06	1.00E-01
ICOSLG	4	0.01	0.11	8.98E-01
IDI2	1	0.06	0.14	6.55E-01
IDS	3	0.01	0.20	9.73E-01
IDUA	13	0.03	0.07	7.06E-01
IFNG	2	1.08	0.58	6.15E-02
IFNGR1	10	-0.03	0.14	8.29E-01
IFNGR2	8	-0.06	0.04	2.03E-01
IFNL1	3	0.78	0.36	3.10E-02
IFNLR1	2	-1.02	0.44	2.11E-02
IGF1R	9	0.32	0.10	1.31E-03
IGF2R	14	-0.09	0.10	3.61E-01
IGFBP1	2	-0.52	0.45	2.44E-01
IGFBP2	2	-0.69	0.53	1.92E-01
IGFBP3	5	-0.11	0.09	2.32E-01
IGFBP4	3	0.01	0.48	9.78E-01
IGFBP6	3	-0.15	0.46	7.41E-01
IGFBP7	3	-0.08	0.13	5.61E-01
IGFBPL1	2	-0.07	0.11	5.03E-01
IGSF3	5	0.17	0.22	4.36E-01
IGSF8	5	0.06	0.23	7.83E-01
IKBKG	1	-0.03	0.90	9.73E-01

IKZF2	1	0.08	0.43	8.60E-01
IL10	5	-0.17	0.18	3.60E-01
IL10RA	1	-0.22	0.31	4.78E-01
IL10RB	6	0.11	0.08	1.93E-01
IL11	1	0.98	0.82	2.29E-01
IL12A_IL12B	15	-0.06	0.08	4.01E-01
IL12B	16	-0.02	0.08	7.60E-01
IL12RB1	3	0.04	0.09	6.94E-01
IL13	1	0.45	0.49	3.60E-01
IL13RA1	2	-0.47	0.48	3.20E-01
IL15	21	-0.03	0.13	8.05E-01
IL15RA	1	-0.03	0.19	8.63E-01
IL16	1	-0.97	0.68	1.55E-01
IL17C	2	0.41	0.33	2.17E-01
IL17D	2	-0.15	0.12	2.15E-01
IL17F	1	0.41	0.70	5.59E-01
IL17RA	3	0.00	0.05	9.52E-01
IL17RB	9	-0.03	0.06	5.34E-01
IL18	8	-0.32	0.11	3.95E-03
IL18BP	9	-0.27	0.18	1.44E-01
IL18R1	12	0.00	0.06	9.58E-01
IL18RAP	3	-0.12	0.29	6.78E-01
IL19	3	-0.39	0.09	2.20E-05
IL1A	1	-1.07	0.39	6.08E-03
IL1B	2	-0.04	0.17	8.37E-01
IL1R1	13	-0.14	0.10	1.75E-01
IL1R2	13	0.01	0.08	9.47E-01
IL1RAP	3	1.05	0.54	5.10E-02
IL1RL1	12	-0.03	0.06	6.13E-01
IL1RL2	12	0.04	0.09	6.73E-01
IL1RN	2	0.04	0.20	8.36E-01
IL20RA	1	0.70	0.61	2.54E-01
IL22RA1	2	0.33	0.15	2.78E-02
IL2RA	8	-0.02	0.08	7.66E-01
IL32	7	-0.06	0.15	6.90E-01
IL33	1	-1.95	0.84	2.09E-02
IL34	1	0.13	0.06	3.57E-02
IL3RA	4	0.19	0.29	5.19E-01
IL4R	5	0.02	0.12	8.86E-01
IL5RA	22	0.01	0.09	9.46E-01
IL6	4	0.30	0.18	9.00E-02
IL6R	5	0.11	0.04	5.88E-03
IL6ST	11	0.35	0.11	2.31E-03
IL7	3	-0.14	0.35	6.81E-01
IL7R	10	0.04	0.05	4.16E-01
ILKAP	1	-0.61	0.76	4.27E-01
IMPA1	2	0.09	0.22	6.75E-01
ING1	1	-0.66	0.83	4.27E-01
INHBC	6	0.00	0.08	9.60E-01
INPP1	3	0.75	0.33	2.27E-02
IPCEF1	2	-0.31	0.42	4.61E-01
IQGAP2	1	0.30	0.31	3.36E-01
IRAG2	1	1.10	0.67	1.02E-01
IRAK1	1	-0.03	0.78	9.73E-01
IRAK4	1	0.83	0.61	1.74E-01
ISLR2	7	0.26	0.08	9.13E-04
ISM1	1	0.03	0.26	9.17E-01
ITGA11	4	-0.29	0.16	7.86E-02
ITGA5	9	-0.19	0.18	3.05E-01

ITGA6	3	0.44	0.17	9.56E-03
ITGAM	10	0.12	0.15	4.32E-01
ITGAV	6	0.14	0.20	4.81E-01
ITGB1	6	0.68	0.20	4.97E-04
ITGB1BP2	4	0.01	0.32	9.87E-01
ITGB2	10	-0.07	0.15	6.39E-01
ITGB5	5	0.40	0.28	1.52E-01
ITGB6	9	0.61	0.14	9.75E-06
ITGB7	16	-0.14	0.08	7.37E-02
ITIH3	7	0.00	0.12	9.90E-01
ITM2A	1	-0.22	0.20	2.76E-01
JAM2	4	0.07	0.25	7.72E-01
JCHAIN	12	0.08	0.20	7.00E-01
KAZALD1	3	0.07	0.45	8.70E-01
KDR	11	0.11	0.08	1.78E-01
KEL	14	0.32	0.09	2.03E-04
KIFBP	3	0.44	0.30	1.45E-01
KIR2DL3	2	-1.82	0.46	7.23E-05
KIR3DL1	2	-0.04	0.05	4.23E-01
KIRREL2	7	0.02	0.11	8.56E-01
KIT	18	-0.06	0.12	6.06E-01
KITLG	15	0.17	0.11	1.41E-01
KLB	7	0.05	0.06	4.06E-01
KLK1	12	-0.19	0.05	7.83E-05
KLK10	3	-0.03	0.08	7.27E-01
KLK11	2	0.39	0.49	4.24E-01
KLK12	5	-0.06	0.04	9.55E-02
KLK13	10	0.00	0.09	9.93E-01
KLK14	9	0.01	0.11	8.87E-01
KLK4	3	0.15	0.11	1.56E-01
KLK6	2	0.02	0.23	9.32E-01
KLK8	3	-0.02	0.10	8.62E-01
KLRB1	10	-0.69	0.17	4.56E-05
KLRD1	10	-0.15	0.08	7.28E-02
KRT18	6	-0.01	0.30	9.75E-01
KRT19	3	0.44	0.27	1.04E-01
KRT5	4	-0.24	0.22	2.76E-01
KYAT1	4	-0.32	0.32	3.23E-01
KYNU	3	0.39	0.17	2.07E-02
L1CAM	6	0.28	0.18	1.13E-01
LACTB2	3	0.10	0.22	6.54E-01
LAG3	10	-0.26	0.14	6.59E-02
LAIR1	3	-2.29	0.52	1.18E-05
LAIR2	3	0.01	0.06	8.78E-01
LAMA4	11	0.07	0.14	6.17E-01
LAMP2	2	0.88	0.56	1.18E-01
LAMP3	8	-0.28	0.27	2.97E-01
LAT	5	-0.36	0.29	2.21E-01
LAT2	1	-0.02	0.71	9.73E-01
LAYN	3	-0.03	0.11	8.02E-01
LBP	2	-0.02	0.07	8.16E-01
LBR	5	0.11	0.22	6.22E-01
LCN2	16	0.12	0.15	4.26E-01
LDLR	15	-0.51	0.15	7.53E-04
LEFTY2	3	2.64	0.44	1.24E-09
LEP	1	-0.56	1.01	5.77E-01
LEPR	9	-0.19	0.14	1.80E-01
LGALS1	2	0.26	0.16	1.01E-01
LGALS3	4	-0.86	0.32	6.63E-03

LGALS4	3	0.17	0.09	5.27E-02
LGALS7_LGALS7B	6	-0.38	0.11	7.78E-04
LGALS8	6	0.19	0.09	3.98E-02
LGALS9	3	-1.73	0.32	7.04E-08
LGMN	19	0.08	0.12	5.24E-01
LHB	1	-0.91	0.51	7.37E-02
LIFR	10	0.33	0.11	1.91E-03
LILRA2	11	-0.05	0.07	4.96E-01
LILRA5	9	0.16	0.18	3.51E-01
LILRB1	7	0.13	0.07	7.58E-02
LILRB2	9	0.05	0.05	2.75E-01
LILRB4	2	-2.13	0.52	3.53E-05
LILRB5	2	-0.02	0.04	6.67E-01
LPCAT2	1	-0.68	0.40	9.16E-02
LPL	6	0.06	0.11	5.96E-01
LPO	13	-0.04	0.09	6.42E-01
LRIG1	10	-0.05	0.05	4.07E-01
LRP1	10	-0.13	0.17	4.31E-01
LRP11	6	0.06	0.07	3.83E-01
LRPAP1	7	-0.23	0.06	1.23E-04
LRRC25	12	-0.08	0.09	3.73E-01
LRRN1	6	0.07	0.06	2.10E-01
LSP1	2	-0.43	0.29	1.36E-01
LTA	8	-0.02	0.06	6.96E-01
LTA4H	1	-0.13	0.46	7.69E-01
LTBP2	8	0.34	0.22	1.22E-01
LTBP3	5	-0.18	0.09	3.98E-02
LTBR	13	-0.33	0.10	9.82E-04
LXN	1	0.70	0.26	7.54E-03
LY6D	5	0.01	0.10	9.33E-01
LY75	6	0.04	0.05	3.99E-01
LY9	11	-0.08	0.08	3.19E-01
LY96	10	-0.31	0.20	1.14E-01
LYAR	1	0.12	0.84	8.88E-01
LYN	5	-0.06	0.35	8.59E-01
LYPD3	17	-0.01	0.08	8.60E-01
LYPD8	7	0.06	0.08	4.71E-01
MAD1L1	1	-0.06	0.35	8.57E-01
MAGED1	2	0.76	0.45	9.32E-02
MANF	6	0.34	0.31	2.68E-01
MANSC1	12	0.13	0.18	4.49E-01
MAP2K6	2	1.03	0.51	4.46E-02
MAP3K5	1	-0.03	0.80	9.73E-01
MAP4K5	3	-0.17	0.23	4.58E-01
MARCO	9	-0.06	0.12	6.09E-01
MASP1	10	-0.04	0.11	7.22E-01
MATN2	5	0.20	0.10	3.84E-02
MATN3	3	0.18	0.08	2.06E-02
MAVS	3	0.20	0.38	5.98E-01
MAX	5	0.23	0.33	4.88E-01
MB	8	-0.01	0.29	9.65E-01
MCAM	8	0.11	0.15	4.76E-01
MCFD2	4	-0.34	0.24	1.47E-01
MDGA1	6	-0.01	0.04	8.53E-01
MDK	13	-0.28	0.17	9.38E-02
MED18	1	0.60	0.77	4.37E-01
MEGF10	11	0.02	0.14	8.98E-01
MEGF9	11	-0.02	0.11	8.24E-01
MEP1B	14	-0.05	0.04	1.82E-01

MEPE	11	0.37	0.18	4.06E-02
MERTK	12	0.24	0.13	5.93E-02
MESD	4	-0.11	0.35	7.63E-01
MET	17	0.23	0.11	3.56E-02
METAP1D	3	-0.85	0.42	4.52E-02
METAP2	4	-0.28	0.28	3.30E-01
MFAP5	4	0.41	0.19	3.66E-02
MFGE8	11	0.08	0.10	4.42E-01
MGLL	3	-0.01	0.39	9.85E-01
MGMT	3	0.04	0.11	6.82E-01
MIA	5	0.12	0.06	3.91E-02
MICB_MICA	8	-0.04	0.05	4.19E-01
MIF	3	-0.12	0.33	7.04E-01
MILR1	8	0.00	0.10	9.72E-01
MITD1	2	-0.40	0.49	4.16E-01
MLN	13	0.07	0.17	6.99E-01
MME	13	-0.09	0.08	2.68E-01
MMP1	10	-0.08	0.09	3.96E-01
MMP10	2	-0.10	0.14	4.44E-01
MMP12	2	-0.08	0.09	3.74E-01
MMP13	1	0.00	0.32	9.89E-01
MMP3	4	0.07	0.09	4.35E-01
MMP7	5	0.09	0.14	5.22E-01
MMP8	2	0.04	0.11	6.89E-01
MMP9	9	0.18	0.15	2.47E-01
MNDA	1	-0.24	0.90	7.91E-01
MOG	2	-0.30	0.22	1.68E-01
MPHOSPH8	2	-0.29	0.48	5.52E-01
MPI	4	-0.48	0.19	1.29E-02
MPIG6B	4	-0.08	0.29	7.98E-01
MPO	6	-0.20	0.29	4.92E-01
MSLN	7	-0.23	0.07	1.16E-03
MSMB	5	0.00	0.05	9.86E-01
MSR1	11	0.05	0.10	6.17E-01
MSRA	2	0.91	0.54	8.96E-02
MSTN	6	0.11	0.31	7.16E-01
MUC13	4	0.28	0.15	5.52E-02
MUC16	1	-0.47	0.66	4.73E-01
MVK	1	-0.20	0.53	7.01E-01
MYOC	9	0.30	0.14	2.48E-02
MZB1	7	-0.07	0.22	7.58E-01
MZT1	2	0.53	0.57	3.59E-01
NAAA	15	0.10	0.06	1.31E-01
NADK	5	-0.26	0.14	6.58E-02
NBL1	3	-0.41	0.33	2.16E-01
NBN	3	0.09	0.38	8.11E-01
NCAM1	7	0.07	0.14	6.10E-01
NCAM2	5	-0.22	0.19	2.42E-01
NCAN	9	0.20	0.18	2.82E-01
NCF2	4	-0.01	0.18	9.36E-01
NCK2	1	-1.58	0.89	7.49E-02
NCR1	22	-0.29	0.10	5.24E-03
NCS1	7	0.08	0.25	7.34E-01
NECTIN2	4	0.28	0.18	1.23E-01
NECTIN4	6	-0.23	0.14	9.19E-02
NEFL	2	-0.93	0.70	1.85E-01
NELL1	16	-0.10	0.09	2.73E-01
NELL2	13	0.19	0.18	2.79E-01
NFASC	4	0.13	0.11	2.23E-01

NFATC1	2	0.23	0.37	5.37E-01
NFATC3	1	-0.63	0.76	4.04E-01
NFKBIE	2	0.13	0.20	5.04E-01
NID1	9	-0.07	0.15	6.28E-01
NID2	12	0.08	0.07	2.59E-01
NINJ1	1	-0.56	0.64	3.81E-01
NME3	12	0.05	0.11	6.55E-01
NMNAT1	4	0.09	0.16	5.72E-01
NOMO1	3	-0.80	0.31	8.96E-03
NOS1	1	0.56	0.71	4.31E-01
NOS3	2	-0.73	0.53	1.64E-01
NOTCH1	7	0.03	0.14	8.52E-01
NOTCH3	11	0.39	0.19	3.87E-02
NPDC1	5	-0.72	0.35	4.08E-02
NPPB	2	0.88	0.24	2.01E-04
NPPC	2	1.07	0.67	1.09E-01
NPTX1	12	0.00	0.08	9.49E-01
NPTXR	3	0.01	0.08	9.48E-01
NPY	9	-0.32	0.18	7.84E-02
NRCAM	7	0.16	0.22	4.46E-01
NRP1	6	0.14	0.13	3.02E-01
NRP2	9	0.09	0.19	6.48E-01
NRTN	1	-0.50	0.81	5.38E-01
NSFL1C	2	0.33	0.44	4.51E-01
NT5C3A	4	-0.76	0.31	1.61E-02
NT5E	8	0.04	0.08	6.22E-01
NTF3	4	-0.31	0.21	1.36E-01
NTF4	1	0.87	0.86	3.08E-01
NTproBNP	4	0.55	0.16	5.19E-04
NTRK2	7	0.18	0.25	4.77E-01
NTRK3	9	0.45	0.16	5.15E-03
NUB1	4	-0.02	0.21	9.34E-01
NUCB2	5	-0.02	0.11	8.59E-01
NUDC	2	0.57	0.45	2.02E-01
NUDT2	2	-0.23	0.18	1.89E-01
NUDT5	2	-0.08	0.51	8.79E-01
OBP2B	11	0.08	0.08	3.06E-01
ODAM	2	0.05	0.17	7.85E-01
OGFR	2	-0.23	0.33	4.86E-01
OGN	4	0.17	0.08	2.57E-02
OLR1	6	0.04	0.24	8.67E-01
OMD	5	-0.24	0.23	2.98E-01
OMG	8	0.43	0.20	3.45E-02
OPTC	7	0.10	0.15	5.05E-01
OSCAR	8	-0.04	0.08	5.93E-01
OSM	8	0.02	0.17	9.13E-01
OSMR	5	0.09	0.08	2.84E-01
OXT	9	-0.08	0.08	3.17E-01
P4HB	3	-0.55	0.42	1.95E-01
PADI2	1	-0.20	0.36	5.88E-01
PADI4	1	0.15	0.17	3.86E-01
PAEP	3	-0.10	0.06	1.36E-01
PAG1	6	-1.06	0.26	4.16E-05
PAK4	1	-0.03	0.86	9.73E-01
PAM	4	-0.03	0.09	7.19E-01
PAMR1	4	0.24	0.11	3.69E-02
PAPPA	11	0.07	0.15	6.52E-01
PARK7	5	0.08	0.23	7.17E-01
PARP1	2	0.08	0.26	7.50E-01

PCDH1	5	-0.37	0.26	1.44E-01
PCDH17	8	-0.12	0.09	1.90E-01
PCOLCE	1	-0.19	0.27	4.86E-01
PCSK9	8	-0.10	0.13	4.34E-01
PDCD1	10	0.21	0.22	3.26E-01
PDCD1LG2	8	0.04	0.06	5.28E-01
PDCD5	2	-0.01	0.12	9.42E-01
PDCD6	4	0.05	0.04	2.70E-01
PDGFA	16	-0.14	0.13	2.73E-01
PDGFB	13	-0.23	0.15	1.22E-01
PDGFC	4	-0.30	0.15	4.53E-02
PDGFRA	10	0.13	0.13	3.24E-01
PDGFRB	2	0.03	0.04	4.01E-01
PDLIM7	3	0.50	0.40	2.11E-01
PEAR1	7	-0.33	0.16	3.70E-02
PEBP1	2	0.14	0.57	8.10E-01
PECAM1	7	0.29	0.09	1.29E-03
PFKFB2	4	-0.12	0.21	5.62E-01
PGF	3	-0.13	0.42	7.60E-01
PGLYRP1	11	-0.04	0.13	7.24E-01
PI3	4	-0.38	0.15	1.24E-02
PIGR	6	0.28	0.20	1.64E-01
PIK3AP1	6	0.08	0.19	6.87E-01
PIK3IP1	3	0.14	0.15	3.61E-01
PILRA	6	-0.04	0.05	4.37E-01
PILRB	4	-0.03	0.04	4.54E-01
PKLR	4	0.37	0.22	8.56E-02
PLA2G10	13	0.35	0.10	3.23E-04
PLA2G15	8	0.18	0.12	1.41E-01
PLA2G1B	17	0.11	0.14	4.48E-01
PLA2G2A	6	0.11	0.07	1.05E-01
PLA2G4A	2	0.06	0.54	9.07E-01
PLA2G7	9	-0.47	0.21	2.63E-02
PLAT	4	-1.01	0.34	2.68E-03
PLAU	8	0.18	0.13	1.50E-01
PLAUR	9	-0.07	0.16	6.61E-01
PLIN1	1	-0.80	0.54	1.37E-01
PLIN3	2	-0.24	0.51	6.41E-01
PLPBP	2	-0.27	0.50	5.87E-01
PLTP	11	0.22	0.09	1.54E-02
PLXDC1	3	-0.16	0.18	3.68E-01
PLXNA4	5	-0.23	0.24	3.38E-01
PLXNB2	11	0.04	0.06	5.08E-01
PLXNB3	5	-0.37	0.35	2.97E-01
PM20D1	6	0.59	0.27	2.95E-02
PMVK	6	-0.01	0.26	9.60E-01
PNLIPRP2	7	-0.01	0.04	7.33E-01
PNPT1	2	-0.54	0.49	2.71E-01
PODXL	6	0.28	0.08	3.97E-04
PODXL2	11	0.10	0.18	5.81E-01
POLR2F	1	-4.42	0.86	2.41E-07
PON2	3	0.04	0.06	4.65E-01
PON3	9	0.33	0.11	4.04E-03
PPCDC	2	0.01	0.13	9.45E-01
PPIB	3	-0.48	0.44	2.72E-01
PPME1	1	0.68	0.85	4.26E-01
PPP1R12A	3	0.27	0.45	5.45E-01
PPP1R2	3	-0.04	0.41	9.24E-01
PPP1R9B	2	0.65	0.53	2.24E-01

PPP3R1	4	-0.10	0.30	7.38E-01
PPY	6	0.53	0.19	4.36E-03
PRCP	12	0.08	0.12	5.34E-01
PRDX1	2	-0.48	0.49	3.25E-01
PRDX3	1	-0.63	0.71	3.70E-01
PRDX5	2	0.18	0.29	5.38E-01
PRDX6	1	-0.23	0.32	4.78E-01
PRELP	1	-0.07	0.10	4.51E-01
PRKAB1	1	-0.03	0.84	9.73E-01
PRKAR1A	4	0.62	0.35	8.14E-02
PRKRA	1	-0.01	0.44	9.73E-01
PRL	3	0.05	0.41	9.11E-01
PROC	7	-0.04	0.16	7.88E-01
PROK1	2	0.00	0.13	9.93E-01
PRSS2	11	0.17	0.16	2.84E-01
PRSS27	16	-0.23	0.06	4.67E-04
PRSS8	7	-0.03	0.22	9.05E-01
PRTFDC1	3	-0.14	0.21	5.11E-01
PRTG	8	-0.03	0.09	7.32E-01
PRTN3	12	-0.32	0.19	9.11E-02
PSG1	6	-0.18	0.28	5.27E-01
PSIP1	2	0.23	0.33	4.80E-01
PSME1	2	0.16	0.45	7.30E-01
PSME2	1	0.22	0.50	6.63E-01
PSMG3	1	-1.25	0.89	1.60E-01
PSPN	7	-0.17	0.34	6.04E-01
PSRC1	2	0.24	0.25	3.33E-01
PTGDS	2	-0.59	0.61	3.40E-01
PTH1R	1	-0.10	0.19	5.88E-01
PTN	3	0.46	0.26	8.11E-02
PTPN1	2	0.49	0.45	2.72E-01
PTPN6	2	0.76	0.48	1.09E-01
PTPRF	12	0.28	0.15	6.43E-02
PTPRM	8	0.26	0.08	7.58E-04
PTPRN2	1	0.15	0.29	6.12E-01
PTPRS	5	-0.01	0.15	9.72E-01
PTS	4	0.09	0.19	6.27E-01
PTX3	5	-0.01	0.12	9.59E-01
PVALB	6	0.00	0.06	9.96E-01
PVR	2	-0.06	0.07	3.35E-01
PXN	3	0.01	0.07	8.69E-01
QDPR	1	-0.02	0.73	9.73E-01
QPCT	11	0.03	0.12	8.29E-01
RABEPK	1	-0.19	0.30	5.35E-01
RABGAP1L	2	0.06	0.47	9.04E-01
RAD23B	3	-0.36	0.45	4.22E-01
RARRES1	1	-0.24	0.10	1.21E-02
RARRES2	3	0.40	0.21	5.96E-02
RASSF2	1	-0.38	0.48	4.24E-01
RBKS	1	0.02	0.12	8.89E-01
RBP2	4	0.03	0.31	9.29E-01
RBP5	3	-0.04	0.15	7.91E-01
RCOR1	1	-1.18	0.76	1.20E-01
REG1A	6	0.02	0.11	8.44E-01
REG1B	7	0.04	0.10	7.05E-01
REG3A	8	-0.20	0.14	1.68E-01
REG4	2	0.30	0.25	2.37E-01
RELT	4	-0.09	0.13	4.86E-01
REN	2	0.31	0.29	2.81E-01

RET	8	-0.22	0.11	4.21E-02
RETN	15	-0.08	0.12	4.69E-01
RGMA	17	0.05	0.10	6.60E-01
RGMB	4	-0.27	0.22	2.23E-01
RGS8	1	-0.09	0.26	7.21E-01
RHOC	3	0.60	0.52	2.52E-01
RILP	3	-0.25	0.39	5.25E-01
RNASE3	4	0.10	0.17	5.44E-01
RNASET2	7	0.07	0.10	5.08E-01
ROBO1	8	0.20	0.16	2.14E-01
ROBO2	5	-0.15	0.27	5.82E-01
ROR1	12	0.06	0.12	6.31E-01
RP2	2	-6.12	0.39	4.29E-56
RRM2	1	-0.82	0.75	2.71E-01
RRM2B	1	-0.02	0.49	9.73E-01
RSPO1	8	-0.01	0.15	9.64E-01
RSPO3	8	-0.57	0.20	4.27E-03
RTBDN	2	0.28	0.27	3.02E-01
RTN4R	9	-0.01	0.14	9.16E-01
RUVBL1	1	-0.08	0.20	6.85E-01
RWDD1	2	0.15	0.30	6.13E-01
S100A11	2	0.08	0.20	6.81E-01
S100A12	4	0.02	0.17	8.82E-01
S100A16	3	0.91	0.31	3.15E-03
S100A4	4	-0.24	0.42	5.62E-01
S100P	5	1.11	0.28	7.08E-05
SAMD9L	2	0.00	0.15	9.86E-01
SCAMP3	4	0.38	0.32	2.41E-01
SCARA5	6	0.01	0.14	9.24E-01
SCARB1	1	-0.25	0.84	7.68E-01
SCARB2	4	0.19	0.18	2.96E-01
SCARF1	7	-0.18	0.11	9.12E-02
SCARF2	8	0.02	0.13	8.83E-01
SCG2	4	-0.32	0.36	3.71E-01
SCG3	5	0.02	0.07	7.41E-01
SCGB1A1	15	0.01	0.11	9.01E-01
SCGB3A2	15	0.24	0.13	6.10E-02
SCGN	3	0.77	0.23	8.74E-04
SCLY	6	0.17	0.34	6.25E-01
SCP2	1	-1.65	0.84	5.12E-02
SCRN1	2	0.21	0.32	5.20E-01
SDC1	13	-0.17	0.14	2.23E-01
SDC4	20	0.07	0.11	5.43E-01
SELE	9	0.19	0.06	7.73E-04
SELP	10	0.16	0.12	1.75E-01
SELPLG	10	0.12	0.08	1.29E-01
SEMA3F	5	0.04	0.31	8.90E-01
SEMA4C	5	0.64	0.19	7.12E-04
SEMA4D	6	-0.27	0.26	2.90E-01
SEMA7A	17	-0.27	0.12	3.22E-02
SEPTIN9	2	0.29	0.41	4.86E-01
SERPINA11	8	0.13	0.07	6.91E-02
SERPINA12	5	0.05	0.07	4.24E-01
SERPINA9	3	0.11	0.10	2.71E-01
SERPINB1	3	-0.32	0.37	3.85E-01
SERPINB6	1	-0.62	0.79	4.27E-01
SERPINB8	2	0.01	0.06	8.43E-01
SERPINB9	2	-0.35	0.35	3.08E-01
SERPINE1	14	-0.30	0.14	3.44E-02

SESTD1	3	0.20	0.35	5.60E-01
SETMAR	2	-0.27	0.20	1.76E-01
SEZ6L	7	-0.13	0.21	5.46E-01
SEZ6L2	8	0.32	0.18	7.51E-02
SF3B4	4	-0.03	0.37	9.44E-01
SFRP1	5	0.15	0.20	4.66E-01
SFTPA1	1	-0.01	0.20	9.65E-01
SFTPA2	6	0.00	0.07	9.50E-01
SFTPD	10	0.04	0.05	4.58E-01
SH2B3	4	-0.39	0.35	2.71E-01
SH2D1A	2	-1.41	0.38	2.03E-04
SHMT1	4	-0.02	0.06	7.01E-01
SIAE	8	-0.05	0.17	7.79E-01
SIGLEC1	9	-0.03	0.15	8.61E-01
SIGLEC10	7	-0.15	0.10	1.27E-01
SIGLEC5	3	-0.05	0.06	3.77E-01
SIGLEC6	21	-0.26	0.09	3.45E-03
SIGLEC7	7	-0.08	0.12	5.01E-01
SIGLEC9	10	0.06	0.05	1.91E-01
SIRPA	7	0.03	0.04	3.62E-01
SIRPB1	11	-0.02	0.07	7.45E-01
SIRT2	3	0.42	0.27	1.16E-01
SIT1	6	-1.00	0.24	4.11E-05
SKAP1	7	0.15	0.26	5.57E-01
SKAP2	3	-0.56	0.42	1.84E-01
SLAMF1	3	0.50	0.27	6.37E-02
SLAMF6	3	-0.56	0.22	1.21E-02
SLAMF7	7	0.14	0.07	2.90E-02
SLAMF8	5	0.00	0.05	9.50E-01
SLC16A1	4	0.02	0.27	9.39E-01
SLC27A4	2	-0.06	0.41	8.75E-01
SLC39A14	2	-0.57	0.34	9.13E-02
SLC39A5	7	-0.31	0.21	1.43E-01
SLIT2	1	-1.36	0.74	6.51E-02
SLITRK2	11	-0.18	0.18	3.20E-01
SLITRK6	7	0.02	0.11	8.88E-01
SMAD1	5	0.08	0.35	8.23E-01
SMAD5	1	-0.14	0.26	5.92E-01
SMOC1	8	-0.15	0.13	2.51E-01
SMOC2	5	-0.03	0.12	8.13E-01
SMPD1	14	0.09	0.08	2.72E-01
SMPDL3A	19	0.11	0.05	4.11E-02
SNAP23	2	-0.14	0.36	7.08E-01
SNAP29	5	-0.24	0.30	4.26E-01
SNCG	1	0.03	0.05	4.87E-01
SNX9	2	0.22	0.41	5.87E-01
SOD1	3	-0.05	0.36	8.83E-01
SOD2	8	0.01	0.20	9.56E-01
SORCS2	3	0.05	0.10	5.95E-01
SORD	3	0.26	0.28	3.61E-01
SORT1	12	-0.25	0.14	6.48E-02
SOST	12	0.15	0.15	3.40E-01
SPARC	12	-0.24	0.16	1.36E-01
SPARCL1	4	0.01	0.06	8.87E-01
SPINK1	2	0.13	0.11	2.24E-01
SPINK4	5	-0.18	0.05	1.13E-03
SPINK5	6	-0.40	0.17	1.83E-02
SPINK6	1	0.09	0.12	4.86E-01
SPINT1	8	-0.02	0.12	8.38E-01

SPINT2	11	-0.11	0.15	4.63E-01
SPOCK1	11	-0.14	0.19	4.55E-01
SPON1	5	0.11	0.14	4.25E-01
SPON2	1	-0.35	0.22	9.94E-02
SPP1	7	0.40	0.27	1.41E-01
SPRY2	6	0.05	0.24	8.43E-01
SRC	1	-1.26	0.82	1.25E-01
SRP14	2	-0.79	0.37	3.48E-02
SRPK2	3	0.42	0.33	2.06E-01
SSC4D	11	0.00	0.11	9.91E-01
SSC5D	10	-0.03	0.16	8.53E-01
ST3GAL1	7	-0.22	0.14	1.13E-01
ST6GAL1	5	-0.37	0.36	3.10E-01
STAMBP	3	0.07	0.44	8.67E-01
STAT5B	3	0.39	0.43	3.64E-01
STC1	5	0.76	0.25	2.66E-03
STC2	4	-1.67	0.40	3.13E-05
STIP1	2	-0.33	0.45	4.71E-01
STK11	4	0.28	0.36	4.41E-01
STK24	2	-0.35	0.59	5.58E-01
STK4	4	-0.58	0.36	1.06E-01
STX16	1	-1.20	0.77	1.20E-01
STX4	2	0.98	0.54	6.93E-02
STX6	1	1.87	0.86	3.01E-02
STX8	3	-0.01	0.38	9.85E-01
SUGT1	1	-0.02	0.69	9.73E-01
SULT1A1	1	-0.77	0.45	9.03E-02
SULT2A1	3	0.10	0.15	4.92E-01
SUMF2	14	-0.18	0.09	4.65E-02
SUSD1	4	-0.22	0.21	3.08E-01
SUSD2	3	0.10	0.10	2.89E-01
TACC3	4	0.14	0.32	6.60E-01
TACSTD2	13	0.00	0.06	9.98E-01
TAFA5	2	0.25	0.26	3.46E-01
TARBP2	3	0.67	0.42	1.13E-01
TBC1D17	1	0.20	0.11	6.80E-02
TBC1D23	4	0.32	0.25	2.01E-01
TBC1D5	2	0.70	0.54	1.92E-01
TBCB	4	0.43	0.38	2.54E-01
TBCC	4	0.01	0.26	9.73E-01
TBL1X	5	-0.21	0.25	4.14E-01
TCL1A	17	-0.15	0.11	1.88E-01
TCN2	1	0.23	0.09	9.03E-03
TDGF1	2	0.02	0.04	5.41E-01
TDRKH	4	-0.06	0.13	6.68E-01
TEK	9	0.01	0.07	9.05E-01
TFF1	5	0.21	0.13	1.02E-01
TFF2	8	0.17	0.18	3.35E-01
TFF3	3	0.46	0.12	2.47E-04
TFPI	8	-0.08	0.11	4.61E-01
TFPI2	7	0.24	0.19	2.12E-01
TFRC	9	0.43	0.15	4.32E-03
TGFA	4	-0.83	0.27	1.77E-03
TGFB1	7	-0.19	0.15	2.06E-01
TGFBI	8	0.04	0.07	5.82E-01
TGFBR2	7	0.62	0.23	5.86E-03
TGFBR3	5	0.08	0.22	7.14E-01
TGM2	2	0.02	0.25	9.29E-01
THBD	14	0.09	0.14	5.06E-01

THBS2	7	0.19	0.09	3.95E-02
THBS4	5	0.12	0.13	3.47E-01
THOP1	1	-0.31	0.18	9.35E-02
THPO	14	-0.18	0.15	2.26E-01
THY1	3	0.22	0.13	1.01E-01
TIA1	2	-0.36	0.44	4.16E-01
TIE1	11	-0.04	0.08	5.87E-01
TIGAR	2	-0.08	0.53	8.77E-01
TIMD4	22	-0.05	0.09	5.75E-01
TIMP1	5	-0.37	0.24	1.25E-01
TIMP3	13	0.09	0.07	1.83E-01
TIMP4	3	-0.02	0.13	8.84E-01
TINAGL1	8	-0.53	0.20	7.52E-03
TJAP1	1	-0.02	0.70	9.73E-01
TLR3	5	0.29	0.46	5.22E-01
TMPRSS15	6	0.39	0.14	7.34E-03
TMPRSS5	16	0.03	0.06	6.28E-01
TMSB10	6	-0.21	0.27	4.34E-01
TNC	13	0.00	0.09	9.81E-01
TNF	3	-0.47	0.24	4.78E-02
TNFAIP8	1	0.79	0.56	1.58E-01
TNFRSF10A	2	-0.02	0.09	8.56E-01
TNFRSF10B	2	0.15	0.13	2.61E-01
TNFRSF10C	15	0.12	0.05	2.05E-02
TNFRSF11A	5	-0.02	0.09	7.81E-01
TNFRSF11B	8	0.19	0.18	2.79E-01
TNFRSF12A	2	-1.06	0.52	4.08E-02
TNFRSF13B	9	-0.20	0.21	3.52E-01
TNFRSF13C	9	-0.19	0.22	4.00E-01
TNFRSF14	5	0.02	0.28	9.31E-01
TNFRSF19	4	0.29	0.16	6.64E-02
TNFRSF1A	7	0.53	0.24	2.83E-02
TNFRSF1B	6	-0.43	0.20	3.23E-02
TNFRSF21	12	-0.02	0.12	8.60E-01
TNFRSF4	6	-0.87	0.22	8.51E-05
TNFRSF6B	3	-0.14	0.14	3.26E-01
TNFRSF8	7	-0.78	0.16	1.30E-06
TNFRSF9	5	-0.46	0.21	3.07E-02
TNFSF10	9	-0.02	0.09	8.34E-01
TNFSF11	7	-0.09	0.14	5.42E-01
TNFSF12	16	0.04	0.11	6.93E-01
TNFSF13	7	-0.27	0.13	2.90E-02
TNFSF13B	4	-0.09	0.28	7.32E-01
TNFSF14	5	0.00	0.15	9.84E-01
TNR	16	-0.01	0.09	8.81E-01
TNXB	6	0.22	0.10	2.90E-02
TP53	1	-0.65	0.75	3.91E-01
TP53INP1	2	-0.25	0.38	5.08E-01
TPMT	2	-0.09	0.09	3.41E-01
TPP1	11	-0.23	0.12	5.16E-02
TPPP3	1	0.59	0.71	4.09E-01
TPSAB1	13	-0.01	0.05	8.61E-01
TRAF2	3	0.05	0.44	9.12E-01
TREM2	7	0.19	0.09	2.91E-02
TREML2	7	-0.03	0.15	8.62E-01
TRIAP1	6	0.16	0.21	4.47E-01
TRIM21	3	1.16	0.33	3.77E-04
TRIM5	1	0.25	0.15	9.55E-02
TSHB	14	0.06	0.15	7.08E-01

TSPAN1	2	1.01	0.58	8.29E-02
TST	1	0.01	0.45	9.80E-01
TXLNA	3	0.22	0.34	5.11E-01
TXNDC15	10	-0.08	0.05	1.31E-01
TXNDC5	4	-0.09	0.30	7.59E-01
TXNRD1	4	-0.08	0.38	8.33E-01
TYMP	2	-0.19	0.23	4.04E-01
TYRO3	8	-0.08	0.07	2.26E-01
UBAC1	1	0.38	0.61	5.36E-01
ULBP2	6	-0.53	0.21	1.01E-02
UMOD	15	-0.13	0.06	2.19E-02
USO1	3	0.26	0.43	5.39E-01
USP8	3	0.51	0.39	1.89E-01
UXS1	2	0.21	0.23	3.73E-01
VAMP5	1	-1.67	0.74	2.48E-02
VASH1	4	-0.07	0.27	7.94E-01
VASN	5	0.11	0.10	2.62E-01
VAT1	9	-0.22	0.18	2.13E-01
VCAM1	10	-0.23	0.16	1.53E-01
VCAN	13	0.00	0.06	9.44E-01
VEGFA	13	-0.03	0.06	5.86E-01
VEGFC	14	-0.12	0.17	4.82E-01
VEGFD	2	-0.32	0.45	4.72E-01
VMO1	6	0.04	0.06	4.75E-01
VNN2	10	-0.04	0.06	5.15E-01
VPS37A	2	-0.76	0.59	2.03E-01
VPS53	2	0.83	0.57	1.43E-01
VSIG4	8	0.20	0.26	4.48E-01
VSIR	8	-0.11	0.12	3.59E-01
VSTM1	2	-0.01	0.06	8.51E-01
VSTM2L	2	-0.26	0.21	2.14E-01
VTA1	3	-0.28	0.41	4.90E-01
VTCN1	2	-1.62	0.40	4.75E-05
VWA1	5	0.61	0.30	4.36E-02
VWC2	3	-0.03	0.09	7.13E-01
VWF	6	-0.23	0.13	8.75E-02
WARS	3	-0.61	0.18	6.85E-04
WASF1	3	0.05	0.15	7.48E-01
WASF3	1	0.05	0.33	8.86E-01
WFDC12	9	-0.21	0.15	1.66E-01
WFDC2	3	-0.92	0.39	1.87E-02
WFIKKN1	12	-0.17	0.14	2.11E-01
WFIKKN2	4	0.26	0.07	1.64E-04
WIF1	13	0.18	0.13	1.57E-01
WWP2	3	0.14	0.23	5.29E-01
XCL1	9	-0.03	0.06	6.60E-01
XG	1	-0.87	0.83	2.97E-01
XPNPEP2	12	-0.09	0.16	5.89E-01
XRCC4	1	0.70	0.69	3.10E-01
YES1	2	0.04	0.47	9.38E-01
YTHDF3	1	-0.02	0.52	9.73E-01
ZBTB16	7	0.04	0.12	7.70E-01
ZBTB17	1	0.82	0.62	1.87E-01

Supplementary Table 18. Function of proteins affecting healthspan or lifespan in MR analysis.

Protein	Function
AGRP	Agouti-related protein; Plays a role in weight homeostasis. Involved in the control of feeding behavior through the central melanocortin system. Acts as alpha melanocyte-stimulating hormone antagonist by inhibiting cAMP production mediated by stimulation of melanocortin receptors within the hypothalamus and adrenal gland. Has very low activity with MC5R (By similarity). Is an inverse agonist for MC3R and MC4R being able to suppress their constitutive activity. It promotes MC3R and MC4R endocytosis in an arrestin-dependent manner; Endogenous ligands
CA11	Carbonic anhydrase-related protein 11; Does not have a catalytic activity; Carbonic anhydrases
CCN1	Cyclin-A2; Cyclin which controls both the G1/S and the G2/M transition phases of the cell cycle. Functions through the formation of specific serine/threonine protein kinase holoenzyme complexes with the cyclin-dependent protein kinases CDK1 or CDK2. The cyclin subunit confers the substrate specificity of these complexes and differentially interacts with and activates CDK1 and CDK2 throughout the cell cycle; Cyclins
CD27	Tumor necrosis factor receptor superfamily member 7; CD27 antigen; Receptor for CD70/CD27L. May play a role in survival of activated T-cells. May play a role in apoptosis through association with SIVA1
CD74	HLA class II histocompatibility antigen gamma chain; Plays a critical role in MHC class II antigen processing by stabilizing peptide-free class II alpha/beta heterodimers in a complex soon after their synthesis and directing transport of the complex from the endoplasmic reticulum to the endosomal/lysosomal system where the antigen processing and binding of antigenic peptides to MHC class II takes place. Serves as cell surface receptor for the cytokine MIF; CD molecules
CDH1	Cadherin-1; Cadherins are calcium-dependent cell adhesion proteins. They preferentially interact with themselves in a homophilic manner in connecting cells; cadherins may thus contribute to the sorting of heterogeneous cell types. CDH1 is involved in mechanisms regulating cell-cell adhesions, mobility and proliferation of epithelial cells. Has a potent invasive suppressor role. It is a ligand for integrin alpha-E/beta-7
CDH17	Cadherin-17; Cadherins are calcium-dependent cell adhesion proteins. They preferentially interact with themselves in a homophilic manner in connecting cells; cadherins may thus contribute to the sorting of heterogeneous cell types. LI-cadherin may have a role in the morphological organization of liver and intestine. Involved in intestinal peptide transport
CDHR2	Cadherin-related family member 2; Intermicrovillar adhesion molecule that forms, via its extracellular domain, calcium-dependent heterophilic complexes with CDHR5 on adjacent microvilli. Thereby, controls the packing of microvilli at the apical membrane of epithelial cells. Through its cytoplasmic domain, interacts with microvillus cytoplasmic proteins to form the intermicrovillar adhesion complex/IMAC. This complex plays a central role in microvilli and epithelial brush border differentiation. May also play a role in cell-cell adhesion and contact inhibition in epithelial cells; Cadhe [...]
CEACAM21	Carcinoembryonic antigen-related cell adhesion molecule 21; Carcinoembryonic antigen related cell adhesion molecule family; Belongs to the immunoglobulin superfamily. CEA family
CPE	Carboxypeptidase E; Removes residual C-terminal Arg or Lys remaining after initial endoprotease cleavage during prohormone processing. Processes proinsulin; Belongs to the peptidase M14 family
CXADR	Coxsackievirus and adenovirus receptor; Component of the epithelial apical junction complex that may function as a homophilic cell adhesion molecule and is essential for tight junction integrity. Also involved in transepithelial migration of leukocytes through adhesive interactions with JAML a transmembrane protein of the plasma membrane of leukocytes. The interaction between both receptors also mediates the activation of gamma-delta T-cells, a subpopulation of T-cells residing in epithelia and involved in tissue homeostasis and repair. Upon epithelial CXADR-binding, JAML induces downs [...]
CXCL13	C-X-C motif chemokine 13; Chemotactic for B-lymphocytes but not for T-lymphocytes, monocytes and neutrophils. Does not induce calcium release in B- lymphocytes. Binds to BLR1/CXCR5; Chemokine ligands

CXCL9	C-X-C motif chemokine 9; Cytokine that affects the growth, movement, or activation state of cells that participate in immune and inflammatory response. Chemotactic for activated T-cells. Binds to CXCR3; Belongs to the intercrine alpha (chemokine CxC) family
F3	Coagulation factor iii (tissue factor); Tissue factor; Initiates blood coagulation by forming a complex with circulating factor VII or VIIa. The [TF:VIIa] complex activates factors IX or X by specific limited protolysis. TF plays a role in normal hemostasis by initiating the cell-surface assembly and propagation of the coagulation protease cascade
FASLG	Tumor necrosis factor ligand superfamily member 6; Cytokine that binds to TNFRSF6/FAS, a receptor that transduces the apoptotic signal into cells. Involved in cytotoxic T-cell-mediated apoptosis, natural killer cell-mediated apoptosis and in T-cell development. Initiates fratricidal/suicidal activation-induced cell death (AICD) in antigen-activated T-cells contributing to the termination of immune responses (By similarity). TNFRSF6/FAS-mediated apoptosis has also a role in the induction of peripheral tolerance (By similarity). Binds to TNFRSF6B/DcR3, a decoy receptor that blocks apopto [...]
FES	Tyrosine-protein kinase Fes/Fps; Tyrosine-protein kinase that acts downstream of cell surface receptors and plays a role in the regulation of the actin cytoskeleton, microtubule assembly, cell attachment and cell spreading. Plays a role in FCER1 (high affinity immunoglobulin epsilon receptor)-mediated signaling in mast cells. Acts down- stream of the activated FCER1 receptor and the mast/stem cell growth factor receptor KIT. Plays a role in the regulation of mast cell degranulation. Plays a role in the regulation of cell differentiation and promotes neurite outgrowth in response to NGF [...]
FOXO3	Forkhead box protein O3; Transcriptional activator which triggers apoptosis in the absence of survival factors, including neuronal cell death upon oxidative stress. Recognizes and binds to the DNA sequence 5'-[AG]TAAA[TC]A-3'. Participates in post-transcriptional regulation of MYC: following phosphorylation by MAPKAPK5, promotes induction of miR-34b and miR-34c expression, 2 post- transcriptional regulators of MYC that bind to the 3'UTR of MYC transcript and prevent its translation; Forkhead boxes
FURIN	Furin, paired basic amino acid cleaving enzyme; Furin; Furin is likely to represent the ubiquitous endoprotease activity within constitutive secretory pathways and capable of cleavage at the RX(K/R)R consensus motif; Proprotein convertase subtilisin/kexin family
GCNT1	Beta-1,3-galactosyl-O-glycosyl-glycoprotein beta-1,6-N-acetylglucosaminyltransferase; Glycosyltransferase that catalyzes the transfer of an N- acetylglucosamine moiety onto mucin-type core 1 O-glycan to form the branched mucin-type core 2 O-glycan. Mucin-type core 2 O- glycans play an important role in leukocyte extravasation as they serve as scaffolds for the display of the selectin ligand sialyl Lewis X by leukocytes; Glucosaminyl transferases/xylosyltransferases
GFAP	Glial fibrillary acidic protein; GFAP, a class-III intermediate filament, is a cell- specific marker that, during the development of the central nervous system, distinguishes astrocytes from other glial cells
GP1BA	Platelet glycoprotein Ib alpha chain; GP-Ib, a surface membrane protein of platelets, participates in the formation of platelet plugs by binding to the A1 domain of vWF, which is already bound to the subendothelium; CD molecules
GPNMB	Transmembrane glycoprotein NMB; Could be a melanogenic enzyme; Belongs to the PMEL/NMB family
GRN	Granulin precursor; Granulins; Granulins have possible cytokine-like activity. They may play a role in inflammation, wound repair, and tissue remodeling
GZMB	Granzyme B; This enzyme is necessary for target cell lysis in cell- mediated immune responses. It cleaves after Asp. Seems to be linked to an activation cascade of caspases (aspartate-specific cysteine proteases) responsible for apoptosis execution. Cleaves caspase-3, -7, -9 and 10 to give rise to active enzymes mediating apoptosis; Belongs to the peptidase S1 family. Granzyme subfamily
HLA-DRA	Major histocompatibility complex, class ii, dr alpha; HLA class II histocompatibility antigen, DR alpha chain; Binds peptides derived from antigens that access the endocytic route of antigen presenting cells (APC) and presents them on the cell surface for recognition by the CD4 T-cells. The peptide binding cleft accommodates peptides of 10-30 residues. The peptides presented by MHC class II molecules are generated mostly by degradation of proteins that access the endocytic route, where they are processed by lysosomal proteases and other hydrolases. Exogenous antigens that have been end [...]

IGFBP1	Insulin-like growth factor-binding protein 1; IGF-binding proteins prolong the half-life of the IGFs and have been shown to either inhibit or stimulate the growth promoting effects of the IGFs on cell culture. They alter the interaction of IGFs with their cell surface receptors. Promotes cell migration
IL19	Interleukin-19; May play some important roles in inflammatory responses. Up-regulates IL-6 and TNF-alpha and induces apoptosis (By similarity); Interleukins
ITGB6	Integrin subunit beta 6; Integrin beta-6; Integrin alpha-V/beta-6 is a receptor for fibronectin and cytotactin. It recognizes the sequence R-G-D in its ligands. Internalisation of integrin alpha-V/beta-6 via clathrin-mediated endocytosis promotes carcinoma cell invasion. ITGAV:ITGB6 acts as a receptor for fibrillin-1 (FBN1) and mediates R-G-D-dependent cell adhesion to FBN1
KIR2DL3	Killer cell immunoglobulin-like receptor 2DL3; Receptor on natural killer (NK) cells for HLA-C alleles (HLA-Cw1, HLA-Cw3 and HLA-Cw7). Inhibits the activity of NK cells thus preventing cell lysis; CD molecules
LAIR1	Leukocyte-associated immunoglobulin-like receptor 1; Functions as an inhibitory receptor that plays a constitutive negative regulatory role on cytolytic function of natural killer (NK) cells, B-cells and T-cells. Activation by Tyr phosphorylation results in recruitment and activation of the phosphatases PTPN6 and PTPN11. It also reduces the increase of intracellular calcium evoked by B-cell receptor ligation. May also play its inhibitory role independently of SH2-containing phosphatases. Modulates cytokine production in CD4+ T-cells, down- regulating IL2 and IFNG production while induc [...]
LDLR	Low-density lipoprotein receptor; Binds LDL, the major cholesterol-carrying lipoprotein of plasma, and transports it into cells by endocytosis. In order to be internalized, the receptor-ligand complexes must first cluster into clathrin-coated pits; Belongs to the LDLR family
LEFTY2	Left-right determination factor 2; Required for left-right (L-R) asymmetry determination of organ systems in mammals. May play a role in endometrial bleeding
LGALS9	Galectin-9; Binds galactosides. Has high affinity for the Forssman pentasaccharide. Ligand for HAVCR2/TIM3. Binding to HAVCR2 induces T-helper type 1 lymphocyte (Th1) death. Also stimulates bactericidal activity in infected macrophages by causing macrophage activation and IL1B secretion which restricts intracellular bacterial growth (By similarity). Ligand for P4HB; the interaction retains P4HB at the cell surface of Th2 T-helper cells, increasing disulfide reductase activity at the plasma membrane, altering the plasma membrane redox state and enhancing cell migration. Ligand for CD44; [...]
LILRB4	Leukocyte immunoglobulin-like receptor subfamily B member 4; Receptor for class I MHC antigens. Recognizes a broad spectrum of HLA-A, HLA-B, HLA-C and HLA-G alleles. Involved in the down-regulation of the immune response and the development of tolerance, e.g. towards transplants. Interferes with TNFRSF5- signaling and NF-kappa-B up-regulation. Inhibits receptor-mediated phosphorylation of cellular proteins and mobilization of intracellular calcium ions; CD molecules
PAG1	Phosphoprotein associated with glycosphingolipid-enriched microdomains 1; Negatively regulates TCR (T-cell antigen receptor)- mediated signaling in T-cells and FCER1 (high affinity immunoglobulin epsilon receptor)-mediated signaling in mast cells. Promotes CSK activation and recruitment to lipid rafts, which results in LCK inhibition. Inhibits immunological synapse formation by preventing dynamic arrangement of lipid raft proteins. May be involved in cell adhesion signaling
PCSK9	Proprotein convertase subtilisin/kexin type 9; Crucial player in the regulation of plasma cholesterol homeostasis. Binds to low-density lipid receptor family members: low density lipoprotein receptor (LDLR), very low density lipoprotein receptor (VLDLR), apolipoprotein E receptor (LRP1/APOER) and apolipoprotein receptor 2 (LRP8/APOER2), and promotes their degradation in intracellular acidic compartments. Acts via a non-proteolytic mechanism to enhance the degradation of the hepatic LDLR through a clathrin LDLRAP1/ARH-mediated pathway. May prevent the recycling of LDLR from endosomes to [...]
PLA2G7	Platelet-activating factor acetylhydrolase; Modulates the action of platelet-activating factor (PAF) by hydrolyzing the sn-2 ester bond to yield the biologically inactive lyso-PAF. Has a specificity for substrates with a short residue at the sn-2 position. It is inactive against long-chain phospholipids; Phospholipases

POLR2F	DNA-directed RNA polymerases I, II, and III subunit RPABC2; DNA-dependent RNA polymerases catalyze the transcription of DNA into RNA using the four ribonucleoside triphosphates as substrates. Common component of RNA polymerases I, II, and III which synthesize ribosomal RNA precursors, mRNA precursors and many functional non-coding RNAs, and small RNAs, such as 5S rRNA and tRNAs, respectively. Pol II is the central component of the basal RNA polymerase II transcription machinery. Pols are composed of mobile elements that move relative to each other. In Pol II, POLR2F/RPB6 is part of the [...]
RP2	Protein XRP2; Acts as a GTPase-activating protein (GAP) involved in trafficking between the Golgi and the ciliary membrane. Involved in localization of proteins, such as NPHP3, to the cilium membrane by inducing hydrolysis of GTP ARL3, leading to the release of UNC119 (or UNC119B). Acts as a GTPase-activating protein (GAP) for tubulin in concert with tubulin-specific chaperone C, but does not enhance tubulin heterodimerization. Acts as guanine nucleotide dissociation inhibitor towards ADP-ribosylation factor-like proteins; Belongs to the TBCC family
SIT1	Signaling threshold-regulating transmembrane adapter 1; Negatively regulates TCR (T-cell antigen receptor)- mediated signaling in T-cells. Involved in positive selection of T-cells
STC2	Stanniocalcin-2; Has an anti-hypocalcemic action on calcium and phosphate homeostasis; Belongs to the stanniocalcin family
TNFRSF8	Tumor necrosis factor receptor superfamily member 8; Receptor for TNFSF8/CD30L. May play a role in the regulation of cellular growth and transformation of activated lymphoblasts. Regulates gene expression through activation of NF- kappa-B
VCAM1	Vascular cell adhesion protein 1; Important in cell-cell recognition. Appears to function in leukocyte-endothelial cell adhesion. Interacts with integrin alpha-4/beta-1 (ITGA4/ITGB1) on leukocytes, and mediates both adhesion and signal transduction. The VCAM1/ITGA4/ITGB1 interaction may play a pathophysiologic role both in immune responses and in leukocyte emigration to sites of inflammation; C2-set domain containing
VSTM2L	V-set and transmembrane domain-containing protein 2-like protein; V-set and transmembrane domain containing 2 like
