# Supplementary material for: Using genetics and proteomics data to identify proteins causally related to COVID-19, healthspan and lifespan: a Mendelian randomization study
Source: Aging (Albany NY). 2024 Apr 3;16(7):6384–416. doi: 10.18632/aging.205711 (PMC11042960; doi:10.18632/aging.205711)
Supplement: Supplementary Tables 6-14 and 19-33 [file aging-16-205711-s003.pdf]

SUPPLEMENTARY TABLES

Supplementary Table 6. Sensitivity analysis of genetically predicted proteins with severe COVID-19 using the weighted mode and weighted median methods.

Protein	No. of SNPs	BETA (Weighted Mode)	SE (Weighted Mode)	*P*-val (Weighted Mode)	BETA (Weighted Median)	SE (Weighted Median)	*P*-val (Weighted Median)
ADGRG2	6	0.15	0.15	3.36E-01	0.18	0.16	2.60E-01
AMY2B	9	−0.08	0.04	3.09E-02	−0.09	0.04	2.36E-02
CCL15	4	0.47	0.08	1.23E-09	0.39	0.08	1.85E-07
CD109	6	−0.49	0.08	5.78E-10	−0.48	0.08	1.31E-10
CD209	8	0.11	0.02	3.56E-11	0.11	0.02	1.28E-11
CD34	3	−0.24	0.04	5.26E-10	−0.23	0.04	2.46E-09
CDH15	4	0.31	0.05	6.57E-11	0.30	0.05	1.62E-10
CKMT1A_CKMT1B	6	0.10	0.20	6.16E-01	0.25	0.14	8.03E-02
CX3CL1	7	0.02	0.49	9.61E-01	−0.55	0.17	1.45E-03
ERBB4	16	−0.14	0.08	7.33E-02	−0.15	0.07	3.54E-02
FGF19	5	0.37	0.06	5.58E-09	0.31	0.06	8.35E-07
GOLM2	9	0.29	0.07	1.88E-05	0.17	0.08	3.01E-02
ICAM5	6	−0.09	0.02	3.58E-05	−0.09	0.02	7.01E-05
ISLR2	8	−0.16	0.03	4.74E-10	−0.17	0.03	9.15E-11
KEL	16	−0.13	0.04	4.93E-04	−0.08	0.05	9.28E-02
KLK1	13	−0.10	0.02	2.16E-09	−0.10	0.02	4.65E-10
LAMP3	10	−0.09	0.09	2.94E-01	−0.19	0.10	5.00E-02
LEFTY2	4	0.04	0.35	9.07E-01	−0.57	0.31	6.21E-02
LGALS4	3	−0.19	0.03	3.31E-11	−0.19	0.03	2.59E-11
LGALS8	6	−0.22	0.04	3.48E-07	−0.20	0.04	3.83E-06
MNDA	1	NA	NA	NA	NA	NA	NA
MUC13	4	−0.03	0.10	7.50E-01	−0.18	0.09	3.98E-02
NRCAM	6	−0.11	0.17	5.02E-01	−0.25	0.15	8.32E-02
PECAM1	8	−0.19	0.03	6.47E-10	−0.19	0.03	5.32E-10
PODXL	6	−0.20	0.03	1.59E-09	−0.19	0.03	1.68E-08
PTPRM	8	−0.16	0.03	1.18E-09	−0.16	0.03	8.58E-10
REG1A	5	0.13	0.06	1.95E-02	0.17	0.06	5.56E-03
REG1B	7	0.09	0.05	7.77E-02	0.15	0.06	1.69E-02
SCG2	4	−0.16	0.25	5.22E-01	−0.85	0.19	1.17E-05
SCGN	5	0.66	0.27	1.55E-02	0.23	0.13	7.21E-02
SEMA4C	4	−0.43	0.07	6.90E-10	−0.43	0.07	1.95E-10
SFTPD	11	−0.08	0.02	1.04E-05	−0.09	0.02	7.16E-07
TDGF1	1	NA	NA	NA	NA	NA	NA
VAMP5	2	NA	NA	NA	NA	NA	NA
VTCN1	2	NA	NA	NA	NA	NA	NA

The results for proteins with number of SNPs less than 3 are filled with NA values.

Supplementary Table 7. Sensitivity analysis of genetically predicted proteins with COVID-19 hospitalization using the weighted mode and weighted median methods.

Protein	No. of SNPs	BETA (Weighted Mode)	SE (Weighted Mode)	*P*-val (Weighted Mode)	BETA (Weighted Median)	SE (Weighted Median)	*P*-val (Weighted Median)
ADGRG1	9	−0.04	0.08	6.66E-01	−0.13	0.07	7.43E-02
ADGRG2	6	0.11	0.09	2.52E-01	0.17	0.13	1.80E-01
AMY2A	8	−0.04	0.03	2.65E-01	−0.05	0.04	2.11E-01
AMY2B	9	−0.04	0.02	1.31E-01	−0.05	0.03	8.59E-02
CA4	9	0.04	0.03	1.67E-01	0.05	0.03	1.16E-01
CCL15	4	0.28	0.05	9.09E-08	0.26	0.05	2.61E-07
CD109	6	−0.42	0.05	1.28E-14	−0.40	0.06	2.91E-12
CD209	8	0.11	0.01	4.03E-21	0.11	0.01	5.57E-20
CD34	3	−0.22	0.03	4.84E-16	−0.19	0.03	1.23E-13
CDH15	4	0.17	0.03	1.06E-07	0.17	0.03	8.76E-08
CLEC14A	8	−0.27	0.04	4.35E-14	−0.22	0.04	2.22E-07
CTSS	7	−0.06	0.08	4.95E-01	−0.09	0.08	2.66E-01
CX3CL1	7	−0.02	0.22	9.46E-01	−0.26	0.12	3.27E-02
EFNA1	4	0.13	0.03	1.84E-05	0.13	0.03	1.37E-05
ERBB4	16	−0.03	0.05	5.63E-01	−0.06	0.05	2.04E-01
FGF19	5	0.24	0.04	3.28E-08	0.23	0.04	2.98E-08
GOLM2	9	0.11	0.08	2.12E-01	0.12	0.05	1.54E-02
GP2	8	0.16	0.02	2.67E-15	0.15	0.02	6.80E-10
ICAM2	10	−0.05	0.01	1.51E-06	−0.02	0.03	4.19E-01
ICAM5	7	−0.04	0.02	1.12E-02	−0.07	0.02	5.37E-05
ISLR2	8	−0.14	0.02	1.38E-15	−0.13	0.02	3.87E-13
KLK1	13	−0.06	0.01	8.04E-08	−0.06	0.01	5.47E-08
LEFTY2	4	−0.10	0.15	4.79E-01	−0.33	0.19	8.40E-02
LGALS4	3	−0.19	0.02	3.97E-22	−0.18	0.02	8.61E-22
MET	20	−0.26	0.04	8.78E-10	−0.21	0.05	1.49E-05
NELL2	14	−0.44	0.07	2.36E-09	−0.28	0.08	7.18E-04
NRCAM	7	0.00	0.08	9.67E-01	−0.04	0.09	6.38E-01
PECAM1	8	−0.16	0.02	1.35E-15	−0.15	0.02	1.37E-13
PODXL	6	−0.17	0.02	3.06E-14	−0.14	0.02	1.63E-09
PRSS27	18	−0.08	0.02	2.14E-07	−0.07	0.02	1.08E-05
PTPRM	8	−0.14	0.02	5.37E-16	−0.14	0.02	3.28E-16
REG1A	5	0.12	0.04	7.72E-04	0.17	0.04	2.68E-05
REG1B	7	0.09	0.03	1.15E-02	0.08	0.04	3.96E-02
SCG2	4	0.01	0.15	9.33E-01	−0.48	0.12	1.20E-04
SCGN	5	0.09	0.16	5.68E-01	0.13	0.07	7.15E-02
SELE	9	−0.10	0.01	3.55E-16	−0.10	0.01	5.32E-15
SEMA3F	5	−0.12	0.10	2.31E-01	−0.15	0.10	1.36E-01
SEMA4C	5	−0.36	0.05	1.03E-14	−0.34	0.05	1.18E-13
SFTPA2	6	0.07	0.02	1.61E-05	0.07	0.02	1.75E-05
SFTPD	11	−0.07	0.01	6.42E-09	−0.07	0.01	1.70E-08
TDGF1	1	NA	NA	NA	NA	NA	NA
TGFBR2	8	−0.57	0.08	8.99E-12	−0.30	0.11	5.09E-03
VAMP5	2	NA	NA	NA	NA	NA	NA

The results for proteins with number of SNPs less than 3 are filled with NA values.

Supplementary Table 8. Sensitivity analysis of genetically predicted proteins with COVID-19 infection using the weighted mode and weighted median methods.

Protein	No. of SNPs	BETA (Weighted Mode)	SE (Weighted Mode)	*P*-val (Weighted Mode)	BETA (Weighted Median)	SE (Weighted Median)	*P*-val (Weighted Median)
ADAM15	8	0.04	0.01	1.50E-09	0.04	0.01	1.58E-09
ADGRG1	10	0.02	0.03	5.94E-01	−0.01	0.03	7.22E-01
ADGRG2	6	0.04	0.04	3.23E-01	0.07	0.05	1.30E-01
AMY2A	8	0.00	0.01	8.32E-01	−0.02	0.02	3.48E-01
AMY2B	9	−0.01	0.01	3.75E-01	−0.03	0.01	4.90E-02
BST2	11	−0.01	0.03	8.44E-01	0.00	0.03	9.41E-01
CA4	11	0.00	0.01	8.09E-01	0.00	0.01	7.70E-01
CCL15	4	0.19	0.03	1.32E-12	0.16	0.03	6.19E-09
CCL28	18	0.00	0.02	9.62E-01	−0.01	0.02	5.38E-01
CD109	6	−0.36	0.03	1.20E-37	−0.29	0.04	1.84E-12
CD209	8	0.10	0.01	5.45E-54	0.10	0.01	1.10E-48
CD34	3	−0.18	0.01	3.99E-42	−0.16	0.01	2.50E-38
CD58	12	−0.19	0.01	1.21E-40	−0.13	0.03	5.94E-06
CDH17	12	0.09	0.01	1.19E-14	0.02	0.03	5.69E-01
CKMT1A_CKMT1B	8	0.01	0.03	7.50E-01	0.02	0.03	5.08E-01
CLEC14A	9	−0.24	0.02	1.05E-37	−0.21	0.02	2.65E-18
CTSS	7	−0.06	0.04	8.33E-02	−0.07	0.04	7.34E-02
CX3CL1	8	−0.05	0.06	3.36E-01	−0.16	0.07	3.30E-02
DPP10	5	−0.01	0.02	4.96E-01	−0.02	0.02	2.59E-01
DRAXIN	17	−0.05	0.02	6.29E-03	−0.05	0.02	5.51E-03
EFNA1	4	0.10	0.01	8.92E-12	0.10	0.01	1.81E-11
F2R	8	−0.03	0.03	3.60E-01	−0.03	0.03	1.87E-01
FCGR2B	4	−0.01	0.01	4.36E-01	−0.01	0.02	5.55E-01
FGF19	5	0.16	0.02	4.81E-14	0.15	0.02	6.08E-15
FGFR2	8	0.00	0.04	9.14E-01	0.01	0.03	7.81E-01
FLT4	12	0.00	0.01	7.98E-01	0.00	0.01	5.96E-01
FOLR1	8	−0.06	0.05	2.88E-01	−0.13	0.05	5.44E-03
GKN1	4	0.10	0.11	3.50E-01	0.27	0.07	5.46E-05
GOLM2	8	0.03	0.02	1.24E-01	0.06	0.02	1.71E-02
ICAM2	10	−0.01	0.01	3.96E-01	0.00	0.02	9.41E-01
ICAM5	7	−0.01	0.01	1.37E-01	−0.01	0.01	1.77E-01
IDS	3	−0.01	0.02	7.23E-01	−0.02	0.02	3.63E-01
ISLR2	8	−0.13	0.01	6.41E-39	−0.11	0.01	2.62E-26
ITGA6	7	−0.03	0.02	1.43E-01	−0.03	0.02	8.71E-02
ITGB1	7	0.01	0.03	7.85E-01	0.00	0.03	9.52E-01
KEL	17	−0.01	0.01	6.63E-01	−0.01	0.01	4.39E-01
KLK1	13	−0.04	0.01	1.32E-14	−0.04	0.01	8.85E-14
LEFTY2	3	−0.02	0.06	7.72E-01	−0.24	0.09	5.42E-03
MNDA	1	NA	NA	NA	NA	NA	NA
MUC13	4	−0.03	0.02	2.23E-01	−0.15	0.05	1.88E-03
NELL2	15	0.04	0.06	4.54E-01	−0.20	0.05	3.72E-05
NME3	14	−0.02	0.02	2.30E-01	−0.02	0.02	1.39E-01
NRCAM	7	0.00	0.03	9.40E-01	−0.04	0.04	4.03E-01
PECAM1	8	−0.14	0.01	1.53E-43	−0.14	0.01	9.26E-37
PLAT	4	−0.05	0.05	3.13E-01	−0.11	0.05	4.46E-02
PODXL	6	−0.15	0.01	5.01E-29	−0.11	0.01	1.81E-16

PTPRM	8	−0.12	0.01	1.26E-44	−0.12	0.01	7.06E-46
REG1A	6	0.02	0.02	1.76E-01	0.04	0.02	1.13E-01
REG1B	7	0.03	0.02	3.16E-02	0.02	0.02	4.75E-01
S100A16	3	0.02	0.04	6.24E-01	0.03	0.05	5.49E-01
SCARF2	8	−0.03	0.02	6.28E-02	−0.03	0.02	3.68E-02
SCG2	4	−0.13	0.06	2.44E-02	−0.33	0.06	1.93E-08
SEMA3F	6	−0.01	0.06	8.04E-01	−0.03	0.05	5.54E-01
SEMA4C	6	−0.32	0.02	3.69E-38	−0.26	0.03	1.49E-21
SFTPD	12	−0.03	0.01	1.27E-06	−0.03	0.01	1.88E-05
SLITRK2	11	0.18	0.04	3.55E-06	0.09	0.03	4.29E-03
SPINK5	7	−0.02	0.01	2.06E-01	−0.02	0.02	2.14E-01
STC1	6	0.04	0.03	2.45E-01	0.01	0.04	6.90E-01
TDGF1	2	NA	NA	NA	NA	NA	NA
TGFBR2	8	−0.04	0.07	5.73E-01	−0.19	0.07	8.11E-03
ULBP2	8	0.01	0.01	5.83E-01	0.01	0.01	5.51E-01
VAMP5	2	NA	NA	NA	NA	NA	NA
VTCN1	2	NA	NA	NA	NA	NA	NA

The results for proteins with number of SNPs less than 3 are filled with NA values.

Supplementary Table 9. Sensitivity analysis of genetically predicted proteins with severe COVID-19 using MR-SPI.

Protein	No. of SNPs	No. of valid SNPs	BETA (MR-SPI)	SE (MR-SPI)	*P*-val (MR-SPI)
ADGRG2	6	5	0.25	0.13	5.34E-02
AMY2B	9	7	−0.06	0.04	7.58E-02
CCL15	4	4	0.34	0.07	1.74E-06
CD109	6	6	−0.35	0.08	4.11E-06
CD209	8	8	0.11	0.02	1.47E-10
CD34	3	3	−0.21	0.04	3.12E-07
CDH15	4	4	0.28	0.05	2.19E-09
CKMT1A_CKMT1B	6	5	0.10	0.11	3.74E-01
CX3CL1	7	6	−0.46	0.11	3.84E-05
ERBB4	16	15	−0.21	0.06	3.10E-04
FGF19	5	5	0.30	0.05	2.53E-08
GOLM2	9	8	0.22	0.05	4.70E-06
ICAM5	6	6	−0.09	0.02	1.31E-05
ISLR2	8	8	−0.13	0.03	5.72E-07
KEL	16	15	−0.10	0.03	3.83E-04
KLK1	13	11	−0.10	0.02	2.04E-10
LAMP3	10	6	−0.11	0.08	1.64E-01
LEFTY2	4	4	−0.16	0.21	4.40E-01
LGALS4	3	3	−0.19	0.03	5.43E-11
LGALS8	6	6	−0.22	0.03	5.08E-10
MUC13	4	4	−0.35	0.07	1.25E-07
NRCAM	6	5	−0.12	0.12	2.99E-01
PECAM1	8	8	−0.16	0.03	5.00E-08
PODXL	6	6	−0.13	0.03	2.34E-06
PTPRM	8	7	−0.15	0.03	5.94E-09
REG1A	5	5	0.53	0.06	2.30E-17
REG1B	7	7	0.09	0.04	2.55E-02
SCG2	4	4	−0.06	0.18	7.59E-01

SCGN	5	5	0.11	0.10	2.42E-01
SEMA4C	4	4	−0.37	0.07	7.94E-08
SFTPD	11	8	−0.09	0.02	5.49E-07

The results for proteins with number of SNPs less than 3 are omitted.

Supplementary Table 10. Sensitivity analysis of genetically predicted proteins with COVID-19 hospitalization using MR-SPI.

Protein	No. of SNPs	No. of valid SNPs	BETA (MR-SPI)	SE (MR-SPI)	*P*-val (MR-SPI)
ADGRG1	9	8	−0.04	0.05	4.80E-01
ADGRG2	6	4	0.16	0.08	5.14E-02
AMY2A	8	6	−0.03	0.03	2.51E-01
AMY2B	9	7	−0.03	0.02	1.45E-01
CA4	9	8	0.03	0.03	3.23E-01
CCL15	4	4	0.24	0.05	6.20E-07
CD109	6	6	−0.32	0.05	5.44E-11
CD209	8	8	0.10	0.01	6.80E-19
CD34	3	3	−0.17	0.03	3.61E-11
CDH15	4	4	0.16	0.03	1.98E-07
CLEC14A	8	8	0.09	0.06	1.83E-01
CTSS	7	6	−0.07	0.06	2.56E-01
CX3CL1	7	5	0.00	0.09	9.63E-01
EFNA1	4	4	0.13	0.03	4.10E-06
ERBB4	16	14	−0.04	0.03	2.56E-01
FGF19	5	5	0.18	0.04	2.13E-07
GOLM2	9	7	0.06	0.04	9.16E-02
GP2	8	7	0.17	0.02	1.91E-21
ICAM2	10	9	−0.10	0.01	1.85E-12
ICAM5	7	7	−0.20	0.03	3.39E-13
ISLR2	8	7	0.02	0.04	6.94E-01
KLK1	13	13	−0.05	0.01	1.20E-06
LEFTY2	4	4	−0.15	0.12	2.11E-01
LGALS4	3	3	−0.18	0.02	6.56E-21
MET	20	18	0.01	0.03	6.78E-01
NELL2	14	11	−0.03	0.06	6.35E-01
NRCAM	7	6	−0.03	0.07	6.39E-01
PECAM1	8	8	−0.14	0.02	1.26E-12
PODXL	6	6	−0.12	0.02	9.56E-11
PRSS27	18	17	−0.08	0.02	9.75E-08
PTPRM	8	7	−0.13	0.02	9.40E-15
REG1A	5	4	0.14	0.03	3.03E-07
REG1B	7	5	0.06	0.03	4.02E-02
SCG2	4	4	0.15	0.12	2.32E-01
SCGN	5	5	0.20	0.05	4.09E-05
SELE	9	8	−0.09	0.01	3.79E-14
SEMA3F	5	4	−0.07	0.08	3.57E-01
SEMA4C	5	5	−0.25	0.04	2.42E-08
SFTPA2	6	6	0.07	0.02	1.83E-05
SFTPD	11	9	−0.07	0.01	3.26E-09
TGFBR2	8	7	−0.05	0.07	4.95E-01

The results for proteins with number of SNPs less than 3 are omitted.

Supplementary Table 11. Sensitivity analysis of genetically predicted proteins with COVID-19 infection using MR-SPI.

Protein	No. of SNPs	No. of valid SNPs	BETA (MR-SPI)	SE (MR-SPI)	*P*-val (MR-SPI)
ADAM15	8	7	0.03	0.01	1.57E-09
ADGRG1	10	9	−0.02	0.02	3.00E-01
ADGRG2	6	4	0.04	0.04	2.76E-01
AMY2A	8	6	0.00	0.01	9.70E-01
AMY2B	9	7	0.00	0.01	8.96E-01
BST2	11	9	−0.02	0.02	3.13E-01
CA4	11	10	0.00	0.01	7.67E-01
CCL15	4	3	−0.10	0.05	4.49E-02
CCL28	18	17	−0.01	0.02	3.55E-01
CD109	6	5	−0.01	0.04	8.30E-01
CD209	8	8	0.11	0.01	8.13E-76
CD34	3	2	0.02	0.04	5.63E-01
CD58	12	11	−0.02	0.01	2.66E-01
CDH17	12	11	0.08	0.01	4.59E-15
CKMT1A_CKMT1B	8	6	0.02	0.03	5.17E-01
CLEC14A	9	7	−0.01	0.03	6.32E-01
CTSS	7	6	−0.11	0.03	1.73E-04
CX3CL1	8	6	0.03	0.04	3.79E-01
DPP10	5	4	−0.01	0.02	5.21E-01
DRAXIN	17	16	−0.04	0.01	2.21E-03
EFNA1	4	4	0.09	0.01	1.01E-10
F2R	8	7	−0.02	0.02	3.01E-01
FCGR2B	4	3	−0.01	0.01	6.02E-01
FGF19	5	5	0.13	0.02	1.22E-15
FGFR2	8	7	0.01	0.03	7.29E-01
FLT4	12	11	0.00	0.01	6.78E-01
FOLR1	8	5	−0.05	0.03	1.18E-01
GKN1	4	4	0.47	0.06	6.15E-16
GOLM2	8	6	0.02	0.02	2.22E-01
ICAM2	10	8	0.00	0.01	6.79E-01
ICAM5	7	6	−0.01	0.01	8.60E-02
IDS	3	2	−0.01	0.02	5.18E-01
ISLR2	8	6	−0.02	0.02	2.58E-01
ITGA6	7	6	−0.03	0.02	5.95E-02
ITGB1	7	6	0.00	0.02	8.30E-01
KEL	17	14	0.00	0.01	6.26E-01
KLK1	13	12	0.01	0.02	5.11E-01
LEFTY2	3	2	−0.03	0.06	6.71E-01
MUC13	4	3	−0.02	0.02	2.47E-01
NELL2	15	13	−0.03	0.02	1.85E-01
NME3	14	12	−0.01	0.01	5.66E-01
NRCAM	7	6	−0.04	0.03	1.44E-01
PECAM1	8	8	−0.01	0.02	8.32E-01
PLAT	4	4	−0.24	0.05	1.62E-06
PODXL	6	5	−0.02	0.01	1.59E-01
PTPRM	8	7	−0.11	0.01	4.97E-43
REG1A	6	4	0.03	0.01	4.30E-02
REG1B	7	5	0.01	0.01	2.62E-01

S100A16	3	2	0.02	0.04	6.09E-01
SCARF2	8	7	−0.03	0.01	3.07E-02
SCG2	4	2	−0.10	0.06	8.04E-02
SEMA3F	6	5	−0.01	0.04	8.91E-01
SEMA4C	6	5	−0.02	0.04	6.01E-01
SFTPD	12	10	−0.03	0.01	5.31E-06
SLITRK2	11	11	0.09	0.02	1.23E-05
SPINK5	7	6	−0.02	0.01	1.34E-01
STC1	6	5	0.03	0.03	3.91E-01
TGFBR2	8	7	−0.02	0.03	4.77E-01
ULBP2	8	7	0.00	0.01	8.05E-01

The results for proteins with number of SNPs less than 3 are omitted.

Supplementary Table 12. Sensitivity analysis of genetically predicted proteins with severe COVID-19 using the cis-SNPs only.

Protein	BETA (cis SNPs)	SE (cis SNPs)	*P*-val (cis SNPs)
ADGRG2	NA	NA	NA
AMY2B	−0.08	0.04	2.32E-02
CCL15	NA	NA	NA
CD109	NA	NA	NA
CD209	0.08	0.04	6.45E-02
CD34	NA	NA	NA
CDH15	0.31	0.05	4.55E-11
CKMT1A_CKMT1B	NA	NA	NA
CX3CL1	NA	NA	NA
ERBB4	−0.15	0.08	7.59E-02
FGF19	NA	NA	NA
GOLM2	NA	NA	NA
ICAM5	−0.09	0.02	1.80E-05
ISLR2	−0.08	0.07	2.71E-01
KEL	−0.04	0.06	4.55E-01
KLK1	−0.10	0.02	2.42E-10
LAMP3	−0.09	0.10	3.63E-01
LEFTY2	NA	NA	NA
LGALS4	−0.31	0.22	1.66E-01
LGALS8	−0.01	0.05	8.01E-01
MNDA	NA	NA	NA
MUC13	−0.02	0.07	7.64E-01
NRCAM	NA	NA	NA
PECAM1	NA	NA	NA
PODXL	−0.01	0.06	8.97E-01
PTPRM	−0.37	0.22	8.93E-02
REG1A	0.10	0.06	8.88E-02
REG1B	0.08	0.04	8.88E-02
SCG2	NA	NA	NA
SCGN	0.18	0.17	2.85E-01
SEMA4C	NA	NA	NA
SFTPD	−0.09	0.02	8.14E-07
TDGF1	NA	NA	NA

VAMP5	0.16	0.20	4.24E-01
VTCN1	−0.02	0.28	9.56E-01

The results for proteins without cis-SNPs are filled with NA values.

Supplementary Table 13. Sensitivity analysis of genetically predicted proteins with COVID-19 hospitalization using the cis-SNPs only.

Protein	BETA (cis SNPs)	SE (cis SNPs)	*P*-val (cis SNPs)
ADGRG1	−0.31	0.15	3.57E-02
ADGRG2	NA	NA	NA
AMY2A	−0.04	0.03	1.95E-01
AMY2B	−0.04	0.02	8.00E-02
CA4	0.04	0.03	1.68E-01
CCL15	NA	NA	NA
CD109	NA	NA	NA
CD209	0.05	0.03	1.33E-01
CD34	NA	NA	NA
CDH15	0.17	0.03	4.43E-08
CLEC14A	NA	NA	NA
CTSS	NA	NA	NA
CX3CL1	NA	NA	NA
EFNA1	0.13	0.03	1.55E-05
ERBB4	−0.05	0.06	3.52E-01
FGF19	NA	NA	NA
GOLM2	NA	NA	NA
GP2	0.00	0.04	9.30E-01
ICAM2	NA	NA	NA
ICAM5	−0.04	0.01	2.52E-03
ISLR2	−0.05	0.05	2.78E-01
KLK1	−0.06	0.01	2.65E-08
LEFTY2	NA	NA	NA
LGALS4	−0.05	0.12	6.60E-01
MET	−0.02	0.08	8.05E-01
NELL2	NA	NA	NA
NRCAM	NA	NA	NA
PECAM1	NA	NA	NA
PODXL	−0.05	0.04	2.45E-01
PRSS27	−0.03	0.06	6.77E-01
PTPRM	−0.25	0.15	1.09E-01
REG1A	0.08	0.04	5.68E-02
REG1B	0.06	0.03	5.68E-02
SCG2	NA	NA	NA
SCGN	0.11	0.12	3.38E-01
SELE	−0.03	0.12	7.85E-01
SEMA3F	NA	NA	NA
SEMA4C	NA	NA	NA
SFTPA2	0.07	0.02	1.30E-05
SFTPD	−0.07	0.01	1.40E-07
TDGF1	NA	NA	NA

TGFBR2	NA	NA	NA
VAMP5	0.14	0.14	3.37E-01

The results for proteins without cis-SNPs are filled with NA values.

Supplementary Table 14. Sensitivity analysis of genetically predicted proteins with COVID-19 infection using the cis-SNPs only.

Protein	BETA (cis SNPs)	SE (cis SNPs)	*P*-val (cis SNPs)
ADAM15	0.03	0.01	4.44E-08
ADGRG1	−0.03	0.07	6.93E-01
ADGRG2	NA	NA	NA
AMY2A	−0.01	0.01	6.83E-01
AMY2B	−0.01	0.01	3.94E-01
BST2	0.00	0.03	9.13E-01
CA4	0.00	0.01	8.81E-01
CCL15	NA	NA	NA
CCL28	NA	NA	NA
CD109	NA	NA	NA
CD209	0.02	0.01	8.28E-02
CD34	NA	NA	NA
CD58	−0.02	0.03	4.40E-01
CDH17	NA	NA	NA
CKMT1A_CKMT1B	0.02	0.05	6.82E-01
CLEC14A	NA	NA	NA
CTSS	NA	NA	NA
CX3CL1	NA	NA	NA
DPP10	−0.02	0.02	3.90E-01
DRAXIN	−0.05	0.02	9.96E-03
EFNA1	0.10	0.01	6.46E-12
F2R	−0.01	0.04	8.88E-01
FCGR2B	−0.01	0.01	4.67E-01
FGF19	NA	NA	NA
FGFR2	0.01	0.04	7.20E-01
FLT4	−0.02	0.02	2.91E-01
FOLR1	−0.12	0.08	1.27E-01
GKN1	NA	NA	NA
GOLM2	NA	NA	NA
ICAM2	NA	NA	NA
ICAM5	−0.01	0.01	1.39E-01
IDS	NA	NA	NA
ISLR2	−0.04	0.02	1.05E-01
ITGA6	−0.03	0.02	1.29E-01
ITGB1	NA	NA	NA
KEL	0.00	0.02	8.64E-01
KLK1	−0.04	0.01	1.21E-14
LEFTY2	NA	NA	NA
MNDA	NA	NA	NA
MUC13	−0.03	0.02	1.11E-01
NELL2	NA	NA	NA
NME3	−0.03	0.03	3.89E-01
NRCAM	NA	NA	NA

PECAM1	NA	NA	NA
PLAT	−0.03	0.06	6.39E-01
PODXL	−0.04	0.02	3.86E-02
PTPRM	−0.03	0.07	6.20E-01
REG1A	0.01	0.02	4.95E-01
REG1B	0.01	0.01	4.95E-01
S100A16	0.02	0.05	7.45E-01
SCARF2	−0.03	0.02	4.90E-02
SCG2	NA	NA	NA
SEMA3F	NA	NA	NA
SEMA4C	NA	NA	NA
SFTPD	−0.03	0.01	5.36E-07
SLITRK2	NA	NA	NA
SPINK5	−0.02	0.01	1.93E-01
STC1	−0.07	0.08	3.52E-01
TDGF1	NA	NA	NA
TGFBR2	NA	NA	NA
ULBP2	0.01	0.01	6.55E-01
VAMP5	0.15	0.08	5.59E-02
VTCN1	−0.11	0.06	9.55E-02

The results for proteins without cis-SNPs are filled with NA values.

Supplementary Table 19. Sensitivity analysis of genetically predicted proteins with healthspan using the weighted mode and weighted median methods.

Protein	No. of SNPs	BETA (Weighted Mode)	SE (Weighted Mode)	*P*-val (Weighted Mode)	BETA (Weighted Median)	SE (Weighted Median)	*P*-val (Weighted Median)
FOXO3	1	NA	NA	NA	NA	NA	NA
GPNMB	6	0.43	0.14	2.59E-03	0.44	0.14	1.96E-03
HLA-DRA	3	−0.41	0.09	4.89E-06	−0.42	0.09	3.67E-06
PLA2G7	8	−0.30	0.67	6.51E-01	−1.26	0.40	1.80E-03

The results for proteins with number of SNPs less than 3 are filled with NA values.

Supplementary Table 20. Sensitivity analysis of genetically predicted proteins with father's attained age using the weighted mode and weighted median methods.

Protein	No. of SNPs	BETA (Weighted Mode)	SE (Weighted Mode)	*P*-val (Weighted Mode)	BETA (Weighted Median)	SE (Weighted Median)	*P*-val (Weighted Median)
AGRP	9	−0.74	0.41	7.16E-02	−0.83	0.35	1.83E-02
CA11	2	NA	NA	NA	NA	NA	NA
CCN1	11	−0.34	0.38	3.59E-01	−0.40	0.31	2.01E-01
CD27	7	−0.46	0.18	1.09E-02	−0.54	0.18	1.94E-03
CD74	6	−0.29	0.23	1.98E-01	−0.32	0.23	1.54E-01
CDH1	6	0.34	0.09	7.10E-05	0.35	0.09	6.90E-05
CEACAM21	2	NA	NA	NA	NA	NA	NA
CPE	6	1.32	0.42	1.54E-03	1.35	0.37	2.83E-04
CXCL13	7	−0.40	0.36	2.73E-01	−0.75	0.40	6.28E-02
CXCL9	5	−0.46	0.32	1.52E-01	−1.09	0.36	2.13E-03
F3	8	0.47	0.12	1.01E-04	0.49	0.12	8.17E-05

FASLG	18	−0.20	0.15	1.87E-01	−0.25	0.15	7.99E-02
FES	1	NA	NA	NA	NA	NA	NA
FURIN	3	−1.32	0.24	7.34E-08	−1.03	0.22	3.57E-06
GCNT1	4	−2.15	0.33	1.27E-10	−1.81	0.39	2.73E-06
GP1BA	14	−0.03	0.30	9.16E-01	−0.12	0.24	6.10E-01
GRN	10	−0.41	0.07	2.41E-08	−0.38	0.07	2.35E-07
GZMB	8	−0.56	0.29	5.12E-02	−0.58	0.27	3.36E-02
IGFBP1	2	NA	NA	NA	NA	NA	NA
KIR2DL3	2	NA	NA	NA	NA	NA	NA
LAIR1	3	−0.72	0.71	3.13E-01	−1.63	0.82	4.61E-02
LDLR	15	−1.00	0.26	9.71E-05	−0.96	0.22	1.46E-05
LEFTY2	3	1.74	0.58	2.96E-03	2.21	0.73	2.39E-03
LGALS9	3	−1.43	1.03	1.67E-01	−1.77	0.56	1.66E-03
LILRB4	2	NA	NA	NA	NA	NA	NA
PAG1	6	−0.56	0.43	1.95E-01	−0.63	0.40	1.19E-01
PCSK9	8	−0.48	0.14	6.45E-04	−0.49	0.14	4.77E-04
POLR2F	1	NA	NA	NA	NA	NA	NA
RP2	2	NA	NA	NA	NA	NA	NA
SIT1	6	−0.30	0.64	6.39E-01	−0.75	0.40	5.84E-02
VCAM1	10	−0.42	0.82	6.08E-01	−0.76	0.30	1.22E-02
VSTM2L	2	NA	NA	NA	NA	NA	NA

The results for proteins with number of SNPs less than 3 are filled with NA values.

Supplementary Table 21. Sensitivity analysis of genetically predicted proteins with mother's attained age using the weighted mode and weighted median methods.

Protein	No. of SNPs	BETA (Weighted Mode)	SE (Weighted Mode)	*P*-val (Weighted Mode)	BETA (Weighted Median)	SE (Weighted Median)	*P*-val (Weighted Median)
CDH1	6	0.50	0.09	1.35E-08	0.50	0.09	2.03E-08
CDH17	10	0.36	0.08	1.05E-05	0.31	0.10	2.07E-03
CDHR2	6	−0.17	0.49	7.36E-01	0.05	0.48	9.22E-01
CPE	6	0.54	0.42	1.98E-01	0.67	0.38	8.17E-02
CXADR	5	1.06	0.23	4.07E-06	1.10	0.23	1.41E-06
CXCL9	5	−0.32	0.45	4.75E-01	−0.90	0.36	1.20E-02
F3	8	0.69	0.12	1.71E-08	0.71	0.13	2.46E-08
FOXO3	1	NA	NA	NA	NA	NA	NA
GFAP	7	−0.06	0.38	8.85E-01	−0.21	0.32	5.16E-01
IL19	3	−0.41	0.11	1.11E-04	−0.40	0.10	3.29E-05
ITGB6	9	0.52	0.16	1.47E-03	0.53	0.16	6.94E-04
LAIR1	3	−1.21	1.06	2.53E-01	−2.01	0.88	2.32E-02
LEFTY2	3	2.09	0.77	6.45E-03	2.54	0.57	1.03E-05
LGALS9	3	−2.64	0.51	1.99E-07	−2.46	0.49	4.59E-07
LILRB4	2	NA	NA	NA	NA	NA	NA
POLR2F	1	NA	NA	NA	NA	NA	NA
RP2	2	NA	NA	NA	NA	NA	NA
STC2	4	−0.92	0.65	1.55E-01	−1.13	0.56	4.34E-02
TNFRSF8	7	−0.50	0.22	2.03E-02	−0.56	0.22	9.55E-03

The results for proteins with number of SNPs less than 3 are filled with NA values.

Supplementary Table 22. Sensitivity analysis of genetically predicted proteins with healthspan using MR-SPI.

Protein	No. of SNPs	No. of valid SNPs	BETA (MR-SPI)	SE (MR-SPI)	*P*-val (MR-SPI)
GPNMB	6	5	0.31	0.13	2.22E-02
HLA-DRA	3	3	−0.42	0.09	3.16E-06
PLA2G7	8	8	−1.20	0.27	8.80E-06

The results for proteins with number of SNPs less than 3 are omitted.

Supplementary Table 23. Sensitivity analysis of genetically predicted proteins with father's attained age using MR-SPI.

Protein	No. of SNPs	No. of valid SNPs	BETA (MR-SPI)	SE (MR-SPI)	*P*-val (MR-SPI)
AGRP	9	7	−0.93	0.26	2.94E-04
CCN1	11	8	−0.49	0.22	2.49E-02
CD27	7	6	−0.49	0.17	2.92E-03
CD74	6	5	−0.18	0.19	3.51E-01
CDH1	6	6	0.37	0.08	8.32E-06
CPE	6	5	0.91	0.28	1.33E-03
CXCL13	7	6	−0.94	0.28	8.23E-04
CXCL9	5	4	−0.67	0.26	9.59E-03
F3	8	8	0.43	0.11	3.54E-05
FASLG	18	17	−0.17	0.11	1.31E-01
FURIN	3	3	−1.05	0.21	3.14E-07
GCNT1	4	4	−2.19	0.32	5.14E-12
GP1BA	14	13	−0.24	0.17	1.63E-01
GRN	10	10	−0.36	0.07	3.06E-08
GZMB	8	7	−0.56	0.22	1.02E-02
LAIR1	3	2	−0.71	0.64	2.69E-01
LDLR	15	13	−1.21	0.16	2.68E-14
LEFTY2	3	3	4.15	0.65	1.64E-10
LGALS9	3	3	−2.82	0.42	2.88E-11
PAG1	6	5	−0.45	0.29	1.16E-01
PCSK9	8	7	−0.54	0.13	3.34E-05
SIT1	6	5	−0.10	0.29	7.17E-01
VCAM1	10	8	−0.05	0.19	8.14E-01

The results for proteins with number of SNPs less than 3 are omitted.

Supplementary Table 24. Sensitivity analysis of genetically predicted proteins with mother's attained age using MR-SPI.

Protein	No. of SNPs	No. of valid SNPs	BETA (MR-SPI)	SE (MR-SPI)	*P*-val (MR-SPI)
CDH1	6	6	0.53	0.08	3.84E-10
CDH17	10	10	0.37	0.07	3.96E-07
CDHR2	6	5	−0.05	0.36	8.89E-01
CPE	6	6	1.23	0.26	2.27E-06
CXADR	5	5	0.79	0.18	9.95E-06
CXCL9	5	5	−1.65	0.29	8.56E-09
F3	8	8	0.64	0.11	1.60E-09
GFAP	7	6	−0.23	0.25	3.43E-01

IL19	3	3	−0.39	0.09	2.37E-05
ITGB6	9	8	0.51	0.14	2.86E-04
LAIR1	3	3	−2.28	0.56	4.09E-05
LEFTY2	3	3	2.65	0.46	7.68E-09
LGALS9	3	2	−2.65	0.42	2.34E-10
STC2	4	4	−1.66	0.42	6.53E-05
TNFRSF8	7	7	−0.33	0.18	6.78E-02

The results for proteins with number of SNPs less than 3 are omitted.

Supplementary Table 25. Sensitivity analysis of genetically predicted proteins with healthspan using the cis-SNPs only.

Protein	BETA (cis SNPs)	SE (cis SNPs)	*P*-val (cis SNPs)
FOXO3	NA	NA	NA
GPNMB	0.47	0.15	1.28E-03
HLA-DRA	−0.40	0.09	9.04E-06
PLA2G7	NA	NA	NA

The results for proteins without cis-SNPs are filled with NA values.

Supplementary Table 26. Sensitivity analysis of genetically predicted proteins with father's attained age using the cis-SNPs only.

Protein	BETA (cis SNPs)	SE (cis SNPs)	*P*-val (cis SNPs)
AGRP	−0.07	0.60	9.01E-01
CA11	NA	NA	NA
CCN1	−0.16	0.42	7.08E-01
CD27	−0.52	0.18	3.53E-03
CD74	−0.23	0.81	7.80E-01
CDH1	NA	NA	NA
CEACAM21	NA	NA	NA
CPE	NA	NA	NA
CXCL13	−0.80	0.86	3.53E-01
CXCL9	−0.50	0.35	1.55E-01
F3	0.28	0.24	2.59E-01
FASLG	−0.99	0.60	9.87E-02
FES	1.78	0.33	4.80E-08
FURIN	−1.32	0.24	2.69E-08
GCNT1	NA	NA	NA
GP1BA	NA	NA	NA
GRN	−0.19	0.19	3.19E-01
GZMB	NA	NA	NA
IGFBP1	NA	NA	NA
KIR2DL3	NA	NA	NA
LAIR1	NA	NA	NA
LDLR	NA	NA	NA
LEFTY2	NA	NA	NA
LGALS9	NA	NA	NA
LILRB4	NA	NA	NA
PAG1	−0.64	0.61	2.91E-01
PCSK9	−0.44	0.14	2.25E-03

POLR2F	NA	NA	NA
RP2	NA	NA	NA
SIT1	NA	NA	NA
VCAM1	NA	NA	NA
VSTM2L	−0.04	0.39	9.23E-01

The results for proteins without cis-SNPs are filled with NA values.

Supplementary Table 27. Sensitivity analysis of genetically predicted proteins with mother's attained age using the cis-SNPs only.

Protein	BETA (cis SNPs)	SE (cis SNPs)	*P*-val (cis SNPs)
CDH1	NA	NA	NA
CDH17	NA	NA	NA
CDHR2	NA	NA	NA
CPE	NA	NA	NA
CXADR	0.85	0.38	2.54E-02
CXCL9	−0.07	0.35	8.32E-01
F3	0.30	0.24	2.22E-01
FOXO3	NA	NA	NA
GFAP	−0.16	0.54	7.67E-01
IL19	−0.18	0.15	2.31E-01
ITGB6	0.49	0.16	3.17E-03
LAIR1	NA	NA	NA
LEFTY2	NA	NA	NA
LGALS9	NA	NA	NA
LILRB4	NA	NA	NA
POLR2F	NA	NA	NA
RP2	NA	NA	NA
STC2	NA	NA	NA
TNFRSF8	−0.36	0.21	9.29E-02

The results for proteins without cis-SNPs are filled with NA values.

Supplementary Table 28. Heterogeneity test of protein on severe COVID.

Protein	No. of SNPs	*Q* statistic	*P*-val of *Q* statistic
ADGRG2	6	42.14	5.51E-08
AMY2B	9	72.35	1.67E-12
CCL15	4	11.65	8.70E-03
CD109	6	6.06	3.01E-01
CD209	8	11.84	1.06E-01
CD34	3	3.65	1.61E-01
CDH15	4	2.48	4.79E-01
CKMT1A_CKMT1B	6	26.02	8.86E-05
CX3CL1	7	43.16	1.09E-07
ERBB4	16	44.94	7.82E-05
FGF19	5	11.67	1.99E-02
GOLM2	9	62.23	1.70E-10
ICAM5	6	16.57	5.38E-03
ISLR2	8	23.48	1.40E-03
KEL	16	72.11	1.88E-09

KLK1	13	42.97	2.28E-05
LAMP3	10	144.56	1.18E-26
LEFTY2	4	22.69	4.69E-05
LGALS4	3	5.58	6.14E-02
LGALS8	6	15.10	9.96E-03
MNDA	1	NA	NA
MUC13	4	24.43	2.03E-05
NRCAM	6	27.86	3.88E-05
PECAM1	8	13.05	7.08E-02
PODXL	6	14.95	1.06E-02
PTPRM	8	30.44	7.87E-05
REG1A	5	35.36	3.91E-07
REG1B	7	49.90	4.93E-09
SCG2	4	31.72	6.01E-07
SCGN	5	16.97	1.96E-03
SEMA4C	4	3.36	3.40E-01
SFTPD	11	98.80	9.47E-17
TDGF1	1	NA	NA
VAMP5	2	NA	NA
VTCN1	2	NA	NA

Supplementary Table 29. Heterogeneity test of protein on COVID hospitalization.

Protein	No. of SNPs	*Q* statistic	*P*-val of *Q* statistic
ADGRG1	9	57.47	1.46E-09
ADGRG2	6	92.04	2.50E-18
AMY2A	8	95.88	7.64E-18
AMY2B	9	99.13	6.44E-18
CA4	9	77.39	1.64E-13
CCL15	4	4.34	2.27E-01
CD109	6	23.11	3.22E-04
CD209	8	23.60	1.34E-03
CD34	3	11.84	2.68E-03
CDH15	4	6.17	1.04E-01
CLEC14A	8	73.72	2.60E-13
CTSS	7	84.18	4.89E-16
CX3CL1	7	47.96	1.20E-08
EFNA1	4	2.57	4.63E-01
ERBB4	16	79.76	7.72E-11
FGF19	5	13.19	1.04E-02
GOLM2	9	89.74	5.25E-16
GP2	8	86.98	5.15E-16
ICAM2	10	70.56	1.18E-11
ICAM5	7	42.99	1.17E-07
ISLR2	8	41.26	7.22E-07
KLK1	13	23.58	2.32E-02
LEFTY2	4	28.70	2.59E-06
LGALS4	3	7.15	2.81E-02
MET	20	77.14	5.75E-09
NELL2	14	89.19	2.00E-13
NRCAM	7	53.77	8.22E-10

PECAM1	8	26.75	3.69E-04
PODXL	6	19.27	1.71E-03
PRSS27	18	100.56	6.99E-14
PTPRM	8	44.44	1.75E-07
REG1A	5	69.14	3.46E-14
REG1B	7	85.00	3.30E-16
SCG2	4	55.74	4.78E-12
SCGN	5	13.73	8.21E-03
SELE	9	52.18	1.55E-08
SEMA3F	5	47.19	1.39E-09
SEMA4C	5	18.87	8.34E-04
SFTPA2	6	5.05	4.10E-01
SFTPD	11	55.32	2.75E-08
TDGF1	1	NA	NA
TGFBR2	8	33.02	2.63E-05
VAMP5	2	NA	NA

Supplementary Table 30. Heterogeneity test of protein on SARS-CoV-2 infection.

Protein	No. of SNPs	*Q* statistic	*P*-val of *Q* statistic
ADAM15	8	17.87	1.26E-02
ADGRG1	10	180.92	3.25E-34
ADGRG2	6	260.26	3.46E-54
AMY2A	8	331.37	1.19E-67
AMY2B	9	342.70	3.28E-69
BST2	11	351.39	2.02E-69
CA4	11	306.13	7.87E-60
CCL15	4	30.43	1.12E-06
CCL28	18	192.87	7.72E-32
CD109	6	70.21	9.24E-14
CD209	8	40.82	8.77E-07
CD34	3	28.96	5.16E-07
CD58	12	86.67	7.46E-14
CDH17	12	391.15	4.63E-77
CKMT1A_CKMT1B	8	161.54	1.52E-31
CLEC14A	9	126.33	1.63E-23
CTSS	7	275.64	1.34E-56
CX3CL1	8	158.83	5.66E-31
DPP10	5	190.95	3.31E-40
DRAXIN	17	68.97	1.51E-08
EFNA1	4	7.70	5.27E-02
F2R	8	185.14	1.60E-36
FCGR2B	4	310.89	4.36E-67
FGF19	5	12.76	1.25E-02
FGFR2	8	328.93	3.98E-67
FLT4	12	203.99	1.10E-37
FOLR1	8	109.09	1.42E-20
GKN1	4	49.76	8.98E-11
GOLM2	8	238.79	6.71E-48
ICAM2	10	166.16	3.89E-31
ICAM5	7	161.01	3.62E-32

IDS	3	333.48	3.86E-73
ISLR2	8	59.19	2.19E-10
ITGA6	7	176.19	2.18E-35
ITGB1	7	184.40	3.93E-37
KEL	17	223.16	1.59E-38
KLK1	13	67.09	1.12E-09
LEFTY2	3	109.90	1.37E-24
MNDA	1	NA	NA
MUC13	4	109.02	1.78E-23
NELL2	15	178.72	1.18E-30
NME3	14	227.26	3.30E-41
NRCAM	7	165.94	3.27E-33
PECAM1	8	57.85	4.04E-10
PLAT	4	16.33	9.69E-04
PODXL	6	68.83	1.80E-13
PTPRM	8	72.39	4.86E-13
REG1A	6	291.32	7.34E-61
REG1B	7	326.00	2.18E-67
S100A16	3	38.70	3.96E-09
SCARF2	8	180.12	1.84E-35
SCG2	4	117.26	3.00E-25
SEMA3F	6	168.95	1.22E-34
SEMA4C	6	45.21	1.31E-08
SFTPD	12	65.80	7.62E-10
SLITRK2	11	22.54	1.26E-02
SPINK5	7	40.67	3.37E-07
STC1	6	197.29	1.08E-40
TDGF1	2	NA	NA
TGFBR2	8	108.75	1.67E-20
ULBP2	8	327.41	8.38E-67
VAMP5	2	NA	NA
VTCN1	2	NA	NA

Supplementary Table 31. Heterogeneity test of protein on healthspan.

Protein	No. of SNPs	*Q* statistic	*P*-val of *Q* statistic
FOXO3	1	NA	NA
GPNMB	6	113.95	5.99E-23
HLA-DRA	3	1.29	5.24E-01
PLA2G7	8	15.97	2.54E-02

Supplementary Table 32. Heterogeneity test of protein on father's attained age.

Protein	No. of SNPs	*Q* statistic	*P*-val of *Q* statistic
AGRP	9	68.91	8.08E-12
CA11	2	NA	NA
CCN1	11	115.51	4.10E-20
CD27	7	62.53	1.38E-11
CD74	6	36.35	8.08E-07
CDH1	6	11.08	4.98E-02
CEACAM21	2	NA	NA

CPE	6	30.68	1.08E-05
CXCL13	7	54.56	5.70E-10
CXCL9	5	25.75	3.55E-05
F3	8	4.36	7.37E-01
FASLG	18	53.29	1.29E-05
FES	1	NA	NA
FURIN	3	4.72	9.44E-02
GCNT1	4	48.76	1.46E-10
GP1BA	14	41.85	6.94E-05
GRN	10	15.78	7.16E-02
GZMB	8	39.66	1.46E-06
IGFBP1	2	NA	NA
KIR2DL3	2	NA	NA
LAIR1	3	25.64	2.71E-06
LDLR	15	53.37	1.65E-06
LEFTY2	3	14.54	6.97E-04
LGALS9	3	35.69	1.78E-08
LILRB4	2	NA	NA
PAG1	6	36.71	6.83E-07
PCSK9	8	27.93	2.26E-04
POLR2F	1	NA	NA
RP2	2	NA	NA
SIT1	6	43.76	2.59E-08
VCAM1	10	59.77	1.48E-09
VSTM2L	2	NA	NA

Supplementary Table 33. Heterogeneity test of protein on mother's attained age.

Protein	No. of SNPs	*Q* statistic	*P*-val of *Q* statistic
CDH1	6	3.37	6.43E-01
CDH17	10	15.02	9.05E-02
CDHR2	6	283.66	3.26E-59
CPE	6	17.55	3.56E-03
CXADR	5	7.74	1.01E-01
CXCL9	5	17.38	1.63E-03
F3	8	9.46	2.21E-01
FOXO3	1	NA	NA
GFAP	7	252.84	1.01E-51
IL19	3	5.17	7.52E-02
ITGB6	9	24.14	2.17E-03
LAIR1	3	10.44	5.42E-03
LEFTY2	3	3.51	1.73E-01
LGALS9	3	17.25	1.79E-04
LILRB4	2	NA	NA
POLR2F	1	NA	NA
RP2	2	NA	NA
STC2	4	11.75	8.29E-03
TNFRSF8	7	38.97	7.25E-07
